# Diels–Alder Reactions in [Pd_6_L_4_]^12+^ Metallocages: The Key Roles of Preorganization and Confinement

**DOI:** 10.1021/acs.inorgchem.6c01395

**Published:** 2026-06-30

**Authors:** Giuseppe Sciortino, Irina Cuesta, Gantulga Norjmaa, Gregori Ujaque

**Affiliations:** Departament de Química and Red ORFEO−CINQA, 16719 Universitat Autònoma de Barcelona, Til·lers, sn, Cerdanyola del Vallès, Catalonia 08193, Spain

## Abstract

The Diels–Alder reaction between maleimide and naphthalene-based compounds typically requires harsh conditions or electronic activation, such as the presence of Lewis acids. Fujita and co-workers, however, demonstrated that this reaction can proceed smoothly in confined spaces inside the [Pd_6_L_4_]^12+^ metallocage. In the present work, a computational study combining several techniques is performed in solution and within the metallocage to elucidate the origin of the rate acceleration as well as the regio- and stereoselectivity observed. MD simulations reveal how the reactants accommodate and behave inside the cavity and allow identification of pro-reactive conformations. QM/MM calculations provide Gibbs energy profiles for the reactions in solution and within the metallocage. The roles of confinement, microsolvation, and the metallocage environment are evaluated, showing that reaction acceleration mainly arises from confinement-induced preorganization of the reactants. The analysis also provides a unified mechanistic explanation for the substrate-dependent reactivity observed experimentally.

## Graphical abstract

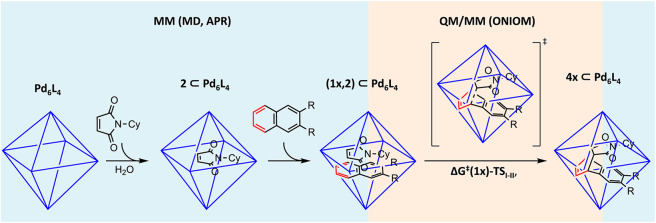

## Introduction

Supramolecular chemistry is an interdisciplinary field with very broad applications in many areas.^1^ Among those, their use as supramolecular catalysts is one of the most attractive and stimulating.^2^ Molecular compounds that can mediate or catalyze chemical reactions in their cavities are also named molecular flasks.^3,4^

Numerous supramolecular hosts have been synthesized during the past years.^5–10^ Among them, one of the most exciting developments is the so-called metallocages, also named Supramolecular Organometallic Complexes (SOCs)^11^ or Metal–Organic Cages (MOCs) or Coordination Cages.^12,13^ The capability for tailoring the cavities of the MOCs has led to develop many applications containing catalysis.^8,14–16^ In this sense, several metallocages have been designed as supramolecular catalysts,^17–21^ including Fujita’s^22^ octahedral [Pd_6_L_4_]^12+^, Raymond’s^23^ tetrahedral [Ga_4_L_6_]^12–^, and Lusby’s^24–26^ lantern [Pd_2_L_4_]^4+^ metallocages, among others.^27–30^

The use of computational methods to analyze chemical reactivity is widespread,^4,31–33^ although their application to reactions accelerated in confined spaces is still comparatively scarce. For organic hosts, examples exist for many receptors,^34–36^ cavitands,^37–40^ capsules,^41–44^ etc. For MOCs, studies on the Raymond [Ga_4_L_6_]^12–^ metallocage include C–C reductive elimination,^45–49^ Nazarov^50,51^ and Prins cyclizations,^52^ orthoformate hydrolysis,^53^ and aza-Cope rearrangement.^54^ For Fujita’s [Pd_6_L_4_]^12+^ metallocage, computational analysis has been reported for amide hydrolysis^55^ and the Diels–Alder reaction of anthracene.^56,57^ For Lusby’s [Pd_2_L_4_]^4+^ metallocage, the Diels–Alder and Michael addition reactions have also been examined.^24–26^

In this study, we investigate the intriguing reactivity observed in the Diels–Alder (DA) reaction between a set of R,R-ethylnaphthalenes (**1**) and N-cyclohexylmaleimide (**2**), in the presence of cationic [Pd_6_L_4_]^12+^ metallocage (**3**), Scheme 1.^3,58,59^ The DA reaction of naphthalene-based dienes typically requires either electronic activation or harsh conditions. Nevertheless, in the presence of the Fujita’s [Pd_6_L_4_]^12+^ cage, the reaction proceeds smoothly under relative mild conditions.

**Scheme 1:**
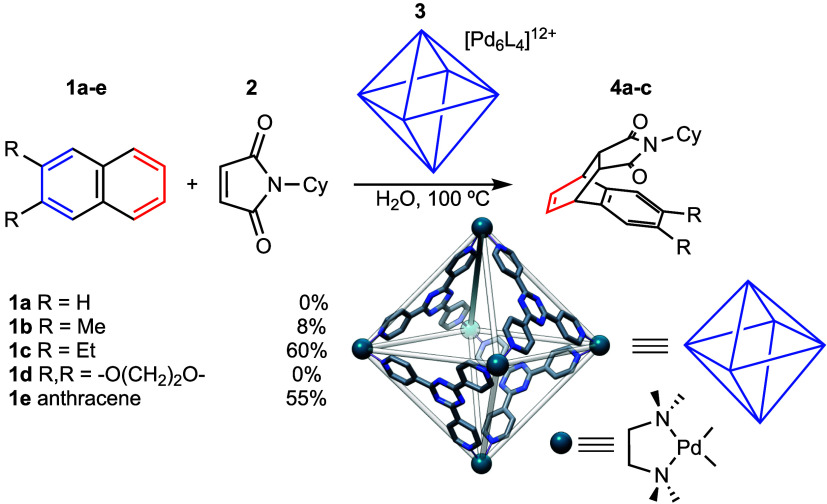
Diels–Alder Reaction of Naphthalenes 1a-e with Maleimide 2 within the [Pd_6_L_4_]^12+^ Metal-Organic Cage **3** Analyzed in this Work, with NO_3_^–^ as Counterions in the Experimental Work

The regioselectivity in solution differs from that found inside the metallocage. For example, for **1b** and **1c** (Table 1), the lowest computed barrier in solution corresponds to the exo pathway, whereas within the metallocage, the lowest barrier is associated with the endo pathway. Moreover, the preferred product also depends on the substituents on the naphthalene units. The different theoretical pathways and the corresponding regioisomeric outcomes are listed in Scheme 2. The reaction proceeds with yields ranging from 8% for methyl-substituted naphthalene **1b** to 60% for the ethyl-substituted naphthalene **1c**. In contrast, for unsubstituted naphthalene, **1a**, or when including a more constrained substituent such as *R*,*R* = −O­(CH_2_)_2_O–, **1d**, the reaction does not take place (Scheme 1). In the case of anthracene, the DA reaction occurs under much milder conditions, even in solution; its reactivity has been studied by Xu and co-workers using both bowl-shaped and octahedral cages.^56,57^

**Scheme 2:**
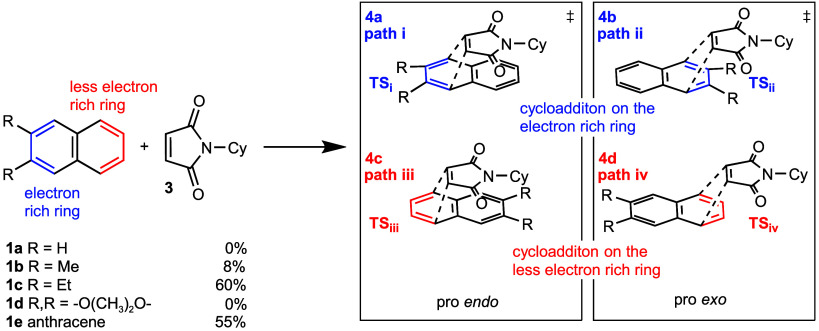
General Pathways and Yields for the Diels–Alder Cycloaddition between Substituted Naphthalene and Maleimide

**Table 1:** Gibbs Energy Barriers for the Diels–Alder Reaction between Substituted Naphthalenes **1a**–**e** and N-Cyclohexylmaleimide **2** in Solution (H_2_O) through the Four Possible Pathways (Scheme 1)­^a^

**reac.**	**paths**					
	without explicit H_2_O				H_2_O^b^	H_2_O^b^
	**TS**_**i**_	**TS**_**ii**_	**TS**_**iii**_	**TS**_**iiv**_	**TS**	**TS**
**1a** ^c^			40.7	**40.5**	39.6	37.4
**1b** ^c^	39.1	**38.9**	40.2	40.9	38.1	36.4
**1c** ^c^	40.3	**39.4**	40.7	40.8	38.4	36.2
**1d** ^c^	**40.1**	40.9	40.2	41.1	39.1	36.7
**1e** ^c^ ^,^ ^d^	38.3	39.2	**29.0** ^e^			


a *T* = 100 °C for 1**a–d** and 52 °C for 1**e**.

b The pathway calculated corresponds to the lowest barrier without explicit water molecules shown in bold. Water molecules are shown in Scheme 3.

c Δ*G* with respect to reactants at infinite distance for the bulk simulations (H_2_O molecules have been included, cyclohexylmaleimide).

d Toluene solvent, at 52 °C.

e Internal Diene

The aim of the present work is to investigate the factors driving the rate enhancement, the origin of the selectivity, and, critically, the reasons behind the lack of reactivity observed for certain dienes. Although a recent computational investigation has examined specific aspects of the Diels–Alder activity of the Fujita’s [Pd_6_L_4_]^12+^ metallocage, focusing on 9-hydroxymethylanthracene and selected mechanistic features, a unified explanation of substrate dependency and nonreactivity is still lacking.^56,57^ The present work directly extends and complements this study by providing a comprehensive mechanistic analysis that systematically rationalizes both reactive and nonreactive systems. The reaction is analyzed both in bulk solution (H_2_O) and inside the [Pd_6_L_4_]^12+^ octahedral metallocage, **3**. We combine Molecular Dynamics (MD) simulations to characterize the behavior of the reactants and the metallocage in aqueous solution with quantum mechanical (QM) and QM/MM calculations to draw Gibbs energy reaction profiles (see Computational details). Identifying the factors governing effective reactivity within the cavity of [Pd_6_L_4_]^12+^ is critical for the rational design of supramolecular catalysts and, more broadly, for advancing our understanding of chemical reactivity in confined spaces.

## Computational Details

### QM and QM/MM Calculations

Full DFT simulations were carried out in Gaussian 16 ^60^ using the B3LYP-D3 ^61–63^ functional, including the Grimme’s correction for dispersion. The Pd atom was described using the scalar-relativistic Stuttgart–Dresden SDD pseudopotential along with its associated double-ζ basis set; a set of *f* polarization functions was also added.^64^ For H, C, O, and N, the 6–31G­(d,p) basis set was employed.

QM/MM calculations were performed on the ONIOM scheme implemented in Gaussian 16.^60^ The reactants for the DA reaction (naphthalene, 2,3-ethylnaphthalene, 2,3-methylnaphthalene, 2,3-(ethylenedioxy)­naphthalene, or anthracene + *N*-cyclohexylmaleimide + *n*H_2_O with *n* = 0–2) were described at the DFT level (B3LYP-D3 functional combined with the 6–31g­(d,p) basis set (BS1)), whereas the metallocage was described at the MM level including solvent effect. The General Amber Force Field (GAFF2) was employed for the MM part; Pd-bonding force constants and equilibrium parameters were obtained through the Seminario method,^72^ using the MCPB.py module.^65^ Metal center parametrization was based on the optimized geometries and harmonic frequencies of the full metallocage obtained at the DFT level. The partial charges of the MM region were obtained by RESP calculations and incorporated into the QM Hamiltonian by the electronic embedding framework.

Frequency calculations were completed for all optimized geometries to confirm the nature of the stationary points as either transition states or minima. All simulations were optimized in solvent by the PCM continuum model for water, representing the solute–solvent boundary as solvent accessible surface (SAS). All transition states were optimized as first-order saddle points at the corresponding DFT or QM/MM level of theory, and their nature was confirmed by the presence of a single imaginary frequency associated with the expected bond-forming reaction coordinate. The connection between transition states and intermediates was confirmed by the usual intrinsic reaction coordinate (IRC) and subsequent optimization to minima. Energies of the lowest energy pathways were refined by single-point calculations on the optimized geometries, using the extended triple-ζ *def2*-TZVP (BS2).^66,67^ The final energies given are relative to qh-Gibbs (quasi-rigid-rotor-harmonic-oscillator)^68^ energies in solution at 373 K and 1 M^69^ obtained with a frequency cutoff of 100 cm^–1^,^70^ by adding the thermal and entropic corrections computed with 6–31g­(d,p) to the electronic energy computed with *def2*-TZVP.

### Molecular Dynamics (MD) Simulations

The molecular dynamics (MD) simulations were performed using the PMEMD module implemented in Amber18,^71^ exploring the behavior of the empty [Pd_4_L_6_]^12+^ cage in solution and of the [Pd_4_L_6_]^12+^ cage along with both reactants (*N*-cyclohexylmaleimide and 2,3-*"R"* naphthalene) inside and outside the cage. The General Amber Force Field (GAFF2) was used for the organic ligands, while Pd-bonding force constants and equilibrium parameters were obtained through the Seminario method,^72^ using the MCPB.py module.^65^ Metal center parametrization was based on the optimized geometries and harmonic frequencies of the full metallocage obtained at the DFT level (*vide supra*). Parameters were the same as those described in the previous section.

MD simulations were carried out by solvating the system (reactants + metallocage) in a cubic box of TIP3P explicit water molecules. The 12+ charge of the metallocage was neutralized with the proper number of Cl^–^ anions as a model for the experimentally employed nitrate counteranions. Analysis of the trajectories shows that no counterion is encapsulated inside the metallocage during the simulations. The box dimension was set to fit the experimental Pd_4_L_6_ concentration (∼10 mM) corresponding to 55 × 55 × 55 Å. The resulting systems were then subjected to a minimization followed by thermalization, increasing the temperature from 0 to 300 K (time step of 20 ps), followed by 100 ns of NPT production using the Langevin thermostat under periodic boundary conditions with an electrostatic cutoff of 10.0 Å and the Particle Mesh Ewald method for long-range electrostatic interactions.^73^ The cavity and the solvent molecules inside it along the MD trajectory were analyzed using an in-house modification of the C3 code.^74^

In order to determine Δ*G*_bind_, we applied the Attach-Pull-Release method (APR method)^75^ starting from four different 500 ps pre-equilibrated systems exploring the binding of each substrate (*N*-cyclohexylmaleimide or 2,3-“*R*” naphthalene) into the empty cage or into the cage containing the other reactant. The simulation details are as follows: minimization with maxcyc = 50,000, 1 ps at 10K, 100 ps heating to 298.15 K, equilibration with 250 ps, and production with 2.5–25 ns for each window, depending on the standard error of the mean of the restraint forces.

The distance force constant was 5.0 kcal/mol·Å^2^ between the one atom of the guest and one of the dummy atoms placed at the bottom of the metallocage. The distance between these two atoms is changed by 0.4 Å in every simulation window from 6 Å (guest inside) to 24 Å (guest outside) during the simulation. The angle force constants were 100.0 kcal/mol·rad^2^ for two host restraints and two guest restraints as the default value in the APR method.^75^

The present computational strategy is designed to provide a mechanistic comparison between the reaction in bulk solution and under confinement, combining APR Gibbs energies for encapsulation with MD sampling of proreactive conformations and QM/MM Gibbs energy profiles for the chemical step.

## Results and Discussion

The Diels–Alder reaction between *N*-cyclohexylmaleimide, **2**, and several naphthalene-based dienes with 2,3-*R* substituents (*R* = H **1a**, *R* = Me, **1b**, *R* = Et, **1c**, *R*,*R* = −O–CH_2_–CH_2_–O–, **1d**, and anthracene, **1e**) is computationally investigated. The first subsection includes the study of the reaction in bulk solution, whereas the second one describes the study within the metallocage’s cavity in the same solvent.

### Diels–Alder Reaction in Solution

The reaction between maleimide **2** and substituted naphthalenes **1a**–**e** can proceed through four different pathways, depending on the relative orientation of the reactants, pro-*endo* or pro-*exo*, Scheme 2.^3^

The naphthalene itself is an inert substrate toward the Diels–Alder reaction; however, under harsh conditions of pressure, temperature, and excess of maleimide in organic solvents, related systems show a slight *exo* stereoselectivity.^76–79^ In contrast, for anthracene, the reaction becomes feasible under milder conditions.^80^ It is well-known that the presence of electron-donating alkyl groups on naphthalene favors cycloaddition at the more electron-rich substituted ring.

The computed Gibbs energy barriers for the cycloaddition according to the four pathways described in Scheme 2 are given in Table 1.

The Gibbs energy barrier for the reaction of maleimide with the selected naphthalenes **1a**–**d** is similar for all systems (38.9 to 41.1 kcal/mol). For most of these substrates, the *exo* pathway is slightly favored. In all of these cases, however, the reaction is too energy-demanding to proceed even at 100 °C. These results are consistent with experimental observations, which show that none of the Diels–Alder reactions involving these naphthalene-based reactants takes place in solution. For compound **1e**, the computed barrier of 29.0 kcal·mol^–1^ in toluene agrees with experiment^80^ and with previous calculations.^56^ Anthracene **1e** was included as a control case for the computational approach, allowing direct comparison with the available experimental data both in solution and within the metallocage.

The effect of microsolvation on the Diels–Alder reaction can be relevant, as demonstrated by Head-Gordon and co-workers.^81^ In our work, microsolvation was considered by including one or two explicit H_2_O molecules in the model (Scheme 3; note that no additional solvent molecules were added, in order to retain a model directly comparable to the molecular arrangements found inside the metallocage; *vide infra*).

**Scheme 3:**
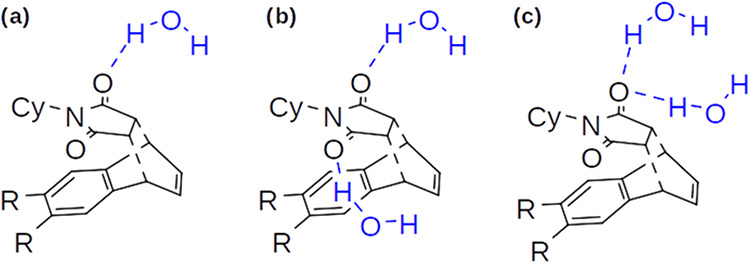
Models of Microsolvation Considered: (a) One Water Molecule, (b) Two Water Molecules, One Bound to Each Carbonyl Group, (c) Two Water Molecules Bound to the Same Carbonyl Group

For the system containing two explicit water molecules, one configuration has each H_2_O molecule forming a H-bond with a different CO group (Scheme 3b), whereas in the other configuration, both H_2_O molecules are bound to the same CO of **2** (Scheme 3c). The latter arrangement was the most stable.

Among the four possible pathways, **TSi-iv**, for those exhibiting the lowest energy barrier in the bare system (shown in bold in Table 1), were also computed in the presence of one and two explicit water molecules (last two columns in Table 1). Microsolvation effects in water decrease the barriers by up to ∼3.5 kcal·mol^–1^, in agreement with previous studies,^81^ although the observed effect is not large enough to make these reactions feasible in solution.

For those dienes that react inside the metallocage (**1b**, **1c**, and **1e**), the corresponding Gibbs energy barrier in solution was further analyzed. The overall barrier was decomposed into two contributions, one associated with the formation of the van der Waals complex (i.e., association of the reactants) and the other corresponding to the transition state (Table 2).

**Table 2:**
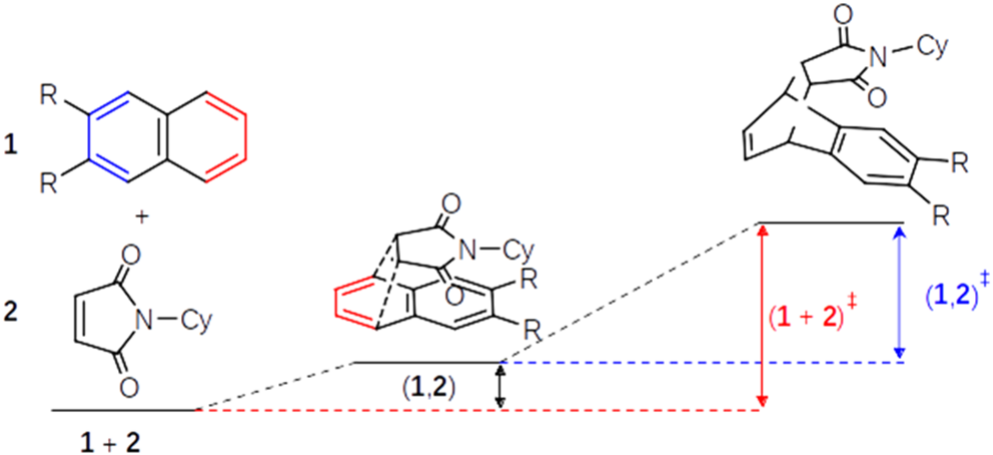
Contributions to Δ*G* for the DA Reaction in Solution (in kcal·mol^–1^)

	**1b**	**1c**	**1e**
**1**+**2**	0.0	0.0	0.0
(**1**,**2**)	5.8	6.1	6.8
(**1**,**2**)^‡^	30.7	30.1	22.2
(**1**+**2**)^‡^	36.5	36.2	29.0
**exp.**			28.0

In all cases, the first term is very similar (∼6 kcal/mol), whereas the barrier depends on the nature of the reactants. For the naphthalene-based substrates, **1b** and **1c**, the barriers are 36.5 and 36.2 kcal·mol^–1^, whereas for the anthracene, **1e**, it is 29.0 kcal·mol^–1^, respectively.

### Diels–Alder Reaction Inside the Metallocage

The experimental yields of the Diels–Alder reaction within metallocage 3 depend on the nature of the naphthalene-based diene, ranging from 0% for **1a** and **1d** to 8% for **1b** and up to 60% for **1c**.^58^ The origin of this difference is striking because in some cases (i.e., **1c** vs **1d**) their chain length and volume are quite similar. Interestingly, in all cases, the selectivity is reversed relative to reaction in bulk; the cycloaddition takes place on the less electron-rich ring, with exclusive formation of the *endo* adduct.

When switching from bulk solvent to the confined space of a metallocage, several aspects must be considered: (i) the energy associated with the reactants’ encapsulation; (ii) the relative stability and mobility of the pro-reactive conformation of the encapsulated reactants; and (iii) the influence of confinement on the energy barrier and selectivity of the process. With these aspects in mind, we set up a multistep computational strategy combining different levels of theory, each suited to the specific phenomenon under investigation, following previous studies.^45–47,50,52^ The first section describes classical MD simulations to analyze the behavior of the reactants inside the cavity, along with APR calculations to obtain Δ*G*_binding_ of the species involved. The second subsection presents QM/MM calculations aimed at determining Gibbs energy barriers and elucidating the mechanism governing the selectivity of the process.

### MD Simulations: Binding of Reactants to [Pd_6_L_4_]^12+^

The Δ*G*_bind_ values for the reactants’ encapsulation within the metallocage **3** obtained using the APR method^75,82^ are gathered in Table 3. The total binding Gibbs energy (Δ*G*_bind,TOT_) corresponds to the binding of two consecutive reactants. Encapsulating naphthalene **1c** followed by maleimide **2**, forming (**1c**,**2**)⊂**3**, or the reverse order, forming (**2**,**1c**)⊂**3**, gives rise to very similar binding energies within associated error (−15.8 ± 1.0 and −16.0 ± 1.0 kcal·mol^–1^, respectively; Table 3).

**Table 3:** Δ*G*_bind_ for Encapsulating One and Two Consecutive Reactants and for **4c** Product to Metallocage **3** (in kcal·mol^–1^)

**1** ^**st**^ **guest**	**Δ*G***_**bind**_	**2** ^**nd**^ **guest**	**Δ*G***_**bind**_	**Δ*G***_**bind**,**TOT**_	**yield** ^a^
**2**	–3.7 ± 0.3	**1a**	–5.6 ± 0.4	–9.3 ± 0.7	0%
		**1b**	–5.9 ± 0.5	–9,6 ± 0.8	8%
		**1c**	–12.1 ± 0.7	–15.8 ± 1.0	60%
		**1d**	–7.3 ± 0.6	–11.0 ± 0.9	0%
		**1e**	–9.7 ± 0.6	–13.0 ± 1.0	55% (98%)^b^
**1c**	–6.4 ± 0.4	**2**	–9.6 ± 0.6	–16.0 ± 1.0	
**4c**	–16.6 ± 0.6				

a At 100 °C, 5–16h.

b Relative to the 9-hydroxymethylanthracene ⊂ **3**.^56^

This agreement exhibits the consistency of the methodology employed. These values also indicate that encapsulation of the second reactant becomes more favorable once the first is already bound. This behavior is observed for all reactants analyzed: the Δ*G*_bind_ of maleimide **2** changes from −3.7 ± 0.3 to −9.6 ± 0.6 kcal·mol^–1^, whereas the Δ*G*_bind_ of naphthalene **1c** changes from −6.4 ± 0.4 to −12.1 ± 0.7 kcal·mol^–1^, respectively. A comparison of Δ*G*_bind_ values among the naphthalene-based reactants shows that the binding strength increases with molecular size. Upon prior encapsulation of maleimide **2**, the Δ*G*_bind_ values for **1a**, **1b**, and **1c** are −5.6 ± 0.4, −5,9 ± 0.5, and −12.1 ± 0.7 kcal·mol^–1^, respectively. This trend is consistent with the expected behavior for the encapsulation of apolar molecules in a highly polar solvent such as water. For **1c**, the Δ*G*_bind_ is particularly high, but remains consistent with the Δ*G*_bind,TOT_ value obtained through two different binding pathways.

The Δ*G*_bind_ computed for the product, **4**⊂**3**, is −16.6 ± 0.6 kcal·mol^–1^ (Table 3). This value is similar to the Δ*G*_bind,TOT_ of both reactants, although the product complex is slightly more favorable. These results indicate that replacing the product with “diene + dienophile” reactants is thermodynamically feasible, though not favorable. Therefore, the Gibbs energy associated with product displacement by the reactants prevents the metallocage from operating in consecutive catalytic cycles, as observed experimentally.

### MD Simulations: Behavior of Encapsulated Reactants

To analyze the behavior of encapsulated “diene + dienophile” reactants, we performed 100 ns MD simulations for each reactant pair “diene + dienophile”. The motion of the encapsulated dienes is relatively constrained, with the naphthalene moiety of **1b**, **1c**, and **1d** mostly remaining stacked against one of the aromatic walls of the metallocage, whereas **1a** shows greater mobility freedom. Maleimide **2**, in turn, generally tends to remain on top of the naphthalene moiety for all dienes (Figures S2–S5).

Analysis of the water molecules inside the cavity shows that up to three molecules can be accommodated in the case of **1c** and up to four for **1a**, **1b**, and **1d** (Table 4). However, the average number of water molecules observed inside the cage is between 1 and 2. These findings prompted us to employ computational models, including one or two water molecules, when analyzing the energy profiles both inside the cavity (*vide infra*) and in solution (*vide supra*); therefore, the microsolvated models are consistent and comparable with one another. Analysis of the trajectories shows that when a water molecule is present inside the cavity, it typically forms a hydrogen bond with one of the oxygen atoms of the maleimide.

**Table 4:** Analysis of Reactants and Number of Water Solvent Molecules (*n*) Inside Metallocage **3** along a 100 ns MD Simulation

**species**	***R*,*R***	***n***_**max,H2O**_	***n***_**average,H2O**_	**pop. (%)** ^a^
**(2,1a)** ⊂ **3**	**H**	4	2.3	9.6
**(2,1b)** ⊂ **3**	**Me**	4	1.6	23.3
**(2,1c)** ⊂ **3**	**Et**	3	1.0	57.4
**(2,1d)** ⊂ **3**	**–O(CH**_**2**_ **)**_**2**_ **O–**	4	1.1	6.5

a Population describes the percentage of molecular arrangements that can be defined as pro-reactive during the MD simulation, defined using a C–C distance cutoff of 5 Å for both C···C forming bonds.

When a second water molecule enters the cavity, it alternates between interacting with the same or the other oxygen atom of the maleimide (Scheme 3). The third and fourth H_2_O molecules generally interact with the maleimide or form a water chain with the already encapsulated water molecules.

Fujita and co-workers suggested that the formation of a pro-reactive conformation is crucial for the reaction outcome.^58^ We defined a pro-reactive conformation as a molecular arrangement in which the distance between the two carbon atoms involved in the formation of a new C–C bond is shorter than 5.0 Å, with both forming C–C bonds simultaneously within this distance. These parameters ensure that the reagents adopt the proper diene-dienophile orientation for the reaction to take place. MD simulations allow us to determine whether pro-reactive conformations are accessible and to evaluate their population along the simulation. The results are gathered in Table 4.

The pro-reactive conformation observed for **1a**–**d** is characterized by a pro-endo arrangement of the reactants, with the maleimidic alkene (dienophile) pointing toward the diene of the nonsubstituted ring (path iii; Scheme 2). Within the time scale of the MD simulations, only pro-endo arrangements are observed as pro-reactive conformations, whereas alternative arrangements leading to other isomers are not detected. A convergence examination based on Principal Component Analysis shows recurrent visits to the main conformational basins throughout the trajectory, although rare alternative arrangements cannot be completely excluded (see the Supporting Information for further details). The population of the pro-reactive conformation for **1a** and **1d** is 9.6 and 6.5%, respectively, whereas for **1b** and **1c** it increases to 23.3 and 57.4%, respectively (Table 4). For **1b** and **1c**, these populations correlate with the observed yields of 8 and 60%, respectively. A complementary NCI analysis reveals that host–guest interactions play a major role in the stabilization of the confined complexes, while pro-reactive arrangements are characterized by enhanced substrate–substrate interactions, in line with the key role of confinement-induced preorganization in promoting reactivity within the metallocage (see the Supporting Information for further details). Indeed, the higher the population of the pro-reactive conformation, the higher the observed yield. Fujita and co-workers hypothesized that the key role of the cage is related to preorganization and packing effects,^58^ and the observations obtained from the MD simulations support this hypothesis.

### QM/MM Analysis of Gibbs Energy Profiles

The mechanism for the Diels–Alder reaction inside metallocage **3** was computed for the reactions between maleimide **2** and dienes **1a**–**e** at the DFT/MM level. The Gibbs energies for encapsulated intermediates and transition states for pathways i–iv are gathered in Table S1. We focus our analysis on paths i and iii because pro-endo pathways are the most favored ones inside the cage. For these pathways, we also computed the reaction barriers, including one or two water molecules in the model, as suggested by MD simulations, which indicate that these are the most frequently sampled water occupancies inside the metallocage for the reactant complexes. Additional calculations with larger microsolvation show that the inclusion of a third water molecule does not significantly modify the barrier, whereas the inclusion of the fourth water molecule leads to a higher barrier (Table S4). Thus, although microsolvation affects the barriers, the overall mechanistic picture remains dominated by confinement-induced preorganization. Compared with the “naked” reactants inside the cavity, the inclusion of explicit water molecules reduces the barrier for most dienes around 3 kcal·mol^–1^ (Tables 5 and S2).

**Table 5:** Δ*G* ^‡^ and Δ*G*_r_ (kcal·mol^–1^) for the Diels–Alder Reaction between Substituted Naphthalenes **1a**–**e** with N-Cyclohexylmaleimide **2** in Metallocage **3** through Paths i and iii^a^ ^,^ ^b^

**diene 1x**		**[1x,2,(H**_**2**_ **O)** *_**n**_* **] ⊂ 3**			**[1x,2,(H**_**2**_ **O)** *_**n**_* **]⊂3**		
		*n* = 0	*n* = 1	*n* = 2	*n* = 0	*n* = 1	*n* = 2
			**path i**			**path iii**	
**1a**	**Δ*G*** ‡_**I–II**_ ^c^				31.5	27.5	27.0^d^
	**Δ*G***_**r**_				5.0	6.0	4.2
**1b**	**Δ*G*** ‡_**I–II**_ ^c^	31.5	27.6	29.1^d^	33.0	28.2	27.9
	**Δ*G***_**r**_	8.7	3.8	1.5	11.3	5.9	6.6
**1c**	**Δ*G*** ‡_**I–II**_ ^c^	32.3	29.9	29.7	34.9	29.4	29.7^d^
	**Δ*G***	6.7	3.0	7.1	11.2	1.6	7.1
**1d**	**Δ*G*** ‡_**I–II**_ ^c^	35.9	32.1	29.2^d^	30.7	33.0	29.3^d^
	**Δ*G***_**r**_	9.0	9.9	7.2	10.3	5.9	7.6
**1e**	**Δ*G*** ‡_**I–II**_ ^c^			28.4			29.7
	**Δ*G***_**r**_			4.5			4.4

a *T* = 100 °C. Calculations at the ONIOM­(DFT:MM) level.

b Only the lowest conformations were reported.

c Δ*G* ^‡^ with respect to (1x,2)⊂Pd_6_L_4_ in the lowest energetic intermediate.

d Both H_2_O molecules are bound to the same carbonyl group.

These results indicate that microsolvation slightly stabilizes the process inside the cavity, as it does in solution. The barrier decrease introduced by microsolvation is, on average, quite similar for both environments (in solution and within the cage); thus, it does not significantly modify the energy barrier upon encapsulation. In addition, calculations with D_2_O as explicit solvent show that the activation barrier remains essentially unchanged, further indicating that hydrogen-bonding effects are present but do not play a dominant role. A global electron density transfer (GEDT) analysis indicates that the reaction has a polar character, with negligible electron density transfer between the water molecules and the reacting species (Table S5).^83^

The stereo- and regioselectivity of the reaction is defined by the preferred pathway. The presence of the metallocage forces an *endo* orientation for all of the reactants studied, thereby defining the stereoselectivity. Regarding regioselectivity, despite the addition over the less electron-rich ring of the naphthalene being favored (in agreement with experiment), the values of the Gibbs energy barriers do not introduce a clear-cut differentiation. For **1b**, the energy barriers are 29.1 vs 27.9 kcal·mol^–1^, whereas for **1c**, these barriers are 31.1 vs 30.2 kcal·mol^–1^, for paths **i** and **iii**, respectively; therefore, all of them are accessible. Note, however, that MD simulations of **1b** and **1c** with **2** inside the cavity show that the pro-reactive molecular disposition, corresponding to that of path **iii**, is the only sampled configuration; none of the other prereactive configurations giving rise to pathways **i**, **ii**, and **iv** are observed.

Comparison with the process in solution shows that both stereo- and regioselectivity changes upon encapsulation, in agreement with experimental results. Moreover, the results obtained in the present work are consistent with the previous computational studies on the DA reaction of 9-hydroxymethylanthracene and *N*-cyclohexylphthalimide inside the same metallocage. In those studies, the origin of the regioselectivity was mainly attributed to confinement effects and noncovalent interaction (i.e., for the DA reaction of anthracene, the transition state of the 9,10-addition is more outstretched compared to the 1,4-addition upon encapsulation by the metallocage).^56^ Interestingly, the relative energies of the encapsulated reactants leading to path iii are always the most favorable ones (Δ*G*_r_ in Table S1), which is consistent with the MD simulations where the pro-reactive conformations corresponding to path iii are the only ones observed (*vide supra*).

The Gibbs energy barriers obtained for all dienes analyzed present similar values (27.0–30.2 kcal·mol^–1^). Therefore, the difference observed in their reactivity (**1a** and **1d** are unreactive) cannot be attributed to the intrinsic energy barriers. The lack of reactivity is likely related to the formation of a pro-reactive complex within the metallocage environment. Indeed, the observed reactivity appears to correlate with the population of the prereactive conformations (Table 4), as originally suggested by Fujita.

### Comparing Reactions in the Solvent and Inside the Metallocage

For all of the computed Diels–Alder reactions, the energy barriers in solution and inside the metallocage differ substantially. For the reaction between **2** and **1b** (including microsolvation), the energy barriers are 36.4 and 27.9 kcal·mol^–1^, respectively. Thus, encapsulation reduces the barrier by 8.6 kcal·mol^–1^. For the reaction between **2** and **1c**, the Gibbs energy barriers (also including microsolvation) are 36.2 kcal·mol^–1^ in solution and 30.2 kcal·mol^–1^ inside the cage, corresponding to a decrease of 6.0 kcal/mol. For the nonreactive dienes, the computed barriers follow a similar trend. For the reaction between **2** and the unsubstituted naphthalene **1a**, the energy barriers, including microsolvation, are 37.4 kcal·mol^–1^ in solution and 27.0 kcal·mol^–1^ inside the cage, corresponding to a decrease of 10.4 kcal·mol^–1^. For the reaction between substrates **2** and **1d**, the barriers are 36.7 and 29.3 kcal·mol^–1^, respectively, representing a reduction of 7.4 kcal·mol^–1^.

For anthracene, **1e**, however, the barrier decreases by only 0.6 kcal·mol-1 (29.0 kcal·mol^–1^ in solution and 28.4 kcal·mol^–1^ inside the cage). These results are consistent with the previous computational study by Xu and co-workers on the reaction between 9-hydroxymethylanthracene and N-cyclohexylphthalimide inside the same metallocage (25.5 kcal·mol^–1^ in solution and 24.8 kcal·mol^–1^ inside the cage).^56^

Reactions in the confined space become feasible because their barriers decrease by 6.0–10.4 kcal·mol^–1^, except for anthracene (1.2 kcal·mol^–1^). One of the main reasons for such a decrease is that encapsulation of the reactants is thermodynamically favored (see Δ*G*_bind,TOT_ in Table 3). Once encapsulated, the reactants spontaneously adopt a proper relative orientation for the reaction to take place, as confirmed by the MD analysis showing that pro-reactive configurations are accessible. In solution, bringing the reactants together has a Gibbs energy cost of around 6 kcal·mol^–1^ (Table 2). This energetic penalty essentially disappears once the reactants are confined inside the metallocage, in agreement with other computational studies.^56,39^

To better understand the origin of the rate acceleration, we performed a decomposition analysis of the Gibbs energy barrier for the reaction between maleimide **2** and naphthalene **1c** in bulk solution and inside the metallocage.^35^ The decomposition separates the Gibbs energy into a potential energy contribution (Δ*E*_aq_) and a thermal correction (ΔTh_aq_). Although the Δ*E*_aq_/ΔTh_aq_ decomposition does not correspond to a strict enthalpy/entropy separation, it indicates that the barrier reduction under confinement arises mainly from the thermal contribution, consistent with confinement-induced preorganization of the reactants within the metallocage. For the reaction in solution, the Gibbs energy barrier is 36.2 kcal·mol^–1^ with a potential energy barrier, Δ*E*_aq_, of 28.3 kcal·mol^–1^, and a thermal contribution, ΔTh_aq_, of 7.9 kcal·mol^–1^. For the reaction inside the metallocage, the potential energy barrier is 27.3 kcal·mol^–1^, whereas the thermal contribution is 2.9 kcal·mol^–1^, giving an overall Gibbs energy barrier of 30.2 kcal·mol^–1^.

Thus, the encapsulation decreases the potential energy barrier by 1.0 kcal·mol^–1^ and the thermal effects contribution by 5.0 kcal·mol^–1^, resulting in an overall reduction of 6.0 kcal·mol^–1^ (Scheme 4).

**Scheme 4:**
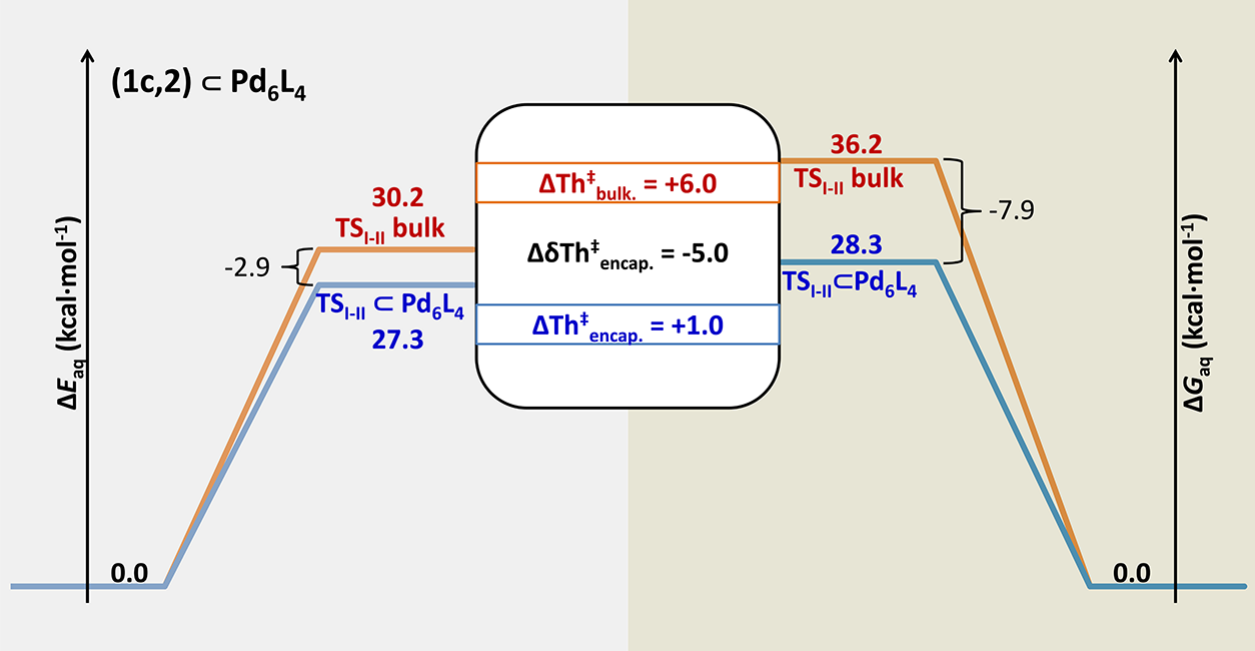
Representation of Gibbs Energy Barrier Decomposition in Potential Energy (Δ*E*_aq_) and Thermal Correction (ΔTh_aq_) for the Most Favored TS (Including 1 H_2_O Explicit Molecule) of the DA Reaction between **1c** and **2** in Bulk (Red Profile) and Inside the Metallocage (Blue Profile)

These results indicate that the environment created by the metallocage contributes to the acceleration of the reaction through both potential energy stabilization (Δδ*E*_aq_) and entropic effects (ΔδTh_aq_). The thermal contribution is particularly significant: although positive in both environments, it is much larger in solution (7.9 vs 2.9 kcal·mol^–1^). This term is comparable to the energy required to form the van der Waals complex in solution, suggesting that the main role of the Pd_6_L_4_ metallocage is to preorganize the reactants and facilitate their approach.

Regarding selectivity, encapsulation also modifies the preferred pathways. In solution, the favored one is the pro-*exo* (path (ii)), whereas inside the cavity, the reaction proceeds through the pro-*endo* pathway (path (iii)).

## Conclusions

The Diels–Alder reaction between maleimide and naphthalene-based dienes is analyzed by means of multiscale methods. The results provide a unified mechanistic interpretation of the substrate-dependent reactivity observed experimentally. The intrinsic reaction barriers for the different dienes are similar once encapsulated, indicating that the lack of reactivity for some substrates cannot be attributed to unfavorable transition-state energetics. Instead, reactivity is governed by the accessibility and population of pro-reactive conformations within the cavity, which depends on how efficiently each diene can be preorganized inside the confined environment. Overall, the analysis shows that microsolvation effects are relatively minor, whereas the hydrophobic environment and, most importantly, confinement effects, by bringing the reactants together and promoting preorganization, play the dominant role in accelerating the Diels–Alder reaction. These findings go beyond previous computational studies and provide a comprehensive mechanistic framework that rationalizes both reactive and nonreactive substrates, offering valuable insights into the factors controlling chemical reactivity in confined spaces.

## Supplementary Material



## Data Availability

All computational results are available at the CORA repository and can be accessed via: https://doi.org/10.34810/DATA3409.

## Notes

Supporting Information tables, supporting Information figures, and Cartesian coordinates of the optimized structures, references (PDF)

## Abbreviations

DFT: density functional theory
B3LYP-D3: dispersion-corrected Becke three-parameter Lee–Yang–Parr exchange–correlation functional
quasi-RRHO: quasi-rigid-rotor-harmonic-oscillator approximation
MD: molecular dynamics
MCPB.py: python-based metal center parameter builder program
RESP: restrained electrostatic potential method
APR: attach-pull-release method.
