# Supplementary material for: Diels–Alder Reactions in [Pd6L4]12+ Metallocages: The Key Roles of Preorganization and Confinement
Source: Inorg Chem. 2026 Jun 30;65(28):16163–72. doi: 10.1021/acs.inorgchem.6c01395 (PMC13390044; doi:10.1021/acs.inorgchem.6c01395)
Supplement: Supplementary file 1 [file ic6c01395_si_001.pdf]

Supporting Information

Diels−Alder Reactions in $[Pd_6L_4]^{12+}$ Metallocages: The Key Roles of Preorganization and Confinement

Giuseppe Sciortino,* Irina Cuesta, Gantulga Norjmaa, Gregori Ujaque*

Departament de Química, Universitat Autònoma de Barcelona, 08193 Cerdanyola del Vallès, Catalonia, Spain E-mail: giuseppe.sciortino@uab.cat; gregori.ujaque@uab.cat

Contents

Behavior of reactants inside the cavity ………………………………..……… S2
Analysis of all reaction pathways for encapsulated reactants. ……..………..……… S4
Analysis of encapsulates solvent molecules ……………………………..…… S6
Analysis of Non-Covalent Interactions ………….…………………….…..… S8
Analysis of Microsolvation …………………………………………..……… S9
Global Electron Density Transfer (GEDT) Analysis ……...……………….……… S9
Analysis of convergence ……………………………………………..………. S10
Cartesian Coordinates ……………………………………………………… S11

Behavior of reactants inside the cavity

In addition to the pro-active conformation, we also analysed the behaviour of reactants inside the cavity along the simulations. In Figure S1 are shown snapshots of the MD simulation for each of the reactants.

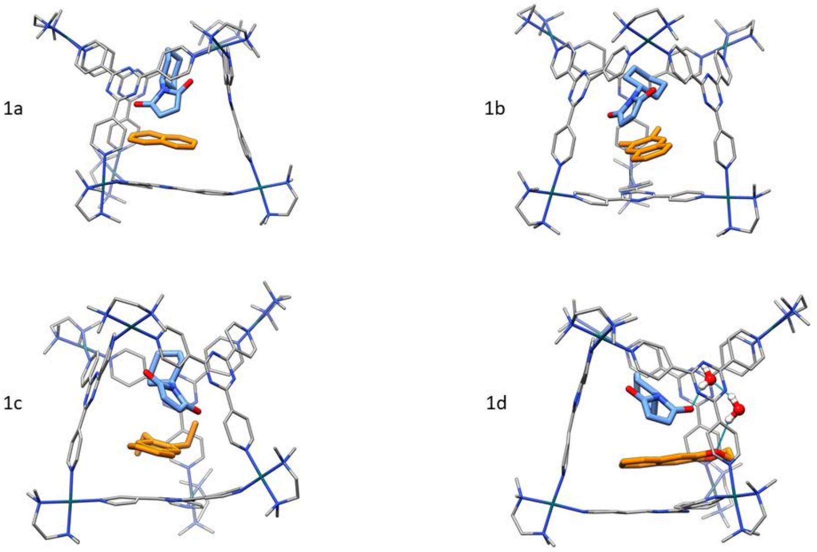

Figure S1. Snapshots of the most representative structures for each of the reactants (**1a**-**1d**) inside the metallocage **3**.

We measured the distance between center of mass (COM) of the guest and the center of mass of each of the four ligands of the $[Pd_6L_4]^{12+}$ metallocage. We observed that for all cases naphthalene moieties mostly remains stacked to one of the sides of the metallocage (aromatic walls, Figures S2-S5). We also observed that the smaller of the substituents of the naphthalene-based dienes, the greater the relative loose to move, but mostly parallel to the aromatic walls.

(a) (b)

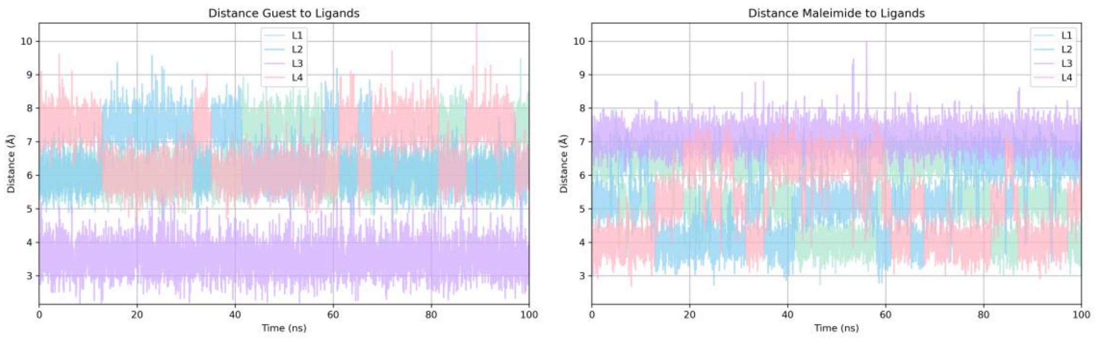

*Figure S2. Distance between the center of mass of each ligand of the metallocage along the MD simulation with the center of mass of: (a) diene **1a**, (b) maleimide **2**.*

(a) (b)

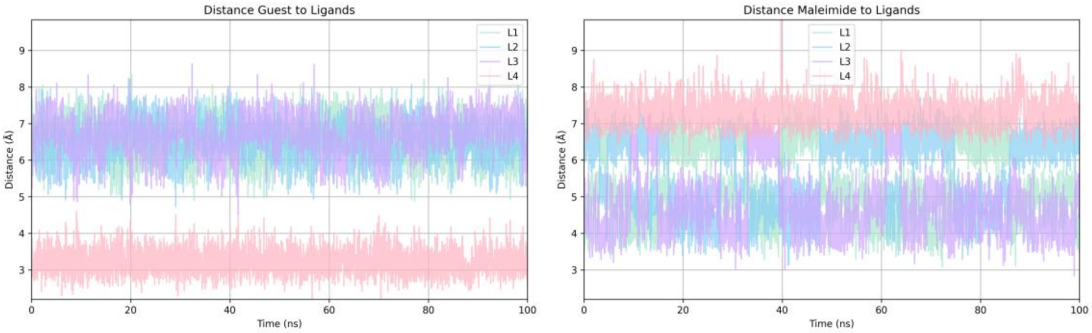

*Figure S3. Distance between the center of mass of each ligand of the metallocage along the MD simulation with the center of mass of: (a) diene **1b**, (b) maleimide **2**.*

(a) (b)

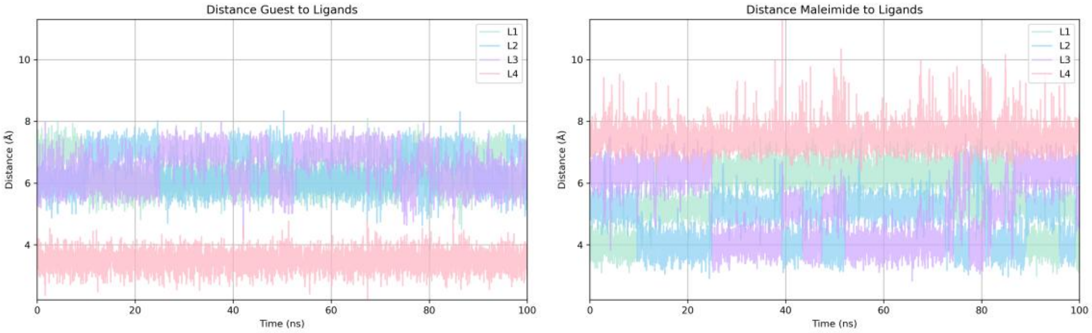

*Figure S4. Distance between the center of mass of each ligand of the metallocage along the MD simulation with the center of mass of: (a) diene **1c**, (b) maleimide **2**.*

(a) (b)

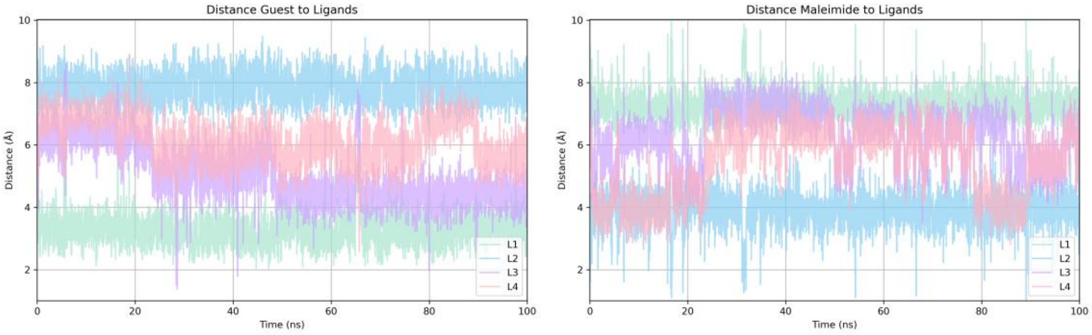

*Figure S5. Distance between the center of mass of each ligand of the metallocage along the MD simulation with the center of mass of: (a) diene **1d**, (b) maleimide **2**.*

For the case of the reaction product **4c** (given by the reaction of 1c + 2) for the encapsulated reaction, the MD simulation was performed. The most common structure along the dynamics for the product of the reaction, **4c**, was then optimized at DFT/MM level. The structure is shown in Figure S6.

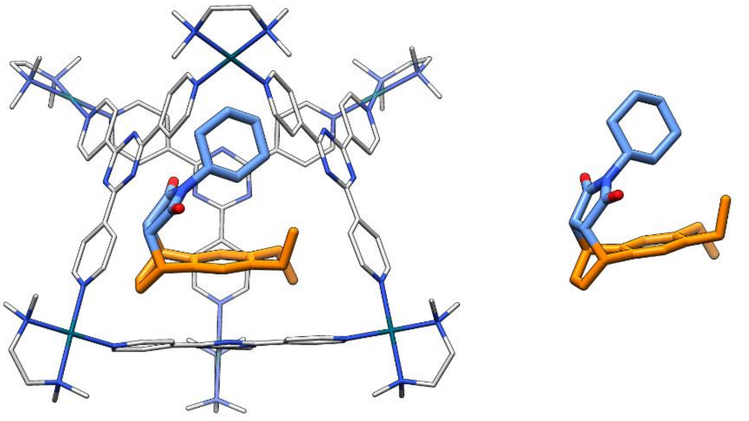

Figure S6. DFT/MM optimized structure of ***4_{iii}c*** *included into the cavity of the metallocage,* ***4_{iii}c⊂Pd_6L_4****.*

Analysis of all reaction pathways for encapsulated reactants

The Gibbs energy barriers observed for reaction between **1a-d** and **2** without solvent molecules inside the cavity, **(1b,2)⊂Pd_6L_4**, reveal that the four possible pathways, **TS_{i-iv}**, give rise to relatively similar energies. These results clearly show that the cage enhances the reaction rate: whereas in solution the corresponding barriers are around 40 kcal·mol^{-1}, inside the cage they are around 30 kcal/mol (Table S1).

Table S1. ONIOM(DFT:MM) ΔG values of transition states and product for the Diels-Alder reaction between substituted naphtalenes ***1a-d*** *with N-cyclohexylmaleimide* ***2*** *in metallocage* ***3****. T= 100 ºC.*[a,b]

**Species**	**$(1x,2)\subset 3$**				**$[1x,2,(H_2O)_n]\subset 3$**	
					(H_2O)	$(H_2O)_2$
**1a**			**Path iii**	**Path iv**	**Path iii**	**Path iii**
**$\Delta\Delta G_{(1a,2)\subset 3}$**	--	--	1.0	0.0	--	--
**$TS_{I\text{-}II}$** [c]	--	--	31.5	32.8	27.5	27.0 [d]
**ΔG_r** [c]	--	--	5.0	7.5	6.0	4.2
**1b**	**Path i**	**Path ii**	**Path iii**	**Path iv**	**Path iii**	**Path iii**
**$\Delta\Delta G_{(1b,2)\subset 3}$**	3.7	2.3	**0.0**	2.6	--	--
**$TS_{I\text{-}II}$** [c]	31.5	31.8	33.4	33.0	28.2	27.8 [d]
**ΔG_r** [c]	8.7	7.8	7.2	11.3	5.9	6.6
**1c**	**Path i**	**Path ii**	**Path iii**	**Path iv**	**Path iii**	**Path iii**
**$\Delta\Delta G_{(1c,2)\subset 3}$**	2.5	2.8	**0.0**	2.2	**--**	--
**$TS_{I\text{-}II}$** [c]	32.3	35.3	**28.5**	34.9	**29.4**	29.7 [d]
**ΔG_r** [c]	6.7	10.5	5.9	11.2	1.6	7.1
**1d**	**Path i**	**Path ii**	**Path iii**	**Path iv**	**Path iii**	**Path iii**
**$\Delta\Delta G_{(1d,2)\subset 3}$**	**0.0**	2.8	7.1	2.9	**--**	--
**$TS_{I\text{-}II}$** [c]	**30.7**	35.9	30.1	32.5	33.0	**29.3** [d]
**ΔG_r** [c]	10.3	9.0	6.7	4.5	5.9	7.6

[a] Only the lowest conformations are reported. [b] Gibbs energy values in kcal·mol^{-1}. [c] ΔG respect to $(1x,2)\subset Pd_6L_4$ in the lowest energetic intermediate. [d] Both H_2O bound to one carbonyl group.

Analysis of encapsulates molecules

The number of water solvent molecules and counterions encapsulated within the metallocage was calculated throughout the molecular dynamics (MD) simulation. The results for water molecules are shown in the following figures for each naphthalene-based diene. No counterion was observed inside the metallocage during any of the simulations, as the cavity remained occupied by the reactants and solvent molecules.

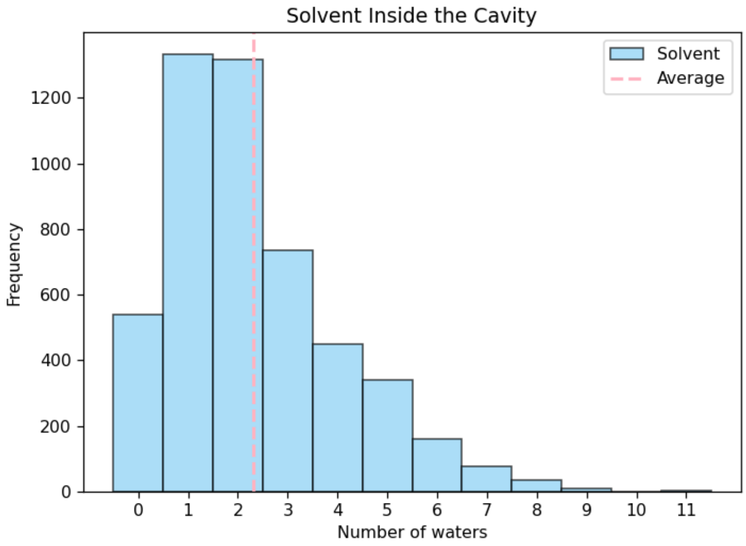

*Figure S7. Number of frames that contain a certain number of water molecules for **1a**.*

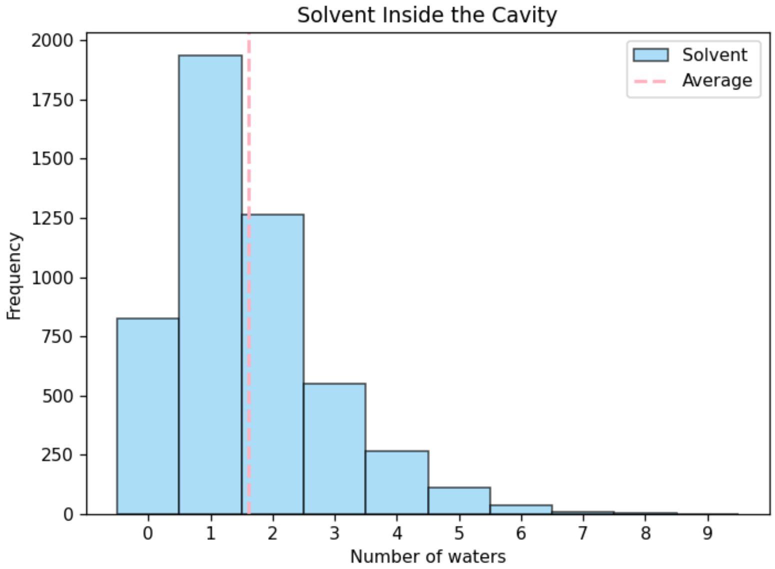

*Figure S8. Number of frames that contain a certain number of water molecules for **1b**.*

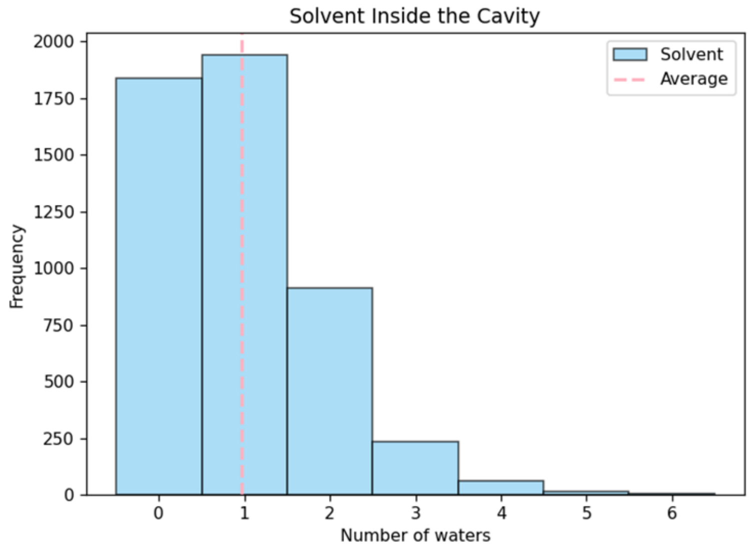

Figure S9. Number of frames that contain a certain number of water molecules for ***1c****.*

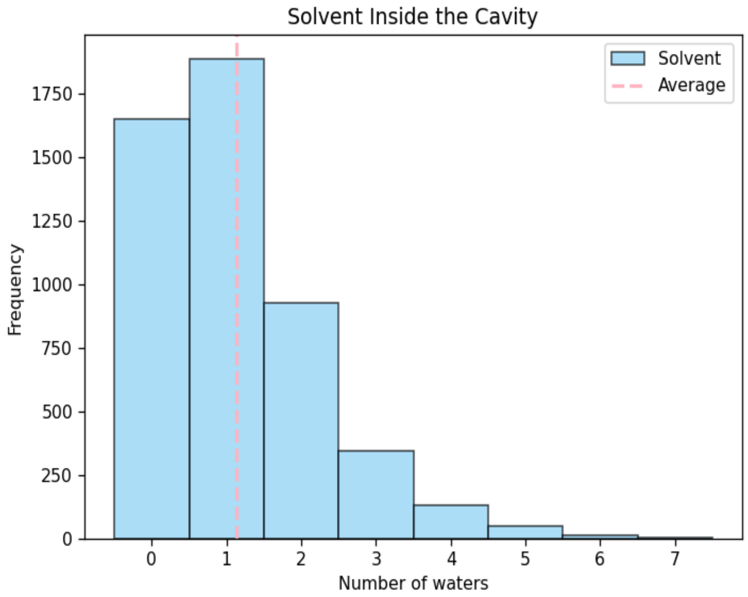

Figure S10. Number of frames that contain a certain number of water molecules for ***1d****.*

Analysis of Non-Covalent Interactions

The Non-Covalent Interactions (NCI) method identifies non-covalent interactions by analyzing the electron density ρ and its derivatives, specifically the reduced density gradient. With the NCIPLOT4 program, a semiquantitative evaluation of NCI strengths is achieved by integrating ρ within the NCI regions. We performed NCI analysis on the optimized geometries and report the integrated ρ value corresponding to van der Waals interactions. The analysis was carried out for the three most populated proreactive and non-proreactive structures obtained from the MD simulations, as well as for the transition states corresponding to the four possible reaction pathways (Tables S2 and S3).

Table S2. Comparative analysis of the average of the most populated strucutre for proreactive and non-proreactive configuration.

	NON PROREACTIVE	PROREACTIVE
Cage - Naphtalene-based	2.23	1.94
Cage - Maleimide	1.73	1.66
Naphtalene-based - Maleimide	0.80	1.01

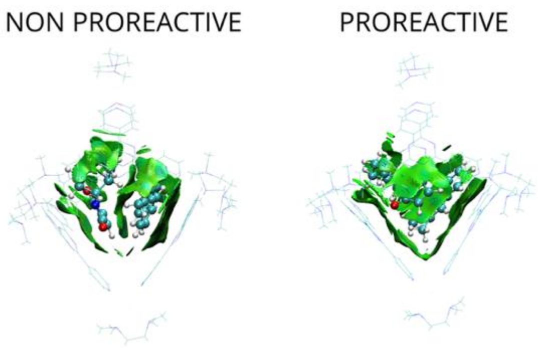

Figure S11. Representation of the Van der Waals interactions for the non-proreactive and proreactive cases.

Table S3. Comparative analysis interactions for the transition states corresponding to the four possible reaction pathways.

	TS1	TS2	TS3	TS4
Cage - Naphtalene-based	2.19	2.02	2.33	2.21
Cage - Maleimide	1.63	1.01	1.62	0.98
Naphtalene-based - Maleimide	1.02	1.17	1.00	0.88

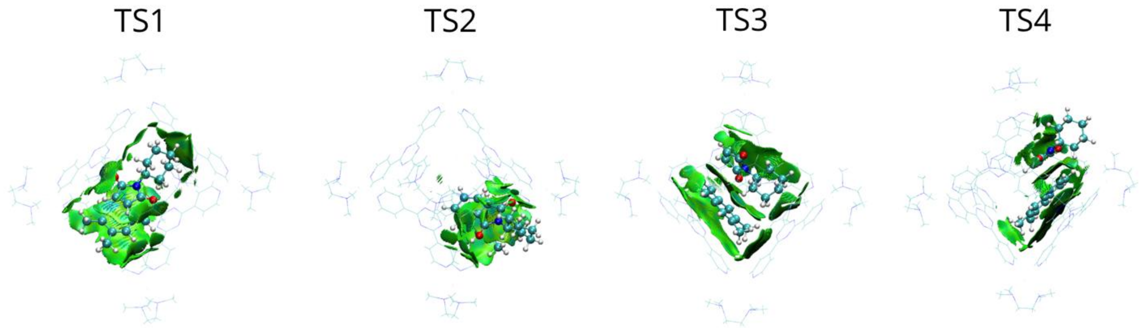

Figure S12. Representation of the Van der Waals interactions for the transition states corresponding to the four possible reaction pathways.

Analysis of Microsolvation

We systematically analyzed the effect of microsolvation on the reaction barriers by including different numbers of explicit water molecules in our computational model (for the methyl case of the TS3 pathway). The results show that the lowest activation barriers are obtained with one or two explicit water molecules. The inclusion of a third water molecules does not modify significantly the barrier, whereas the inclusion of the fourth water molecules leads to much higher barriers.

	**1 H_2O**	**2 H_2O**	**3 H_2O**	**4 H_2O**
Energy (kcal/mol)	28,2	27,8	28.4	35.6

Table S4. Activation barriers (kcal/mol) for the TS3 pathway and methyl case with 1–4 explicit water molecules.

Global Electron Density Transfer (GEDT) Analysis

To gain further insight into the electronic factors governing reactivity, a Global Electron Density Transfer (GEDT) analysis was performed at the transition states for path iii with one and two explicit water molecules. NPA charges were computed and the GEDT was calculated as, GEDT = $-\Sigma$ q(f), where the sum comes from all natural atomic charges of the maleimide fragment at the transition state (Table S5). Positive GEDT value were obtained in both cases (0.16e for 1 H_2O and 0.17e for 2 H_2O, respectively), indicating a electron density transfer from the naphthalene diene to the maleimide dienophile at the transition state consistent with a polar Diels-Alder mechanism.

	**1 H_2O**	**2 H_2O**
Malimide (Dienofile)	-0.16 e	-0.17 e
Naphtalene (Diene)	+0.17 e	+0.19 e
H_2O	-0.01 e	-0.02 e

Table S5. Natural Population Analysis (NPA) charges of the transition state (path iii) with one and two water molecules.

Analysis of convergence

Convergence of the MD trajectories was assessed via principal component analysis (PCA; Figure S13). Projection of the trajectories onto the first principal components reveals recurrent exploration of the dominant conformational basins throughout the simulation, indicating stable sampling of the relevant conformational landscape. These results are consistent with the preferential observation of pro-endo pro-reactive arrangements under confinement. Although exo-like arrangements cannot be completely ruled out and may occur as rare events, no such conformations were observed within the simulated time scale.

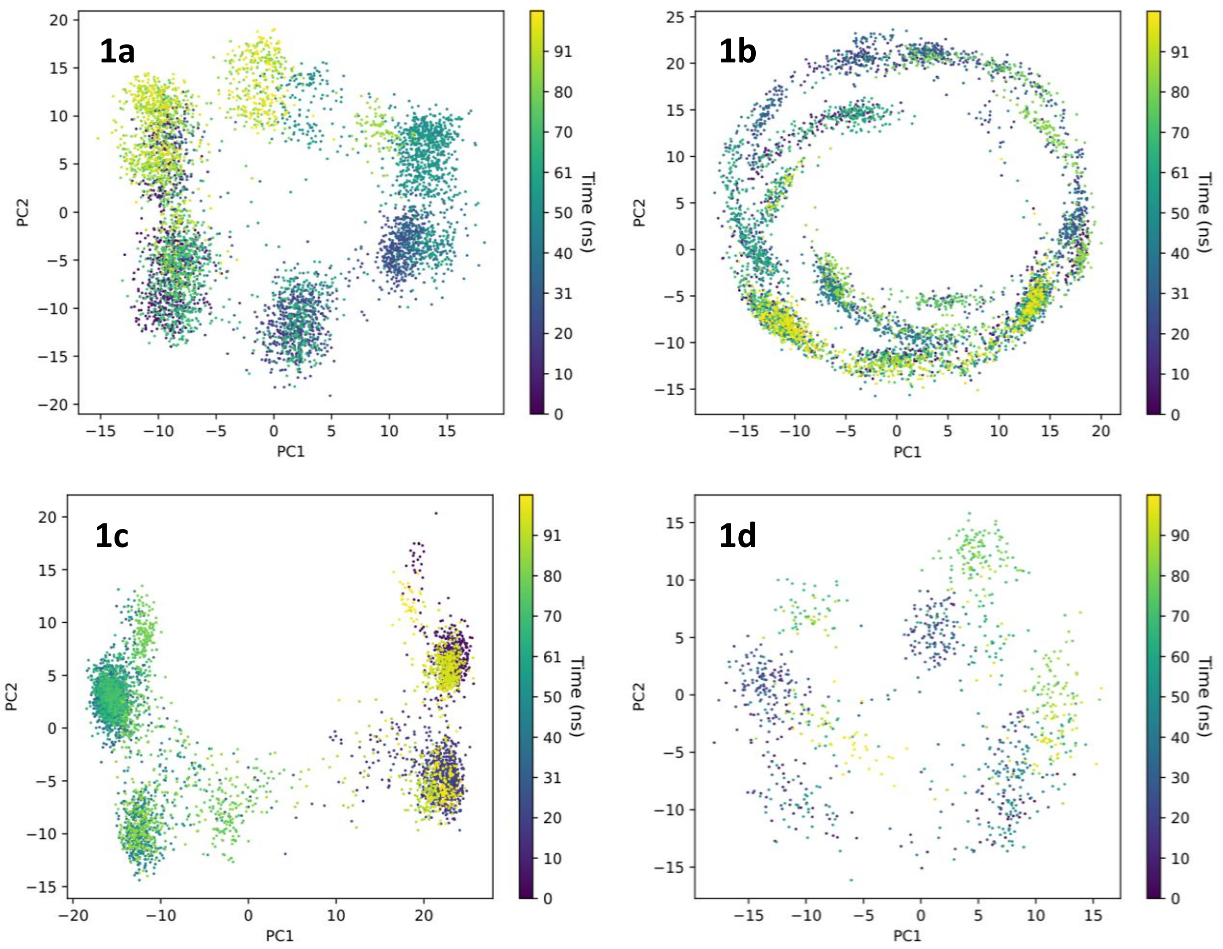

*Figure S13. Principal Component Analysis (PCA) of MD trajectories for **1a–1d**..*

Cartesian Coordinates

Table1_1a_TS_1wat

Property	Value
Charge	0
Electronic Energy, BS1 (a.u.)	-1056.467858
Thermal and entropic correction, BS1 (a.u.)	0.330218
Electronic Energy, BS2 (a.u.)	-1056.854961
Number of Imaginary Frequencies	1
Imaginary frequencies (cm-1)	-501.0658

Molecular Geometry in Cartesian Coordinates

C	-4.099515	0.530499	0.059633
C	-2.798783	0.858039	-0.440694
C	-2.274729	2.185612	-0.149862
C	-3.229656	3.227811	-0.108729
C	-4.609133	2.832190	-0.367946
C	-5.021499	1.542939	0.101835
H	-0.177038	1.683271	-0.056548
H	-4.376244	-0.508145	0.209731
H	-2.084197	0.051248	-0.582558
C	-0.909520	2.484781	-0.010763
C	-2.807161	4.554723	0.070732
H	-5.355749	3.619671	-0.432144
H	-6.068865	1.349484	0.307349
C	-1.456153	4.833797	0.238700
C	-0.506780	3.798205	0.197776
H	-3.542398	5.354842	0.087977
C	-4.051493	0.077856	-2.819989
C	-3.332976	1.329427	-2.465375
C	-4.295667	2.371024	-2.406670
C	-5.621909	1.771812	-2.744981
H	-2.308927	1.458101	-2.787752
H	-4.099624	3.393539	-2.700417
C	-6.458662	-0.614072	-3.095410
C	-7.361878	-0.717511	-1.855617
C	-7.267887	-0.357708	-4.377062
H	-5.915718	-1.557897	-3.213723
C	-8.433603	-1.798927	-2.057392
H	-7.836500	0.256071	-1.688263
H	-6.737005	-0.947802	-0.987660
C	-8.331852	-1.449958	-4.564498
H	-7.745951	0.623712	-4.299458
H	-6.587641	-0.325110	-5.235935
C	-9.248792	-1.554347	-3.336088
H	-9.091000	-1.840496	-1.181673
H	-7.946337	-2.783631	-2.127119
H	-8.920053	-1.244249	-5.465902
H	-7.836428	-2.417932	-4.727985
H	-9.984928	-2.354166	-3.477048
H	-9.813801	-0.618165	-3.227464
N	-5.407843	0.390334	-2.900711
O	-6.701213	2.327841	-2.841608
O	-3.582913	-1.045874	-2.975390
H	0.547039	4.027323	0.324997
H	-1.130959	5.857619	0.397632
H	-5.466801	-3.058773	-1.186824
O	-4.710404	-2.569021	-0.843694
H	-4.259628	-2.227176	-1.636091

Table1_1a_TS_2wat

Property	Value
Charge	0
Electronic Energy, BS1 (a.u.)	-1132.912141
Thermal and entropic correction, BS1 (a.u.)	0.352348

Property	Value
Electronic Energy, BS2 (a.u.)	-1133.335806
Number of Imaginary Frequencies	1
Imaginary frequencies (cm-1)	-503.143

Molecular Geometry in Cartesian Coordinates

C	-4.218252	0.581485	0.111569
C	-2.880509	0.834909	-0.323379
C	-2.331061	2.160795	-0.092717
C	-3.247879	3.235845	-0.144040
C	-4.627789	2.872409	-0.458290
C	-5.106904	1.622091	0.063385
H	-0.260024	1.591089	0.114927
H	-4.530629	-0.438438	0.309937
H	-2.198705	-0.009369	-0.405442
C	-0.961978	2.420102	0.090600
C	-2.787031	4.554564	-0.011531
H	-5.344140	3.682276	-0.573387
H	-6.168412	1.475778	0.231910
C	-1.434563	4.795455	0.201916
C	-0.521671	3.727822	0.252158
H	-3.492560	5.379421	-0.066128
C	-4.080719	0.047826	-2.800808
C	-3.344354	1.274254	-2.442594
C	-4.282349	2.342724	-2.411634
C	-5.617229	1.766073	-2.774643
H	-2.302065	1.369468	-2.711024
H	-4.053107	3.344604	-2.751389
C	-6.491787	-0.612247	-3.122810
C	-7.367392	-0.747546	-1.866158
C	-7.324258	-0.312152	-4.380036
H	-5.962299	-1.557583	-3.283575
C	-8.457946	-1.808151	-2.077987
H	-7.823664	0.226856	-1.654638
H	-6.729027	-1.018613	-1.019464
C	-8.399801	-1.391548	-4.575651
H	-7.794529	0.668990	-4.266844
H	-6.660828	-0.259812	-5.251018
C	-9.294435	-1.518511	-3.333068
H	-9.098940	-1.863411	-1.190894
H	-7.982298	-2.794340	-2.180995
H	-9.002186	-1.157001	-5.460455
H	-7.916296	-2.359147	-4.773060
H	-10.042352	-2.305983	-3.481330
H	-9.846596	-0.579299	-3.189529
N	-5.426759	0.378604	-2.917158
O	-6.681834	2.342979	-2.890523
O	-3.641136	-1.097775	-2.949155
H	0.533447	3.928114	0.413097
H	-1.079936	5.814431	0.325403
H	-2.538355	-2.418898	-0.624913
O	-1.709291	-2.095697	-1.015769
H	-2.020461	-1.762956	-1.872581
H	-4.914152	-3.466648	-0.894079
O	-4.581264	-2.569638	-0.769532
H	-4.406143	-2.226765	-1.665979

Table1_1a_TSiii_nowat

Property	Value
Charge	0
Electronic Energy, BS1 (a.u.)	-980.028670
Thermal and entropic correction, BS1 (a.u.)	0.309915
Electronic Energy, BS2 (a.u.)	-980.377074
Number of Imaginary Frequencies	1
Imaginary frequencies (cm-1)	-499.7468

Molecular Geometry in Cartesian Coordinates

C	-4.141179	0.833260	-0.435138
C	-2.812120	0.910181	0.089785

C	-2.050050	2.110929	-0.200512
C	-2.791501	3.314605	-0.270008
C	-4.221871	3.194923	-0.051272
C	-4.858682	1.997743	-0.510258
H	-0.085173	1.218890	-0.276609
H	-4.601383	-0.133584	-0.612765
H	-2.252493	-0.008004	0.247535
C	-0.652037	2.142177	-0.351410
C	-2.123200	4.535261	-0.474682
H	-4.796465	4.116516	-0.010761
H	-5.917121	2.001995	-0.750677
C	-0.749921	4.544349	-0.672291
C	-0.014463	3.345404	-0.616026
H	-2.693135	5.459427	-0.496342
C	-3.328933	3.711072	2.617753
C	-4.162328	2.621518	2.022848
C	-3.415221	1.423948	2.107789
C	-2.111114	1.753256	2.764323
H	-5.235465	2.683241	2.140290
H	-3.841256	0.447988	2.294866
C	-1.037658	3.892429	3.647983
C	-0.948942	3.486175	5.130340
C	0.320161	3.758029	2.944251
H	-1.366810	4.936003	3.588634
C	0.131302	4.300683	5.857302
H	-0.710468	2.417654	5.181571
H	-1.927220	3.628465	5.604263
C	1.392712	4.574441	3.680874
H	0.601414	2.700018	2.922386
H	0.222999	4.089554	1.908035
C	1.493295	4.170010	5.159194
H	0.201323	3.977884	6.902551
H	-0.163931	5.359653	5.875133
H	2.360695	4.449508	3.181705
H	1.143688	5.643694	3.616964
H	2.244757	4.781242	5.672882
H	1.833281	3.126948	5.224923
N	-2.097192	3.138632	2.967215
O	-1.216778	0.984127	3.073543
O	-3.624118	4.882069	2.776139
H	1.061389	3.366605	-0.761790
H	-0.236792	5.482277	-0.862644

Table1_1a_TSiv_nowat

Property	Value
Charge	0
Electronic Energy, BS1 (a.u.)	-980.027961
Thermal and entropic correction, BS1 (a.u.)	0.309513
Electronic Energy, BS2 (a.u.)	-980.376952
Number of Imaginary Frequencies	1
Imaginary frequencies (cm-1)	-505.0725

Molecular Geometry in Cartesian Coordinates

C	-4.072002	0.565816	0.116086
C	-2.780116	0.889786	-0.406317
C	-2.261683	2.223696	-0.133764
C	-3.222509	3.260602	-0.089751
C	-4.602684	2.856477	-0.322967
C	-5.002778	1.570258	0.159077
H	-0.159810	1.734848	-0.065220
H	-4.338351	-0.468969	0.303888
H	-2.061642	0.085416	-0.540549
C	-0.896399	2.532469	-0.017410
C	-2.804803	4.592109	0.069965
H	-5.354564	3.638436	-0.389780
H	-6.045801	1.372990	0.381655
C	-1.453549	4.880807	0.215014
C	-0.498583	3.850184	0.171272
H	-3.544597	5.387945	0.089792
C	-4.050390	0.062432	-2.773422
C	-3.334876	1.324874	-2.422739
C	-4.299893	2.362251	-2.376682
C	-5.628000	1.757462	-2.698864
H	-2.314558	1.457345	-2.756532

H	-4.107961	3.384560	-2.673563
C	-6.464233	-0.627535	-3.019754
C	-7.439944	-0.653392	-1.830833
C	-7.203782	-0.425463	-4.353370
H	-5.923903	-1.580229	-3.058046
C	-8.516773	-1.729631	-2.033754
H	-7.907915	0.333629	-1.743788
H	-6.879047	-0.833576	-0.906427
C	-8.279310	-1.505121	-4.545455
H	-7.664697	0.567966	-4.347861
H	-6.478897	-0.447205	-5.175257
C	-9.260506	-1.532747	-3.363542
H	-9.220506	-1.714964	-1.193617
H	-8.043223	-2.721784	-2.029209
H	-8.815530	-1.332884	-5.485698
H	-7.795992	-2.488557	-4.635498
H	-10.004040	-2.325946	-3.504005
H	-9.812028	-0.582770	-3.330695
N	-5.417063	0.378992	-2.825952
O	-6.705252	2.319692	-2.806717
O	-3.586147	-1.048905	-2.955663
H	0.555581	4.086695	0.280931
H	-1.132135	5.908139	0.358221

Table1_1b_TS_1wat

Property	Value
Charge	0
Electronic Energy, BS1 (a.u.)	-1135.126382
Thermal and entropic correction, BS1 (a.u.)	0.380055
Electronic Energy, BS2 (a.u.)	-1135.145930
Number of Imaginary Frequencies	1
Imaginary frequencies (cm-1)	-496.9386

Molecular Geometry in Cartesian Coordinates

C	1.247333	-0.197905	-0.747282
C	0.054945	0.271920	-1.390554
C	-1.207181	0.019427	-0.719384
C	-1.178745	0.061732	0.691610
C	0.114394	0.331215	1.295020
C	1.275718	-0.173984	0.620558
H	-2.451383	-0.199006	-2.465649
H	2.134173	-0.410658	-1.335399
H	0.039173	0.346088	-2.474624
C	-2.432883	-0.175644	-1.378995
C	-2.382363	-0.051445	1.409227
H	0.145533	0.450984	2.374594
H	2.186336	-0.366722	1.178451
C	-3.590397	-0.270690	0.756740
C	-3.612963	-0.350712	-0.665476
H	-2.365080	0.032456	2.492381
C	-4.871119	-0.388509	1.544825
H	-5.334414	-1.374060	1.415322
H	-5.606221	0.353730	1.212780
C	-4.916631	-0.571963	-1.392011
H	-5.619412	0.248956	-1.202416
H	-5.411854	-1.492112	-1.061345
C	-0.929463	2.944903	1.097556
C	0.347852	2.346624	0.624748
C	0.306854	2.336444	-0.792740
C	-0.984214	2.963848	-1.199737
H	1.240150	2.520486	1.209989
H	1.167183	2.492638	-1.428543
C	-3.015191	3.895099	0.049008
C	-2.914764	5.355136	-0.423938
C	-4.109241	3.127285	-0.703922
H	-3.275765	3.884373	1.110282
C	-4.271524	6.061575	-0.279585
H	-2.596698	5.362865	-1.472699
H	-2.143609	5.874116	0.157047
C	-5.459860	3.841215	-0.547869
H	-3.837802	3.056205	-1.762211
H	-4.162414	2.110882	-0.311579
C	-5.382192	5.300791	-1.020025
H	-4.196268	7.090242	-0.650312

H	-4.530627	6.128555	0.786593
H	-6.231985	3.297160	-1.104197
H	-5.756456	3.816174	0.510311
H	-6.346624	5.801483	-0.874732
H	-5.173880	5.319045	-2.099112
N	-1.705797	3.228032	-0.022367
O	-1.376432	3.217509	-2.327794
O	-1.274941	3.137366	2.261094
O	-3.952869	2.518840	3.063974
H	-3.018987	2.660751	2.825818
H	-4.182788	3.319874	3.552474
H	-4.693629	-0.221380	2.608533
H	-4.762537	-0.638035	-2.471868

Table1_1b_TS_2wat

Property	Value
Charge	0
Electronic Energy, BS1 (a.u.)	-1211.561299
Thermal and entropic correction, BS1 (a.u.)	0.403413
Electronic Energy, BS2 (a.u.)	-1211.603763
Number of Imaginary Frequencies	1
Imaginary frequencies (cm-1)	-486.5222

Molecular Geometry in Cartesian Coordinates

C	-4.049595	0.476169	0.100082
C	-2.773513	0.791631	-0.464811
C	-2.217872	2.115580	-0.270805
C	-3.151771	3.171722	-0.189446
C	-4.546541	2.780042	-0.351017
C	-4.961339	1.510264	0.195669
H	-0.125507	1.582365	-0.319707
H	-2.088279	-0.033720	-0.643533
C	-0.840627	2.396904	-0.243217
C	-2.703138	4.496974	-0.081923
H	-5.285525	3.579318	-0.340416
C	-1.339486	4.759806	-0.022088
C	-0.408170	3.709345	-0.103729
H	-3.424696	5.308261	-0.034824
C	-4.173450	0.093263	-2.906573
C	-3.468311	1.329964	-2.553007
C	-4.443068	2.341277	-2.345027
C	-5.780625	1.709135	-2.597157
H	-2.460082	1.492041	-2.904357
H	-4.308716	3.367855	-2.661813
C	-6.573875	-0.662510	-3.118732
C	-7.654544	-0.752020	-2.030805
C	-7.190004	-0.400565	-4.504142
H	-6.041729	-1.618394	-3.152579
C	-8.676817	-1.842860	-2.384552
H	-8.153667	0.217896	-1.945168
H	-7.187252	-0.961486	-1.062898
C	-8.225290	-1.481399	-4.850235
H	-7.664774	0.587087	-4.490110
H	-6.391411	-0.372962	-5.254119
C	-9.305236	-1.591394	-3.763537
H	-9.451535	-1.887476	-1.611036
H	-8.177437	-2.822422	-2.387062
H	-8.679022	-1.261869	-5.823151
H	-7.715925	-2.450053	-4.952504
H	-10.012547	-2.391362	-4.010212
H	-9.881806	-0.656547	-3.730917
N	-5.544337	0.338614	-2.802934
O	-6.876894	2.239217	-2.602511
O	-3.691575	-0.995423	-3.234061
H	0.654738	3.927573	-0.061681
H	-0.990175	5.782547	0.083557
H	-4.252927	-2.655078	-2.639351
O	-4.376358	-3.457708	-2.098177
H	-4.862794	-3.143741	-1.326160
H	-2.075682	-1.708072	-2.259869
O	-1.764926	-2.261777	-1.525797
H	-2.432263	-2.968840	-1.531308
C	-4.355422	-0.964520	0.437361
H	-4.304361	-1.136264	1.518940

H	-5.359645	-1.257929	0.114456
C	-6.379137	1.374046	0.683205
H	-6.631958	0.349844	0.960997
H	-6.540541	2.006481	1.564193
H	-7.084051	1.708904	-0.086010
H	-3.622510	-1.620372	-0.037844

Table1_1b_TSiii_nowat

Property	Value
Charge	0
Electronic Energy, BS1 (a.u.)	-1058.676672
Thermal and entropic correction, BS1 (a.u.)	0.360980
Electronic Energy, BS2 (a.u.)	-1058.686881
Number of Imaginary Frequencies	1
Imaginary frequencies (cm-1)	-502.4355

Molecular Geometry in Cartesian Coordinates

C	-4.151822	0.836852	-0.439972
C	-2.824412	0.902418	0.095304
C	-2.050067	2.095214	-0.192270
C	-2.776277	3.303104	-0.266621
C	-4.208814	3.199693	-0.057920
C	-4.856209	2.007847	-0.521306
H	-0.094438	1.192896	-0.227448
H	-4.620194	-0.125758	-0.619566
H	-2.274514	-0.021133	0.256579
C	-0.650438	2.123053	-0.310292
C	-2.083413	4.513078	-0.442254
H	-4.774506	4.127045	-0.021229
H	-5.912987	2.023391	-0.768803
C	-0.702815	4.534560	-0.599081
C	0.027794	3.313706	-0.537955
H	-2.643531	5.444255	-0.463836
C	1.530620	3.321360	-0.669210
H	1.853313	3.762833	-1.619324
H	1.995141	3.914618	0.128373
H	1.938988	2.309362	-0.611884
C	0.022891	5.842843	-0.796527
H	0.743203	6.023721	0.011034
H	0.592306	5.853512	-1.733429
H	-0.673986	6.684395	-0.817028
C	-3.348914	3.711073	2.615006
C	-4.175542	2.622562	2.011200
C	-3.427783	1.423417	2.100394
C	-2.125543	1.755523	2.760145
H	-5.249355	2.682335	2.124002
H	-3.856339	0.450134	2.296395
C	-1.059390	3.896474	3.649348
C	-0.931976	3.461538	5.120546
C	0.285418	3.802169	2.914658
H	-1.407338	4.935215	3.618624
C	0.150838	4.278970	5.840517
H	-0.675693	2.396454	5.145252
H	-1.901857	3.578416	5.618063
C	1.363192	4.616126	3.645738
H	0.581071	2.749452	2.855071
H	0.156276	4.160565	1.891717
C	1.500041	4.182074	5.112581
H	0.247685	3.937513	6.877644
H	-0.159284	5.332904	5.884931
H	2.322155	4.514057	3.123852
H	1.099483	5.683185	3.609651
H	2.253691	4.792816	5.623535
H	1.855287	3.142707	5.148838
N	-2.118320	3.139821	2.971736
O	-1.225240	0.990839	3.063898
O	-3.645692	4.881749	2.774642

Table1_1b_TSii_nowat

Property	Value
Charge	0
Electronic Energy, BS1 (a.u.)	-1058.679032
Thermal and entropic correction, BS1 (a.u.)	0.360872
Electronic Energy, BS2 (a.u.)	-1058.688958
Number of Imaginary Frequencies	1
Imaginary frequencies (cm-1)	-477.863

Molecular Geometry in Cartesian Coordinates

```
C          -4.075656        0.572148        0.200085
C          -2.802058        0.899973       -0.379070
C          -2.246690        2.221277       -0.156219
C          -3.189979        3.270979       -0.088680
C          -4.575730        2.872261       -0.243726
C          -4.995629        1.600923        0.279829
H          -0.149232        1.704339       -0.182026
H          -2.101003        0.082632       -0.535226
C          -0.872291        2.512964       -0.115023
C          -2.749068        4.601342        0.018717
H          -5.321179        3.664070       -0.280697
C          -1.389379        4.873889        0.091398
C          -0.450046        3.828487        0.024624
H          -3.477264        5.407287        0.056069
C          -4.116780        0.060805       -2.727961
C          -3.412520        1.332268       -2.407874
C          -4.387582        2.353978       -2.326616
C          -5.718060        1.725558       -2.585240
H          -2.400088        1.473012       -2.760191
H          -4.229454        3.378950       -2.633094
C          -6.513978       -0.662087       -3.010072
C          -7.548567       -0.763145       -1.878851
C          -7.189464       -0.410228       -4.370110
H          -5.955362       -1.603274       -3.064724
C          -8.588737       -1.849960       -2.189058
H          -8.043229        0.207533       -1.769369
H          -7.034859       -0.978423       -0.936609
C          -8.232085       -1.497565       -4.670197
H          -7.670250        0.573697       -4.341556
H          -6.423293       -0.379480       -5.153389
C          -9.270358       -1.602200       -3.543074
H          -9.333008       -1.891140       -1.385520
H          -8.093739       -2.831480       -2.208860
H          -8.723267       -1.287718       -5.627291
H          -7.724607       -2.466249       -4.783639
H          -9.988818       -2.401362       -3.759617
H          -9.842894       -0.665534       -3.492105
N          -5.490978        0.351000       -2.731491
O          -6.812271        2.265894       -2.637996
O          -3.642939       -1.046838       -2.921555
C          -4.346415       -0.879485        0.509800
H          -4.186434       -1.490484       -0.386407
H          -3.657588       -1.248373        1.278343
H          -5.362531       -1.056132        0.864227
C          -6.432170        1.477329        0.721817
H          -7.100466        1.810084       -0.079895
H          -6.710911        0.459074        0.993409
H          -6.621919        2.118421        1.590553
H           0.611007        4.053627        0.078081
H          -1.047559        5.899347        0.195999
```

Table1_1b_TSi_nowat

Property	Value
Charge	0
Electronic Energy, BS1 (a.u.)	-1058.676534
Thermal and entropic correction, BS1 (a.u.)	0.359442
Electronic Energy, BS2 (a.u.)	-1058.688612

Property	Value
Number of Imaginary Frequencies	1
Imaginary frequencies (cm-1)	-492.2427

Molecular Geometry in Cartesian Coordinates

```
C          -4.163694         0.820098        -0.425794
C          -2.830611         0.915857         0.095222
C          -2.056194         2.104300        -0.207728
C          -2.791088         3.308558        -0.283126
C          -4.218952         3.186820        -0.055509
C          -4.882032         1.996976        -0.507646
H          -0.096471         1.200538        -0.283600
H          -2.274100        -0.005006         0.254565
C          -0.659192         2.126006        -0.363379
C          -2.120339         4.525066        -0.499256
H          -4.790103         4.111952        -0.023514
C          -0.746846         4.526716        -0.700203
C          -0.016645         3.325206        -0.637424
H          -2.686538         5.451466        -0.525592
C          -3.327283         3.724451         2.607025
C          -4.162018         2.638031         2.010740
C          -3.418982         1.435646         2.102716
C          -2.117816         1.761639         2.763781
H          -5.235468         2.703339         2.131410
H          -3.851232         0.463072         2.297657
C          -1.034834         3.900467         3.636275
C          -0.937545         3.497554         5.119016
C           0.318454         3.761477         2.924464
H          -1.361918         4.944744         3.576698
C           0.149269         4.310998         5.837292
H          -0.701537         2.428569         5.171074
H          -1.912491         3.643304         5.598775
C           1.397920         4.576437         3.652431
H           0.596555         2.702589         2.903064
H           0.215411         4.090762         1.888013
C           1.506574         4.175087         5.131056
H           0.225156         3.990747         6.882968
H          -0.142956         5.370854         5.854293
H           2.362564         4.447857         3.147649
H           1.151459         5.646275         3.587818
H           2.262973         4.785299         5.638776
H           1.843978         3.131183         5.196974
N          -2.098922         3.147961         2.962803
O          -1.226104         0.990814         3.078291
O          -3.617137         4.897825         2.761072
C          -6.359779         2.013362        -0.802848
H          -6.744074         3.033170        -0.883736
H          -6.582576         1.496461        -1.742631
H          -6.932324         1.498602        -0.018962
C          -4.809601        -0.526879        -0.626221
H          -4.078987        -1.337448        -0.567932
H          -5.585656        -0.717384         0.127702
H          -5.301110        -0.589758        -1.603285
H           1.058955         3.340679        -0.786136
H          -0.230075         5.461028        -0.898642
```

Table1_1b_TSiv_nowat

Property	Value
Charge	0
Electronic Energy, BS1 (a.u.)	-1058.674169
Thermal and entropic correction, BS1 (a.u.)	0.360280
Electronic Energy, BS2 (a.u.)	-1058.685789
Number of Imaginary Frequencies	1
Imaginary frequencies (cm-1)	-507.4779

Molecular Geometry in Cartesian Coordinates

```
C          -4.073832         0.564459         0.119818
C          -2.780656         0.889919        -0.402390
C          -2.264567         2.223417        -0.127118
C          -3.222515         3.257354        -0.083101
```

C	-4.602298	2.855834	-0.318858
C	-5.004071	1.568435	0.162907
H	-0.165217	1.743567	-0.066992
H	-4.340047	-0.470660	0.306313
H	-2.061873	0.085632	-0.536226
C	-0.902143	2.542082	-0.018656
C	-2.795301	4.585544	0.068381
H	-5.353969	3.638181	-0.385003
H	-6.047465	1.371494	0.384323
C	-1.446489	4.898508	0.207714
C	-0.480369	3.855710	0.163241
H	-3.535851	5.381922	0.087996
C	0.988207	4.172703	0.306918
H	1.328740	4.863688	-0.473526
H	1.203019	4.655265	1.268000
H	1.598484	3.268273	0.243005
C	-1.011224	6.330924	0.398956
H	-0.472853	6.464391	1.345011
H	-0.329949	6.653932	-0.397317
H	-1.867697	7.009862	0.402815
C	-4.051674	0.061119	-2.769677
C	-3.337037	1.321845	-2.413728
C	-4.303034	2.360112	-2.367445
C	-5.629530	1.756047	-2.694745
H	-2.317365	1.455816	-2.749047
H	-4.109970	3.381772	-2.666007
C	-6.465123	-0.628355	-3.020437
C	-7.442024	-0.655952	-1.832492
C	-7.203801	-0.424635	-4.354320
H	-5.924861	-1.581092	-3.059596
C	-8.518653	-1.731928	-2.037683
H	-7.909968	0.331010	-1.744665
H	-6.881837	-0.837030	-0.907850
C	-8.279219	-1.503918	-4.548907
H	-7.664486	0.568897	-4.347659
H	-6.478266	-0.445198	-5.175692
C	-9.261358	-1.533314	-3.367805
H	-9.223190	-1.718576	-1.198150
H	-8.045102	-2.724114	-2.034195
H	-8.814810	-1.330443	-5.489323
H	-7.795872	-2.487277	-4.639928
H	-10.004812	-2.326325	-3.510000
H	-9.812899	-0.583366	-3.334068
N	-5.418479	0.377643	-2.824393
O	-6.707196	2.317769	-2.804773
O	-3.587804	-1.050281	-2.954440

Table1_1c_TS_1wat

Property	Value
Charge	0
Electronic Energy, BS1 (a.u.)	-1213.763004
Thermal and entropic correction, BS1 (a.u.)	0.434323
Electronic Energy, BS2 (a.u.)	-1214.185933
Number of Imaginary Frequencies	1
Imaginary frequencies (cm-1)	-497.3521

Molecular Geometry in Cartesian Coordinates

C	1.250153	-0.190055	-0.762514
C	0.060123	0.302166	-1.395074
C	-1.204180	0.033111	-0.733936
C	-1.178239	0.024625	0.674844
C	0.111143	0.278990	1.290713
C	1.275708	-0.202402	0.605479
H	-2.433476	-0.087430	-2.486193
H	2.138219	-0.387152	-1.354256
H	0.046673	0.405165	-2.476876
C	-2.431359	-0.122301	-1.402383
C	-2.381129	-0.132151	1.387004
H	0.139324	0.369855	2.373237
H	2.185261	-0.409553	1.159999
C	-3.589289	-0.329116	0.728688
C	-3.614363	-0.323436	-0.702074
H	-2.345265	-0.107611	2.470444
C	-4.885752	-0.516825	1.496629

C	-4.779249	-0.509666	3.023763
H	-5.356216	-1.453814	1.168662
H	-5.586287	0.273355	1.191385
H	-5.766051	-0.682556	3.464710
H	-4.112165	-1.299642	3.385501
H	-4.412470	0.455462	3.385847
C	-4.939538	-0.487000	-1.423935
C	-4.899788	-0.380339	-2.949937
H	-5.638393	0.268304	-1.039209
H	-5.382362	-1.451991	-1.141476
H	-5.906873	-0.499267	-3.360166
H	-4.520765	0.595262	-3.272499
H	-4.265048	-1.153358	-3.395405
C	-0.936366	2.924382	1.131157
C	0.342088	2.324531	0.663773
C	0.317211	2.336703	-0.753811
C	-0.968916	2.972085	-1.166959
H	1.228034	2.481845	1.262984
H	1.184999	2.504177	-1.376654
C	-2.997690	3.921297	0.065273
C	-2.917087	5.344196	-0.513236
C	-4.109215	3.097100	-0.596645
H	-3.229126	3.991777	1.130485
C	-4.273965	6.052661	-0.377886
H	-2.631024	5.282414	-1.568550
H	-2.132663	5.905556	0.007603
C	-5.458790	3.816202	-0.461153
H	-3.862902	2.941372	-1.652712
H	-4.148773	2.114950	-0.123484
C	-5.399334	5.239235	-1.035860
H	-4.215012	7.054433	-0.818684
H	-4.505174	6.189918	0.687970
H	-6.241042	3.231571	-0.959354
H	-5.727432	3.863500	0.603394
H	-6.362148	5.746200	-0.901717
H	-5.217721	5.183304	-2.118527
N	-1.691606	3.246140	0.006190
O	-1.358278	3.217715	-2.297798
O	-1.295630	3.094880	2.294063
O	-4.012775	2.850559	3.116504
H	-3.069259	2.863964	2.869902
H	-4.104179	3.607753	3.709061

Table1_1c_TS_2wat

Property	Value
Charge	0
Electronic Energy, BS1 (a.u.)	-1290.195873
Thermal and entropic correction, BS1 (a.u.)	0.456684
Electronic Energy, BS2 (a.u.)	-1290.666867
Number of Imaginary Frequencies	1
Imaginary frequencies (cm-1)	-486.1626

Molecular Geometry in Cartesian Coordinates

C	-3.982226	0.536565	0.198375
C	-2.734894	0.865737	-0.429313
C	-2.216849	2.215898	-0.272565
C	-3.183097	3.240498	-0.212455
C	-4.566621	2.798443	-0.343393
C	-4.934454	1.541181	0.250491
H	-0.114112	1.743577	-0.355218
H	-2.002401	0.083334	-0.597986
C	-0.850265	2.539032	-0.278765
C	-2.777074	4.582456	-0.149595
H	-5.320008	3.578478	-0.392356
C	-1.421439	4.890599	-0.116840
C	-0.457953	3.869193	-0.183829
H	-3.524053	5.371388	-0.117543
C	-4.161891	-0.030220	-2.754799
C	-3.419340	1.212572	-2.443812
C	-4.375431	2.261997	-2.368307
C	-5.710255	1.665523	-2.665509
H	-2.409906	1.314675	-2.817389
H	-4.189826	3.272308	-2.707318
C	-6.559085	-0.688688	-3.202350
C	-7.602850	-0.876602	-2.093539
C	-7.208439	-0.302707	-4.543384

H	-6.034905	-1.636561	-3.339748
C	-8.649323	-1.917969	-2.516965
H	-8.080213	0.088477	-1.888465
H	-7.095960	-1.215705	-1.186567
C	-8.246250	-1.358704	-4.953311
H	-7.690639	0.674027	-4.438835
H	-6.429854	-0.205614	-5.309350
C	-9.304727	-1.552795	-3.856989
H	-9.408891	-2.017896	-1.732964
H	-8.152181	-2.893470	-2.602423
H	-8.718761	-1.066232	-5.898055
H	-7.738536	-2.316063	-5.138015
H	-10.021546	-2.327405	-4.154012
H	-9.875549	-0.620857	-3.739505
N	-5.512463	0.274924	-2.818782
O	-6.789608	2.222690	-2.754979
O	-3.698675	-1.166548	-2.922305
H	0.597954	4.122826	-0.167467
H	-1.104290	5.926952	-0.046264
H	-4.634842	-2.602455	-2.094660
O	-5.267127	-3.158813	-1.606433
H	-4.728004	-3.632815	-0.962722
H	-1.885750	-1.259556	-2.551174
O	-0.989832	-1.179446	-2.170436
H	-0.700092	-2.088542	-2.031037
C	-4.300505	-0.883271	0.626468
H	-4.761459	-0.844070	1.621945
H	-5.066210	-1.306056	-0.035944
C	-3.115373	-1.853407	0.660496
H	-2.324660	-1.502511	1.331972
H	-2.667749	-1.982508	-0.328899
H	-3.443291	-2.834009	1.021327
C	-6.335405	1.277131	0.771287
H	-6.734648	0.380997	0.283586
H	-6.249984	1.009476	1.834377
C	-7.347243	2.416366	0.618839
H	-7.493704	2.678043	-0.433014
H	-7.030161	3.315417	1.158455
H	-8.315152	2.111070	1.027555

Table1_1c_TSiii_nowat

Property	Value
Charge	0
Electronic Energy, BS1 (a.u.)	-1137.311853
Thermal and entropic correction, BS1 (a.u.)	0.414959
Electronic Energy, BS2 (a.u.)	-1137.707053
Number of Imaginary Frequencies	1
Imaginary frequencies (cm-1)	-503.3273

Molecular Geometry in Cartesian Coordinates

C	1.141224	-0.176182	-0.818208
C	-0.064135	0.294957	-1.434370
C	-1.313672	0.008706	-0.754990
C	-1.267808	0.008733	0.652711
C	0.025951	0.284650	1.249376
C	1.186262	-0.184357	0.549631
H	-2.564344	-0.132588	-2.489376
H	2.024340	-0.363991	-1.420815
H	-0.097582	0.398958	-2.515841
C	-2.547235	-0.164406	-1.405853
C	-2.459587	-0.150552	1.381093
H	0.065205	0.378914	2.331548
H	2.107029	-0.379017	1.090572
C	-3.674496	-0.358880	0.740514
C	-3.718312	-0.371717	-0.688991
H	-2.408278	-0.110670	2.463327
C	-4.961891	-0.531148	1.526976
C	-4.839875	-0.472202	3.051298
H	-5.429598	-1.483427	1.241175
H	-5.673810	0.241134	1.202691
H	-5.823115	-0.601201	3.513139
H	-4.185645	-1.261805	3.435286
H	-4.438379	0.490607	3.384010
C	-5.050402	-0.559258	-1.392505

C	-5.028721	-0.478826	-2.920478
H	-5.753332	0.196096	-1.014614
H	-5.481246	-1.523483	-1.088761
H	-6.038290	-0.615226	-3.319054
H	-4.662693	0.494597	-3.263364
H	-4.390968	-1.253425	-3.358748
C	-1.056341	2.922568	1.132508
C	0.216211	2.327559	0.622461
C	0.152272	2.340364	-0.792675
C	-1.162600	2.944503	-1.176426
H	1.118078	2.500157	1.193720
H	0.998382	2.527731	-1.439529
C	-3.166265	3.852089	0.116221
C	-3.100916	5.299207	-0.404713
C	-4.272217	3.050103	-0.584258
H	-3.373382	3.874299	1.192167
C	-4.463831	5.993424	-0.264046
H	-2.800486	5.274085	-1.458177
H	-2.327252	5.847702	0.145258
C	-5.629081	3.755396	-0.444959
H	-4.010280	2.938021	-1.641763
H	-4.310170	2.048911	-0.152278
C	-5.571836	5.197396	-0.970085
H	-4.406339	7.011930	-0.665230
H	-4.713848	6.089975	0.802308
H	-6.399613	3.183202	-0.975326
H	-5.923837	3.769801	0.614469
H	-6.541069	5.692181	-0.836531
H	-5.370249	5.179377	-2.050299
N	-1.855234	3.201045	0.013274
O	-1.579608	3.176546	-2.298980
O	-1.380273	3.131534	2.288193

Table1_1c_TSii_nowat

Property	Value
Charge	0
Electronic Energy, BS1 (a.u.)	-1137.315663
Thermal and entropic correction, BS1 (a.u.)	0.415271
Electronic Energy, BS2 (a.u.)	-1137.709469
Number of Imaginary Frequencies	1
Imaginary frequencies (cm-1)	-496.1922

Molecular Geometry in Cartesian Coordinates

C	0.159943	-1.217784	1.593911
C	-0.637710	-1.495481	0.434312
C	-1.926704	-0.831445	0.308068
C	-1.981169	0.486227	0.807680
C	-0.736600	0.994140	1.367263
C	0.115568	0.081742	2.074099
H	-2.992488	-2.389746	-0.740836
H	-0.550692	-2.474815	-0.025073
C	-3.042988	-1.378846	-0.344711
C	-3.151593	1.245861	0.651395
H	-0.730474	2.037351	1.666287
C	-4.261754	0.683517	0.032243
C	-4.207323	-0.629604	-0.466418
H	-3.184892	2.265997	1.025024
C	1.631856	1.484519	-0.091893
C	0.259895	1.080790	-0.510816
C	0.338133	-0.248512	-1.000508
C	1.762133	-0.683329	-0.886702
H	-0.397015	1.839862	-0.913437
H	-0.245033	-0.626267	-1.829436
C	3.904184	0.401484	-0.022348
C	4.392841	-0.732056	0.892254
C	4.660467	0.410449	-1.364033
H	4.071062	1.360721	0.480405
C	5.913124	-0.650456	1.100118
H	4.123471	-1.691562	0.438638
H	3.879443	-0.667938	1.857491
C	6.177489	0.490574	-1.141043
H	4.410705	-0.507278	-1.907851
H	4.310623	1.256467	-1.966852
C	6.666679	-0.651019	-0.237869

H	6.244769	-1.484124	1.729544
H	6.154772	0.271574	1.648035
H	6.696840	0.468403	-2.105959
H	6.426537	1.453367	-0.672333
H	7.746074	-0.566643	-0.066138
H	6.499646	-1.610826	-0.746312
N	2.453648	0.354632	-0.245061
O	2.263930	-1.733019	-1.259006
O	2.014258	2.565040	0.325787
H	-5.078653	-1.058422	-0.952556
H	-5.174902	1.262226	-0.071202
C	1.051978	0.549932	3.173049
H	0.876853	-0.077194	4.058116
H	2.086062	0.335914	2.871244
C	0.956365	2.026906	3.564987
H	-0.049399	2.289890	3.910191
H	1.216445	2.673534	2.721856
H	1.653585	2.242480	4.380137
C	1.114651	-2.245807	2.171733
H	2.119121	-1.811984	2.225508
H	0.823280	-2.421859	3.217230
C	1.201859	-3.585345	1.434986
H	1.540322	-3.444398	0.404283
H	0.237099	-4.103775	1.417296
H	1.918862	-4.240654	1.938553

Table1_1c_TSi_nowat

Property	Value
Charge	0
Electronic Energy, BS1 (a.u.)	-1137.311280
Thermal and entropic correction, BS1 (a.u.)	0.414042
Electronic Energy, BS2 (a.u.)	-1137.706766
Number of Imaginary Frequencies	1
Imaginary frequencies (cm-1)	-490.2019

Molecular Geometry in Cartesian Coordinates

C	1.117063	-0.178961	-0.821805
C	-0.102183	0.291099	-1.414261
C	-1.352556	0.001865	-0.737114
C	-1.297869	0.011034	0.672789
C	0.003518	0.299320	1.247552
C	1.171113	-0.178362	0.562565
H	-2.618656	-0.203019	-2.474601
H	-0.155851	0.392491	-2.493374
C	-2.578169	-0.217790	-1.389347
C	-2.471967	-0.185097	1.420472
H	0.035188	0.402776	2.327361
C	-3.667204	-0.450212	0.765749
C	-3.718903	-0.471812	-0.640427
H	-2.429517	-0.146646	2.505073
C	-1.079725	2.937943	1.125699
C	0.188601	2.331925	0.620017
C	0.125257	2.338778	-0.795335
C	-1.180402	2.955608	-1.182994
H	1.091596	2.504324	1.190989
H	0.973894	2.512353	-1.443767
C	-3.185490	3.869111	0.103064
C	-3.104375	5.326357	-0.386991
C	-4.284744	3.088003	-0.631025
H	-3.407589	3.871143	1.176249
C	-4.464234	6.026868	-0.249478
H	-2.790369	5.320080	-1.436989
H	-2.334158	5.858247	0.183803
C	-5.639104	3.798520	-0.489067
H	-4.011101	3.006956	-1.688182
H	-4.333915	2.073074	-0.230108
C	-5.568590	5.251145	-0.983104
H	-4.395772	7.052598	-0.630226
H	-4.725953	6.104503	0.815635
H	-6.408314	3.242977	-1.038237
H	-5.943955	3.792829	0.567591
H	-6.536620	5.748474	-0.849401
H	-5.355808	5.254762	-2.061358
N	-1.876482	3.214589	0.004625

O	-1.588273	3.195693	-2.307499
O	-1.401932	3.155008	2.280629
H	-4.570120	-0.633771	1.340617
H	-4.661783	-0.670125	-1.141397
C	2.475462	-0.424044	1.296169
H	2.888839	-1.380759	0.950873
H	3.212472	0.330380	0.979950
C	2.402831	-0.437613	2.825956
H	1.688521	-1.184950	3.186738
H	2.103804	0.535406	3.229368
H	3.382671	-0.678609	3.248093
C	2.360711	-0.424508	-1.654583
H	3.122087	0.326141	-1.392047
H	2.796263	-1.384320	-1.347024
C	2.171011	-0.429521	-3.174285
H	1.841749	0.545726	-3.547690
H	1.431356	-1.175192	-3.483821
H	3.115661	-0.667496	-3.671639

Table1_1c_TSiv_nowat

Property	Value
Charge	0
Electronic Energy, BS1 (a.u.)	-1137.310024
Thermal and entropic correction, BS1 (a.u.)	0.414096
Electronic Energy, BS2 (a.u.)	-1137.706001
Number of Imaginary Frequencies	1
Imaginary frequencies (cm-1)	-506.7845

Molecular Geometry in Cartesian Coordinates

C	0.331305	-1.160268	1.438151
C	-0.538210	-1.469701	0.343545
C	-1.860653	-0.859587	0.347416
C	-1.936754	0.442476	0.885697
C	-0.683088	1.005629	1.367418
C	0.257549	0.103771	1.960517
H	-2.937428	-2.427747	-0.659718
H	1.124708	-1.846576	1.713803
H	-0.425127	-2.432582	-0.147899
C	-3.002065	-1.428445	-0.233996
C	-3.151888	1.138499	0.826339
H	-0.686774	2.039919	1.701552
H	0.988679	0.471740	2.672688
C	-4.296174	0.561764	0.277742
C	-4.219174	-0.749532	-0.265868
H	-3.203574	2.148479	1.227939
C	-5.570547	1.381628	0.215722
C	-5.698141	2.159584	-1.107226
H	-5.579491	2.092465	1.049694
H	-6.450173	0.744620	0.351216
H	-6.622331	2.746104	-1.131799
H	-4.853772	2.845021	-1.232717
H	-5.701991	1.482110	-1.967225
C	-5.436036	-1.456131	-0.830581
C	-6.237908	-2.195484	0.256569
H	-5.110147	-2.179315	-1.586884
H	-6.090597	-0.748867	-1.349501
H	-7.109723	-2.700379	-0.172126
H	-5.613603	-2.947705	0.749297
H	-6.590581	-1.504007	1.028647
C	1.579621	1.586555	-0.196880
C	0.195011	1.172075	-0.568613
C	0.273201	-0.138056	-1.107526
C	1.707928	-0.552922	-1.077242
H	-0.503281	1.926488	-0.905331
H	-0.356961	-0.503643	-1.907163
C	3.824573	0.443234	-0.068310
C	4.110815	-0.687969	0.934059
C	4.731169	0.343147	-1.307021
H	4.003394	1.407157	0.421584
C	5.593546	-0.699513	1.334190
H	3.841031	-1.641973	0.467228
H	3.470724	-0.560845	1.814735
C	6.211197	0.330106	-0.895937
H	4.482061	-0.577108	-1.845892

H	4.520577	1.184188	-1.977667
C	6.504254	-0.801415	0.101158
H	5.786537	-1.528737	2.024481
H	5.829121	0.224960	1.880571
H	6.843657	0.231221	-1.785620
H	6.469071	1.293578	-0.433203
H	7.557672	-0.779211	0.403861
H	6.335990	-1.768953	-0.392087
N	2.407193	0.478065	-0.437247
O	2.201946	-1.589406	-1.490372
O	1.958953	2.651321	0.258031

Table1_1d_TS_1wat

Property	Value
Charge	0
Electronic Energy, BS1 (a.u.)	-1284.324600
Thermal and entropic correction, BS1 (a.u.)	0.371033
Electronic Energy, BS2 (a.u.)	-1284.802868
Number of Imaginary Frequencies	1
Imaginary frequencies (cm-1)	-492.7688

Molecular Geometry in Cartesian Coordinates

C	-4.162464	0.903498	-0.446765
C	-2.837552	0.984819	0.073963
C	-2.081516	2.192301	-0.212877
C	-2.831715	3.391640	-0.255514
C	-4.258995	3.256763	-0.010979
C	-4.888060	2.075080	-0.508867
H	-0.112630	1.314309	-0.314005
H	-2.296161	0.051675	0.200150
C	-0.685917	2.234659	-0.370594
C	-2.175333	4.619371	-0.446393
H	-4.860981	4.160339	0.022563
C	-0.800978	4.639579	-0.657514
C	-0.056565	3.446685	-0.620989
H	-2.745584	5.543112	-0.440973
O	-4.735929	-0.333657	-0.584300
O	-6.229890	2.111373	-0.759704
C	-3.345757	3.729676	2.638544
C	-4.181920	2.659246	2.021736
C	-3.435792	1.452947	2.091675
C	-2.149315	1.763985	2.781025
H	-5.256205	2.721464	2.141877
H	-3.867340	0.472702	2.247642
C	-1.064970	3.883368	3.707986
C	-0.941521	3.440175	5.175403
C	0.278619	3.774117	2.974600
H	-1.376207	4.930460	3.681826
C	0.130132	4.274327	5.894824
H	-0.674726	2.377697	5.200665
H	-1.913034	3.548081	5.671921
C	1.337119	4.623105	3.693060
H	0.584813	2.723197	2.942780
H	0.155243	4.102507	1.939622
C	1.480550	4.206098	5.164837
H	0.235636	3.931812	6.930738
H	-0.200540	5.321501	5.941359
H	2.299333	4.536027	3.175009
H	1.039156	5.679382	3.636473
H	2.217567	4.840091	5.671771
H	1.862949	3.176650	5.210802
N	-2.137109	3.151592	3.019427
O	-1.255593	0.995393	3.087511
O	-3.635668	4.914119	2.762606
C	-6.740880	0.863124	-1.248222
H	-6.475978	0.750993	-2.307196
H	-7.827440	0.912406	-1.150436
C	-6.164061	-0.295718	-0.446769
H	-6.539517	-1.253453	-0.812577
H	-6.429265	-0.184422	0.613025
H	1.017431	3.476553	-0.776411
H	-0.299077	5.583465	-0.851346
O	-1.513498	6.738078	2.064513
H	-1.093512	6.087657	1.488506

H	-2.325782	6.282141	2.344897

Table1_1d_TS_2wat

Property	Value
Charge	0
Electronic Energy, BS1 (a.u.)	-1360.770386
Thermal and entropic correction, BS1 (a.u.)	0.393419
Electronic Energy, BS2 (a.u.)	-1361.284171
Number of Imaginary Frequencies	1
Imaginary frequencies (cm-1)	-493.6768

Molecular Geometry in Cartesian Coordinates

C	-4.169587	0.905552	-0.377559
C	-2.857417	1.017264	0.193102
C	-2.109476	2.228529	-0.127489
C	-2.878387	3.411702	-0.212761
C	-4.309204	3.271006	-0.039887
C	-4.911946	2.064763	-0.481874
H	-0.124850	1.383061	-0.163260
H	-2.297027	0.091991	0.299039
C	-0.712892	2.290812	-0.257695
C	-2.242232	4.648079	-0.434058
H	-4.909575	4.175157	-0.009110
C	-0.864466	4.686196	-0.613866
C	-0.099540	3.508025	-0.524967
H	-2.851449	5.543492	-0.483944
O	-4.716686	-0.341519	-0.484015
O	-6.250523	2.067518	-0.765153
C	-3.439171	3.607810	2.818611
C	-4.220404	2.568569	2.136734
C	-3.422860	1.390405	2.084828
C	-2.128111	1.718789	2.780632
H	-5.299164	2.596194	2.195044
H	-3.825201	0.398513	2.260060
C	-1.130980	3.817535	3.833790
C	-0.887861	3.240347	5.238492
C	0.167341	3.890748	3.019303
H	-1.523862	4.832618	3.943248
C	0.170998	4.070523	5.980647
H	-0.550975	2.203078	5.137326
H	-1.831940	3.227848	5.795366
C	1.218139	4.723333	3.767383
H	0.535306	2.873528	2.848194
H	-0.043419	4.322864	2.037728
C	1.475960	4.165092	5.175103
H	0.359311	3.634146	6.968106
H	-0.219659	5.083338	6.154154
H	2.148679	4.752203	3.189071
H	0.862306	5.760649	3.844021
H	2.204649	4.789123	5.705355
H	1.919318	3.163235	5.088895
N	-2.184766	3.077347	3.125572
O	-1.188546	0.976612	2.993955
O	-3.781008	4.763161	3.087741
C	-6.714863	0.804494	-1.256893
H	-6.403320	0.681297	-2.302174
H	-7.805394	0.831440	-1.206271
C	-6.152575	-0.331643	-0.414367
H	-6.489017	-1.302855	-0.782232
H	-6.465173	-0.211932	0.630977
H	0.977087	3.553003	-0.656913
H	-0.373707	5.631345	-0.829477
O	-4.689507	6.435268	0.914927
H	-3.841778	6.866044	1.119394
H	-4.778836	5.834674	1.672031
O	-2.032922	6.755108	2.131449
H	-2.574983	6.153828	2.674109
H	-1.576308	6.152294	1.528927

Table1_1d_TSiii_nowat

Property	Value
Charge	0
Electronic Energy, BS1 (a.u.)	-1207.884054
Thermal and entropic correction, BS1 (a.u.)	0.349765
Electronic Energy, BS2 (a.u.)	-1208.323958
Number of Imaginary Frequencies	1
Imaginary frequencies (cm-1)	-502.8531

Molecular Geometry in Cartesian Coordinates

```
C          -4.145789        0.842378       -0.441357
C          -2.817663        0.910848        0.092893
C          -2.041748        2.101111       -0.202458
C          -2.771405        3.312095       -0.283826
C          -4.202675        3.206398       -0.068633
C          -4.850867        2.012330       -0.525509
H          -0.061436        1.206563       -0.252500
H          -4.614083       -0.121514       -0.613938
H          -2.267592       -0.011983        0.256781
C          -0.644348        2.118447       -0.325170
C          -2.089014        4.526652       -0.453150
H          -4.768938        4.133319       -0.034680
H          -5.908696        2.026616       -0.768352
C          -0.711090        4.532482       -0.605621
C           0.015752        3.314804       -0.558278
H          -2.620037        5.472375       -0.468664
O           1.380256        3.289690       -0.688450
O          -0.080476        5.737369       -0.786135
C          -3.327186        3.718296        2.600815
C          -4.166340        2.636235        2.003471
C          -3.429173        1.429965        2.094452
C          -2.126441        1.749552        2.757643
H          -5.239114        2.706034        2.120564
H          -3.867395        0.460955        2.290390
C          -1.031768        3.884298        3.626133
C          -0.923725        3.482926        5.108210
C           0.316589        3.740755        2.906872
H          -1.355815        4.929619        3.567678
C           0.169945        4.297245        5.815705
H          -0.688677        2.413770        5.160037
H          -1.894355        3.630955        5.595722
C           1.403685        4.556125        3.621304
H           0.593368        2.681960        2.880466
H           0.204946        4.069346        1.872828
C           1.522577        4.159199        5.100454
H           0.254025        3.979283        6.861347
H          -0.121483        5.357279        5.832782
H           2.363835        4.421680        3.108916
H           1.158854        5.626522        3.555236
H           2.282525        4.770633        5.601047
H           1.860305        3.115515        5.166068
N          -2.101425        3.135464        2.957376
O          -1.237877        0.975438        3.072145
O          -3.609883        4.893189        2.755168
C           1.329552        5.700600       -0.548200
H           1.526094        5.658224        0.531735
H           1.736285        6.631393       -0.950109
C           1.938425        4.488865       -1.232109
H           3.016066        4.434155       -1.061767
H           1.745569        4.534741       -2.312514
```

Table1_1d_TSii_nowat

Property	Value
Charge	0
Electronic Energy, BS1 (a.u.)	-1207.879419
Thermal and entropic correction, BS1 (a.u.)	0.349113
Electronic Energy, BS2 (a.u.)	-1208.322256

Property	Value
Number of Imaginary Frequencies	1
Imaginary frequencies (cm-1)	-500.3529

Molecular Geometry in Cartesian Coordinates

C	-3.774454	0.488743	0.375033
C	-2.656998	0.908320	-0.408255
C	-2.202570	2.280425	-0.203382
C	-3.218746	3.233056	0.046610
C	-4.581098	2.718983	0.061011
C	-4.784546	1.410474	0.574515
H	-0.097908	1.968398	-0.561083
H	-1.915547	0.144378	-0.626240
C	-0.873025	2.701386	-0.354121
C	-2.887371	4.593772	0.145136
H	-5.408859	3.416927	0.147854
C	-1.561487	4.994429	0.026522
C	-0.553739	4.048463	-0.223113
H	-3.671191	5.325110	0.322662
C	-4.372084	0.006200	-2.491999
C	-3.565462	1.247197	-2.244799
C	-4.495342	2.311589	-2.111330
C	-5.855649	1.774430	-2.357471
H	-2.622456	1.343508	-2.768491
H	-4.295239	3.338635	-2.382319
C	-6.779082	-0.590647	-2.782673
C	-8.150224	-0.284243	-2.159943
C	-6.891969	-0.703074	-4.314783
H	-6.418868	-1.553867	-2.399742
C	-9.151309	-1.388191	-2.534405
H	-8.501876	0.681788	-2.529138
H	-8.067002	-0.186769	-1.073492
C	-7.913978	-1.779226	-4.710653
H	-7.199032	0.274381	-4.709269
H	-5.906410	-0.935971	-4.730605
C	-9.281582	-1.524973	-4.059372
H	-10.126944	-1.168834	-2.085923
H	-8.819464	-2.347229	-2.110865
H	-8.008795	-1.821174	-5.801800
H	-7.539043	-2.761821	-4.390921
H	-9.979772	-2.331304	-4.312687
H	-9.707874	-0.597180	-4.465881
N	-5.728128	0.370271	-2.411874
O	-6.891198	2.401041	-2.504199
O	-3.974234	-1.121705	-2.719509
H	0.478834	4.371008	-0.317345
H	-1.303725	6.044813	0.123942
O	-3.883462	-0.828236	0.710570
O	-6.014732	1.063901	1.070360
C	-6.227149	-0.354419	1.080634
H	-7.088443	-0.536235	1.726940
H	-6.450983	-0.697901	0.065557
C	-4.989520	-1.078639	1.587597
H	-5.140541	-2.160072	1.597285
H	-4.737403	-0.736714	2.599800

Table1_1d_TSi_nowat

Property	Value
Charge	0
Electronic Energy, BS1 (a.u.)	-1207.884110
Thermal and entropic correction, BS1 (a.u.)	0.349746
Electronic Energy, BS2 (a.u.)	-1208.324106
Number of Imaginary Frequencies	1
Imaginary frequencies (cm-1)	-496.35

Molecular Geometry in Cartesian Coordinates

C	-4.172073	0.867294	-0.444713
C	-2.843859	0.924810	0.067651
C	-2.058786	2.107706	-0.237231
C	-2.778637	3.324909	-0.297127

C	-4.208865	3.229654	-0.050790
C	-4.868109	2.055168	-0.526852
H	-0.111755	1.180669	-0.317929
H	-2.326305	-0.018974	0.213103
C	-0.661972	2.114271	-0.387120
C	-2.090546	4.533536	-0.494621
H	-4.788134	4.148567	-0.036522
C	-0.715775	4.519503	-0.691496
C	-0.001938	3.308981	-0.641677
H	-2.643474	5.467932	-0.508571
O	-4.778645	-0.358621	-0.557392
O	-6.211084	2.120488	-0.773211
C	-3.311826	3.747878	2.600000
C	-4.153753	2.674519	1.985986
C	-3.423990	1.460843	2.085619
C	-2.127728	1.769146	2.758832
H	-5.228027	2.748133	2.102472
H	-3.867974	0.490099	2.264325
C	-1.024869	3.893396	3.644849
C	-0.930722	3.481251	5.125202
C	0.326801	3.744761	2.931484
H	-1.341398	4.941277	3.591193
C	0.164402	4.280250	5.847113
H	-0.705159	2.409784	5.171129
H	-1.903906	3.633791	5.606418
C	1.414686	4.545217	3.662968
H	0.594302	2.683266	2.904639
H	0.226429	4.080297	1.896655
C	1.519984	4.134930	5.139399
H	0.237777	3.953655	6.890997
H	-0.117456	5.342802	5.870007
H	2.377786	4.409766	3.157047
H	1.178746	5.617749	3.604124
H	2.282511	4.735052	5.649957
H	1.847257	3.087467	5.199578
N	-2.095841	3.155462	2.966743
O	-1.244887	0.988875	3.075940
O	-3.589096	4.924642	2.751706
C	-6.756491	0.875688	-1.229889
H	-6.502401	0.732229	-2.287939
H	-7.840970	0.954176	-1.126254
C	-6.202732	-0.279687	-0.407053
H	-6.607864	-1.235053	-0.746954
H	-6.455385	-0.136395	0.652191
H	1.074195	3.311520	-0.786437
H	-0.187018	5.449787	-0.876373

Table1_1d_TSiv_nowat

Property	Value
Charge	0
Electronic Energy, BS1 (a.u.)	-1207.881700
Thermal and entropic correction, BS1 (a.u.)	0.349369
Electronic Energy, BS2 (a.u.)	-1208.322256
Number of Imaginary Frequencies	1
Imaginary frequencies (cm-1)	-508.7365

Molecular Geometry in Cartesian Coordinates

C	-4.058600	0.578776	0.138858
C	-2.766068	0.917062	-0.377258
C	-2.256541	2.249834	-0.087611
C	-3.224357	3.280867	-0.037844
C	-4.599890	2.870128	-0.281971
C	-4.995035	1.576139	0.188389
H	-0.129966	1.800022	-0.057125
H	-4.318664	-0.459952	0.313352
H	-2.042446	0.118233	-0.516444
C	-0.894775	2.567638	0.007129
C	-2.817479	4.611636	0.128290
H	-5.356496	3.647957	-0.342842
H	-6.038139	1.370898	0.403333
C	-1.469572	4.915194	0.267431
C	-0.498404	3.885100	0.193571
H	-3.538768	5.421610	0.171901
O	0.841542	4.152066	0.304054

O	-1.113962	6.224295	0.463604
C	-4.029982	0.098244	-2.754645
C	-3.323753	1.359687	-2.386105
C	-4.296283	2.392056	-2.332436
C	-5.618275	1.783046	-2.668085
H	-2.304249	1.502781	-2.718361
H	-4.108719	3.417230	-2.622661
C	-6.438774	-0.603401	-3.016572
C	-7.416569	-0.647858	-1.829830
C	-7.177601	-0.392413	-4.349251
H	-5.892542	-1.552374	-3.063818
C	-8.486286	-1.728632	-2.045623
H	-7.890723	0.335353	-1.733588
H	-6.856035	-0.833713	-0.906338
C	-8.246058	-1.476633	-4.554455
H	-7.644498	0.598116	-4.334173
H	-6.451240	-0.401115	-5.170104
C	-9.229062	-1.522703	-3.374598
H	-9.191624	-1.727258	-1.206650
H	-8.006556	-2.717842	-2.050648
H	-8.781897	-1.298201	-5.493809
H	-7.756480	-2.456109	-4.653716
H	-9.967484	-2.318977	-3.524596
H	-9.786474	-0.576507	-3.332855
N	-5.398662	0.407200	-2.810388
O	-6.699355	2.339169	-2.774515
O	-3.559390	-1.009139	-2.947406
C	0.224319	6.399793	0.937508
H	0.282155	6.129791	2.000650
H	0.457576	7.460780	0.823037
C	1.176446	5.532207	0.132607
H	2.207300	5.646500	0.475368
H	1.117418	5.801183	-0.930763

Table1_1e_TSiii_nowat

Property	Value
Charge	0
Electronic Energy, BS1 (a.u.)	-1248.251831
Thermal and entropic correction, BS1 (a.u.)	0.394908
Electronic Energy, BS2 (a.u.)	-1248.698560
Number of Imaginary Frequencies	1
Imaginary frequencies (cm-1)	-414.2089

Molecular Geometry in Cartesian Coordinates

C	-4.177872	0.811127	-0.376545
C	-2.789499	0.840475	0.047388
C	-2.051857	2.058979	-0.205268
C	-2.786140	3.282905	-0.188648
C	-4.193988	3.207691	0.080724
C	-4.898434	2.037958	-0.373438
H	-0.079929	1.197560	-0.424124
C	-0.652684	2.114702	-0.436874
C	-2.116575	4.513663	-0.369334
H	-4.745466	4.142290	0.147959
C	-0.762205	4.534232	-0.640681
C	-0.033781	3.326255	-0.681389
H	-2.688712	5.435739	-0.323730
C	-3.351043	3.715684	2.736909
C	-4.171196	2.610306	2.152977
C	-3.417067	1.430049	2.256027
C	-2.115221	1.781712	2.865988
H	-5.248429	2.665795	2.230178
H	-3.810422	0.429735	2.363715
C	-1.027897	3.929908	3.706088
C	-0.885695	3.548679	5.190260
C	0.305357	3.797640	2.956282
H	-1.367224	4.969465	3.642517
C	0.209028	4.388458	5.864881
H	-0.632090	2.484265	5.253574
H	-1.848644	3.688528	5.695348
C	1.391146	4.641620	3.640304
H	0.603120	2.744381	2.943672
H	0.167569	4.108117	1.917737
C	1.547193	4.265598	5.121206

H	0.320539	4.080677	6.911164
H	-0.101147	5.443120	5.878566
H	2.342897	4.517065	3.110530
H	1.126343	5.706243	3.564452
H	2.305827	4.898150	5.597632
H	1.906211	3.229390	5.193626
N	-2.104707	3.158467	3.069763
O	-1.185417	1.027497	3.155463
O	-3.656391	4.883181	2.896767
H	1.031663	3.348931	-0.891549
H	-0.255748	5.478749	-0.816804
C	-6.272112	2.053667	-0.690891
H	-6.809308	2.998010	-0.663764
C	-6.919033	0.885248	-1.051944
H	-7.971259	0.904409	-1.320089
C	-6.209818	-0.331852	-1.074661
C	-4.870023	-0.373931	-0.728207
H	-6.718069	-1.246139	-1.366958
H	-4.350396	-1.324904	-0.755678
C	-2.090258	-0.505942	0.221349
H	-2.783152	-1.180732	0.746358
H	-1.932858	-0.939220	-0.776926
O	-0.832576	-0.490167	0.853019
H	-0.934916	-0.093716	1.740659

Table1_1e_TSii_nowat

Property	Value
Charge	0
Electronic Energy, BS1 (a.u.)	-1248.230681
Thermal and entropic correction, BS1 (a.u.)	0.393389
Electronic Energy, BS2 (a.u.)	-1248.680787
Number of Imaginary Frequencies	1
Imaginary frequencies (cm-1)	-493.1103

Molecular Geometry in Cartesian Coordinates

C	-4.091691	0.566574	0.174803
C	-2.815435	0.905521	-0.353823
C	-2.305515	2.245812	-0.087676
C	-3.276311	3.293684	-0.031781
C	-4.659891	2.857854	-0.244195
C	-5.030057	1.567908	0.240385
H	-0.220641	1.743559	-0.042777
H	-4.350770	-0.471806	0.353946
H	-2.087263	0.115048	-0.512907
C	-0.957869	2.540697	0.015215
C	-2.883183	4.627468	0.114137
H	-5.432352	3.613527	-0.320892
H	-6.068895	1.357434	0.472022
C	-1.494527	4.937883	0.242274
C	-0.524439	3.869067	0.200851
C	-4.076320	0.130136	-2.766899
C	-3.399552	1.403619	-2.397491
C	-4.393042	2.406590	-2.298929
C	-5.708593	1.762797	-2.604089
H	-2.379828	1.573031	-2.715092
H	-4.256598	3.451858	-2.543199
C	-6.468657	-0.637844	-2.999794
C	-7.460429	-0.728701	-1.828050
C	-7.193651	-0.424196	-4.339793
H	-5.899547	-1.572223	-3.056025
C	-8.496385	-1.834794	-2.077724
H	-7.964771	0.237893	-1.720909
H	-6.908293	-0.916134	-0.899769
C	-8.229816	-1.532409	-4.578505
H	-7.688289	0.552733	-4.317132
H	-6.455421	-0.400713	-5.149819
C	-9.226121	-1.627272	-3.413302
H	-9.213595	-1.865372	-1.249077
H	-7.989311	-2.810238	-2.091380
H	-8.758552	-1.349063	-5.521166
H	-7.712171	-2.495923	-4.690324
H	-9.938431	-2.442674	-3.587201
H	-9.811869	-0.698722	-3.363310
N	-5.455287	0.395332	-2.764435

O	-6.805580	2.290953	-2.682902
O	-3.578083	-0.956464	-3.008681
C	-0.996176	6.267464	0.406758
C	0.349829	6.522919	0.522194
H	0.694558	7.545825	0.644287
C	1.294610	5.465573	0.485009
H	2.354372	5.683643	0.579513
C	0.862429	4.172321	0.328710
H	1.574527	3.351608	0.296454
H	-1.686762	7.101983	0.440212
C	-3.945737	5.704282	0.039105
H	-4.793523	5.446218	0.679882
H	-3.576745	6.671080	0.395543
O	-4.491879	5.824796	-1.282883
H	-3.759404	6.047997	-1.874419

Table1_1e_TSi_nowat

Property	Value
Charge	0
Electronic Energy, BS1 (a.u.)	-1248.238261
Thermal and entropic correction, BS1 (a.u.)	0.395131
Electronic Energy, BS2 (a.u.)	-1248.683860
Number of Imaginary Frequencies	1
Imaginary frequencies (cm-1)	-482.8275

Molecular Geometry in Cartesian Coordinates

C	-4.221378	0.895916	-0.479356
C	-2.890501	0.887313	0.027722
C	-2.049211	2.060403	-0.198179
C	-2.769681	3.302250	-0.240005
C	-4.209125	3.233546	-0.014615
C	-4.898041	2.088298	-0.510888
H	-4.721220	-0.046654	-0.681906
H	-2.388226	-0.061041	0.156040
C	-0.650880	2.043487	-0.297959
C	-2.101040	4.499079	-0.414282
H	-4.737838	4.180898	0.048850
H	-5.958673	2.142014	-0.736923
C	-0.708852	4.509781	-0.623288
C	0.028400	3.270679	-0.578732
H	-2.650400	5.436560	-0.409646
C	-3.385145	3.703043	2.658466
C	-4.192560	2.612252	2.034771
C	-3.426733	1.424557	2.110383
C	-2.121764	1.782840	2.734153
H	-5.267347	2.654182	2.147023
H	-3.826783	0.436764	2.290652
C	-1.070637	3.917919	3.651625
C	-0.903759	3.466409	5.113338
C	0.259368	3.864750	2.886915
H	-1.439757	4.949062	3.643513
C	0.174011	4.301153	5.821122
H	-0.620351	2.407781	5.121485
H	-1.865248	3.553799	5.632812
C	1.330186	4.698229	3.605442
H	0.585348	2.822174	2.813493
H	0.109417	4.233099	1.871030
C	1.509148	4.251428	5.063373
H	0.301739	3.944981	6.850055
H	-0.163706	5.345178	5.889183
H	2.277182	4.624864	3.058175
H	1.038002	5.758014	3.585096
H	2.254939	4.878476	5.566610
H	1.895348	3.222524	5.080644
N	-2.134225	3.149490	2.986746
O	-1.175137	1.039192	2.988249
O	-3.701835	4.862035	2.852761
C	1.439274	3.344915	-0.801774
C	2.073744	4.545728	-1.017124
C	1.342498	5.760640	-1.031009
H	1.858890	6.701991	-1.194537
C	-0.016880	5.736110	-0.844424
H	-0.594271	6.656922	-0.859784
H	2.033090	2.439234	-0.814993

```
H          3.147419        4.563903       -1.181328
C          0.181555        0.767496       -0.147309
H          0.356647        0.330038       -1.139438
H          1.166512        1.042695        0.250077
O         -0.365541       -0.270997        0.647478
H         -0.549554        0.089300        1.536846
```

Table1_Anthracene_ref

Property	Value
Charge	0
Electronic Energy, BS1 (a.u.)	-654.094933
Thermal and entropic correction, BS1 (a.u.)	0.185005
Electronic Energy, BS2 (a.u.)	-654.147042
Number of Imaginary Frequencies	0
Imaginary frequencies (cm-1)	None

Molecular Geometry in Cartesian Coordinates

```
C         -4.099959        0.549562       -0.126515
C         -2.770755        0.853416       -0.001143
C         -2.331228        2.211995        0.081120
C         -3.305560        3.277435        0.042215
C         -4.683463        2.910894       -0.108166
C         -5.064414        1.595592       -0.186696
H         -0.242293        1.725222        0.233168
H         -4.423930       -0.485334       -0.189166
H         -2.022034        0.066107        0.035558
C         -0.974307        2.529063        0.199191
C         -2.883516        4.626145        0.155608
H         -5.430025        3.689554       -0.202645
H         -6.114918        1.345574       -0.306220
C         -1.502805        4.932439        0.237068
C         -0.534844        3.853299        0.267024
C         -0.985620        6.270982        0.289651
C          0.359490        6.520228        0.374350
H          0.715147        7.546264        0.405837
C          1.300541        5.452034        0.419065
H          2.361870        5.671069        0.491393
C          0.860185        4.157950        0.364914
H          1.565020        3.330711        0.391112
H         -1.666692        7.112632        0.247556
C         -3.940078        5.714133        0.175682
H         -4.705747        5.467748        0.918753
H         -3.516318        6.674802        0.486005
O         -4.645325        5.850188       -1.063715
H         -3.982360        5.891816       -1.766712
```

Table1_cage

Property	Value
Charge	12
Electronic Energy, BS1 (a.u.)	-0.157641
Thermal and entropic correction, BS1 (a.u.)	2.279996
Electronic Energy, BS2 (a.u.)	-0.157641
Number of Imaginary Frequencies	0
Imaginary frequencies (cm-1)	None

Molecular Geometry in Cartesian Coordinates

```
C          8.078818        0.879201        6.790902
H          8.343864        0.355411        7.718228
H          7.379441        1.678438        7.055844
H          7.539014        0.157210        6.165073
C          9.393794        0.992410        4.697014
```

H	8.634162	0.247948	4.426786
H	9.284376	1.842810	4.016390
H	10.370180	0.534266	4.494070
C	10.513336	1.328117	6.881669
H	11.266148	0.771080	6.308607
H	10.345723	0.721217	7.781176
C	11.184578	2.655014	7.343900
H	12.213251	2.607556	6.964896
H	11.308381	2.557537	8.429771
C	11.486278	4.789350	6.150016
H	11.028635	4.951975	5.169866
H	11.717183	5.775951	6.571088
H	12.450643	4.298531	5.969829
C	10.228111	4.737701	8.247832
H	9.145004	4.876930	8.310620
H	10.532980	4.219230	9.165189
H	10.688472	5.732800	8.289747
N	2.746301	15.273534	10.906790
N	-0.244354	15.046879	10.922739
C	3.643383	14.266779	11.534515
H	3.355544	13.261387	11.213516
H	4.698256	14.406410	11.266837
H	3.600447	14.271280	12.630584
C	3.529980	16.243197	10.095571
H	3.187647	16.220323	9.056866
H	3.426530	17.279974	10.438631
H	4.605541	16.026659	10.090156
C	1.933602	15.991321	11.954315
H	2.263842	15.691832	12.957006
H	2.145203	17.067647	11.926962
C	0.381186	15.872211	11.978901
H	-0.020761	16.893556	11.950891
H	0.103708	15.492735	12.971151
C	-1.098467	15.820801	9.992089
H	-2.105454	15.394370	9.899665
H	-1.225626	16.863790	10.309039
H	-0.650198	15.835520	8.993522
C	-0.898093	13.821206	11.436689
H	-0.360001	12.934258	11.087390
H	-0.915166	13.783075	12.533412
H	-1.939616	13.734095	11.102104
N	2.385199	1.130769	-6.113842
N	2.344542	3.490259	-7.828851
C	3.607849	0.608574	-5.447733
H	3.385045	-0.095534	-4.636185
H	4.282801	0.083340	-6.134936
H	4.179524	1.435594	-5.016906
C	1.160001	0.523628	-5.529457
H	1.376663	-0.166793	-4.704750
H	0.505509	1.307847	-5.138233
H	0.569592	-0.044423	-6.259098
C	2.442060	0.905704	-7.587922
H	1.609438	0.271808	-7.918902
H	3.338445	0.334874	-7.862095
C	2.425763	2.158750	-8.498236
H	3.319133	2.093190	-9.132484
H	1.590730	2.021755	-9.197169
C	1.095565	4.202284	-8.208970
H	1.281244	5.206694	-8.609685
H	0.510285	3.670798	-8.969704
H	0.448018	4.312671	-7.334568
C	3.542249	4.315238	-8.139292
H	3.288320	5.296108	-8.560451
H	4.121589	4.489000	-7.227954
H	4.220568	3.838590	-8.857896
C	-6.816320	15.385814	-0.247862
H	-7.285855	14.993035	0.663026
H	-5.737810	15.442038	-0.068753
H	-7.180688	16.413652	-0.371633
C	-7.841434	13.302838	-1.106562
H	-8.130369	13.257795	-0.048834
H	-8.765471	13.204896	-1.690555
H	-7.224424	12.424473	-1.321614
C	-7.660178	15.288906	-2.575814
H	-7.876653	16.324399	-2.281961
H	-8.635859	14.871397	-2.857426
C	-6.800759	15.352325	-3.872863
H	-7.475000	15.039418	-4.680240
H	-6.640515	16.419338	-4.072331
C	-5.608922	13.487176	-4.958070
H	-4.853156	13.541459	-5.751669
H	-5.479534	12.527587	-4.448230
H	-6.583347	13.448191	-5.460332
C	-4.389654	15.516272	-4.338365
H	-3.839453	15.190541	-5.229981
H	-4.723933	16.541054	-4.542457
H	-3.670534	15.577175	-3.516840
N	7.989073	13.625066	0.042186

C	7.139571	14.190334	0.924648
H	6.792375	15.188590	0.699473
C	6.703278	13.547320	2.099224
H	6.024581	14.076865	2.755578
C	7.158001	12.238635	2.399924
C	8.043141	11.655317	1.458617
H	8.445994	10.660336	1.598383
C	8.424167	12.377189	0.311344
H	9.101333	11.930891	-0.402611
C	6.753015	11.550440	3.582897
C	5.592711	11.507265	5.532086
C	4.737896	12.151322	6.476350
C	4.239380	13.461698	6.266719
H	4.484219	14.025975	5.376013
C	3.411528	14.061856	7.235155
H	3.036830	15.062609	7.073996
C	3.512377	12.202553	8.592971
H	3.218099	11.720912	9.514416
C	4.343976	11.521338	7.683547
H	4.672996	10.521351	7.935632
C	6.841503	9.716094	4.915458
C	7.339542	8.405414	5.182222
C	6.986047	7.691968	6.355120
H	6.318238	8.113153	7.095597
C	8.221663	7.742457	4.292308
H	8.550276	8.204171	3.370088
C	8.698382	6.454831	4.604574
H	9.373530	5.953892	3.925589
C	7.513871	6.406093	6.581421
H	7.245127	5.866236	7.478188
C	3.409445	3.097923	-2.909209
H	4.338222	3.116417	-3.461180
C	3.430355	3.178666	-1.503656
H	4.388738	3.254315	-1.006168
C	1.106609	2.973603	-2.949332
H	0.200826	2.894656	-3.532939
C	1.028787	3.046350	-1.544844
H	0.051626	3.017038	-1.079994
C	2.214893	3.146358	-0.774520
C	2.188417	3.199856	0.651656
C	1.025278	3.143720	2.598776
C	3.288331	3.304663	2.633750
C	4.510824	3.380616	3.366667
C	4.535859	3.389950	4.784763
H	3.625726	3.345690	5.369015
C	5.771069	3.444202	2.719040
H	5.855345	3.443615	1.639872
C	6.952066	3.503789	3.483910
H	7.913404	3.548991	2.992488
C	5.767074	3.451827	5.465576
H	5.788720	3.455275	6.546138
C	-0.215437	3.026248	3.294603
C	-1.452131	2.912382	2.611112
H	-1.505193	2.918929	1.529963
C	-2.648807	2.776113	3.340930
H	-3.588655	2.685257	2.815478
C	-1.527394	2.864243	5.353242
H	-1.573729	2.843036	6.432575
C	-0.282507	3.004835	4.710478
H	0.607086	3.085302	5.321968
C	-3.734628	13.487029	0.653078
H	-3.518770	14.525292	0.444023
C	-4.758125	11.528141	-0.014448
H	-5.353293	11.010709	-0.753302
C	-4.318909	10.847922	1.137343
H	-4.593243	9.807747	1.256956
C	-3.543093	11.529559	2.109214
C	-3.250540	12.889599	1.833197
H	-2.665142	13.493383	2.514687
C	-3.091636	10.885671	3.300358
C	-3.015306	9.049835	4.631738
C	-1.989372	10.966118	5.282038
N	-4.203277	5.015180	5.446670
C	-3.396043	7.699824	4.896049
C	-4.186116	6.952061	3.986750
H	-4.523739	7.373263	3.048468
C	-4.560315	5.631726	4.301609
H	-5.166478	5.065909	3.608923
C	-3.017484	7.032059	6.088090
H	-2.412810	7.518334	6.842916
C	-3.438994	5.707729	6.316167
H	-3.152079	5.202902	7.227567
C	-1.236889	11.698844	6.248597
C	-0.792394	11.108058	7.458955
H	-1.001915	10.072740	7.695492
C	-0.064342	11.874827	8.389299
H	0.272468	11.426719	9.313198
C	-0.165954	13.749640	7.045004

H	0.089738	14.790109	6.903237
C	-0.896801	13.062312	6.057068
H	-1.190870	13.601253	5.165585
C	3.294664	5.980066	-5.001974
H	4.244870	5.494528	-5.172651
C	0.992280	5.865436	-5.078295
H	0.108270	5.288456	-5.308931
C	0.860846	7.176105	-4.580759
H	-0.134969	7.578007	-4.444280
C	3.262068	7.295504	-4.500026
H	4.201500	7.794227	-4.298692
C	2.017710	7.938222	-4.279588
C	1.934257	9.276651	-3.790661
C	0.687319	11.074569	-3.190157
C	2.952148	11.185609	-3.106470
C	-0.586793	11.697498	-3.029465
C	-1.797093	11.018915	-3.320018
H	-1.802818	9.995023	-3.671119
C	-3.029776	11.682067	-3.164315
H	-3.949877	11.163658	-3.392591
C	-1.993687	13.613554	-2.452395
H	-2.088244	14.635161	-2.112580
C	-0.716207	13.035218	-2.578758
H	0.148754	13.637334	-2.331754
C	4.144027	11.928718	-2.852510
C	4.107848	13.272333	-2.399767
H	3.172152	13.787019	-2.223263
C	5.309840	13.971099	-2.175710
H	5.284134	14.996081	-1.834559
C	5.432810	11.369097	-3.045729
H	5.563882	10.351163	-3.389671
C	6.580878	12.145438	-2.794833
H	7.563892	11.722006	-2.945045
C	11.031577	14.556184	-0.830290
H	10.642250	13.545025	-0.981087
H	11.990461	14.606718	-1.360806
H	11.265240	14.660693	0.236636
C	9.653341	16.480070	-0.208833
H	8.573177	16.429139	-0.043524
H	10.142055	16.240692	0.743902
H	9.890148	17.529952	-0.420842
C	10.586146	16.326401	-2.484066
H	10.678277	17.389375	-2.227801
H	11.620586	16.022674	-2.688010
C	9.864575	16.295910	-3.863674
H	9.653192	17.337830	-4.137991
H	10.600576	15.958585	-4.605258
C	7.407662	16.285957	-4.181928
H	6.757019	16.231332	-3.303267
H	7.628003	17.346161	-4.360446
H	6.831760	15.933823	-5.047186
C	8.752465	14.328626	-4.878836
H	7.948874	14.307139	-5.625825
H	9.698561	14.331906	-5.435063
H	8.708339	13.391522	-4.314782
N	-4.814246	0.863436	5.932184
N	-6.370742	3.198775	6.721394
C	-4.856211	0.270295	4.569051
H	-4.016669	-0.408099	4.371767
H	-5.769549	-0.306293	4.376638
H	-4.820732	1.063102	3.816518
C	-3.655283	0.339708	6.703114
H	-3.036157	-0.356869	6.124045
H	-3.011858	1.166415	7.017246
H	-3.949330	-0.194991	7.614881
C	-6.093825	0.618081	6.659761
H	-5.919057	0.034311	7.572505
H	-6.767744	-0.011569	6.064780
C	-6.916950	1.858695	7.085244
H	-7.922810	1.720671	6.668562
H	-7.063458	1.776799	8.169894
C	-7.291391	3.916897	5.800190
H	-7.578538	4.909003	6.170442
H	-6.811393	4.056748	4.827457
H	-8.227048	3.374618	5.614732
C	-6.097583	4.009201	7.938272
H	-6.591554	4.988852	7.919394
H	-6.421318	3.519442	8.865197
H	-5.022547	4.185469	8.035559
N	9.262006	1.444675	6.101519
N	10.570678	3.990582	7.008051
N	10.039428	15.556194	-1.308755
N	8.632312	15.483235	-3.958429
N	-7.094766	14.539865	-1.432224
N	-5.500721	14.596544	-3.974069
N	5.916387	12.203757	4.419728
N	6.013764	10.259164	5.835553
N	7.247582	10.306547	3.769613

N	8.360114	5.791607	5.729339
N	3.051929	13.453015	8.384091
N	2.272706	2.991144	-3.627287
N	3.378859	3.291991	1.285244
N	0.976994	3.135148	1.247909
N	2.144624	3.236322	3.350626
N	-2.695399	2.745533	4.688947
N	6.955172	3.504716	4.831611
N	-4.477487	12.824111	-0.255648
N	-3.444113	9.592130	3.470554
N	-2.275176	9.677154	5.572772
N	-2.364668	11.628008	4.164643
N	0.242594	13.172518	8.192184
N	2.184189	5.272266	-5.294453
N	0.696881	9.796678	-3.630263
N	1.776708	11.820323	-2.900897
N	3.099787	9.914980	-3.543316
N	6.525813	13.423656	-2.370633
N	-3.136865	12.958618	-2.741964
Pd	1.472182	14.204020	9.531825
Pd	-4.976217	13.704716	-2.082728
Pd	8.737185	3.741945	5.904042
Pd	8.265601	14.460625	-1.857092
Pd	2.299245	3.223295	-5.715770
Pd	-4.517994	2.959293	5.697000

Table1_Et_ref

Property	Value
Charge	0
Electronic Energy, BS1 (a.u.)	-543.190651
Thermal and entropic correction, BS1 (a.u.)	0.210125
Electronic Energy, BS2 (a.u.)	-543.373565
Number of Imaginary Frequencies	0
Imaginary frequencies (cm-1)	None

Molecular Geometry in Cartesian Coordinates

C	2.074331	6.185031	5.962635
C	2.477330	6.320982	4.652458
C	1.558835	6.732445	3.649147
C	0.204157	7.005523	4.019574
C	-0.181190	6.856153	5.379266
C	0.731833	6.455478	6.329699
H	2.958441	6.668305	2.007656
H	2.784850	5.869416	6.721315
H	3.506052	6.113872	4.367664
C	1.928582	6.882391	2.287982
C	-0.700823	7.418527	3.008402
H	-1.211342	7.064795	5.657689
H	0.426343	6.344983	7.366286
C	-0.323375	7.562301	1.687586
C	1.033610	7.282654	1.315099
H	-1.729509	7.631514	3.293819
C	-1.334298	8.075260	0.680383
C	-1.266281	9.604567	0.511547
H	-2.341433	7.798739	1.012337
H	-1.193008	7.594879	-0.293207
H	-2.001529	9.953449	-0.221120
H	-1.467376	10.104147	1.464612
H	-0.274460	9.924547	0.176044
C	1.491665	7.350842	-0.128878
C	1.215554	6.041593	-0.891469
H	2.567888	7.556135	-0.153110
H	1.011784	8.184643	-0.651407
H	1.550646	6.112866	-1.931536
H	1.739847	5.205236	-0.418204
H	0.147698	5.800072	-0.894565

Table1_H_ref

Property	Value
Charge	0
Electronic Energy, BS1 (a.u.)	-385.908951
Thermal and entropic correction, BS1 (a.u.)	0.106275
Electronic Energy, BS2 (a.u.)	-386.044794
Number of Imaginary Frequencies	0
Imaginary frequencies (cm-1)	None

Molecular Geometry in Cartesian Coordinates

```
C      -4.141875      0.817773     -0.235028
C      -2.765542      0.847566     -0.260299
C      -2.065151      2.080863     -0.355176
C      -2.816720      3.300515     -0.424596
C      -4.236366      3.234447     -0.396183
C      -4.884740      2.023305     -0.303663
H      -0.077984      1.221275     -0.330620
H      -4.665037     -0.131313     -0.161890
H      -2.193614     -0.075512     -0.207521
C      -0.645583      2.147017     -0.383526
C      -2.116295      4.533713     -0.519370
H      -4.803767      4.160332     -0.448654
H      -5.970104      1.986575     -0.282469
C      -0.739800      4.563610     -0.544697
C       0.002956      3.358258     -0.476090
H      -2.688035      5.456937     -0.571694
H       1.088338      3.394733     -0.496923
H      -0.216877      5.512859     -0.617465
```

Table1_maleimide_1wat_ref

Property	Value
Charge	0
Electronic Energy, BS1 (a.u.)	-670.582953
Thermal and entropic correction, BS1 (a.u.)	0.190598
Electronic Energy, BS2 (a.u.)	-670.842948
Number of Imaginary Frequencies	0
Imaginary frequencies (cm-1)	None

Molecular Geometry in Cartesian Coordinates

```
C      -0.815474      3.180235      1.106489
C       0.480162      2.668969      0.546367
C       0.401704      2.694457     -0.786614
C      -0.949811      3.228715     -1.174839
H       1.289715      2.343341      1.185628
H       1.131780      2.395420     -1.526678
C      -2.987800      4.038883      0.144636
C      -3.189700      5.355169     -0.620988
C      -4.017496      2.976080     -0.267464
H      -3.124285      4.231420      1.210928
C      -4.615846      5.880090     -0.388223
H      -3.023203      5.180182     -1.688937
H      -2.446827      6.090238     -0.289568
C      -5.438463      3.507889     -0.027789
H      -3.871111      2.728179     -1.325943
H      -3.846829      2.063847      0.315294
C      -5.670592      4.830914     -0.774037
H      -4.767714      6.804816     -0.956505
H      -4.737398      6.138506      0.673119
H      -6.175443      2.758460     -0.338559
H      -5.569613      3.662050      1.051420
H      -6.676899      5.213234     -0.566534
H      -5.618078      4.649934     -1.857013
N      -1.612459      3.532219      0.023279
O      -1.398032      3.367407     -2.295256
```

O	-1.119724	3.261190	2.287329
O	-3.820143	3.585025	3.341364
H	-2.902855	3.376537	3.094832
H	-3.757379	4.476815	3.704975

Table1_maleimide2_ref

Property	Value
Charge	0
Electronic Energy, BS1 (a.u.)	-594.160519
Thermal and entropic correction, BS1 (a.u.)	0.179066
Electronic Energy, BS2 (a.u.)	-594.202755
Number of Imaginary Frequencies	0
Imaginary frequencies (cm-1)	None

Molecular Geometry in Cartesian Coordinates

C	3.090522	8.968765	3.659212
C	3.259431	8.556375	5.093038
C	2.932390	9.590812	5.871838
C	2.529515	10.743196	4.995101
H	3.594945	7.564956	5.368133
H	2.930323	9.667919	6.951279
C	2.364782	11.078628	2.468028
C	0.885612	11.493390	2.401422
C	3.300659	12.293122	2.350492
H	2.575346	10.390548	1.641870
C	0.602535	12.284209	1.115372
H	0.654389	12.110744	3.276628
H	0.254574	10.598608	2.455044
C	3.006977	13.080237	1.064530
H	3.150719	12.937706	3.223774
H	4.342353	11.952217	2.369093
C	1.531620	13.500981	0.990229
H	-0.447225	12.599135	1.097979
H	0.748567	11.627221	0.246104
H	3.660227	13.958830	1.011147
H	3.248804	12.454885	0.193274
H	1.335498	14.033469	0.052036
H	1.315807	14.204898	1.806183
N	2.649371	10.296067	3.677740
O	2.168462	11.850648	5.349632
O	3.290659	8.300809	2.662682

Table1_maleimide_2wat_ref

Property	Value
Charge	0
Electronic Energy, BS1 (a.u.)	-747.018092
Thermal and entropic correction, BS1 (a.u.)	0.210257
Electronic Energy, BS2 (a.u.)	-747.317752
Number of Imaginary Frequencies	0
Imaginary frequencies (cm-1)	None

Molecular Geometry in Cartesian Coordinates

C	-0.909316	3.347910	1.328405
C	0.424743	2.826027	0.880838
C	0.415515	2.762744	-0.453560
C	-0.920498	3.242288	-0.947154
H	1.185569	2.567456	1.607096
H	1.188951	2.430441	-1.132838
C	-3.043657	4.100499	0.210679
C	-3.222083	5.358509	-0.652319
C	-4.031148	2.991813	-0.183166

H	-3.241318	4.361250	1.252487
C	-4.667407	5.869353	-0.534352
H	-2.993672	5.116139	-1.695262
H	-2.510840	6.128423	-0.330965
C	-5.471674	3.510819	-0.060452
H	-3.820945	2.675001	-1.212036
H	-3.879975	2.125380	0.470460
C	-5.681572	4.776172	-0.906446
H	-4.801148	6.750981	-1.171342
H	-4.852762	6.195673	0.498659
H	-6.176976	2.727627	-0.361002
H	-5.666916	3.732928	0.996875
H	-6.703898	5.151673	-0.781685
H	-5.564618	4.524483	-1.970034
N	-1.654809	3.612408	0.197223
O	-1.316972	3.300242	-2.092114
O	-1.281886	3.503008	2.490813
O	1.045167	2.586373	3.938603
H	0.201388	3.004361	3.695244
H	0.797243	1.677308	4.147892
O	-4.069903	3.774128	3.357987
H	-3.130214	3.596591	3.186654
H	-4.071989	4.679085	3.693439

Table1_maleimide_ref

Property	Value
Charge	0
Electronic Energy, BS1 (a.u.)	-594.147418
Thermal and entropic correction, BS1 (a.u.)	0.171530
Electronic Energy, BS2 (a.u.)	-594.368082
Number of Imaginary Frequencies	0
Imaginary frequencies (cm-1)	None

Molecular Geometry in Cartesian Coordinates

C	3.090373	8.966545	3.657597
C	3.256083	8.555808	5.093607
C	2.930708	9.592056	5.871051
C	2.531480	10.746310	4.992739
H	3.588837	7.564180	5.369859
H	2.927604	9.670184	6.950083
C	2.367421	11.077246	2.466775
C	0.887887	11.491992	2.402066
C	3.301184	12.293709	2.349657
H	2.578576	10.387733	1.641387
C	0.602681	12.283264	1.116482
H	0.659937	12.108582	3.278726
H	0.256594	10.597642	2.456953
C	3.005858	13.081460	1.064192
H	3.149041	12.935608	3.224554
H	4.343569	11.955367	2.368283
C	1.529993	13.501814	0.992537
H	-0.447559	12.595604	1.098303
H	0.750201	11.627653	0.246439
H	3.659100	13.959596	1.008922
H	3.245834	12.456505	0.192178
H	1.331977	14.036638	0.056479
H	1.314776	14.203393	1.810532
N	2.652769	10.296172	3.676102
O	2.172740	11.854670	5.344475
O	3.289200	8.298646	2.661881

Table1_Me_ref

Property	Value
Charge	0
Electronic Energy, BS1 (a.u.)	-464.555166

Property	Value
Thermal and entropic correction, BS1 (a.u.)	0.156625
Electronic Energy, BS2 (a.u.)	-464.557366
Number of Imaginary Frequencies	0
Imaginary frequencies (cm-1)	None

Molecular Geometry in Cartesian Coordinates

C	2.080158	6.193527	5.979582
C	2.485612	6.340835	4.671010
C	1.564923	6.740857	3.665839
C	0.204659	6.989990	4.031814
C	-0.183086	6.829591	5.389007
C	0.732598	6.440332	6.342133
H	2.976419	6.714025	2.031041
H	2.793177	5.887127	6.739670
H	3.518839	6.152180	4.389758
C	1.941069	6.904336	2.306396
C	-0.706720	7.389351	3.018801
H	-1.217694	7.019733	5.664111
H	0.424494	6.320974	7.376947
C	-0.322918	7.543013	1.703006
C	1.041470	7.293050	1.335902
H	-1.740693	7.578240	3.300250
C	-1.321179	7.969162	0.654488
H	-2.312732	8.120475	1.088828
H	-1.413734	7.220267	-0.141410
C	1.479390	7.456054	-0.099077
H	2.542319	7.230566	-0.217699
H	1.310753	8.478640	-0.457835
H	-1.020470	8.905546	0.169112
H	0.918470	6.793253	-0.769073

Table1_O_ref

Property	Value
Charge	0
Electronic Energy, BS1 (a.u.)	-613.762637
Thermal and entropic correction, BS1 (a.u.)	0.145386
Electronic Energy, BS2 (a.u.)	-613.990166
Number of Imaginary Frequencies	0
Imaginary frequencies (cm-1)	None

Molecular Geometry in Cartesian Coordinates

C	-4.147962	0.834087	-0.172147
C	-2.771526	0.846015	-0.230013
C	-2.058331	2.067922	-0.369598
C	-2.797215	3.295775	-0.451307
C	-4.216662	3.247881	-0.388169
C	-4.877604	2.046798	-0.251651
H	-0.059917	1.198585	-0.399930
H	-4.679928	-0.106900	-0.065177
H	-2.209290	-0.082561	-0.169715
C	-0.643586	2.113094	-0.443107
C	-2.086978	4.515846	-0.577923
H	-4.773539	4.179653	-0.448903
H	-5.962582	2.025030	-0.204502
C	-0.711650	4.534467	-0.635337
C	0.022803	3.310095	-0.578889
H	-2.619031	5.461159	-0.624429
O	1.392916	3.297506	-0.645535
O	-0.073247	5.742525	-0.758837
C	1.322212	5.702678	-0.449719
H	1.463678	5.630517	0.637464
H	1.745946	6.645073	-0.804562
C	1.969152	4.511755	-1.133445
H	3.038142	4.458015	-0.914353
H	1.827248	4.584027	-2.220554

Table2_1_2_1b

Property	Value
Charge	0
Electronic Energy, BS1 (a.u.)	-1211.561299
Thermal and entropic correction, BS1 (a.u.)	0.403413
Electronic Energy, BS2 (a.u.)	-1211.603763
Number of Imaginary Frequencies	1
Imaginary frequencies (cm-1)	-486.5222

Molecular Geometry in Cartesian Coordinates

```
C          -4.049595      0.476169      0.100082
C          -2.773513      0.791631     -0.464811
C          -2.217872      2.115580     -0.270805
C          -3.151771      3.171722     -0.189446
C          -4.546541      2.780042     -0.351017
C          -4.961339      1.510264      0.195669
H          -0.125507      1.582365     -0.319707
H          -2.088279     -0.033720     -0.643533
C          -0.840627      2.396904     -0.243217
C          -2.703138      4.496974     -0.081923
H          -5.285525      3.579318     -0.340416
C          -1.339486      4.759806     -0.022088
C          -0.408170      3.709345     -0.103729
H          -3.424696      5.308261     -0.034824
C          -4.173450      0.093263     -2.906573
C          -3.468311      1.329964     -2.553007
C          -4.443068      2.341277     -2.345027
C          -5.780625      1.709135     -2.597157
H          -2.460082      1.492041     -2.904357
H          -4.308716      3.367855     -2.661813
C          -6.573875     -0.662510     -3.118732
C          -7.654544     -0.752020     -2.030805
C          -7.190004     -0.400565     -4.504142
H          -6.041729     -1.618394     -3.152579
C          -8.676817     -1.842860     -2.384552
H          -8.153667      0.217896     -1.945168
H          -7.187252     -0.961486     -1.062898
C          -8.225290     -1.481399     -4.850235
H          -7.664774      0.587087     -4.490110
H          -6.391411     -0.372962     -5.254119
C          -9.305236     -1.591394     -3.763537
H          -9.451535     -1.887476     -1.611036
H          -8.177437     -2.822422     -2.387062
H          -8.679022     -1.261869     -5.823151
H          -7.715925     -2.450053     -4.952504
H         -10.012547     -2.391362     -4.010212
H          -9.881806     -0.656547     -3.730917
N          -5.544337      0.338614     -2.802934
O          -6.876894      2.239217     -2.602511
O          -3.691575     -0.995423     -3.234061
H           0.654738      3.927573     -0.061681
H          -0.990175      5.782547      0.083557
H          -4.252927     -2.655078     -2.639351
O          -4.376358     -3.457708     -2.098177
H          -4.862794     -3.143741     -1.326160
H          -2.075682     -1.708072     -2.259869
O          -1.764926     -2.261777     -1.525797
H          -2.432263     -2.968840     -1.531308
C          -4.355422     -0.964520      0.437361
H          -4.304361     -1.136264      1.518940
H          -5.359645     -1.257929      0.114456
C          -6.379137      1.374046      0.683205
H          -6.631958      0.349844      0.960997
H          -6.540541      2.006481      1.564193
H          -7.084051      1.708904     -0.086010
H          -3.622510     -1.620372     -0.037844
```

Table2_1_2*_1c

Property	Value
Charge	0

Property	Value
Electronic Energy, BS1 (a.u.)	-1290.195873
Thermal and entropic correction, BS1 (a.u.)	0.456684
Electronic Energy, BS2 (a.u.)	-1290.666867
Number of Imaginary Frequencies	1
Imaginary frequencies (cm-1)	-486.1626

Molecular Geometry in Cartesian Coordinates

```
C          -3.982226        0.536565        0.198375
C          -2.734894        0.865737       -0.429313
C          -2.216849        2.215898       -0.272565
C          -3.183097        3.240498       -0.212455
C          -4.566621        2.798443       -0.343393
C          -4.934454        1.541181        0.250491
H          -0.114112        1.743577       -0.355218
H          -2.002401        0.083334       -0.597986
C          -0.850265        2.539032       -0.278765
C          -2.777074        4.582456       -0.149595
H          -5.320008        3.578478       -0.392356
C          -1.421439        4.890599       -0.116840
C          -0.457953        3.869193       -0.183829
H          -3.524053        5.371388       -0.117543
C          -4.161891       -0.030220       -2.754799
C          -3.419340        1.212572       -2.443812
C          -4.375431        2.261997       -2.368307
C          -5.710255        1.665523       -2.665509
H          -2.409906        1.314675       -2.817389
H          -4.189826        3.272308       -2.707318
C          -6.559085       -0.688688       -3.202350
C          -7.602850       -0.876602       -2.093539
C          -7.208439       -0.302707       -4.543384
H          -6.034905       -1.636561       -3.339748
C          -8.649323       -1.917969       -2.516965
H          -8.080213        0.088477       -1.888465
H          -7.095960       -1.215705       -1.186567
C          -8.246250       -1.358704       -4.953311
H          -7.690639        0.674027       -4.438835
H          -6.429854       -0.205614       -5.309350
C          -9.304727       -1.552795       -3.856989
H          -9.408891       -2.017896       -1.732964
H          -8.152181       -2.893470       -2.602423
H          -8.718761       -1.066232       -5.898055
H          -7.738536       -2.316063       -5.138015
H         -10.021546       -2.327405       -4.154012
H          -9.875549       -0.620857       -3.739505
N          -5.512463        0.274924       -2.818782
O          -6.789608        2.222690       -2.754979
O          -3.698675       -1.166548       -2.922305
H           0.597954        4.122826       -0.167467
H          -1.104290        5.926952       -0.046264
H          -4.634842       -2.602455       -2.094660
O          -5.267127       -3.158813       -1.606433
H          -4.728004       -3.632815       -0.962722
H          -1.885750       -1.259556       -2.551174
O          -0.989832       -1.179446       -2.170436
H          -0.700092       -2.088542       -2.031037
C          -4.300505       -0.883271        0.626468
H          -4.761459       -0.844070        1.621945
H          -5.066210       -1.306056       -0.035944
C          -3.115373       -1.853407        0.660496
H          -2.324660       -1.502511        1.331972
H          -2.667749       -1.982508       -0.328899
H          -3.443291       -2.834009        1.021327
C          -6.335405        1.277131        0.771287
H          -6.734648        0.380997        0.283586
H          -6.249984        1.009476        1.834377
C          -7.347243        2.416366        0.618839
H          -7.493704        2.678043       -0.433014
H          -7.030161        3.315417        1.158455
H          -8.315152        2.111070        1.027555
```

Table2_1_2_1c

Property	Value
Charge	0
Electronic Energy, BS1 (a.u.)	-1290.236296
Thermal and entropic correction, BS1 (a.u.)	0.453773
Electronic Energy, BS2 (a.u.)	-1290.711941
Number of Imaginary Frequencies	0
Imaginary frequencies (cm-1)	None

Molecular Geometry in Cartesian Coordinates

```
C         -3.877139     0.508953     0.511554
C         -2.568490     0.860201     0.245113
C         -2.173673     2.208038     0.028644
C         -3.166252     3.230721     0.091194
C         -4.512279     2.850357     0.345399
C         -4.883639     1.537089     0.554336
H         -0.094363     1.769635    -0.364421
H         -1.803356     0.098191     0.156463
C         -0.835655     2.560560    -0.288998
C         -2.783956     4.577568    -0.145415
H         -5.261076     3.635782     0.366931
C         -1.475428     4.893969    -0.444860
C         -0.493306     3.875777    -0.521985
H         -3.541618     5.355980    -0.093837
C         -4.155584    -0.080725    -2.910261
C         -3.371488     1.185656    -2.793439
C         -4.232834     2.208625    -2.802504
C         -5.620802     1.661536    -2.936130
H         -2.297395     1.172803    -2.675010
H         -4.037382     3.265774    -2.698819
C         -6.575874    -0.687290    -3.339917
C         -7.585066    -0.800363    -2.189575
C         -7.258862    -0.327978    -4.670855
H         -6.082660    -1.655782    -3.455986
C         -8.682617    -1.816168    -2.538357
H         -8.019416     0.188129    -2.000427
H         -7.056063    -1.129352    -1.292269
C         -8.348595    -1.358160    -5.005569
H         -7.704155     0.668721    -4.585040
H         -6.506960    -0.284639    -5.467733
C         -9.372923    -1.474352    -3.866818
H         -9.415806    -1.859921    -1.724789
H         -8.223818    -2.811639    -2.604466
H         -8.843314    -1.081338    -5.943506
H         -7.881999    -2.339228    -5.173073
H        -10.126777    -2.232006    -4.110893
H         -9.905915    -0.518611    -3.763767
N         -5.488536     0.258150    -3.029374
O         -6.672223     2.269415    -2.958711
O         -3.706728    -1.229510    -2.895615
H          0.531661     4.136732    -0.769620
H         -1.193758     5.926956    -0.628292
H         -4.729086    -2.610180    -2.058239
O         -5.407362    -3.122404    -1.585611
H         -4.922380    -3.586897    -0.893673
H         -1.895931    -1.148122    -2.326253
O         -1.036790    -0.865076    -1.964433
H         -0.380640    -1.362484    -2.466974
C         -4.275178    -0.937726     0.740420
H         -4.836153    -1.003245     1.682642
H         -4.984489    -1.249368    -0.036230
C         -3.130612    -1.954605     0.770637
H         -2.413147    -1.730857     1.567017
H         -2.576677    -1.975456    -0.172464
H         -3.525324    -2.958488     0.962181
C         -6.327334     1.156573     0.827087
H         -6.582731     0.293445     0.205048
H         -6.405931     0.793451     1.862346
C         -7.373559     2.248495     0.588691
H         -7.330446     2.614633    -0.442146
H         -7.238965     3.100691     1.262968
H         -8.377271     1.848680     0.762375
```

Table2_1_2_1c.qfiTable2_1_2_1c

Property	Value
Charge	0
Electronic Energy, BS1 (a.u.)	-1290.236296
Thermal and entropic correction, BS1 (a.u.)	0.471119
Electronic Energy, BS2 (a.u.)	—
Number of Imaginary Frequencies	0
Imaginary frequencies (cm-1)	None

Molecular Geometry in Cartesian Coordinates

C	-3.877139	0.508953	0.511554
C	-2.568490	0.860201	0.245113
C	-2.173673	2.208038	0.028644
C	-3.166252	3.230721	0.091194
C	-4.512279	2.850357	0.345399
C	-4.883639	1.537089	0.554336
H	-0.094363	1.769635	-0.364421
H	-1.803356	0.098191	0.156463
C	-0.835655	2.560560	-0.288998
C	-2.783956	4.577568	-0.145415
H	-5.261076	3.635782	0.366931
C	-1.475428	4.893969	-0.444860
C	-0.493306	3.875777	-0.521985
H	-3.541618	5.355980	-0.093837
C	-4.155584	-0.080725	-2.910261
C	-3.371488	1.185656	-2.793439
C	-4.232834	2.208625	-2.802504
C	-5.620802	1.661536	-2.936130
H	-2.297395	1.172803	-2.675010
H	-4.037382	3.265774	-2.698819
C	-6.575874	-0.687290	-3.339917
C	-7.585066	-0.800363	-2.189575
C	-7.258862	-0.327978	-4.670855
H	-6.082660	-1.655782	-3.455986
C	-8.682617	-1.816168	-2.538357
H	-8.019416	0.188129	-2.000427
H	-7.056063	-1.129352	-1.292269
C	-8.348595	-1.358160	-5.005569
H	-7.704155	0.668721	-4.585040
H	-6.506960	-0.284639	-5.467733
C	-9.372923	-1.474352	-3.866818
H	-9.415806	-1.859921	-1.724789
H	-8.223818	-2.811639	-2.604466
H	-8.843314	-1.081338	-5.943506
H	-7.881999	-2.339228	-5.173073
H	-10.126777	-2.232006	-4.110893
H	-9.905915	-0.518611	-3.763767
N	-5.488536	0.258150	-3.029374
O	-6.672223	2.269415	-2.958711
O	-3.706728	-1.229510	-2.895615
H	0.531661	4.136732	-0.769620
H	-1.193758	5.926956	-0.628292
H	-4.729086	-2.610180	-2.058239
O	-5.407362	-3.122404	-1.585611
H	-4.922380	-3.586897	-0.893673
H	-1.895931	-1.148122	-2.326253
O	-1.036790	-0.865076	-1.964433
H	-0.380640	-1.362484	-2.466974
C	-4.275178	-0.937726	0.740420
H	-4.836153	-1.003245	1.682642
H	-4.984489	-1.249368	-0.036230
C	-3.130612	-1.954605	0.770637
H	-2.413147	-1.730857	1.567017
H	-2.576677	-1.975456	-0.172464
H	-3.525324	-2.958488	0.962181
C	-6.327334	1.156573	0.827087
H	-6.582731	0.293445	0.205048
H	-6.405931	0.793451	1.862346
C	-7.373559	2.248495	0.588691
H	-7.330446	2.614633	-0.442146
H	-7.238965	3.100691	1.262968
H	-8.377271	1.848680	0.762375

Table2_1_2*_1e

Property	Value
Charge	0
Electronic Energy, BS1 (a.u.)	-1248.251831
Thermal and entropic correction, BS1 (a.u.)	0.394908
Electronic Energy, BS2 (a.u.)	-1248.698560
Number of Imaginary Frequencies	1
Imaginary frequencies (cm-1)	-414.2089

Molecular Geometry in Cartesian Coordinates

```
C          -4.177872        0.811127       -0.376545
C          -2.789499        0.840475        0.047388
C          -2.051857        2.058979       -0.205268
C          -2.786140        3.282905       -0.188648
C          -4.193988        3.207691        0.080724
C          -4.898434        2.037958       -0.373438
H          -0.079929        1.197560       -0.424124
C          -0.652684        2.114702       -0.436874
C          -2.116575        4.513663       -0.369334
H          -4.745466        4.142290        0.147959
C          -0.762205        4.534232       -0.640681
C          -0.033781        3.326255       -0.681389
H          -2.688712        5.435739       -0.323730
C          -3.351043        3.715684        2.736909
C          -4.171196        2.610306        2.152977
C          -3.417067        1.430049        2.256027
C          -2.115221        1.781712        2.865988
H          -5.248429        2.665795        2.230178
H          -3.810422        0.429735        2.363715
C          -1.027897        3.929908        3.706088
C          -0.885695        3.548679        5.190260
C           0.305357        3.797640        2.956282
H          -1.367224        4.969465        3.642517
C           0.209028        4.388458        5.864881
H          -0.632090        2.484265        5.253574
H          -1.848644        3.688528        5.695348
C           1.391146        4.641620        3.640304
H           0.603120        2.744381        2.943672
H           0.167569        4.108117        1.917737
C           1.547193        4.265598        5.121206
H           0.320539        4.080677        6.911164
H          -0.101147        5.443120        5.878566
H           2.342897        4.517065        3.110530
H           1.126343        5.706243        3.564452
H           2.305827        4.898150        5.597632
H           1.906211        3.229390        5.193626
N          -2.104707        3.158467        3.069763
O          -1.185417        1.027497        3.155463
O          -3.656391        4.883181        2.896767
H           1.031663        3.348931       -0.891549
H          -0.255748        5.478749       -0.816804
C          -6.272112        2.053667       -0.690891
H          -6.809308        2.998010       -0.663764
C          -6.919033        0.885248       -1.051944
H          -7.971259        0.904409       -1.320089
C          -6.209818       -0.331852       -1.074661
C          -4.870023       -0.373931       -0.728207
H          -6.718069       -1.246139       -1.366958
H          -4.350396       -1.324904       -0.755678
C          -2.090258       -0.505942        0.221349
H          -2.783152       -1.180732        0.746358
H          -1.932858       -0.939220       -0.776926
O          -0.832576       -0.490167        0.853019
H          -0.934916       -0.093716        1.740659
```

Table2_1_2_1e

Property	Value
Charge	0
Electronic Energy, BS1 (a.u.)	-1248.280046

Property	Value
Thermal and entropic correction, BS1 (a.u.)	0.392047
Electronic Energy, BS2 (a.u.)	-1248.731027
Number of Imaginary Frequencies	0
Imaginary frequencies (cm-1)	None

Molecular Geometry in Cartesian Coordinates

```
C          -3.472050        0.952341       -0.137964
C          -2.064884        1.113390       -0.130027
C          -1.494887        2.359086       -0.490768
C          -2.347224        3.460296       -0.874443
C          -3.733176        3.277973       -0.899631
C          -4.315377        2.057327       -0.549499
H           0.582612        1.799817       -0.148725
C          -0.079746        2.590209       -0.478090
C          -1.759163        4.719270       -1.215897
H          -4.373298        4.106123       -1.195295
C          -0.401157        4.892353       -1.191743
C           0.446283        3.809325       -0.820133
H          -2.418128        5.537304       -1.495608
C          -4.071214        4.103375        3.466152
C          -5.242360        3.268722        3.043356
C          -4.828158        2.008030        2.897885
C          -3.362502        1.947990        3.215240
H          -6.225538        3.692817        2.888806
H          -5.373752        1.131373        2.578806
C          -1.614164        3.686723        3.903301
C          -1.022977        2.891225        5.077985
C          -0.697572        3.658516        2.671791
H          -1.749865        4.727446        4.217263
C           0.384109        3.405561        5.419046
H          -0.970631        1.833081        4.801871
H          -1.688757        2.969857        5.945537
C           0.706975        4.164660        3.025545
H          -0.628855        2.633797        2.296406
H          -1.138340        4.263507        1.872606
C           1.307089        3.365894        4.191466
H           0.805449        2.809698        6.237001
H           0.314700        4.439673        5.786012
H           1.348453        4.098470        2.139893
H           0.655689        5.227950        3.301114
H           2.299236        3.754368        4.450886
H           1.445320        2.322098        3.876643
N          -2.972091        3.232354        3.563255
O          -2.652934        0.951958        3.175915
O          -4.042953        5.297456        3.687250
H           1.522111        3.957930       -0.796817
H           0.035216        5.852508       -1.451462
C          -5.736813        1.889957       -0.575330
H          -6.346504        2.732661       -0.891582
C          -6.318087        0.705498       -0.212481
H          -7.397667        0.589347       -0.240299
C          -5.500076       -0.380665        0.213527
C          -4.134676       -0.259718        0.253341
H          -5.966117       -1.314551        0.515100
H          -3.549966       -1.101299        0.602991
C          -1.133127       -0.006570        0.302619
H          -1.660947       -0.964624        0.349176
H          -0.347069       -0.135923       -0.449147
O          -0.458938        0.261513        1.531469
H          -1.141588        0.439782        2.201291
```

Table2_Et_2wat_ref

Property	Value
Charge	0
Electronic Energy, BS1 (a.u.)	-543.190651
Thermal and entropic correction, BS1 (a.u.)	0.210125
Electronic Energy, BS2 (a.u.)	-543.373565

Property	Value
Number of Imaginary Frequencies	0
Imaginary frequencies (cm-1)	None

Molecular Geometry in Cartesian Coordinates

C	2.074331	6.185031	5.962635
C	2.477330	6.320982	4.652458
C	1.558835	6.732445	3.649147
C	0.204157	7.005523	4.019574
C	-0.181190	6.856153	5.379266
C	0.731833	6.455478	6.329699
H	2.958441	6.668305	2.007656
H	2.784850	5.869416	6.721315
H	3.506052	6.113872	4.367664
C	1.928582	6.882391	2.287982
C	-0.700823	7.418527	3.008402
H	-1.211342	7.064795	5.657689
H	0.426343	6.344983	7.366286
C	-0.323375	7.562301	1.687586
C	1.033610	7.282654	1.315099
H	-1.729509	7.631514	3.293819
C	-1.334298	8.075260	0.680383
C	-1.266281	9.604567	0.511547
H	-2.341433	7.798739	1.012337
H	-1.193008	7.594879	-0.293207
H	-2.001529	9.953449	-0.221120
H	-1.467376	10.104147	1.464612
H	-0.274460	9.924547	0.176044
C	1.491665	7.350842	-0.128878
C	1.215554	6.041593	-0.891469
H	2.567888	7.556135	-0.153110
H	1.011784	8.184643	-0.651407
H	1.550646	6.112866	-1.931536
H	1.739847	5.205236	-0.418204
H	0.147698	5.800072	-0.894565

Table2_maleimide_2wat_ref

Property	Value
Charge	0
Electronic Energy, BS1 (a.u.)	-747.018092
Thermal and entropic correction, BS1 (a.u.)	0.210257
Electronic Energy, BS2 (a.u.)	-747.317752
Number of Imaginary Frequencies	0
Imaginary frequencies (cm-1)	None

Molecular Geometry in Cartesian Coordinates

C	-0.909316	3.347910	1.328405
C	0.424743	2.826027	0.880838
C	0.415515	2.762744	-0.453560
C	-0.920498	3.242288	-0.947154
H	1.185569	2.567456	1.607096
H	1.188951	2.430441	-1.132838
C	-3.043657	4.100499	0.210679
C	-3.222083	5.358509	-0.652319
C	-4.031148	2.991813	-0.183166
H	-3.241318	4.361250	1.252487
C	-4.667407	5.869353	-0.534352
H	-2.993672	5.116139	-1.695262
H	-2.510840	6.128423	-0.330965
C	-5.471674	3.510819	-0.060452
H	-3.820945	2.675001	-1.212036
H	-3.879975	2.125380	0.470460
C	-5.681572	4.776172	-0.906446
H	-4.801148	6.750981	-1.171342
H	-4.852762	6.195673	0.498659
H	-6.176976	2.727627	-0.361002
H	-5.666916	3.732928	0.996875
H	-6.703898	5.151673	-0.781685
H	-5.564618	4.524483	-1.970034
N	-1.654809	3.612408	0.197223

O	-1.316972	3.300242	-2.092114
O	-1.281886	3.503008	2.490813
O	1.045167	2.586373	3.938603
H	0.201388	3.004361	3.695244
H	0.797243	1.677308	4.147892
O	-4.069903	3.774128	3.357987
H	-3.130214	3.596591	3.186654
H	-4.071989	4.679085	3.693439

Table2_maleimide_toluene

Property	Value
Charge	0
Electronic Energy, BS1 (a.u.)	-594.160519
Thermal and entropic correction, BS1 (a.u.)	0.179066
Electronic Energy, BS2 (a.u.)	-594.202755
Number of Imaginary Frequencies	0
Imaginary frequencies (cm-1)	None

Molecular Geometry in Cartesian Coordinates

C	3.090522	8.968765	3.659212
C	3.259431	8.556375	5.093038
C	2.932390	9.590812	5.871838
C	2.529515	10.743196	4.995101
H	3.594945	7.564956	5.368133
H	2.930323	9.667919	6.951279
C	2.364782	11.078628	2.468028
C	0.885612	11.493390	2.401422
C	3.300659	12.293122	2.350492
H	2.575346	10.390548	1.641870
C	0.602535	12.284209	1.115372
H	0.654389	12.110744	3.276628
H	0.254574	10.598608	2.455044
C	3.006977	13.080237	1.064530
H	3.150719	12.937706	3.223774
H	4.342353	11.952217	2.369093
C	1.531620	13.500981	0.990229
H	-0.447225	12.599135	1.097979
H	0.748567	11.627221	0.246104
H	3.660227	13.958830	1.011147
H	3.248804	12.454885	0.193274
H	1.335498	14.033469	0.052036
H	1.315807	14.204898	1.806183
N	2.649371	10.296067	3.677740
O	2.168462	11.850648	5.349632
O	3.290659	8.300809	2.662682

Table2_Me_2wat_ref

Property	Value
Charge	0
Electronic Energy, BS1 (a.u.)	-464.555166
Thermal and entropic correction, BS1 (a.u.)	0.156625
Electronic Energy, BS2 (a.u.)	-464.557366
Number of Imaginary Frequencies	0
Imaginary frequencies (cm-1)	None

Molecular Geometry in Cartesian Coordinates

C	2.080158	6.193527	5.979582
C	2.485612	6.340835	4.671010
C	1.564923	6.740857	3.665839
C	0.204659	6.989990	4.031814
C	-0.183086	6.829591	5.389007

C	0.732598	6.440332	6.342133
H	2.976419	6.714025	2.031041
H	2.793177	5.887127	6.739670
H	3.518839	6.152180	4.389758
C	1.941069	6.904336	2.306396
C	-0.706720	7.389351	3.018801
H	-1.217694	7.019733	5.664111
H	0.424494	6.320974	7.376947
C	-0.322918	7.543013	1.703006
C	1.041470	7.293050	1.335902
H	-1.740693	7.578240	3.300250
C	-1.321179	7.969162	0.654488
H	-2.312732	8.120475	1.088828
H	-1.413734	7.220267	-0.141410
C	1.479390	7.456054	-0.099077
H	2.542319	7.230566	-0.217699
H	1.310753	8.478640	-0.457835
H	-1.020470	8.905546	0.169112
H	0.918470	6.793253	-0.769073

Table3_Ant_toluene_ref

Property	Value
Charge	0
Electronic Energy, BS1 (a.u.)	-654.094933
Thermal and entropic correction, BS1 (a.u.)	0.185005
Electronic Energy, BS2 (a.u.)	-654.147042
Number of Imaginary Frequencies	0
Imaginary frequencies (cm-1)	None

Molecular Geometry in Cartesian Coordinates

C	-4.099959	0.549562	-0.126515
C	-2.770755	0.853416	-0.001143
C	-2.331228	2.211995	0.081120
C	-3.305560	3.277435	0.042215
C	-4.683463	2.910894	-0.108166
C	-5.064414	1.595592	-0.186696
H	-0.242293	1.725222	0.233168
H	-4.423930	-0.485334	-0.189166
H	-2.022034	0.066107	0.035558
C	-0.974307	2.529063	0.199191
C	-2.883516	4.626145	0.155608
H	-5.430025	3.689554	-0.202645
H	-6.114918	1.345574	-0.306220
C	-1.502805	4.932439	0.237068
C	-0.534844	3.853299	0.267024
C	-0.985620	6.270982	0.289651
C	0.359490	6.520228	0.374350
H	0.715147	7.546264	0.405837
C	1.300541	5.452034	0.419065
H	2.361870	5.671069	0.491393
C	0.860185	4.157950	0.364914
H	1.565020	3.330711	0.391112
H	-1.666692	7.112632	0.247556
C	-3.940078	5.714133	0.175682
H	-4.705747	5.467748	0.918753
H	-3.516318	6.674802	0.486005
O	-4.645325	5.850188	-1.063715
H	-3.982360	5.891816	-1.766712

Table5_1a_reactant_1wat

Property	Value
Charge	0
Electronic Energy, BS1 (a.u.)	-1056.725729
Thermal and entropic correction, BS1 (a.u.)	2.716658
Electronic Energy, BS2 (a.u.)	-1057.098036
Number of Imaginary Frequencies	0
Imaginary frequencies (cm-1)	None

Molecular Geometry in Cartesian Coordinates

C	8.432847	1.055238	6.907828
H	8.821541	0.609244	7.831874
H	7.673384	1.783479	7.206144
H	7.921538	0.248409	6.367933
C	9.590837	1.096013	4.748829
H	8.865168	0.287466	4.594971
H	9.416932	1.843799	3.970054
H	10.579324	0.662026	4.553196
C	10.798414	1.702464	6.797264
H	11.558049	1.188320	6.194644
H	10.739111	1.106642	7.717137
C	11.408635	3.066904	7.200787
H	12.412079	3.101794	6.757436
H	11.595460	3.013145	8.280968
C	11.416763	5.187497	5.967130
H	10.936450	5.256576	4.987424
H	11.510478	6.207681	6.359810
H	12.439609	4.831490	5.791305
C	10.201926	5.012412	8.087301
H	9.111802	4.989202	8.170105
H	10.597727	4.566741	9.008432
H	10.511207	6.065111	8.096834
N	3.186873	15.019034	10.411635
N	0.322424	15.348895	10.859201
C	3.933357	13.833795	10.909714
H	3.358772	12.924087	10.715539
H	4.914289	13.714943	10.432983
H	4.118420	13.863067	11.990800
C	3.995401	15.780667	9.422560
H	3.449723	15.868863	8.479593
H	4.224023	16.803245	9.748371
H	4.960091	15.306124	9.203550
C	2.767556	15.897919	11.542555
H	3.186382	15.539704	12.491714
H	3.183707	16.907305	11.430896
C	1.247374	16.074815	11.777955
H	1.052804	17.154820	11.757511
H	1.062037	15.789612	12.821495
C	-0.557741	16.300759	10.129881
H	-1.624687	16.074848	10.250928
H	-0.426241	17.342031	10.449728
H	-0.336827	16.270054	9.059099
C	-0.483332	14.341557	11.598894
H	-0.222681	13.334606	11.260510
H	-0.313806	14.364553	12.682613
H	-1.563612	14.467248	11.453165
N	2.422298	1.262234	-6.204836
N	2.387980	3.661127	-7.865263
C	3.656517	0.716304	-5.580675
H	3.447989	-0.003887	-4.779642
H	4.310088	0.201113	-6.295592
H	4.245271	1.529295	-5.146379
C	1.207073	0.650069	-5.604961
H	1.438320	-0.058270	-4.799578
H	0.566080	1.429460	-5.183220
H	0.596943	0.100156	-6.332335
C	2.441651	1.070186	-7.684535
H	1.591820	0.457011	-8.010876
H	3.322536	0.492166	-7.992048
C	2.425731	2.343667	-8.565759
H	3.301730	2.275503	-9.223517
H	1.571394	2.237984	-9.246577
C	1.152858	4.412957	-8.213338
H	1.359391	5.420650	-8.595076
H	0.544857	3.913819	-8.978102
H	0.518372	4.520478	-7.329073
C	3.602485	4.461796	-8.173409
H	3.368539	5.458992	-8.566709
H	4.199550	4.597234	-7.267140
H	4.257363	3.985558	-8.913677
C	-6.605365	15.823022	-0.762554
H	-7.190938	15.554384	0.125520
H	-5.557784	15.890651	-0.456853
H	-6.917525	16.837451	-1.040798
C	-7.692650	13.753546	-1.496511
H	-8.082148	13.857494	-0.476130
H	-8.568383	13.696098	-2.154840
H	-7.188481	12.784601	-1.557711
C	-7.185696	15.516923	-3.127789
H	-7.392711	16.579727	-2.948426
H	-8.144809	15.113435	-3.477235
C	-6.219365	15.459511	-4.336699
H	-6.800211	15.057408	-5.176620
H	-6.019688	16.499737	-4.624362
C	-4.890590	13.536172	-5.083842
H	-3.988236	13.527640	-5.707773

H	-4.904440	12.604913	-4.511115
H	-5.741878	13.491352	-5.774634
C	-3.768938	15.589060	-4.354366
H	-3.063632	15.206729	-5.102750
H	-4.040563	16.601760	-4.677790
H	-3.223285	15.695948	-3.412742
N	8.030869	13.630375	-0.052697
C	7.138552	14.177976	0.798431
H	6.817222	15.187662	0.585997
C	6.631816	13.504847	1.927746
H	5.928933	14.024275	2.565722
C	7.062290	12.185826	2.218056
C	7.995563	11.623234	1.311043
H	8.387573	10.623307	1.446194
C	8.441213	12.371524	0.204375
H	9.152772	11.938322	-0.483838
C	6.593878	11.469951	3.360816
C	5.362111	11.395123	5.265042
C	4.410901	11.999127	6.143758
C	3.870191	13.285802	5.891513
H	4.154864	13.861458	5.020226
C	2.953162	13.855614	6.795320
H	2.547073	14.838663	6.603725
C	3.032049	12.005095	8.168056
H	2.684140	11.514460	9.065870
C	3.955250	11.355912	7.322607
H	4.310103	10.372733	7.602764
C	6.688213	9.641739	4.700563
C	7.198603	8.335053	4.966777
C	6.901521	7.636962	6.164646
H	6.292928	8.080652	6.940999
C	8.038518	7.659344	4.045284
H	8.323034	8.105805	3.101478
C	8.518409	6.370000	4.343394
H	9.152753	5.855000	3.636403
C	7.430093	6.348069	6.378055
H	7.206552	5.818161	7.292328
C	3.492306	3.091573	-2.960223
H	4.416242	3.116734	-3.520210
C	3.527030	3.085317	-1.552057
H	4.491888	3.100217	-1.061695
C	1.185941	3.037147	-2.983562
H	0.273962	3.016457	-3.562845
C	1.122454	3.030792	-1.576041
H	0.148556	2.997830	-1.104950
C	2.317224	3.058296	-0.812707
C	2.303431	3.062975	0.614899
C	1.150377	3.068900	2.570250
C	3.419328	3.077480	2.591210
C	4.650348	3.116338	3.312227
C	4.696070	3.056470	4.727487
H	3.794476	2.953023	5.316991
C	5.901121	3.221737	2.653641
H	5.973612	3.267864	1.575027
C	7.090002	3.272168	3.405820
H	8.041360	3.355442	2.900068
C	5.935990	3.113789	5.394245
H	5.968363	3.064718	6.472881
C	-0.093298	3.165178	3.265081
C	-1.331462	3.212349	2.575946
H	-1.382931	3.162674	1.496075
C	-2.535070	3.320214	3.297311
H	-3.477585	3.355002	2.769505
C	-1.417783	3.348337	5.315743
H	-1.470850	3.413214	6.392813
C	-0.164604	3.240234	4.679354
H	0.728186	3.220718	5.291108
C	-4.022392	13.856301	0.726230
H	-3.791346	14.891600	0.520626
C	-5.012409	11.895700	0.034318
H	-5.572668	11.365977	-0.723071
C	-4.641167	11.230971	1.219193
H	-4.936962	10.197082	1.342399
C	-3.918448	11.923403	2.222567
C	-3.611486	13.278577	1.942902
H	-3.076166	13.897518	2.651208
C	-3.536668	11.298413	3.447544
C	-3.544953	9.489412	4.814569
C	-2.574541	11.423319	5.498492
N	-4.435937	5.351398	5.508171
C	-3.851970	8.115034	5.048742
C	-4.490830	7.310000	4.072318
H	-4.796233	7.714383	3.115644
C	-4.758379	5.953697	4.344491
H	-5.256289	5.344914	3.602774
C	-3.522007	7.469321	6.267461
H	-3.044531	8.002092	7.080069
C	-3.825311	6.105231	6.445637

H	-3.576256	5.618738	7.377171
C	-1.824769	12.152438	6.469512
C	-1.450693	11.579162	7.709801
H	-1.743065	10.572839	7.978117
C	-0.662804	12.314011	8.615054
H	-0.374423	11.865899	9.554596
C	-0.603993	14.135558	7.213081
H	-0.274108	15.149399	7.037233
C	-1.382340	13.478480	6.240326
H	-1.629724	14.008934	5.330302
C	3.387118	6.103720	-5.047934
H	4.330706	5.612689	-5.238370
C	1.082853	5.992221	-5.048377
H	0.191582	5.412661	-5.242601
C	0.969546	7.311767	-4.568954
H	-0.021213	7.717516	-4.409066
C	3.371895	7.429906	-4.571935
H	4.318053	7.930194	-4.409055
C	2.136215	8.076537	-4.315299
C	2.069917	9.415654	-3.824368
C	0.843760	11.187286	-3.107549
C	3.108202	11.317822	-3.148347
C	-0.425577	11.792016	-2.859682
C	-1.641908	11.106464	-3.106921
H	-1.656324	10.090438	-3.480746
C	-2.872170	11.753596	-2.883071
H	-3.798273	11.230863	-3.075566
C	-1.821046	13.684361	-2.177013
H	-1.912520	14.697512	-1.811913
C	-0.545350	13.118000	-2.370135
H	0.325542	13.723760	-2.155357
C	4.306195	12.066381	-2.943513
C	4.284189	13.406686	-2.482223
H	3.354676	13.920009	-2.273884
C	5.491603	14.110247	-2.307615
H	5.470818	15.135522	-1.967012
C	5.588604	11.515261	-3.192214
H	5.710591	10.499776	-3.547182
C	6.742784	12.294566	-2.980605
H	7.718198	11.869800	-3.170461
C	11.143624	14.481980	-0.732721
H	10.737678	13.490154	-0.950522
H	12.124892	14.534577	-1.220843
H	11.334763	14.529827	0.346633
C	9.784901	16.412069	-0.076586
H	8.697149	16.388700	0.035170
H	10.220359	16.111862	0.884688
H	10.063606	17.462625	-0.226088
C	10.796865	16.328413	-2.311735
H	10.882876	17.386004	-2.031181
H	11.831959	16.017899	-2.503535
C	10.070638	16.297343	-3.679294
H	9.868955	17.342723	-3.945961
H	10.814292	15.971888	-4.417970
C	7.650580	16.326921	-4.089492
H	6.948224	16.314412	-3.251383
H	7.911630	17.377841	-4.266290
H	7.105054	15.991504	-4.980378
C	8.984720	14.394406	-4.785782
H	8.195869	14.404645	-5.548371
H	9.936620	14.444696	-5.328925
H	8.953776	13.417195	-4.295421
N	-4.402722	1.125119	5.792713
N	-6.298024	3.157802	6.674048
C	-4.260724	0.590184	4.411673
H	-3.302408	0.081981	4.247109
H	-5.041072	-0.134641	4.147547
H	-4.332157	1.402222	3.683741
C	-3.231209	0.753647	6.629524
H	-2.467161	0.196698	6.072755
H	-2.757991	1.655165	7.028742
H	-3.497772	0.131137	7.492831
C	-5.673989	0.650646	6.414169
H	-5.469867	0.033312	7.298390
H	-6.215373	-0.022799	5.737552
C	-6.689704	1.730290	6.862077
H	-7.631996	1.508871	6.344611
H	-6.909916	1.524865	7.917567
C	-7.294808	3.874019	5.833333
H	-7.687281	4.778958	6.313915
H	-6.839266	4.177745	4.886591
H	-8.167659	3.259000	5.580612
C	-6.120703	3.838677	7.983651
H	-6.730872	4.745416	8.080697
H	-6.376579	3.201690	8.839589
H	-5.074499	4.130382	8.111451
N	9.482860	1.722941	6.093628
N	10.630265	4.293319	6.858484

N	10.198810	15.530887	-1.201030
N	8.828801	15.475345	-3.775986
N	-6.750013	14.841440	-1.870492
N	-4.945945	14.701055	-4.161048
N	5.692238	12.091285	4.154261
N	5.831971	10.172825	5.605992
N	7.091997	10.230180	3.553730
N	8.228993	5.722343	5.489584
N	2.542979	13.238203	7.921294
N	2.346354	3.063365	-3.670763
N	3.498355	3.123863	1.242245
N	1.095231	3.058262	1.218981
N	2.278187	3.045382	3.317015
N	-2.587099	3.380387	4.643029
N	7.118640	3.217122	4.753068
N	-4.715946	13.187527	-0.218273
N	-3.863046	9.995490	3.602330
N	-2.916131	10.151774	5.808006
N	-2.845908	12.051743	4.331717
N	-0.234803	13.572204	8.383076
N	2.266455	5.392520	-5.290061
N	0.839929	9.922950	-3.584045
N	1.941500	11.928180	-2.837752
N	3.241429	10.057802	-3.619176
N	6.704588	13.573411	-2.552472
N	-2.966981	13.021707	-2.435383
Pd	1.454895	14.291625	9.391109
Pd	-4.838639	13.938757	-2.170937
Pd	8.862724	3.743785	5.800676
Pd	8.449005	14.537407	-1.899770
Pd	2.361488	3.346865	-5.756760
Pd	-4.428530	3.257248	5.653413
C	2.774258	5.602185	6.111981
C	2.956161	5.944267	4.783425
C	1.831946	6.284673	3.979195
C	0.599010	6.590254	4.634768
C	0.583367	6.589220	6.056640
C	1.576382	5.936721	6.766471
H	2.839041	6.096433	2.082054
H	3.603520	5.207307	6.701161
H	3.924654	5.869760	4.298706
C	1.876594	6.259321	2.560215
C	-0.566463	6.788431	3.851403
H	-0.279493	7.009891	6.572307
H	1.492671	5.805309	7.848233
C	-0.516901	6.641043	2.482428
C	0.717700	6.394563	1.830167
H	-1.498717	7.006654	4.353230
H	0.739653	6.313303	0.749596
H	-1.421896	6.744012	1.889624
C	3.118926	8.924615	4.125251
C	3.175061	8.340699	5.486624
C	2.080452	8.658990	6.142802
C	1.239582	9.515775	5.272602
H	4.126327	7.894274	5.863216
H	1.836407	8.554618	7.216161
C	1.524423	10.440827	2.910949
C	0.077798	10.188333	2.457936
C	1.772541	11.922824	3.242424
H	2.204739	10.149155	2.101738
C	-0.287299	11.103210	1.285792
H	-0.586616	10.380718	3.301518
H	-0.031434	9.138779	2.182247
C	1.386155	12.824839	2.066382
H	1.175069	12.181938	4.130138
H	2.828496	12.057251	3.503004
C	-0.064688	12.580544	1.632477
H	-1.333055	10.926563	0.989131
H	0.328391	10.841884	0.414410
H	1.535668	13.875193	2.331764
H	2.053295	12.615756	1.218752
H	-0.314862	13.220481	0.778665
H	-0.734921	12.868086	2.453897
N	1.902817	9.587987	4.038468
O	0.228705	10.127772	5.568318
O	3.976828	8.924440	3.245489
O	5.287617	6.496064	2.638893
H	5.429039	6.473604	1.684122
H	4.959378	7.400410	2.806544

Table5_1a_TSiii_TSi-ii_h2o_1wat

Property	Value
Charge	0
Electronic Energy, BS1 (a.u.)	-1056.687167

Thermal and entropic correction, BS1 (a.u.)	2.717515
Electronic Energy, BS2 (a.u.)	-1057.055162
Number of Imaginary Frequencies	0
Imaginary frequencies (cm-1)	None

Molecular Geometry in Cartesian Coordinates

C	8.432924	1.055202	6.908719
H	8.821782	0.609383	7.832781
H	7.673419	1.783414	7.207013
H	7.921627	0.248245	6.369002
C	9.590577	1.095846	4.749539
H	8.864922	0.287251	4.595863
H	9.416532	1.843546	3.970716
H	10.579053	0.661880	4.553805
C	10.798442	1.702591	6.797705
H	11.558034	1.188517	6.194972
H	10.739360	1.106792	7.717607
C	11.408573	3.067117	7.201074
H	12.411946	3.102113	6.757569
H	11.595565	3.013424	8.281229
C	11.416286	5.187675	5.967365
H	10.935799	5.256754	4.987741
H	11.510005	6.207853	6.360058
H	12.439121	4.831730	5.791353
C	10.201746	5.012482	8.087695
H	9.111649	4.988995	8.170739
H	10.597853	4.566962	9.008768
H	10.510778	6.065256	8.097097
N	3.186223	15.018785	10.412553
N	0.321698	15.349003	10.859293
C	3.931968	13.833456	10.911519
H	3.357165	12.923888	10.717301
H	4.913157	13.714075	10.435450
H	4.116353	13.863051	11.992711
C	3.995568	15.779655	9.423551
H	3.450325	15.867873	8.480335
H	4.224653	16.802203	9.749137
H	4.960075	15.304496	9.205069
C	2.766693	15.898354	11.542861
H	3.185386	15.540704	12.492291
H	3.182814	16.907693	11.430675
C	1.246465	16.075294	11.777938
H	1.051862	17.155285	11.757071
H	1.060960	15.790460	12.821549
C	-0.558609	16.300553	10.129719
H	-1.625512	16.074380	10.250644
H	-0.427420	17.341901	10.449448
H	-0.337558	16.269762	9.058968
C	-0.483903	14.341676	11.599168
H	-0.223121	13.334704	11.260952
H	-0.314344	14.364878	12.682876
H	-1.564205	14.467204	11.453460
N	2.422213	1.262217	-6.204551
N	2.388142	3.661605	-7.864292
C	3.656448	0.716135	-5.580545
H	3.447936	-0.004219	-4.779651
H	4.309986	0.201065	-6.295581
H	4.245241	1.529021	-5.146105
C	1.207004	0.649828	-5.604869
H	1.438276	-0.058856	-4.799798
H	0.566065	1.429056	-5.182746
H	0.596807	0.100252	-6.332444
C	2.441603	1.070606	-7.684306
H	1.591927	0.457300	-8.010805
H	3.322628	0.492900	-7.992007
C	2.425308	2.344329	-8.565169
H	3.300941	2.276239	-9.223421
H	1.570593	2.238919	-9.245554
C	1.153380	4.414087	-8.212261
H	1.360381	5.421893	-8.593448
H	0.545363	3.915585	-8.977426
H	0.518725	4.521407	-7.328099
C	3.603020	4.461838	-8.172086
H	3.369551	5.459191	-8.565265
H	4.199949	4.596884	-7.265670
H	4.257860	3.985458	-8.912293
C	-6.604977	15.821860	-0.760172
H	-7.189414	15.552805	0.128520
H	-5.557051	15.889764	-0.455733
H	-6.917776	16.836271	-1.037783
C	-7.692159	13.752008	-1.493262
H	-8.080192	13.855372	-0.472264
H	-8.568856	13.694455	-2.150300
H	-7.187668	12.783297	-1.555627
C	-7.188343	15.516103	-3.124701
H	-7.396293	16.578601	-2.944602

H	-8.147404	15.111782	-3.473324
C	-6.223221	15.460315	-4.334655
H	-6.804690	15.058578	-5.174322
H	-6.024496	16.500902	-4.621653
C	-4.894244	13.538226	-5.084626
H	-3.992780	13.530784	-5.709851
H	-4.906598	12.606582	-4.512496
H	-5.746477	13.493208	-5.774239
C	-3.772873	15.591261	-4.355101
H	-3.068290	15.209840	-5.104625
H	-4.045423	16.604038	-4.677494
H	-3.226095	15.697757	-3.414079
N	8.030815	13.630533	-0.052022
C	7.138622	14.178182	0.799205
H	6.817345	15.187893	0.586812
C	6.631938	13.505073	1.928561
H	5.929186	14.024578	2.566610
C	7.062326	12.186009	2.218804
C	7.995504	11.623388	1.311711
H	8.387467	10.623434	1.446799
C	8.441098	12.371655	0.205006
H	9.152542	11.938399	-0.483288
C	6.593918	11.470089	3.361531
C	5.362091	11.395185	5.265718
C	4.410854	11.999152	6.144427
C	3.870003	13.285743	5.892078
H	4.154570	13.861361	5.020736
C	2.952942	13.855548	6.795841
H	2.546778	14.838550	6.604146
C	3.032119	12.005210	8.168811
H	2.684344	11.514665	9.066727
C	3.955321	11.356008	7.323367
H	4.310328	10.372919	7.603654
C	6.688161	9.641782	4.701160
C	7.198431	8.335034	4.967307
C	6.901640	7.637108	6.165347
H	6.293532	8.081031	6.941960
C	8.037903	7.659080	4.045583
H	8.322154	8.105366	3.101614
C	8.517596	6.369643	4.343606
H	9.151547	5.854435	3.636414
C	7.429993	6.348106	6.378657
H	7.206651	5.818328	7.293058
C	3.492077	3.090875	-2.959372
H	4.416036	3.115943	-3.519325
C	3.526751	3.084844	-1.551204
H	4.491588	3.099856	-1.060803
C	1.185730	3.036384	-2.982793
H	0.273769	3.015574	-3.562104
C	1.122190	3.030236	-1.575272
H	0.148268	2.997274	-1.104229
C	2.316922	3.057902	-0.811889
C	2.303076	3.062766	0.615715
C	1.149957	3.069103	2.571035
C	3.418908	3.077602	2.592056
C	4.649949	3.116197	3.313038
C	4.695726	3.056294	4.728286
H	3.794150	2.952882	5.317809
C	5.900711	3.221304	2.654396
H	5.973166	3.267402	1.575782
C	7.089624	3.271626	3.406522
H	8.040960	3.354787	2.900717
C	5.935687	3.113445	5.394994
H	5.968104	3.064340	6.473626
C	-0.093792	3.165349	3.265746
C	-1.331911	3.211929	2.576486
H	-1.383282	3.161900	1.496624
C	-2.535620	3.319579	3.297711
H	-3.478100	3.353841	2.769808
C	-1.418521	3.348691	5.316244
H	-1.471702	3.413852	6.393296
C	-0.165245	3.240853	4.679989
H	0.727507	3.221825	5.291814
C	-4.020404	13.855320	0.725088
H	-3.789822	14.890765	0.519697
C	-5.009535	11.894363	0.032780
H	-5.569518	11.364539	-0.724734
C	-4.637967	11.229574	1.217523
H	-4.933205	10.195500	1.340513
C	-3.915648	11.922175	2.221072
C	-3.609260	13.277534	1.941648
H	-3.074309	13.896595	2.650113
C	-3.533823	11.297192	3.446042
C	-3.542746	9.488424	4.813363
C	-2.572150	11.422283	5.497202
N	-4.436703	5.351253	5.508245
C	-3.850848	8.114371	5.047988
C	-4.490091	7.309429	4.071737

H	-4.795032	7.713676	3.114856
C	-4.758569	5.953389	4.344322
H	-5.256733	5.344668	3.602726
C	-3.521615	7.468876	6.267015
H	-3.044002	8.001594	7.079578
C	-3.825861	6.105057	6.445581
H	-3.577401	5.618732	7.377353
C	-1.823290	12.151752	6.468646
C	-1.450095	11.578838	7.709370
H	-1.742566	10.572564	7.977778
C	-0.663046	12.314065	8.615049
H	-0.375401	11.866264	9.554965
C	-0.603448	14.135245	7.212680
H	-0.273612	15.149091	7.036819
C	-1.380955	13.477826	6.239498
H	-1.627686	14.008026	5.329147
C	3.386946	6.103575	-5.046752
H	4.330534	5.612521	-5.237136
C	1.082669	5.991947	-5.046850
H	0.191411	5.412260	-5.240783
C	0.969355	7.311644	-4.567835
H	-0.021403	7.717409	-4.407993
C	3.371707	7.429907	-4.571157
H	4.317862	7.930298	-4.408573
C	2.136020	8.076547	-4.314550
C	2.069704	9.415814	-3.824018
C	0.843489	11.187671	-3.107840
C	3.107940	11.318103	-3.148247
C	-0.425886	11.792659	-2.860806
C	-1.642180	11.107184	-3.108434
H	-1.656543	10.090996	-3.481816
C	-2.872478	11.754671	-2.885792
H	-3.798549	11.232062	-3.078781
C	-1.821470	13.685594	-2.179997
H	-1.912989	14.698988	-1.815609
C	-0.545741	13.118891	-2.371947
H	0.325115	13.724632	-2.156967
C	4.305926	12.066617	-2.943242
C	4.283907	13.406884	-2.481836
H	3.354388	13.920237	-2.273593
C	5.491335	14.110360	-2.306962
H	5.470561	15.135602	-1.966257
C	5.588344	11.515447	-3.191787
H	5.710343	10.499985	-3.546816
C	6.742533	12.294658	-2.979900
H	7.717956	11.869845	-3.169601
C	11.143622	14.481880	-0.732681
H	10.737623	13.490093	-0.950564
H	12.124800	14.534538	-1.220978
H	11.334965	14.529552	0.346645
C	9.785083	16.411911	-0.075999
H	8.697342	16.388665	0.035897
H	10.220629	16.111476	0.885162
H	10.063912	17.462457	-0.225339
C	10.796578	16.328545	-2.311372
H	10.882490	17.386133	-2.030780
H	11.831687	16.018161	-2.503298
C	10.070224	16.297458	-3.678863
H	9.868421	17.342828	-3.945473
H	10.813837	15.972095	-4.417620
C	7.650165	16.326792	-4.089021
H	6.947865	16.314381	-3.250867
H	7.911137	17.377701	-4.266004
H	7.104601	15.991204	-4.979818
C	8.984458	14.394322	-4.785163
H	8.195616	14.404441	-5.547763
H	9.936360	14.444633	-5.328302
H	8.953580	13.417150	-4.294722
N	-4.403313	1.125083	5.793598
N	-6.299029	3.157810	6.673888
C	-4.260935	0.589734	4.412757
H	-3.302482	0.081676	4.248541
H	-5.041085	-0.135336	4.148719
H	-4.332394	1.401527	3.684550
C	-3.231947	0.754017	6.630794
H	-2.467796	0.196803	6.074432
H	-2.758791	1.655731	7.029657
H	-3.498676	0.131924	7.494350
C	-5.674682	0.650648	6.414878
H	-5.470697	0.033533	7.299283
H	-6.215861	-0.022995	5.738294
C	-6.690584	1.730318	6.862314
H	-7.632777	1.508649	6.344774
H	-6.910917	1.525190	7.917836
C	-7.295660	3.873671	5.832697
H	-7.688332	4.778729	6.312892
H	-6.839866	4.177159	4.885993
H	-8.168382	3.258484	5.579939

C	-6.122112	3.839127	7.983317
H	-6.732392	4.745839	8.079914
H	-6.378163	3.202395	8.839392
H	-5.075967	4.130961	8.111300
N	9.482766	1.722897	6.094294
N	10.630004	4.293421	6.858834
N	10.198734	15.530873	-1.200654
N	8.828462	15.475337	-3.775460
N	-6.750589	14.840508	-1.868183
N	-4.949160	14.702467	-4.160992
N	5.692254	12.091376	4.154971
N	5.831816	10.172800	5.606545
N	7.091973	10.230283	3.554374
N	8.228442	5.722136	5.489946
N	2.542898	13.238232	7.921919
N	2.346160	3.062539	-3.669968
N	3.497973	3.123869	1.243084
N	1.094866	3.058020	1.219765
N	2.277745	3.045888	3.317856
N	-2.587787	3.380120	4.643405
N	7.118326	3.216639	4.753771
N	-4.713726	13.186407	-0.219480
N	-3.860352	9.994320	3.600927
N	-2.913464	10.150638	5.806620
N	-2.843252	12.050586	4.330299
N	-0.235009	13.572254	8.383084
N	2.266280	5.392247	-5.288481
N	0.839708	9.923169	-3.583900
N	1.941213	11.928527	-2.837858
N	3.241193	10.058044	-3.618964
N	6.704326	13.573462	-2.551659
N	-2.967357	13.023036	-2.438844
Pd	1.454468	14.291635	9.391505
Pd	-4.839122	13.938924	-2.171524
Pd	8.862418	3.743647	5.801217
Pd	8.448781	14.537523	-1.899163
Pd	2.361336	3.346719	-5.755873
Pd	-4.429336	3.257135	5.653647
C	2.763446	5.602815	6.133776
C	2.985009	6.145586	4.825043
C	1.821728	6.300777	3.967114
C	0.603363	6.601734	4.617706
C	0.674821	6.760335	6.059075
C	1.594715	5.922666	6.768695
H	2.838347	6.095877	2.084804
H	3.567890	5.104669	6.664260
H	3.933771	5.973143	4.325544
C	1.875903	6.249533	2.562865
C	-0.562241	6.779340	3.853749
H	-0.216777	7.113937	6.569961
H	1.432527	5.692778	7.816056
C	-0.517669	6.635416	2.475152
C	0.707148	6.388472	1.827868
H	-1.494506	7.012061	4.355479
H	0.733812	6.310666	0.747393
H	-1.422807	6.742485	1.887246
C	3.121605	8.899536	4.117397
C	3.147771	8.159716	5.415590
C	1.966914	8.515015	6.116280
C	1.234513	9.499362	5.276010
H	4.103936	8.014861	5.894302
H	1.898459	8.613972	7.189700
C	1.533437	10.442105	2.913794
C	0.086844	10.188492	2.461250
C	1.776062	11.924352	3.244532
H	2.213785	10.145546	2.109448
C	-0.283986	11.102623	1.284029
H	-0.582264	10.380895	3.304931
H	-0.026564	9.138714	2.186095
C	1.388779	12.825546	2.063361
H	1.178735	12.183573	4.127480
H	2.832019	12.064315	3.499887
C	-0.062075	12.580214	1.630059
H	-1.324938	10.920436	0.992589
H	0.331568	10.841315	0.412549
H	1.537646	13.876075	2.328388
H	2.055820	12.616597	1.215597
H	-0.312954	13.219672	0.776100
H	-0.732107	12.867687	2.451660
N	1.916864	9.589717	4.051180
O	0.239949	10.138213	5.567334
O	3.979171	8.926906	3.242118
O	5.289565	6.493466	2.637091
H	5.428563	6.464338	1.682015
H	4.962073	7.398248	2.799034

Table5_1a_TSiii_DG_h2o_1wat

Property	Value
Charge	0
Electronic Energy, BS1 (a.u.)	-1056.732799
Thermal and entropic correction, BS1 (a.u.)	2.728616
Electronic Energy, BS2 (a.u.)	-1057.098456
Number of Imaginary Frequencies	0
Imaginary frequencies (cm-1)	None

Molecular Geometry in Cartesian Coordinates

C	8.432847	1.055238	6.907828
H	8.821541	0.609244	7.831874
H	7.673384	1.783479	7.206144
H	7.921538	0.248409	6.367933
C	9.590837	1.096013	4.748829
H	8.865168	0.287466	4.594971
H	9.416932	1.843799	3.970054
H	10.579324	0.662026	4.553196
C	10.798414	1.702464	6.797264
H	11.558049	1.188320	6.194644
H	10.739111	1.106642	7.717137
C	11.408635	3.066904	7.200787
H	12.412079	3.101794	6.757436
H	11.595460	3.013145	8.280968
C	11.416763	5.187497	5.967130
H	10.936450	5.256576	4.987424
H	11.510478	6.207681	6.359810
H	12.439609	4.831490	5.791305
C	10.201926	5.012412	8.087301
H	9.111802	4.989202	8.170105
H	10.597727	4.566741	9.008432
H	10.511207	6.065111	8.096834
N	3.186873	15.019034	10.411635
N	0.322424	15.348895	10.859201
C	3.933357	13.833795	10.909714
H	3.358772	12.924087	10.715539
H	4.914289	13.714943	10.432983
H	4.118420	13.863067	11.990800
C	3.995401	15.780667	9.422560
H	3.449723	15.868863	8.479593
H	4.224023	16.803245	9.748371
H	4.960091	15.306124	9.203550
C	2.767556	15.897919	11.542555
H	3.186382	15.539704	12.491714
H	3.183707	16.907305	11.430896
C	1.247374	16.074815	11.777955
H	1.052804	17.154820	11.757511
H	1.062037	15.789612	12.821495
C	-0.557741	16.300759	10.129881
H	-1.624687	16.074848	10.250928
H	-0.426241	17.342031	10.449728
H	-0.336827	16.270054	9.059099
C	-0.483332	14.341557	11.598894
H	-0.222681	13.334606	11.260510
H	-0.313806	14.364553	12.682613
H	-1.563612	14.467248	11.453165
N	2.422298	1.262234	-6.204836
N	2.387980	3.661127	-7.865263
C	3.656517	0.716304	-5.580675
H	3.447989	-0.003887	-4.779642
H	4.310088	0.201113	-6.295592
H	4.245271	1.529295	-5.146379
C	1.207073	0.650069	-5.604961
H	1.438320	-0.058270	-4.799578
H	0.566080	1.429460	-5.183220
H	0.596943	0.100156	-6.332335
C	2.441651	1.070186	-7.684535
H	1.591820	0.457011	-8.010876
H	3.322536	0.492166	-7.992048
C	2.425731	2.343667	-8.565759
H	3.301730	2.275503	-9.223517
H	1.571394	2.237984	-9.246577
C	1.152858	4.412957	-8.213338
H	1.359391	5.420650	-8.595076
H	0.544857	3.913819	-8.978102
H	0.518372	4.520478	-7.329073
C	3.602485	4.461796	-8.173409
H	3.368539	5.458992	-8.566709
H	4.199550	4.597234	-7.267140
H	4.257363	3.985558	-8.913677
C	-6.605365	15.823022	-0.762554
H	-7.190938	15.554384	0.125520
H	-5.557784	15.890651	-0.456853

H	-6.917525	16.837451	-1.040798
C	-7.692650	13.753546	-1.496511
H	-8.082148	13.857494	-0.476130
H	-8.568383	13.696098	-2.154840
H	-7.188481	12.784601	-1.557711
C	-7.185696	15.516923	-3.127789
H	-7.392711	16.579727	-2.948426
H	-8.144809	15.113435	-3.477235
C	-6.219365	15.459511	-4.336699
H	-6.800211	15.057408	-5.176620
H	-6.019688	16.499737	-4.624362
C	-4.890590	13.536172	-5.083842
H	-3.988236	13.527640	-5.707773
H	-4.904440	12.604913	-4.511115
H	-5.741878	13.491352	-5.774634
C	-3.768938	15.589060	-4.354366
H	-3.063632	15.206729	-5.102750
H	-4.040563	16.601760	-4.677790
H	-3.223285	15.695948	-3.412742
N	8.030869	13.630375	-0.052697
C	7.138552	14.177976	0.798431
H	6.817222	15.187662	0.585997
C	6.631816	13.504847	1.927746
H	5.928933	14.024275	2.565722
C	7.062290	12.185826	2.218056
C	7.995563	11.623234	1.311043
H	8.387573	10.623307	1.446194
C	8.441213	12.371524	0.204375
H	9.152772	11.938322	-0.483838
C	6.593878	11.469951	3.360816
C	5.362111	11.395123	5.265042
C	4.410901	11.999127	6.143758
C	3.870191	13.285802	5.891513
H	4.154864	13.861458	5.020226
C	2.953162	13.855614	6.795320
H	2.547073	14.838663	6.603725
C	3.032049	12.005095	8.168056
H	2.684140	11.514460	9.065870
C	3.955250	11.355912	7.322607
H	4.310103	10.372733	7.602764
C	6.688213	9.641739	4.700563
C	7.198603	8.335053	4.966777
C	6.901521	7.636962	6.164646
H	6.292928	8.080652	6.940999
C	8.038518	7.659344	4.045284
H	8.323034	8.105805	3.101478
C	8.518409	6.370000	4.343394
H	9.152753	5.855000	3.636403
C	7.430093	6.348069	6.378055
H	7.206552	5.818161	7.292328
C	3.492306	3.091573	-2.960223
H	4.416242	3.116734	-3.520210
C	3.527030	3.085317	-1.552057
H	4.491888	3.100217	-1.061695
C	1.185941	3.037147	-2.983562
H	0.273962	3.016457	-3.562845
C	1.122454	3.030792	-1.576041
H	0.148556	2.997830	-1.104950
C	2.317224	3.058296	-0.812707
C	2.303431	3.062975	0.614899
C	1.150377	3.068900	2.570250
C	3.419328	3.077480	2.591210
C	4.650348	3.116338	3.312227
C	4.696070	3.056470	4.727487
H	3.794476	2.953023	5.316991
C	5.901121	3.221737	2.653641
H	5.973612	3.267864	1.575027
C	7.090002	3.272168	3.405820
H	8.041360	3.355442	2.900068
C	5.935990	3.113789	5.394245
H	5.968363	3.064718	6.472881
C	-0.093298	3.165178	3.265081
C	-1.331462	3.212349	2.575946
H	-1.382931	3.162674	1.496075
C	-2.535070	3.320214	3.297311
H	-3.477585	3.355002	2.769505
C	-1.417783	3.348337	5.315743
H	-1.470850	3.413214	6.392813
C	-0.164604	3.240234	4.679354
H	0.728186	3.220718	5.291108
C	-4.022392	13.856301	0.726230
H	-3.791346	14.891600	0.520626
C	-5.012409	11.895700	0.034318
H	-5.572668	11.365977	-0.723071
C	-4.641167	11.230971	1.219193
H	-4.936962	10.197082	1.342399
C	-3.918448	11.923403	2.222567
C	-3.611486	13.278577	1.942902

H	-3.076166	13.897518	2.651208
C	-3.536668	11.298413	3.447544
C	-3.544953	9.489412	4.814569
C	-2.574541	11.423319	5.498492
N	-4.435937	5.351398	5.508171
C	-3.851970	8.115034	5.048742
C	-4.490830	7.310000	4.072318
H	-4.796233	7.714383	3.115644
C	-4.758379	5.953697	4.344491
H	-5.256289	5.344914	3.602774
C	-3.522007	7.469321	6.267461
H	-3.044531	8.002092	7.080069
C	-3.825311	6.105231	6.445637
H	-3.576256	5.618738	7.377171
C	-1.824769	12.152438	6.469512
C	-1.450693	11.579162	7.709801
H	-1.743065	10.572839	7.978117
C	-0.662804	12.314011	8.615054
H	-0.374423	11.865899	9.554596
C	-0.603993	14.135558	7.213081
H	-0.274108	15.149399	7.037233
C	-1.382340	13.478480	6.240326
H	-1.629724	14.008934	5.330302
C	3.387118	6.103720	-5.047934
H	4.330706	5.612689	-5.238370
C	1.082853	5.992221	-5.048377
H	0.191582	5.412661	-5.242601
C	0.969546	7.311767	-4.568954
H	-0.021213	7.717516	-4.409066
C	3.371895	7.429906	-4.571935
H	4.318053	7.930194	-4.409055
C	2.136215	8.076537	-4.315299
C	2.069917	9.415654	-3.824368
C	0.843760	11.187286	-3.107549
C	3.108202	11.317822	-3.148347
C	-0.425577	11.792016	-2.859682
C	-1.641908	11.106464	-3.106921
H	-1.656324	10.090438	-3.480746
C	-2.872170	11.753596	-2.883071
H	-3.798273	11.230863	-3.075566
C	-1.821046	13.684361	-2.177013
H	-1.912520	14.697512	-1.811913
C	-0.545350	13.118000	-2.370135
H	0.325542	13.723760	-2.155357
C	4.306195	12.066381	-2.943513
C	4.284189	13.406686	-2.482223
H	3.354676	13.920009	-2.273884
C	5.491603	14.110247	-2.307615
H	5.470818	15.135522	-1.967012
C	5.588604	11.515261	-3.192214
H	5.710591	10.499776	-3.547182
C	6.742784	12.294566	-2.980605
H	7.718198	11.869800	-3.170461
C	11.143624	14.481980	-0.732721
H	10.737678	13.490154	-0.950522
H	12.124892	14.534577	-1.220843
H	11.334763	14.529827	0.346633
C	9.784901	16.412069	-0.076586
H	8.697149	16.388700	0.035170
H	10.220359	16.111862	0.884688
H	10.063606	17.462625	-0.226088
C	10.796865	16.328413	-2.311735
H	10.882876	17.386004	-2.031181
H	11.831959	16.017899	-2.503535
C	10.070638	16.297343	-3.679294
H	9.868955	17.342723	-3.945961
H	10.814292	15.971888	-4.417970
C	7.650580	16.326921	-4.089492
H	6.948224	16.314412	-3.251383
H	7.911630	17.377841	-4.266290
H	7.105054	15.991504	-4.980378
C	8.984720	14.394406	-4.785782
H	8.195869	14.404645	-5.548371
H	9.936620	14.444696	-5.328925
H	8.953776	13.417195	-4.295421
N	-4.402722	1.125119	5.792713
N	-6.298024	3.157802	6.674048
C	-4.260724	0.590184	4.411673
H	-3.302408	0.081981	4.247109
H	-5.041072	-0.134641	4.147547
H	-4.332157	1.402222	3.683741
C	-3.231209	0.753647	6.629524
H	-2.467161	0.196698	6.072755
H	-2.757991	1.655165	7.028742
H	-3.497772	0.131137	7.492831
C	-5.673989	0.650646	6.414169
H	-5.469867	0.033312	7.298390
H	-6.215373	-0.022799	5.737552

C	-6.689704	1.730290	6.862077
H	-7.631996	1.508871	6.344611
H	-6.909916	1.524865	7.917567
C	-7.294808	3.874019	5.833333
H	-7.687281	4.778958	6.313915
H	-6.839266	4.177745	4.886591
H	-8.167659	3.259000	5.580612
C	-6.120703	3.838677	7.983651
H	-6.730872	4.745416	8.080697
H	-6.376579	3.201690	8.839589
H	-5.074499	4.130382	8.111451
N	9.482860	1.722941	6.093628
N	10.630265	4.293319	6.858484
N	10.198810	15.530887	-1.201030
N	8.828801	15.475345	-3.775986
N	-6.750013	14.841440	-1.870492
N	-4.945945	14.701055	-4.161048
N	5.692238	12.091285	4.154261
N	5.831971	10.172825	5.605992
N	7.091997	10.230180	3.553730
N	8.228993	5.722343	5.489584
N	2.542979	13.238203	7.921294
N	2.346354	3.063365	-3.670763
N	3.498355	3.123863	1.242245
N	1.095231	3.058262	1.218981
N	2.278187	3.045382	3.317015
N	-2.587099	3.380387	4.643029
N	7.118640	3.217122	4.753068
N	-4.715946	13.187527	-0.218273
N	-3.863046	9.995490	3.602330
N	-2.916131	10.151774	5.808006
N	-2.845908	12.051743	4.331717
N	-0.234803	13.572204	8.383076
N	2.266455	5.392520	-5.290061
N	0.839929	9.922950	-3.584045
N	1.941500	11.928180	-2.837752
N	3.241429	10.057802	-3.619176
N	6.704588	13.573411	-2.552472
N	-2.966981	13.021707	-2.435383
Pd	1.454895	14.291625	9.391109
Pd	-4.838639	13.938757	-2.170937
Pd	8.862724	3.743785	5.800676
Pd	8.449005	14.537407	-1.899770
Pd	2.361488	3.346865	-5.756760
Pd	-4.428530	3.257248	5.653413
C	2.756073	5.602185	6.157444
C	3.010717	6.317070	4.865260
C	1.813761	6.311951	3.961010
C	0.608103	6.608439	4.607490
C	0.756129	6.907466	6.065733
C	1.612753	5.909443	6.775564
H	2.839041	6.096433	2.091147
H	3.539871	5.016359	6.637512
H	3.942839	6.060708	4.353262
C	1.876594	6.241136	2.569308
C	-0.557370	6.770246	3.860496
H	-0.161287	7.200839	6.572307
H	1.383558	5.596176	7.793677
C	-0.516901	6.631950	2.473335
C	0.699515	6.385470	1.830167
H	-1.489624	7.015747	4.362323
H	0.730560	6.313303	0.749596
H	-1.421896	6.744012	1.889624
C	3.118926	8.879152	4.116158
C	3.120505	8.004267	5.359325
C	1.862226	8.386207	6.097339
C	1.221397	9.479404	5.281695
H	4.080864	8.121593	5.926865
H	1.945520	8.663731	7.170698
C	1.533516	10.440827	2.920042
C	0.086891	10.188333	2.467029
C	1.772541	11.922824	3.251517
H	2.213832	10.140062	2.119923
C	-0.287299	11.103210	1.285792
H	-0.586616	10.380718	3.310611
H	-0.031434	9.138779	2.191340
C	1.386155	12.824839	2.066382
H	1.175069	12.181938	4.130138
H	2.828496	12.066344	3.503004
C	-0.064688	12.580544	1.632477
H	-1.323962	10.917470	0.998224
H	0.328391	10.841884	0.414410
H	1.535668	13.875193	2.331764
H	2.053295	12.615756	1.218752
H	-0.314862	13.220481	0.778665
H	-0.734921	12.868086	2.453897
N	1.921002	9.587987	4.065746
O	0.237798	10.136865	5.568318

O	3.976828	8.933533	3.245489
O	5.287617	6.496064	2.638893
H	5.429039	6.464511	1.684122
H	4.959378	7.400410	2.797451

Table5_1a_reactant_2wat

Property	Value
Charge	0
Electronic Energy, BS1 (a.u.)	-1133.163488
Thermal and entropic correction, BS1 (a.u.)	2.746376
Electronic Energy, BS2 (a.u.)	-1133.572562
Number of Imaginary Frequencies	0
Imaginary frequencies (cm-1)	None

Molecular Geometry in Cartesian Coordinates

C	8.468015	1.050094	6.961471
H	8.839052	0.621212	7.900782
H	7.677442	1.756332	7.229820
H	7.999050	0.224877	6.411026
C	9.693428	1.112325	4.840584
H	8.998311	0.280849	4.669162
H	9.519637	1.851732	4.053732
H	10.700972	0.709663	4.677612
C	10.815495	1.765970	6.923777
H	11.600833	1.243415	6.362730
H	10.735304	1.200052	7.860735
C	11.395173	3.151720	7.298434
H	12.412901	3.181763	6.888648
H	11.545603	3.140715	8.385514
C	11.407483	5.197163	5.942654
H	10.934516	5.203184	4.956679
H	11.493778	6.239872	6.272977
H	12.432954	4.835507	5.796729
C	10.183569	5.143808	8.064166
H	9.093495	5.125654	8.145319
H	10.576018	4.752718	9.011233
H	10.493047	6.195192	8.014142
N	3.161892	15.056809	10.444895
N	0.287930	15.357240	10.849382
C	3.922630	13.878114	10.937061
H	3.367110	12.961121	10.723076
H	4.911503	13.782555	10.471514
H	4.093383	13.896113	12.020750
C	3.967437	15.842231	9.472378
H	3.432056	15.925507	8.523007
H	4.170619	16.866856	9.808413
H	4.944200	15.388964	9.262340
C	2.716608	15.915617	11.581331
H	3.121708	15.546757	12.532364
H	3.127459	16.929333	11.491397
C	1.191348	16.079512	11.792065
H	0.989670	17.158325	11.778961
H	0.989628	15.782533	12.829277
C	-0.579316	16.311180	10.107507
H	-1.647943	16.082250	10.205551
H	-0.456781	17.351032	10.435431
H	-0.336507	16.286761	9.041217
C	-0.530352	14.342880	11.565800
H	-0.259901	13.338500	11.227420
H	-0.383651	14.359699	12.652952
H	-1.607790	14.466565	11.398403
N	2.445333	1.353811	-6.352182
N	2.379728	3.957373	-7.670721
C	3.692260	0.743474	-5.818885
H	3.500021	-0.083930	-5.124236
H	4.345842	0.341452	-6.603110
H	4.275193	1.495485	-5.279944
C	1.243847	0.649889	-5.830128
H	1.492630	-0.169621	-5.144191
H	0.607855	1.352353	-5.283497
H	0.623280	0.210837	-6.621224
C	2.452573	1.366099	-7.844427
H	1.598680	0.804848	-8.245017
H	3.329251	0.833101	-8.234292
C	2.437656	2.748136	-8.543894
H	3.320949	2.776709	-9.194689
H	1.591575	2.730127	-9.242772
C	1.132541	4.729106	-7.916649
H	1.322078	5.783882	-8.152077
H	0.534711	4.331435	-8.746328
H	0.494308	4.699880	-7.029667
C	3.581047	4.812087	-7.864368

H	3.331004	5.846025	-8.133057
H	4.168761	4.845096	-6.942592
H	4.250448	4.443351	-8.651569
C	-6.556807	15.795018	-0.662947
H	-7.070817	15.503695	0.261652
H	-5.491826	15.884186	-0.432329
H	-6.911653	16.804156	-0.907399
C	-7.643447	13.706563	-1.341740
H	-7.965905	13.793578	-0.296647
H	-8.559896	13.631506	-1.940292
H	-7.121398	12.751259	-1.445336
C	-7.293557	15.495305	-2.985565
H	-7.519129	16.549539	-2.779742
H	-8.261330	15.066558	-3.275701
C	-6.408396	15.478684	-4.256437
H	-7.024536	15.050048	-5.057200
H	-6.275125	16.526734	-4.553948
C	-5.026279	13.644730	-5.124387
H	-4.158546	13.699267	-5.793597
H	-4.960192	12.699101	-4.579006
H	-5.909487	13.574019	-5.771500
C	-3.969609	15.730122	-4.392342
H	-3.292259	15.408617	-5.193354
H	-4.305522	16.739453	-4.660961
H	-3.374508	15.829185	-3.480111
N	8.133626	13.712704	0.058183
C	7.207176	14.232967	0.889972
H	6.890031	15.246963	0.690491
C	6.660927	13.532109	1.982949
H	5.934845	14.036326	2.606934
C	7.087496	12.209389	2.260112
C	8.066013	11.677677	1.384102
H	8.471113	10.683194	1.521249
C	8.545720	12.452883	0.311048
H	9.292163	12.040072	-0.352248
C	6.582977	11.468472	3.370717
C	5.307345	11.373886	5.244308
C	4.374277	11.991797	6.132129
C	3.863502	13.293571	5.893869
H	4.150982	13.867824	5.022572
C	2.980042	13.884662	6.817285
H	2.600287	14.880863	6.639788
C	3.030291	12.023218	8.179320
H	2.686648	11.538515	9.082166
C	3.919288	11.353346	7.313690
H	4.254290	10.360653	7.583765
C	6.640924	9.621406	4.686400
C	7.151551	8.315711	4.959281
C	6.822514	7.608794	6.143925
H	6.189077	8.044280	6.904988
C	8.024215	7.650056	4.061016
H	8.338175	8.105462	3.130894
C	8.498848	6.359233	4.364969
H	9.155680	5.850703	3.674145
C	7.348709	6.320241	6.365130
H	7.100267	5.782389	7.268629
C	3.487687	2.813127	-2.886630
H	4.413640	2.787335	-3.443146
C	3.524781	2.857543	-1.479560
H	4.490069	2.857742	-0.989920
C	1.186871	2.813007	-2.908893
H	0.272999	2.784151	-3.485488
C	1.122674	2.862941	-1.502898
H	0.148339	2.863838	-1.031722
C	2.316742	2.893055	-0.740455
C	2.303034	2.948168	0.685383
C	1.145579	3.038999	2.634732
C	3.414709	2.994908	2.662733
C	4.645973	3.031791	3.384049
C	4.690088	3.023201	4.800421
H	3.786557	2.953023	5.391541
C	5.899543	3.087521	2.724493
H	5.975017	3.085843	1.645133
C	7.088148	3.151897	3.476286
H	8.040817	3.199866	2.968848
C	5.930202	3.089587	5.466499
H	5.960576	3.080109	6.546521
C	-0.101975	3.156773	3.318834
C	-1.334088	3.203577	2.618561
H	-1.377955	3.148069	1.538674
C	-2.543993	3.310380	3.328589
H	-3.482346	3.339455	2.793062
C	-1.444282	3.347246	5.356954
H	-1.508026	3.412464	6.433456
C	-0.185098	3.240151	4.731778
H	0.702483	3.217524	5.350819
C	-3.899814	13.834387	0.650958
H	-3.683845	14.874229	0.451632

C	-4.866698	11.863490	-0.051922
H	-5.421790	11.332444	-0.812454
C	-4.479849	11.195233	1.125941
H	-4.760300	10.156437	1.243382
C	-3.763890	11.890917	2.132138
C	-3.474447	13.251962	1.860598
H	-2.945115	13.872896	2.571756
C	-3.378881	11.264884	3.355727
C	-3.405862	9.461954	4.730534
C	-2.436284	11.395756	5.415089
N	-4.440352	5.369606	5.497909
C	-3.752394	8.100657	4.984644
C	-4.416700	7.300281	4.021912
H	-4.705629	7.697235	3.057060
C	-4.735758	5.960726	4.320930
H	-5.255022	5.357144	3.589562
C	-3.449183	7.467145	6.216309
H	-2.960324	7.998420	7.022986
C	-3.804860	6.120010	6.421239
H	-3.579746	5.644733	7.364789
C	-1.718826	12.132967	6.403909
C	-1.358467	11.559288	7.647967
H	-1.636483	10.546423	7.906203
C	-0.612887	12.307784	8.577280
H	-0.338131	11.862257	9.522080
C	-0.564939	14.139376	7.189573
H	-0.256503	15.162485	7.028806
C	-1.303277	13.470458	6.193793
H	-1.544561	14.001188	5.282401
C	3.373659	5.999502	-4.550692
H	4.318390	5.509072	-4.738398
C	1.069050	5.911022	-4.636606
H	0.180122	5.349894	-4.888192
C	0.950352	7.207037	-4.097442
H	-0.042059	7.617200	-3.960815
C	3.352226	7.297907	-4.003824
H	4.297099	7.782236	-3.794388
C	2.114069	7.948121	-3.768591
C	2.043072	9.281649	-3.262399
C	0.814475	11.121335	-2.753170
C	3.078432	11.208525	-2.658909
C	-0.450878	11.778489	-2.684322
C	-1.661744	11.105627	-2.984912
H	-1.677078	10.061483	-3.269880
C	-2.884088	11.802840	-2.939860
H	-3.804417	11.289005	-3.179292
C	-1.838244	13.761545	-2.317984
H	-1.924953	14.808272	-2.063332
C	-0.569927	13.148396	-2.339175
H	0.297761	13.751905	-2.106621
C	4.277376	11.971987	-2.522775
C	4.257072	13.347592	-2.180674
H	3.329441	13.872539	-1.992487
C	5.462363	14.073224	-2.108829
H	5.440594	15.124517	-1.859105
C	5.557541	11.410659	-2.760784
H	5.680150	10.373021	-3.043416
C	6.709250	12.217024	-2.667251
H	7.682303	11.788858	-2.863864
C	11.184776	14.595336	-0.817724
H	10.790635	13.587904	-0.976547
H	12.137765	14.649540	-1.358547
H	11.430870	14.688523	0.247534
C	9.820321	16.521480	-0.164302
H	8.742015	16.471632	0.013406
H	10.319972	16.273753	0.780644
H	10.059670	17.572009	-0.371460
C	10.713545	16.369880	-2.446575
H	10.758903	17.443919	-2.225480
H	11.755139	16.096793	-2.659048
C	9.945954	16.227429	-3.784409
H	9.705930	17.246957	-4.112409
H	10.674355	15.873124	-4.525030
C	7.515982	16.165858	-4.120012
H	6.845541	16.208014	-3.257371
H	7.743744	17.204376	-4.391497
H	6.946580	15.746755	-4.959048
C	8.881180	14.222070	-4.719569
H	8.076729	14.164863	-5.463454
H	9.820915	14.250265	-5.285196
H	8.877948	13.281775	-4.160509
N	-4.458447	1.148427	5.823959
N	-6.340536	3.210671	6.664994
C	-4.317246	0.599925	4.448156
H	-3.367042	0.073678	4.293637
H	-5.108698	-0.113008	4.184725
H	-4.369704	1.407420	3.713409
C	-3.294552	0.770444	6.668634

H	-2.534424	0.200912	6.119308
H	-2.812827	1.669265	7.063714
H	-3.571636	0.157139	7.535189
C	-5.737344	0.694541	6.445204
H	-5.543970	0.088790	7.339839
H	-6.280543	0.015680	5.775490
C	-6.746338	1.789510	6.870506
H	-7.686482	1.569030	6.348744
H	-6.976504	1.601106	7.927044
C	-7.320843	3.921863	5.801227
H	-7.709250	4.838871	6.261620
H	-6.851272	4.204805	4.854924
H	-8.196993	3.311263	5.549204
C	-6.173290	3.910983	7.965719
H	-6.778543	4.823069	8.041823
H	-6.443422	3.289153	8.828354
H	-5.126704	4.197888	8.101132
N	9.524151	1.743530	6.176698
N	10.617840	4.355224	6.880575
N	10.193566	15.605597	-1.274919
N	8.725765	15.368157	-3.784713
N	-6.755193	14.820564	-1.768451
N	-5.091971	14.780835	-4.166706
N	5.652702	12.074000	4.141677
N	5.753142	10.138504	5.569427
N	7.068504	10.221696	3.553818
N	8.175792	5.702680	5.497340
N	2.573797	13.271439	7.946769
N	2.343938	2.791211	-3.602868
N	3.497865	3.001391	1.313077
N	1.094666	2.997488	1.284806
N	2.270798	3.014400	3.385968
N	-2.607417	3.373043	4.673224
N	7.115657	3.152849	4.824583
N	-4.590926	13.162012	-0.292398
N	-3.706291	9.961648	3.511233
N	-2.766259	10.120058	5.719162
N	-2.699158	12.022291	4.245225
N	-0.210152	13.577423	8.364681
N	2.256361	5.315586	-4.870832
N	0.812749	9.823081	-3.127222
N	1.909684	11.856386	-2.454530
N	3.213570	9.903286	-2.987741
N	6.672318	13.528552	-2.353123
N	-2.980102	13.109119	-2.618865
Pd	1.453822	14.312939	9.398840
Pd	-4.847543	13.970312	-2.210658
Pd	8.854996	3.743222	5.848177
Pd	8.429431	14.554585	-1.837805
Pd	2.360664	3.356289	-5.624462
Pd	-4.458749	3.279116	5.665207
C	2.655660	5.620349	5.897575
C	2.908509	5.906434	4.566607
C	1.842273	6.281570	3.698713
C	0.596236	6.654131	4.284976
C	0.504126	6.687743	5.702877
C	1.440051	6.027007	6.480035
H	2.944867	6.027058	1.859863
H	3.441484	5.221227	6.538194
H	3.901971	5.778387	4.136379
C	1.966063	6.237615	2.284693
C	-0.514155	6.881076	3.434071
H	-0.361073	7.154541	6.161208
H	1.304376	5.930898	7.556261
C	-0.398025	6.716962	2.071334
C	0.861267	6.424093	1.487459
H	-1.470462	7.139370	3.877380
H	-1.266231	6.846834	1.431388
H	0.956116	6.378569	0.409464
C	3.166199	8.878908	3.810215
C	3.212638	8.319797	5.183401
C	2.150091	8.693793	5.852638
C	1.358611	9.604971	4.994373
H	4.147485	7.805845	5.542710
H	1.897615	8.588524	6.916947
C	1.664174	10.526571	2.635751
C	0.242072	10.243237	2.133879
C	1.867135	12.007078	2.997191
H	2.359093	10.274118	1.829138
C	-0.093519	11.144203	0.942690
H	-0.464538	10.408441	2.952655
H	0.173004	9.189205	1.844584
C	1.521349	12.897134	1.799487
H	1.222047	12.255335	3.852430
H	2.905771	12.163768	3.312303
C	0.095517	12.624102	1.304390
H	-1.117538	10.953214	0.603813
H	0.578182	10.876074	0.117715

H	1.638619	13.951095	2.063707
H	2.229924	12.694003	0.983603
H	-0.132865	13.265430	0.446988
H	-0.611225	12.896619	2.099967
N	2.025760	9.655869	3.751611
O	0.394039	10.286871	5.290469
O	3.983872	8.762503	2.889356
O	5.397508	6.326791	2.646400
H	5.673856	6.212304	1.729150
H	5.046924	7.237744	2.694708
O	2.695117	8.687101	0.102851
H	3.127362	8.555470	0.963724
H	3.421249	8.907739	-0.494456

Table5_1a_TSiii_TSi-ii_h2o_2wat

Property	Value
Charge	0
Electronic Energy, BS1 (a.u.)	-1133.121908
Thermal and entropic correction, BS1 (a.u.)	2.742678
Electronic Energy, BS2 (a.u.)	-1133.527326
Number of Imaginary Frequencies	0
Imaginary frequencies (cm-1)	None

Molecular Geometry in Cartesian Coordinates

C	8.467905	1.049836	6.961078
H	8.838663	0.620903	7.900475
H	7.677180	1.755958	7.229261
H	7.999198	0.224631	6.410395
C	9.693920	1.112494	4.840539
H	8.999085	0.280831	4.668885
H	9.519972	1.851991	4.053807
H	10.701622	0.710200	4.677619
C	10.815349	1.765861	6.924210
H	11.600740	1.242861	6.363652
H	10.734734	1.200336	7.861367
C	11.395278	3.151602	7.298513
H	12.412986	3.181395	6.888667
H	11.545798	3.140765	8.385577
C	11.407753	5.196854	5.942364
H	10.934667	5.202814	4.956433
H	11.494228	6.239602	6.272520
H	12.433160	4.835037	5.796416
C	10.183998	5.143984	8.063948
H	9.093925	5.125953	8.145195
H	10.576476	4.753037	9.011062
H	10.493557	6.195332	8.013720
N	3.161809	15.056540	10.445162
N	0.287795	15.357068	10.849464
C	3.922644	13.877816	10.937080
H	3.367122	12.960855	10.722922
H	4.911474	13.782338	10.471429
H	4.093465	13.895662	12.020762
C	3.967322	15.842258	9.472829
H	3.432035	15.925689	8.523424
H	4.170361	16.866847	9.809051
H	4.944177	15.389191	9.262787
C	2.716440	15.915097	11.581765
H	3.121397	15.545942	12.532744
H	3.127379	16.928806	11.492196
C	1.191174	16.079075	11.792386
H	0.989602	17.157911	11.779527
H	0.989303	15.781889	12.829510
C	-0.579187	16.311223	10.107541
H	-1.647884	16.082677	10.205698
H	-0.456268	17.351074	10.435313
H	-0.336456	16.286657	9.041247
C	-0.530744	14.342718	11.565587
H	-0.260184	13.338338	11.227266
H	-0.384398	14.359537	12.652783
H	-1.608119	14.466403	11.397787
N	2.444997	1.353599	-6.351988
N	2.379854	3.957068	-7.670759
C	3.691864	0.743026	-5.818783
H	3.499514	-0.084303	-5.124075
H	4.345294	0.340826	-6.603046
H	4.275021	1.494918	-5.279909
C	1.243427	0.649989	-5.829728
H	1.492098	-0.169562	-5.143796
H	0.607683	1.352637	-5.283049
H	0.622673	0.211062	-6.620739
C	2.452082	1.365745	-7.844230
H	1.597974	0.804707	-8.244660

H	3.328559	0.832465	-8.234152
C	2.437511	2.747728	-8.543819
H	3.320824	2.776026	-9.194601
H	1.591435	2.729842	-9.242705
C	1.132776	4.728989	-7.916731
H	1.322481	5.783687	-8.152374
H	0.534780	4.331297	-8.746283
H	0.494634	4.700053	-7.029679
C	3.581299	4.811549	-7.864561
H	3.331439	5.845466	-8.133501
H	4.168969	4.844704	-6.942761
H	4.250653	4.442500	-8.651649
C	-6.556837	15.794963	-0.662992
H	-7.070969	15.503661	0.261545
H	-5.491853	15.883925	-0.432280
H	-6.911533	16.804153	-0.907465
C	-7.643480	13.706561	-1.341929
H	-7.965979	13.793517	-0.296847
H	-8.559879	13.631467	-1.940557
H	-7.121370	12.751301	-1.445539
C	-7.293474	15.495321	-2.985662
H	-7.519300	16.549482	-2.779737
H	-8.261106	15.066410	-3.276018
C	-6.408117	15.478944	-4.256383
H	-7.024220	15.050498	-5.057271
H	-6.274807	16.527032	-4.553736
C	-5.026007	13.645043	-5.124447
H	-4.158088	13.699435	-5.793429
H	-4.960304	12.699329	-4.579180
H	-5.909043	13.574546	-5.771811
C	-3.969318	15.730373	-4.392179
H	-3.292006	15.408987	-5.193271
H	-4.305241	16.739740	-4.660634
H	-3.374153	15.829270	-3.479949
N	8.133566	13.712877	0.058217
C	7.207143	14.233077	0.890068
H	6.889953	15.247067	0.690618
C	6.660981	13.532171	1.983059
H	5.934938	14.036356	2.607113
C	7.087564	12.209443	2.260137
C	8.066059	11.677788	1.384064
H	8.471208	10.683319	1.521159
C	8.545733	12.453070	0.311045
H	9.292198	12.040324	-0.352260
C	6.583083	11.468505	3.370745
C	5.307484	11.373908	5.244346
C	4.374409	11.991789	6.132187
C	3.863700	13.293602	5.894003
H	4.151250	13.867896	5.022756
C	2.980266	13.884693	6.817458
H	2.600603	14.880947	6.640047
C	3.030334	12.023115	8.179314
H	2.686638	11.538343	9.082098
C	3.919346	11.353276	7.313690
H	4.254279	10.360553	7.583735
C	6.641048	9.621412	4.686401
C	7.151666	8.315711	4.959305
C	6.822648	7.608815	6.143976
H	6.189240	8.044333	6.905046
C	8.024325	7.650031	4.061051
H	8.338321	8.105432	3.130939
C	8.499016	6.359246	4.365068
H	9.155922	5.850742	3.674296
C	7.348865	6.320274	6.365217
H	7.100478	5.782433	7.268739
C	3.487819	2.813288	-2.886588
H	4.413756	2.787540	-3.443136
C	3.524906	2.857712	-1.479513
H	4.490193	2.857962	-0.989869
C	1.186997	2.813032	-2.908838
H	0.273120	2.784085	-3.485428
C	1.122794	2.862994	-1.502853
H	0.148460	2.863794	-1.031679
C	2.316861	2.893161	-0.740407
C	2.303127	2.948275	0.685435
C	1.145604	3.039156	2.634753
C	3.414733	2.995015	2.662809
C	4.645964	3.031955	3.384166
C	4.690024	3.023496	4.800541
H	3.786474	2.953426	5.391663
C	5.899551	3.087583	2.724649
H	5.975071	3.085798	1.645291
C	7.088138	3.151933	3.476476
H	8.040830	3.199774	2.969073
C	5.930122	3.089792	5.466648
H	5.960481	3.080421	6.546672
C	-0.101973	3.156907	3.318812
C	-1.334054	3.203706	2.618490

H	-1.377875	3.148199	1.538602
C	-2.543991	3.310456	3.328465
H	-3.482321	3.339491	2.792898
C	-1.444372	3.347338	5.356873
H	-1.508172	3.412581	6.433371
C	-0.185156	3.240274	4.731753
H	0.702396	3.217644	5.350834
C	-3.899880	13.834356	0.650957
H	-3.683871	14.874181	0.451592
C	-4.866666	11.863436	-0.051998
H	-5.421716	11.332392	-0.812571
C	-4.479827	11.195180	1.125879
H	-4.760261	10.156379	1.243317
C	-3.763936	11.890879	2.132118
C	-3.474578	13.251947	1.860620
H	-2.945244	13.872877	2.571768
C	-3.378900	11.264830	3.355692
C	-3.405771	9.461837	4.730423
C	-2.436239	11.395635	5.415032
N	-4.440514	5.369547	5.497816
C	-3.752328	8.100538	4.984510
C	-4.416730	7.300208	4.021802
H	-4.705704	7.697174	3.056969
C	-4.735904	5.960695	4.320851
H	-5.255283	5.357168	3.589519
C	-3.449110	7.466994	6.216160
H	-2.960213	7.998239	7.022834
C	-3.804898	6.119894	6.421111
H	-3.579811	5.644614	7.364671
C	-1.718782	12.132816	6.403871
C	-1.358365	11.559075	7.647880
H	-1.636357	10.546188	7.906058
C	-0.612781	12.307542	8.577221
H	-0.337992	11.861999	9.522004
C	-0.564917	14.139201	7.189603
H	-0.256493	15.162314	7.028883
C	-1.303274	13.470326	6.193814
H	-1.544598	14.001108	5.282463
C	3.373719	5.999368	-4.550580
H	4.318462	5.508917	-4.738170
C	1.069133	5.911011	-4.636945
H	0.180225	5.349964	-4.888780
C	0.950417	7.206989	-4.097674
H	-0.041990	7.617219	-3.961230
C	3.352274	7.297722	-4.003616
H	4.297153	7.781958	-3.794008
C	2.114113	7.948005	-3.768574
C	2.043090	9.281526	-3.262334
C	0.814482	11.121227	-2.753209
C	3.078418	11.208385	-2.658703
C	-0.450859	11.778395	-2.684369
C	-1.661743	11.105554	-2.984897
H	-1.677094	10.061413	-3.269882
C	-2.884058	11.802819	-2.939888
H	-3.804403	11.289041	-3.179390
C	-1.838172	13.761501	-2.318052
H	-1.924864	14.808224	-2.063381
C	-0.569880	13.148308	-2.339244
H	0.297817	13.751791	-2.106658
C	4.277348	11.971859	-2.522546
C	4.256993	13.347499	-2.180610
H	3.329337	13.872437	-1.992510
C	5.462256	14.073186	-2.108835
H	5.440452	15.124497	-1.859213
C	5.557532	11.410532	-2.760440
H	5.680181	10.372865	-3.042942
C	6.709206	12.216958	-2.667018
H	7.682286	11.788829	-2.863606
C	11.184538	14.595592	-0.817505
H	10.790435	13.588151	-0.976378
H	12.137671	14.649810	-1.358078
H	11.430361	14.688819	0.247809
C	9.820086	16.521909	-0.164605
H	8.741757	16.472193	0.013045
H	10.319624	16.274359	0.780446
H	10.059573	17.572363	-0.371956
C	10.713494	16.369805	-2.446768
H	10.758857	17.443900	-2.225974
H	11.755102	16.096620	-2.659049
C	9.946101	16.226945	-3.784680
H	9.706469	17.246398	-4.113204
H	10.674504	15.872056	-4.525013
C	7.516025	16.166078	-4.119950
H	6.845743	16.208410	-3.257179
H	7.744091	17.204515	-4.391500
H	6.946359	15.747093	-4.958869
C	8.880568	14.221853	-4.719538
H	8.075900	14.164724	-5.463189

H	9.820140	14.249852	-5.285448
H	8.877379	13.281602	-4.160402
N	-4.458371	1.148368	5.823846
N	-6.340564	3.210497	6.664916
C	-4.317318	0.599826	4.448044
H	-3.367135	0.073565	4.293444
H	-5.108797	-0.113110	4.184708
H	-4.369845	1.407308	3.713291
C	-3.294331	0.770492	6.668372
H	-2.534159	0.201145	6.118914
H	-2.812709	1.669358	7.063480
H	-3.571220	0.157055	7.534895
C	-5.737152	0.694416	6.445290
H	-5.543628	0.088799	7.339982
H	-6.280354	0.015412	5.775723
C	-6.746217	1.789318	6.870580
H	-7.686387	1.568710	6.348915
H	-6.976272	1.600969	7.927151
C	-7.320962	3.921526	5.801119
H	-7.709441	4.838533	6.261454
H	-6.851439	4.204445	4.854781
H	-8.197054	3.310818	5.549163
C	-6.173334	3.910955	7.965565
H	-6.778649	4.823007	8.041583
H	-6.443392	3.289210	8.828284
H	-5.126765	4.197947	8.100908
N	9.524233	1.743456	6.176711
N	10.618083	4.355134	6.880454
N	10.193393	15.605777	-1.275003
N	8.725622	15.368077	-3.784764
N	-6.755192	14.820569	-1.768538
N	-5.091688	14.781072	-4.166639
N	5.652824	12.074038	4.141723
N	5.753299	10.138531	5.569439
N	7.068602	10.221719	3.553813
N	8.175966	5.702710	5.497446
N	2.573927	13.271384	7.946865
N	2.344067	2.791273	-3.602810
N	3.497939	3.001472	1.313155
N	1.094741	2.997624	1.284825
N	2.270807	3.014606	3.386016
N	-2.607479	3.373111	4.673097
N	7.115603	3.152946	4.824767
N	-4.590913	13.161969	-0.292436
N	-3.706222	9.961568	3.511141
N	-2.766104	10.119894	5.719043
N	-2.699202	12.022224	4.245216
N	-0.210091	13.577198	8.364671
N	2.256458	5.315535	-4.871010
N	0.812757	9.822976	-3.127272
N	1.909670	11.856275	-2.454477
N	3.213570	9.903128	-2.987475
N	6.672225	13.528519	-2.353046
N	-2.980044	13.109115	-2.618926
Pd	1.453834	14.312793	9.398913
Pd	-4.847433	13.970361	-2.210691
Pd	8.855075	3.743233	5.848290
Pd	8.429293	14.554705	-1.837770
Pd	2.360766	3.356165	-5.624456
Pd	-4.458856	3.279036	5.665018
C	2.645677	5.625673	5.917531
C	2.948370	6.116479	4.606506
C	1.837132	6.296969	3.688774
C	0.601169	6.664605	4.270173
C	0.604105	6.848044	5.708051
C	1.460113	6.007436	6.485131
H	2.944502	6.027324	1.864787
H	3.406541	5.121529	6.503092
H	3.911812	5.888426	4.161254
C	1.965757	6.227997	2.289712
C	-0.514286	6.871629	3.439347
H	-0.290984	7.259984	6.166454
H	1.244622	5.821422	7.531405
C	-0.398327	6.712444	2.066613
C	0.850892	6.419499	1.487595
H	-1.465535	7.144989	3.882755
H	-1.266602	6.847351	1.431764
H	0.950615	6.373942	0.409587
C	3.161285	8.854201	3.805107
C	3.172829	8.135325	5.118394
C	2.020491	8.559371	5.822667
C	1.348841	9.590394	4.999377
H	4.127682	7.955917	5.587491
H	1.942920	8.628909	6.896971
C	1.669205	10.526877	2.640706
C	0.247100	10.238474	2.138879
C	1.867134	12.007403	3.002102
H	2.364104	10.269490	1.839053

C	-0.088539	11.144354	0.947649
H	-0.459507	10.408646	2.957662
H	0.178091	9.189425	1.849608
C	1.521248	12.897403	1.799375
H	1.222088	12.255676	3.852371
H	2.905786	12.169123	3.312160
C	0.095417	12.624279	1.304311
H	-1.112527	10.948279	0.608740
H	0.578229	10.876249	0.117728
H	1.638472	13.951386	2.063526
H	2.229816	12.694268	0.983480
H	-0.133030	13.265560	0.446889
H	-0.611341	12.896765	2.099884
N	2.035870	9.651201	3.766540
O	0.399283	10.292310	5.290496
O	3.983851	8.767632	2.889165
O	5.397230	6.326769	2.645979
H	5.673330	6.212269	1.728665
H	5.051682	7.237726	2.689403
O	2.694494	8.687177	0.102720
H	3.131639	8.550437	0.958614
H	3.420725	8.907590	-0.494544

Table5_1a_TSiii_DG_h2o_2wat

Property	Value
Charge	0
Electronic Energy, BS1 (a.u.)	-1133.166835
Thermal and entropic correction, BS1 (a.u.)	2.750123
Electronic Energy, BS2 (a.u.)	-1133.570195
Number of Imaginary Frequencies	0
Imaginary frequencies (cm-1)	None

Molecular Geometry in Cartesian Coordinates

C	8.468015	1.050094	6.961471
H	8.839052	0.621212	7.900782
H	7.677442	1.756332	7.229820
H	7.999050	0.224877	6.411026
C	9.693428	1.112325	4.840584
H	8.998311	0.280849	4.669162
H	9.519637	1.851732	4.053732
H	10.700972	0.709663	4.677612
C	10.815495	1.765970	6.923777
H	11.600833	1.243415	6.362730
H	10.735304	1.200052	7.860735
C	11.395173	3.151720	7.298434
H	12.412901	3.181763	6.888648
H	11.545603	3.140715	8.385514
C	11.407483	5.197163	5.942654
H	10.934516	5.203184	4.956679
H	11.493778	6.239872	6.272977
H	12.432954	4.835507	5.796729
C	10.183569	5.143808	8.064166
H	9.093495	5.125654	8.145319
H	10.576018	4.752718	9.011233
H	10.493047	6.195192	8.014142
N	3.161892	15.056809	10.444895
N	0.287930	15.357240	10.849382
C	3.922630	13.878114	10.937061
H	3.367110	12.961121	10.723076
H	4.911503	13.782555	10.471514
H	4.093383	13.896113	12.020750
C	3.967437	15.842231	9.472378
H	3.432056	15.925507	8.523007
H	4.170619	16.866856	9.808413
H	4.944200	15.388964	9.262340
C	2.716608	15.915617	11.581331
H	3.121708	15.546757	12.532364
H	3.127459	16.929333	11.491397
C	1.191348	16.079512	11.792065
H	0.989670	17.158325	11.778961
H	0.989628	15.782533	12.829277
C	-0.579316	16.311180	10.107507
H	-1.647943	16.082250	10.205551
H	-0.456781	17.351032	10.435431
H	-0.336507	16.286761	9.041217
C	-0.530352	14.342880	11.565800
H	-0.259901	13.338500	11.227420
H	-0.383651	14.359699	12.652952
H	-1.607790	14.466565	11.398403
N	2.445333	1.353811	-6.352182
N	2.379728	3.957373	-7.670721
C	3.692260	0.743474	-5.818885

H	3.500021	-0.083930	-5.124236
H	4.345842	0.341452	-6.603110
H	4.275193	1.495485	-5.279944
C	1.243847	0.649889	-5.830128
H	1.492630	-0.169621	-5.144191
H	0.607855	1.352353	-5.283497
H	0.623280	0.210837	-6.621224
C	2.452573	1.366099	-7.844427
H	1.598680	0.804848	-8.245017
H	3.329251	0.833101	-8.234292
C	2.437656	2.748136	-8.543894
H	3.320949	2.776709	-9.194689
H	1.591575	2.730127	-9.242772
C	1.132541	4.729106	-7.916649
H	1.322078	5.783882	-8.152077
H	0.534711	4.331435	-8.746328
H	0.494308	4.699880	-7.029667
C	3.581047	4.812087	-7.864368
H	3.331004	5.846025	-8.133057
H	4.168761	4.845096	-6.942592
H	4.250448	4.443351	-8.651569
C	-6.556807	15.795018	-0.662947
H	-7.070817	15.503695	0.261652
H	-5.491826	15.884186	-0.432329
H	-6.911653	16.804156	-0.907399
C	-7.643447	13.706563	-1.341740
H	-7.965905	13.793578	-0.296647
H	-8.559896	13.631506	-1.940292
H	-7.121398	12.751259	-1.445336
C	-7.293557	15.495305	-2.985565
H	-7.519129	16.549539	-2.779742
H	-8.261330	15.066558	-3.275701
C	-6.408396	15.478684	-4.256437
H	-7.024536	15.050048	-5.057200
H	-6.275125	16.526734	-4.553948
C	-5.026279	13.644730	-5.124387
H	-4.158546	13.699267	-5.793597
H	-4.960192	12.699101	-4.579006
H	-5.909487	13.574019	-5.771500
C	-3.969609	15.730122	-4.392342
H	-3.292259	15.408617	-5.193354
H	-4.305522	16.739453	-4.660961
H	-3.374508	15.829185	-3.480111
N	8.133626	13.712704	0.058183
C	7.207176	14.232967	0.889972
H	6.890031	15.246963	0.690491
C	6.660927	13.532109	1.982949
H	5.934845	14.036326	2.606934
C	7.087496	12.209389	2.260112
C	8.066013	11.677677	1.384102
H	8.471113	10.683194	1.521249
C	8.545720	12.452883	0.311048
H	9.292163	12.040072	-0.352248
C	6.582977	11.468472	3.370717
C	5.307345	11.373886	5.244308
C	4.374277	11.991797	6.132129
C	3.863502	13.293571	5.893869
H	4.150982	13.867824	5.022572
C	2.980042	13.884662	6.817285
H	2.600287	14.880863	6.639788
C	3.030291	12.023218	8.179320
H	2.686648	11.538515	9.082166
C	3.919288	11.353346	7.313690
H	4.254290	10.360653	7.583765
C	6.640924	9.621406	4.686400
C	7.151551	8.315711	4.959281
C	6.822514	7.608794	6.143925
H	6.189077	8.044280	6.904988
C	8.024215	7.650056	4.061016
H	8.338175	8.105462	3.130894
C	8.498848	6.359233	4.364969
H	9.155680	5.850703	3.674145
C	7.348709	6.320241	6.365130
H	7.100267	5.782389	7.268629
C	3.487687	2.813127	-2.886630
H	4.413640	2.787335	-3.443146
C	3.524781	2.857543	-1.479560
H	4.490069	2.857742	-0.989920
C	1.186871	2.813007	-2.908893
H	0.272999	2.784151	-3.485488
C	1.122674	2.862941	-1.502898
H	0.148339	2.863838	-1.031722
C	2.316742	2.893055	-0.740455
C	2.303034	2.948168	0.685383
C	1.145579	3.038999	2.634732
C	3.414709	2.994908	2.662733
C	4.645973	3.031791	3.384049
C	4.690088	3.023201	4.800421

H	3.786557	2.953023	5.391541
C	5.899543	3.087521	2.724493
H	5.975017	3.085843	1.645133
C	7.088148	3.151897	3.476286
H	8.040817	3.199866	2.968848
C	5.930202	3.089587	5.466499
H	5.960576	3.080109	6.546521
C	-0.101975	3.156773	3.318834
C	-1.334088	3.203577	2.618561
H	-1.377955	3.148069	1.538674
C	-2.543993	3.310380	3.328589
H	-3.482346	3.339455	2.793062
C	-1.444282	3.347246	5.356954
H	-1.508026	3.412464	6.433456
C	-0.185098	3.240151	4.731778
H	0.702483	3.217524	5.350819
C	-3.899814	13.834387	0.650958
H	-3.683845	14.874229	0.451632
C	-4.866698	11.863490	-0.051922
H	-5.421790	11.332444	-0.812454
C	-4.479849	11.195233	1.125941
H	-4.760300	10.156437	1.243382
C	-3.763890	11.890917	2.132138
C	-3.474447	13.251962	1.860598
H	-2.945115	13.872896	2.571756
C	-3.378881	11.264884	3.355727
C	-3.405862	9.461954	4.730534
C	-2.436284	11.395756	5.415089
N	-4.440352	5.369606	5.497909
C	-3.752394	8.100657	4.984644
C	-4.416700	7.300281	4.021912
H	-4.705629	7.697235	3.057060
C	-4.735758	5.960726	4.320930
H	-5.255022	5.357144	3.589562
C	-3.449183	7.467145	6.216309
H	-2.960324	7.998420	7.022986
C	-3.804860	6.120010	6.421239
H	-3.579746	5.644733	7.364789
C	-1.718826	12.132967	6.403909
C	-1.358467	11.559288	7.647967
H	-1.636483	10.546423	7.906203
C	-0.612887	12.307784	8.577280
H	-0.338131	11.862257	9.522080
C	-0.564939	14.139376	7.189573
H	-0.256503	15.162485	7.028806
C	-1.303277	13.470458	6.193793
H	-1.544561	14.001188	5.282401
C	3.373659	5.999502	-4.550692
H	4.318390	5.509072	-4.738398
C	1.069050	5.911022	-4.636606
H	0.180122	5.349894	-4.888192
C	0.950352	7.207037	-4.097442
H	-0.042059	7.617200	-3.960815
C	3.352226	7.297907	-4.003824
H	4.297099	7.782236	-3.794388
C	2.114069	7.948121	-3.768591
C	2.043072	9.281649	-3.262399
C	0.814475	11.121335	-2.753170
C	3.078432	11.208525	-2.658909
C	-0.450878	11.778489	-2.684322
C	-1.661744	11.105627	-2.984912
H	-1.677078	10.061483	-3.269880
C	-2.884088	11.802840	-2.939860
H	-3.804417	11.289005	-3.179292
C	-1.838244	13.761545	-2.317984
H	-1.924953	14.808272	-2.063332
C	-0.569927	13.148396	-2.339175
H	0.297761	13.751905	-2.106621
C	4.277376	11.971987	-2.522775
C	4.257072	13.347592	-2.180674
H	3.329441	13.872539	-1.992487
C	5.462363	14.073224	-2.108829
H	5.440594	15.124517	-1.859105
C	5.557541	11.410659	-2.760784
H	5.680150	10.373021	-3.043416
C	6.709250	12.217024	-2.667251
H	7.682303	11.788858	-2.863864
C	11.184776	14.595336	-0.817724
H	10.790635	13.587904	-0.976547
H	12.137765	14.649540	-1.358547
H	11.430870	14.688523	0.247534
C	9.820321	16.521480	-0.164302
H	8.742015	16.471632	0.013406
H	10.319972	16.273753	0.780644
H	10.059670	17.572009	-0.371460
C	10.713545	16.369880	-2.446575
H	10.758903	17.443919	-2.225480
H	11.755139	16.096793	-2.659048

C	9.945954	16.227429	-3.784409
H	9.705930	17.246957	-4.112409
H	10.674355	15.873124	-4.525030
C	7.515982	16.165858	-4.120012
H	6.845541	16.208014	-3.257371
H	7.743744	17.204376	-4.391497
H	6.946580	15.746755	-4.959048
C	8.881180	14.222070	-4.719569
H	8.076729	14.164863	-5.463454
H	9.820915	14.250265	-5.285196
H	8.877948	13.281775	-4.160509
N	-4.458447	1.148427	5.823959
N	-6.340536	3.210671	6.664994
C	-4.317246	0.599925	4.448156
H	-3.367042	0.073678	4.293637
H	-5.108698	-0.113008	4.184725
H	-4.369704	1.407420	3.713409
C	-3.294552	0.770444	6.668634
H	-2.534424	0.200912	6.119308
H	-2.812827	1.669265	7.063714
H	-3.571636	0.157139	7.535189
C	-5.737344	0.694541	6.445204
H	-5.543970	0.088790	7.339839
H	-6.280543	0.015680	5.775490
C	-6.746338	1.789510	6.870506
H	-7.686482	1.569030	6.348744
H	-6.976504	1.601106	7.927044
C	-7.320843	3.921863	5.801227
H	-7.709250	4.838871	6.261620
H	-6.851272	4.204805	4.854924
H	-8.196993	3.311263	5.549204
C	-6.173290	3.910983	7.965719
H	-6.778543	4.823069	8.041823
H	-6.443422	3.289153	8.828354
H	-5.126704	4.197888	8.101132
N	9.524151	1.743530	6.176698
N	10.617840	4.355224	6.880575
N	10.193566	15.605597	-1.274919
N	8.725765	15.368157	-3.784713
N	-6.755193	14.820564	-1.768451
N	-5.091971	14.780835	-4.166706
N	5.652702	12.074000	4.141677
N	5.753142	10.138504	5.569427
N	7.068504	10.221696	3.553818
N	8.175792	5.702680	5.497340
N	2.573797	13.271439	7.946769
N	2.343938	2.791211	-3.602868
N	3.497865	3.001391	1.313077
N	1.094666	2.997488	1.284806
N	2.270798	3.014400	3.385968
N	-2.607417	3.373043	4.673224
N	7.115657	3.152849	4.824583
N	-4.590926	13.162012	-0.292398
N	-3.706291	9.961648	3.511233
N	-2.766259	10.120058	5.719162
N	-2.699158	12.022291	4.245225
N	-0.210152	13.577423	8.364681
N	2.256361	5.315586	-4.870832
N	0.812749	9.823081	-3.127222
N	1.909684	11.856386	-2.454530
N	3.213570	9.903286	-2.987741
N	6.672318	13.528552	-2.353123
N	-2.980102	13.109119	-2.618865
Pd	1.453822	14.312939	9.398840
Pd	-4.847543	13.970312	-2.210658
Pd	8.854996	3.743222	5.848177
Pd	8.429431	14.554585	-1.837805
Pd	2.360664	3.356289	-5.624462
Pd	-4.458749	3.279116	5.665207
C	2.635957	5.630201	5.936983
C	2.987323	6.320212	4.645421
C	1.832421	6.311125	3.679010
C	0.606088	6.673834	4.255421
C	0.701163	7.003002	5.712729
C	1.479459	5.987599	6.489887
H	2.944867	6.027058	1.869715
H	3.372521	5.024190	6.469231
H	3.921674	5.995127	4.185638
C	1.966063	6.217912	2.294545
C	-0.514155	6.861373	3.443923
H	-0.223147	7.361429	6.171060
H	1.186154	5.714158	7.507002
C	-0.398025	6.707110	2.061482
C	0.841564	6.414241	1.487459
H	-1.460610	7.149222	3.887232
H	-1.266231	6.846834	1.431388
H	0.946264	6.368717	0.409464
C	3.156347	8.829649	3.800363

C	3.133824	7.955279	5.055327
C	1.893943	8.427794	5.793527
C	1.338908	9.575416	5.004225
H	4.108077	8.101401	5.631376
H	1.986281	8.667338	6.877539
C	1.674026	10.526571	2.645603
C	0.251924	10.233385	2.143731
C	1.867135	12.007078	3.007043
H	2.368945	10.264266	1.848841
C	-0.083667	11.144203	0.952542
H	-0.454686	10.408441	2.962507
H	0.182856	9.189205	1.854436
C	1.521349	12.897134	1.799487
H	1.222047	12.255335	3.852430
H	2.905771	12.173620	3.312303
C	0.095517	12.624102	1.304390
H	-1.107686	10.943362	0.613665
H	0.578182	10.876074	0.117715
H	1.638619	13.951095	2.063707
H	2.229924	12.694003	0.983603
H	-0.132865	13.265430	0.446988
H	-0.611225	12.896619	2.099967
N	2.045463	9.646017	3.781166
O	0.403891	10.296723	5.290469
O	3.983872	8.772355	2.889356
O	5.397508	6.326791	2.646400
H	5.673856	6.212304	1.729150
H	5.056776	7.237744	2.684856
O	2.695117	8.687101	0.102851
H	3.137214	8.545618	0.953872
H	3.421249	8.907739	-0.494456

Table5_1b_TSi_reactant_1wat

Property	Value
Charge	0
Electronic Energy, BS1 (a.u.)	-1135.375707
Thermal and entropic correction, BS1 (a.u.)	2.773390
Electronic Energy, BS2 (a.u.)	-1135.769854
Number of Imaginary Frequencies	0
Imaginary frequencies (cm-1)	None

Molecular Geometry in Cartesian Coordinates

C	8.606842	0.994793	6.797838
H	9.009243	0.558913	7.720972
H	7.805998	1.676578	7.096014
H	8.145663	0.168366	6.242508
C	9.781751	1.126380	4.651828
H	9.106715	0.278128	4.482102
H	9.568052	1.873125	3.881587
H	10.795882	0.755158	4.458190
C	10.934359	1.770759	6.720739
H	11.724060	1.295579	6.124809
H	10.894975	1.170460	7.638732
C	11.473788	3.161937	7.132716
H	12.479217	3.244541	6.700391
H	11.651304	3.115830	8.214761
C	11.390255	5.272274	5.885015
H	10.914628	5.306486	4.901244
H	11.430513	6.300173	6.266475
H	12.430654	4.965336	5.720393
C	10.181738	5.057666	8.005165
H	9.093836	4.986940	8.089093
H	10.596738	4.635924	8.929091
H	10.444838	6.122794	8.007858
N	3.173665	14.994801	10.384663
N	0.318192	15.390395	10.829119
C	3.879397	13.793968	10.904024
H	3.275609	12.900428	10.722097
H	4.857462	13.635971	10.432911
H	4.061771	13.834513	11.985170
C	4.011979	15.717832	9.391341
H	3.473886	15.814353	8.444951
H	4.272685	16.735989	9.706849
H	4.961312	15.208989	9.182613
C	2.774148	15.902217	11.500226
H	3.181020	15.548074	12.456092
H	3.216179	16.898833	11.374368
C	1.258301	16.120952	11.728415
H	1.087377	17.204041	11.679711
H	1.066305	15.866975	12.778823
C	-0.562537	16.339993	10.097149
H	-1.629635	16.120289	10.228223

H	-0.424113	17.383329	10.407174
H	-0.350077	16.298988	9.024949
C	-0.486390	14.398701	11.590339
H	-0.233842	13.385980	11.263204
H	-0.305553	14.435185	12.671885
H	-1.567243	14.529135	11.453493
N	2.433615	1.286003	-6.225375
N	2.395608	3.665373	-7.913066
C	3.667471	0.746503	-5.595065
H	3.458381	0.037494	-4.784308
H	4.319537	0.220716	-6.303603
H	4.258069	1.564303	-5.172518
C	1.217960	0.681728	-5.618555
H	1.448419	-0.015338	-4.803162
H	0.576370	1.466817	-5.208449
H	0.608809	0.121786	-6.339032
C	2.453076	1.076873	-7.702762
H	1.602575	0.461007	-8.022201
H	3.333411	0.494436	-8.003408
C	2.438956	2.340210	-8.598497
H	3.317656	2.266303	-9.252038
H	1.587455	2.225284	-9.281368
C	1.156393	4.407145	-8.267949
H	1.357510	5.411313	-8.661615
H	0.549580	3.896144	-9.025814
H	0.522887	4.521835	-7.383866
C	3.605813	4.468471	-8.231838
H	3.366496	5.460827	-8.634107
H	4.204300	4.615578	-7.328288
H	4.261115	3.987946	-8.968958
C	-6.603010	15.831342	-0.814519
H	-7.183414	15.560831	0.076374
H	-5.554158	15.902572	-0.514046
H	-6.919846	16.844743	-1.091243
C	-7.686670	13.758033	-1.543185
H	-8.070527	13.860006	-0.520480
H	-8.566030	13.698330	-2.196469
H	-7.179872	12.790713	-1.608090
C	-7.194101	15.523276	-3.176756
H	-7.405781	16.584894	-2.995804
H	-8.152581	15.115165	-3.522527
C	-6.232467	15.471715	-4.389654
H	-6.815130	15.068619	-5.227840
H	-6.038123	16.513173	-4.676480
C	-4.899276	13.554853	-5.145311
H	-3.998476	13.549722	-5.771511
H	-4.909131	12.622662	-4.574088
H	-5.752296	13.508808	-5.833882
C	-3.782644	15.610990	-4.417237
H	-3.079229	15.232869	-5.169507
H	-4.059588	16.623268	-4.737413
H	-3.233134	15.718072	-3.477855
N	8.005129	13.612967	-0.047362
C	7.118259	14.162279	0.808131
H	6.797895	15.172767	0.598006
C	6.614585	13.488682	1.938545
H	5.915523	14.008619	2.580326
C	7.042682	12.167987	2.224951
C	7.969140	11.603332	1.312087
H	8.357673	10.601595	1.443426
C	8.412337	12.352029	0.204582
H	9.118588	11.917561	-0.488468
C	6.577564	11.452096	3.369164
C	5.344728	11.373219	5.272872
C	4.388148	11.982415	6.142983
C	3.850090	13.268553	5.880363
H	4.143081	13.841949	5.010233
C	2.919255	13.840149	6.768369
H	2.514109	14.821508	6.566199
C	2.983408	11.997223	8.149751
H	2.623647	11.509996	9.044476
C	3.919052	11.345360	7.319039
H	4.270369	10.364478	7.601978
C	6.664145	9.616545	4.701708
C	7.168884	8.302921	4.948032
C	6.863730	7.579598	6.128865
H	6.256387	8.010694	6.910593
C	8.008771	7.642102	4.014934
H	8.300681	8.105920	3.081923
C	8.475022	6.341516	4.281700
H	9.103962	5.836934	3.562498
C	7.382726	6.280834	6.313286
H	7.151271	5.729927	7.213197
C	3.512977	3.136183	-3.005551
H	4.434554	3.176289	-3.568519
C	3.552636	3.102890	-1.598064
H	4.519312	3.111886	-1.111241
C	1.206009	3.075336	-3.021623

H	0.292001	3.065361	-3.597992
C	1.147951	3.040951	-1.614161
H	0.176056	2.998167	-1.139657
C	2.345625	3.055785	-0.855021
C	2.337987	3.028013	0.572452
C	1.195404	2.993295	2.534772
C	3.464741	2.999774	2.542212
C	4.702418	3.021131	3.252199
C	4.760988	2.915426	4.663014
H	3.864662	2.786894	5.254915
C	5.947631	3.153893	2.587772
H	6.011670	3.231121	1.510423
C	7.141902	3.195771	3.332631
H	8.088199	3.303037	2.821971
C	6.006380	2.961614	5.320685
H	6.045990	2.877765	6.396858
C	-0.044995	3.103603	3.234750
C	-1.284167	3.167997	2.548727
H	-1.339729	3.111624	1.469406
C	-2.483434	3.308402	3.271432
H	-3.425881	3.358556	2.744693
C	-1.361919	3.332329	5.286967
H	-1.411191	3.412639	6.363333
C	-0.112239	3.189573	4.648779
H	0.782413	3.161588	5.257124
C	-4.016919	13.876858	0.666659
H	-3.779081	14.909022	0.453658
C	-5.005220	11.912698	-0.020134
H	-5.556732	11.377783	-0.780277
C	-4.651427	11.258029	1.175729
H	-4.951968	10.226320	1.305163
C	-3.939938	11.957711	2.182228
C	-3.623240	13.308880	1.893583
H	-3.093178	13.932085	2.602187
C	-3.576723	11.342961	3.418061
C	-3.599518	9.543658	4.798073
C	-2.645237	11.485772	5.481678
N	-4.375113	5.378624	5.465615
C	-3.875478	8.161482	5.026738
C	-4.476402	7.341149	4.038823
H	-4.780480	7.740256	3.079544
C	-4.704138	5.975580	4.301231
H	-5.172506	5.354275	3.550734
C	-3.543036	7.521286	6.248031
H	-3.092545	8.067002	7.067343
C	-3.800510	6.145878	6.415251
H	-3.542364	5.661929	7.345544
C	-1.892603	12.216032	6.449763
C	-1.551632	11.657828	7.705980
H	-1.878906	10.667097	7.990069
C	-0.750814	12.385283	8.605447
H	-0.488197	11.946943	9.556607
C	-0.621160	14.176134	7.169946
H	-0.258318	15.175944	6.979401
C	-1.409825	13.524514	6.201343
H	-1.630069	14.043398	5.277776
C	3.399912	6.137163	-5.118131
H	4.343645	5.644022	-5.302222
C	1.095608	6.027563	-5.124761
H	0.204304	5.447399	-5.316552
C	0.982101	7.350207	-4.654763
H	-0.008758	7.756867	-4.498001
C	3.384559	7.466204	-4.650316
H	4.330446	7.966680	-4.486747
C	2.148692	8.115666	-4.402500
C	2.081589	9.456939	-3.917610
C	0.852495	11.229221	-3.208004
C	3.117432	11.358426	-3.236470
C	-0.418174	11.828527	-2.953579
C	-1.632925	11.139095	-3.198142
H	-1.644868	10.124731	-3.576587
C	-2.864732	11.778928	-2.962140
H	-3.789513	11.252488	-3.150746
C	-1.818096	13.710589	-2.251768
H	-1.911997	14.721189	-1.880176
C	-0.541023	13.151127	-2.455860
H	0.328695	13.758770	-2.241952
C	4.315455	12.101073	-3.010929
C	4.292357	13.438421	-2.541157
H	3.361820	13.954973	-2.345982
C	5.500089	14.133741	-2.337853
H	5.478524	15.155862	-1.987855
C	5.599081	11.545354	-3.242357
H	5.721876	10.531723	-3.602285
C	6.753303	12.316980	-3.004735
H	7.729728	11.888784	-3.181127
C	11.126264	14.465895	-0.663315
H	10.717817	13.477358	-0.890630

H	12.115369	14.514094	-1.135819
H	11.300766	14.509064	0.419046
C	9.766679	16.400137	-0.021667
H	8.677295	16.379497	0.073233
H	10.186448	16.095077	0.945061
H	10.050427	17.450434	-0.163099
C	10.816877	16.321287	-2.239345
H	10.909555	17.375854	-1.949674
H	11.851437	16.001844	-2.418963
C	10.109452	16.307999	-3.617016
H	9.921080	17.357352	-3.877777
H	10.860250	15.981485	-4.347968
C	7.694243	16.364590	-4.053365
H	6.982062	16.347309	-3.223662
H	7.966481	17.415392	-4.213120
H	7.156229	16.045681	-4.954904
C	9.018840	14.428820	-4.758619
H	8.235732	14.452304	-5.526865
H	9.975122	14.480088	-5.293943
H	8.978749	13.446185	-4.279872
N	-4.372257	1.148550	5.770530
N	-6.250572	3.200648	6.645994
C	-4.242705	0.610640	4.389414
H	-3.291627	0.090128	4.221076
H	-5.033188	-0.104470	4.129012
H	-4.307139	1.423077	3.661345
C	-3.200584	0.765252	6.601585
H	-2.444973	0.200608	6.041060
H	-2.716497	1.661861	6.998241
H	-3.469118	0.145255	7.466091
C	-5.645083	0.687597	6.398917
H	-5.443285	0.075346	7.287204
H	-6.192653	0.012827	5.728621
C	-6.652896	1.776948	6.840452
H	-7.596181	1.559809	6.322941
H	-6.875958	1.578047	7.896589
C	-7.240460	3.919138	5.799028
H	-7.629165	4.828382	6.274489
H	-6.780606	4.215875	4.852185
H	-8.116323	3.308377	5.546418
C	-6.072797	3.886700	7.952688
H	-6.677071	4.798011	8.043338
H	-6.335812	3.255887	8.811053
H	-5.025187	4.171417	8.082277
N	9.627007	1.728947	6.003093
N	10.642412	4.350351	6.781247
N	10.195206	15.521991	-1.142924
N	8.861366	15.498748	-3.737393
N	-6.749696	14.849230	-1.921731
N	-4.955344	14.718047	-4.220443
N	5.679999	12.072804	4.165702
N	5.810442	10.146881	5.612216
N	7.072158	10.211600	3.559359
N	8.176736	5.668509	5.410132
N	2.494111	13.228291	7.891651
N	2.364276	3.117751	-3.711627
N	3.535459	3.080101	1.194727
N	1.133517	3.002255	1.183319
N	2.327702	2.952733	3.275400
N	-2.532296	3.384536	4.616393
N	7.183839	3.102903	4.677572
N	-4.701524	13.201457	-0.279458
N	-3.904719	10.041062	3.578834
N	-3.002213	10.220791	5.801031
N	-2.894628	12.102281	4.303941
N	-0.280214	13.624197	8.354000
N	2.279331	5.425391	-5.358968
N	0.850531	9.966902	-3.689041
N	1.949099	11.971471	-2.937135
N	3.252652	10.098196	-3.706420
N	6.714240	13.592097	-2.565840
N	-2.962588	13.043868	-2.506235
Pd	1.426871	14.306460	9.362233
Pd	-4.836440	13.953213	-2.231924
Pd	8.901104	3.716507	5.725268
Pd	8.452383	14.541335	-1.877321
Pd	2.373835	3.375578	-5.801282
Pd	-4.376108	3.282103	5.629010
C	2.683552	5.591455	6.414573
C	2.894603	5.973255	5.084426
C	1.789623	6.208868	4.205361
C	0.543462	6.522510	4.807743
C	0.515249	6.605300	6.233560
C	1.447215	5.904270	7.010995
H	2.867515	5.981071	2.355202
H	0.816704	6.171255	0.943184
H	3.876402	5.856375	4.632028
C	1.888977	6.148674	2.796680

C	-0.593892	6.698416	3.988169
H	-0.383207	6.994505	6.710796
H	-1.380124	6.632392	1.992351
C	-0.498840	6.533845	2.619812
C	0.754021	6.274371	2.019857
H	-1.545854	6.933747	4.450708
C	3.810294	5.016631	7.234120
H	3.953269	5.546575	8.179530
H	4.747427	5.060628	6.670954
H	3.618126	3.969391	7.499743
C	1.188101	5.636690	8.470164
H	1.462191	4.610117	8.736549
H	0.136232	5.778763	8.726352
H	1.778872	6.294951	9.124605
C	3.032425	8.843260	4.314356
C	3.060123	8.238778	5.672821
C	1.917344	8.574102	6.315072
C	1.132928	9.460324	5.425063
H	4.014322	7.930435	6.114379
H	1.712821	8.559658	7.383217
C	1.462019	10.343678	3.047141
C	0.014990	10.105631	2.587798
C	1.730153	11.828476	3.347253
H	2.142746	10.021270	2.251405
C	-0.327498	11.001814	1.388849
H	-0.654182	10.326403	3.421784
H	-0.114322	9.052575	2.332390
C	1.368758	12.711415	2.147789
H	1.131908	12.116380	4.223900
H	2.786786	11.951523	3.608905
C	-0.082507	12.482995	1.707001
H	-1.369491	10.834503	1.094097
H	0.288337	10.712045	0.529854
H	1.536378	13.764509	2.391450
H	2.038613	12.470445	1.311434
H	-0.313732	13.106301	0.835620
H	-0.754671	12.802486	2.514852
N	1.822281	9.513396	4.201571
O	0.124546	10.090342	5.689414
O	3.900740	8.828225	3.445596
O	5.238317	6.406053	2.870667
H	5.376751	6.407545	1.914848
H	4.886973	7.300153	3.053384

Table5_1b_TSi_TSi-ii_h2o_1wat

Property	Value
Charge	0
Electronic Energy, BS1 (a.u.)	-1135.336529
Thermal and entropic correction, BS1 (a.u.)	2.774086
Electronic Energy, BS2 (a.u.)	-1135.726494
Number of Imaginary Frequencies	0
Imaginary frequencies (cm-1)	None

Molecular Geometry in Cartesian Coordinates

C	8.607761	0.993338	6.797272
H	9.010290	0.556504	7.719901
H	7.807832	1.675799	7.096334
H	8.145378	0.167718	6.241746
C	9.781598	1.124811	4.650676
H	9.105870	0.277092	4.481052
H	9.567899	1.872026	3.880895
H	10.795331	0.752956	4.456176
C	10.935988	1.767052	6.719234
H	11.725054	1.291978	6.122378
H	10.896697	1.165807	7.636610
C	11.476519	3.157428	7.132460
H	12.481823	3.239857	6.699812
H	11.654443	3.110015	8.214384
C	11.394070	5.269594	5.887771
H	10.918302	5.305729	4.904130
H	11.435126	6.296860	6.270849
H	12.434235	4.962214	5.722514
C	10.185458	5.052582	8.007617
H	9.097516	4.982324	8.091380
H	10.600194	4.629287	8.930950
H	10.449114	6.117571	8.011846
N	3.173762	14.992575	10.382105
N	0.318565	15.390212	10.826526
C	3.879071	13.791070	10.900493
H	3.274764	12.897951	10.718230
H	4.856883	13.632866	10.428925
H	4.061872	13.830853	11.981596

C	4.012187	15.715936	9.389125
H	3.474057	15.813001	8.442815
H	4.273031	16.733918	9.705081
H	4.961428	15.207033	9.180115
C	2.774849	15.899360	11.498401
H	3.180836	15.543825	12.454126
H	3.218169	16.895550	11.373692
C	1.259178	16.119811	11.726066
H	1.089446	17.203084	11.677263
H	1.066576	15.866092	12.776425
C	-0.561251	16.340703	10.094625
H	-1.628554	16.121906	10.225558
H	-0.421968	17.383861	10.404868
H	-0.348707	16.299706	9.022439
C	-0.486952	14.399070	11.587480
H	-0.235054	13.386178	11.260375
H	-0.306369	14.435356	12.669076
H	-1.567671	14.530315	11.450360
N	2.436683	1.286621	-6.224812
N	2.395656	3.664064	-7.915090
C	3.671612	0.748946	-5.595067
H	3.463936	0.040526	-4.783437
H	4.323530	0.223091	-6.303690
H	4.261796	1.567739	-5.173869
C	1.222136	0.681894	-5.616212
H	1.453948	-0.013766	-4.799997
H	0.579994	1.466865	-5.206746
H	0.613065	0.120259	-6.335434
C	2.454980	1.075851	-7.701975
H	1.604491	0.459249	-8.020027
H	3.335336	0.493478	-8.002681
C	2.439581	2.338187	-8.599095
H	3.318016	2.264168	-9.252984
H	1.587829	2.221943	-9.281430
C	1.155667	4.404507	-8.270033
H	1.355816	5.408272	-8.665203
H	0.548626	3.892053	-9.026735
H	0.522757	4.519979	-7.385624
C	3.605069	4.467710	-8.235483
H	3.364766	5.459585	-8.638357
H	4.204158	4.615966	-7.332529
H	4.260130	3.987002	-8.972700
C	-6.605784	15.834393	-0.821109
H	-7.187420	15.564967	0.069311
H	-5.557254	15.905287	-0.519401
H	-6.921730	16.847725	-1.099091
C	-7.689561	13.760943	-1.549217
H	-8.074332	13.863816	-0.526948
H	-8.568323	13.701171	-2.203296
H	-7.183152	12.793352	-1.612972
C	-7.194484	15.524578	-3.183727
H	-7.405503	16.586536	-3.004018
H	-8.152977	15.116858	-3.529928
C	-6.231883	15.471017	-4.395758
H	-6.813943	15.066896	-5.233867
H	-6.037044	16.512032	-4.683859
C	-4.898181	13.553148	-5.147972
H	-3.996576	13.547101	-5.773001
H	-4.909064	12.621547	-4.575791
H	-5.750334	13.506653	-5.837587
C	-3.782059	15.609896	-4.420817
H	-3.077484	15.230549	-5.171384
H	-4.058499	16.621658	-4.743060
H	-3.234052	15.718537	-3.480744
N	7.994542	13.605234	-0.055443
C	7.110318	14.155811	0.801886
H	6.790522	15.166562	0.592201
C	6.608363	13.482884	1.933431
H	5.911402	14.003814	2.576692
C	7.035625	12.161739	2.219063
C	7.959134	11.595723	1.303995
H	8.346521	10.593435	1.434493
C	8.400663	12.343693	0.195287
H	9.104479	11.908216	-0.499651
C	6.572720	11.446923	3.364836
C	5.342845	11.369787	5.270571
C	4.386646	11.979693	6.140648
C	3.850179	13.266601	5.878357
H	4.144215	13.840104	5.008635
C	2.919531	13.838867	6.766116
H	2.515659	14.820790	6.564135
C	2.980645	11.995126	8.146504
H	2.619645	11.507830	9.040669
C	3.915913	11.342519	7.315955
H	4.267996	10.360576	7.600916
C	6.661901	9.612885	4.699260
C	7.169031	8.300432	4.946993
C	6.866116	7.578209	6.129052

H	6.255487	8.009316	6.910522
C	8.009425	7.639786	4.014211
H	8.299926	8.102856	3.080412
C	8.478232	6.340423	4.282419
H	9.107740	5.836058	3.563562
C	7.387560	6.280601	6.314846
H	7.157783	5.730560	7.215694
C	3.515545	3.139779	-3.007258
H	4.436962	3.180104	-3.570469
C	3.555575	3.106434	-1.599744
H	4.522303	3.115598	-1.113029
C	1.208549	3.078635	-3.022751
H	0.294398	3.068576	-3.598889
C	1.150868	3.044295	-1.615318
H	0.179065	3.001480	-1.140630
C	2.348727	3.059261	-0.856456
C	2.341242	3.031349	0.571013
C	1.198442	2.994589	2.533114
C	3.467775	3.000836	2.540917
C	4.705248	3.022280	3.251320
C	4.763604	2.915553	4.662129
H	3.867204	2.786675	5.253911
C	5.950512	3.156376	2.587258
H	6.014708	3.234591	1.509994
C	7.144643	3.198228	3.332332
H	8.090970	3.306529	2.821927
C	6.008913	2.961572	5.320014
H	6.048381	2.876783	6.396122
C	-0.042021	3.104613	3.232970
C	-1.280952	3.171372	2.546754
H	-1.336367	3.116722	1.467332
C	-2.480230	3.311735	3.269417
H	-3.422484	3.363475	2.742514
C	-1.359149	3.331351	5.285244
H	-1.408444	3.410291	6.361709
C	-0.109521	3.187961	4.647119
H	0.784957	3.157694	5.255602
C	-4.021221	13.879959	0.664357
H	-3.782135	14.911667	0.450542
C	-5.011237	11.916265	-0.021306
H	-5.562775	11.381213	-0.781331
C	-4.658954	11.262303	1.175371
H	-4.960616	10.230993	1.305478
C	-3.947216	11.962065	2.181644
C	-3.628861	13.312633	1.892032
H	-3.098319	13.935766	2.600339
C	-3.584675	11.347647	3.417835
C	-3.606671	9.548062	4.797463
C	-2.652185	11.490020	5.480994
N	-4.372112	5.380844	5.461945
C	-3.879579	8.165129	5.025121
C	-4.477921	7.343925	4.036361
H	-4.782446	7.742866	3.077147
C	-4.702280	5.977607	4.297791
H	-5.168671	5.355529	3.546713
C	-3.546066	7.525047	6.246185
H	-3.096955	8.071413	7.065813
C	-3.799853	6.148832	6.412359
H	-3.540295	5.664740	7.342161
C	-1.896859	12.218909	6.448010
C	-1.555913	11.660328	7.704055
H	-1.884762	10.670198	7.988516
C	-0.753383	12.386562	8.602961
H	-0.491021	11.947877	9.554033
C	-0.621875	14.177110	7.167205
H	-0.257765	15.176434	6.976531
C	-1.412136	13.526598	6.199113
H	-1.632434	14.045909	5.275781
C	3.399343	6.140229	-5.125097
H	4.343360	5.647623	-5.309162
C	1.095125	6.028325	-5.128627
H	0.204184	5.446848	-5.318103
C	0.980862	7.351830	-4.661158
H	-0.010157	7.757883	-4.503839
C	3.383220	7.470205	-4.660079
H	4.328854	7.971855	-4.498667
C	2.147026	8.118936	-4.411918
C	2.079389	9.461005	-3.929302
C	0.850323	11.232790	-3.218485
C	3.115131	11.363578	-3.251032
C	-0.420258	11.830996	-2.960966
C	-1.635036	11.140338	-3.201883
H	-1.647059	10.125835	-3.579960
C	-2.866796	11.779036	-2.962483
H	-3.791606	11.251649	-3.148283
C	-1.820022	13.711822	-2.255478
H	-1.913797	14.722353	-1.883674
C	-0.543004	13.153598	-2.463244

H	0.326744	13.762122	-2.252018
C	4.313264	12.105993	-3.025482
C	4.290510	13.442400	-2.553117
H	3.360087	13.958457	-2.356150
C	5.498476	14.137126	-2.349003
H	5.477207	15.158583	-1.997004
C	5.596691	11.550604	-3.258669
H	5.719256	10.537683	-3.620658
C	6.751070	12.321604	-3.020008
H	7.727333	11.893613	-3.197771
C	11.118614	14.456140	-0.655465
H	10.708773	13.468862	-0.885593
H	12.109538	14.502763	-1.124293
H	11.289270	14.497408	0.427592
C	9.760459	16.392074	-0.016017
H	8.670728	16.372798	0.074958
H	10.176413	16.085021	0.951729
H	10.046095	17.442189	-0.154944
C	10.819626	16.314930	-2.229436
H	10.914926	17.368431	-1.936745
H	11.853675	15.992473	-2.406589
C	10.116479	16.307730	-3.609327
H	9.931556	17.358347	-3.867455
H	10.868774	15.981535	-4.338884
C	7.702600	16.371738	-4.052446
H	6.987639	16.352768	-3.225136
H	7.977759	17.422533	-4.207125
H	7.166808	16.057599	-4.957002
C	9.024600	14.435227	-4.760528
H	8.242991	14.462376	-5.530185
H	9.982026	14.486772	-5.293784
H	8.982100	13.451120	-4.285056
N	-4.370175	1.150913	5.768823
N	-6.248238	3.203702	6.642981
C	-4.238948	0.612509	4.388052
H	-3.287808	0.091644	4.221168
H	-5.029301	-0.102457	4.126855
H	-4.302136	1.424704	3.659592
C	-3.199648	0.767709	6.601522
H	-2.443264	0.203004	6.042103
H	-2.716139	1.664390	6.998724
H	-3.469373	0.147846	7.465750
C	-5.643895	0.690402	6.395712
H	-5.443264	0.077471	7.283794
H	-6.191305	0.016404	5.724512
C	-6.651294	1.780171	6.837179
H	-7.594560	1.563538	6.319426
H	-6.874685	1.581247	7.893243
C	-7.237636	3.922761	5.795930
H	-7.625805	4.832243	6.271359
H	-6.777549	4.219191	4.849116
H	-8.113849	3.312521	5.543270
C	-6.070354	3.889502	7.949796
H	-6.674234	4.801071	8.040485
H	-6.333786	3.258687	8.808033
H	-5.022619	4.173717	8.079577
N	9.628186	1.726883	6.002317
N	10.645753	4.346770	6.782693
N	10.191718	15.515004	-1.137031
N	8.866742	15.502027	-3.736095
N	-6.751702	14.851414	-1.927656
N	-4.955078	14.717308	-4.224377
N	5.677509	12.068902	4.162945
N	5.809204	10.143804	5.610413
N	7.067280	10.206445	3.555144
N	8.181948	5.668393	5.411944
N	2.493003	13.226934	7.888827
N	2.366614	3.121193	-3.713022
N	3.538685	3.082705	1.193476
N	1.136727	3.005837	1.181744
N	2.330614	2.952144	3.273800
N	-2.529313	3.385931	4.614477
N	7.186391	3.104047	4.677181
N	-4.705934	13.204443	-0.281617
N	-3.912537	10.045689	3.578498
N	-3.010793	10.225718	5.800869
N	-2.901846	12.106732	4.303430
N	-0.281134	13.624764	8.351126
N	2.279193	5.426861	-5.363035
N	0.848340	9.970222	-3.698939
N	1.946896	11.976072	-2.950229
N	3.250307	10.103487	-3.721258
N	6.712537	13.595796	-2.578422
N	-2.964611	13.043954	-2.506487
Pd	1.426486	14.305641	9.359532
Pd	-4.838479	13.954401	-2.234988
Pd	8.904120	3.715341	5.725841
Pd	8.449871	14.539308	-1.880617

Pd	2.375359	3.376611	-5.802995
Pd	-4.373498	3.284355	5.626423
C	2.688345	5.587120	6.438998
C	2.910473	6.077707	5.113147
C	1.785731	6.206343	4.209460
C	0.547998	6.515628	4.812954
C	0.580060	6.689363	6.248266
C	1.476414	5.895010	7.029405
H	2.865999	5.983803	2.363341
H	0.810826	6.190198	0.954086
H	3.878478	5.905456	4.649993
C	1.888786	6.142662	2.807551
C	-0.590519	6.694408	4.007811
H	-0.331252	7.045439	6.725075
H	-1.379973	6.650645	2.010796
C	-0.498140	6.541432	2.633310
C	0.747751	6.281577	2.031674
H	-1.537292	6.940367	4.473838
C	3.827619	5.012150	7.238891
H	3.980189	5.552097	8.179389
H	4.758024	5.057766	6.666359
H	3.643056	3.967948	7.516168
C	1.207815	5.639470	8.488298
H	1.484842	4.620066	8.771284
H	0.156707	5.784437	8.742837
H	1.795747	6.309571	9.128898
C	3.023243	8.825738	4.322256
C	3.040427	8.129372	5.643311
C	1.852226	8.478084	6.310016
C	1.117057	9.432186	5.440079
H	3.994020	7.992003	6.131187
H	1.747028	8.575036	7.381246
C	1.448964	10.334700	3.062575
C	-0.001247	10.108772	2.608009
C	1.725837	11.817235	3.363384
H	2.124161	10.005667	2.266553
C	-0.341902	11.007614	1.409624
H	-0.668786	10.335174	3.444099
H	-0.140437	9.056983	2.353128
C	1.368713	12.704487	2.162899
H	1.131757	12.108589	4.238975
H	2.783923	11.934894	3.619830
C	-0.084800	12.486985	1.723877
H	-1.385651	10.845566	1.116439
H	0.269864	10.712379	0.546659
H	1.544113	13.756089	2.407640
H	2.035965	12.460024	1.325515
H	-0.312115	13.111821	0.852599
H	-0.753748	12.812022	2.532204
N	1.812749	9.501360	4.220390
O	0.110895	10.064497	5.705657
O	3.889899	8.831537	3.454714
O	5.234635	6.410354	2.877738
H	5.372359	6.411896	1.921806
H	4.882917	7.302648	3.059971

Table5_1b_TSi_DG_h2o_1wat

Property	Value
Charge	0
Electronic Energy, BS1 (a.u.)	-1135.383728
Thermal and entropic correction, BS1 (a.u.)	2.780842
Electronic Energy, BS2 (a.u.)	-1135.771180
Number of Imaginary Frequencies	0
Imaginary frequencies (cm-1)	None

Molecular Geometry in Cartesian Coordinates

C	8.664045	0.997215	6.820429
H	9.073001	0.590374	7.753751
H	7.834983	1.652639	7.100299
H	8.239792	0.146260	6.272786
C	9.861397	1.137624	4.687828
H	9.229444	0.254870	4.528581
H	9.620275	1.858692	3.901413
H	10.894009	0.811714	4.511316
C	10.958388	1.867395	6.759097
H	11.774054	1.410006	6.184689
H	10.929410	1.286909	7.690145
C	11.437724	3.287557	7.146150
H	12.446097	3.398639	6.727302
H	11.599759	3.273616	8.231540
C	11.290454	5.356584	5.837027
H	10.823625	5.344033	4.848537

H	11.290134	6.395812	6.188733
H	12.342625	5.082394	5.689746
C	10.069981	5.158574	7.952621
H	8.985512	5.045302	8.033219
H	10.495454	4.781871	8.891180
H	10.289464	6.233196	7.924588
N	3.147255	15.055514	10.482207
N	0.274247	15.398729	10.860336
C	3.878998	13.869566	10.999943
H	3.305892	12.961024	10.795900
H	4.869744	13.746311	10.545068
H	4.040823	13.901642	12.084682
C	3.980171	15.812499	9.509984
H	3.458139	15.895448	8.553324
H	4.199833	16.837027	9.835923
H	4.950289	15.337459	9.317683
C	2.704437	15.938186	11.601267
H	3.093746	15.576816	12.561716
H	3.132447	16.943784	11.501182
C	1.180017	16.129160	11.794379
H	0.993903	17.210183	11.756649
H	0.965456	15.857235	12.835881
C	-0.586801	16.347566	10.104457
H	-1.656709	16.124611	10.202495
H	-0.460798	17.390691	10.420447
H	-0.341656	16.309437	9.039105
C	-0.550553	14.398959	11.589231
H	-0.282507	13.388882	11.266932
H	-0.407237	14.431520	12.676495
H	-1.626885	14.524319	11.416217
N	2.410478	1.274491	-6.279701
N	2.373562	3.689255	-7.916999
C	3.646240	0.721057	-5.665281
H	3.439592	-0.006444	-4.870405
H	4.296514	0.211840	-6.387447
H	4.237665	1.529045	-5.225355
C	1.196719	0.658128	-5.681230
H	1.429984	-0.056706	-4.882205
H	0.557517	1.434429	-5.251249
H	0.584126	0.114491	-6.411270
C	2.424259	1.096506	-7.761217
H	1.572715	0.487168	-8.090272
H	3.303491	0.520717	-8.077526
C	2.406122	2.378443	-8.630129
H	3.279000	2.315369	-9.292500
H	1.548513	2.280511	-9.307980
C	1.138633	4.446747	-8.253293
H	1.345714	5.458191	-8.624648
H	0.527471	3.956847	-9.021479
H	0.506992	4.545834	-7.366025
C	3.588526	4.490558	-8.221930
H	3.355052	5.491478	-8.606029
H	4.188573	4.617300	-7.316331
H	4.240439	4.019586	-8.968167
C	-6.563263	15.819282	-0.748125
H	-7.123405	15.550136	0.156107
H	-5.507894	15.891131	-0.471534
H	-6.886302	16.832111	-1.019812
C	-7.658218	13.741758	-1.447392
H	-8.016641	13.843637	-0.415396
H	-8.553617	13.679618	-2.078353
H	-7.151530	12.775403	-1.524564
C	-7.210076	15.505252	-3.095227
H	-7.427081	16.564972	-2.909422
H	-8.171993	15.088946	-3.421037
C	-6.273519	15.462038	-4.328006
H	-6.869462	15.052830	-5.153812
H	-6.095540	16.505196	-4.619128
C	-4.931771	13.565118	-5.118603
H	-4.043417	13.575972	-5.762349
H	-4.917565	12.629179	-4.553638
H	-5.797383	13.512144	-5.790722
C	-3.825611	15.628484	-4.395805
H	-3.133194	15.264924	-5.165362
H	-4.118934	16.640974	-4.700250
H	-3.258590	15.731874	-3.466425
N	8.031346	13.624055	-0.002083
C	7.146126	14.178341	0.852407
H	6.832910	15.191006	0.642097
C	6.636397	13.508300	1.982549
H	5.939419	14.032261	2.623225
C	7.054955	12.184793	2.269117
C	7.980740	11.615321	1.358485
H	8.363712	10.611412	1.490194
C	8.430937	12.361270	0.252240
H	9.137357	11.922797	-0.438162
C	6.580619	11.469012	3.409700
C	5.343794	11.387956	5.311362

C	4.408696	12.007316	6.198103
C	3.888905	13.303999	5.950300
H	4.180850	13.878020	5.080286
C	2.978853	13.886180	6.852986
H	2.586438	14.874835	6.661166
C	3.035762	12.036195	8.227802
H	2.686566	11.553934	9.129496
C	3.948856	11.373335	7.380316
H	4.299484	10.390268	7.663854
C	6.631650	9.614032	4.718067
C	7.103182	8.283416	4.938153
C	6.760774	7.534779	6.092721
H	6.142675	7.956479	6.872964
C	7.943672	7.628977	4.001103
H	8.260625	8.109330	3.084700
C	8.378344	6.312567	4.240964
H	9.006201	5.812824	3.517318
C	7.248624	6.220254	6.250747
H	6.985699	5.647666	7.129167
C	3.496347	3.063725	-3.024211
H	4.417234	3.107204	-3.588095
C	3.537888	3.019327	-1.617266
H	4.505666	3.024032	-1.132514
C	1.189514	3.006064	-3.037549
H	0.274809	3.001848	-3.612898
C	1.133090	2.960072	-1.630257
H	0.161932	2.914278	-1.154499
C	2.331800	2.967509	-0.872870
C	2.326844	2.932326	0.554299
C	1.190518	2.921162	2.520864
C	3.459704	2.909189	2.519258
C	4.698925	2.921379	3.225397
C	4.756897	2.811120	4.634591
H	3.860779	2.674933	5.222671
C	5.944739	3.049576	2.562196
H	6.009276	3.126700	1.484903
C	7.137435	3.091789	3.310098
H	8.084109	3.197601	2.800142
C	5.999175	2.860472	5.294585
H	6.033827	2.777981	6.370598
C	-0.044465	3.057379	3.226103
C	-1.287931	3.107215	2.546940
H	-1.350572	3.021367	1.469914
C	-2.481709	3.270255	3.274067
H	-3.427612	3.309440	2.752679
C	-1.346733	3.346361	5.280383
H	-1.389902	3.453186	6.354929
C	-0.101319	3.186938	4.637634
H	0.797331	3.177219	5.240893
C	-3.955473	13.856397	0.680246
H	-3.719737	14.889134	0.467125
C	-4.949744	11.892748	-0.001301
H	-5.507277	11.359091	-0.757924
C	-4.586600	11.236692	1.190757
H	-4.888511	10.205647	1.321691
C	-3.866991	11.934896	2.192607
C	-3.551755	13.286219	1.903060
H	-3.017565	13.908327	2.609400
C	-3.501056	11.320168	3.427690
C	-3.542041	9.525701	4.813857
C	-2.571990	11.462608	5.492498
N	-4.358316	5.369387	5.488841
C	-3.837306	8.148205	5.046998
C	-4.449751	7.332546	4.062007
H	-4.752491	7.732742	3.102712
C	-4.687678	5.968913	4.325804
H	-5.163129	5.350914	3.576889
C	-3.506399	7.505836	6.267392
H	-3.048723	8.047810	7.084937
C	-3.773787	6.132619	6.435797
H	-3.514689	5.647051	7.365178
C	-1.825805	12.194038	6.465429
C	-1.478590	11.631082	7.718294
H	-1.797356	10.635859	7.997089
C	-0.688646	12.363384	8.624215
H	-0.422911	11.925211	9.574857
C	-0.576199	14.164216	7.198362
H	-0.224843	15.169467	7.014812
C	-1.354916	13.508378	6.224410
H	-1.579801	14.029711	5.303378
C	3.389110	6.101186	-5.077216
H	4.330958	5.608945	-5.272777
C	1.084473	5.995727	-5.076118
H	0.191372	5.419720	-5.272379
C	0.975440	7.311552	-4.586541
H	-0.014216	7.717364	-4.420293
C	3.378221	7.423072	-4.588999
H	4.325487	7.919662	-4.421574

C	2.144481	8.071518	-4.328192
C	2.080709	9.407333	-3.827607
C	0.853695	11.183531	-3.123985
C	3.118738	11.308635	-3.148654
C	-0.415598	11.796177	-2.896598
C	-1.632327	11.111985	-3.145467
H	-1.647006	10.090134	-3.503113
C	-2.861849	11.768926	-2.946753
H	-3.788356	11.248133	-3.142732
C	-1.809005	13.706140	-2.261135
H	-1.899369	14.726192	-1.915510
C	-0.534260	13.130408	-2.430012
H	0.337037	13.736085	-2.216940
C	4.315755	12.055568	-2.931626
C	4.290320	13.395140	-2.467711
H	3.359259	13.909624	-2.269421
C	5.496274	14.096307	-2.275122
H	5.472910	15.120485	-1.931251
C	5.600211	11.502958	-3.165899
H	5.724396	10.487768	-3.521052
C	6.752984	12.279998	-2.937580
H	7.730283	11.854295	-3.115502
C	11.141217	14.478562	-0.671294
H	10.733062	13.485661	-0.879860
H	12.123328	14.524289	-1.158447
H	11.331053	14.536178	0.407857
C	9.781914	16.414297	-0.034001
H	8.693936	16.391133	0.074971
H	10.215108	16.121497	0.930583
H	10.060546	17.463864	-0.190462
C	10.802656	16.314597	-2.264332
H	10.893221	17.372988	-1.988277
H	11.836525	15.998455	-2.453382
C	10.077562	16.281810	-3.632458
H	9.878317	17.326972	-3.901826
H	10.821118	15.953033	-4.369762
C	7.657532	16.315829	-4.043027
H	6.954876	16.305759	-3.205092
H	7.920764	17.366016	-4.220917
H	7.111719	15.980394	-4.933802
C	8.987981	14.379665	-4.736297
H	8.197500	14.388352	-5.497180
H	9.938864	14.428865	-5.281342
H	8.958149	13.403500	-4.243921
N	-4.348071	1.139043	5.783178
N	-6.222943	3.186783	6.677027
C	-4.243973	0.602712	4.399539
H	-3.293644	0.087740	4.211070
H	-5.035777	-0.116371	4.154688
H	-4.328191	1.415728	3.674348
C	-3.160233	0.756610	6.591322
H	-2.415959	0.191269	6.016532
H	-2.667967	1.653623	6.976867
H	-3.411664	0.137461	7.461590
C	-5.608174	0.675540	6.434889
H	-5.389622	0.073320	7.326057
H	-6.160566	-0.009297	5.778866
C	-6.618061	1.762062	6.878501
H	-7.563601	1.539286	6.367471
H	-6.833779	1.565650	7.936626
C	-7.221601	3.899307	5.835317
H	-7.611691	4.807357	6.311952
H	-6.769121	4.197211	4.885119
H	-8.096024	3.283878	5.589188
C	-6.038525	3.876767	7.980720
H	-6.644362	4.786894	8.072752
H	-6.294485	3.247689	8.842487
H	-4.990890	4.164021	8.103114
N	9.663961	1.758037	6.025172
N	10.567447	4.434443	6.753173
N	10.199322	15.525098	-1.150734
N	8.833993	15.462349	-3.728143
N	-6.733609	14.835073	-1.849954
N	-4.985320	14.721710	-4.185200
N	5.682935	12.090300	4.206558
N	5.777440	10.143913	5.629036
N	7.063071	10.223147	3.592518
N	8.046895	5.616436	5.345718
N	2.560443	13.274598	7.978690
N	2.346916	3.052555	-3.728961
N	3.526696	2.983273	1.171774
N	1.123780	2.907955	1.169173
N	2.325079	2.881992	3.256993
N	-2.521844	3.380888	4.616990
N	7.178738	3.003403	4.656094
N	-4.648487	13.181905	-0.260448
N	-3.840990	10.022092	3.593035
N	-2.929075	10.196990	5.811737

N	-2.814481	12.077471	4.311933
N	-0.234089	13.609830	8.380700
N	2.266074	5.395291	-5.324425
N	0.850382	9.916586	-3.592421
N	1.951147	11.922779	-2.849497
N	3.252870	10.047595	-3.617071
N	6.711021	13.557662	-2.505901
N	-2.955202	13.045060	-2.521812
Pd	1.437406	14.329102	9.424394
Pd	-4.825039	13.945350	-2.204468
Pd	8.859975	3.707143	5.700534
Pd	8.451711	14.527879	-1.850777
Pd	2.354136	3.354955	-5.811759
Pd	-4.356834	3.273093	5.645415
C	2.596717	5.777164	6.474461
C	2.942230	6.464525	5.153593
C	1.785418	6.356795	4.178847
C	0.561005	6.749073	4.741300
C	0.702196	7.218343	6.174037
C	1.415526	6.150456	6.994610
H	2.855327	5.870124	2.386962
H	0.782397	5.917406	0.970721
H	3.874733	6.111528	4.713455
C	1.881617	6.084267	2.817086
C	-0.586083	6.790025	3.956789
H	-0.247506	7.533305	6.613151
H	-1.393151	6.500044	1.976443
C	-0.506504	6.464032	2.598655
C	0.724416	6.131639	2.029818
H	-1.526721	7.106029	4.391102
C	3.588221	4.865338	7.145580
H	3.761201	5.161051	8.184295
H	4.551604	4.898992	6.627626
H	3.249720	3.823689	7.177027
C	0.827505	5.701117	8.297044
H	1.365028	4.855223	8.728818
H	-0.219258	5.402375	8.161958
H	0.820826	6.503130	9.044249
C	3.107231	8.831649	4.207831
C	3.021911	7.999113	5.480614
C	1.703483	8.441060	6.144164
C	1.190392	9.566431	5.267306
H	3.892701	8.203272	6.106168
H	1.832433	8.809680	7.163392
C	1.687386	10.494485	2.955728
C	0.319824	10.101256	2.367285
C	1.772815	11.993513	3.277521
H	2.468606	10.242349	2.233154
C	-0.022674	10.968362	1.146802
H	-0.446341	10.236350	3.137042
H	0.333077	9.041429	2.099587
C	1.429359	12.832163	2.037784
H	1.073713	12.219961	4.090454
H	2.783713	12.228967	3.626469
C	0.046894	12.462792	1.485344
H	-1.017863	10.697706	0.776431
H	0.683366	10.749953	0.334440
H	1.476815	13.897023	2.280938
H	2.184091	12.652379	1.260051
H	-0.179859	13.068815	0.600954
H	-0.713470	12.703234	2.241018
N	2.003588	9.667263	4.137986
O	0.219035	10.262987	5.484775
O	3.983491	8.783548	3.356886
O	5.235021	6.279332	2.775419
H	5.362785	6.257248	1.818251
H	4.917602	7.185824	2.941699

Table5_1b_TSi_TSi-ii_h2o_2_1

Property	Value
Charge	0
Electronic Energy, BS1 (a.u.)	-1211.809097
Thermal and entropic correction, BS1 (a.u.)	2.766874
Electronic Energy, BS2 (a.u.)	-1212.239758
Number of Imaginary Frequencies	0
Imaginary frequencies (cm-1)	None

Molecular Geometry in Cartesian Coordinates

C	7.777584	0.663203	5.373465
H	7.882987	-0.174447	6.074838
H	7.032192	1.345960	5.792399
H	7.363309	0.246312	4.446816

C	9.438860	1.450032	3.717008
H	8.754337	0.882849	3.073345
H	9.428411	2.492144	3.380856
H	10.446280	1.055411	3.532238
C	10.153868	0.914384	6.028396
H	10.966976	0.484199	5.428893
H	9.810941	0.089059	6.666286
C	10.794835	1.983267	6.961322
H	11.878271	1.824907	6.873837
H	10.567105	1.691849	7.995051
C	11.558031	4.065365	5.899669
H	11.118952	4.443408	4.971240
H	12.057730	4.907492	6.395164
H	12.345275	3.356417	5.612478
C	10.377182	4.135823	8.045804
H	9.355147	4.499069	8.181635
H	10.602188	3.485468	8.901059
H	11.046199	5.001036	8.134119
N	2.780723	15.360832	10.851879
N	-0.187037	15.339094	10.429023
C	3.720053	14.418484	11.515860
H	3.526704	13.395602	11.181094
H	4.772017	14.640319	11.296610
H	3.628842	14.418471	12.609578
C	3.520737	16.409323	10.103287
H	3.259834	16.360784	9.041819
H	3.278611	17.425085	10.440657
H	4.610304	16.304758	10.180820
C	1.862051	15.978774	11.869684
H	1.946642	15.464823	12.835739
H	2.204282	16.999412	12.086429
C	0.341565	16.120398	11.569189
H	0.137362	17.189960	11.428153
H	-0.193155	15.854966	12.490720
C	-0.633647	16.177266	9.292899
H	-1.662886	15.949334	8.987334
H	-0.604187	17.249132	9.526789
H	0.015849	16.012511	8.427456
C	-1.178228	14.311118	10.823492
H	-0.776818	13.309633	10.640686
H	-1.440553	14.368069	11.887728
H	-2.117214	14.402054	10.263028
N	2.276544	1.111788	-6.030311
N	2.246891	3.321551	-7.935822
C	3.478211	0.647605	-5.287254
H	3.229693	0.018445	-4.423419
H	4.168258	0.060970	-5.906570
H	4.043535	1.507144	-4.915952
C	1.033704	0.550184	-5.437429
H	1.223991	-0.063448	-4.547987
H	0.362006	1.360664	-5.141282
H	0.471157	-0.083652	-6.134327
C	2.378858	0.767066	-7.478446
H	1.573525	0.082476	-7.774198
H	3.297904	0.203443	-7.683947
C	2.345786	1.939195	-8.490253
H	3.240996	1.832181	-9.116139
H	1.514211	1.733121	-9.176182
C	0.995308	3.987735	-8.383662
H	1.176465	4.950008	-8.878737
H	0.413380	3.382806	-9.090100
H	0.346895	4.178296	-7.523499
C	3.440456	4.128001	-8.306728
H	3.181459	5.068410	-8.809141
H	4.013181	4.382052	-7.410654
H	4.127475	3.599282	-8.979276
C	-6.305372	15.561247	-0.161461
H	-6.627281	15.253714	0.841595
H	-5.211407	15.539537	-0.182998
H	-6.614090	16.607208	-0.284225
C	-7.623353	13.534104	-0.689790
H	-7.722076	13.568540	0.402516
H	-8.641689	13.488781	-1.096546
H	-7.126721	12.594282	-0.950795
C	-7.551086	15.417999	-2.296745
H	-7.663696	16.476008	-2.025386
H	-8.579475	15.048089	-2.402548
C	-6.905125	15.374096	-3.712471
H	-7.658191	14.921292	-4.369641
H	-6.871701	16.419424	-4.044969
C	-5.650477	13.673490	-4.974097
H	-4.932179	13.818287	-5.790767
H	-5.448680	12.693035	-4.532653
H	-6.639954	13.611146	-5.443777
C	-4.538036	15.743897	-4.292108
H	-4.032939	15.524077	-5.241095
H	-4.949879	16.756033	-4.388589
H	-3.769054	15.791346	-3.515436

N	8.257803	13.597835	0.062977
C	7.475284	14.194173	0.986153
H	7.105347	15.181527	0.750082
C	7.134421	13.596930	2.215495
H	6.503725	14.148482	2.900026
C	7.616618	12.302109	2.531771
C	8.438720	11.689413	1.553382
H	8.861712	10.705195	1.704523
C	8.724506	12.365166	0.350972
H	9.351058	11.894102	-0.392950
C	7.279389	11.647336	3.754827
C	6.137927	11.618531	5.719139
C	5.222986	12.248173	6.617715
C	4.718137	13.552120	6.381425
H	5.024805	14.131513	5.520214
C	3.808099	14.131441	7.286819
H	3.436095	15.129860	7.106360
C	3.838463	12.264734	8.635076
H	3.487291	11.778883	9.533800
C	4.752974	11.604669	7.789404
H	5.089577	10.612771	8.059320
C	7.386861	9.825438	5.105741
C	7.839839	8.490930	5.339640
C	7.497522	7.772920	6.511389
H	6.914606	8.224203	7.302069
C	8.645907	7.794443	4.404693
H	8.984593	8.260533	3.488211
C	9.029376	6.462786	4.658529
H	9.645841	5.939036	3.941855
C	7.924631	6.440353	6.672807
H	7.655894	5.897047	7.567684
C	3.266942	3.278419	-2.978743
H	4.189580	3.388234	-3.530943
C	3.288503	3.250084	-1.571105
H	4.245015	3.334452	-1.071670
C	0.970926	3.042193	-3.028048
H	0.067490	2.974142	-3.616960
C	0.896887	2.993915	-1.621326
H	-0.075205	2.879337	-1.159121
C	2.078445	3.106737	-0.845102
C	2.054786	3.086458	0.582346
C	0.914781	2.875494	2.534386
C	3.159455	3.211210	2.561365
C	4.368324	3.445274	3.283709
C	4.416292	3.430767	4.700025
H	3.531648	3.236689	5.292346
C	5.592896	3.718272	2.624103
H	5.661122	3.754324	1.544647
C	6.761095	3.946757	3.375129
H	7.693183	4.153381	2.869488
C	5.634798	3.676015	5.364655
H	5.670467	3.668871	6.444703
C	-0.321161	2.741950	3.235769
C	-1.565886	2.673866	2.560465
H	-1.626926	2.681364	1.480121
C	-2.764041	2.612399	3.298959
H	-3.710816	2.575257	2.779823
C	-1.628523	2.638759	5.302198
H	-1.669616	2.618607	6.381115
C	-0.380673	2.703474	4.651265
H	0.516193	2.732270	5.255686
C	-3.407065	13.425303	0.489697
H	-3.069386	14.415583	0.218308
C	-4.639891	11.557605	-0.076844
H	-5.273019	11.062446	-0.799788
C	-4.312549	10.914076	1.132407
H	-4.716468	9.926190	1.313722
C	-3.478406	11.563269	2.077895
C	-3.020835	12.857520	1.720265
H	-2.388581	13.437813	2.379479
C	-3.129117	10.952879	3.320424
C	-3.240548	9.189100	4.745352
C	-2.062085	11.042771	5.320601
N	-4.111998	5.033611	5.338331
C	-3.559304	7.813177	4.962678
C	-4.289316	7.054627	4.011439
H	-4.666181	7.497642	3.098989
C	-4.534658	5.686201	4.237370
H	-5.078933	5.107565	3.505024
C	-3.129213	7.111585	6.117472
H	-2.574634	7.606489	6.902629
C	-3.419406	5.738659	6.256690
H	-3.081441	5.200255	7.130054
C	-1.185590	11.725697	6.218907
C	-0.853246	11.199910	7.493426
H	-1.271862	10.265723	7.844848
C	0.021944	11.908146	8.342190
H	0.269909	11.516208	9.318782

C	0.272337	13.606023	6.800119
H	0.720949	14.556993	6.550524
C	-0.592931	12.971809	5.887419
H	-0.793028	13.465949	4.944970
C	3.242481	6.013718	-5.288006
H	4.182911	5.500502	-5.429763
C	0.941253	5.953308	-5.388922
H	0.045346	5.391118	-5.610138
C	0.837245	7.285054	-4.941658
H	-0.150629	7.713812	-4.831821
C	3.237947	7.348337	-4.835743
H	4.187563	7.830496	-4.641624
C	2.008284	8.026418	-4.641709
C	1.950258	9.367646	-4.155198
C	0.730827	11.181993	-3.544075
C	2.995962	11.234442	-3.392340
C	-0.537428	11.811867	-3.360925
C	-1.755689	11.127705	-3.599523
H	-1.773524	10.100432	-3.940712
C	-2.984328	11.785734	-3.396071
H	-3.910166	11.258960	-3.579664
C	-1.931676	13.731066	-2.761423
H	-2.017310	14.760045	-2.442958
C	-0.656537	13.157899	-2.932721
H	0.213717	13.773851	-2.746272
C	4.193294	11.911570	-3.006776
C	4.175024	13.220279	-2.459701
H	3.250488	13.766152	-2.324020
C	5.381729	13.841778	-2.080777
H	5.370175	14.838004	-1.662101
C	5.471584	11.311628	-3.139946
H	5.591273	10.316189	-3.548311
C	6.624638	12.010226	-2.730637
H	7.597879	11.549063	-2.823997
C	11.188900	14.538618	-1.098398
H	10.808470	13.514415	-1.152623
H	12.100233	14.567734	-1.708321
H	11.507158	14.715140	-0.063212
C	9.825501	16.475352	-0.484966
H	8.761997	16.419002	-0.234652
H	10.389769	16.300016	0.439592
H	10.025514	17.514071	-0.775452
C	10.583347	16.200456	-2.812628
H	10.677344	17.277582	-2.625175
H	11.603979	15.898932	-3.079667
C	9.758097	16.082457	-4.127410
H	9.509968	17.104368	-4.443006
H	10.439480	15.710491	-4.903912
C	7.284681	16.026988	-4.248094
H	6.708717	16.021046	-3.317313
H	7.473926	17.076134	-4.508749
H	6.646208	15.616275	-5.040420
C	8.601610	14.048516	-4.937549
H	7.736645	13.967940	-5.608077
H	9.495685	14.036471	-5.574000
H	8.630104	13.146275	-4.319147
N	-5.072282	0.955541	5.973462
N	-6.434860	3.439288	6.665741
C	-5.142832	0.309956	4.635495
H	-4.356860	-0.439804	4.480057
H	-6.095688	-0.202208	4.452657
H	-5.035531	1.064556	3.851238
C	-3.970112	0.373602	6.784743
H	-3.398616	-0.390970	6.243791
H	-3.269513	1.159848	7.079795
H	-4.318986	-0.101408	7.710200
C	-6.377466	0.843874	6.688464
H	-6.264485	0.276961	7.621439
H	-7.095450	0.255002	6.103310
C	-7.096523	2.162638	7.064385
H	-8.106493	2.096315	6.640001
H	-7.259695	2.128089	8.149203
C	-7.288265	4.205910	5.719074
H	-7.474959	5.235431	6.049303
H	-6.805322	4.257033	4.739274
H	-8.273751	3.750534	5.559354
C	-6.098493	4.257856	7.860891
H	-6.497440	5.278562	7.806116
H	-6.477470	3.830215	8.797599
H	-5.013136	4.333400	7.968062
N	9.057711	1.374325	5.145786
N	10.504607	3.439411	6.738894
N	10.143019	15.493028	-1.555629
N	8.535422	15.252326	-4.077139
N	-6.852640	14.682415	-1.220539
N	-5.560591	14.727310	-3.928157
N	6.472345	12.314287	4.609856
N	6.553255	10.365606	6.024529

N	7.757680	10.395512	3.938556
N	8.674814	5.779080	5.766797
N	3.368954	13.507613	8.399086
N	2.133033	3.175564	-3.700190
N	3.235983	3.261933	1.213624
N	0.852806	2.943011	1.184225
N	2.036668	2.992417	3.281633
N	-2.807390	2.596902	4.647599
N	6.794902	3.924964	4.722311
N	-4.201616	12.791338	-0.395742
N	-3.618296	9.717694	3.559512
N	-2.503973	9.818915	5.693864
N	-2.298028	11.642591	4.132602
N	0.578204	13.089290	8.006515
N	2.118724	5.318946	-5.564177
N	0.722025	9.903699	-3.980218
N	1.830264	11.897212	-3.214852
N	3.123573	9.972993	-3.862446
N	6.587555	13.253171	-2.210213
N	-3.084637	13.066499	-2.984013
Pd	1.665842	14.205916	9.407209
Pd	-4.868219	13.753716	-2.131050
Pd	8.625159	3.687513	5.717998
Pd	8.350118	14.340011	-1.896527
Pd	2.181139	3.233216	-5.806955
Pd	-4.603126	3.010402	5.657020
C	3.063424	6.089819	6.910249
C	3.923351	6.710818	6.023935
C	3.656386	6.790321	4.631476
C	2.403706	6.293966	4.156334
C	1.496463	5.726455	5.089190
C	1.813961	5.582919	6.425316
H	5.535102	7.695575	4.050575
H	2.772604	6.985837	0.835219
H	4.829651	7.169364	6.411152
C	4.574788	7.334085	3.695883
C	2.092371	6.397770	2.774335
H	0.535920	5.383713	4.714307
H	4.997300	7.742902	1.636688
C	3.008045	6.923121	1.890838
C	4.267263	7.379050	2.353425
H	1.113824	6.062219	2.444328
C	3.404629	5.980564	8.374395
H	4.371391	6.437227	8.597972
H	3.442749	4.935151	8.699935
C	0.837252	4.954310	7.386578
H	1.239832	4.039539	7.835249
H	-0.101644	4.701318	6.885925
C	2.362966	9.927487	4.909812
C	1.877465	9.251014	6.150493
C	0.729127	8.630263	5.856587
C	0.413497	8.880862	4.410121
H	2.416486	9.297357	7.081370
H	0.078672	8.051456	6.490445
C	1.523459	10.350760	2.583246
C	0.599763	9.766453	1.508334
C	1.314892	11.866526	2.727232
H	2.554611	10.184009	2.266029
C	0.883583	10.464412	0.167222
H	-0.446705	9.919814	1.787894
H	0.750490	8.688231	1.425284
C	1.597415	12.563870	1.391573
H	0.279509	12.050403	3.044019
H	1.975464	12.259526	3.504543
C	0.718000	11.986894	0.276293
H	0.214700	10.054977	-0.597171
H	1.909669	10.234781	-0.150643
H	1.441993	13.641427	1.491013
H	2.655434	12.410237	1.143173
H	0.963643	12.469365	-0.673834
H	-0.336620	12.218694	0.486753
N	1.411978	9.703282	3.910970
O	-0.538116	8.445203	3.780973
O	3.395761	10.561733	4.772338
O	-1.268126	5.779151	3.298087
H	-1.403828	5.818060	2.342666
H	-1.127900	6.709959	3.542109
O	4.733653	10.866335	2.182512
H	4.340475	10.943502	3.067255
H	4.833824	9.911766	2.074212
H	2.653655	6.465953	9.006222
H	0.603716	5.623401	8.221210

Table5_1b_TSi_TSi-ii_2wat

Property	Value

Charge	0
Electronic Energy, BS1 (a.u.)	-1211.770368
Thermal and entropic correction, BS1 (a.u.)	2.771511
Electronic Energy, BS2 (a.u.)	-1212.196750
Number of Imaginary Frequencies	0
Imaginary frequencies (cm-1)	None

Molecular Geometry in Cartesian Coordinates

C	7.796676	0.638934	5.499107
H	7.863327	-0.158784	6.250026
H	7.028126	1.340436	5.837451
H	7.434541	0.170230	4.575464
C	9.542676	1.343201	3.893672
H	8.897424	0.735787	3.246205
H	9.542382	2.366101	3.502584
H	10.561241	0.949658	3.783040
C	10.136211	0.924183	6.262175
H	10.969754	0.449568	5.727630
H	9.754612	0.142957	6.932760
C	10.749925	2.041992	7.155061
H	11.835457	1.878335	7.113182
H	10.484545	1.811122	8.195158
C	11.537284	4.047586	5.972647
H	11.105075	4.381819	5.023900
H	12.048342	4.907601	6.423964
H	12.314108	3.311825	5.727285
C	10.344317	4.258151	8.102757
H	9.322838	4.628105	8.219414
H	10.571340	3.660445	8.995213
H	11.013058	5.127449	8.138134
N	2.742668	15.377154	10.872090
N	-0.213079	15.303350	10.412403
C	3.696940	14.453735	11.541365
H	3.526478	13.427437	11.204704
H	4.745563	14.697123	11.328944
H	3.598900	14.450811	12.634447
C	3.465877	16.443082	10.131604
H	3.215877	16.390286	9.067538
H	3.198063	17.452660	10.468051
H	4.556758	16.362684	10.219058
C	1.802774	15.972818	11.883960
H	1.895565	15.462312	12.851147
H	2.116509	17.002408	12.101175
C	0.280698	16.072818	11.575640
H	0.043832	17.138410	11.457460
H	-0.252545	15.769127	12.486122
C	-0.663612	16.153510	9.287014
H	-1.684046	15.910126	8.964769
H	-0.659195	17.221078	9.541495
H	-0.000280	16.018902	8.426768
C	-1.183301	14.243369	10.771842
H	-0.751956	13.256306	10.579462
H	-1.465609	14.275102	11.832019
H	-2.114235	14.319196	10.195978
N	2.257441	1.119874	-5.993859
N	2.223375	3.322995	-7.907507
C	3.464976	0.657716	-5.259178
H	3.223365	0.033256	-4.389991
H	4.148540	0.066868	-5.881651
H	4.034916	1.518254	-4.897546
C	1.019352	0.561180	-5.388486
H	1.217064	-0.051541	-4.500054
H	0.351952	1.373187	-5.086911
H	0.449143	-0.072730	-6.079097
C	2.347575	0.769642	-7.441560
H	1.536115	0.089055	-7.729680
H	3.261781	0.199660	-7.651204
C	2.316041	1.938406	-8.457319
H	3.209246	1.826170	-9.085151
H	1.482048	1.732812	-9.140435
C	0.972574	3.991519	-8.354133
H	1.155311	4.951192	-8.853664
H	0.386361	3.385745	-9.056310
H	0.327456	4.187590	-7.492832
C	3.418274	4.124459	-8.284881
H	3.160656	5.064030	-8.789647
H	3.994541	4.379711	-7.391420
H	4.101473	3.591364	-8.957872
C	-6.276822	15.579984	-0.109103
H	-6.596123	15.267967	0.893413
H	-5.183320	15.546250	-0.136975
H	-6.574642	16.630223	-0.221811
C	-7.623755	13.574186	-0.645365
H	-7.719685	13.604102	0.447322
H	-8.643494	13.543704	-1.049948

H	-7.139412	12.629642	-0.912523
C	-7.533211	15.467860	-2.239975
H	-7.627262	16.526258	-1.963116
H	-8.568009	15.114803	-2.340794
C	-6.897600	15.419648	-3.660233
H	-7.656446	14.968496	-4.311861
H	-6.862891	16.464533	-3.994047
C	-5.653567	13.722455	-4.936399
H	-4.936944	13.869568	-5.754115
H	-5.454524	12.738353	-4.501819
H	-6.644612	13.667381	-5.403701
C	-4.530840	15.784091	-4.244507
H	-4.030651	15.569415	-5.197269
H	-4.938773	16.798696	-4.331236
H	-3.758418	15.822223	-3.470802
N	8.247848	13.584825	0.068840
C	7.462059	14.181097	0.989042
H	7.098286	15.171028	0.754320
C	7.110313	13.581009	2.213541
H	6.477773	14.133621	2.895415
C	7.583879	12.282570	2.528202
C	8.410257	11.669712	1.553428
H	8.827468	10.682976	1.704307
C	8.707135	12.348845	0.355372
H	9.336305	11.877580	-0.386163
C	7.232695	11.625314	3.746092
C	6.084053	11.604771	5.705928
C	5.189232	12.244509	6.616118
C	4.698953	13.554870	6.389503
H	4.998115	14.129709	5.522632
C	3.814536	14.145295	7.312761
H	3.453201	15.148825	7.139563
C	3.846124	12.279557	8.659763
H	3.507172	11.797301	9.565198
C	4.733125	11.607056	7.796392
H	5.061334	10.611763	8.063356
C	7.310226	9.799306	5.091993
C	7.749251	8.462903	5.335440
C	7.378099	7.749442	6.502130
H	6.769784	8.202294	7.274074
C	8.583688	7.768276	4.424825
H	8.943158	8.233270	3.515487
C	8.978963	6.444843	4.701606
H	9.623140	5.923179	4.008229
C	7.819553	6.424795	6.686434
H	7.535813	5.884796	7.578756
C	3.254970	3.277447	-2.949172
H	4.178497	3.373603	-3.502542
C	3.278008	3.245326	-1.541169
H	4.236418	3.311910	-1.042617
C	0.955700	3.076637	-2.995795
H	0.050598	3.021111	-3.583806
C	0.882941	3.028711	-1.588931
H	-0.090450	2.926240	-1.126648
C	2.066845	3.120363	-0.813447
C	2.043449	3.093914	0.614593
C	0.891138	2.953015	2.566985
C	3.143802	3.228871	2.596517
C	4.356040	3.446111	3.318768
C	4.391247	3.507953	4.734762
H	3.493829	3.389823	5.327487
C	5.598514	3.617432	2.658069
H	5.677540	3.587047	1.579078
C	6.772478	3.818795	3.408161
H	7.720142	3.941631	2.903434
C	5.616062	3.720904	5.399052
H	5.645323	3.767378	6.478184
C	-0.350248	2.849060	3.263781
C	-1.591030	2.769015	2.581640
H	-1.644837	2.753007	1.500928
C	-2.793914	2.718539	3.313570
H	-3.738303	2.670890	2.791034
C	-1.668801	2.788521	5.324741
H	-1.718960	2.791116	6.403604
C	-0.417743	2.844755	4.679788
H	0.475842	2.882340	5.287487
C	-3.381032	13.423860	0.507421
H	-3.035519	14.411385	0.236664
C	-4.643942	11.574145	-0.051985
H	-5.292677	11.090456	-0.768747
C	-4.307035	10.920041	1.148978
H	-4.719918	9.936091	1.331445
C	-3.451854	11.554085	2.085756
C	-2.984181	12.844893	1.728915
H	-2.335059	13.413872	2.381556
C	-3.092166	10.933522	3.320085
C	-3.208389	9.170074	4.743609
C	-1.990719	11.001048	5.301711

N	-4.263661	5.071743	5.415098
C	-3.580738	7.812274	4.983151
C	-4.356489	7.074013	4.052544
H	-4.721455	7.519237	3.136351
C	-4.664972	5.723498	4.305643
H	-5.246504	5.160274	3.590246
C	-3.169794	7.111207	6.145042
H	-2.582939	7.592377	6.915331
C	-3.527113	5.757870	6.313300
H	-3.210496	5.220839	7.195335
C	-1.111472	11.682577	6.197485
C	-0.752205	11.142322	7.458301
H	-1.142915	10.191645	7.796563
C	0.108596	11.861343	8.312740
H	0.376724	11.458661	9.279227
C	0.298264	13.592262	6.799183
H	0.718286	14.559746	6.563438
C	-0.552413	12.947632	5.880853
H	-0.772043	13.450455	4.947888
C	3.226605	6.025834	-5.277777
H	4.166636	5.511830	-5.419490
C	0.924776	5.964168	-5.368788
H	0.028124	5.400270	-5.582441
C	0.822125	7.299371	-4.931955
H	-0.165216	7.729585	-4.822484
C	3.223273	7.364220	-4.837192
H	4.173542	7.848777	-4.652463
C	1.994158	8.042854	-4.642150
C	1.938130	9.388980	-4.168528
C	0.722864	11.199092	-3.536902
C	2.988275	11.253304	-3.405721
C	-0.542861	11.830482	-3.345088
C	-1.763890	11.152079	-3.584564
H	-1.786305	10.126696	-3.931013
C	-2.989460	11.813784	-3.375187
H	-3.917747	11.291630	-3.559640
C	-1.927981	13.751660	-2.732079
H	-2.008451	14.779376	-2.408073
C	-0.655580	13.173975	-2.908334
H	0.217992	13.784628	-2.720119
C	4.187639	11.923850	-3.016338
C	4.171898	13.221387	-2.443419
H	3.247747	13.763743	-2.292123
C	5.380329	13.835150	-2.057871
H	5.371081	14.822486	-1.618369
C	5.465080	11.326960	-3.168951
H	5.583076	10.340296	-3.598501
C	6.619942	12.017368	-2.751869
H	7.592774	11.558349	-2.859093
C	11.187228	14.518993	-1.088169
H	10.803139	13.496328	-1.146277
H	12.098237	14.547521	-1.698619
H	11.506536	14.690225	-0.052426
C	9.829003	16.456677	-0.465684
H	8.765075	16.403045	-0.216745
H	10.391591	16.274677	0.458575
H	10.032935	17.496150	-0.750744
C	10.587089	16.191169	-2.794231
H	10.687175	17.266527	-2.599902
H	11.605713	15.885798	-3.064426
C	9.759538	16.087201	-4.108764
H	9.508093	17.112179	-4.411518
H	10.440827	15.726647	-4.890727
C	7.286447	16.025002	-4.231218
H	6.709632	16.007690	-3.301133
H	7.472528	17.077338	-4.481046
H	6.649910	15.620342	-5.028200
C	8.609938	14.057619	-4.939445
H	7.745671	13.981080	-5.611377
H	9.504604	14.054388	-5.575171
H	8.640661	13.149417	-4.329976
N	-5.036963	0.939208	5.919005
N	-6.509219	3.337283	6.683604
C	-5.102013	0.338480	4.560257
H	-4.286713	-0.369592	4.365872
H	-6.034787	-0.208135	4.373807
H	-5.041501	1.124537	3.802411
C	-3.899024	0.375873	6.693369
H	-3.308224	-0.348681	6.119083
H	-3.223738	1.178589	7.002235
H	-4.213283	-0.141607	7.608363
C	-6.324698	0.747852	6.648528
H	-6.171336	0.169509	7.568532
H	-7.019757	0.134985	6.060402
C	-7.105043	2.021238	7.057334
H	-8.112867	1.915014	6.636013
H	-7.260613	1.955050	8.141809
C	-7.399511	4.078455	5.750956

H	-7.640944	5.089131	6.103190
H	-6.916384	4.177725	4.774849
H	-8.359128	3.575354	5.577278
C	-6.213873	4.148597	7.894439
H	-6.672163	5.144951	7.863458
H	-6.560844	3.679857	8.823842
H	-5.134021	4.285856	7.997182
N	9.087699	1.339882	5.302618
N	10.473281	3.482809	6.840816
N	10.144287	15.478613	-1.540948
N	8.539566	15.252617	-4.067185
N	-6.840060	14.715671	-1.171802
N	-5.556285	14.769181	-3.884081
N	6.424398	12.297351	4.596475
N	6.481863	10.346788	6.008835
N	7.703006	10.370257	3.932462
N	8.605801	5.768237	5.807740
N	3.387258	13.527993	8.433750
N	2.118632	3.192918	-3.669607
N	3.228877	3.232212	1.247543
N	0.836891	2.989479	1.215316
N	2.009682	3.080543	3.318232
N	-2.841594	2.726159	4.661767
N	6.791717	3.869355	4.754573
N	-4.195406	12.804421	-0.369908
N	-3.594882	9.704680	3.562969
N	-2.438414	9.780784	5.677462
N	-2.252380	11.616890	4.127680
N	0.625308	13.065556	7.995059
N	2.101848	5.328832	-5.543363
N	0.710971	9.922955	-3.980160
N	1.823726	11.911066	-3.206437
N	3.113423	9.999833	-3.896836
N	6.585361	13.249288	-2.205877
N	-3.084019	13.092856	-2.956455
Pd	1.664811	14.202453	9.414494
Pd	-4.863334	13.780913	-2.095755
Pd	8.604296	3.678117	5.787771
Pd	8.349175	14.331524	-1.888260
Pd	2.163897	3.242330	-5.777777
Pd	-4.659494	3.022222	5.669987
C	3.166716	6.361354	6.677550
C	3.708359	7.298992	5.729661
C	3.532228	7.020610	4.315570
C	2.291115	6.445351	3.968500
C	1.393926	6.159536	5.073160
C	1.962223	5.780500	6.338541
H	5.402320	7.814888	3.576895
H	2.634911	6.430037	0.590292
H	4.602949	7.850120	6.009388
C	4.448695	7.369026	3.309350
C	1.948874	6.280252	2.614998
H	0.412243	5.777547	4.807249
H	4.864464	7.325481	1.204064
C	2.877653	6.593234	1.633140
C	4.135690	7.119825	1.979598
H	0.957596	5.913283	2.369007
C	3.779302	6.207201	8.044198
H	4.783648	6.634899	8.092581
H	3.843519	5.154123	8.334868
C	1.163085	4.955471	7.313280
H	1.600409	3.959475	7.451501
H	0.135376	4.822352	6.964104
C	2.543858	9.695613	4.778542
C	2.215492	8.790249	5.914037
C	0.966420	8.186969	5.602096
C	0.520990	8.740697	4.294633
H	2.517167	9.083458	6.910719
H	0.208462	7.933561	6.330404
C	1.594705	10.313628	2.543320
C	0.714598	9.743509	1.423922
C	1.316775	11.808458	2.766205
H	2.633380	10.220704	2.222670
C	0.990620	10.519822	0.124952
H	-0.341236	9.832644	1.690863
H	0.919257	8.678592	1.289543
C	1.587261	12.583393	1.469989
H	0.270715	11.933958	3.074845
H	1.953388	12.182632	3.573642
C	0.754039	12.026054	0.308613
H	0.354284	10.121419	-0.672791
H	2.031920	10.355773	-0.183173
H	1.383667	13.647606	1.616527
H	2.654341	12.489099	1.229796
H	0.994971	12.567475	-0.611935
H	-0.313546	12.199343	0.509566
N	1.526848	9.578750	3.824888
O	-0.522881	8.502427	3.698143

O	3.536111	10.400234	4.645315
O	-1.370295	5.881353	3.353136
H	-1.566172	5.848978	2.408311
H	-1.207176	6.828841	3.520032
O	4.820896	10.709076	2.047815
H	4.445396	10.755633	2.943806
H	4.835602	9.760799	1.865101
H	3.177833	6.696293	8.819584
H	1.123448	5.413336	8.306249

Table5_1b_TSiii_TSi-ii_h2o_2_2

Property	Value
Charge	0
Electronic Energy, BS1 (a.u.)	-1135.375891
Thermal and entropic correction, BS1 (a.u.)	2.773481
Electronic Energy, BS2 (a.u.)	-1135.769436
Number of Imaginary Frequencies	0
Imaginary frequencies (cm-1)	None

Molecular Geometry in Cartesian Coordinates

C	8.376144	1.050101	6.914237
H	8.759112	0.593798	7.835627
H	7.623323	1.783410	7.216753
H	7.858046	0.251985	6.367957
C	9.539336	1.093463	4.758051
H	8.806610	0.292644	4.597560
H	9.374123	1.847593	3.983454
H	10.524322	0.651656	4.562213
C	10.747368	1.677119	6.812942
H	11.504012	1.160128	6.208997
H	10.680684	1.076216	7.729003
C	11.368300	3.033689	7.226450
H	12.374279	3.060827	6.788325
H	11.549195	2.973180	8.307286
C	11.401722	5.156998	5.998108
H	10.924960	5.232302	5.017158
H	11.504410	6.175540	6.392817
H	12.421468	4.791127	5.824574
C	10.180704	4.989946	8.115301
H	9.090101	4.981233	8.194348
H	10.567387	4.536130	9.036325
H	10.504033	6.038383	8.129395
N	3.147009	15.042966	10.469511
N	0.269486	15.324706	10.864580
C	3.908037	13.869131	10.972827
H	3.356255	12.949389	10.760860
H	4.900076	13.773819	10.513939
H	4.072089	13.893604	12.057416
C	3.956870	15.825996	9.498399
H	3.427363	15.905424	8.545452
H	4.156471	16.852195	9.831801
H	4.935819	15.373755	9.296080
C	2.692228	15.906478	11.598631
H	3.097569	15.547196	12.553220
H	3.095554	16.922686	11.502932
C	1.165236	16.059796	11.804737
H	0.955211	17.136912	11.784966
H	0.963500	15.767321	12.843234
C	-0.608228	16.267219	10.120280
H	-1.674232	16.026559	10.218618
H	-0.497518	17.309055	10.446087
H	-0.365061	16.243630	9.054076
C	-0.537572	14.303866	11.584368
H	-0.256602	13.301527	11.248703
H	-0.390326	14.325357	12.671350
H	-1.616352	14.415686	11.417340
N	2.418164	1.256201	-6.220636
N	2.374955	3.688408	-7.832793
C	3.655952	0.705305	-5.607830
H	3.452319	-0.036531	-4.825418
H	4.315107	0.213694	-6.334166
H	4.236859	1.511939	-5.151632
C	1.207055	0.624580	-5.632684
H	1.443486	-0.100407	-4.843805
H	0.562457	1.390655	-5.192653
H	0.598744	0.087493	-6.371149
C	2.437549	1.094227	-7.703969
H	1.592406	0.480803	-8.041856
H	3.322622	0.529483	-8.023933
C	2.409858	2.385088	-8.559477
H	3.280729	2.332517	-9.225394
H	1.550477	2.290588	-9.235571

C	1.141785	4.450163	-8.165993
H	1.351120	5.463911	-8.529814
H	0.531817	3.966916	-8.939357
H	0.508159	4.543838	-7.279538
C	3.591518	4.492082	-8.124412
H	3.360320	5.496580	-8.500346
H	4.187258	4.609909	-7.214906
H	4.246913	4.027780	-8.871817
C	-6.552385	15.795253	-0.668103
H	-7.085666	15.514835	0.248899
H	-5.490549	15.873190	-0.419671
H	-6.890872	16.807336	-0.923383
C	-7.651358	13.715899	-1.354480
H	-7.991350	13.812960	-0.315758
H	-8.558123	13.647411	-1.968377
H	-7.138208	12.754251	-1.443052
C	-7.254698	15.492721	-3.000850
H	-7.477383	16.548845	-2.801558
H	-8.219711	15.069689	-3.308136
C	-6.345852	15.468253	-4.254789
H	-6.953219	15.051077	-5.068198
H	-6.192392	16.515210	-4.546289
C	-4.979197	13.611753	-5.097476
H	-4.099766	13.650095	-5.752333
H	-4.939674	12.666400	-4.549411
H	-5.852388	13.554824	-5.759400
C	-3.902073	15.682996	-4.354898
H	-3.217444	15.349065	-5.144603
H	-4.219008	16.696240	-4.631808
H	-3.319840	15.776556	-3.433888
N	8.056390	13.640080	-0.028209
C	7.163145	14.189851	0.820775
H	6.839523	15.197726	0.603055
C	6.658022	13.521641	1.954104
H	5.954741	14.042610	2.590269
C	7.092425	12.205675	2.251855
C	8.028745	11.641900	1.348901
H	8.425433	10.644652	1.490115
C	8.471105	12.384546	0.237449
H	9.184277	11.949164	-0.447704
C	6.624887	11.492637	3.396945
C	5.386130	11.416932	5.296571
C	4.435190	12.019933	6.176363
C	3.895111	13.307767	5.928132
H	4.179011	13.884952	5.057565
C	2.984672	13.878582	6.838619
H	2.581314	14.864107	6.653557
C	3.065474	12.023995	8.207365
H	2.722232	11.533157	9.106973
C	3.982904	11.375008	7.355851
H	4.335085	10.391242	7.633637
C	6.717704	9.664931	4.738138
C	7.218262	8.353899	5.002459
C	6.906769	7.651993	6.194572
H	6.296536	8.096786	6.968973
C	8.059225	7.675834	4.083772
H	8.354682	8.125136	3.144709
C	8.523774	6.379696	4.377654
H	9.157514	5.862299	3.671921
C	7.421508	6.356943	6.404726
H	7.187165	5.824169	7.314784
C	3.469998	3.066030	-2.939343
H	4.396443	3.074941	-3.495934
C	3.499555	3.104769	-1.531770
H	4.463056	3.134236	-1.039287
C	1.165583	2.996028	-2.969605
H	0.256542	2.945787	-3.551721
C	1.096089	3.033217	-1.563031
H	0.121160	2.999590	-1.094477
C	2.287482	3.088557	-0.796487
C	2.269423	3.115894	0.630652
C	1.114286	3.137003	2.583363
C	3.383703	3.131999	2.606778
C	4.614980	3.156351	3.328199
C	4.660203	3.084462	4.742806
H	3.757868	2.979866	5.330945
C	5.866799	3.258056	2.670996
H	5.940208	3.310766	1.592859
C	7.055675	3.298152	3.424011
H	8.007754	3.380227	2.919494
C	5.899986	3.132137	5.410753
H	5.931195	3.075475	6.489085
C	-0.129955	3.230796	3.277507
C	-1.369079	3.272066	2.589315
H	-1.420841	3.225455	1.509379
C	-2.573573	3.358535	3.312887
H	-3.518119	3.382530	2.787981
C	-1.454516	3.385894	5.330492

H	-1.507167	3.440449	6.408338
C	-0.200947	3.298204	4.692096
H	0.692334	3.282394	5.303340
C	-3.932208	13.811746	0.692829
H	-3.708597	14.850064	0.494181
C	-4.906150	11.846232	-0.013924
H	-5.461281	11.318544	-0.776831
C	-4.530415	11.176551	1.167208
H	-4.818979	10.140143	1.285613
C	-3.818119	11.869701	2.177654
C	-3.519350	13.228697	1.906121
H	-2.992798	13.848588	2.620218
C	-3.445010	11.244744	3.405493
C	-3.484700	9.446854	4.785801
C	-2.502081	11.376443	5.464888
N	-4.495744	5.345588	5.541299
C	-3.829090	8.084779	5.038618
C	-4.513607	7.291185	4.084699
H	-4.828412	7.696263	3.131338
C	-4.818557	5.947199	4.377183
H	-5.350537	5.347909	3.651929
C	-3.501979	7.442790	6.259592
H	-2.996624	7.968657	7.059649
C	-3.847692	6.092135	6.459109
H	-3.603125	5.609952	7.394111
C	-1.765650	12.109425	6.443358
C	-1.396828	11.536563	7.685506
H	-1.682971	10.527323	7.949663
C	-0.627509	12.278551	8.601136
H	-0.345661	11.832862	9.543813
C	-0.573000	14.103577	7.203127
H	-0.252180	15.121280	7.033286
C	-1.333317	13.439807	6.220699
H	-1.577915	13.969374	5.309551
C	3.369699	6.066210	-4.951176
H	4.312842	5.587217	-5.172512
C	1.066188	5.950878	-4.942414
H	0.175045	5.380075	-5.162429
C	0.953722	7.248369	-4.404960
H	-0.036327	7.650950	-4.235616
C	3.355476	7.370291	-4.418401
H	4.302609	7.866683	-4.248261
C	2.120838	8.006020	-4.132042
C	2.056646	9.337923	-3.621089
C	0.833333	11.133927	-2.965359
C	3.098809	11.257336	-3.000384
C	-0.434636	11.767994	-2.795681
C	-1.651514	11.093307	-3.067663
H	-1.668370	10.065494	-3.406383
C	-2.878020	11.772181	-2.933685
H	-3.804076	11.259815	-3.153480
C	-1.823627	13.714727	-2.269850
H	-1.910930	14.746933	-1.961411
C	-0.551522	13.118163	-2.377356
H	0.319824	13.720684	-2.155457
C	4.297813	12.013418	-2.830923
C	4.277334	13.367253	-2.411523
H	3.348885	13.885621	-2.211226
C	5.484758	14.078257	-2.269787
H	5.464121	15.113140	-1.959662
C	5.579159	11.458302	-3.076782
H	5.700387	10.433149	-3.402982
C	6.733606	12.246681	-2.901462
H	7.708410	11.819534	-3.089946
C	11.155603	14.495368	-0.762298
H	10.751660	13.498475	-0.959621
H	12.130069	14.545009	-1.264180
H	11.360956	14.560436	0.313539
C	9.795867	16.428207	-0.116692
H	8.709927	16.399657	0.010864
H	10.246712	16.145198	0.842721
H	10.066197	17.478004	-0.285695
C	10.778954	16.316490	-2.363601
H	10.858929	17.379348	-2.101776
H	11.814566	16.011369	-2.561081
C	10.039011	16.255818	-3.722808
H	9.832942	17.295268	-4.008639
H	10.775205	15.916481	-4.462695
C	7.613781	16.276097	-4.100064
H	6.923703	16.277302	-3.251850
H	7.871191	17.324162	-4.297958
H	7.056100	15.925567	-4.977516
C	8.940715	14.334004	-4.783768
H	8.142205	14.332122	-5.536149
H	9.885740	14.375458	-5.339551
H	8.915629	13.364738	-4.277429
N	-4.408497	1.120869	5.804081
N	-6.328501	3.125094	6.696963

C	-4.259250	0.592396	4.421326
H	-3.294595	0.096792	4.255643
H	-5.030144	-0.141591	4.154675
H	-4.340773	1.405721	3.695841
C	-3.232227	0.762518	6.639990
H	-2.461197	0.216493	6.082052
H	-2.770484	1.668949	7.041444
H	-3.490787	0.134821	7.502016
C	-5.673274	0.627195	6.423763
H	-5.460435	0.004743	7.302321
H	-6.208759	-0.046507	5.742692
C	-6.699437	1.691855	6.883512
H	-7.642665	1.459581	6.372533
H	-6.908854	1.480098	7.939951
C	-7.338035	3.828982	5.861071
H	-7.741131	4.727634	6.344679
H	-6.889991	4.140639	4.913436
H	-8.203258	3.202506	5.610271
C	-6.156379	3.806418	8.007100
H	-6.781456	4.702456	8.109089
H	-6.397250	3.163502	8.862957
H	-5.114774	4.116318	8.130244
N	9.433784	1.713326	6.106275
N	10.603772	4.269197	6.885584
N	10.199466	15.532667	-1.233480
N	8.797222	15.430763	-3.789436
N	-6.743618	14.817645	-1.772147
N	-5.041495	14.751015	-4.143407
N	5.715013	12.111148	4.183976
N	5.855560	10.194780	5.638228
N	7.127919	10.255228	3.594649
N	8.219794	5.728126	5.517997
N	2.578989	13.258672	7.964917
N	2.327868	3.007187	-3.654199
N	3.463284	3.184741	1.258218
N	1.059497	3.123300	1.231891
N	2.241334	3.105730	3.330864
N	-2.624799	3.406467	4.659173
N	7.083456	3.234849	4.770846
N	-4.620882	13.142829	-0.254517
N	-3.780389	9.943821	3.563509
N	-2.848056	10.106544	5.775582
N	-2.760076	11.999801	4.292403
N	-0.212011	13.542261	8.376391
N	2.249024	5.364573	-5.218946
N	0.827883	9.842986	-3.367384
N	1.932480	11.876019	-2.704591
N	3.228548	9.984099	-3.434552
N	6.696852	13.537602	-2.510637
N	-2.969890	13.061516	-2.550379
Pd	1.446504	14.288663	9.416407
Pd	-4.838120	13.943206	-2.179993
Pd	8.832138	3.741161	5.823079
Pd	8.445353	14.520981	-1.894318
Pd	2.346126	3.331225	-5.731448
Pd	-4.463532	3.252951	5.671391
C	2.777787	5.650682	6.019180
C	3.004641	6.079818	4.703504
C	1.885507	6.344372	3.851590
C	0.649307	6.651087	4.477354
C	0.634944	6.717546	5.905667
C	1.580405	5.981350	6.639196
H	2.932262	6.162124	1.982312
H	3.584872	5.212600	6.600985
H	3.977948	5.958232	4.235178
C	1.959242	6.324808	2.440287
C	-0.486102	6.864395	3.670168
H	-0.247639	7.113437	6.398654
H	1.438779	5.809531	7.702893
C	-0.428794	6.750791	2.292419
C	0.828021	6.492595	1.661190
H	-1.431962	7.097885	4.150539
C	0.910617	6.428712	0.156908
H	0.229926	5.675072	-0.250403
H	1.924535	6.189881	-0.170691
H	0.627203	7.385262	-0.298662
C	-1.662436	6.971211	1.454817
H	-1.540942	7.840037	0.797857
H	-2.539099	7.154787	2.078668
H	-1.880163	6.120609	0.802236
C	3.202613	8.955612	4.038696
C	3.201311	8.300984	5.373032
C	2.051306	8.643024	6.015497
C	1.285009	9.554671	5.133249
H	4.139207	7.979740	5.822002
H	1.853634	8.630426	7.083598
C	1.646965	10.487302	2.785183
C	0.243549	10.160865	2.251796

C	1.797065	11.979617	3.124477
H	2.383861	10.230931	2.016350
C	-0.105659	11.045311	1.046838
H	-0.480984	10.319815	3.053823
H	0.207501	9.105492	1.983582
C	1.437742	12.851095	1.914815
H	1.133991	12.212157	3.968928
H	2.826407	12.172802	3.447084
C	0.028114	12.533233	1.398987
H	-1.119921	10.813528	0.699793
H	0.575406	10.808184	0.216892
H	1.521368	13.910079	2.173048
H	2.162659	12.666614	1.110548
H	-0.206208	13.159338	0.531302
H	-0.700044	12.786263	2.181117
N	2.002253	9.645205	3.930494
O	0.263929	10.169006	5.383983
O	4.081647	8.957685	3.181856
O	5.379908	6.514472	2.608876
H	5.602408	6.492236	1.669861
H	5.056553	7.423997	2.759368

Table5_1b_TSiii_TSi-ii_h2o_1wat

Property	Value
Charge	0
Electronic Energy, BS1 (a.u.)	-1135.336660
Thermal and entropic correction, BS1 (a.u.)	2.775370
Electronic Energy, BS2 (a.u.)	-1135.726038
Number of Imaginary Frequencies	0
Imaginary frequencies (cm-1)	None

Molecular Geometry in Cartesian Coordinates

C	8.377789	1.049798	6.914644
H	8.761251	0.594217	7.836187
H	7.624602	1.782819	7.216954
H	7.860033	0.251092	6.368897
C	9.540242	1.092941	4.758051
H	8.807907	0.291651	4.598136
H	9.374334	1.846662	3.983206
H	10.525396	0.651590	4.562037
C	10.748675	1.678003	6.812291
H	11.505508	1.161621	6.208062
H	10.682749	1.076993	7.728337
C	11.368693	3.035024	7.225696
H	12.374553	3.062905	6.787344
H	11.549867	2.974624	8.306492
C	11.400417	5.158589	5.997745
H	10.923955	5.233194	5.016603
H	11.501718	6.177314	6.392344
H	12.420667	4.793907	5.824662
C	10.179397	4.990158	8.114825
H	9.088779	4.980683	8.193670
H	10.566219	4.536394	9.035814
H	10.502026	6.038806	8.129209
N	3.146608	15.042402	10.470113
N	0.269096	15.324708	10.864908
C	3.907255	13.868389	10.973590
H	3.355255	12.948782	10.761571
H	4.899333	13.772800	10.514846
H	4.071154	13.892855	12.058203
C	3.956812	15.825158	9.499065
H	3.427348	15.904895	8.546124
H	4.156845	16.851249	9.832551
H	4.935572	15.372516	9.296722
C	2.691890	15.906138	11.599084
H	3.097140	15.546947	12.553747
H	3.095347	16.922282	11.503251
C	1.164910	16.059689	11.805094
H	0.955064	17.136839	11.785280
H	0.963060	15.767275	12.843585
C	-0.608437	16.267343	10.120535
H	-1.674479	16.026811	10.218769
H	-0.497643	17.309160	10.446370
H	-0.365177	16.243752	9.054349
C	-0.538158	14.304009	11.584663
H	-0.257387	13.301630	11.248965
H	-0.390915	14.325454	12.671646
H	-1.616915	14.416046	11.417630
N	2.417356	1.255979	-6.221801
N	2.374232	3.688744	-7.833104
C	3.655234	0.704748	-5.609489
H	3.451716	-0.037332	-4.827279

H	4.314159	0.213315	-6.336154
H	4.236331	1.511176	-5.153160
C	1.206320	0.624314	-5.633737
H	1.442855	-0.100845	-4.845044
H	0.561859	1.390343	-5.193418
H	0.597831	0.087423	-6.372197
C	2.436324	1.094502	-7.705192
H	1.590958	0.481382	-8.043069
H	3.321195	0.529672	-8.025567
C	2.408708	2.385664	-8.560251
H	3.279423	2.333140	-9.226374
H	1.549170	2.291556	-9.236200
C	1.141174	4.450899	-8.165821
H	1.350683	5.464713	-8.529362
H	0.530928	3.968063	-8.939223
H	0.507738	4.544439	-7.279221
C	3.590918	4.492263	-8.124621
H	3.359870	5.496961	-8.500106
H	4.186826	4.609608	-7.215161
H	4.246090	4.028113	-8.872312
C	-6.552256	15.795585	-0.669668
H	-7.085846	15.515763	0.247336
H	-5.490467	15.873381	-0.421011
H	-6.890412	16.807627	-0.925552
C	-7.651316	13.715978	-1.355174
H	-7.991251	13.813465	-0.316474
H	-8.558120	13.647363	-1.969000
H	-7.138278	12.754242	-1.443418
C	-7.254375	15.491994	-3.002344
H	-7.477387	16.548125	-2.803442
H	-8.219195	15.068632	-3.309775
C	-6.345153	15.467358	-4.256011
H	-6.952268	15.050061	-5.069547
H	-6.191619	16.514272	-4.547621
C	-4.978178	13.610704	-5.097949
H	-4.098505	13.648912	-5.752484
H	-4.938851	12.665464	-4.549663
H	-5.851133	13.553626	-5.760172
C	-3.901355	15.682118	-4.355429
H	-3.216357	15.347944	-5.144710
H	-4.218222	16.695244	-4.632847
H	-3.319545	15.776026	-3.434184
N	8.057211	13.640252	-0.027104
C	7.163859	14.190023	0.821805
H	6.840334	15.197934	0.604107
C	6.658585	13.521831	1.955088
H	5.955282	14.042794	2.591233
C	7.092884	12.205845	2.252847
C	8.029263	11.642045	1.349967
H	8.425904	10.644778	1.491163
C	8.471815	12.384697	0.238607
H	9.185099	11.949295	-0.446420
C	6.625202	11.492787	3.397868
C	5.386215	11.416884	5.297349
C	4.435173	12.019831	6.177084
C	3.895112	13.307671	5.928850
H	4.179172	13.884893	5.058359
C	2.984441	13.878397	6.839168
H	2.581053	14.863913	6.654099
C	3.065080	12.023770	8.207855
H	2.721714	11.532897	9.107398
C	3.982713	11.374861	7.356480
H	4.337653	10.391131	7.634313
C	6.717560	9.664778	4.738684
C	7.217722	8.353494	5.002521
C	6.905635	7.651037	6.194147
H	6.295074	8.095506	6.968457
C	8.058766	7.675652	4.083728
H	8.354625	8.125325	3.144970
C	8.522802	6.379207	4.377034
H	9.156510	5.861945	3.671175
C	7.419956	6.355734	6.403791
H	7.185138	5.822517	7.313467
C	3.469737	3.064625	-2.939971
H	4.396147	3.073188	-3.496625
C	3.499426	3.103512	-1.532408
H	4.462976	3.132755	-1.040010
C	1.165313	2.995236	-2.970033
H	0.256211	2.945083	-3.552064
C	1.095936	3.032659	-1.563458
H	0.121037	2.999366	-1.094817
C	2.287413	3.087729	-0.797025
C	2.269494	3.115216	0.630109
C	1.114541	3.136926	2.582923
C	3.383953	3.131024	2.606120
C	4.615281	3.155003	3.327457
C	4.660578	3.082402	4.742018
H	3.758328	2.977338	5.330185

C	5.867067	3.257069	2.670248
H	5.940428	3.310313	1.592131
C	7.055965	3.296947	3.423249
H	8.008021	3.379349	2.918737
C	5.900378	3.129874	5.409930
H	5.931600	3.072682	6.488232
C	-0.129600	3.231448	3.277143
C	-1.368756	3.272816	2.589029
H	-1.420582	3.225818	1.509114
C	-2.573184	3.359758	3.312658
H	-3.517744	3.383707	2.787777
C	-1.453983	3.387699	5.330155
H	-1.506522	3.442733	6.407983
C	-0.200474	3.299498	4.691704
H	0.692852	3.283789	5.302884
C	-3.932091	13.811600	0.692271
H	-3.708461	14.849903	0.493563
C	-4.906383	11.846154	-0.014141
H	-5.461812	11.318484	-0.776842
C	-4.530378	11.176470	1.166897
H	-4.819048	10.140104	1.285429
C	-3.817884	11.869647	2.177194
C	-3.518961	13.228571	1.905479
H	-2.992202	13.848470	2.619417
C	-3.444906	11.244838	3.405146
C	-3.485203	9.447216	4.785778
C	-2.502563	11.376838	5.464798
N	-4.496423	5.346036	5.541651
C	-3.829717	8.085197	5.038716
C	-4.514173	7.291501	4.084840
H	-4.828931	7.696438	3.131399
C	-4.819179	5.947568	4.377483
H	-5.351175	5.348201	3.652309
C	-3.502761	7.443355	6.259803
H	-2.997526	7.969328	7.059863
C	-3.848485	6.092718	6.459437
H	-3.604036	5.610661	7.394540
C	-1.766279	12.109846	6.443371
C	-1.397810	11.537000	7.685627
H	-1.684262	10.527860	7.949822
C	-0.628432	12.278855	8.601315
H	-0.346851	11.833148	9.544061
C	-0.573258	14.103772	7.203196
H	-0.252166	15.121390	7.033363
C	-1.333629	13.440129	6.220708
H	-1.578010	13.969699	5.309508
C	3.369379	6.065629	-4.951167
H	4.312463	5.586832	-5.173176
C	1.065891	5.950062	-4.941335
H	0.174707	5.379259	-5.161182
C	0.953538	7.247294	-4.403246
H	-0.036502	7.649628	-4.233202
C	3.355280	7.369489	-4.417841
H	4.302433	7.865943	-4.247984
C	2.120716	8.004980	-4.130648
C	2.056645	9.336750	-3.619336
C	0.833360	11.132742	-2.963617
C	3.098831	11.256240	-2.998863
C	-0.434607	11.766925	-2.794407
C	-1.651478	11.092147	-3.066213
H	-1.668335	10.064128	-3.404286
C	-2.877954	11.771198	-2.932917
H	-3.804010	11.258802	-3.152635
C	-1.823555	13.714046	-2.269955
H	-1.910842	14.746438	-1.962130
C	-0.551473	13.117329	-2.376852
H	0.319884	13.719899	-2.155128
C	4.297819	12.012374	-2.829468
C	4.277276	13.366220	-2.410075
H	3.348827	13.884589	-2.209807
C	5.484657	14.077281	-2.268342
H	5.463999	15.112168	-1.958243
C	5.579180	11.457297	-3.075312
H	5.700398	10.432133	-3.401484
C	6.733598	12.245722	-2.899953
H	7.708428	11.818613	-3.088380
C	11.155825	14.495818	-0.762400
H	10.751929	13.498785	-0.959113
H	12.130124	14.545318	-1.264619
H	11.361535	14.561432	0.313336
C	9.796001	16.428735	-0.117249
H	8.710089	16.400149	0.010540
H	10.247055	16.146127	0.842184
H	10.066252	17.478477	-0.286730
C	10.778517	16.316223	-2.364353
H	10.858584	17.379165	-2.102892
H	11.814057	16.011041	-2.562111
C	10.038066	16.255119	-3.723257

H	9.831739	17.294468	-4.009270
H	10.774027	15.915712	-4.463342
C	7.612690	16.274943	-4.099582
H	6.922909	16.276227	-3.251126
H	7.869848	17.323012	-4.297783
H	7.054771	15.924142	-4.976776
C	8.939631	14.332862	-4.783299
H	8.140789	14.330642	-5.535328
H	9.884411	14.374320	-5.339499
H	8.914931	13.363738	-4.276675
N	-4.406577	1.121388	5.804187
N	-6.327834	3.124440	6.696930
C	-4.256433	0.592904	4.421533
H	-3.291186	0.098365	4.256134
H	-5.026436	-0.141994	4.154813
H	-4.338708	1.406040	3.695919
C	-3.230414	0.763876	6.640609
H	-2.458983	0.217934	6.083142
H	-2.769184	1.670664	7.041838
H	-3.488974	0.136431	7.502819
C	-5.671293	0.626927	6.423361
H	-5.458389	0.004261	7.301751
H	-6.206324	-0.046764	5.741922
C	-6.698021	1.690974	6.883253
H	-7.641128	1.458271	6.372247
H	-6.907325	1.478931	7.939657
C	-7.337759	3.827965	5.861190
H	-7.741418	4.726232	6.345045
H	-6.889886	4.140176	4.913657
H	-8.202599	3.201052	5.610169
C	-6.156007	3.805648	8.007167
H	-6.781505	4.701376	8.109301
H	-6.396536	3.162495	8.862942
H	-5.114553	4.116049	8.130320
N	9.434821	1.713257	6.106081
N	10.603162	4.269969	6.885027
N	10.199422	15.532790	-1.233768
N	8.796378	15.429858	-3.789199
N	-6.743458	14.817470	-1.773265
N	-5.040822	14.750151	-4.144129
N	5.715278	12.111262	4.184879
N	5.855457	10.194596	5.638849
N	7.128155	10.255356	3.595495
N	8.218287	5.727150	5.516944
N	2.578545	13.258420	7.965353
N	2.327542	3.006011	-3.654746
N	3.463427	3.183884	1.257565
N	1.059626	3.123051	1.231457
N	2.241647	3.105197	3.330323
N	-2.624333	3.408190	4.658939
N	7.083831	3.233027	4.770061
N	-4.621123	13.142739	-0.254861
N	-3.780476	9.943982	3.563304
N	-2.848691	10.106997	5.775591
N	-2.760070	11.999975	4.292078
N	-0.212566	13.542440	8.376542
N	2.248643	5.363995	-5.218701
N	0.827920	9.841692	-3.365235
N	1.932493	11.874883	-2.702961
N	3.228565	9.983025	-3.433126
N	6.696756	13.536650	-2.509128
N	-2.969803	13.060757	-2.550373
Pd	1.446044	14.288493	9.416822
Pd	-4.838005	13.942686	-2.180547
Pd	8.832048	3.740654	5.822359
Pd	8.445312	14.520521	-1.893729
Pd	2.345666	3.330825	-5.731878
Pd	-4.462948	3.253429	5.671345
C	2.770990	5.649316	6.031921
C	3.023662	6.188953	4.726685
C	1.879905	6.352548	3.845296
C	0.652643	6.659239	4.468656
C	0.691094	6.817783	5.910792
C	1.590901	5.974541	6.641178
H	2.930954	6.159550	1.983839
H	3.562933	5.151257	6.580718
H	3.983319	6.012503	4.250352
C	1.958377	6.318502	2.442173
C	-0.485394	6.862850	3.672758
H	-0.210580	7.175730	6.400877
H	1.407829	5.747971	7.685643
C	-0.428941	6.750998	2.289516
C	0.821654	6.490450	1.660671
H	-1.427838	7.106735	4.153366
C	0.909039	6.428453	0.156376
H	0.226882	5.675825	-0.253064
H	1.922419	6.187687	-0.171473
H	0.627237	7.385317	-0.299530

C	-1.662457	6.976385	1.455038
H	-1.539374	7.844964	0.797993
H	-2.538500	7.161909	2.079190
H	-1.884873	6.126205	0.802593
C	3.202594	8.942509	4.037299
C	3.184198	8.200432	5.332881
C	1.983935	8.561501	5.999827
C	1.278962	9.545084	5.136202
H	4.124373	8.047944	5.839791
H	1.888439	8.665290	7.070643
C	1.650587	10.488696	2.788794
C	0.247534	10.162263	2.254489
C	1.797881	11.980972	3.128219
H	2.387941	10.229577	2.023152
C	-0.101034	11.046831	1.049455
H	-0.480285	10.320984	3.056037
H	0.209160	9.106954	1.986066
C	1.439116	12.852486	1.915694
H	1.134438	12.213559	3.969653
H	2.827103	12.176782	3.448527
C	0.029713	12.534694	1.399202
H	-1.115063	10.812283	0.701760
H	0.577878	10.809907	0.217197
H	1.522685	13.911467	2.173973
H	2.164359	12.667989	1.111709
H	-0.204211	13.160920	0.531509
H	-0.698867	12.787566	2.181000
N	2.007798	9.646359	3.939654
O	0.266271	10.173423	5.384003
O	4.081747	8.961160	3.183252
O	5.379003	6.514284	2.609276
H	5.601351	6.492551	1.670215
H	5.058143	7.423652	2.757546

Table5_1b_TSiii_DG_h2o_2

**Property**	**Value**
Charge	0
Electronic Energy, BS1 (a.u.)	-1135.380676
Thermal and entropic correction, BS1 (a.u.)	2.781115
Electronic Energy, BS2 (a.u.)	-1135.767313
Number of Imaginary Frequencies	0
Imaginary frequencies (cm-1)	None

Molecular Geometry in Cartesian Coordinates

C	8.376144	1.050101	6.914237
H	8.759112	0.593798	7.835627
H	7.623323	1.783410	7.216753
H	7.858046	0.251985	6.367957
C	9.539336	1.093463	4.758051
H	8.806610	0.292644	4.597560
H	9.374123	1.847593	3.983454
H	10.524322	0.651656	4.562213
C	10.747368	1.677119	6.812942
H	11.504012	1.160128	6.208997
H	10.680684	1.076216	7.729003
C	11.368300	3.033689	7.226450
H	12.374279	3.060827	6.788325
H	11.549195	2.973180	8.307286
C	11.401722	5.156998	5.998108
H	10.924960	5.232302	5.017158
H	11.504410	6.175540	6.392817
H	12.421468	4.791127	5.824574
C	10.180704	4.989946	8.115301
H	9.090101	4.981233	8.194348
H	10.567387	4.536130	9.036325
H	10.504033	6.038383	8.129395
N	3.147009	15.042966	10.469511
N	0.269486	15.324706	10.864580
C	3.908037	13.869131	10.972827
H	3.356255	12.949389	10.760860
H	4.900076	13.773819	10.513939
H	4.072089	13.893604	12.057416
C	3.956870	15.825996	9.498399
H	3.427363	15.905424	8.545452
H	4.156471	16.852195	9.831801
H	4.935819	15.373755	9.296080
C	2.692228	15.906478	11.598631
H	3.097569	15.547196	12.553220
H	3.095554	16.922686	11.502932
C	1.165236	16.059796	11.804737
H	0.955211	17.136912	11.784966
H	0.963500	15.767321	12.843234

C	-0.608228	16.267219	10.120280
H	-1.674232	16.026559	10.218618
H	-0.497518	17.309055	10.446087
H	-0.365061	16.243630	9.054076
C	-0.537572	14.303866	11.584368
H	-0.256602	13.301527	11.248703
H	-0.390326	14.325357	12.671350
H	-1.616352	14.415686	11.417340
N	2.418164	1.256201	-6.220636
N	2.374955	3.688408	-7.832793
C	3.655952	0.705305	-5.607830
H	3.452319	-0.036531	-4.825418
H	4.315107	0.213694	-6.334166
H	4.236859	1.511939	-5.151632
C	1.207055	0.624580	-5.632684
H	1.443486	-0.100407	-4.843805
H	0.562457	1.390655	-5.192653
H	0.598744	0.087493	-6.371149
C	2.437549	1.094227	-7.703969
H	1.592406	0.480803	-8.041856
H	3.322622	0.529483	-8.023933
C	2.409858	2.385088	-8.559477
H	3.280729	2.332517	-9.225394
H	1.550477	2.290588	-9.235571
C	1.141785	4.450163	-8.165993
H	1.351120	5.463911	-8.529814
H	0.531817	3.966916	-8.939357
H	0.508159	4.543838	-7.279538
C	3.591518	4.492082	-8.124412
H	3.360320	5.496580	-8.500346
H	4.187258	4.609909	-7.214906
H	4.246913	4.027780	-8.871817
C	-6.552385	15.795253	-0.668103
H	-7.085666	15.514835	0.248899
H	-5.490549	15.873190	-0.419671
H	-6.890872	16.807336	-0.923383
C	-7.651358	13.715899	-1.354480
H	-7.991350	13.812960	-0.315758
H	-8.558123	13.647411	-1.968377
H	-7.138208	12.754251	-1.443052
C	-7.254698	15.492721	-3.000850
H	-7.477383	16.548845	-2.801558
H	-8.219711	15.069689	-3.308136
C	-6.345852	15.468253	-4.254789
H	-6.953219	15.051077	-5.068198
H	-6.192392	16.515210	-4.546289
C	-4.979197	13.611753	-5.097476
H	-4.099766	13.650095	-5.752333
H	-4.939674	12.666400	-4.549411
H	-5.852388	13.554824	-5.759400
C	-3.902073	15.682996	-4.354898
H	-3.217444	15.349065	-5.144603
H	-4.219008	16.696240	-4.631808
H	-3.319840	15.776556	-3.433888
N	8.056390	13.640080	-0.028209
C	7.163145	14.189851	0.820775
H	6.839523	15.197726	0.603055
C	6.658022	13.521641	1.954104
H	5.954741	14.042610	2.590269
C	7.092425	12.205675	2.251855
C	8.028745	11.641900	1.348901
H	8.425433	10.644652	1.490115
C	8.471105	12.384546	0.237449
H	9.184277	11.949164	-0.447704
C	6.624887	11.492637	3.396945
C	5.386130	11.416932	5.296571
C	4.435190	12.019933	6.176363
C	3.895111	13.307767	5.928132
H	4.179011	13.884952	5.057565
C	2.984672	13.878582	6.838619
H	2.581314	14.864107	6.653557
C	3.065474	12.023995	8.207365
H	2.722232	11.533157	9.106973
C	3.982904	11.375008	7.355851
H	4.340750	10.391242	7.633637
C	6.717704	9.664931	4.738138
C	7.218262	8.353899	5.002459
C	6.906769	7.651993	6.194572
H	6.296536	8.096786	6.968973
C	8.059225	7.675834	4.083772
H	8.354682	8.125136	3.144709
C	8.523774	6.379696	4.377654
H	9.157514	5.862299	3.671921
C	7.421508	6.356943	6.404726
H	7.187165	5.824169	7.314784
C	3.469998	3.066030	-2.939343
H	4.396443	3.074941	-3.495934
C	3.499555	3.104769	-1.531770

H	4.463056	3.134236	-1.039287
C	1.165583	2.996028	-2.969605
H	0.256542	2.945787	-3.551721
C	1.096089	3.033217	-1.563031
H	0.121160	2.999590	-1.094477
C	2.287482	3.088557	-0.796487
C	2.269423	3.115894	0.630652
C	1.114286	3.137003	2.583363
C	3.383703	3.131999	2.606778
C	4.614980	3.156351	3.328199
C	4.660203	3.084462	4.742806
H	3.757868	2.979866	5.330945
C	5.866799	3.258056	2.670996
H	5.940208	3.310766	1.592859
C	7.055675	3.298152	3.424011
H	8.007754	3.380227	2.919494
C	5.899986	3.132137	5.410753
H	5.931195	3.075475	6.489085
C	-0.129955	3.230796	3.277507
C	-1.369079	3.272066	2.589315
H	-1.420841	3.225455	1.509379
C	-2.573573	3.358535	3.312887
H	-3.518119	3.382530	2.787981
C	-1.454516	3.385894	5.330492
H	-1.507167	3.440449	6.408338
C	-0.200947	3.298204	4.692096
H	0.692334	3.282394	5.303340
C	-3.932208	13.811746	0.692829
H	-3.708597	14.850064	0.494181
C	-4.906150	11.846232	-0.013924
H	-5.461281	11.318544	-0.776831
C	-4.530415	11.176551	1.167208
H	-4.818979	10.140143	1.285613
C	-3.818119	11.869701	2.177654
C	-3.519350	13.228697	1.906121
H	-2.992798	13.848588	2.620218
C	-3.445010	11.244744	3.405493
C	-3.484700	9.446854	4.785801
C	-2.502081	11.376443	5.464888
N	-4.495744	5.345588	5.541299
C	-3.829090	8.084779	5.038618
C	-4.513607	7.291185	4.084699
H	-4.828412	7.696263	3.131338
C	-4.818557	5.947199	4.377183
H	-5.350537	5.347909	3.651929
C	-3.501979	7.442790	6.259592
H	-2.996624	7.968657	7.059649
C	-3.847692	6.092135	6.459109
H	-3.603125	5.609952	7.394111
C	-1.765650	12.109425	6.443358
C	-1.396828	11.536563	7.685506
H	-1.682971	10.527323	7.949663
C	-0.627509	12.278551	8.601136
H	-0.345661	11.832862	9.543813
C	-0.573000	14.103577	7.203127
H	-0.252180	15.121280	7.033286
C	-1.333317	13.439807	6.220699
H	-1.577915	13.969374	5.309551
C	3.369699	6.066210	-4.951176
H	4.312842	5.587217	-5.172512
C	1.066188	5.950878	-4.942414
H	0.175045	5.380075	-5.162429
C	0.953722	7.248369	-4.404960
H	-0.036327	7.650950	-4.235616
C	3.355476	7.370291	-4.418401
H	4.302609	7.866683	-4.248261
C	2.120838	8.006020	-4.132042
C	2.056646	9.337923	-3.621089
C	0.833333	11.133927	-2.965359
C	3.098809	11.257336	-3.000384
C	-0.434636	11.767994	-2.795681
C	-1.651514	11.093307	-3.067663
H	-1.668370	10.065494	-3.406383
C	-2.878020	11.772181	-2.933685
H	-3.804076	11.259815	-3.153480
C	-1.823627	13.714727	-2.269850
H	-1.910930	14.746933	-1.961411
C	-0.551522	13.118163	-2.377356
H	0.319824	13.720684	-2.155457
C	4.297813	12.013418	-2.830923
C	4.277334	13.367253	-2.411523
H	3.348885	13.885621	-2.211226
C	5.484758	14.078257	-2.269787
H	5.464121	15.113140	-1.959662
C	5.579159	11.458302	-3.076782
H	5.700387	10.433149	-3.402982
C	6.733606	12.246681	-2.901462
H	7.708410	11.819534	-3.089946

C	11.155603	14.495368	-0.762298
H	10.751660	13.498475	-0.959621
H	12.130069	14.545009	-1.264180
H	11.360956	14.560436	0.313539
C	9.795867	16.428207	-0.116692
H	8.709927	16.399657	0.010864
H	10.246712	16.145198	0.842721
H	10.066197	17.478004	-0.285695
C	10.778954	16.316490	-2.363601
H	10.858929	17.379348	-2.101776
H	11.814566	16.011369	-2.561081
C	10.039011	16.255818	-3.722808
H	9.832942	17.295268	-4.008639
H	10.775205	15.916481	-4.462695
C	7.613781	16.276097	-4.100064
H	6.923703	16.277302	-3.251850
H	7.871191	17.324162	-4.297958
H	7.056100	15.925567	-4.977516
C	8.940715	14.334004	-4.783768
H	8.142205	14.332122	-5.536149
H	9.885740	14.375458	-5.339551
H	8.915629	13.364738	-4.277429
N	-4.408497	1.120869	5.804081
N	-6.328501	3.125094	6.696963
C	-4.259250	0.592396	4.421326
H	-3.294595	0.096792	4.255643
H	-5.030144	-0.141591	4.154675
H	-4.340773	1.405721	3.695841
C	-3.232227	0.762518	6.639990
H	-2.461197	0.216493	6.082052
H	-2.770484	1.668949	7.041444
H	-3.490787	0.134821	7.502016
C	-5.673274	0.627195	6.423763
H	-5.460435	0.004743	7.302321
H	-6.208759	-0.046507	5.742692
C	-6.699437	1.691855	6.883512
H	-7.642665	1.459581	6.372533
H	-6.908854	1.480098	7.939951
C	-7.338035	3.828982	5.861071
H	-7.741131	4.727634	6.344679
H	-6.889991	4.140639	4.913436
H	-8.203258	3.202506	5.610271
C	-6.156379	3.806418	8.007100
H	-6.781456	4.702456	8.109089
H	-6.397250	3.163502	8.862957
H	-5.114774	4.116318	8.130244
N	9.433784	1.713326	6.106275
N	10.603772	4.269197	6.885584
N	10.199466	15.532667	-1.233480
N	8.797222	15.430763	-3.789436
N	-6.743618	14.817645	-1.772147
N	-5.041495	14.751015	-4.143407
N	5.715013	12.111148	4.183976
N	5.855560	10.194780	5.638228
N	7.127919	10.255228	3.594649
N	8.219794	5.728126	5.517997
N	2.578989	13.258672	7.964917
N	2.327868	3.007187	-3.654199
N	3.463284	3.184741	1.258218
N	1.059497	3.123300	1.231891
N	2.241334	3.105730	3.330864
N	-2.624799	3.406467	4.659173
N	7.083456	3.234849	4.770846
N	-4.620882	13.142829	-0.254517
N	-3.780389	9.943821	3.563509
N	-2.848056	10.106544	5.775582
N	-2.760076	11.999801	4.292403
N	-0.212011	13.542261	8.376391
N	2.249024	5.364573	-5.218946
N	0.827883	9.842986	-3.367384
N	1.932480	11.876019	-2.704591
N	3.228548	9.984099	-3.434552
N	6.696852	13.537602	-2.510637
N	-2.969890	13.061516	-2.550379
Pd	1.446504	14.288663	9.416407
Pd	-4.838120	13.943206	-2.179993
Pd	8.832138	3.741161	5.823079
Pd	8.445353	14.520981	-1.894318
Pd	2.346126	3.331225	-5.731448
Pd	-4.463532	3.252951	5.671391
C	2.766457	5.650682	6.047505
C	3.044296	6.312079	4.754489
C	1.874177	6.361367	3.840260
C	0.654972	6.662417	4.460359
C	0.748242	6.915818	5.916997
C	1.603065	5.964355	6.644861
H	2.932262	6.162124	1.987977
H	3.545217	5.093637	6.561330

H	3.989278	6.077195	4.269167
C	1.959242	6.313478	2.445952
C	-0.486102	6.853065	3.675833
H	-0.173995	7.232400	6.404319
H	1.376465	5.679238	7.668904
C	-0.428794	6.745126	2.286754
C	0.816691	6.486930	1.661190
H	-1.426297	7.103550	4.156204
C	0.910617	6.428712	0.156908
H	0.229926	5.675072	-0.256068
H	1.924535	6.189881	-0.170691
H	0.627203	7.385262	-0.298662
C	-1.662436	6.971211	1.454817
H	-1.540942	7.840037	0.797857
H	-2.539099	7.154787	2.078668
H	-1.885828	6.120609	0.802236
C	3.202613	8.927287	4.033031
C	3.167322	8.091382	5.288058
C	1.915348	8.473077	5.981508
C	1.273679	9.532011	5.138914
H	4.110882	8.121363	5.855991
H	1.927278	8.698405	7.055273
C	1.652630	10.487302	2.790848
C	0.249214	10.160865	2.257461
C	1.797065	11.979617	3.130142
H	2.389526	10.225266	2.027680
C	-0.099994	11.045311	1.052503
H	-0.480984	10.319815	3.059488
H	0.207501	9.105492	1.989247
C	1.437742	12.851095	1.914815
H	1.133991	12.212157	3.968928
H	2.826407	12.178467	3.447084
C	0.028114	12.533233	1.398987
H	-1.114256	10.807863	0.705458
H	0.575406	10.808184	0.216892
H	1.521368	13.910079	2.173048
H	2.162659	12.666614	1.110548
H	-0.206208	13.159338	0.531302
H	-0.700044	12.786263	2.181117
N	2.013583	9.645205	3.947489
O	0.269594	10.174671	5.383983
O	4.081647	8.963350	3.181856
O	5.379908	6.514472	2.608876
H	5.602408	6.492236	1.669861
H	5.062218	7.423997	2.753703

Table5_1b_TSiii_reactant_2wat

Property	Value
Charge	0
Electronic Energy, BS1 (a.u.)	-1211.818734
Thermal and entropic correction, BS1 (a.u.)	2.796168
Electronic Energy, BS2 (a.u.)	-1212.247176
Number of Imaginary Frequencies	0
Imaginary frequencies (cm-1)	None

Molecular Geometry in Cartesian Coordinates

C	8.411921	1.027625	6.966465
H	8.782614	0.593506	7.903488
H	7.629942	1.741877	7.239207
H	7.931910	0.208300	6.416770
C	9.628227	1.081205	4.840096
H	8.923933	0.257025	4.670629
H	9.458852	1.823092	4.054658
H	10.630737	0.668250	4.672113
C	10.766929	1.718749	6.919019
H	11.545893	1.194966	6.350313
H	10.686914	1.145978	7.851830
C	11.358327	3.097219	7.302288
H	12.373418	3.125233	6.885875
H	11.516051	3.074403	8.388164
C	11.378314	5.163861	5.979202
H	10.904329	5.190775	4.994189
H	11.470951	6.199878	6.328377
H	12.401554	4.799119	5.825238
C	10.151578	5.080455	8.098111
H	9.061527	5.060305	8.179452
H	10.544817	4.675680	9.039075
H	10.460425	6.132671	8.063210
N	3.102113	15.071578	10.543915
N	0.213656	15.331234	10.864523
C	3.867517	13.904846	11.057080
H	3.332720	12.978997	10.828603

H	4.870221	13.825045	10.618857
H	4.008378	13.925436	12.145024
C	3.921952	15.869982	9.594062
H	3.413729	15.942117	8.628871
H	4.096369	16.898617	9.933860
H	4.912652	15.434290	9.414067
C	2.611796	15.922598	11.667640
H	2.993952	15.558134	12.629811
H	3.011138	16.941981	11.590818
C	1.078902	16.065260	11.833651
H	0.862943	17.141228	11.814927
H	0.850878	15.765042	12.864471
C	-0.642497	16.274133	10.096013
H	-1.710552	16.031518	10.161303
H	-0.543438	17.315390	10.427308
H	-0.366514	16.253645	9.037685
C	-0.612886	14.306946	11.556901
H	-0.319354	13.305805	11.228366
H	-0.499343	14.326586	12.647927
H	-1.686267	14.417055	11.357167
N	2.416789	1.301875	-6.324937
N	2.329560	3.872458	-7.705287
C	3.667258	0.709156	-5.780186
H	3.479501	-0.102847	-5.066510
H	4.320752	0.291591	-6.556322
H	4.248595	1.475525	-5.259780
C	1.218951	0.606333	-5.783732
H	1.472155	-0.195519	-5.078851
H	0.581282	1.319421	-5.253354
H	0.598277	0.146086	-6.562669
C	2.420494	1.278297	-7.817069
H	1.572258	0.697597	-8.201613
H	3.302269	0.746331	-8.196702
C	2.386415	2.642748	-8.549476
H	3.262249	2.661926	-9.210635
H	1.532927	2.601748	-9.238327
C	1.076978	4.632585	-7.959276
H	1.260198	5.680156	-8.229040
H	0.471158	4.208471	-8.769774
H	0.449271	4.628479	-7.064095
C	3.525425	4.727479	-7.929359
H	3.268599	5.754120	-8.218851
H	4.121583	4.783482	-7.014287
H	4.189188	4.344002	-8.714305
C	-6.518695	15.734352	-0.599248
H	-6.994077	15.434317	0.343132
H	-5.446569	15.833149	-0.409255
H	-6.892119	16.741081	-0.825414
C	-7.615228	13.641082	-1.246609
H	-7.904095	13.724525	-0.191455
H	-8.550321	13.559630	-1.814746
H	-7.090218	12.689618	-1.368267
C	-7.337330	15.438600	-2.894593
H	-7.565656	16.489384	-2.674781
H	-8.310522	15.002227	-3.153507
C	-6.496524	15.438771	-4.195306
H	-7.136482	15.010837	-4.977578
H	-6.382628	16.490078	-4.489450
C	-5.129349	13.624415	-5.125743
H	-4.289205	13.695525	-5.827753
H	-5.030104	12.675098	-4.592057
H	-6.036026	13.547879	-5.738785
C	-4.065753	15.712684	-4.412200
H	-3.415367	15.405402	-5.240700
H	-4.419124	16.721996	-4.657337
H	-3.438648	15.806474	-3.521065
N	8.101363	13.690459	0.080530
C	7.179647	14.215798	0.914394
H	6.858267	15.226794	0.707236
C	6.645850	13.524184	2.019913
H	5.922315	14.030982	2.644913
C	7.083571	12.207421	2.308757
C	8.053714	11.669635	1.427433
H	8.467168	10.679757	1.572298
C	8.520279	12.434970	0.341826
H	9.262617	12.018545	-0.323909
C	6.600000	11.476730	3.435228
C	5.348650	11.390537	5.325799
C	4.417916	12.007070	6.217080
C	3.901766	13.306377	5.977251
H	4.186405	13.880915	5.105407
C	3.016754	13.895450	6.900017
H	2.633083	14.890041	6.722233
C	3.076494	12.036848	8.266421
H	2.736203	11.552917	9.170967
C	3.968747	11.369472	7.401777
H	4.309713	10.379372	7.675190
C	6.676134	9.635833	4.759090

C	7.172923	8.322133	5.018986
C	6.841582	7.609528	6.199500
H	6.218735	8.047561	6.968040
C	8.028006	7.651014	4.108019
H	8.342207	8.109834	3.179770
C	8.482777	6.349306	4.395365
H	9.125172	5.836300	3.694346
C	7.347104	6.309875	6.403676
H	7.095014	5.767248	7.303129
C	3.458120	2.857531	-2.895365
H	4.384297	2.825879	-3.451422
C	3.493873	2.935327	-1.489763
H	4.459144	2.952177	-1.000483
C	1.157552	2.834797	-2.917995
H	0.244844	2.781090	-3.494443
C	1.091713	2.917483	-1.513634
H	0.117325	2.917208	-1.043001
C	2.285224	2.971693	-0.751601
C	2.270963	3.043506	0.673498
C	1.114407	3.138867	2.622373
C	3.383485	3.073666	2.650193
C	4.613873	3.080896	3.374154
C	4.654422	3.051930	4.790416
H	3.748837	2.990182	5.378808
C	5.869956	3.124828	2.718714
H	5.947595	3.134963	1.639623
C	7.057441	3.163827	3.474648
H	8.012386	3.203905	2.970628
C	5.893362	3.092695	5.460561
H	5.920465	3.068308	6.540460
C	-0.132057	3.243246	3.310544
C	-1.367785	3.273827	2.615709
H	-1.414831	3.222717	1.535777
C	-2.576566	3.344489	3.332914
H	-3.518933	3.353448	2.803954
C	-1.468185	3.389205	5.356716
H	-1.528151	3.443253	6.434172
C	-0.210323	3.315856	4.724471
H	0.679962	3.305487	5.339974
C	-3.826006	13.774236	0.618920
H	-3.626527	14.818300	0.423889
C	-4.782889	11.797183	-0.081888
H	-5.347173	11.267275	-0.836469
C	-4.367933	11.124573	1.083768
H	-4.637499	10.082795	1.199040
C	-3.648601	11.822888	2.085921
C	-3.374748	13.187931	1.817659
H	-2.844014	13.810345	2.526630
C	-3.256554	11.197665	3.307752
C	-3.314787	9.406747	4.695664
C	-2.334027	11.336839	5.375869
N	-4.485769	5.360527	5.519946
C	-3.703886	8.060412	4.966744
C	-4.390302	7.267420	4.013790
H	-4.664995	7.658499	3.042629
C	-4.756133	5.944835	4.333573
H	-5.295101	5.347953	3.611335
C	-3.430269	7.437574	6.210111
H	-2.931015	7.965395	7.012480
C	-3.832354	6.106874	6.433946
H	-3.631227	5.641244	7.387448
C	-1.641318	12.081202	6.377128
C	-1.302420	11.512262	7.629522
H	-1.578768	10.498335	7.884832
C	-0.583243	12.268075	8.573576
H	-0.326393	11.826585	9.525251
C	-0.518847	14.097452	7.183259
H	-0.214443	15.122405	7.027502
C	-1.230005	13.420873	6.172666
H	-1.454314	13.947042	5.254395
C	3.344370	5.982231	-4.628996
H	4.288251	5.503869	-4.849071
C	1.039617	5.877602	-4.665032
H	0.150167	5.315951	-4.913648
C	0.923796	7.161496	-4.096639
H	-0.067614	7.563402	-3.933486
C	3.326066	7.270695	-4.059709
H	4.272421	7.759971	-3.868840
C	2.089251	7.907778	-3.786332
C	2.020854	9.237731	-3.270844
C	0.792440	11.077670	-2.771279
C	3.056771	11.168540	-2.680894
C	-0.471032	11.739967	-2.720689
C	-1.682544	11.068438	-3.020152
H	-1.698868	10.021680	-3.294039
C	-2.901700	11.772945	-2.997430
H	-3.821999	11.260845	-3.241174
C	-1.852327	13.734314	-2.397291

H	-1.934976	14.785652	-2.161208
C	-0.586727	13.115908	-2.400032
H	0.281495	13.720720	-2.172543
C	4.253916	11.931525	-2.528278
C	4.228794	13.301886	-2.165623
H	3.299605	13.821967	-1.972227
C	5.431597	14.029640	-2.081032
H	5.405548	15.077159	-1.817081
C	5.536339	11.376130	-2.768219
H	5.661936	10.343242	-3.066184
C	6.685588	12.184377	-2.660672
H	7.660674	11.762427	-2.860838
C	11.157417	14.567269	-0.812620
H	10.761786	13.559943	-0.968336
H	12.106616	14.621020	-1.360414
H	11.411402	14.659924	0.250833
C	9.794712	16.490550	-0.146580
H	8.717428	16.441118	0.037134
H	10.299581	16.240615	0.795016
H	10.033089	17.541503	-0.352373
C	10.675625	16.345814	-2.434135
H	10.735401	17.417388	-2.204379
H	11.711246	16.063528	-2.663132
C	9.887521	16.225415	-3.762254
H	9.652212	17.250804	-4.075044
H	10.601819	15.873358	-4.517486
C	7.449989	16.193385	-4.045924
H	6.795401	16.216412	-3.170611
H	7.681697	17.237255	-4.292199
H	6.862768	15.802462	-4.886394
C	8.783909	14.251521	-4.717572
H	7.960544	14.214920	-5.441798
H	9.708937	14.287285	-5.306453
H	8.789519	13.298932	-4.179743
N	-4.450497	1.141292	5.837438
N	-6.355154	3.178006	6.688240
C	-4.300073	0.594479	4.461928
H	-3.342631	0.081083	4.308891
H	-5.081236	-0.129483	4.197783
H	-4.362831	1.400670	3.726640
C	-3.282608	0.779678	6.683734
H	-2.515849	0.216740	6.136838
H	-2.811063	1.685483	7.075195
H	-3.553040	0.166786	7.552704
C	-5.724115	0.669990	6.456615
H	-5.523591	0.063364	7.349080
H	-6.259280	-0.012833	5.784456
C	-6.745891	1.751575	6.885728
H	-7.683224	1.523751	6.362107
H	-6.974922	1.555398	7.941109
C	-7.346581	3.885818	5.834368
H	-7.743547	4.794268	6.304447
H	-6.883572	4.183016	4.889157
H	-8.216696	3.267597	5.579937
C	-6.188001	3.870679	7.993035
H	-6.802712	4.775505	8.079359
H	-6.446043	3.239596	8.852648
H	-5.143882	4.168217	8.124301
N	9.471675	1.711510	6.178329
N	10.584994	4.309472	6.902743
N	10.162715	15.577736	-1.261727
N	8.659085	15.377932	-3.754435
N	-6.750371	14.762707	-1.700827
N	-5.171770	14.751955	-4.156481
N	5.678603	12.085039	4.214482
N	5.798812	10.156573	5.650366
N	7.093328	10.233647	3.621670
N	8.156553	5.686718	5.523209
N	2.613827	13.282203	8.030202
N	2.315101	2.806323	-3.610932
N	3.466469	3.094149	1.300586
N	1.061986	3.103418	1.272043
N	2.239586	3.097363	3.372492
N	-2.634551	3.392238	4.678492
N	7.081502	3.148614	4.823157
N	-4.526808	13.100594	-0.316510
N	-3.587994	9.895458	3.465651
N	-2.664535	10.060994	5.679696
N	-2.583102	11.957981	4.199636
N	-0.186830	13.540688	8.367546
N	2.225515	5.296036	-4.938096
N	0.788526	9.767495	-3.103266
N	1.887284	11.817818	-2.487195
N	3.193342	9.867029	-3.024125
N	6.643473	13.491581	-2.329789
N	-2.996191	13.084350	-2.695948
Pd	1.436202	14.301541	9.450245
Pd	-4.853946	13.927331	-2.216788

Pd	8.821780	3.718845	5.858281
Pd	8.398379	14.523994	-1.819310
Pd	2.327599	3.321164	-5.645365
Pd	-4.479928	3.271635	5.679133
C	2.624972	5.687886	5.799759
C	2.908409	5.978542	4.474198
C	1.868283	6.389938	3.593689
C	0.640578	6.824673	4.160226
C	0.533617	6.867246	5.580062
C	1.422638	6.143519	6.367664
H	2.961388	6.053422	1.768699
H	3.383028	5.247527	6.445054
H	3.901312	5.809398	4.055453
C	1.991646	6.325672	2.182614
C	-0.434960	7.125016	3.294154
H	-0.314464	7.374661	6.034161
H	1.262791	6.051719	7.443497
C	-0.333513	6.966821	1.926657
C	0.918878	6.567923	1.351475
H	-1.374606	7.454966	3.723732
C	-1.504901	7.289722	1.032613
H	-1.256494	8.108503	0.346653
H	-2.375737	7.599223	1.615293
C	1.056059	6.451393	-0.144600
H	0.304563	5.778978	-0.567225
H	2.049283	6.086124	-0.414387
C	3.300383	8.948567	3.755053
C	3.302689	8.355590	5.110694
C	2.243656	8.766746	5.775124
C	1.506253	9.728152	4.928605
H	4.218668	7.814619	5.479824
H	1.992119	8.670242	6.844806
C	1.872277	10.671648	2.591062
C	0.490247	10.331912	2.019444
C	1.991664	12.154332	2.976763
H	2.617177	10.464874	1.816998
C	0.161646	11.230316	0.826599
H	-0.260089	10.444761	2.808657
H	0.496999	9.286193	1.714902
C	1.662925	13.037586	1.768509
H	1.298021	12.367775	3.801440
H	3.006392	12.354254	3.339963
C	0.271680	12.712673	1.208848
H	-0.838747	10.995918	0.443728
H	0.882262	11.001577	0.027386
H	1.726624	14.093572	2.042456
H	2.413485	12.868249	0.982922
H	0.057002	13.357857	0.347453
H	-0.480439	12.947371	1.974170
N	2.207984	9.789974	3.706296
O	0.551855	10.428124	5.212830
O	4.119547	8.806628	2.836758
O	5.446704	6.324049	2.624030
H	5.718107	6.193262	1.707803
H	5.118715	7.243658	2.664389
O	2.977860	8.930136	0.083140
H	3.385973	8.765317	0.951393
H	3.717303	8.949414	-0.531987
H	-1.795503	6.441055	0.408037
H	0.938292	7.432624	-0.614388

Table5_1b_TSiii_TSi-ii_h2o_2

Property	Value
Charge	0
Electronic Energy, BS1 (a.u.)	-1211.775935
Thermal and entropic correction, BS1 (a.u.)	2.794825
Electronic Energy, BS2 (a.u.)	-1212.202233
Number of Imaginary Frequencies	0
Imaginary frequencies (cm-1)	None

Molecular Geometry in Cartesian Coordinates

C	8.409098	1.027597	6.966042
H	8.778778	0.592755	7.903126
H	7.627482	1.742310	7.238592
H	7.928806	0.208918	6.415621
C	9.626932	1.081089	4.840537
H	8.922655	0.256999	4.670559
H	9.457933	1.823258	4.055291
H	10.629486	0.668165	4.672761
C	10.764633	1.717034	6.920535
H	11.543262	1.191777	6.352729
H	10.683288	1.145015	7.853690

C	11.357676	3.094975	7.303172
H	12.372738	3.121665	6.886605
H	11.515522	3.072367	8.389036
C	11.379819	5.161276	5.979532
H	10.906015	5.188095	4.994403
H	11.473161	6.197396	6.328216
H	12.402819	4.795686	5.825981
C	10.153018	5.079723	8.098482
H	9.062930	5.061005	8.179684
H	10.545562	4.674604	9.039586
H	10.463247	6.131532	8.063466
N	3.101670	15.072253	10.543564
N	0.213045	15.330934	10.863537
C	3.867423	13.905890	11.057048
H	3.332973	12.979814	10.828654
H	4.870206	13.826348	10.618957
H	4.008131	13.926721	12.145005
C	3.921379	15.870809	9.593716
H	3.413373	15.942442	8.628361
H	4.095232	16.899614	9.933284
H	4.912331	15.435540	9.414088
C	2.610821	15.923278	11.667057
H	2.993186	15.559269	12.629317
H	3.009541	16.942886	11.589995
C	1.077838	16.065002	11.833049
H	0.861303	17.140859	11.814764
H	0.849897	15.764293	12.863744
C	-0.642938	16.273792	10.094781
H	-1.711020	16.031253	10.159955
H	-0.543837	17.315099	10.425904
H	-0.366797	16.253184	9.036509
C	-0.613608	14.306473	11.555539
H	-0.319963	13.305399	11.226898
H	-0.500335	14.325893	12.646595
H	-1.686945	14.416577	11.355556
N	2.416422	1.302558	-6.323177
N	2.329524	3.872487	-7.704742
C	3.667238	0.710321	-5.778695
H	3.479957	-0.101496	-5.064685
H	4.320527	0.292662	-6.554949
H	4.248607	1.477002	-5.258780
C	1.218936	0.607080	-5.781107
H	1.472567	-0.194100	-5.075618
H	0.581158	1.320407	-5.251170
H	0.598245	0.145954	-6.559511
C	2.419455	1.278244	-7.815298
H	1.570741	0.697816	-8.199206
H	3.300803	0.745639	-8.195030
C	2.385831	2.642358	-8.548354
H	3.261608	2.660893	-9.209608
H	1.532252	2.601355	-9.237092
C	1.077344	4.633105	-7.959228
H	1.261092	5.680528	-8.229200
H	0.471549	4.209058	-8.769780
H	0.449415	4.629497	-7.064198
C	3.525821	4.726831	-7.929067
H	3.269535	5.753422	-8.219222
H	4.121784	4.783113	-7.013882
H	4.189585	4.342576	-8.713631
C	-6.520729	15.733337	-0.598596
H	-6.996630	15.432814	0.343369
H	-5.448752	15.832302	-0.407909
H	-6.894195	16.740094	-0.824559
C	-7.616254	13.639989	-1.247442
H	-7.905745	13.722920	-0.192418
H	-8.550984	13.558382	-1.816152
H	-7.090832	12.688766	-1.369177
C	-7.338230	15.438408	-2.894412
H	-7.566807	16.489057	-2.674207
H	-8.311288	15.002013	-3.153800
C	-6.497017	15.439238	-4.194861
H	-7.136739	15.011632	-4.977507
H	-6.383077	16.490679	-4.488508
C	-5.129776	13.624964	-5.125375
H	-4.289573	13.696116	-5.827311
H	-5.030563	12.675611	-4.591744
H	-6.036409	13.548469	-5.738486
C	-4.066220	15.713164	-4.411538
H	-3.415922	15.406071	-5.240175
H	-4.419549	16.722557	-4.656399
H	-3.439007	15.806733	-3.520470
N	8.101352	13.690809	0.080624
C	7.179559	14.215951	0.914502
H	6.858183	15.226980	0.707518
C	6.645710	13.524103	2.019834
H	5.922103	14.030785	2.644847
C	7.083571	12.207350	2.308543
C	8.053829	11.669781	1.427220

H	8.467350	10.679918	1.572009
C	8.520354	12.435307	0.341722
H	9.262759	12.019068	-0.324057
C	6.600049	11.476512	3.434937
C	5.348488	11.390195	5.325360
C	4.417810	12.006782	6.216628
C	3.901416	13.305947	5.976561
H	4.185624	13.880218	5.104405
C	3.016875	13.895286	6.899612
H	2.633124	14.889835	6.721790
C	3.077200	12.036997	8.266451
H	2.737258	11.553252	9.171224
C	3.969078	11.369426	7.401608
H	4.310022	10.379325	7.675034
C	6.676995	9.636043	4.759324
C	7.174327	8.322662	5.019759
C	6.842685	7.610124	6.200243
H	6.219368	8.048081	6.968494
C	8.030086	7.651731	4.109302
H	8.344579	8.110553	3.181153
C	8.484943	6.350100	4.396902
H	9.127779	5.837216	3.696205
C	7.348320	6.310560	6.404665
H	7.095989	5.767918	7.304038
C	3.457244	2.859039	-2.894287
H	4.383430	2.826856	-3.450289
C	3.492972	2.937020	-1.488704
H	4.458238	2.953407	-0.999408
C	1.156650	2.837470	-2.917006
H	0.243944	2.784149	-3.493501
C	1.090799	2.920408	-1.512649
H	0.116385	2.920804	-1.042047
C	2.284327	2.974053	-0.750584
C	2.270108	3.045837	0.674515
C	1.113542	3.139899	2.623425
C	3.382639	3.075328	2.651246
C	4.613056	3.082740	3.375189
C	4.653652	3.054557	4.791474
H	3.748064	2.988864	5.379973
C	5.869146	3.126033	2.719703
H	5.946794	3.135463	1.640606
C	7.056671	3.164953	3.475581
H	8.011615	3.204354	2.971521
C	5.892634	3.095234	5.461570
H	5.919769	3.071377	6.541479
C	-0.133024	3.242347	3.311691
C	-1.368759	3.272524	2.616820
H	-1.415741	3.222645	1.536828
C	-2.577646	3.340859	3.334045
H	-3.520023	3.349262	2.805094
C	-1.469356	3.384190	5.358015
H	-1.529462	3.436596	6.435546
C	-0.211394	3.312946	4.725732
H	0.678842	3.302366	5.341324
C	-3.827072	13.774108	0.619479
H	-3.628394	14.818431	0.425011
C	-4.782879	11.796833	-0.082087
H	-5.347117	11.266968	-0.836733
C	-4.367014	11.123826	1.083022
H	-4.635801	10.081795	1.197825
C	-3.647678	11.822013	2.085245
C	-3.374973	13.187433	1.817716
H	-2.844509	13.809876	2.526886
C	-3.254468	11.196280	3.306439
C	-3.311079	9.404772	4.693624
C	-2.330822	11.335021	5.374087
N	-4.483409	5.359238	5.519032
C	-3.700429	8.058550	4.964931
C	-4.387300	7.265589	4.012263
H	-4.661974	7.656537	3.041035
C	-4.753664	5.943241	4.332473
H	-5.292948	5.346357	3.610459
C	-3.426728	7.435869	6.208375
H	-2.927095	7.963667	7.010522
C	-3.829350	6.105424	6.432684
H	-3.628118	5.639908	7.386226
C	-1.638760	12.079593	6.375612
C	-1.299458	11.510501	7.627826
H	-1.574725	10.496178	7.882706
C	-0.581254	12.266699	8.572293
H	-0.324093	11.825101	9.523835
C	-0.518453	14.096646	7.182662
H	-0.215026	15.121945	7.027284
C	-1.228742	13.419739	6.171694
H	-1.453417	13.946038	5.253589
C	3.344114	5.983546	-4.629359
H	4.287965	5.505152	-4.849474
C	1.039365	5.878980	-4.665186

H	0.149906	5.317275	-4.913644
C	0.923617	7.162953	-4.096976
H	-0.067769	7.564932	-3.933855
C	3.325892	7.272068	-4.060199
H	4.272269	7.761348	-3.869455
C	2.089113	7.909223	-3.786811
C	2.020752	9.239188	-3.271354
C	0.792294	11.078871	-2.770913
C	3.056615	11.169868	-2.680886
C	-0.471260	11.741024	-2.720184
C	-1.682651	11.069445	-3.020048
H	-1.698796	10.022791	-3.294332
C	-2.901904	11.773763	-2.997250
H	-3.822083	11.261612	-3.241334
C	-1.852884	13.735069	-2.396285
H	-1.935698	14.786327	-2.159891
C	-0.587189	13.116833	-2.399050
H	0.280918	13.721671	-2.171203
C	4.253786	11.932794	-2.528163
C	4.228729	13.302947	-2.164736
H	3.299567	13.822872	-1.970784
C	5.431545	14.030691	-2.080170
H	5.405576	15.078101	-1.815781
C	5.536161	11.377562	-2.768808
H	5.661711	10.344870	-3.067466
C	6.685424	12.185763	-2.661146
H	7.660467	11.763973	-2.861868
C	11.157487	14.567881	-0.812315
H	10.761890	13.560592	-0.968420
H	12.106723	14.621905	-1.360021
H	11.411377	14.660158	0.251191
C	9.794756	16.490926	-0.145664
H	8.717505	16.441310	0.038206
H	10.299802	16.240820	0.795793
H	10.032941	17.541968	-0.351217
C	10.675584	16.346878	-2.433309
H	10.735480	17.418372	-2.203201
H	11.711148	16.064559	-2.662527
C	9.887302	16.226989	-3.761376
H	9.651618	17.252472	-4.073575
H	10.601638	15.875635	-4.516899
C	7.449822	16.194227	-4.045473
H	6.795107	16.216961	-3.170247
H	7.681269	17.238186	-4.291625
H	6.862824	15.803192	-4.886049
C	8.784431	14.252870	-4.717200
H	7.961543	14.216543	-5.441977
H	9.709839	14.288578	-5.305476
H	8.789580	13.300160	-4.179583
N	-4.455638	1.140172	5.839598
N	-6.356697	3.180828	6.689144
C	-4.306395	0.592319	4.464360
H	-3.350415	0.076055	4.311830
H	-5.089564	-0.129499	4.200300
H	-4.366546	1.398360	3.728683
C	-3.288253	0.776916	6.685885
H	-2.522388	0.212710	6.139042
H	-2.815276	1.682093	7.077076
H	-3.559550	0.164614	7.554998
C	-5.730003	0.671562	6.459285
H	-5.530433	0.065398	7.352279
H	-6.266328	-0.010979	5.787767
C	-6.749952	1.755216	6.887539
H	-7.687610	1.528656	6.363949
H	-6.979450	1.560167	7.943029
C	-7.346870	3.889797	5.834781
H	-7.742360	4.799177	6.304292
H	-6.883327	4.185638	4.889415
H	-8.218007	3.272887	5.580671
C	-6.188399	3.874063	7.993496
H	-6.801520	4.780029	8.079189
H	-6.447628	3.243997	8.853498
H	-5.143751	4.169824	8.124646
N	9.469907	1.711004	6.178892
N	10.585630	4.307954	6.903318
N	10.162755	15.578450	-1.261101
N	8.659122	15.379112	-3.753910
N	-6.751563	14.762118	-1.700734
N	-5.172268	14.752402	-4.156000
N	5.678243	12.084546	4.213902
N	5.799267	10.156558	5.650284
N	7.093821	10.233627	3.621643
N	8.158201	5.687456	5.524563
N	2.614511	13.282315	8.030143
N	2.314232	2.808281	-3.609877
N	3.465623	3.096033	1.301635
N	1.061146	3.105852	1.273061
N	2.238712	3.103290	3.373527

N	-2.635642	3.386852	4.679666
N	7.080770	3.150325	4.824092
N	-4.527761	13.100548	-0.316100
N	-3.584987	9.893758	3.463863
N	-2.660369	10.058839	5.677465
N	-2.580889	11.956535	4.198275
N	-0.186101	13.539789	8.366815
N	2.225213	5.297353	-4.938288
N	0.788426	9.768811	-3.103344
N	1.887108	11.818936	-2.486482
N	3.193242	9.868605	-3.025029
N	6.643361	13.492779	-2.329554
N	-2.996623	13.085048	-2.695333
Pd	1.436289	14.301488	9.449691
Pd	-4.854632	13.927594	-2.216300
Pd	8.821759	3.718948	5.859070
Pd	8.398424	14.524850	-1.818954
Pd	2.327110	3.322150	-5.644589
Pd	-4.481237	3.270424	5.680087
C	2.622454	5.696078	5.817394
C	2.960526	6.178340	4.511304
C	1.867305	6.403936	3.583074
C	0.646511	6.827741	4.149303
C	0.634842	7.001679	5.587426
C	1.445802	6.121874	6.370973
H	2.968153	6.059995	1.772784
H	3.356119	5.163271	6.412121
H	3.920121	5.914976	4.077583
C	1.997269	6.319197	2.186069
C	-0.433613	7.108273	3.296180
H	-0.247341	7.450058	6.037031
H	1.212569	5.941144	7.414375
C	-0.329775	6.958658	1.919309
C	0.915218	6.564239	1.349562
H	-1.370350	7.447817	3.725493
C	-1.500913	7.284097	1.029019
H	-1.253613	8.103645	0.343625
H	-2.373065	7.591360	1.610906
C	1.063344	6.454551	-0.146493
H	0.314445	5.779726	-0.574804
H	2.058072	6.093402	-0.416173
C	3.296358	8.923788	3.744576
C	3.263332	8.181550	5.043853
C	2.124724	8.645821	5.745451
C	1.502436	9.713904	4.933881
H	4.205093	7.953926	5.518110
H	2.041051	8.703202	6.819775
C	1.875144	10.670379	2.593415
C	0.491163	10.326502	2.026126
C	1.991417	12.153125	2.978431
H	2.617512	10.458506	1.821836
C	0.159683	11.229364	0.833752
H	-0.256578	10.445215	2.817657
H	0.491253	9.280568	1.722350
C	1.659640	13.036283	1.766058
H	1.300195	12.367041	3.800157
H	3.007231	12.357567	3.333942
C	0.266732	12.711830	1.210269
H	-0.841874	10.990531	0.453666
H	0.873099	10.999987	0.032608
H	1.724449	14.092290	2.039664
H	2.407963	12.866560	0.978422
H	0.050054	13.351915	0.349201
H	-0.483240	12.947160	1.977474
N	2.218866	9.783999	3.717337
O	0.563352	10.433693	5.215297
O	4.119027	8.809737	2.830078
O	5.453135	6.326281	2.624134
H	5.722763	6.191927	1.707883
H	5.128159	7.245365	2.656130
O	2.980429	8.933494	0.078617
H	3.392964	8.763606	0.942212
H	3.720213	8.952871	-0.540947
H	-1.793685	6.434888	0.403915
H	0.942094	7.435723	-0.615476

Table5_1b_TSiii_DG_h2o_1wat

Property	Value
Charge	0
Electronic Energy, BS1 (a.u.)	-1211.819829
Thermal and entropic correction, BS1 (a.u.)	2.804012
Electronic Energy, BS2 (a.u.)	-1212.243711

Number of Imaginary Frequencies	0
Imaginary frequencies (cm-1)	None

Molecular Geometry in Cartesian Coordinates

C	8.411921	1.027625	6.966465
H	8.782614	0.593506	7.903488
H	7.629942	1.741877	7.239207
H	7.931910	0.208300	6.416770
C	9.628227	1.081205	4.840096
H	8.923933	0.257025	4.670629
H	9.458852	1.823092	4.054658
H	10.630737	0.668250	4.672113
C	10.766929	1.718749	6.919019
H	11.545893	1.194966	6.350313
H	10.686914	1.145978	7.851830
C	11.358327	3.097219	7.302288
H	12.373418	3.125233	6.885875
H	11.516051	3.074403	8.388164
C	11.378314	5.163861	5.979202
H	10.904329	5.190775	4.994189
H	11.470951	6.199878	6.328377
H	12.401554	4.799119	5.825238
C	10.151578	5.080455	8.098111
H	9.061527	5.060305	8.179452
H	10.544817	4.675680	9.039075
H	10.460425	6.132671	8.063210
N	3.102113	15.071578	10.543915
N	0.213656	15.331234	10.864523
C	3.867517	13.904846	11.057080
H	3.332720	12.978997	10.828603
H	4.870221	13.825045	10.618857
H	4.008378	13.925436	12.145024
C	3.921952	15.869982	9.594062
H	3.413729	15.942117	8.628871
H	4.096369	16.898617	9.933860
H	4.912652	15.434290	9.414067
C	2.611796	15.922598	11.667640
H	2.993952	15.558134	12.629811
H	3.011138	16.941981	11.590818
C	1.078902	16.065260	11.833651
H	0.862943	17.141228	11.814927
H	0.850878	15.765042	12.864471
C	-0.642497	16.274133	10.096013
H	-1.710552	16.031518	10.161303
H	-0.543438	17.315390	10.427308
H	-0.366514	16.253645	9.037685
C	-0.612886	14.306946	11.556901
H	-0.319354	13.305805	11.228366
H	-0.499343	14.326586	12.647927
H	-1.686267	14.417055	11.357167
N	2.416789	1.301875	-6.324937
N	2.329560	3.872458	-7.705287
C	3.667258	0.709156	-5.780186
H	3.479501	-0.102847	-5.066510
H	4.320752	0.291591	-6.556322
H	4.248595	1.475525	-5.259780
C	1.218951	0.606333	-5.783732
H	1.472155	-0.195519	-5.078851
H	0.581282	1.319421	-5.253354
H	0.598277	0.146086	-6.562669
C	2.420494	1.278297	-7.817069
H	1.572258	0.697597	-8.201613
H	3.302269	0.746331	-8.196702
C	2.386415	2.642748	-8.549476
H	3.262249	2.661926	-9.210635
H	1.532927	2.601748	-9.238327
C	1.076978	4.632585	-7.959276
H	1.260198	5.680156	-8.229040
H	0.471158	4.208471	-8.769774
H	0.449271	4.628479	-7.064095
C	3.525425	4.727479	-7.929359
H	3.268599	5.754120	-8.218851
H	4.121583	4.783482	-7.014287
H	4.189188	4.344002	-8.714305
C	-6.518695	15.734352	-0.599248
H	-6.994077	15.434317	0.343132
H	-5.446569	15.833149	-0.409255
H	-6.892119	16.741081	-0.825414
C	-7.615228	13.641082	-1.246609
H	-7.904095	13.724525	-0.191455
H	-8.550321	13.559630	-1.814746
H	-7.090218	12.689618	-1.368267
C	-7.337330	15.438600	-2.894593
H	-7.565656	16.489384	-2.674781
H	-8.310522	15.002227	-3.153507
C	-6.496524	15.438771	-4.195306
H	-7.136482	15.010837	-4.977578

H	-6.382628	16.490078	-4.489450
C	-5.129349	13.624415	-5.125743
H	-4.289205	13.695525	-5.827753
H	-5.030104	12.675098	-4.592057
H	-6.036026	13.547879	-5.738785
C	-4.065753	15.712684	-4.412200
H	-3.415367	15.405402	-5.240700
H	-4.419124	16.721996	-4.657337
H	-3.438648	15.806474	-3.521065
N	8.101363	13.690459	0.080530
C	7.179647	14.215798	0.914394
H	6.858267	15.226794	0.707236
C	6.645850	13.524184	2.019913
H	5.922315	14.030982	2.644913
C	7.083571	12.207421	2.308757
C	8.053714	11.669635	1.427433
H	8.467168	10.679757	1.572298
C	8.520279	12.434970	0.341826
H	9.262617	12.018545	-0.323909
C	6.600000	11.476730	3.435228
C	5.348650	11.390537	5.325799
C	4.417916	12.007070	6.217080
C	3.901766	13.306377	5.977251
H	4.186405	13.880915	5.105407
C	3.016754	13.895450	6.900017
H	2.633083	14.890041	6.722233
C	3.076494	12.036848	8.266421
H	2.736203	11.552917	9.170967
C	3.968747	11.369472	7.401777
H	4.309713	10.379372	7.675190
C	6.676134	9.635833	4.759090
C	7.172923	8.322133	5.018986
C	6.841582	7.609528	6.199500
H	6.218735	8.047561	6.968040
C	8.028006	7.651014	4.108019
H	8.342207	8.109834	3.179770
C	8.482777	6.349306	4.395365
H	9.125172	5.836300	3.694346
C	7.347104	6.309875	6.403676
H	7.095014	5.767248	7.303129
C	3.458120	2.857531	-2.895365
H	4.384297	2.825879	-3.451422
C	3.493873	2.935327	-1.489763
H	4.459144	2.952177	-1.000483
C	1.157552	2.834797	-2.917995
H	0.244844	2.781090	-3.494443
C	1.091713	2.917483	-1.513634
H	0.117325	2.917208	-1.043001
C	2.285224	2.971693	-0.751601
C	2.270963	3.043506	0.673498
C	1.114407	3.138867	2.622373
C	3.383485	3.073666	2.650193
C	4.613873	3.080896	3.374154
C	4.654422	3.051930	4.790416
H	3.748837	2.980324	5.378808
C	5.869956	3.124828	2.718714
H	5.947595	3.134963	1.639623
C	7.057441	3.163827	3.474648
H	8.012386	3.203905	2.970628
C	5.893362	3.092695	5.460561
H	5.920465	3.068308	6.540460
C	-0.132057	3.243246	3.310544
C	-1.367785	3.273827	2.615709
H	-1.414831	3.222717	1.535777
C	-2.576566	3.344489	3.332914
H	-3.518933	3.353448	2.803954
C	-1.468185	3.389205	5.356716
H	-1.528151	3.443253	6.434172
C	-0.210323	3.315856	4.724471
H	0.679962	3.305487	5.339974
C	-3.826006	13.774236	0.618920
H	-3.626527	14.818300	0.423889
C	-4.782889	11.797183	-0.081888
H	-5.347173	11.267275	-0.836469
C	-4.367933	11.124573	1.083768
H	-4.637499	10.082795	1.199040
C	-3.648601	11.822888	2.085921
C	-3.374748	13.187931	1.817659
H	-2.844014	13.810345	2.526630
C	-3.256554	11.197665	3.307752
C	-3.314787	9.406747	4.695664
C	-2.334027	11.336839	5.375869
N	-4.485769	5.360527	5.519946
C	-3.703886	8.060412	4.966744
C	-4.390302	7.267420	4.013790
H	-4.664995	7.658499	3.042629
C	-4.756133	5.944835	4.333573
H	-5.295101	5.347953	3.611335

C	-3.430269	7.437574	6.210111
H	-2.931015	7.965395	7.012480
C	-3.832354	6.106874	6.433946
H	-3.631227	5.641244	7.387448
C	-1.641318	12.081202	6.377128
C	-1.302420	11.512262	7.629522
H	-1.578768	10.498335	7.884832
C	-0.583243	12.268075	8.573576
H	-0.326393	11.826585	9.525251
C	-0.518847	14.097452	7.183259
H	-0.214443	15.122405	7.027502
C	-1.230005	13.420873	6.172666
H	-1.454314	13.947042	5.254395
C	3.344370	5.982231	-4.628996
H	4.288251	5.503869	-4.849071
C	1.039617	5.877602	-4.665032
H	0.150167	5.315951	-4.913648
C	0.923796	7.161496	-4.096639
H	-0.067614	7.563402	-3.933486
C	3.326066	7.270695	-4.059709
H	4.272421	7.759971	-3.868840
C	2.089251	7.907778	-3.786332
C	2.020854	9.237731	-3.270844
C	0.792440	11.077670	-2.771279
C	3.056771	11.168540	-2.680894
C	-0.471032	11.739967	-2.720689
C	-1.682544	11.068438	-3.020152
H	-1.698868	10.021680	-3.294039
C	-2.901700	11.772945	-2.997430
H	-3.821999	11.260845	-3.241174
C	-1.852327	13.734314	-2.397291
H	-1.934976	14.785652	-2.161208
C	-0.586727	13.115908	-2.400032
H	0.281495	13.720720	-2.172543
C	4.253916	11.931525	-2.528278
C	4.228794	13.301886	-2.165623
H	3.299605	13.821967	-1.972227
C	5.431597	14.029640	-2.081032
H	5.405548	15.077159	-1.817081
C	5.536339	11.376130	-2.768219
H	5.661936	10.343242	-3.066184
C	6.685588	12.184377	-2.660672
H	7.660674	11.762427	-2.860838
C	11.157417	14.567269	-0.812620
H	10.761786	13.559943	-0.968336
H	12.106616	14.621020	-1.360414
H	11.411402	14.659924	0.250833
C	9.794712	16.490550	-0.146580
H	8.717428	16.441118	0.037134
H	10.299581	16.240615	0.795016
H	10.033089	17.541503	-0.352373
C	10.675625	16.345814	-2.434135
H	10.735401	17.417388	-2.204379
H	11.711246	16.063528	-2.663132
C	9.887521	16.225415	-3.762254
H	9.652212	17.250804	-4.075044
H	10.601819	15.873358	-4.517486
C	7.449989	16.193385	-4.045924
H	6.795401	16.216412	-3.170611
H	7.681697	17.237255	-4.292199
H	6.862768	15.802462	-4.886394
C	8.783909	14.251521	-4.717572
H	7.960544	14.214920	-5.441798
H	9.708937	14.287285	-5.306453
H	8.789519	13.298932	-4.179743
N	-4.450497	1.141292	5.837438
N	-6.355154	3.178006	6.688240
C	-4.300073	0.594479	4.461928
H	-3.342631	0.081083	4.308891
H	-5.081236	-0.129483	4.197783
H	-4.362831	1.400670	3.726640
C	-3.282608	0.779678	6.683734
H	-2.515849	0.216740	6.136838
H	-2.811063	1.685483	7.075195
H	-3.553040	0.166786	7.552704
C	-5.724115	0.669990	6.456615
H	-5.523591	0.063364	7.349080
H	-6.259280	-0.012833	5.784456
C	-6.745891	1.751575	6.885728
H	-7.683224	1.523751	6.362107
H	-6.974922	1.555398	7.941109
C	-7.346581	3.885818	5.834368
H	-7.743547	4.794268	6.304447
H	-6.883572	4.183016	4.889157
H	-8.216696	3.267597	5.579937
C	-6.188001	3.870679	7.993035
H	-6.802712	4.775505	8.079359
H	-6.446043	3.239596	8.852648

H	-5.143882	4.168217	8.124301
N	9.471675	1.711510	6.178329
N	10.584994	4.309472	6.902743
N	10.162715	15.577736	-1.261727
N	8.659085	15.377932	-3.754435
N	-6.750371	14.762707	-1.700827
N	-5.171770	14.751955	-4.156481
N	5.678603	12.085039	4.214482
N	5.798812	10.156573	5.650366
N	7.093328	10.233647	3.621670
N	8.156553	5.686718	5.523209
N	2.613827	13.282203	8.030202
N	2.315101	2.806323	-3.610932
N	3.466469	3.094149	1.300586
N	1.061986	3.103418	1.272043
N	2.239586	3.107221	3.372492
N	-2.634551	3.392238	4.678492
N	7.081502	3.148614	4.823157
N	-4.526808	13.100594	-0.316510
N	-3.587994	9.895458	3.465651
N	-2.664535	10.060994	5.679696
N	-2.583102	11.957981	4.199636
N	-0.186830	13.540688	8.367546
N	2.225515	5.296036	-4.938096
N	0.788526	9.767495	-3.103266
N	1.887284	11.817818	-2.487195
N	3.193342	9.867029	-3.024125
N	6.643473	13.491581	-2.329789
N	-2.996191	13.084350	-2.695948
Pd	1.436202	14.301541	9.450245
Pd	-4.853946	13.927331	-2.216788
Pd	8.821780	3.718845	5.858281
Pd	8.398379	14.523994	-1.819310
Pd	2.327599	3.321164	-5.645365
Pd	-4.479928	3.271635	5.679133
C	2.605255	5.697744	5.839192
C	3.006993	6.382735	4.553065
C	1.858425	6.419513	3.573972
C	0.650436	6.844390	4.140509
C	0.750501	7.172855	5.599779
C	1.462071	6.104086	6.377522
H	2.961388	6.053422	1.778557
H	3.304161	5.050360	6.376046
H	3.930887	6.016423	4.104744
C	1.991646	6.305955	2.192472
C	-0.434960	7.105299	3.304012
H	-0.166589	7.561970	6.044019
H	1.144491	5.824977	7.384347
C	-0.333513	6.956963	1.916799
C	0.899161	6.558065	1.351475
H	-1.364748	7.464824	3.733590
C	-1.504901	7.289722	1.032613
H	-1.256494	8.108503	0.346653
H	-2.375737	7.599223	1.615293
C	1.056059	6.451393	-0.144600
H	0.304563	5.778978	-0.577083
H	2.049283	6.086124	-0.414387
C	3.290525	8.899276	3.745195
C	3.213964	8.000689	4.982536
C	1.977480	8.510428	5.715974
C	1.486536	9.698577	4.938463
H	4.179235	8.110370	5.578408
H	2.080844	8.749109	6.795515
C	1.882135	10.671648	2.600920
C	0.500105	10.322054	2.029302
C	1.991664	12.154332	2.986621
H	2.627035	10.455016	1.836715
C	0.171504	11.230316	0.836457
H	-0.250231	10.444761	2.818515
H	0.496999	9.276335	1.724760
C	1.662925	13.037586	1.768509
H	1.298021	12.367775	3.801440
H	3.006392	12.364112	3.339963
C	0.271680	12.712673	1.208848
H	-0.828889	10.986060	0.453586
H	0.882262	11.001577	0.037244
H	1.726624	14.093572	2.042456
H	2.413485	12.868249	0.982922
H	0.057002	13.347999	0.347453
H	-0.480439	12.947371	1.974170
N	2.227701	9.780116	3.735871
O	0.561713	10.437982	5.212830
O	4.119547	8.816486	2.836758
O	5.446704	6.324049	2.624030
H	5.718107	6.193262	1.707803
H	5.128573	7.243658	2.654531
O	2.977860	8.930136	0.083140
H	3.395831	8.755459	0.941535

H	3.717303	8.949414	-0.541845
H	-1.805361	6.441055	0.408037
H	0.938292	7.432624	-0.614388

Table5_1c_TSi_reactant_1wat

Property	Value
Charge	0
Electronic Energy, BS1 (a.u.)	-1214.008709
Thermal and entropic correction, BS1 (a.u.)	2.719108
Electronic Energy, BS2 (a.u.)	-1214.425633
Number of Imaginary Frequencies	0
Imaginary frequencies (cm-1)	None

Molecular Geometry in Cartesian Coordinates

C	8.345269	0.904705	6.788819
H	8.700294	0.420650	7.707279
H	7.596560	1.641499	7.092110
H	7.828012	0.127842	6.211943
C	9.559507	0.979408	4.661707
H	8.825151	0.187221	4.468716
H	9.414796	1.752159	3.901550
H	10.545924	0.536475	4.475682
C	10.725532	1.500845	6.757859
H	11.481587	0.963837	6.170942
H	10.623168	0.902150	7.672047
C	11.373401	2.840787	7.183936
H	12.387782	2.840752	6.764845
H	11.532678	2.776323	8.267911
C	11.475937	4.946913	5.929542
H	11.003925	5.020659	4.946008
H	11.606952	5.967843	6.309430
H	12.484802	4.548479	5.764596
C	10.249682	4.844556	8.047556
H	9.159766	4.870600	8.127957
H	10.623297	4.390823	8.973971
H	10.605395	5.882519	8.048376
N	3.194242	15.029456	10.600111
N	0.301863	15.230588	10.932764
C	3.970169	13.890660	11.158363
H	3.446070	12.951448	10.963224
H	4.975813	13.806139	10.727743
H	4.105833	13.952611	12.245354
C	4.009638	15.803213	9.626023
H	3.504086	15.838937	8.657351
H	4.173515	16.844355	9.931501
H	5.005087	15.371250	9.463820
C	2.690334	15.914762	11.690841
H	3.093604	15.603968	12.663198
H	3.057639	16.941398	11.565686
C	1.155864	16.016373	11.870907
H	0.908137	17.084684	11.825651
H	0.948150	15.738230	12.912122
C	-0.595384	16.127124	10.155573
H	-1.653368	15.848183	10.237536
H	-0.530332	17.177475	10.466036
H	-0.329957	16.096906	9.095018
C	-0.480986	14.197513	11.661473
H	-0.164240	13.198950	11.347140
H	-0.347461	14.243187	12.749519
H	-1.560696	14.272416	11.480502
N	2.393817	1.236358	-6.133249
N	2.375161	3.531055	-7.934945
C	3.623493	0.722294	-5.474500
H	3.409131	0.062391	-4.624597
H	4.269916	0.151025	-6.152429
H	4.222383	1.557272	-5.100931
C	1.173922	0.667151	-5.501557
H	1.399745	0.004937	-4.656460
H	0.537149	1.473505	-5.126145
H	0.561833	0.079505	-6.197131
C	2.417593	0.955606	-7.598580
H	1.560046	0.337168	-7.893096
H	3.291032	0.346792	-7.865238
C	2.429512	2.174216	-8.554276
H	3.321621	2.066715	-9.184550
H	1.592603	2.028729	-9.249236
C	1.136288	4.248890	-8.336947
H	1.336576	5.233277	-8.778186
H	0.537541	3.698322	-9.073239
H	0.495078	4.403926	-7.465048
C	3.584744	4.323493	-8.281916
H	3.344836	5.292075	-8.738225

H	4.171255	4.522205	-7.380300
H	4.251406	3.808834	-8.985050
C	-6.377639	15.486353	-0.151856
H	-6.731028	15.119331	0.819876
H	-5.293182	15.608858	-0.083866
H	-6.799501	16.492113	-0.272795
C	-7.499387	13.403528	-0.792275
H	-7.639555	13.403142	0.295903
H	-8.503545	13.347856	-1.230934
H	-6.989740	12.473770	-1.059587
C	-7.449546	15.296867	-2.351334
H	-7.669354	16.328509	-2.047985
H	-8.438251	14.851417	-2.520610
C	-6.767280	15.386356	-3.738587
H	-7.485921	14.983108	-4.463541
H	-6.709659	16.454134	-3.985917
C	-5.484045	13.657579	-4.917506
H	-4.732919	13.784635	-5.707087
H	-5.306303	12.685550	-4.448915
H	-6.454410	13.589520	-5.424998
C	-4.385443	15.734348	-4.222387
H	-3.824748	15.482054	-5.131254
H	-4.786497	16.743428	-4.379161
H	-3.666088	15.805883	-3.401507
N	7.978414	13.565884	0.001377
C	7.106619	14.107877	0.876328
H	6.741172	15.099360	0.650107
C	6.676668	13.450512	2.045890
H	5.986983	13.965620	2.701583
C	7.169505	12.156562	2.352851
C	8.076687	11.598460	1.417140
H	8.508963	10.616349	1.561438
C	8.443490	12.328733	0.270002
H	9.135005	11.899595	-0.440842
C	6.784966	11.461464	3.539117
C	5.595477	11.385869	5.472147
C	4.638174	11.981368	6.350586
C	4.038344	13.235364	6.066362
H	4.286066	13.795615	5.174165
C	3.105725	13.792531	6.962871
H	2.654353	14.750951	6.746944
C	3.286572	11.992693	8.393138
H	2.976222	11.518572	9.312352
C	4.228704	11.358815	7.556950
H	4.635243	10.404399	7.865336
C	6.912884	9.633487	4.878614
C	7.385889	8.305006	5.103315
C	7.027286	7.562982	6.256480
H	6.411459	7.994477	7.033175
C	8.226356	7.640833	4.173137
H	8.562501	8.123501	3.264819
C	8.635564	6.314739	4.414189
H	9.265668	5.807182	3.698225
C	7.482370	6.237667	6.411812
H	7.198451	5.668491	7.284911
C	3.466626	3.174864	-2.999656
H	4.390329	3.189258	-3.560373
C	3.502533	3.138529	-1.591874
H	4.468090	3.116551	-1.103157
C	1.158295	3.185207	-3.020449
H	0.245575	3.201166	-3.599110
C	1.097236	3.157517	-1.612822
H	0.123572	3.145829	-1.140114
C	2.293253	3.133955	-0.850770
C	2.281206	3.104945	0.577170
C	1.132031	3.096208	2.535220
C	3.400100	3.007045	2.550546
C	4.632879	3.017813	3.270530
C	4.676445	2.974631	4.686654
H	3.772773	2.889495	5.274980
C	5.886268	3.103590	2.612392
H	5.960582	3.126918	1.532963
C	7.073950	3.170756	3.366985
H	8.026759	3.246443	2.863123
C	5.913868	3.052410	5.356048
H	5.943032	3.033698	6.435385
C	-0.107107	3.194099	3.238745
C	-1.345658	3.310460	2.559110
H	-1.400270	3.329076	1.478250
C	-2.545741	3.391100	3.290965
H	-3.488989	3.471547	2.769456
C	-1.424180	3.272745	5.301360
H	-1.471206	3.265656	6.380116
C	-0.174444	3.186619	4.655123
H	0.718696	3.113547	5.261885
C	-3.617393	13.580390	0.700939
H	-3.336068	14.596952	0.465495
C	-4.650994	11.628999	0.029666

H	-5.190361	11.093246	-0.738714
C	-4.352115	10.993163	1.250048
H	-4.686529	9.973567	1.391893
C	-3.646544	11.693442	2.261221
C	-3.273684	13.026155	1.950743
H	-2.736386	13.642662	2.660087
C	-3.340755	11.092760	3.520198
C	-3.439861	9.307144	4.918018
C	-2.414390	11.216690	5.590035
N	-4.541584	5.210897	5.589124
C	-3.833569	7.955265	5.157674
C	-4.483394	7.179567	4.165325
H	-4.743460	7.591870	3.199011
C	-4.810837	5.835516	4.424345
H	-5.308976	5.250532	3.663923
C	-3.589480	7.300976	6.390613
H	-3.127917	7.818405	7.221801
C	-3.942352	5.945382	6.550758
H	-3.743168	5.449941	7.490000
C	-1.661149	11.946535	6.559685
C	-1.270737	11.380342	7.798779
H	-1.563029	10.376600	8.077415
C	-0.491385	12.133583	8.700439
H	-0.203642	11.704487	9.648637
C	-0.453903	13.947321	7.285523
H	-0.132297	14.961891	7.098714
C	-1.231145	13.276169	6.323247
H	-1.483565	13.799885	5.410846
C	3.353795	6.130429	-5.221662
H	4.300691	5.633243	-5.377811
C	1.050873	6.025163	-5.279240
H	0.162496	5.444363	-5.481818
C	0.928856	7.356036	-4.835248
H	-0.064593	7.767498	-4.710017
C	3.330335	7.466645	-4.775134
H	4.273779	7.967755	-4.599891
C	2.090523	8.121301	-4.562054
C	2.015053	9.467837	-4.092798
C	0.777007	11.262189	-3.455945
C	3.043436	11.361693	-3.378359
C	-0.495890	11.868339	-3.229075
C	-1.709930	11.169931	-3.448633
H	-1.720482	10.147135	-3.803569
C	-2.942911	11.808943	-3.211946
H	-3.865706	11.273193	-3.383736
C	-1.900581	13.760720	-2.567515
H	-1.993948	14.783050	-2.230301
C	-0.622717	13.205216	-2.774059
H	0.243225	13.829532	-2.597419
C	4.239217	12.077771	-3.069206
C	4.213274	13.407104	-2.576113
H	3.282411	13.935783	-2.417836
C	5.420651	14.079466	-2.303368
H	5.399954	15.096164	-1.937859
C	5.523811	11.502944	-3.242866
H	5.649078	10.493627	-3.614191
C	6.677501	12.251889	-2.939834
H	7.655086	11.811223	-3.075562
C	11.088284	14.449653	-0.701147
H	10.690185	13.456628	-0.926947
H	12.063128	14.517568	-1.199884
H	11.291161	14.486854	0.376494
C	9.718657	16.358948	-0.008287
H	8.632878	16.321320	0.117160
H	10.170926	16.054570	0.943942
H	9.984055	17.414094	-0.149617
C	10.706986	16.310972	-2.254219
H	10.751861	17.372109	-1.977571
H	11.754693	16.037752	-2.433893
C	9.999159	16.245086	-3.630160
H	9.781387	17.282370	-3.915154
H	10.758114	15.923352	-4.354776
C	7.588343	16.221618	-4.084868
H	6.876260	16.227148	-3.255194
H	7.836121	17.270286	-4.292131
H	7.058992	15.849999	-4.971202
C	8.968562	14.296469	-4.710851
H	8.198887	14.279927	-5.492635
H	9.933529	14.345818	-5.230676
H	8.936779	13.330410	-4.198817
N	-4.332483	0.981829	5.658855
N	-6.301940	2.888317	6.647540
C	-4.142791	0.533357	4.253034
H	-3.161224	0.074780	4.079884
H	-4.886968	-0.206444	3.932181
H	-4.234597	1.382411	3.570917
C	-3.165492	0.606526	6.499050
H	-2.370286	0.109616	5.929303

H	-2.735957	1.501908	6.956784
H	-3.426285	-0.072053	7.320869
C	-5.597331	0.425811	6.222630
H	-5.385703	-0.253556	7.058320
H	-6.112601	-0.206136	5.487877
C	-6.646064	1.439436	6.742183
H	-7.581972	1.221263	6.211992
H	-6.856119	1.157880	7.782136
C	-7.322964	3.624030	5.853767
H	-7.743099	4.483840	6.390576
H	-6.880336	4.001462	4.927551
H	-8.176254	2.998566	5.562884
C	-6.146162	3.488718	7.998545
H	-6.790103	4.363376	8.155574
H	-6.375329	2.787621	8.810818
H	-5.111211	3.810875	8.143950
N	9.430319	1.570941	6.020387
N	10.649555	4.096055	6.826727
N	10.128345	15.494439	-1.146938
N	8.774734	15.397609	-3.730183
N	-6.714816	14.571210	-1.274293
N	-5.433847	14.733096	-3.891416
N	5.901973	12.079206	4.354168
N	6.059347	10.155881	5.793871
N	7.310230	10.233887	3.734666
N	8.274846	5.619597	5.511692
N	2.736160	13.191853	8.111779
N	2.318750	3.191262	-3.707797
N	3.478860	3.087637	1.203172
N	1.074682	3.140954	1.184672
N	2.258870	2.993352	3.276743
N	-2.596014	3.365015	4.638428
N	7.097308	3.147610	4.715448
N	-4.300236	12.901686	-0.244153
N	-3.714306	9.806466	3.692908
N	-2.754106	9.940693	5.897042
N	-2.683797	11.849370	4.423950
N	-0.080587	13.396396	8.458631
N	2.238123	5.415399	-5.474834
N	0.781154	9.988299	-3.906289
N	1.871155	11.998198	-3.157353
N	3.182860	10.097289	-3.833983
N	6.634898	13.520905	-2.485293
N	-3.047164	13.083815	-2.782965
Pd	1.538863	14.204818	9.525589
Pd	-4.870886	13.818479	-2.049963
Pd	8.862303	3.613488	5.768186
Pd	8.377388	14.494853	-1.839342
Pd	2.338109	3.345478	-5.810768
Pd	-4.436461	3.115507	5.639059
C	2.033346	5.748253	6.506871
C	2.472024	5.782298	5.195327
C	1.653014	6.242759	4.131098
C	0.333391	6.698796	4.433987
C	-0.058127	6.744645	5.793956
C	0.743636	6.284460	6.821840
H	3.119990	5.970932	2.572731
H	1.567971	6.597143	0.739879
H	3.476993	5.451920	4.946927
C	2.091926	6.248519	2.779443
C	-0.525182	7.079324	3.369370
H	-1.031989	7.159879	6.032426
H	-0.766608	7.282979	1.255736
C	-0.096132	7.012827	2.063567
C	1.231427	6.612322	1.769200
H	-1.533796	7.411852	3.596376
C	0.218173	6.325942	8.242265
C	-0.546981	5.050636	8.631799
H	-0.450086	7.187750	8.349986
H	1.031397	6.483588	8.954900
H	-0.889034	5.096307	9.668048
H	-1.427825	4.927935	7.989319
H	0.076638	4.158567	8.522586
C	2.949073	5.209432	7.588625
C	3.860145	6.288691	8.194321
H	3.573536	4.416413	7.164430
H	2.371831	4.735406	8.386214
H	4.456527	5.890407	9.018839
H	4.542742	6.675361	7.429544
H	3.280487	7.130967	8.583568
C	3.305908	9.147665	4.106920
C	3.019526	8.832053	5.542693
C	1.901458	9.470941	5.896965
C	1.420192	10.266923	4.720277
H	3.651020	8.180802	6.124547
H	1.378950	9.464257	6.842580
C	2.288468	10.631296	2.338263
C	0.925473	10.505268	1.640189

C	2.733310	12.098518	2.442629
H	3.032922	10.070972	1.763726
C	0.974486	11.170325	0.255788
H	0.164652	10.993373	2.257425
H	0.655781	9.450063	1.558123
C	2.772736	12.759015	1.058384
H	2.025389	12.631354	3.087671
H	3.715844	12.147946	2.919604
C	1.415870	12.636985	0.353849
H	-0.010505	11.096234	-0.218875
H	1.675158	10.618562	-0.386045
H	3.057408	13.811095	1.157542
H	3.545180	12.269288	0.447165
H	1.467966	13.093927	-0.638971
H	0.668514	13.203527	0.923832
N	2.297979	9.991755	3.665130
O	0.480859	11.035859	4.677629
O	4.255456	8.773049	3.433676
O	5.349184	6.162426	3.590898
H	5.611862	6.064738	2.666565
H	5.038221	7.085123	3.630333

Table5_1c_TSi_TSi-ii_h2o

Property	Value
Charge	0
Electronic Energy, BS1 (a.u.)	-1213.972035
Thermal and entropic correction, BS1 (a.u.)	2.734444
Electronic Energy, BS2 (a.u.)	-1214.384620
Number of Imaginary Frequencies	0
Imaginary frequencies (cm-1)	None

Molecular Geometry in Cartesian Coordinates

C	8.310691	0.945712	6.726043
H	8.645719	0.434620	7.637330
H	7.572536	1.689973	7.036796
H	7.785462	0.191828	6.126382
C	9.550694	1.043131	4.614892
H	8.798912	0.275210	4.393291
H	9.437031	1.837440	3.872105
H	10.528096	0.579335	4.431542
C	10.702173	1.495714	6.734687
H	11.446591	0.937303	6.152816
H	10.573519	0.898290	7.646374
C	11.387905	2.814574	7.167396
H	12.402229	2.787333	6.749075
H	11.544106	2.740795	8.251215
C	11.545954	4.916561	5.909335
H	11.069138	5.002861	4.928871
H	11.709511	5.933973	6.286250
H	12.541609	4.488508	5.738848
C	10.336072	4.853447	8.037814
H	9.248298	4.913701	8.128619
H	10.704020	4.389402	8.961422
H	10.724056	5.879742	8.034330
N	3.217579	15.022034	10.542587
N	0.330692	15.227233	10.916520
C	3.994831	13.881401	11.095152
H	3.464914	12.943966	10.907038
H	4.995801	13.792009	10.654630
H	4.141467	13.944894	12.180636
C	4.024812	15.788864	9.556337
H	3.507423	15.824405	8.593930
H	4.198119	16.829933	9.856963
H	5.015773	15.350967	9.382894
C	2.730549	15.913165	11.636167
H	3.152396	15.609706	12.602963
H	3.092097	16.940139	11.497652
C	1.199156	16.010262	11.843647
H	0.948551	17.078228	11.807412
H	1.009854	15.727101	12.887024
C	-0.576754	16.126151	10.153991
H	-1.633787	15.848195	10.250533
H	-0.506078	17.175861	10.465405
H	-0.326749	16.097620	9.089680
C	-0.442922	14.193481	11.654069
H	-0.132654	13.195230	11.332105
H	-0.293416	14.236111	12.740147
H	-1.525056	14.270409	11.489111
N	2.415174	1.243187	-6.164624
N	2.382748	3.612256	-7.867246
C	3.651457	0.711834	-5.532217
H	3.445608	0.008893	-4.715370

H	4.303646	0.181852	-6.237539
H	4.240927	1.534461	-5.117940
C	1.202242	0.637967	-5.553134
H	1.436262	-0.056749	-4.736732
H	0.559979	1.422515	-5.142900
H	0.592496	0.074847	-6.270623
C	2.434673	1.024993	-7.640690
H	1.581174	0.412003	-7.957603
H	3.312082	0.435685	-7.936534
C	2.430819	2.282871	-8.544119
H	3.315330	2.203969	-9.189180
H	1.585482	2.164736	-9.234064
C	1.146378	4.351736	-8.236497
H	1.350503	5.353769	-8.634006
H	0.545959	3.836480	-8.996600
H	0.505455	4.470940	-7.358623
C	3.595206	4.413566	-8.181739
H	3.358975	5.403114	-8.592729
H	4.185894	4.567312	-7.274127
H	4.256636	3.928134	-8.910114
C	-6.440643	15.544375	-0.207705
H	-6.833312	15.190814	0.753946
H	-5.358249	15.656531	-0.100361
H	-6.847495	16.552952	-0.354130
C	-7.553819	13.462654	-0.867411
H	-7.742493	13.480219	0.213207
H	-8.537680	13.402141	-1.349221
H	-7.035609	12.527340	-1.096257
C	-7.436809	15.340382	-2.441750
H	-7.658523	16.376799	-2.156717
H	-8.422907	14.901007	-2.639742
C	-6.707151	15.409620	-3.805947
H	-7.400771	14.997588	-4.550044
H	-6.638858	16.473918	-4.065169
C	-5.382439	13.671709	-4.924069
H	-4.599676	13.791700	-5.683537
H	-5.226757	12.702017	-4.443001
H	-6.331828	13.603738	-5.469808
C	-4.307676	15.750595	-4.199163
H	-3.709813	15.489622	-5.081442
H	-4.700959	16.758448	-4.381525
H	-3.623238	15.829278	-3.349412
N	7.959806	13.589486	-0.051956
C	7.063979	14.115375	0.808132
H	6.684643	15.099919	0.574718
C	6.626072	13.450141	1.970118
H	5.914293	13.951716	2.612795
C	7.139960	12.166808	2.287300
C	8.073521	11.625495	1.367610
H	8.521506	10.651725	1.520239
C	8.443691	12.361317	0.224995
H	9.153043	11.943896	-0.474869
C	6.752326	11.467689	3.470177
C	5.553628	11.383841	5.395685
C	4.592975	11.971749	6.275010
C	3.987226	13.223674	5.994832
H	4.223490	13.782427	5.098457
C	3.069595	13.783626	6.904727
H	2.616953	14.742630	6.694786
C	3.268926	11.985272	8.334882
H	2.973421	11.514310	9.260554
C	4.195761	11.347973	7.484852
H	4.605300	10.393616	7.788825
C	6.923786	9.663278	4.835176
C	7.424760	8.349028	5.080811
C	7.085350	7.622112	6.249034
H	6.471064	8.059630	7.023088
C	8.273171	7.683893	4.158919
H	8.594776	8.156416	3.240046
C	8.704811	6.368731	4.421108
H	9.339394	5.859341	3.710337
C	7.563585	6.308306	6.425972
H	7.297194	5.751284	7.312530
C	3.472075	3.090128	-2.950107
H	4.396773	3.116280	-3.508831
C	3.505532	3.054685	-1.542227
H	4.470219	3.046823	-1.051264
C	1.164465	3.066768	-2.975675
H	0.252920	3.069434	-3.556198
C	1.100363	3.036050	-1.568430
H	0.125837	3.009330	-1.098057
C	2.294873	3.030508	-0.804025
C	2.279746	3.005763	0.623703
C	1.124301	3.025596	2.579259
C	3.393963	2.996066	2.602427
C	4.628814	3.054929	3.316330
C	4.678523	3.080421	4.732313
H	3.776851	3.016399	5.325744

C	5.878932	3.116893	2.648283
H	5.949771	3.091417	1.568700
C	7.070242	3.222900	3.391774
H	8.019654	3.282869	2.880055
C	5.920120	3.188623	5.391253
H	5.958320	3.216271	6.470345
C	-0.124886	3.101056	3.268198
C	-1.358029	3.155971	2.569889
H	-1.400540	3.148369	1.488438
C	-2.570650	3.205087	3.282340
H	-3.508596	3.242682	2.746397
C	-1.473060	3.163712	5.311812
H	-1.539411	3.162785	6.390272
C	-0.210643	3.121707	4.683791
H	0.677058	3.089046	5.301905
C	-3.690081	13.616829	0.739258
H	-3.429998	14.642548	0.520122
C	-4.705458	11.662768	0.048114
H	-5.253419	11.134850	-0.719566
C	-4.370583	11.006785	1.248374
H	-4.686307	9.979562	1.376549
C	-3.658556	11.698472	2.260632
C	-3.313588	13.043275	1.970377
H	-2.773551	13.655106	2.681559
C	-3.323169	11.080440	3.503446
C	-3.367367	9.271610	4.873743
C	-2.388255	11.196104	5.568902
N	-4.470555	5.174261	5.532971
C	-3.733165	7.909251	5.099561
C	-4.408287	7.144731	4.114868
H	-4.690593	7.568199	3.159667
C	-4.749403	5.803836	4.373617
H	-5.273390	5.229618	3.622363
C	-3.449378	7.238473	6.315556
H	-2.957675	7.742791	7.137191
C	-3.831233	5.891048	6.481654
H	-3.624912	5.387900	7.415426
C	-1.642624	11.931468	6.540042
C	-1.242435	11.364634	7.775276
H	-1.517938	10.354632	8.047180
C	-0.476597	12.126067	8.681278
H	-0.181127	11.697036	9.627003
C	-0.468728	13.947320	7.277132
H	-0.164733	14.968399	7.096474
C	-1.232782	13.268461	6.309954
H	-1.493363	13.793763	5.400749
C	3.353839	6.107810	-5.082410
H	4.301089	5.615463	-5.250593
C	1.050039	5.990772	-5.113058
H	0.162542	5.405665	-5.308003
C	0.927092	7.319337	-4.661610
H	-0.066617	7.725718	-4.522662
C	3.328770	7.442486	-4.631471
H	4.271783	7.947648	-4.465553
C	2.088278	8.090559	-4.402639
C	2.012632	9.437809	-3.935222
C	0.775899	11.234325	-3.303370
C	3.042209	11.348528	-3.268862
C	-0.496709	11.847040	-3.094211
C	-1.711057	11.149081	-3.313709
H	-1.721867	10.120320	-3.650850
C	-2.943683	11.798123	-3.105344
H	-3.867275	11.264420	-3.279624
C	-1.899857	13.756758	-2.481717
H	-1.992430	14.784425	-2.161022
C	-0.622457	13.192308	-2.664321
H	0.244403	13.815566	-2.487839
C	4.239275	12.084463	-3.017161
C	4.214210	13.421229	-2.545373
H	3.282947	13.939857	-2.358480
C	5.421984	14.113925	-2.332217
H	5.400574	15.136954	-1.984525
C	5.524011	11.525031	-3.234117
H	5.648617	10.512178	-3.595849
C	6.677950	12.294130	-2.987778
H	7.655240	11.864414	-3.156483
C	11.086038	14.475673	-0.742650
H	10.693138	13.485502	-0.989187
H	12.060650	14.558276	-1.239517
H	11.287819	14.492273	0.335728
C	9.705936	16.364218	-0.013517
H	8.619899	16.321252	0.108636
H	10.156300	16.041252	0.933394
H	9.968998	17.422764	-0.132227
C	10.697209	16.365868	-2.258337
H	10.722974	17.423577	-1.966644
H	11.750642	16.111281	-2.431847
C	10.003757	16.306867	-3.641769

H	9.776311	17.344544	-3.917648
H	10.773507	16.001891	-4.362247
C	7.600549	16.257662	-4.131872
H	6.878235	16.256015	-3.311076
H	7.840218	17.308797	-4.336172
H	7.085598	15.880354	-5.024213
C	9.011264	14.350183	-4.744264
H	8.257610	14.332238	-5.541592
H	9.986474	14.407546	-5.243785
H	8.974697	13.381860	-4.236523
N	-4.520082	0.938738	5.658929
N	-6.382621	2.977439	6.601245
C	-4.421965	0.465258	4.252141
H	-3.490758	-0.077672	4.047593
H	-5.238624	-0.209004	3.965208
H	-4.463632	1.315210	3.566363
C	-3.340948	0.497931	6.449510
H	-2.607729	-0.057169	5.851078
H	-2.831095	1.367109	6.871926
H	-3.605111	-0.152627	7.292651
C	-5.788961	0.468414	6.287997
H	-5.582271	-0.176252	7.151843
H	-6.351330	-0.177612	5.601715
C	-6.781141	1.550422	6.779095
H	-7.736954	1.344940	6.280332
H	-6.979400	1.329866	7.835841
C	-7.361658	3.699133	5.744548
H	-7.767905	4.598458	6.224206
H	-6.884658	4.013796	4.811776
H	-8.226137	3.084198	5.464562
C	-6.228820	3.654261	7.916198
H	-6.841418	4.560251	8.005406
H	-6.499428	3.014795	8.765837
H	-5.186128	3.947102	8.064181
N	9.417251	1.607136	5.984587
N	10.701527	4.091783	6.814092
N	10.121516	15.524372	-1.168489
N	8.790926	15.445661	-3.762969
N	-6.745006	14.620726	-1.332647
N	-5.370134	14.753228	-3.903274
N	5.845120	12.071080	4.269669
N	6.057870	10.177653	5.742896
N	7.313465	10.259031	3.685972
N	8.361217	5.687170	5.532968
N	2.718456	13.185929	8.060908
N	2.325859	3.090347	-3.661007
N	3.474209	3.027408	1.253072
N	1.071965	3.023066	1.227529
N	2.251087	2.994715	3.329218
N	-2.636451	3.198726	4.628422
N	7.099275	3.257364	4.739260
N	-4.381280	12.946604	-0.205525
N	-3.667136	9.783466	3.659376
N	-2.701449	9.909751	5.862943
N	-2.676428	11.838064	4.412808
N	-0.086456	13.396788	8.447520
N	2.238443	5.389257	-5.325936
N	0.779063	9.952542	-3.731187
N	1.870444	11.978191	-3.026109
N	3.181010	10.078499	-3.708777
N	6.636870	13.569639	-2.551196
N	-3.046217	13.081005	-2.700854
Pd	1.545956	14.202164	9.490822
Pd	-4.881296	13.848727	-2.036826
Pd	8.890644	3.665450	5.772117
Pd	8.376483	14.531022	-1.882615
Pd	2.346079	3.335852	-5.753263
Pd	-4.493194	3.076357	5.614891
C	2.203288	6.030939	6.514388
C	2.744045	6.342734	5.217902
C	1.826593	6.454662	4.099446
C	0.542410	6.960201	4.400872
C	0.340421	7.349856	5.781506
C	0.967561	6.567901	6.811889
H	3.194684	5.880378	2.545741
H	1.464348	6.032463	0.749748
H	3.751068	6.009440	4.979960
C	2.175162	6.177496	2.766200
C	-0.405271	7.111125	3.373576
H	-0.592081	7.856444	6.021361
H	-0.821593	6.817541	1.287446
C	-0.083904	6.735893	2.077603
C	1.214653	6.288841	1.772510
H	-1.389016	7.508626	3.600245
C	0.315489	6.485578	8.172265
C	-0.552197	5.225209	8.315065
H	-0.312370	7.371139	8.324422
H	1.059243	6.496010	8.972977

H	-1.053726	5.198067	9.284918
H	-1.310939	5.204157	7.523537
H	0.054127	4.319638	8.222200
C	3.069505	5.343622	7.546648
C	3.958605	6.321593	8.332997
H	3.704897	4.600812	7.053550
H	2.445923	4.783411	8.247903
H	4.528478	5.802495	9.106864
H	4.666256	6.824843	7.667118
H	3.359828	7.092758	8.824948
C	3.333654	8.953670	4.162078
C	3.017441	8.437984	5.525293
C	1.757168	8.979708	5.886918
C	1.320825	9.866499	4.772234
H	3.838427	8.294779	6.212397
H	1.476595	9.256497	6.894088
C	2.229453	10.441927	2.455124
C	0.937118	10.178673	1.668151
C	2.457603	11.946178	2.676395
H	3.075626	10.031306	1.895718
C	0.955517	10.943704	0.335921
H	0.083619	10.503420	2.272092
H	0.828735	9.106251	1.495956
C	2.473980	12.699907	1.340621
H	1.650153	12.329042	3.309959
H	3.399396	12.095411	3.212805
C	1.184508	12.445931	0.549142
H	0.013910	10.766461	-0.196566
H	1.756848	10.540869	-0.298913
H	2.608796	13.770610	1.520342
H	3.334555	12.361621	0.744967
H	1.228241	12.970696	-0.410498
H	0.336206	12.868116	1.104324
N	2.259180	9.724873	3.739790
O	0.347492	10.596342	4.724328
O	4.342948	8.763219	3.491113
O	5.477055	6.216413	3.437876
H	5.658198	6.103204	2.496155
H	5.195287	7.149990	3.498408

Table5_1c_TSi_DG_h2o

**Property**	**Value**
Charge	0
Electronic Energy, BS1 (a.u.)	-1214.021533
Thermal and entropic correction, BS1 (a.u.)	2.739574
Electronic Energy, BS2 (a.u.)	-1214.432181
Number of Imaginary Frequencies	0
Imaginary frequencies (cm-1)	None

Molecular Geometry in Cartesian Coordinates

C	8.302752	0.934899	6.661330
H	8.632594	0.394420	7.557426
H	7.579387	1.683713	6.994236
H	7.761811	0.206329	6.044654
C	9.538743	1.062702	4.549788
H	8.774716	0.311837	4.312621
H	9.436062	1.875741	3.826012
H	10.508335	0.588041	4.353323
C	10.701822	1.448717	6.676107
H	11.433834	0.886999	6.081840
H	10.565230	0.838066	7.577812
C	11.413582	2.746934	7.128270
H	12.423920	2.710367	6.701082
H	11.577120	2.649430	8.209127
C	11.597786	4.869826	5.909237
H	11.113739	4.984327	4.935129
H	11.782434	5.876487	6.304561
H	12.584248	4.427957	5.721424
C	10.405112	4.786548	8.046673
H	9.319600	4.867368	8.146796
H	10.769608	4.296530	8.958115
H	10.814316	5.804495	8.061194
N	3.042258	15.072620	10.761865
N	0.129659	15.059725	10.905534
C	3.834258	13.977179	11.380613
H	3.382655	13.010833	11.140313
H	4.875223	13.953190	11.034540
H	3.875412	14.039980	12.475280
C	3.886681	15.892642	9.852755
H	3.456331	15.902950	8.847791
H	3.970287	16.939960	10.169389
H	4.913619	15.515753	9.768364

C	2.405379	15.926658	11.806873
H	2.768753	15.653730	12.806000
H	2.701938	16.977179	11.692393
C	0.859240	15.911290	11.890008
H	0.532998	16.957141	11.823134
H	0.608959	15.618610	12.917823
C	-0.802917	15.879149	10.086230
H	-1.833025	15.501995	10.106710
H	-0.853976	16.926724	10.408599
H	-0.477774	15.888046	9.042498
C	-0.592888	13.953962	11.587638
H	-0.164973	12.992054	11.290296
H	-0.525468	14.004791	12.681547
H	-1.662893	13.928463	11.345749
N	2.402223	1.177803	-6.134524
N	2.346791	3.531584	-7.857711
C	3.646252	0.661076	-5.505282
H	3.450628	-0.038318	-4.682929
H	4.299297	0.132228	-6.210661
H	4.230664	1.491366	-5.099273
C	1.197586	0.568974	-5.510241
H	1.441827	-0.117069	-4.689513
H	0.552280	1.352193	-5.102307
H	0.587394	-0.004669	-6.218971
C	2.414147	0.946799	-7.608712
H	1.560764	0.328601	-7.915660
H	3.291597	0.357326	-7.904008
C	2.402224	2.196712	-8.523071
H	3.286458	2.117546	-9.168502
H	1.556820	2.067591	-9.210949
C	1.103849	4.258823	-8.229324
H	1.299399	5.258302	-8.637433
H	0.503986	3.731836	-8.981768
H	0.465591	4.382417	-7.350218
C	3.552279	4.339021	-8.183421
H	3.307383	5.323234	-8.602183
H	4.144870	4.505402	-7.279269
H	4.214881	3.851924	-8.909606
C	-6.639384	15.532778	-0.337792
H	-7.145480	15.185215	0.571687
H	-5.571346	15.600290	-0.113350
H	-6.989780	16.558298	-0.509507
C	-7.759027	13.505845	-1.140993
H	-8.059938	13.520076	-0.085907
H	-8.686923	13.486140	-1.726265
H	-7.252141	12.554492	-1.326555
C	-7.407809	15.398614	-2.664102
H	-7.546124	16.457306	-2.409630
H	-8.417233	15.050484	-2.918246
C	-6.593694	15.363861	-3.981070
H	-7.272034	14.978518	-4.753091
H	-6.427114	16.409598	-4.269637
C	-5.367823	13.452869	-4.916936
H	-4.550179	13.458291	-5.648542
H	-5.305651	12.512523	-4.361394
H	-6.298697	13.416169	-5.496396
C	-4.163485	15.492790	-4.293065
H	-3.551645	15.120976	-5.124447
H	-4.470755	16.509067	-4.569791
H	-3.510075	15.589671	-3.422241
N	7.899615	13.551264	-0.030528
C	7.030225	14.092425	0.847381
H	6.630257	15.065623	0.600992
C	6.643972	13.455266	2.043274
H	5.950981	13.966428	2.698626
C	7.182432	12.185785	2.374983
C	8.088055	11.629347	1.436543
H	8.551909	10.664506	1.598155
C	8.407732	12.336653	0.261362
H	9.094515	11.906774	-0.453486
C	6.841300	11.510722	3.585847
C	5.694708	11.447280	5.543600
C	4.751601	12.042293	6.437485
C	4.149027	13.297706	6.164751
H	4.387379	13.862038	5.272786
C	3.232852	13.855894	7.077067
H	2.781270	14.816356	6.871471
C	3.440751	12.056249	8.506519
H	3.152880	11.589432	9.436907
C	4.366104	11.420997	7.652638
H	4.785223	10.471480	7.958892
C	7.034741	9.710719	4.955473
C	7.514015	8.383635	5.176377
C	7.172929	7.640158	6.334215
H	6.555693	8.061889	7.115797
C	8.341809	7.720821	4.233857
H	8.659574	8.201835	3.318215
C	8.760315	6.396809	4.469453

H	9.378202	5.890449	3.742015
C	7.648003	6.321672	6.488511
H	7.387201	5.754300	7.370366
C	3.460500	3.063782	-2.939845
H	4.382683	3.087277	-3.502626
C	3.500083	3.050436	-1.531436
H	4.466224	3.057573	-1.043381
C	1.153109	3.026042	-2.956013
H	0.239110	3.015090	-3.532599
C	1.094940	3.016137	-1.548514
H	0.121957	2.990478	-1.075184
C	2.292374	3.030671	-0.788497
C	2.281392	3.030276	0.639491
C	1.125116	3.062966	2.595333
C	3.396320	3.042217	2.618963
C	4.634425	3.102128	3.328787
C	4.692202	3.125502	4.744641
H	3.794772	3.057081	5.342189
C	5.881966	3.166747	2.655442
H	5.948664	3.143402	1.575588
C	7.076712	3.277459	3.393151
H	8.022963	3.344760	2.876458
C	5.936862	3.234777	5.398464
H	5.979962	3.260814	6.477783
C	-0.128989	3.135214	3.276732
C	-1.358063	3.198909	2.570658
H	-1.394006	3.199971	1.488983
C	-2.576076	3.252204	3.274131
H	-3.510035	3.300202	2.731859
C	-1.492872	3.191195	5.309475
H	-1.567882	3.188667	6.387535
C	-0.225270	3.146138	4.691237
H	0.658108	3.103993	5.315003
C	-3.860653	13.623612	0.781817
H	-3.569303	14.636635	0.543017
C	-5.004430	11.718448	0.164568
H	-5.617840	11.210384	-0.565479
C	-4.638397	11.054619	1.351004
H	-4.995077	10.044116	1.503058
C	-3.830156	11.713005	2.312343
C	-3.442151	13.039343	1.994554
H	-2.834849	13.627657	2.670356
C	-3.438568	11.078077	3.529951
C	-3.466390	9.261005	4.890537
C	-2.371447	11.142861	5.534025
N	-4.543826	5.155229	5.541641
C	-3.833566	7.899291	5.115865
C	-4.533727	7.142130	4.143127
H	-4.838543	7.572432	3.197843
C	-4.856347	5.795155	4.396047
H	-5.390921	5.224153	3.649981
C	-3.515216	7.218696	6.317072
H	-2.999567	7.715331	7.128650
C	-3.888990	5.868862	6.481294
H	-3.651927	5.356738	7.403064
C	-1.575726	11.849112	6.486987
C	-1.156546	11.260254	7.706903
H	-1.446165	10.253725	7.977611
C	-0.356284	11.995976	8.604116
H	-0.045279	11.550036	9.537176
C	-0.341948	13.834113	7.212819
H	-0.017780	14.849705	7.035556
C	-1.140384	13.178525	6.256650
H	-1.409059	13.714198	5.355807
C	3.308090	6.058134	-5.100289
H	4.258357	5.569992	-5.263546
C	1.004914	5.925058	-5.124163
H	0.121179	5.330885	-5.308127
C	0.873541	7.258306	-4.688721
H	-0.122183	7.659427	-4.549068
C	3.274774	7.397162	-4.664180
H	4.214981	7.909089	-4.503808
C	2.030299	8.039863	-4.441341
C	1.947695	9.388179	-3.978802
C	0.706290	11.172953	-3.322219
C	2.972663	11.300716	-3.311607
C	-0.564630	11.765139	-3.050009
C	-1.779757	11.061043	-3.247037
H	-1.791870	10.042796	-3.614943
C	-3.010719	11.687721	-2.968109
H	-3.935262	11.148558	-3.117984
C	-1.961214	13.636918	-2.313822
H	-2.052925	14.649754	-1.947715
C	-0.686699	13.092777	-2.565637
H	0.180429	13.716542	-2.393313
C	4.167707	12.033178	-3.040505
C	4.139426	13.368591	-2.564613
H	3.207374	13.891608	-2.395755

C	5.345449	14.055036	-2.323674
H	5.321761	15.076122	-1.970483
C	5.453903	11.469382	-3.236178
H	5.581118	10.457177	-3.598901
C	6.606336	12.232412	-2.964392
H	7.585104	11.799401	-3.115551
C	11.015954	14.446339	-0.743378
H	10.627425	13.452815	-0.983022
H	11.990163	14.529816	-1.240916
H	11.217723	14.471212	0.334797
C	9.634341	16.338910	-0.028411
H	8.549053	16.293965	0.098937
H	10.090548	16.028015	0.919802
H	9.893466	17.396905	-0.160082
C	10.615617	16.318890	-2.277464
H	10.643066	17.379327	-1.995995
H	11.667976	16.062770	-2.455203
C	9.913703	16.246890	-3.655979
H	9.683860	17.281903	-3.939767
H	10.679265	15.936099	-4.378436
C	7.507128	16.192323	-4.128318
H	6.790934	16.196225	-3.302219
H	7.744404	17.242140	-4.342057
H	6.986159	15.808291	-5.014291
C	8.915142	14.281739	-4.737316
H	8.155399	14.257118	-5.528614
H	9.886273	14.337015	-5.244967
H	8.884184	13.316909	-4.222716
N	-4.489139	0.919183	5.604627
N	-6.399466	2.897843	6.576882
C	-4.374974	0.471014	4.190833
H	-3.430850	-0.047264	3.981482
H	-5.175344	-0.216347	3.889421
H	-4.433605	1.331335	3.519129
C	-3.302951	0.494287	6.393377
H	-2.550295	-0.025522	5.787575
H	-2.821414	1.367731	6.839956
H	-3.553776	-0.182826	7.219456
C	-5.748558	0.408857	6.221380
H	-5.529004	-0.248752	7.072181
H	-6.296323	-0.234802	5.521205
C	-6.763263	1.459262	6.734515
H	-7.716430	1.239017	6.237013
H	-6.950724	1.217766	7.788661
C	-7.397203	3.608136	5.732431
H	-7.821272	4.492434	6.224478
H	-6.930891	3.944026	4.801936
H	-8.248974	2.977688	5.448106
C	-6.259136	3.559395	7.901181
H	-6.892801	4.449272	8.004425
H	-6.512440	2.901715	8.742266
H	-5.223015	3.875212	8.050678
N	9.416997	1.596797	5.932388
N	10.746670	4.042364	6.805250
N	10.046747	15.487790	-1.176092
N	8.700727	15.383871	-3.762102
N	-6.862835	14.635598	-1.503447
N	-5.310504	14.601289	-3.974225
N	5.952911	12.120793	4.401096
N	6.188537	10.232328	5.878356
N	7.413495	10.308222	3.803585
N	8.426286	5.703895	5.576548
N	2.885681	13.254298	8.232118
N	2.311333	3.046317	-3.646374
N	3.475430	3.064730	1.268878
N	1.074683	3.052373	1.243992
N	2.252510	3.042840	3.346541
N	-2.652061	3.238708	4.619545
N	7.112399	3.310076	4.740538
N	-4.632134	12.982608	-0.120959
N	-3.825649	9.795675	3.703531
N	-2.719381	9.869250	5.840100
N	-2.689926	11.797878	4.393082
N	0.045670	13.262620	8.371280
N	2.197252	5.329323	-5.332414
N	0.712069	9.896747	-3.767437
N	1.799660	11.927137	-3.069892
N	3.114664	10.031929	-3.752171
N	6.561435	13.505915	-2.522363
N	-3.106634	12.954535	-2.515232
Pd	1.527011	14.160542	9.562934
Pd	-4.969214	13.797075	-2.027740
Pd	8.920005	3.667591	5.769744
Pd	8.300580	14.481933	-1.871622
Pd	2.319775	3.273502	-5.741146
Pd	-4.513462	3.056501	5.592373
C	2.239337	6.461523	6.631578
C	2.870124	6.946898	5.327578

C	1.854868	6.838827	4.209981
C	0.631744	7.451409	4.520045
C	0.640060	8.097632	5.892226
C	1.077065	7.066939	6.931083
H	3.068595	5.919770	2.706932
H	1.203548	5.909354	1.017168
H	3.797298	6.437202	5.071688
C	2.084175	6.310550	2.945063
C	-0.386813	7.490203	3.574638
H	-0.312337	8.572704	6.133410
H	-0.976167	6.920762	1.579248
C	-0.184083	6.905262	2.319370
C	1.048833	6.332042	2.003215
H	-1.323688	7.987810	3.797770
C	0.184377	6.761128	8.104589
C	-0.684078	5.522178	7.833046
H	-0.459492	7.622991	8.314162
H	0.770501	6.591567	9.012449
H	-1.419705	5.372061	8.627955
H	-1.194794	5.628007	6.867983
H	-0.055093	4.628745	7.779622
C	2.903519	5.371868	7.426911
C	4.389243	5.628497	7.714987
H	2.804249	4.426386	6.875390
H	2.376294	5.218502	8.372085
H	4.808432	4.811081	8.307483
H	4.955918	5.717233	6.781095
H	4.523519	6.550113	8.288540
C	3.544755	9.139318	4.209109
C	3.118649	8.488380	5.513433
C	1.785702	9.178055	5.866666
C	1.564674	10.197728	4.760174
H	3.896823	8.640403	6.264936
H	1.807771	9.692673	6.830253
C	2.682199	10.812134	2.559229
C	1.552736	10.325269	1.636915
C	2.667798	12.337253	2.729544
H	3.641978	10.508481	2.130358
C	1.641957	11.027353	0.274852
H	0.592133	10.552217	2.111589
H	1.614151	9.238681	1.525021
C	2.755803	13.024637	1.359749
H	1.741768	12.630616	3.231876
H	3.501938	12.645178	3.367090
C	1.626659	12.553535	0.433502
H	0.815767	10.694203	-0.361229
H	2.569214	10.720731	-0.228183
H	2.718671	14.110249	1.487994
H	3.724850	12.785674	0.895204
H	1.711472	13.045966	-0.540307
H	0.663352	12.862545	0.860505
N	2.625661	10.111806	3.856814
O	0.614940	10.946955	4.645372
O	4.528346	8.862389	3.536598
O	5.506206	6.238550	3.466914
H	5.599339	6.088014	2.517535
H	5.285910	7.187371	3.518059

Table5_1c_reactant

Property	Value
Charge	0
Electronic Energy, BS1 (a.u.)	-1290.455014
Thermal and entropic correction, BS1 (a.u.)	2.850834
Electronic Energy, BS2 (a.u.)	-1290.908198
Number of Imaginary Frequencies	0
Imaginary frequencies (cm-1)	None

Molecular Geometry in Cartesian Coordinates

C	8.297136	1.028886	6.862178
H	8.651496	0.544286	7.780601
H	7.556047	1.773111	7.166373
H	7.771002	0.254749	6.289580
C	9.505880	1.085096	4.731353
H	8.764764	0.298012	4.543735
H	9.364793	1.856193	3.968777
H	10.488249	0.633665	4.543921
C	10.682190	1.605946	6.821783
H	11.432896	1.062169	6.234199
H	10.578198	1.010469	7.737905
C	11.340018	2.942709	7.242512
H	12.354541	2.933453	6.823852
H	11.498383	2.881683	8.326834

C	11.457111	5.041655	5.976697
H	10.981954	5.117353	4.994602
H	11.600510	6.062149	6.353359
H	12.461303	4.632599	5.809337
C	10.233227	4.960003	8.097153
H	9.143447	4.994721	8.179097
H	10.605013	4.508778	9.025558
H	10.596262	5.995370	8.091753
N	3.136140	14.989118	10.379485
N	0.268250	15.270290	10.839755
C	3.903095	13.820614	10.885933
H	3.340791	12.900485	10.705824
H	4.883063	13.711070	10.404971
H	4.093815	13.864494	11.965562
C	3.929125	15.754702	9.380961
H	3.380800	15.822569	8.437824
H	4.139561	16.784777	9.695358
H	4.902135	15.295639	9.165928
C	2.706654	15.871306	11.503958
H	3.143806	15.534602	12.452749
H	3.096485	16.889325	11.376396
C	1.185069	16.012185	11.753990
H	0.966614	17.087677	11.739811
H	1.015103	15.719031	12.797940
C	-0.629095	16.207272	10.112154
H	-1.692007	15.965293	10.237400
H	-0.512397	17.250981	10.429807
H	-0.411692	16.178675	9.040712
C	-0.519450	14.251901	11.583883
H	-0.247028	13.248552	11.243856
H	-0.344585	14.277346	12.666732
H	-1.602150	14.362720	11.444100
N	2.395953	1.270027	-6.135995
N	2.410855	3.639438	-7.838885
C	3.607909	0.718030	-5.474538
H	3.371180	0.016901	-4.664445
H	4.268843	0.178829	-6.164633
H	4.200216	1.530314	-5.044161
C	1.158897	0.685151	-5.554002
H	1.361922	-0.017484	-4.736148
H	0.522721	1.480020	-5.154385
H	0.554420	0.136800	-6.287290
C	2.447492	1.051856	-7.611323
H	1.599747	0.440459	-7.946197
H	3.329604	0.460580	-7.888660
C	2.463856	2.309708	-8.514743
H	3.357511	2.226705	-9.146475
H	1.628554	2.195377	-9.217479
C	1.179431	4.380863	-8.220518
H	1.388622	5.384281	-8.612047
H	0.587934	3.868360	-8.989461
H	0.528132	4.497421	-7.349959
C	3.627812	4.438797	-8.141339
H	3.397500	5.424839	-8.563960
H	4.205206	4.599953	-7.226366
H	4.300171	3.948150	-8.856185
C	-6.570324	15.722552	-0.616502
H	-7.099982	15.417369	0.294659
H	-5.508944	15.805156	-0.367873
H	-6.918779	16.736750	-0.848927
C	-7.654470	13.650907	-1.348109
H	-7.993961	13.721910	-0.307093
H	-8.561739	13.591289	-1.962128
H	-7.135093	12.694953	-1.459544
C	-7.268702	15.465708	-2.955601
H	-7.498171	16.515899	-2.733867
H	-8.230913	15.043357	-3.272448
C	-6.358955	15.474094	-4.209225
H	-6.964592	15.073471	-5.032212
H	-6.209047	16.527922	-4.476826
C	-4.988541	13.638655	-5.090537
H	-4.107715	13.689371	-5.742790
H	-4.951115	12.682752	-4.561283
H	-5.860238	13.595104	-5.755549
C	-3.915714	15.697714	-4.309350
H	-3.232795	15.381990	-5.107996
H	-4.235766	16.715700	-4.564427
H	-3.330678	15.773222	-3.388354
N	8.144864	13.662784	0.007703
C	7.248931	14.207131	0.857161
H	6.955410	15.229291	0.664284
C	6.706782	13.520132	1.961097
H	6.012067	14.044923	2.603327
C	7.107442	12.187724	2.229967
C	8.046992	11.627729	1.328828
H	8.422850	10.620121	1.451361
C	8.525743	12.391034	0.247080
H	9.244043	11.959182	-0.434957

C	6.617658	11.462022	3.356437
C	5.370831	11.385202	5.250172
C	4.405659	11.975248	6.123646
C	3.823359	13.240449	5.856703
H	4.085760	13.811873	4.975982
C	2.897071	13.796278	6.761522
H	2.461842	14.765394	6.562608
C	3.041420	11.969116	8.158993
H	2.716379	11.480944	9.066805
C	3.978462	11.337199	7.316201
H	4.366895	10.371360	7.611959
C	6.720940	9.640789	4.703006
C	7.235202	8.338157	4.982083
C	6.920849	7.642965	6.177360
H	6.293532	8.084000	6.940211
C	8.096636	7.663907	4.079760
H	8.399507	8.110756	3.141896
C	8.581979	6.379810	4.394534
H	9.237601	5.868736	3.704117
C	7.455729	6.359620	6.407418
H	7.221716	5.833651	7.321584
C	3.455796	3.151968	-2.914020
H	4.384350	3.161494	-3.467112
C	3.479370	3.164532	-1.506071
H	4.441815	3.174323	-1.010776
C	1.149640	3.108946	-2.954851
H	0.242783	3.080807	-3.541955
C	1.075153	3.122293	-1.547139
H	0.098332	3.097277	-1.081786
C	2.264438	3.148531	-0.775047
C	2.243715	3.157194	0.652952
C	1.089132	3.166939	2.607528
C	3.358585	3.194534	2.630043
C	4.591166	3.217245	3.349529
C	4.637744	3.175876	4.765482
H	3.734917	3.100753	5.357310
C	5.843490	3.281508	2.688223
H	5.915952	3.305589	1.608972
C	7.034642	3.313464	3.438040
H	7.987223	3.364538	2.930765
C	5.879671	3.213097	5.430275
H	5.912771	3.178706	6.509604
C	-0.156922	3.188164	3.304517
C	-1.394945	3.101722	2.619851
H	-1.443123	3.010645	1.542615
C	-2.602483	3.107517	3.343175
H	-3.544806	3.038109	2.818422
C	-1.489469	3.288834	5.357072
H	-1.548295	3.361599	6.433576
C	-0.232594	3.288020	4.717520
H	0.659229	3.358329	5.327084
C	-3.943108	13.742984	0.708361
H	-3.716288	14.782471	0.520001
C	-4.906111	11.782359	-0.027872
H	-5.449251	11.261261	-0.803934
C	-4.549375	11.103908	1.155188
H	-4.838545	10.066453	1.263073
C	-3.853705	11.790311	2.181693
C	-3.549201	13.151006	1.923271
H	-3.032606	13.766787	2.648137
C	-3.495294	11.158649	3.410673
C	-3.509869	9.347718	4.776258
C	-2.554801	11.286751	5.472745
N	-4.461982	5.228473	5.513500
C	-3.835777	7.978405	5.020302
C	-4.486004	7.170683	4.053106
H	-4.784877	7.568423	3.091678
C	-4.773716	5.820501	4.341180
H	-5.279490	5.209155	3.606663
C	-3.516135	7.341113	6.246037
H	-3.036360	7.874659	7.056576
C	-3.841482	5.985170	6.441217
H	-3.598729	5.507329	7.379936
C	-1.831291	12.028401	6.454039
C	-1.441717	11.456857	7.690182
H	-1.713222	10.443760	7.953934
C	-0.669904	12.206607	8.598476
H	-0.370838	11.763188	9.536954
C	-0.658655	14.036854	7.207377
H	-0.354027	15.059325	7.036059
C	-1.424601	13.367286	6.234072
H	-1.688463	13.898189	5.329269
C	3.396937	6.100635	-4.982905
H	4.339638	5.601279	-5.156938
C	1.094944	6.032261	-5.086427
H	0.202951	5.479049	-5.343920
C	0.983033	7.335794	-4.565105
H	-0.006308	7.757101	-4.443787

C	3.383781	7.408712	-4.458562
H	4.330804	7.887244	-4.242780
C	2.149968	8.069481	-4.233079
C	2.083738	9.393077	-3.700419
C	0.853519	11.169563	-3.005052
C	3.119385	11.258117	-2.925291
C	-0.418436	11.793746	-2.829123
C	-1.628822	11.124453	-3.141294
H	-1.637408	10.110491	-3.519726
C	-2.860896	11.787889	-2.987142
H	-3.781331	11.277375	-3.232762
C	-1.824424	13.708828	-2.237379
H	-1.921278	14.727673	-1.889506
C	-0.547157	13.124718	-2.357222
H	0.319660	13.721802	-2.104631
C	4.319507	12.002353	-2.719572
C	4.301382	13.356041	-2.299506
H	3.373514	13.875761	-2.101325
C	5.510139	14.067120	-2.169413
H	5.493293	15.104471	-1.867539
C	5.599426	11.441249	-2.958522
H	5.718938	10.414831	-3.280360
C	6.754722	12.229315	-2.795195
H	7.728106	11.799869	-2.984924
C	11.218932	14.510105	-0.824904
H	10.805114	13.511677	-0.992061
H	12.175049	14.549266	-1.361715
H	11.462639	14.590746	0.241872
C	9.881037	16.451016	-0.159053
H	8.800100	16.420954	0.005222
H	10.364286	16.180370	0.788073
H	10.143394	17.499246	-0.349140
C	10.792335	16.313963	-2.434585
H	10.862944	17.382605	-2.194551
H	11.827682	16.019124	-2.648492
C	10.025188	16.215825	-3.776867
H	9.801614	17.246276	-4.081473
H	10.750137	15.867953	-4.523856
C	7.595467	16.196777	-4.123366
H	6.914858	16.217867	-3.267685
H	7.838383	17.240817	-4.357626
H	7.031982	15.812354	-4.982829
C	8.935658	14.247096	-4.759004
H	8.126461	14.209462	-5.498994
H	9.872243	14.281436	-5.329349
H	8.930592	13.295632	-4.219516
N	-4.508305	1.008551	5.903082
N	-6.371536	3.097425	6.721005
C	-4.402070	0.428319	4.537267
H	-3.460030	-0.109804	4.373790
H	-5.204914	-0.283266	4.306985
H	-4.464539	1.219575	3.786188
C	-3.326310	0.643292	6.728317
H	-2.589207	0.044571	6.178604
H	-2.820705	1.548288	7.077610
H	-3.587207	0.063859	7.622767
C	-5.774864	0.575917	6.563686
H	-5.564903	0.006495	7.478269
H	-6.326128	-0.130632	5.930098
C	-6.782661	1.682995	6.958773
H	-7.722612	1.452759	6.440838
H	-7.015059	1.520261	8.019100
C	-7.355749	3.796899	5.852413
H	-7.738366	4.722512	6.300186
H	-6.891900	4.062716	4.898296
H	-8.235436	3.184656	5.617230
C	-6.189580	3.821254	8.006792
H	-6.783749	4.741700	8.067597
H	-6.463196	3.219857	8.882680
H	-5.139239	4.097241	8.132900
N	9.385386	1.683116	6.087959
N	10.625785	4.201852	6.879739
N	10.249262	15.542981	-1.277731
N	8.792357	15.374973	-3.800301
N	-6.754083	14.767470	-1.741349
N	-5.052265	14.758781	-4.114053
N	5.683691	12.068434	4.125600
N	5.859752	10.174045	5.601167
N	7.110772	10.219660	3.547080
N	8.276336	5.734565	5.538519
N	2.513358	13.183007	7.899592
N	2.315839	3.118736	-3.633283
N	3.437364	3.227316	1.281070
N	1.033926	3.152736	1.255241
N	2.216595	3.188190	3.354852
N	-2.656607	3.193754	4.686881
N	7.063255	3.277333	4.785600
N	-4.617033	13.081025	-0.253744

N	-3.814399	9.851664	3.557871
N	-2.860472	10.001757	5.763683
N	-2.827864	11.915756	4.307900
N	-0.271386	13.475893	8.372176
N	2.276629	5.417928	-5.298537
N	0.851998	9.907951	-3.489173
N	1.948127	11.886031	-2.665417
N	3.252450	9.999418	-3.402065
N	6.719475	13.523517	-2.417248
N	-2.964420	13.059557	-2.552253
Pd	1.412097	14.226664	9.370107
Pd	-4.842193	13.914487	-2.166198
Pd	8.833333	3.728757	5.827560
Pd	8.475679	14.527634	-1.871366
Pd	2.353177	3.363711	-5.725530
Pd	-4.498727	3.135350	5.699943
C	2.760644	5.810861	5.978453
C	2.961521	6.088944	4.641527
C	1.860273	6.442733	3.808708
C	0.639819	6.826911	4.435325
C	0.608423	6.886660	5.855469
C	1.571552	6.225316	6.605533
H	2.879466	6.168651	1.935873
H	3.566094	5.413573	6.595105
H	3.937662	5.954827	4.170949
C	1.918443	6.395133	2.395840
C	-0.477136	7.106376	3.616719
H	-0.238274	7.365349	6.342191
H	1.471232	6.140706	7.689443
C	-0.431419	6.986282	2.240515
C	0.806472	6.629297	1.613650
H	-1.401868	7.411658	4.094628
C	-1.679703	7.278785	1.429367
C	-2.517441	6.029351	1.123271
H	-1.415009	7.771848	0.490580
H	-2.293801	7.999306	1.981327
H	-3.388176	6.274981	0.518057
H	-1.932917	5.284413	0.576193
H	-2.860308	5.568353	2.055368
C	0.957225	6.602206	0.110032
C	1.302341	7.990616	-0.452664
H	0.056911	6.217783	-0.372514
H	1.769269	5.916384	-0.150555
H	1.476350	7.919261	-1.526285
H	0.489105	8.703301	-0.291601
H	2.203015	8.393899	0.019738
C	3.175627	9.068528	3.840094
C	3.250411	8.498745	5.202042
C	2.188314	8.873247	5.887981
C	1.362680	9.750275	5.031705
H	4.204404	8.039480	5.559723
H	1.985513	8.814382	6.970071
C	1.591830	10.616109	2.640284
C	0.117228	10.420461	2.259915
C	1.913972	12.094936	2.915721
H	2.210805	10.291108	1.798558
C	-0.245795	11.294884	1.056473
H	-0.503813	10.689121	3.115025
H	-0.056334	9.367276	2.045302
C	1.535112	12.962673	1.710321
H	1.354248	12.414789	3.807166
H	2.980615	12.193766	3.155497
C	0.059001	12.772233	1.337156
H	-1.301718	11.151718	0.794293
H	0.342202	10.970018	0.183764
H	1.741613	14.014433	1.921728
H	2.159535	12.672151	0.854747
H	-0.191450	13.394176	0.471762
H	-0.568461	13.120256	2.168593
N	1.989679	9.770381	3.767508
O	0.399847	10.428818	5.339287
O	4.026511	9.026004	2.941307
O	5.357659	6.539645	2.602252
H	5.638662	6.436442	1.684863
H	5.023501	7.455172	2.660300
O	4.252266	10.967387	0.698443
H	4.278734	10.231469	1.332989
H	4.192520	11.746723	1.264647

Table5_1c_TSi_TSi-ii_h2o_2

Property	Value
Charge	0
Electronic Energy, BS1 (a.u.)	-1290.415402

Thermal and entropic correction, BS1 (a.u.)	2.856011
Electronic Energy, BS2 (a.u.)	-1290.864747
Number of Imaginary Frequencies	0
Imaginary frequencies (cm-1)	None

Molecular Geometry in Cartesian Coordinates

C	8.291177	1.029373	6.857415
H	8.642826	0.542260	7.775553
H	7.551118	1.774586	7.161628
H	7.764408	0.257473	6.282384
C	9.503894	1.087081	4.728869
H	8.761711	0.301665	4.538518
H	9.365400	1.859972	3.967641
H	10.485787	0.634324	4.542085
C	10.677510	1.601372	6.822431
H	11.427327	1.055041	6.236077
H	10.570115	1.006343	7.738449
C	11.339140	2.936035	7.243848
H	12.353835	2.923898	6.825683
H	11.496796	2.874214	8.328226
C	11.462683	5.034658	5.977926
H	10.987776	5.111516	4.995775
H	11.608932	6.054823	6.354386
H	12.465726	4.622711	5.810806
C	10.238802	4.956794	8.098492
H	9.149153	4.994738	8.180728
H	10.609488	4.504564	9.026849
H	10.604876	5.991086	8.092958
N	3.137979	14.988326	10.376131
N	0.270591	15.270088	10.839035
C	3.905453	13.819767	10.881662
H	3.342809	12.899686	10.702354
H	4.884773	13.710077	10.399424
H	4.097593	13.863709	11.961037
C	3.929883	15.753946	9.376789
H	3.380572	15.821686	8.434216
H	4.140533	16.784056	9.690918
H	4.902713	15.294960	9.160787
C	2.709734	15.870423	11.501136
H	3.147716	15.533494	12.449463
H	3.099664	16.888377	11.373360
C	1.188412	16.011536	11.752628
H	0.970169	17.087076	11.738917
H	1.019349	15.718126	12.796651
C	-0.626884	16.207475	10.112121
H	-1.689815	15.966020	10.238226
H	-0.509418	17.251146	10.429619
H	-0.410339	16.178670	9.040510
C	-0.517011	14.251997	11.583674
H	-0.245333	13.248556	11.243306
H	-0.341326	14.277272	12.666397
H	-1.599762	14.363325	11.444715
N	2.396714	1.270004	-6.132596
N	2.411719	3.637326	-7.838345
C	3.608360	0.718567	-5.470119
H	3.371253	0.018482	-4.659235
H	4.269384	0.178390	-6.159366
H	4.200711	1.531262	-5.040589
C	1.159365	0.686104	-5.550244
H	1.362003	-0.015521	-4.731430
H	0.523216	1.481613	-5.151843
H	0.555020	0.136911	-6.283010
C	2.448625	1.050034	-7.607644
H	1.600748	0.438567	-7.942052
H	3.330606	0.458091	-7.883981
C	2.465795	2.306804	-8.512560
H	3.360124	2.223230	-9.143259
H	1.631259	2.191464	-9.216041
C	1.179755	4.377351	-8.220953
H	1.388208	5.380428	-8.613756
H	0.588633	3.863422	-8.989236
H	0.528359	4.494509	-7.350547
C	3.628094	4.437232	-8.141727
H	3.397057	5.422582	-8.565576
H	4.205329	4.599986	-7.226937
H	4.300853	3.946207	-8.855939
C	-6.572428	15.724210	-0.618841
H	-7.103885	15.419968	0.291585
H	-5.511424	15.806287	-0.368427
H	-6.919811	16.738488	-0.852501
C	-7.656627	13.652784	-1.350984
H	-7.997731	13.724587	-0.310544
H	-8.562970	13.593461	-1.966400
H	-7.137702	12.696410	-1.461027
C	-7.267021	15.466358	-2.958960
H	-7.496608	16.516713	-2.738124

H	-8.228749	15.044125	-3.277425
C	-6.354863	15.473973	-4.210839
H	-6.959195	15.073630	-5.034915
H	-6.203758	16.527654	-4.478350
C	-4.984197	13.637328	-5.089093
H	-4.102018	13.687058	-5.739592
H	-4.948728	12.681569	-4.559477
H	-5.854637	13.594332	-5.755793
C	-3.911319	15.695896	-4.306617
H	-3.227251	15.379535	-5.104023
H	-4.230207	16.714065	-4.562434
H	-3.327901	15.771207	-3.384578
N	8.142770	13.661093	0.005175
C	7.245974	14.204882	0.854072
H	6.952703	15.227200	0.661636
C	6.702695	13.517174	1.957002
H	6.007448	14.041638	2.598932
C	7.103182	12.184648	2.225541
C	8.043374	11.625089	1.324797
H	8.419136	10.617415	1.447133
C	8.523253	12.389106	0.244021
H	9.242268	11.957715	-0.437567
C	6.613191	11.458772	3.351811
C	5.367100	11.382499	5.246083
C	4.402805	11.973128	6.120079
C	3.820775	13.238489	5.853326
H	4.082621	13.809593	4.972241
C	2.895449	13.794819	6.758828
H	2.460329	14.764017	6.560137
C	3.040083	11.967765	8.156392
H	2.715282	11.479662	9.064313
C	3.976325	11.335469	7.313060
H	4.364180	10.369293	7.608380
C	6.718708	9.638846	4.699936
C	7.236090	8.337934	4.981267
C	6.923539	7.644130	6.177822
H	6.295417	8.085075	6.940087
C	8.099280	7.664235	4.080234
H	8.401224	8.110252	3.141679
C	8.587731	6.381871	4.397299
H	9.244899	5.871367	3.707910
C	7.461460	6.362446	6.410040
H	7.228862	5.837654	7.325244
C	3.456615	3.155082	-2.912713
H	4.385216	3.163825	-3.465741
C	3.480093	3.168685	-1.504760
H	4.442497	3.178373	-1.009390
C	1.150442	3.112900	-2.953675
H	0.243612	3.084643	-3.540820
C	1.075870	3.127413	-1.545983
H	0.099000	3.103066	-1.080724
C	2.265101	3.153818	-0.773804
C	2.244229	3.163492	0.654198
C	1.089356	3.173182	2.608590
C	3.358800	3.201732	2.631510
C	4.591305	3.224591	3.351151
C	4.637671	3.186298	4.767220
H	3.734684	3.114058	5.359158
C	5.843798	3.285832	2.689826
H	5.916413	3.307377	1.610533
C	7.034941	3.317792	3.439659
H	7.987630	3.366583	2.932391
C	5.879594	3.223216	5.432060
H	5.912671	3.191212	6.511475
C	-0.156830	3.192417	3.305393
C	-1.394665	3.105513	2.620438
H	-1.442587	3.015554	1.543096
C	-2.602318	3.109215	3.343576
H	-3.544509	3.039322	2.818639
C	-1.489762	3.289148	5.357872
H	-1.548796	3.360472	6.434459
C	-0.232806	3.290369	4.718507
H	0.658856	3.360593	5.328312
C	-3.947629	13.743556	0.711091
H	-3.721128	14.783172	0.523055
C	-4.910179	11.782933	-0.025531
H	-5.453416	11.261921	-0.801582
C	-4.552928	11.104170	1.157216
H	-4.841623	10.066530	1.264741
C	-3.857060	11.790414	2.183691
C	-3.553259	13.151344	1.925711
H	-3.036576	13.767069	2.650562
C	-3.497171	11.158232	3.411965
C	-3.509229	9.346533	4.776485
C	-2.554136	11.285540	5.472968
N	-4.462613	5.227638	5.514010
C	-3.835567	7.977354	5.020669
C	-4.486342	7.169880	4.053629

H	-4.785354	7.567750	3.092298
C	-4.774441	5.819806	4.341777
H	-5.280764	5.208679	3.607457
C	-3.515711	7.339914	6.246284
H	-3.035538	7.873325	7.056697
C	-3.841524	5.984087	6.441545
H	-3.603447	5.506086	7.380183
C	-1.831011	12.027424	6.454292
C	-1.440804	11.455839	7.690206
H	-1.711448	10.442473	7.953753
C	-0.669217	12.205860	8.598446
H	-0.369451	11.762320	9.536646
C	-0.659638	14.036479	7.207862
H	-0.355644	15.059149	7.036619
C	-1.425455	13.366702	6.234634
H	-1.689816	13.897731	5.330062
C	3.397686	6.101603	-4.984029
H	4.340480	5.602246	-5.157556
C	1.095792	6.033323	-5.088886
H	0.203916	5.480205	-5.346948
C	0.983587	7.336836	-4.567570
H	-0.005793	7.758256	-4.446977
C	3.384276	7.409682	-4.459709
H	4.331204	7.888159	-4.243382
C	2.150332	8.070452	-4.234838
C	2.083844	9.393997	-3.702121
C	0.853518	11.169708	-3.004873
C	3.119388	11.258662	-2.926117
C	-0.418533	11.793253	-2.827220
C	-1.628888	11.124079	-3.139790
H	-1.637316	10.110794	-3.520016
C	-2.861116	11.786798	-2.983735
H	-3.781520	11.276306	-3.229486
C	-1.824834	13.706919	-2.231490
H	-1.921901	14.725120	-1.881798
C	-0.547432	13.123403	-2.353001
H	0.319336	13.720289	-2.099783
C	4.319509	12.003116	-2.721311
C	4.301513	13.356783	-2.301235
H	3.373699	13.876325	-2.102319
C	5.510284	14.068034	-2.172165
H	5.493496	15.105377	-1.870269
C	5.599299	11.442232	-2.961397
H	5.718721	10.415844	-3.283360
C	6.754580	12.230480	-2.799088
H	7.727844	11.801185	-2.989759
C	11.217771	14.508998	-0.823398
H	10.804062	13.510679	-0.991457
H	12.174477	14.548284	-1.359152
H	11.460350	14.589142	0.243671
C	9.879325	16.449705	-0.158045
H	8.798211	16.419600	0.005053
H	10.361539	16.178555	0.789462
H	10.141939	17.498008	-0.347362
C	10.793138	16.313712	-2.432629
H	10.863929	17.382164	-2.191804
H	11.828581	16.018658	-2.645768
C	10.027183	16.216766	-3.775682
H	9.804422	17.247516	-4.079873
H	10.752615	15.868948	-4.522226
C	7.597627	16.199384	-4.123610
H	6.916540	16.219880	-3.268290
H	7.841229	17.243541	-4.356632
H	7.034433	15.816155	-4.983798
C	8.937168	14.249695	-4.760559
H	8.128481	14.213392	-5.501170
H	9.874168	14.284074	-5.330222
H	8.931124	13.297644	-4.222134
N	-4.508022	1.007698	5.902508
N	-6.372022	3.095875	6.720505
C	-4.401131	0.427892	4.536557
H	-3.458965	-0.110089	4.373328
H	-5.203800	-0.283707	4.305704
H	-4.463332	1.219363	3.785677
C	-3.326228	0.642501	6.728061
H	-2.588772	0.044131	6.178438
H	-2.820979	1.547514	7.077820
H	-3.587304	0.062730	7.622241
C	-5.774699	0.574550	6.562557
H	-5.564883	0.004755	7.476941
H	-6.325668	-0.131815	5.928509
C	-6.782787	1.681277	6.957903
H	-7.722640	1.450927	6.439841
H	-7.015223	1.518222	8.018173
C	-7.356230	3.795223	5.851804
H	-7.739105	4.720728	6.299583
H	-6.892262	4.061203	4.897790
H	-8.235759	3.182832	5.616416

C	-6.190569	3.819538	8.006460
H	-6.785279	4.739626	8.067427
H	-6.463912	3.217800	8.882199
H	-5.140399	4.096120	8.132700
N	9.382224	1.682732	6.086401
N	10.628890	4.197411	6.881043
N	10.248698	15.542211	-1.276767
N	8.793904	15.376624	-3.800727
N	-6.754886	14.768545	-1.743409
N	-5.048845	14.757794	-4.113075
N	5.679386	12.065242	4.121081
N	5.856515	10.171623	5.597381
N	7.106868	10.216686	3.542925
N	8.283551	5.737683	5.542296
N	2.512359	13.181829	7.897253
N	2.316684	3.121743	-3.632030
N	3.437798	3.233506	1.282495
N	1.034348	3.159622	1.256296
N	2.216663	3.195374	3.356094
N	-2.656693	3.193702	4.687382
N	7.063266	3.284409	4.787275
N	-4.621510	13.081755	-0.251159
N	-3.815179	9.850922	3.558634
N	-2.858350	10.000068	5.763287
N	-2.829196	11.915159	4.308939
N	-0.271541	13.475453	8.372357
N	2.277559	5.418886	-5.300287
N	0.852059	9.908654	-3.490536
N	1.948135	11.886218	-2.665321
N	3.252498	10.000222	-3.403490
N	6.719525	13.524644	-2.421021
N	-2.964767	13.057726	-2.546736
Pd	1.412866	14.226034	9.368534
Pd	-4.842822	13.914073	-2.164491
Pd	8.834973	3.730079	5.828985
Pd	8.475685	14.527344	-1.872888
Pd	2.354196	3.364173	-5.724637
Pd	-4.498964	3.134545	5.699929
C	2.752180	5.824376	6.005089
C	2.997175	6.293660	4.674896
C	1.849010	6.455161	3.803319
C	0.637357	6.827231	4.423502
C	0.686230	7.017319	5.857236
C	1.584290	6.201568	6.612411
H	2.884271	6.183087	1.946429
H	3.540985	5.338675	6.569003
H	3.945339	6.071341	4.194627
C	1.921012	6.394615	2.404209
C	-0.482770	7.088074	3.619658
H	-0.193589	7.429328	6.343991
H	1.413324	6.029520	7.669174
C	-0.431148	6.981225	2.234484
C	0.801263	6.630531	1.614880
H	-1.406626	7.398432	4.096047
C	-1.677158	7.278902	1.424663
C	-2.512899	6.029513	1.113105
H	-1.409628	7.774499	0.488041
H	-2.293666	7.997626	1.976300
H	-3.385621	6.275630	0.504117
H	-1.925552	5.285343	0.567993
H	-2.860222	5.567308	2.042976
C	0.965464	6.611223	0.111749
C	1.312111	8.000615	-0.447512
H	0.066236	6.227811	-0.378342
H	1.777942	5.925618	-0.148025
H	1.491649	7.931446	-1.521126
H	0.499185	8.713394	-0.285309
H	2.212810	8.402331	0.026066
C	3.171433	9.040356	3.828544
C	3.217627	8.323758	5.142077
C	2.081859	8.749347	5.872160
C	1.363066	9.742042	5.043525
H	4.184873	8.157201	5.589324
H	2.038546	8.846947	6.946090
C	1.592243	10.615292	2.646149
C	0.115251	10.421803	2.274183
C	1.913310	12.094002	2.918166
H	2.205929	10.283859	1.805983
C	-0.252863	11.295201	1.066799
H	-0.504901	10.692763	3.132675
H	-0.061431	9.368633	2.062134
C	1.529192	12.961004	1.709092
H	1.358883	12.415595	3.807484
H	2.981346	12.196417	3.147393
C	0.050768	12.772417	1.344125
H	-1.310155	11.148347	0.814612
H	0.325496	10.968594	0.191499
H	1.738428	14.012693	1.918180

H	2.148431	12.668691	0.850312
H	-0.203507	13.393719	0.479355
H	-0.571675	13.122279	2.178532
N	1.999271	9.769657	3.781048
O	0.419537	10.445670	5.349501
O	4.019254	9.021113	2.931903
O	5.362619	6.538760	2.607704
H	5.643880	6.429185	1.691127
H	5.030114	7.453570	2.653794
O	4.238975	10.956957	0.686109
H	4.272149	10.222050	1.321678
H	4.184166	11.737180	1.251096

Table5_1c_reactant_1wat

Property	Value
Charge	0
Electronic Energy, BS1 (a.u.)	-1214.016793
Thermal and entropic correction, BS1 (a.u.)	2.827257
Electronic Energy, BS2 (a.u.)	-1214.432006
Number of Imaginary Frequencies	0
Imaginary frequencies (cm-1)	None

Molecular Geometry in Cartesian Coordinates

C	8.401132	1.034535	6.890766
H	8.782109	0.583704	7.815733
H	7.631725	1.753623	7.186160
H	7.903408	0.227966	6.337584
C	9.586990	1.102222	4.747266
H	8.871228	0.287661	4.578088
H	9.415698	1.853951	3.971870
H	10.582285	0.679395	4.560950
C	10.762548	1.703236	6.815729
H	11.533234	1.196996	6.220210
H	10.695937	1.102699	7.732027
C	11.359131	3.069760	7.232627
H	12.369108	3.110548	6.804552
H	11.530138	3.014199	8.315377
C	11.371575	5.187992	5.994406
H	10.898051	5.254982	5.011306
H	11.461051	6.208853	6.386756
H	12.396211	4.833194	5.825776
C	10.140840	5.013766	8.105749
H	9.050176	4.987894	8.180952
H	10.531259	4.571091	9.030652
H	10.447300	6.067421	8.115583
N	3.137889	15.110185	10.431464
N	0.264481	15.391235	10.857808
C	3.907973	13.942434	10.935349
H	3.354304	13.019893	10.741468
H	4.893241	13.843690	10.462817
H	4.088162	13.978207	12.017096
C	3.933931	15.887336	9.444342
H	3.391369	15.960460	8.498328
H	4.137275	16.915848	9.768336
H	4.910463	15.434551	9.231834
C	2.695058	15.981453	11.559414
H	3.110948	15.629256	12.512122
H	3.096588	16.997255	11.452028
C	1.170336	16.135168	11.781278
H	0.958923	17.211884	11.754845
H	0.980809	15.851344	12.824483
C	-0.622278	16.326621	10.115242
H	-1.687143	16.088226	10.230558
H	-0.506105	17.371803	10.428246
H	-0.393663	16.291004	9.046111
C	-0.533818	14.376461	11.595575
H	-0.254632	13.371506	11.266478
H	-0.375792	14.408526	12.680858
H	-1.614342	14.485004	11.438096
N	2.376968	1.265358	-6.269861
N	2.346414	3.676515	-7.913371
C	3.607080	0.706520	-5.648659
H	3.393171	-0.017717	-4.852486
H	4.257510	0.191335	-6.366709
H	4.201502	1.512367	-5.208844
C	1.157143	0.656737	-5.675697
H	1.383044	-0.062089	-4.877937
H	0.522317	1.436778	-5.245664
H	0.542436	0.119222	-6.408597
C	2.396922	1.083985	-7.751043
H	1.545798	0.475179	-8.082205
H	3.276485	0.505870	-8.062536

C	2.384119	2.363851	-8.623144
H	3.260634	2.299387	-9.280721
H	1.530356	2.263881	-9.305628
C	1.108564	4.428488	-8.251478
H	1.311469	5.439769	-8.625993
H	0.499266	3.934045	-9.018367
H	0.476722	4.527449	-7.364342
C	3.558260	4.481590	-8.220918
H	3.321000	5.479595	-8.610538
H	4.157009	4.615328	-7.315312
H	4.212768	4.009863	-8.964615
C	-6.574709	15.753017	-0.661934
H	-7.114711	15.468913	0.249997
H	-5.515537	15.835761	-0.404288
H	-6.915620	16.764031	-0.918406
C	-7.660594	13.671160	-1.361488
H	-8.010828	13.765743	-0.325867
H	-8.561438	13.601404	-1.983898
H	-7.143654	12.711069	-1.446510
C	-7.252709	15.451836	-3.001708
H	-7.482501	16.506418	-2.802159
H	-8.212358	15.025183	-3.320645
C	-6.329726	15.435563	-4.245496
H	-6.928573	15.024186	-5.068153
H	-6.172238	16.484194	-4.528755
C	-4.960570	13.577890	-5.080036
H	-4.074899	13.613820	-5.726636
H	-4.930739	12.631606	-4.533324
H	-5.827552	13.525284	-5.750512
C	-3.884631	15.645826	-4.326490
H	-3.194539	15.311122	-5.111120
H	-4.197351	16.659891	-4.605334
H	-3.309424	15.737317	-3.400784
N	8.073701	13.646537	-0.018816
C	7.187761	14.204204	0.832798
H	6.870160	15.213665	0.613678
C	6.684812	13.543736	1.971641
H	5.988828	14.072231	2.609692
C	7.112160	12.226148	2.272453
C	8.039880	11.653137	1.365728
H	8.431228	10.654045	1.507887
C	8.481977	12.389318	0.249258
H	9.189734	11.947517	-0.437496
C	6.645554	11.521409	3.423657
C	5.412619	11.464860	5.329047
C	4.452293	12.072424	6.197701
C	3.896777	13.350635	5.931469
H	4.181633	13.922196	5.057441
C	2.961940	13.915287	6.823076
H	2.542957	14.890986	6.621265
C	3.054459	12.077744	8.210557
H	2.705745	11.588734	9.108981
C	3.996206	11.436668	7.379967
H	4.362839	10.461741	7.673035
C	6.723143	9.692878	4.769002
C	7.204205	8.371121	5.026490
C	6.885032	7.659788	6.213290
H	6.283448	8.112358	6.994566
C	8.035742	7.690852	4.100347
H	8.335845	8.146641	3.165911
C	8.493108	6.389388	4.382287
H	9.126109	5.876764	3.672407
C	7.389089	6.354912	6.408304
H	7.154953	5.817282	7.315456
C	3.473857	3.073831	-3.018905
H	4.394783	3.098458	-3.584287
C	3.516274	3.057893	-1.611099
H	4.484469	3.062414	-1.126502
C	1.167325	3.021970	-3.030782
H	0.253097	3.002538	-3.606662
C	1.111301	3.007328	-1.623327
H	0.140505	2.970025	-1.146134
C	2.310263	3.021702	-0.866524
C	2.300295	3.000376	0.560388
C	1.147039	2.982973	2.510940
C	3.417313	3.000361	2.536694
C	4.642377	3.042529	3.269755
C	4.673212	2.981832	4.686614
H	3.765885	2.866773	5.265876
C	5.899657	3.162636	2.626873
H	5.984057	3.208816	1.549199
C	7.078052	3.235049	3.395473
H	8.034695	3.331665	2.901909
C	5.903143	3.062137	5.369828
H	5.922460	3.014863	6.448441
C	-0.093089	3.046309	3.213218
C	-1.339717	3.011736	2.538396
H	-1.387688	2.918618	1.457556

C	-2.539291	3.073603	3.279105
H	-3.490956	3.042403	2.767449
C	-1.392631	3.216332	5.279480
H	-1.433473	3.300338	6.355851
C	-0.147738	3.152867	4.625747
H	0.752671	3.189411	5.225460
C	-3.962723	13.782336	0.721707
H	-3.747709	14.822973	0.524929
C	-4.913766	11.808645	0.008466
H	-5.459871	11.276732	-0.758090
C	-4.539071	11.141936	1.192566
H	-4.818492	10.102705	1.309244
C	-3.839021	11.841478	2.207605
C	-3.551273	13.202784	1.936773
H	-3.032948	13.831539	2.648434
C	-3.462853	11.221087	3.437128
C	-3.454295	9.414796	4.809940
C	-2.521332	11.369463	5.498052
N	-4.319537	5.267612	5.513566
C	-3.751002	8.037200	5.046964
C	-4.400317	7.229013	4.079299
H	-4.723337	7.633241	3.127554
C	-4.661074	5.871506	4.355366
H	-5.171784	5.262653	3.622517
C	-3.396383	7.388562	6.258557
H	-2.908016	7.921024	7.065259
C	-3.689102	6.021734	6.441538
H	-3.416375	5.538730	7.371473
C	-1.802583	12.121928	6.476677
C	-1.428313	11.565674	7.725292
H	-1.707951	10.557655	8.000654
C	-0.660465	12.322548	8.631360
H	-0.374095	11.888175	9.577958
C	-0.622751	14.132823	7.215476
H	-0.309716	15.151096	7.034204
C	-1.383380	13.454819	6.242594
H	-1.631552	13.973481	5.326092
C	3.375176	6.081497	-5.043464
H	4.315576	5.589010	-5.246975
C	1.071136	5.995830	-5.072213
H	0.175880	5.435690	-5.302295
C	0.966408	7.298963	-4.547420
H	-0.021559	7.714103	-4.396840
C	3.368848	7.390709	-4.520728
H	4.318976	7.876859	-4.338127
C	2.137666	8.043725	-4.258137
C	2.076899	9.374141	-3.741191
C	0.852536	11.153401	-3.038691
C	3.119210	11.272370	-3.058054
C	-0.417940	11.772621	-2.832946
C	-1.632237	11.096099	-3.112273
H	-1.644077	10.079381	-3.483220
C	-2.863402	11.757149	-2.939017
H	-3.787097	11.242115	-3.162159
C	-1.819052	13.687408	-2.224674
H	-1.912681	14.707999	-1.881285
C	-0.542201	13.106531	-2.366409
H	0.326722	13.709271	-2.135461
C	4.318307	12.024745	-2.868998
C	4.295842	13.371688	-2.427936
H	3.365961	13.886326	-2.225450
C	5.502435	14.081343	-2.269458
H	5.480372	15.112052	-1.945683
C	5.601149	11.473760	-3.116423
H	5.724792	10.454275	-3.459067
C	6.754604	12.259014	-2.920929
H	7.730321	11.834383	-3.110212
C	11.171670	14.494408	-0.748573
H	10.766166	13.498348	-0.947126
H	12.147433	14.542291	-1.248116
H	11.374615	14.558758	0.327759
C	9.814818	16.430237	-0.105740
H	8.728522	16.405103	0.018761
H	10.262224	16.145635	0.854842
H	10.088575	17.479325	-0.273693
C	10.802427	16.316935	-2.350210
H	10.887946	17.378773	-2.085994
H	11.836452	16.006966	-2.548492
C	10.061850	16.263670	-3.709267
H	9.856247	17.304594	-3.990042
H	10.797896	15.927593	-4.450789
C	7.636966	16.285516	-4.090097
H	6.944755	16.283164	-3.243525
H	7.894667	17.334361	-4.283445
H	7.081442	15.938211	-4.970175
C	8.964967	14.345346	-4.777127
H	8.167696	14.345559	-5.530873
H	9.910767	14.388612	-5.331445

H	8.939720	13.374635	-4.273791
N	-4.447606	1.041816	5.883648
N	-6.237695	3.173250	6.754355
C	-4.388216	0.463831	4.513574
H	-3.463610	-0.097203	4.326047
H	-5.214088	-0.226675	4.299640
H	-4.448672	1.259654	3.767283
C	-3.255859	0.644693	6.680200
H	-2.545759	0.031056	6.110868
H	-2.721240	1.536221	7.021215
H	-3.509928	0.067932	7.578453
C	-5.709319	0.638343	6.572256
H	-5.492204	0.060231	7.479869
H	-6.293146	-0.051750	5.948968
C	-6.679688	1.768713	6.995266
H	-7.637723	1.565032	6.498469
H	-6.891136	1.606802	8.060442
C	-7.225753	3.902438	5.914125
H	-7.574583	4.834239	6.377383
H	-6.779494	4.163217	4.950061
H	-8.126111	3.313372	5.696610
C	-6.004565	3.884434	8.039868
H	-6.571075	4.821053	8.119705
H	-6.274130	3.285991	8.919297
H	-4.943406	4.129582	8.142513
N	9.455749	1.718025	6.095440
N	10.580284	4.293479	6.881227
N	10.218823	15.533839	-1.221760
N	8.819824	15.439263	-3.779850
N	-6.752064	14.776048	-1.769026
N	-5.027506	14.715960	-4.124749
N	5.744055	12.150745	4.212189
N	5.871549	10.237259	5.671085
N	7.132648	10.277406	3.621721
N	8.187244	5.727760	5.516027
N	2.547494	13.300211	7.949459
N	2.323837	3.050207	-3.723198
N	3.496944	3.060033	1.187599
N	1.089927	2.989078	1.161008
N	2.273835	2.971018	3.255351
N	-2.570536	3.171031	4.626370
N	7.091520	3.185549	4.744101
N	-4.638674	13.108062	-0.230481
N	-3.768974	9.912497	3.591862
N	-2.825390	10.085407	5.800008
N	-2.792976	11.987579	4.325670
N	-0.250943	13.586254	8.392598
N	2.250086	5.391135	-5.324208
N	0.847789	9.881060	-3.494957
N	1.951931	11.886009	-2.752469
N	3.249659	10.004725	-3.510605
N	6.715833	13.544223	-2.511486
N	-2.962648	13.031973	-2.512615
Pd	1.425499	14.343177	9.404992
Pd	-4.840126	13.906497	-2.159401
Pd	8.821046	3.736908	5.810790
Pd	8.464058	14.525106	-1.886944
Pd	2.329684	3.347937	-5.806580
Pd	-4.389296	3.168636	5.685945
C	2.434783	5.449119	6.158306
C	2.734375	5.750134	4.836037
C	1.687786	6.155970	3.950055
C	0.402151	6.468831	4.495185
C	0.212367	6.390947	5.911800
C	1.161506	5.785037	6.703142
H	2.865085	6.031986	2.148513
H	3.196079	5.053012	6.816764
H	3.735835	5.642607	4.424217
C	1.867947	6.219669	2.542483
C	-0.638917	6.809124	3.599369
H	-0.713582	6.769707	6.346590
H	0.968035	5.641517	7.759422
C	-0.473490	6.807602	2.227433
C	0.817448	6.499025	1.681169
H	-1.610040	7.061641	4.015482
C	-1.653924	7.154063	1.343038
C	-2.461721	5.913067	0.932478
H	-1.316882	7.695308	0.453713
H	-2.316631	7.845253	1.880139
H	-3.301593	6.173732	0.280547
H	-1.847790	5.175857	0.404213
H	-2.861496	5.426722	1.829134
C	1.078520	6.543520	0.185812
C	1.434076	7.946915	-0.332325
H	0.214194	6.156813	-0.370286
H	1.919176	5.878218	-0.038785
H	1.709628	7.896484	-1.391434
H	0.587689	8.631852	-0.236110

H	2.280877	8.364997	0.223463
C	3.177540	9.067632	4.115884
C	3.250291	8.552340	5.525000
C	2.148769	8.989805	6.196259
C	1.354385	9.839127	5.244814
H	4.108616	8.069340	5.860765
H	1.906789	8.909934	7.222618
C	1.631680	10.610710	2.816155
C	0.177933	10.360609	2.385046
C	1.900717	12.103942	3.070476
H	2.291875	10.259272	2.017325
C	-0.179662	11.214010	1.157757
H	-0.489041	10.615840	3.216703
H	0.051607	9.295308	2.181889
C	1.510344	12.954991	1.852532
H	1.315331	12.413128	3.942287
H	2.959649	12.244117	3.313060
C	0.054910	12.704448	1.437735
H	-1.220812	11.023699	0.869045
H	0.449419	10.917492	0.306114
H	1.670343	14.014931	2.067143
H	2.168421	12.701871	1.009355
H	-0.190825	13.309449	0.558169
H	-0.613518	13.029267	2.247256
N	2.008179	9.808694	3.995978
O	0.374271	10.501557	5.499395
O	4.032174	8.910070	3.260175
O	5.148995	6.386408	2.591631
H	5.464771	6.418085	1.679220
H	4.745865	7.260982	2.728371

Table5_1c_TSiii_TSi-ii_h2o

Property	Value
Charge	0
Electronic Energy, BS1 (a.u.)	-1213.976762
Thermal and entropic correction, BS1 (a.u.)	2.831730
Electronic Energy, BS2 (a.u.)	-1214.388474
Number of Imaginary Frequencies	0
Imaginary frequencies (cm-1)	None

Molecular Geometry in Cartesian Coordinates

C	8.400712	1.058841	6.906613
H	8.783551	0.610562	7.831983
H	7.642716	1.789703	7.202182
H	7.888666	0.254339	6.364005
C	9.568811	1.095605	4.753055
H	8.841460	0.289167	4.596050
H	9.400358	1.843962	3.973628
H	10.556901	0.658847	4.561628
C	10.769059	1.697291	6.807431
H	11.528763	1.177595	6.209681
H	10.702390	1.104173	7.728544
C	11.384748	3.059515	7.210193
H	12.391830	3.085769	6.774578
H	11.562956	3.009369	8.292005
C	11.412605	5.168832	5.957364
H	10.932292	5.234873	4.977489
H	11.516983	6.191012	6.342093
H	12.431589	4.800962	5.783543
C	10.194470	5.023523	8.077807
H	9.104150	5.010183	8.160167
H	10.586223	4.583385	9.003331
H	10.512211	6.073724	8.077697
N	3.135573	14.989127	10.420214
N	0.267696	15.291612	10.867196
C	3.898435	13.809049	10.905787
H	3.335559	12.893620	10.704381
H	4.879848	13.707403	10.426055
H	4.085512	13.830810	11.986706
C	3.932142	15.770225	9.436628
H	3.384097	15.858910	8.495168
H	4.146953	16.793124	9.770690
H	4.903087	15.311092	9.212556
C	2.707666	15.852109	11.560139
H	3.126283	15.485983	12.506372
H	3.117687	16.865314	11.460990
C	1.185790	16.017595	11.792782
H	0.984210	17.096409	11.778651
H	0.999800	15.724737	12.834088
C	-0.614927	16.242301	10.139452
H	-1.681187	16.012307	10.258602
H	-0.487445	17.283161	10.462259

H	-0.392723	16.215123	9.068703
C	-0.535335	14.276587	11.599396
H	-0.267200	13.272655	11.258348
H	-0.370683	14.296047	12.683969
H	-1.615729	14.396394	11.449453
N	2.402750	1.250038	-6.170191
N	2.396336	3.632715	-7.854833
C	3.624908	0.700203	-5.525996
H	3.400970	-0.009922	-4.720117
H	4.282332	0.171905	-6.227751
H	4.215847	1.512546	-5.093595
C	1.175425	0.653460	-5.579420
H	1.391212	-0.052076	-4.767342
H	0.537444	1.441578	-5.169619
H	0.567755	0.104692	-6.309741
C	2.438799	1.043443	-7.647655
H	1.590143	0.430751	-7.977905
H	3.320525	0.458478	-7.939242
C	2.438833	2.308164	-8.541575
H	3.322623	2.230588	-9.187761
H	1.592697	2.198440	-9.231970
C	1.164689	4.381807	-8.220503
H	1.374785	5.385894	-8.609803
H	0.563150	3.875819	-8.985925
H	0.522727	4.497685	-7.342850
C	3.613790	4.429653	-8.161261
H	3.383772	5.421396	-8.570393
H	4.201827	4.577413	-7.250944
H	4.275994	3.943974	-8.888798
C	-6.551473	15.766449	-0.660072
H	-7.092079	15.478766	0.250356
H	-5.491663	15.842424	-0.402755
H	-6.887848	16.780716	-0.909495
C	-7.647197	13.694235	-1.372423
H	-7.996002	13.783446	-0.335865
H	-8.548955	13.632537	-1.994314
H	-7.134585	12.732534	-1.464365
C	-7.233167	15.483655	-3.001266
H	-7.458291	16.537874	-2.794698
H	-8.194928	15.063139	-3.321956
C	-6.311546	15.471249	-4.246145
H	-6.912937	15.067591	-5.070742
H	-6.149922	16.520949	-4.522950
C	-4.950666	13.613046	-5.093558
H	-4.065471	13.649420	-5.740770
H	-4.923981	12.663327	-4.552600
H	-5.818521	13.568005	-5.763448
C	-3.865852	15.671996	-4.328369
H	-3.177797	15.339385	-5.115669
H	-4.174751	16.688958	-4.600732
H	-3.289457	15.755584	-3.402707
N	8.053745	13.651258	-0.031455
C	7.155921	14.197692	0.815046
H	6.837484	15.208316	0.602550
C	6.640558	13.523078	1.940067
H	5.934460	14.041863	2.575017
C	7.068722	12.203556	2.231280
C	8.009243	11.643115	1.330432
H	8.402172	10.643737	1.467000
C	8.462383	12.392482	0.227884
H	9.179633	11.960140	-0.455049
C	6.592894	11.483784	3.368844
C	5.344975	11.397544	5.262706
C	4.387365	11.993264	6.140521
C	3.843366	13.280127	5.896379
H	4.130561	13.863430	5.030967
C	2.921120	13.839924	6.802123
H	2.512003	14.823301	6.618101
C	3.000931	11.978884	8.160324
H	2.651575	11.480556	9.053448
C	3.929095	11.339855	7.312890
H	4.286385	10.356243	7.587757
C	6.677284	9.647303	4.698828
C	7.187699	8.340540	4.965381
C	6.879320	7.637670	6.157722
H	6.261431	8.076786	6.929260
C	8.039467	7.669721	4.051221
H	8.334551	8.121039	3.113003
C	8.518487	6.379886	4.350341
H	9.161460	5.868431	3.648635
C	7.409438	6.349829	6.373453
H	7.179124	5.817241	7.284590
C	3.468881	3.115580	-2.938713
H	4.395325	3.130783	-3.495201
C	3.497507	3.124780	-1.530808
H	4.461448	3.138336	-1.038396
C	1.162902	3.062211	-2.970847
H	0.254045	3.031658	-3.554641

C	1.093324	3.071781	-1.563023
H	0.118274	3.041617	-1.094152
C	2.285348	3.100432	-0.795433
C	2.269643	3.102508	0.632554
C	1.121558	3.104632	2.590387
C	3.390998	3.130477	2.605714
C	4.624223	3.153495	3.323547
C	4.671962	3.097672	4.738834
H	3.770540	3.007733	5.330667
C	5.875281	3.235459	2.662173
H	5.946247	3.273401	1.583224
C	7.066230	3.273567	3.412153
H	8.017852	3.340795	2.904599
C	5.913726	3.140481	5.402917
H	5.947159	3.094738	6.481636
C	-0.120817	3.143177	3.292750
C	-1.363367	3.072370	2.614813
H	-1.417972	2.976146	1.538319
C	-2.566760	3.103763	3.344552
H	-3.512862	3.047460	2.825063
C	-1.440299	3.281862	5.351147
H	-1.491068	3.367004	6.427035
C	-0.187427	3.252586	4.705328
H	0.708461	3.314513	5.309673
C	-3.944672	13.774077	0.706728
H	-3.722419	14.814279	0.516307
C	-4.910819	11.811584	-0.018684
H	-5.460685	11.288644	-0.788681
C	-4.542845	11.134953	1.161557
H	-4.830666	10.097516	1.272952
C	-3.839405	11.823422	2.181190
C	-3.538572	13.184025	1.918583
H	-3.017254	13.801511	2.638667
C	-3.474534	11.194198	3.409340
C	-3.499319	9.389917	4.782757
C	-2.543424	11.330274	5.474170
N	-4.393656	5.258328	5.513111
C	-3.812377	8.017790	5.026747
C	-4.456240	7.204315	4.060080
H	-4.764764	7.601079	3.101298
C	-4.722882	5.849258	4.345120
H	-5.222615	5.232507	3.610969
C	-3.476613	7.381343	6.248727
H	-2.996308	7.919193	7.056024
C	-3.777534	6.019305	6.440251
H	-3.520359	5.540193	7.373385
C	-1.821501	12.071792	6.456731
C	-1.456617	11.505723	7.702862
H	-1.742643	10.497621	7.970834
C	-0.689045	12.252759	8.616115
H	-0.408203	11.810764	9.560884
C	-0.636225	14.071984	7.211802
H	-0.316691	15.089531	7.038285
C	-1.394149	13.403321	6.231100
H	-1.636055	13.928505	5.316684
C	3.393978	6.084952	-5.019016
H	4.336161	5.589859	-5.207176
C	1.090098	5.997264	-5.064645
H	0.197096	5.432891	-5.293112
C	0.980700	7.306923	-4.558202
H	-0.008629	7.722624	-4.419013
C	3.383025	7.401123	-4.515351
H	4.331291	7.890256	-4.330941
C	2.149692	8.055717	-4.269520
C	2.086157	9.389948	-3.764086
C	0.858983	11.167512	-3.064287
C	3.124832	11.288041	-3.078300
C	-0.411120	11.785317	-2.855891
C	-1.625791	11.110798	-3.138361
H	-1.638871	10.095840	-3.514183
C	-2.856046	11.772611	-2.962113
H	-3.780571	11.260182	-3.187752
C	-1.809008	13.697793	-2.236830
H	-1.901462	14.716442	-1.887423
C	-0.533334	13.116507	-2.381765
H	0.336249	13.716686	-2.146725
C	4.321787	12.042016	-2.886901
C	4.296484	13.389180	-2.446323
H	3.366019	13.903659	-2.245444
C	5.501733	14.099797	-2.285954
H	5.478088	15.130324	-1.961791
C	5.605450	11.492517	-3.132716
H	5.730320	10.473140	-3.475282
C	6.757568	12.279336	-2.936030
H	7.734189	11.856141	-3.123766
C	11.158084	14.511000	-0.731304
H	10.755488	13.515390	-0.937746
H	12.137273	14.562840	-1.223701

H	11.353354	14.570269	0.346746
C	9.793435	16.441186	-0.088249
H	8.706242	16.413705	0.027964
H	10.233929	16.151990	0.874108
H	10.066937	17.491517	-0.248591
C	10.797545	16.340920	-2.326331
H	10.879548	17.401556	-2.056255
H	11.833435	16.033432	-2.518650
C	10.066841	16.293215	-3.691027
H	9.862129	17.335245	-3.968318
H	10.808464	15.961363	-4.428890
C	7.644632	16.314868	-4.088120
H	6.946741	16.307443	-3.246285
H	7.902657	17.364939	-4.274232
H	7.095305	15.971710	-4.973701
C	8.978696	14.379329	-4.776232
H	8.186355	14.382757	-5.535168
H	9.928014	14.426274	-5.324209
H	8.950936	13.405989	-4.278038
N	-4.477318	1.041487	5.926541
N	-6.314626	3.151800	6.747189
C	-4.385285	0.453801	4.562988
H	-3.449578	-0.094236	4.396284
H	-5.196372	-0.251005	4.340991
H	-4.444707	1.242339	3.808853
C	-3.293718	0.669313	6.746131
H	-2.565015	0.062358	6.194337
H	-2.778580	1.571527	7.088483
H	-3.554118	0.095763	7.644500
C	-5.743739	0.624050	6.597072
H	-5.533371	0.056772	7.512885
H	-6.305852	-0.079986	5.970251
C	-6.738187	1.742540	6.993638
H	-7.683727	1.519268	6.482834
H	-6.965173	1.586577	8.056147
C	-7.298348	3.857530	5.883105
H	-7.669717	4.787758	6.330764
H	-6.838987	4.116469	4.925048
H	-8.184984	3.252453	5.655599
C	-6.115942	3.878687	8.028749
H	-6.698765	4.806338	8.089333
H	-6.391044	3.284133	8.908860
H	-5.061574	4.142749	8.147071
N	9.457011	1.722838	6.097425
N	10.617603	4.289629	6.855889
N	10.207004	15.551388	-1.205959
N	8.826144	15.468021	-3.774319
N	-6.734209	14.797787	-1.773504
N	-5.012227	14.745451	-4.131266
N	5.682997	12.100019	4.157809
N	5.808140	10.170649	5.595785
N	7.088379	10.241977	3.558157
N	8.218729	5.728107	5.491749
N	2.508944	13.212098	7.921894
N	2.326376	3.078779	-3.653698
N	3.465516	3.171490	1.256717
N	1.061508	3.092174	1.238420
N	2.251434	3.114178	3.333622
N	-2.612426	3.202333	4.687849
N	7.096709	3.225165	4.759633
N	-4.626000	13.110373	-0.249196
N	-3.798511	9.888826	3.561199
N	-2.868633	10.052759	5.775463
N	-2.801518	11.952280	4.302004
N	-0.272773	13.515732	8.386703
N	2.271411	5.390072	-5.297667
N	0.855808	9.896516	-3.523395
N	1.956868	11.900269	-2.774754
N	3.257881	10.020671	-3.529945
N	6.716063	13.564269	-2.526277
N	-2.953017	13.045365	-2.528818
Pd	1.410313	14.248379	9.396975
Pd	-4.826407	13.922951	-2.171365
Pd	8.845866	3.746279	5.801880
Pd	8.458789	14.543881	-1.888723
Pd	2.353402	3.340003	-5.743164
Pd	-4.448752	3.166701	5.712531
C	2.661441	5.660948	6.058115
C	2.942124	6.170560	4.747151
C	1.812025	6.359993	3.854216
C	0.592798	6.728424	4.460562
C	0.623554	6.899731	5.901193
C	1.486787	6.035571	6.650565
H	2.874111	6.110331	2.009662
H	3.429541	5.142584	6.621799
H	3.899182	5.955752	4.281408
C	1.904167	6.317261	2.455199
C	-0.513545	7.002790	3.644624

H	-0.266465	7.299422	6.380044
H	1.285190	5.830697	7.696253
C	-0.443711	6.910560	2.258384
C	0.794840	6.562665	1.651686
H	-1.442406	7.312861	4.110589
C	-1.678429	7.225784	1.437517
C	-2.527698	5.988808	1.113502
H	-1.396605	7.720533	0.504882
H	-2.290277	7.951434	1.985667
H	-3.393359	6.247690	0.499539
H	-1.945480	5.239782	0.569446
H	-2.888163	5.526558	2.038277
C	0.973661	6.547118	0.148296
C	1.304636	7.939800	-0.414381
H	0.084447	6.147531	-0.347942
H	1.799678	5.874025	-0.101766
H	1.515583	7.874294	-1.484167
H	0.475328	8.638540	-0.275359
H	2.184407	8.354740	0.085735
C	3.158199	8.922931	3.988125
C	3.157817	8.192277	5.293130
C	1.986757	8.594341	5.986869
C	1.301257	9.610454	5.147528
H	4.108572	8.026310	5.776233
H	1.916972	8.692918	7.060097
C	1.631841	10.530593	2.782689
C	0.179169	10.316053	2.331662
C	1.910161	12.009012	3.100047
H	2.302495	10.209832	1.978936
C	-0.164727	11.218660	1.139462
H	-0.484850	10.541678	3.170844
H	0.038602	9.266678	2.079023
C	1.542430	12.907467	1.909840
H	1.319018	12.289788	3.980034
H	2.968546	12.129053	3.353618
C	0.086958	12.693975	1.473440
H	-1.207549	11.052598	0.841260
H	0.454412	10.934956	0.276270
H	1.716072	13.956203	2.164824
H	2.204508	12.675183	1.064345
H	-0.145876	13.333304	0.615050
H	-0.580939	13.000621	2.289680
N	1.987883	9.670483	3.922612
O	0.335957	10.294754	5.434057
O	4.008775	8.903894	3.105198
O	5.320633	6.452248	2.644804
H	5.586744	6.416353	1.717605
H	4.990238	7.362887	2.763477

Table5_1c_TSiii_DG_h2o

Property	Value
Charge	0
Electronic Energy, BS1 (a.u.)	-1214.020153
Thermal and entropic correction, BS1 (a.u.)	2.839546
Electronic Energy, BS2 (a.u.)	-1214.429460
Number of Imaginary Frequencies	0
Imaginary frequencies (cm-1)	None

Molecular Geometry in Cartesian Coordinates

C	8.401547	1.059228	6.907188
H	8.784435	0.611490	7.832800
H	7.643164	1.789857	7.202349
H	7.889950	0.254280	6.364818
C	9.569845	1.095772	4.753739
H	8.842916	0.288904	4.596972
H	9.401010	1.843790	3.974071
H	10.558170	0.659475	4.562476
C	10.769655	1.698574	6.808067
H	11.529523	1.178653	6.210719
H	10.703033	1.106027	7.729550
C	11.385033	3.061184	7.209998
H	12.391984	3.087556	6.774092
H	11.563554	3.011596	8.291784
C	11.411947	5.170113	5.956455
H	10.931625	5.235457	4.976535
H	11.515735	6.192530	6.340716
H	12.431144	4.802730	5.782854
C	10.194107	5.025022	8.077077
H	9.103801	5.011365	8.159536
H	10.586049	4.585283	9.002711
H	10.511540	6.075315	8.076632
N	3.135948	14.988859	10.419666

N	0.268205	15.291276	10.867660
C	3.899313	13.808784	10.904459
H	3.336452	12.893312	10.703216
H	4.880418	13.707407	10.424040
H	4.087120	13.830320	11.985256
C	3.931859	15.770440	9.435938
H	3.383390	15.859151	8.494728
H	4.146474	16.793334	9.770134
H	4.902859	15.311670	9.211369
C	2.708420	15.851358	11.560102
H	3.127327	15.484834	12.506054
H	3.118437	16.864595	11.461249
C	1.186621	16.016799	11.793290
H	0.985083	17.095626	11.779703
H	1.000943	15.723496	12.834527
C	-0.614298	16.242323	10.140250
H	-1.680601	16.012664	10.259675
H	-0.486380	17.283115	10.463100
H	-0.392396	16.215149	9.069436
C	-0.534968	14.276272	11.599741
H	-0.267040	13.272355	11.258496
H	-0.370240	14.295486	12.684309
H	-1.615350	14.396314	11.449886
N	2.402810	1.249933	-6.170113
N	2.396566	3.632830	-7.854426
C	3.624968	0.699965	-5.526032
H	3.401030	-0.010254	-4.720236
H	4.282343	0.171728	-6.227879
H	4.215957	1.512227	-5.093547
C	1.175483	0.653334	-5.579365
H	1.391279	-0.052333	-4.767404
H	0.537574	1.441428	-5.169402
H	0.567739	0.104708	-6.309731
C	2.438803	1.043530	-7.647607
H	1.590093	0.430951	-7.977925
H	3.320478	0.458533	-7.939289
C	2.438927	2.308371	-8.541356
H	3.322689	2.230801	-9.187579
H	1.592759	2.198819	-9.231739
C	1.165003	4.382098	-8.220016
H	1.375207	5.386214	-8.609185
H	0.563423	3.876269	-8.985511
H	0.523041	4.497931	-7.342359
C	3.614107	4.429690	-8.160707
H	3.384195	5.421501	-8.569731
H	4.202116	4.577283	-7.250343
H	4.276305	3.944044	-8.888271
C	-6.550825	15.766762	-0.659679
H	-7.091270	15.479039	0.250832
H	-5.490972	15.842735	-0.402521
H	-6.887277	16.781018	-0.909042
C	-7.646731	13.694618	-1.371945
H	-7.995481	13.783872	-0.335373
H	-8.548518	13.632936	-1.993797
H	-7.134179	12.732890	-1.463890
C	-7.232683	15.483999	-3.000830
H	-7.457719	16.538234	-2.794249
H	-8.194492	15.063556	-3.321473
C	-6.311133	15.471510	-4.245761
H	-6.912615	15.067892	-5.070310
H	-6.149449	16.521194	-4.522595
C	-4.950444	13.613192	-5.093221
H	-4.065309	13.649507	-5.740518
H	-4.923784	12.663482	-4.552253
H	-5.818350	13.568172	-5.763044
C	-3.865418	15.672077	-4.328155
H	-3.177467	15.339399	-5.115518
H	-4.174267	16.689067	-4.600472
H	-3.288924	15.755606	-3.402545
N	8.053388	13.651262	-0.032049
C	7.155442	14.197684	0.814335
H	6.837097	15.208340	0.601861
C	6.639848	13.523022	1.939228
H	5.933660	14.041798	2.574087
C	7.067917	12.203469	2.230440
C	8.008523	11.643029	1.329676
H	8.401405	10.643634	1.466247
C	8.461896	12.392442	0.227260
H	9.179246	11.960109	-0.455571
C	6.592031	11.483688	3.367980
C	5.344168	11.397452	5.261892
C	4.386653	11.993149	6.139825
C	3.842794	13.280116	5.895930
H	4.130023	13.863518	5.030596
C	2.920655	13.839862	6.801830
H	2.511621	14.823312	6.618004
C	3.000280	11.978551	8.159665
H	2.650869	11.480083	9.052692

C	3.928346	11.339580	7.312089
H	4.285499	10.355866	7.586767
C	6.676346	9.647148	4.697894
C	7.186791	8.340406	4.964493
C	6.878510	7.637638	6.156921
H	6.260728	8.076832	6.928509
C	8.038489	7.669516	4.050318
H	8.333512	8.120775	3.112051
C	8.517482	6.379675	4.349477
H	9.160344	5.868127	3.647737
C	7.408588	6.349793	6.372685
H	7.178303	5.817256	7.283858
C	3.468906	3.114907	-2.938364
H	4.395374	3.129874	-3.494818
C	3.497491	3.124237	-1.530461
H	4.461422	3.137662	-1.038024
C	1.162924	3.061990	-2.970570
H	0.254080	3.031553	-3.554392
C	1.093301	3.071719	-1.562747
H	0.118231	3.041791	-1.093901
C	2.285304	3.100207	-0.795122
C	2.269557	3.102423	0.632863
C	1.121429	3.105039	2.590672
C	3.390870	3.130459	2.606050
C	4.624108	3.153214	3.323862
C	4.671864	3.097992	4.739167
H	3.770432	3.008814	5.331085
C	5.875185	3.234279	2.662420
H	5.946143	3.271677	1.583452
C	7.066170	3.272153	3.412348
H	8.017796	3.338694	2.904716
C	5.913677	3.140479	5.403181
H	5.947136	3.095166	6.481919
C	-0.120977	3.143795	3.292971
C	-1.363490	3.073184	2.614949
H	-1.418047	2.976934	1.538455
C	-2.566923	3.104848	3.344598
H	-3.513000	3.048737	2.825044
C	-1.440586	3.282758	5.351273
H	-1.491419	3.367947	6.427157
C	-0.187671	3.253228	4.705544
H	0.708189	3.314990	5.309945
C	-3.944169	13.774061	0.707040
H	-3.721792	14.814237	0.516628
C	-4.910516	11.811688	-0.018439
H	-5.460400	11.288820	-0.788476
C	-4.542687	11.135000	1.161817
H	-4.830625	10.097591	1.273184
C	-3.839232	11.823384	2.181498
C	-3.538190	13.183945	1.918907
H	-3.016804	13.801352	2.639012
C	-3.474532	11.194125	3.409679
C	-3.499565	9.389841	4.783090
C	-2.543449	11.330079	5.474534
N	-4.394861	5.258452	5.513570
C	-3.812998	8.017810	5.027136
C	-4.456973	7.204426	4.060469
H	-4.765347	7.601204	3.101643
C	-4.723894	5.849431	4.345547
H	-5.223722	5.232761	3.611396
C	-3.477482	7.381350	6.249172
H	-2.997167	7.919142	7.056503
C	-3.778687	6.019378	6.440719
H	-3.521713	5.540265	7.373906
C	-1.821663	12.071611	6.457179
C	-1.456636	11.505462	7.703228
H	-1.742443	10.497269	7.971082
C	-0.689108	12.252514	8.616508
H	-0.408090	11.810432	9.561186
C	-0.636724	14.071926	7.212443
H	-0.317406	15.089562	7.039044
C	-1.394565	13.403243	6.231693
H	-1.636590	13.928488	5.317343
C	3.394181	6.084719	-5.018423
H	4.336345	5.589586	-5.206569
C	1.090290	5.997053	-5.063961
H	0.197277	5.432657	-5.292332
C	0.980927	7.306757	-4.557630
H	-0.008390	7.722485	-4.418449
C	3.383259	7.400932	-4.514863
H	4.331535	7.890073	-4.330526
C	2.149938	8.055556	-4.269040
C	2.086431	9.389828	-3.763713
C	0.859293	11.167467	-3.064044
C	3.125136	11.287993	-3.078201
C	-0.410785	11.785327	-2.855665
C	-1.625488	11.110822	-3.138029
H	-1.638611	10.095834	-3.513768

C	-2.855721	11.772681	-2.961772
H	-3.780270	11.260252	-3.187315
C	-1.808593	13.697886	-2.236690
H	-1.901009	14.716557	-1.887337
C	-0.532941	13.116562	-2.381654
H	0.336667	13.716742	-2.146702
C	4.322078	12.042062	-2.887102
C	4.296744	13.389322	-2.446843
H	3.366271	13.903785	-2.245970
C	5.501959	14.100065	-2.286813
H	5.478276	15.130668	-1.962900
C	5.605739	11.492602	-3.132985
H	5.730628	10.473154	-3.475334
C	6.757821	12.279572	-2.936678
H	7.734437	11.856413	-3.124514
C	11.157881	14.510829	-0.730939
H	10.755279	13.515276	-0.937654
H	12.137271	14.562654	-1.222937
H	11.352704	14.569955	0.347199
C	9.793271	16.441178	-0.088264
H	8.706071	16.413649	0.027809
H	10.233669	16.152094	0.874170
H	10.066685	17.491525	-0.248643
C	10.798021	16.340805	-2.326056
H	10.880393	17.401370	-2.055815
H	11.833821	16.032975	-2.518306
C	10.067436	16.293583	-3.690836
H	9.862706	17.335709	-3.967746
H	10.809131	15.962051	-4.428772
C	7.645364	16.315159	-4.088665
H	6.947222	16.307705	-3.247043
H	7.903396	17.365250	-4.274647
H	7.096305	15.972022	-4.974423
C	8.979701	14.379690	-4.776489
H	8.187667	14.383228	-5.535745
H	9.929260	14.426603	-5.324051
H	8.951663	13.406319	-4.278363
N	-4.476720	1.041472	5.926023
N	-6.314957	3.150846	6.747019
C	-4.384256	0.454111	4.562358
H	-3.448236	-0.093378	4.395613
H	-5.194943	-0.251095	4.340162
H	-4.444062	1.242765	3.808373
C	-3.293067	0.669628	6.745689
H	-2.563992	0.063162	6.193846
H	-2.778406	1.571987	7.088374
H	-3.553342	0.095712	7.643862
C	-5.743050	0.623354	6.596303
H	-5.532557	0.055913	7.511986
H	-6.304819	-0.080733	5.969230
C	-6.737978	1.741351	6.993067
H	-7.683382	1.517827	6.482122
H	-6.964995	1.585031	8.055517
C	-7.298890	3.856442	5.883060
H	-7.670588	4.786440	6.330926
H	-6.839568	4.115760	4.925081
H	-8.185319	3.251128	5.655378
C	-6.116631	3.877477	8.028777
H	-6.699905	4.804829	8.089606
H	-6.391451	3.282551	8.908724
H	-5.062393	4.142024	8.147172
N	9.457679	1.723380	6.097900
N	10.617388	4.290889	6.855352
N	10.207063	15.551332	-1.205856
N	8.826812	15.468319	-3.774565
N	-6.733700	14.798114	-1.773092
N	-5.011850	14.745627	-4.130951
N	5.682293	12.100008	4.157070
N	5.807178	10.170464	5.594832
N	7.087381	10.241808	3.557196
N	8.217779	5.727988	5.490950
N	2.508450	13.211876	7.921505
N	2.326422	3.078261	-3.653390
N	3.465425	3.171163	1.257044
N	1.061412	3.092415	1.238703
N	2.251286	3.114580	3.333944
N	-2.612680	3.203460	4.687884
N	7.096693	3.224342	4.759848
N	-4.625540	13.110452	-0.248912
N	-3.798681	9.888796	3.561530
N	-2.868562	10.052513	5.775717
N	-2.801540	11.952148	4.302404
N	-0.273058	13.515589	8.387237
N	2.271591	5.389838	-5.296969
N	0.856090	9.896426	-3.523026
N	1.957194	11.900227	-2.774600
N	3.258173	10.020547	-3.529634
N	6.716303	13.564601	-2.527211

N	-2.952634	13.045475	-2.528581
Pd	1.410341	14.248082	9.397022
Pd	-4.825977	13.923150	-2.171054
Pd	8.845764	3.746507	5.801692
Pd	8.458925	14.544046	-1.889125
Pd	2.353558	3.339830	-5.742803
Pd	-4.449036	3.166760	5.712480
C	2.650169	5.661454	6.083317
C	2.980081	6.372471	4.787259
C	1.801031	6.374864	3.844953
C	0.596193	6.737966	4.446073
C	0.723980	7.060141	5.911145
C	1.504501	6.015849	6.655863
H	2.873462	6.110577	2.015409
H	3.388973	5.040654	6.588478
H	3.907733	6.059218	4.306987
C	1.903376	6.307631	2.460672
C	-0.514821	6.992753	3.649345
H	-0.205165	7.391509	6.384967
H	1.224426	5.717818	7.667226
C	-0.444501	6.905681	2.253354
C	0.784466	6.557814	1.651933
H	-1.439008	7.317217	4.115049
C	-1.678930	7.225928	1.437001
C	-2.528356	5.989083	1.112941
H	-1.396722	7.720469	0.504378
H	-2.290789	7.951783	1.984869
H	-3.393696	6.248000	0.498539
H	-1.946071	5.239774	0.569346
H	-2.889348	5.527152	2.037674
C	0.973490	6.547102	0.148601
C	1.304733	7.939736	-0.414036
H	0.084363	6.147572	-0.352743
H	1.799519	5.873932	-0.101221
H	1.515659	7.874208	-1.483825
H	0.475560	8.638633	-0.274989
H	2.184585	8.354486	0.086093
C	3.157546	8.897447	3.982705
C	3.122826	8.009930	5.224279
C	1.864633	8.453094	5.962588
C	1.292002	9.596432	5.152805
H	4.083623	8.157791	5.814854
H	1.970841	8.741707	7.035849
C	1.636698	10.530170	2.787332
C	0.183714	10.316422	2.336933
C	1.911148	12.008508	3.104113
H	2.306792	10.203898	1.988265
C	-0.159900	11.218761	1.144439
H	-0.484723	10.542909	3.176260
H	0.042307	9.267027	2.084799
C	1.543719	12.906777	1.908780
H	1.320417	12.289974	3.979250
H	2.969667	12.132865	3.352459
C	0.088005	12.694053	1.472784
H	-1.202919	11.048421	0.846589
H	0.453890	10.934241	0.276296
H	1.718043	13.955489	2.163413
H	2.205509	12.673762	1.063243
H	-0.144568	13.333139	0.614147
H	-0.579523	13.001562	2.289003
N	1.997605	9.670156	3.937116
O	0.342190	10.301411	5.434603
O	4.007957	8.907146	3.104552
O	5.319673	6.450433	2.644791
H	5.586589	6.414675	1.717817
H	4.994271	7.361108	2.758444

Table5_1c_reactant

Property	Value
Charge	0
Electronic Energy, BS1 (a.u.)	-1290.455014
Thermal and entropic correction, BS1 (a.u.)	2.850834
Electronic Energy, BS2 (a.u.)	-1290.908198
Number of Imaginary Frequencies	0
Imaginary frequencies (cm-1)	None

Molecular Geometry in Cartesian Coordinates

C	8.297136	1.028886	6.862178
H	8.651496	0.544286	7.780601
H	7.556047	1.773111	7.166373
H	7.771002	0.254749	6.289580
C	9.505880	1.085096	4.731353

H	8.764764	0.298012	4.543735
H	9.364793	1.856193	3.968777
H	10.488249	0.633665	4.543921
C	10.682190	1.605946	6.821783
H	11.432896	1.062169	6.234199
H	10.578198	1.010469	7.737905
C	11.340018	2.942709	7.242512
H	12.354541	2.933453	6.823852
H	11.498383	2.881683	8.326834
C	11.457111	5.041655	5.976697
H	10.981954	5.117353	4.994602
H	11.600510	6.062149	6.353359
H	12.461303	4.632599	5.809337
C	10.233227	4.960003	8.097153
H	9.143447	4.994721	8.179097
H	10.605013	4.508778	9.025558
H	10.596262	5.995370	8.091753
N	3.136140	14.989118	10.379485
N	0.268250	15.270290	10.839755
C	3.903095	13.820614	10.885933
H	3.340791	12.900485	10.705824
H	4.883063	13.711070	10.404971
H	4.093815	13.864494	11.965562
C	3.929125	15.754702	9.380961
H	3.380800	15.822569	8.437824
H	4.139561	16.784777	9.695358
H	4.902135	15.295639	9.165928
C	2.706654	15.871306	11.503958
H	3.143806	15.534602	12.452749
H	3.096485	16.889325	11.376396
C	1.185069	16.012185	11.753990
H	0.966614	17.087677	11.739811
H	1.015103	15.719031	12.797940
C	-0.629095	16.207272	10.112154
H	-1.692007	15.965293	10.237400
H	-0.512397	17.250981	10.429807
H	-0.411692	16.178675	9.040712
C	-0.519450	14.251901	11.583883
H	-0.247028	13.248552	11.243856
H	-0.344585	14.277346	12.666732
H	-1.602150	14.362720	11.444100
N	2.395953	1.270027	-6.135995
N	2.410855	3.639438	-7.838885
C	3.607909	0.718030	-5.474538
H	3.371180	0.016901	-4.664445
H	4.268843	0.178829	-6.164633
H	4.200216	1.530314	-5.044161
C	1.158897	0.685151	-5.554002
H	1.361922	-0.017484	-4.736148
H	0.522721	1.480020	-5.154385
H	0.554420	0.136800	-6.287290
C	2.447492	1.051856	-7.611323
H	1.599747	0.440459	-7.946197
H	3.329604	0.460580	-7.888660
C	2.463856	2.309708	-8.514743
H	3.357511	2.226705	-9.146475
H	1.628554	2.195377	-9.217479
C	1.179431	4.380863	-8.220518
H	1.388622	5.384281	-8.612047
H	0.587934	3.868360	-8.989461
H	0.528132	4.497421	-7.349959
C	3.627812	4.438797	-8.141339
H	3.397500	5.424839	-8.563960
H	4.205206	4.599953	-7.226366
H	4.300171	3.948150	-8.856185
C	-6.570324	15.722552	-0.616502
H	-7.099982	15.417369	0.294659
H	-5.508944	15.805156	-0.367873
H	-6.918779	16.736750	-0.848927
C	-7.654470	13.650907	-1.348109
H	-7.993961	13.721910	-0.307093
H	-8.561739	13.591289	-1.962128
H	-7.135093	12.694953	-1.459544
C	-7.268702	15.465708	-2.955601
H	-7.498171	16.515899	-2.733867
H	-8.230913	15.043357	-3.272448
C	-6.358955	15.474094	-4.209225
H	-6.964592	15.073471	-5.032212
H	-6.209047	16.527922	-4.476826
C	-4.988541	13.638655	-5.090537
H	-4.107715	13.689371	-5.742790
H	-4.951115	12.682752	-4.561283
H	-5.860238	13.595104	-5.755549
C	-3.915714	15.697714	-4.309350
H	-3.232795	15.381990	-5.107996
H	-4.235766	16.715700	-4.564427
H	-3.330678	15.773222	-3.388354
N	8.144864	13.662784	0.007703

C	7.248931	14.207131	0.857161
H	6.955410	15.229291	0.664284
C	6.706782	13.520132	1.961097
H	6.012067	14.044923	2.603327
C	7.107442	12.187724	2.229967
C	8.046992	11.627729	1.328828
H	8.422850	10.620121	1.451361
C	8.525743	12.391034	0.247080
H	9.244043	11.959182	-0.434957
C	6.617658	11.462022	3.356437
C	5.370831	11.385202	5.250172
C	4.405659	11.975248	6.123646
C	3.823359	13.240449	5.856703
H	4.085760	13.811873	4.975982
C	2.897071	13.796278	6.761522
H	2.461842	14.765394	6.562608
C	3.041420	11.969116	8.158993
H	2.716379	11.480944	9.066805
C	3.978462	11.337199	7.316201
H	4.366895	10.371360	7.611959
C	6.720940	9.640789	4.703006
C	7.235202	8.338157	4.982083
C	6.920849	7.642965	6.177360
H	6.293532	8.084000	6.940211
C	8.096636	7.663907	4.079760
H	8.399507	8.110756	3.141896
C	8.581979	6.379810	4.394534
H	9.237601	5.868736	3.704117
C	7.455729	6.359620	6.407418
H	7.221716	5.833651	7.321584
C	3.455796	3.151968	-2.914020
H	4.384350	3.161494	-3.467112
C	3.479370	3.164532	-1.506071
H	4.441815	3.174323	-1.010776
C	1.149640	3.108946	-2.954851
H	0.242783	3.080807	-3.541955
C	1.075153	3.122293	-1.547139
H	0.098332	3.097277	-1.081786
C	2.264438	3.148531	-0.775047
C	2.243715	3.157194	0.652952
C	1.089132	3.166939	2.607528
C	3.358585	3.194534	2.630043
C	4.591166	3.217245	3.349529
C	4.637744	3.175876	4.765482
H	3.734917	3.100753	5.357310
C	5.843490	3.281508	2.688223
H	5.915952	3.305589	1.608972
C	7.034642	3.313464	3.438040
H	7.987223	3.364538	2.930765
C	5.879671	3.213097	5.430275
H	5.912771	3.178706	6.509604
C	-0.156922	3.188164	3.304517
C	-1.394945	3.101722	2.619851
H	-1.443123	3.010645	1.542615
C	-2.602483	3.107517	3.343175
H	-3.544806	3.038109	2.818422
C	-1.489469	3.288834	5.357072
H	-1.548295	3.361599	6.433576
C	-0.232594	3.288020	4.717520
H	0.659229	3.358329	5.327084
C	-3.943108	13.742984	0.708361
H	-3.716288	14.782471	0.520001
C	-4.906111	11.782359	-0.027872
H	-5.449251	11.261261	-0.803934
C	-4.549375	11.103908	1.155188
H	-4.838545	10.066453	1.263073
C	-3.853705	11.790311	2.181693
C	-3.549201	13.151006	1.923271
H	-3.032606	13.766787	2.648137
C	-3.495294	11.158649	3.410673
C	-3.509869	9.347718	4.776258
C	-2.554801	11.286751	5.472745
N	-4.461982	5.228473	5.513500
C	-3.835777	7.978405	5.020302
C	-4.486004	7.170683	4.053106
H	-4.784877	7.568423	3.091678
C	-4.773716	5.820501	4.341180
H	-5.279490	5.209155	3.606663
C	-3.516135	7.341113	6.246037
H	-3.036360	7.874659	7.056576
C	-3.841482	5.985170	6.441217
H	-3.598729	5.507329	7.379936
C	-1.831291	12.028401	6.454039
C	-1.441717	11.456857	7.690182
H	-1.713222	10.443760	7.953934
C	-0.669904	12.206607	8.598476
H	-0.370838	11.763188	9.536954
C	-0.658655	14.036854	7.207377

H	-0.354027	15.059325	7.036059
C	-1.424601	13.367286	6.234072
H	-1.688463	13.898189	5.329269
C	3.396937	6.100635	-4.982905
H	4.339638	5.601279	-5.156938
C	1.094944	6.032261	-5.086427
H	0.202951	5.479049	-5.343920
C	0.983033	7.335794	-4.565105
H	-0.006308	7.757101	-4.443787
C	3.383781	7.408712	-4.458562
H	4.330804	7.887244	-4.242780
C	2.149968	8.069481	-4.233079
C	2.083738	9.393077	-3.700419
C	0.853519	11.169563	-3.005052
C	3.119385	11.258117	-2.925291
C	-0.418436	11.793746	-2.829123
C	-1.628822	11.124453	-3.141294
H	-1.637408	10.110491	-3.519726
C	-2.860896	11.787889	-2.987142
H	-3.781331	11.277375	-3.232762
C	-1.824424	13.708828	-2.237379
H	-1.921278	14.727673	-1.889506
C	-0.547157	13.124718	-2.357222
H	0.319660	13.721802	-2.104631
C	4.319507	12.002353	-2.719572
C	4.301382	13.356041	-2.299506
H	3.373514	13.875761	-2.101325
C	5.510139	14.067120	-2.169413
H	5.493293	15.104471	-1.867539
C	5.599426	11.441249	-2.958522
H	5.718938	10.414831	-3.280360
C	6.754722	12.229315	-2.795195
H	7.728106	11.799869	-2.984924
C	11.218932	14.510105	-0.824904
H	10.805114	13.511677	-0.992061
H	12.175049	14.549266	-1.361715
H	11.462639	14.590746	0.241872
C	9.881037	16.451016	-0.159053
H	8.800100	16.420954	0.005222
H	10.364286	16.180370	0.788073
H	10.143394	17.499246	-0.349140
C	10.792335	16.313963	-2.434585
H	10.862944	17.382605	-2.194551
H	11.827682	16.019124	-2.648492
C	10.025188	16.215825	-3.776867
H	9.801614	17.246276	-4.081473
H	10.750137	15.867953	-4.523856
C	7.595467	16.196777	-4.123366
H	6.914858	16.217867	-3.267685
H	7.838383	17.240817	-4.357626
H	7.031982	15.812354	-4.982829
C	8.935658	14.247096	-4.759004
H	8.126461	14.209462	-5.498994
H	9.872243	14.281436	-5.329349
H	8.930592	13.295632	-4.219516
N	-4.508305	1.008551	5.903082
N	-6.371536	3.097425	6.721005
C	-4.402070	0.428319	4.537267
H	-3.460030	-0.109804	4.373790
H	-5.204914	-0.283266	4.306985
H	-4.464539	1.219575	3.786188
C	-3.326310	0.643292	6.728317
H	-2.589207	0.044571	6.178604
H	-2.820705	1.548288	7.077610
H	-3.587207	0.063859	7.622767
C	-5.774864	0.575917	6.563686
H	-5.564903	0.006495	7.478269
H	-6.326128	-0.130632	5.930098
C	-6.782661	1.682995	6.958773
H	-7.722612	1.452759	6.440838
H	-7.015059	1.520261	8.019100
C	-7.355749	3.796899	5.852413
H	-7.738366	4.722512	6.300186
H	-6.891900	4.062716	4.898296
H	-8.235436	3.184656	5.617230
C	-6.189580	3.821254	8.006792
H	-6.783749	4.741700	8.067597
H	-6.463196	3.219857	8.882680
H	-5.139239	4.097241	8.132900
N	9.385386	1.683116	6.087959
N	10.625785	4.201852	6.879739
N	10.249262	15.542981	-1.277731
N	8.792357	15.374973	-3.800301
N	-6.754083	14.767470	-1.741349
N	-5.052265	14.758781	-4.114053
N	5.683691	12.068434	4.125600
N	5.859752	10.174045	5.601167
N	7.110772	10.219660	3.547080

N	8.276336	5.734565	5.538519
N	2.513358	13.183007	7.899592
N	2.315839	3.118736	-3.633283
N	3.437364	3.227316	1.281070
N	1.033926	3.152736	1.255241
N	2.216595	3.188190	3.354852
N	-2.656607	3.193754	4.686881
N	7.063255	3.277333	4.785600
N	-4.617033	13.081025	-0.253744
N	-3.814399	9.851664	3.557871
N	-2.860472	10.001757	5.763683
N	-2.827864	11.915756	4.307900
N	-0.271386	13.475893	8.372176
N	2.276629	5.417928	-5.298537
N	0.851998	9.907951	-3.489173
N	1.948127	11.886031	-2.665417
N	3.252450	9.999418	-3.402065
N	6.719475	13.523517	-2.417248
N	-2.964420	13.059557	-2.552253
Pd	1.412097	14.226664	9.370107
Pd	-4.842193	13.914487	-2.166198
Pd	8.833333	3.728757	5.827560
Pd	8.475679	14.527634	-1.871366
Pd	2.353177	3.363711	-5.725530
Pd	-4.498727	3.135350	5.699943
C	2.760644	5.810861	5.978453
C	2.961521	6.088944	4.641527
C	1.860273	6.442733	3.808708
C	0.639819	6.826911	4.435325
C	0.608423	6.886660	5.855469
C	1.571552	6.225316	6.605533
H	2.879466	6.168651	1.935873
H	3.566094	5.413573	6.595105
H	3.937662	5.954827	4.170949
C	1.918443	6.395133	2.395840
C	-0.477136	7.106376	3.616719
H	-0.238274	7.365349	6.342191
H	1.471232	6.140706	7.689443
C	-0.431419	6.986282	2.240515
C	0.806472	6.629297	1.613650
H	-1.401868	7.411658	4.094628
C	-1.679703	7.278785	1.429367
C	-2.517441	6.029351	1.123271
H	-1.415009	7.771848	0.490580
H	-2.293801	7.999306	1.981327
H	-3.388176	6.274981	0.518057
H	-1.932917	5.284413	0.576193
H	-2.860308	5.568353	2.055368
C	0.957225	6.602206	0.110032
C	1.302341	7.990616	-0.452664
H	0.056911	6.217783	-0.372514
H	1.769269	5.916384	-0.150555
H	1.476350	7.919261	-1.526285
H	0.489105	8.703301	-0.291601
H	2.203015	8.393899	0.019738
C	3.175627	9.068528	3.840094
C	3.250411	8.498745	5.202042
C	2.188314	8.873247	5.887981
C	1.362680	9.750275	5.031705
H	4.204404	8.039480	5.559723
H	1.985513	8.814382	6.970071
C	1.591830	10.616109	2.640284
C	0.117228	10.420461	2.259915
C	1.913972	12.094936	2.915721
H	2.210805	10.291108	1.798558
C	-0.245795	11.294884	1.056473
H	-0.503813	10.689121	3.115025
H	-0.056334	9.367276	2.045302
C	1.535112	12.962673	1.710321
H	1.354248	12.414789	3.807166
H	2.980615	12.193766	3.155497
C	0.059001	12.772233	1.337156
H	-1.301718	11.151718	0.794293
H	0.342202	10.970018	0.183764
H	1.741613	14.014433	1.921728
H	2.159535	12.672151	0.854747
H	-0.191450	13.394176	0.471762
H	-0.568461	13.120256	2.168593
N	1.989679	9.770381	3.767508
O	0.399847	10.428818	5.339287
O	4.026511	9.026004	2.941307
O	5.357659	6.539645	2.602252
H	5.638662	6.436442	1.684863
H	5.023501	7.455172	2.660300
O	4.252266	10.967387	0.698443
H	4.278734	10.231469	1.332989
H	4.192520	11.746723	1.264647

Table5_1c_TSi_TSi-ii_h2o_2

Property	Value
Charge	0
Electronic Energy, BS1 (a.u.)	-1290.415402
Thermal and entropic correction, BS1 (a.u.)	2.856011
Electronic Energy, BS2 (a.u.)	-1290.864747
Number of Imaginary Frequencies	0
Imaginary frequencies (cm-1)	None

Molecular Geometry in Cartesian Coordinates

C	8.291177	1.029373	6.857415
H	8.642826	0.542260	7.775553
H	7.551118	1.774586	7.161628
H	7.764408	0.257473	6.282384
C	9.503894	1.087081	4.728869
H	8.761711	0.301665	4.538518
H	9.365400	1.859972	3.967641
H	10.485787	0.634324	4.542085
C	10.677510	1.601372	6.822431
H	11.427327	1.055041	6.236077
H	10.570115	1.006343	7.738449
C	11.339140	2.936035	7.243848
H	12.353835	2.923898	6.825683
H	11.496796	2.874214	8.328226
C	11.462683	5.034658	5.977926
H	10.987776	5.111516	4.995775
H	11.608932	6.054823	6.354386
H	12.465726	4.622711	5.810806
C	10.238802	4.956794	8.098492
H	9.149153	4.994738	8.180728
H	10.609488	4.504564	9.026849
H	10.604876	5.991086	8.092958
N	3.137979	14.988326	10.376131
N	0.270591	15.270088	10.839035
C	3.905453	13.819767	10.881662
H	3.342809	12.899686	10.702354
H	4.884773	13.710077	10.399424
H	4.097593	13.863709	11.961037
C	3.929883	15.753946	9.376789
H	3.380572	15.821686	8.434216
H	4.140533	16.784056	9.690918
H	4.902713	15.294960	9.160787
C	2.709734	15.870423	11.501136
H	3.147716	15.533494	12.449463
H	3.099664	16.888377	11.373360
C	1.188412	16.011536	11.752628
H	0.970169	17.087076	11.738917
H	1.019349	15.718126	12.796651
C	-0.626884	16.207475	10.112121
H	-1.689815	15.966020	10.238226
H	-0.509418	17.251146	10.429619
H	-0.410339	16.178670	9.040510
C	-0.517011	14.251997	11.583674
H	-0.245333	13.248556	11.243306
H	-0.341326	14.277272	12.666397
H	-1.599762	14.363325	11.444715
N	2.396714	1.270004	-6.132596
N	2.411719	3.637326	-7.838345
C	3.608360	0.718567	-5.470119
H	3.371253	0.018482	-4.659235
H	4.269384	0.178390	-6.159366
H	4.200711	1.531262	-5.040589
C	1.159365	0.686104	-5.550244
H	1.362003	-0.015521	-4.731430
H	0.523216	1.481613	-5.151843
H	0.555020	0.136911	-6.283010
C	2.448625	1.050034	-7.607644
H	1.600748	0.438567	-7.942052
H	3.330606	0.458091	-7.883981
C	2.465795	2.306804	-8.512560
H	3.360124	2.223230	-9.143259
H	1.631259	2.191464	-9.216041
C	1.179755	4.377351	-8.220953
H	1.388208	5.380428	-8.613756
H	0.588633	3.863422	-8.989236
H	0.528359	4.494509	-7.350547
C	3.628094	4.437232	-8.141727
H	3.397057	5.422582	-8.565576
H	4.205329	4.599986	-7.226937
H	4.300853	3.946207	-8.855939
C	-6.572428	15.724210	-0.618841

H	-7.103885	15.419968	0.291585
H	-5.511424	15.806287	-0.368427
H	-6.919811	16.738488	-0.852501
C	-7.656627	13.652784	-1.350984
H	-7.997731	13.724587	-0.310544
H	-8.562970	13.593461	-1.966400
H	-7.137702	12.696410	-1.461027
C	-7.267021	15.466358	-2.958960
H	-7.496608	16.516713	-2.738124
H	-8.228749	15.044125	-3.277425
C	-6.354863	15.473973	-4.210839
H	-6.959195	15.073630	-5.034915
H	-6.203758	16.527654	-4.478350
C	-4.984197	13.637328	-5.089093
H	-4.102018	13.687058	-5.739592
H	-4.948728	12.681569	-4.559477
H	-5.854637	13.594332	-5.755793
C	-3.911319	15.695896	-4.306617
H	-3.227251	15.379535	-5.104023
H	-4.230207	16.714065	-4.562434
H	-3.327901	15.771207	-3.384578
N	8.142770	13.661093	0.005175
C	7.245974	14.204882	0.854072
H	6.952703	15.227200	0.661636
C	6.702695	13.517174	1.957002
H	6.007448	14.041638	2.598932
C	7.103182	12.184648	2.225541
C	8.043374	11.625089	1.324797
H	8.419136	10.617415	1.447133
C	8.523253	12.389106	0.244021
H	9.242268	11.957715	-0.437567
C	6.613191	11.458772	3.351811
C	5.367100	11.382499	5.246083
C	4.402805	11.973128	6.120079
C	3.820775	13.238489	5.853326
H	4.082621	13.809593	4.972241
C	2.895449	13.794819	6.758828
H	2.460329	14.764017	6.560137
C	3.040083	11.967765	8.156392
H	2.715282	11.479662	9.064313
C	3.976325	11.335469	7.313060
H	4.364180	10.369293	7.608380
C	6.718708	9.638846	4.699936
C	7.236090	8.337934	4.981267
C	6.923539	7.644130	6.177822
H	6.295417	8.085075	6.940087
C	8.099280	7.664235	4.080234
H	8.401224	8.110252	3.141679
C	8.587731	6.381871	4.397299
H	9.244899	5.871367	3.707910
C	7.461460	6.362446	6.410040
H	7.228862	5.837654	7.325244
C	3.456615	3.155082	-2.912713
H	4.385216	3.163825	-3.465741
C	3.480093	3.168685	-1.504760
H	4.442497	3.178373	-1.009390
C	1.150442	3.112900	-2.953675
H	0.243612	3.084643	-3.540820
C	1.075870	3.127413	-1.545983
H	0.099000	3.103066	-1.080724
C	2.265101	3.153818	-0.773804
C	2.244229	3.163492	0.654198
C	1.089356	3.173182	2.608590
C	3.358800	3.201732	2.631510
C	4.591305	3.224591	3.351151
C	4.637671	3.186298	4.767220
H	3.734684	3.114058	5.359158
C	5.843798	3.285832	2.689826
H	5.916413	3.307377	1.610533
C	7.034941	3.317792	3.439659
H	7.987630	3.366583	2.932391
C	5.879594	3.223216	5.432060
H	5.912671	3.191212	6.511475
C	-0.156830	3.192417	3.305393
C	-1.394665	3.105513	2.620438
H	-1.442587	3.015554	1.543096
C	-2.602318	3.109215	3.343576
H	-3.544509	3.039322	2.818639
C	-1.489762	3.289148	5.357872
H	-1.548796	3.360472	6.434459
C	-0.232806	3.290369	4.718507
H	0.658856	3.360593	5.328312
C	-3.947629	13.743556	0.711091
H	-3.721128	14.783172	0.523055
C	-4.910179	11.782933	-0.025531
H	-5.453416	11.261921	-0.801582
C	-4.552928	11.104170	1.157216
H	-4.841623	10.066530	1.264741

C	-3.857060	11.790414	2.183691
C	-3.553259	13.151344	1.925711
H	-3.036576	13.767069	2.650562
C	-3.497171	11.158232	3.411965
C	-3.509229	9.346533	4.776485
C	-2.554136	11.285540	5.472968
N	-4.462613	5.227638	5.514010
C	-3.835567	7.977354	5.020669
C	-4.486342	7.169880	4.053629
H	-4.785354	7.567750	3.092298
C	-4.774441	5.819806	4.341777
H	-5.280764	5.208679	3.607457
C	-3.515711	7.339914	6.246284
H	-3.035538	7.873325	7.056697
C	-3.841524	5.984087	6.441545
H	-3.603447	5.506086	7.380183
C	-1.831011	12.027424	6.454292
C	-1.440804	11.455839	7.690206
H	-1.711448	10.442473	7.953753
C	-0.669217	12.205860	8.598446
H	-0.369451	11.762320	9.536646
C	-0.659638	14.036479	7.207862
H	-0.355644	15.059149	7.036619
C	-1.425455	13.366702	6.234634
H	-1.689816	13.897731	5.330062
C	3.397686	6.101603	-4.984029
H	4.340480	5.602246	-5.157556
C	1.095792	6.033323	-5.088886
H	0.203916	5.480205	-5.346948
C	0.983587	7.336836	-4.567570
H	-0.005793	7.758256	-4.446977
C	3.384276	7.409682	-4.459709
H	4.331204	7.888159	-4.243382
C	2.150332	8.070452	-4.234838
C	2.083844	9.393997	-3.702121
C	0.853518	11.169708	-3.004873
C	3.119388	11.258662	-2.926117
C	-0.418533	11.793253	-2.827220
C	-1.628888	11.124079	-3.139790
H	-1.637316	10.110794	-3.520016
C	-2.861116	11.786798	-2.983735
H	-3.781520	11.276306	-3.229486
C	-1.824834	13.706919	-2.231490
H	-1.921901	14.725120	-1.881798
C	-0.547432	13.123403	-2.353001
H	0.319336	13.720289	-2.099783
C	4.319509	12.003116	-2.721311
C	4.301513	13.356783	-2.301235
H	3.373699	13.876325	-2.102319
C	5.510284	14.068034	-2.172165
H	5.493496	15.105377	-1.870269
C	5.599299	11.442232	-2.961397
H	5.718721	10.415844	-3.283360
C	6.754580	12.230480	-2.799088
H	7.727844	11.801185	-2.989759
C	11.217771	14.508998	-0.823398
H	10.804062	13.510679	-0.991457
H	12.174477	14.548284	-1.359152
H	11.460350	14.589142	0.243671
C	9.879325	16.449705	-0.158045
H	8.798211	16.419600	0.005053
H	10.361539	16.178555	0.789462
H	10.141939	17.498008	-0.347362
C	10.793138	16.313712	-2.432629
H	10.863929	17.382164	-2.191804
H	11.828581	16.018658	-2.645768
C	10.027183	16.216766	-3.775682
H	9.804422	17.247516	-4.079873
H	10.752615	15.868948	-4.522226
C	7.597627	16.199384	-4.123610
H	6.916540	16.219880	-3.268290
H	7.841229	17.243541	-4.356632
H	7.034433	15.816155	-4.983798
C	8.937168	14.249695	-4.760559
H	8.128481	14.213392	-5.501170
H	9.874168	14.284074	-5.330222
H	8.931124	13.297644	-4.222134
N	-4.508022	1.007698	5.902508
N	-6.372022	3.095875	6.720505
C	-4.401131	0.427892	4.536557
H	-3.458965	-0.110089	4.373328
H	-5.203800	-0.283707	4.305704
H	-4.463332	1.219363	3.785677
C	-3.326228	0.642501	6.728061
H	-2.588772	0.044131	6.178438
H	-2.820979	1.547514	7.077820
H	-3.587304	0.062730	7.622241
C	-5.774699	0.574550	6.562557

H	-5.564883	0.004755	7.476941
H	-6.325668	-0.131815	5.928509
C	-6.782787	1.681277	6.957903
H	-7.722640	1.450927	6.439841
H	-7.015223	1.518222	8.018173
C	-7.356230	3.795223	5.851804
H	-7.739105	4.720728	6.299583
H	-6.892262	4.061203	4.897790
H	-8.235759	3.182832	5.616416
C	-6.190569	3.819538	8.006460
H	-6.785279	4.739626	8.067427
H	-6.463912	3.217800	8.882199
H	-5.140399	4.096120	8.132700
N	9.382224	1.682732	6.086401
N	10.628890	4.197411	6.881043
N	10.248698	15.542211	-1.276767
N	8.793904	15.376624	-3.800727
N	-6.754886	14.768545	-1.743409
N	-5.048845	14.757794	-4.113075
N	5.679386	12.065242	4.121081
N	5.856515	10.171623	5.597381
N	7.106868	10.216686	3.542925
N	8.283551	5.737683	5.542296
N	2.512359	13.181829	7.897253
N	2.316684	3.121743	-3.632030
N	3.437798	3.233506	1.282495
N	1.034348	3.159622	1.256296
N	2.216663	3.195374	3.356094
N	-2.656693	3.193702	4.687382
N	7.063266	3.284409	4.787275
N	-4.621510	13.081755	-0.251159
N	-3.815179	9.850922	3.558634
N	-2.858350	10.000068	5.763287
N	-2.829196	11.915159	4.308939
N	-0.271541	13.475453	8.372357
N	2.277559	5.418886	-5.300287
N	0.852059	9.908654	-3.490536
N	1.948135	11.886218	-2.665321
N	3.252498	10.000222	-3.403490
N	6.719525	13.524644	-2.421021
N	-2.964767	13.057726	-2.546736
Pd	1.412866	14.226034	9.368534
Pd	-4.842822	13.914073	-2.164491
Pd	8.834973	3.730079	5.828985
Pd	8.475685	14.527344	-1.872888
Pd	2.354196	3.364173	-5.724637
Pd	-4.498964	3.134545	5.699929
C	2.752180	5.824376	6.005089
C	2.997175	6.293660	4.674896
C	1.849010	6.455161	3.803319
C	0.637357	6.827231	4.423502
C	0.686230	7.017319	5.857236
C	1.584290	6.201568	6.612411
H	2.884271	6.183087	1.946429
H	3.540985	5.338675	6.569003
H	3.945339	6.071341	4.194627
C	1.921012	6.394615	2.404209
C	-0.482770	7.088074	3.619658
H	-0.193589	7.429328	6.343991
H	1.413324	6.029520	7.669174
C	-0.431148	6.981225	2.234484
C	0.801263	6.630531	1.614880
H	-1.406626	7.398432	4.096047
C	-1.677158	7.278902	1.424663
C	-2.512899	6.029513	1.113105
H	-1.409628	7.774499	0.488041
H	-2.293666	7.997626	1.976300
H	-3.385621	6.275630	0.504117
H	-1.925552	5.285343	0.567993
H	-2.860222	5.567308	2.042976
C	0.965464	6.611223	0.111749
C	1.312111	8.000615	-0.447512
H	0.066236	6.227811	-0.378342
H	1.777942	5.925618	-0.148025
H	1.491649	7.931446	-1.521126
H	0.499185	8.713394	-0.285309
H	2.212810	8.402331	0.026066
C	3.171433	9.040356	3.828544
C	3.217627	8.323758	5.142077
C	2.081859	8.749347	5.872160
C	1.363066	9.742042	5.043525
H	4.184873	8.157201	5.589324
H	2.038546	8.846947	6.946090
C	1.592243	10.615292	2.646149
C	0.115251	10.421803	2.274183
C	1.913310	12.094002	2.918166
H	2.205929	10.283859	1.805983
C	-0.252863	11.295201	1.066799

H	-0.504901	10.692763	3.132675
H	-0.061431	9.368633	2.062134
C	1.529192	12.961004	1.709092
H	1.358883	12.415595	3.807484
H	2.981346	12.196417	3.147393
C	0.050768	12.772417	1.344125
H	-1.310155	11.148347	0.814612
H	0.325496	10.968594	0.191499
H	1.738428	14.012693	1.918180
H	2.148431	12.668691	0.850312
H	-0.203507	13.393719	0.479355
H	-0.571675	13.122279	2.178532
N	1.999271	9.769657	3.781048
O	0.419537	10.445670	5.349501
O	4.019254	9.021113	2.931903
O	5.362619	6.538760	2.607704
H	5.643880	6.429185	1.691127
H	5.030114	7.453570	2.653794
O	4.238975	10.956957	0.686109
H	4.272149	10.222050	1.321678
H	4.184166	11.737180	1.251096

Table5_1d_TSi_reactant_1wat

Property	Value
Charge	0
Electronic Energy, BS1 (a.u.)	-1284.590231
Thermal and entropic correction, BS1 (a.u.)	2.656968
Electronic Energy, BS2 (a.u.)	-1285.048466
Number of Imaginary Frequencies	0
Imaginary frequencies (cm-1)	None

Molecular Geometry in Cartesian Coordinates

C	8.540676	0.971399	6.787691
H	8.899372	0.531540	7.726745
H	7.715807	1.639435	7.048136
H	8.119865	0.143861	6.202840
C	9.824887	1.132426	4.708063
H	9.171265	0.276471	4.497771
H	9.639480	1.883766	3.935050
H	10.852980	0.777465	4.563633
C	10.858922	1.773500	6.841200
H	11.669113	1.258853	6.308820
H	10.759574	1.214925	7.780780
C	11.405997	3.172077	7.217128
H	12.430301	3.216991	6.825339
H	11.537046	3.171854	8.306743
C	11.397068	5.189440	5.819561
H	10.921597	5.171292	4.834733
H	11.474236	6.239102	6.129529
H	12.425611	4.834421	5.678922
C	10.173965	5.166274	7.941425
H	9.084537	5.142727	8.020869
H	10.567275	4.796077	8.896528
H	10.476885	6.218486	7.872377
N	3.143665	15.006581	10.437797
N	0.278198	15.338479	10.879320
C	3.903659	13.818338	10.907762
H	3.338230	12.907012	10.695918
H	4.884107	13.719770	10.425377
H	4.092503	13.826402	11.988569
C	3.939776	15.795314	9.460277
H	3.388926	15.893340	8.521295
H	4.157237	16.814649	9.803303
H	4.909004	15.336129	9.229221
C	2.722353	15.858123	11.588755
H	3.132242	15.473210	12.531351
H	3.146216	16.867060	11.505712
C	1.201860	16.041381	11.817056
H	1.014890	17.122916	11.813780
H	1.007382	15.739837	12.854323
C	-0.581305	16.309439	10.150895
H	-1.652480	16.098168	10.260506
H	-0.437377	17.345920	10.480841
H	-0.350804	16.284251	9.081867
C	-0.548713	14.333228	11.598592
H	-0.294852	13.326524	11.254617
H	-0.393669	14.343947	12.684714
H	-1.625506	14.472406	11.439633
N	2.491949	1.352299	-6.411930
N	2.416377	3.930996	-7.777103
C	3.738900	0.752481	-5.866772
H	3.546373	-0.063304	-5.158679

H	4.393791	0.337895	-6.643316
H	4.320606	1.513967	-5.339512
C	1.290209	0.657361	-5.878613
H	1.538522	-0.147889	-5.175882
H	0.651971	1.369884	-5.348177
H	0.671688	0.201919	-6.662043
C	2.500077	1.337479	-7.904172
H	1.652749	0.759331	-8.294484
H	3.382804	0.807570	-8.284441
C	2.467800	2.706197	-8.628624
H	3.342421	2.726859	-9.291324
H	1.612784	2.671676	-9.315918
C	1.168332	4.699684	-8.028207
H	1.357732	5.748282	-8.289823
H	0.561652	4.284550	-8.842708
H	0.539116	4.692869	-7.134042
C	3.617184	4.780874	-7.994140
H	3.366427	5.811397	-8.275095
H	4.213239	4.826006	-7.078413
H	4.279049	4.399735	-8.781778
C	-6.601205	15.848203	-0.850435
H	-7.202048	15.596866	0.032624
H	-5.558930	15.921748	-0.528166
H	-6.908386	16.856943	-1.153753
C	-7.676063	13.764692	-1.562375
H	-8.079340	13.885916	-0.549213
H	-8.542803	13.697005	-2.231555
H	-7.171891	12.794617	-1.600356
C	-7.140783	15.495441	-3.219276
H	-7.354372	16.560707	-3.063834
H	-8.091696	15.082030	-3.579282
C	-6.150778	15.418347	-4.407949
H	-6.712458	14.995181	-5.250555
H	-5.951178	16.453579	-4.713225
C	-4.789978	13.494301	-5.095340
H	-3.871251	13.482044	-5.694919
H	-4.812341	12.571214	-4.509473
H	-5.622743	13.434140	-5.807268
C	-3.700889	15.565070	-4.368562
H	-2.974890	15.176875	-5.093874
H	-3.971220	16.571087	-4.713157
H	-3.179120	15.688846	-3.415414
N	8.118451	13.681579	0.084976
C	7.203444	14.212919	0.922715
H	6.904933	15.234267	0.733197
C	6.646031	13.513516	2.010984
H	5.931759	14.025715	2.641906
C	7.049371	12.181343	2.276419
C	8.007874	11.634074	1.387597
H	8.387275	10.627544	1.509578
C	8.502925	12.410254	0.322370
H	9.237739	11.988701	-0.348526
C	6.544302	11.444283	3.388939
C	5.279276	11.348353	5.269181
C	4.338596	11.963323	6.152024
C	3.837001	13.269789	5.918316
H	4.142300	13.852050	5.058383
C	2.934377	13.853557	6.827540
H	2.557678	14.850854	6.649777
C	2.953818	11.980661	8.172171
H	2.588475	11.489927	9.062885
C	3.859225	11.315860	7.318857
H	4.179642	10.318617	7.579123
C	6.591841	9.587022	4.690990
C	7.090022	8.270003	4.936223
C	6.747402	7.530324	6.097096
H	6.100850	7.943774	6.859319
C	7.970695	7.626616	4.029094
H	8.294924	8.105019	3.113573
C	8.452607	6.333770	4.307529
H	9.121802	5.847991	3.611556
C	7.276672	6.237320	6.291065
H	7.015455	5.672588	7.174798
C	3.521782	2.865464	-2.965983
H	4.450539	2.853376	-3.518310
C	3.550215	2.899829	-1.558473
H	4.513679	2.908811	-1.064899
C	1.220139	2.846244	-3.000779
H	0.309857	2.815010	-3.582796
C	1.147541	2.884361	-1.594417
H	0.171120	2.872570	-1.127720
C	2.337156	2.917146	-0.825381
C	2.316140	2.958225	0.601092
C	1.159682	3.032303	2.553501
C	3.427884	2.985681	2.578301
C	4.661401	3.005054	3.295344
C	4.708888	2.974745	4.710043
H	3.807528	2.906046	5.305107

C	5.914282	3.056606	2.634413
H	5.988838	3.067416	1.554348
C	7.105089	3.093056	3.384901
H	8.057116	3.133367	2.874791
C	5.950199	3.019269	5.374393
H	5.979157	3.001205	6.454304
C	-0.085055	3.137251	3.245186
C	-1.322453	3.169724	2.553680
H	-1.372737	3.109070	1.474161
C	-2.528272	3.267664	3.272793
H	-3.471081	3.286183	2.744509
C	-1.414037	3.321358	5.293470
H	-1.470959	3.389889	6.370507
C	-0.159037	3.222380	4.659015
H	0.732374	3.203883	5.272323
C	-4.061633	13.876002	0.722808
H	-3.843966	14.915618	0.524255
C	-5.017151	11.904834	0.015702
H	-5.565538	11.370855	-0.747468
C	-4.643223	11.239460	1.199173
H	-4.924670	10.200910	1.315309
C	-3.936358	11.936977	2.210042
C	-3.653348	13.299421	1.941009
H	-3.135798	13.923979	2.657560
C	-3.546369	11.308834	3.430877
C	-3.521052	9.485928	4.778940
C	-2.586185	11.429886	5.482844
N	-4.390823	5.343299	5.465566
C	-3.806689	8.105337	5.003501
C	-4.457386	7.303245	4.032552
H	-4.779240	7.712329	3.083244
C	-4.726410	5.947359	4.306414
H	-5.238436	5.341813	3.571515
C	-3.455964	7.454769	6.213621
H	-2.968287	7.985396	7.021430
C	-3.761155	6.091749	6.394354
H	-3.500266	5.602273	7.321260
C	-1.844307	12.156333	6.462068
C	-1.470373	11.574692	7.698601
H	-1.758641	10.565245	7.959766
C	-0.694863	12.308592	8.614903
H	-0.410348	11.856336	9.553688
C	-0.640962	14.141748	7.228452
H	-0.317086	15.159452	7.063088
C	-1.411144	13.487625	6.246671
H	-1.660019	14.023461	5.340323
C	3.427580	6.029444	-4.704792
H	4.371922	5.550623	-4.920846
C	1.122437	5.920980	-4.731870
H	0.232429	5.356756	-4.972163
C	1.006612	7.205779	-4.166577
H	0.014913	7.604854	-3.996968
C	3.409323	7.320288	-4.140747
H	4.354299	7.809058	-3.940204
C	2.172046	7.952548	-3.857825
C	2.102756	9.273090	-3.318943
C	0.868901	11.070899	-2.692094
C	3.132312	11.185769	-2.669732
C	-0.400923	11.712922	-2.570298
C	-1.612148	11.039431	-2.869320
H	-1.623381	10.004834	-3.187467
C	-2.839862	11.721920	-2.774791
H	-3.761005	11.207228	-3.008373
C	-1.797695	13.669768	-2.105153
H	-1.891009	14.704683	-1.807997
C	-0.524926	13.068564	-2.172205
H	0.342361	13.669402	-1.931308
C	4.325041	11.957024	-2.527817
C	4.295632	13.318406	-2.134722
H	3.365851	13.823919	-1.909163
C	5.493101	14.055987	-2.064707
H	5.463938	15.098170	-1.780697
C	5.606077	11.418889	-2.809036
H	5.733093	10.389873	-3.120552
C	6.749852	12.234447	-2.707385
H	7.723415	11.822614	-2.932715
C	11.190886	14.554606	-0.736821
H	10.787072	13.552655	-0.907510
H	12.148190	14.604196	-1.270697
H	11.430556	14.634752	0.330938
C	9.831459	16.479992	-0.069382
H	8.750274	16.439075	0.091144
H	10.314037	16.211022	0.878529
H	10.083968	17.531384	-0.255179
C	10.752600	16.359493	-2.342063
H	10.826276	17.425662	-2.092074
H	11.786366	16.064869	-2.563706
C	9.978049	16.277035	-3.681015

H	9.748386	17.310738	-3.969659
H	10.699932	15.942782	-4.437133
C	7.544863	16.253919	-4.006019
H	6.872403	16.256470	-3.143695
H	7.781208	17.302948	-4.224131
H	6.974550	15.881998	-4.866530
C	8.887603	14.322561	-4.689620
H	8.074235	14.297673	-5.425556
H	9.820666	14.369095	-5.264832
H	8.886912	13.360887	-4.168394
N	-4.439404	1.120426	5.778113
N	-6.297771	3.194030	6.644811
C	-4.323193	0.574916	4.398946
H	-3.379536	0.041898	4.228583
H	-5.123749	-0.131447	4.145587
H	-4.380520	1.385003	3.667585
C	-3.265905	0.731065	6.603881
H	-2.519935	0.154879	6.042238
H	-2.770014	1.625320	6.991452
H	-3.534778	0.119536	7.474284
C	-5.712256	0.674432	6.417209
H	-5.510336	0.066748	7.308610
H	-6.269159	-0.000502	5.754790
C	-6.708498	1.775218	6.856860
H	-7.657264	1.559749	6.348681
H	-6.924615	1.588198	7.916603
C	-7.283692	3.908412	5.790174
H	-7.663036	4.827886	6.253165
H	-6.823393	4.187083	4.838095
H	-8.165348	3.301539	5.548611
C	-6.113918	3.895118	7.942979
H	-6.714251	4.810004	8.023873
H	-6.378450	3.275862	8.809261
H	-5.064761	4.177222	8.068046
N	9.593159	1.723080	6.052999
N	10.616337	4.358492	6.774104
N	10.212779	15.579364	-1.189867
N	8.748255	15.431987	-3.708868
N	-6.727114	14.845318	-1.941460
N	-4.876513	14.672000	-4.191289
N	5.628833	12.056104	4.172270
N	5.721826	10.108919	5.590122
N	7.030112	10.198782	3.569884
N	8.121337	5.647799	5.419098
N	2.503164	13.231603	7.942563
N	2.381972	2.837760	-3.687560
N	3.509559	3.006870	1.230528
N	1.106953	2.999267	1.202253
N	2.286243	3.017001	3.303380
N	-2.581973	3.334755	4.617918
N	7.136141	3.076922	4.733386
N	-4.736639	13.201317	-0.231075
N	-3.848163	9.997893	3.571818
N	-2.908549	10.150640	5.779982
N	-2.866462	12.065158	4.321585
N	-0.274080	13.571301	8.395999
N	2.308157	5.339631	-5.005349
N	0.869137	9.786942	-3.113197
N	1.962272	11.811376	-2.404432
N	3.272604	9.916739	-3.112294
N	6.704279	13.534469	-2.349408
N	-2.937533	13.015500	-2.407970
Pd	1.413242	14.284100	9.411325
Pd	-4.811779	13.937612	-2.191025
Pd	8.860739	3.705760	5.752034
Pd	8.452663	14.546698	-1.793937
Pd	2.407751	3.367669	-5.720661
Pd	-4.425009	3.251716	5.625688
C	2.816628	5.663911	5.866441
C	3.012441	6.015129	4.546519
C	1.902589	6.301263	3.702276
C	0.643044	6.566276	4.316431
C	0.574159	6.543428	5.730423
C	1.544773	5.895554	6.465237
H	2.956576	6.098902	1.822944
H	4.021353	6.005616	4.136993
C	1.988556	6.268959	2.284204
C	-0.499877	6.750662	3.497121
H	-0.318510	6.882724	6.249660
C	-0.407594	6.614693	2.128531
C	0.849478	6.380419	1.518340
H	-1.459231	6.946305	3.968335
C	3.058568	9.003218	3.910353
C	3.123636	8.397221	5.266261
C	2.020348	8.662897	5.927674
C	1.184596	9.560818	5.100646
H	4.084600	7.931809	5.633497
H	1.758195	8.488417	6.985709

C	1.461274	10.602075	2.800178
C	0.042701	10.302302	2.293987
C	1.629955	12.073572	3.208984
H	2.154922	10.397499	1.979987
C	-0.301409	11.228479	1.119528
H	-0.661343	10.446093	3.115213
H	-0.021578	9.253031	1.993504
C	1.274292	12.991432	2.035332
H	0.978431	12.281404	4.070862
H	2.665403	12.238174	3.529733
C	-0.141827	12.702514	1.521546
H	-1.319710	11.027742	0.769015
H	0.382233	10.997772	0.292809
H	1.369108	14.040520	2.328296
H	1.993114	12.823924	1.221658
H	-0.376339	13.365018	0.677937
H	-0.858818	12.940908	2.318560
N	1.851891	9.701566	3.875026
O	0.174786	10.156771	5.420055
O	3.892784	8.972262	3.011464
O	2.539181	9.049563	0.169995
H	3.251550	8.980041	0.824154
H	1.848296	8.499602	0.560043
O	1.295523	5.617449	7.763748
O	3.888797	5.262677	6.618867
C	2.318092	4.851232	8.431971
H	2.131542	4.958054	9.501649
H	2.222688	3.800802	8.151964
C	3.681724	5.387545	8.050953
H	4.475561	4.811706	8.530232
H	3.773850	6.438777	8.335708
H	-1.295604	6.698567	1.513617
H	0.909316	6.281185	0.439730

Table5_1d_TSi_reactant_2wat

Property	Value
Charge	0
Electronic Energy, BS1 (a.u.)	-1361.039395
Thermal and entropic correction, BS1 (a.u.)	2.791547
Electronic Energy, BS2 (a.u.)	-1361.534255
Number of Imaginary Frequencies	0
Imaginary frequencies (cm-1)	None

Molecular Geometry in Cartesian Coordinates

C	8.153923	1.050560	6.706913
H	8.388114	0.509149	7.632280
H	7.403781	1.808747	6.953021
H	7.685709	0.325483	6.029176
C	9.583391	1.285557	4.702200
H	8.870235	0.523411	4.362759
H	9.486680	2.151988	4.041080
H	10.586496	0.867986	4.548277
C	10.556142	1.600888	6.957489
H	11.365943	1.097420	6.413133
H	10.364730	0.955565	7.824844
C	11.139435	2.937943	7.502910
H	12.194289	2.939531	7.200064
H	11.188464	2.811867	8.591867
C	11.427854	5.089715	6.336915
H	10.988565	5.268502	5.351143
H	11.639621	6.069039	6.783937
H	12.400538	4.611844	6.165747
C	10.112624	4.987208	8.397453
H	9.023774	5.072032	8.448516
H	10.430274	4.480532	9.317223
H	10.525745	6.002338	8.449131
N	2.522360	15.737596	11.030760
N	-0.398192	15.214409	10.604373
C	3.397119	14.799606	11.783838
H	3.248931	13.779451	11.419122
H	4.464422	15.032826	11.682262
H	3.188205	14.782294	12.860559
C	3.331772	16.791430	10.362368
H	3.158069	16.764515	9.282434
H	3.084861	17.806518	10.697123
H	4.410307	16.670565	10.522947
C	1.495282	16.356890	11.944476
H	1.705776	16.079971	12.985151
H	1.595015	17.449587	11.945363
C	-0.022335	16.073494	11.747462
H	-0.524952	17.046790	11.674232
H	-0.393524	15.640147	12.685599

C	-1.216534	15.910428	9.584389
H	-2.139444	15.365662	9.348129
H	-1.517856	16.917008	9.902399
H	-0.646675	16.020132	8.656824
C	-0.952048	13.901279	11.006517
H	-0.267237	13.098745	10.714345
H	-1.102054	13.824433	12.091015
H	-1.924002	13.699952	10.538977
N	2.303704	1.318224	-5.980200
N	2.353252	3.519244	-7.881658
C	3.417114	0.799635	-5.140607
H	3.066759	0.197740	-4.293009
H	4.117220	0.164395	-5.697594
H	4.002628	1.628801	-4.734865
C	0.992832	0.807019	-5.501515
H	1.079625	0.177425	-4.607095
H	0.337013	1.646922	-5.253371
H	0.464630	0.206442	-6.252722
C	2.521034	0.971998	-7.414620
H	1.745384	0.280303	-7.767804
H	3.458428	0.417634	-7.550637
C	2.550983	2.143785	-8.425291
H	3.501424	2.065662	-8.968502
H	1.790157	1.915264	-9.182571
C	1.066533	4.097657	-8.349810
H	1.188462	5.071850	-8.839838
H	0.539892	3.453819	-9.065369
H	0.392606	4.237710	-7.499174
C	3.497392	4.397103	-8.248199
H	3.182925	5.314623	-8.761016
H	4.048843	4.694603	-7.352298
H	4.221207	3.908303	-8.912162
C	-6.790254	15.877430	-0.414386
H	-7.310256	15.521629	0.484173
H	-5.717480	15.884955	-0.196763
H	-7.100760	16.919146	-0.566088
C	-7.879905	13.830611	-1.279628
H	-8.209841	13.817169	-0.232934
H	-8.785437	13.764154	-1.896283
H	-7.295962	12.921621	-1.456936
C	-7.553478	15.783695	-2.770136
H	-7.738183	16.830970	-2.496787
H	-8.533488	15.402864	-3.086828
C	-6.639263	15.795873	-4.030751
H	-7.299018	15.527457	-4.865810
H	-6.401129	16.850298	-4.219514
C	-5.548704	13.828257	-5.037165
H	-4.769100	13.806785	-5.808912
H	-5.505388	12.877501	-4.498104
H	-6.509081	13.844842	-5.567041
C	-4.214743	15.792972	-4.441918
H	-3.662282	15.404571	-5.306595
H	-4.480266	16.828429	-4.689210
H	-3.515625	15.839342	-3.601823
N	8.252382	13.905391	0.262718
C	7.617414	14.587824	1.240055
H	7.364956	15.617428	1.032016
C	7.291187	14.030497	2.493761
H	6.797084	14.658802	3.228836
C	7.616836	12.676120	2.765675
C	8.283907	11.974438	1.730813
H	8.582371	10.939830	1.844025
C	8.578825	12.623045	0.518209
H	9.097507	12.089273	-0.265351
C	7.280164	12.034936	3.995082
C	6.192780	12.045312	5.983708
C	5.295110	12.694038	6.883994
C	4.831995	14.014264	6.657782
H	5.175424	14.601462	5.815892
C	3.890438	14.589093	7.532620
H	3.532403	15.591816	7.348223
C	3.841077	12.704771	8.851323
H	3.447822	12.202099	9.723609
C	4.781191	12.046313	8.034142
H	5.090770	11.045785	8.306878
C	7.228778	10.156415	5.267967
C	7.584596	8.785586	5.452750
C	7.205156	8.057088	6.608400
H	6.637697	8.519023	7.406255
C	8.338421	8.071999	4.485448
H	8.681631	8.541952	3.572321
C	8.670639	6.721516	4.706477
H	9.253499	6.181271	3.973362
C	7.570008	6.702551	6.736928
H	7.269648	6.145485	7.613054
C	3.396641	3.474674	-2.924415
H	4.295871	3.687720	-3.484341
C	3.455881	3.315742	-1.524703

H	4.416935	3.407743	-1.036076
C	1.119235	3.110840	-2.952945
H	0.205727	3.064645	-3.527137
C	1.080654	2.943406	-1.552655
H	0.125331	2.744145	-1.085341
C	2.270895	3.057618	-0.789048
C	2.267361	2.956725	0.636670
C	1.100146	2.933852	2.582035
C	3.362365	3.106034	2.622334
C	4.564379	3.279489	3.373229
C	4.551602	3.393865	4.787688
H	3.633150	3.329845	5.356125
C	5.834747	3.377779	2.751874
H	5.944806	3.288969	1.679328
C	6.986174	3.592518	3.536501
H	7.954734	3.671747	3.064024
C	5.752993	3.610324	5.489081
H	5.744852	3.702576	6.565856
C	-0.144977	2.969612	3.277498
C	-1.385855	2.866346	2.601699
H	-1.432211	2.722587	1.530617
C	-2.590312	2.910078	3.332918
H	-3.535825	2.824306	2.819381
C	-1.463037	3.142285	5.331626
H	-1.511624	3.240173	6.406648
C	-0.212744	3.117166	4.686243
H	0.678820	3.199155	5.294327
C	-3.768263	13.926580	0.612787
H	-3.535701	14.957392	0.384968
C	-4.869572	11.992178	0.011073
H	-5.510354	11.484151	-0.695587
C	-4.406805	11.314085	1.156075
H	-4.711224	10.286220	1.303193
C	-3.561223	11.978465	2.080495
C	-3.256909	13.331008	1.782878
H	-2.633201	13.928841	2.434853
C	-3.038658	11.317826	3.233953
C	-2.856807	9.448569	4.510362
C	-1.755046	11.341253	5.108221
N	-4.023435	5.412288	5.347485
C	-3.236986	8.100239	4.785295
C	-4.129058	7.386788	3.944702
H	-4.559912	7.840000	3.061626
C	-4.481180	6.058974	4.256209
H	-5.155497	5.517214	3.609900
C	-2.758896	7.400751	5.922159
H	-2.090578	7.865875	6.634587
C	-3.176637	6.076770	6.159846
H	-2.825181	5.548387	7.034587
C	-0.932034	12.050117	6.036565
C	-0.413747	11.432287	7.203366
H	-0.615597	10.394113	7.432984
C	0.364472	12.178854	8.110551
H	0.748115	11.712376	9.007028
C	0.188977	14.082425	6.819475
H	0.436734	15.126833	6.687603
C	-0.603134	13.418806	5.860627
H	-0.957152	13.982415	5.006729
C	3.281026	6.245112	-5.170448
H	4.227849	5.730442	-5.246998
C	0.998800	6.192689	-5.462651
H	0.122228	5.633927	-5.754707
C	0.861658	7.528228	-5.025118
H	-0.128268	7.963257	-4.998990
C	3.243032	7.577559	-4.713990
H	4.177076	8.051818	-4.443413
C	2.002489	8.256833	-4.602973
C	1.909505	9.579641	-4.069078
C	0.669804	11.357459	-3.386681
C	2.928849	11.394888	-3.157019
C	-0.594329	11.992894	-3.184570
C	-1.821270	11.363459	-3.515375
H	-1.847069	10.382027	-3.969675
C	-3.042847	12.023968	-3.264012
H	-3.977592	11.540233	-3.508333
C	-1.960624	13.876816	-2.417172
H	-2.034251	14.867485	-1.990767
C	-0.696874	13.294187	-2.627743
H	0.178823	13.869357	-2.357273
C	4.113025	12.069710	-2.728690
C	4.072452	13.370898	-2.164819
H	3.139269	13.902909	-2.031306
C	5.267581	14.012642	-1.783997
H	5.238187	15.008123	-1.363911
C	5.400037	11.488688	-2.855765
H	5.537009	10.503791	-3.283094
C	6.540545	12.207903	-2.444879
H	7.523687	11.772413	-2.555101

C	11.075368	14.811563	-0.979591
H	10.677916	13.793263	-0.940628
H	11.983153	14.773147	-1.594700
H	11.404155	15.074031	0.033558
C	9.769159	16.835182	-0.543171
H	8.709449	16.826627	-0.270061
H	10.349291	16.743904	0.383570
H	9.980690	17.832942	-0.947904
C	10.490409	16.306674	-2.836643
H	10.776223	17.358155	-2.704371
H	11.419791	15.827179	-3.169270
C	9.534872	16.303545	-4.065213
H	9.326629	17.352664	-4.313005
H	10.110295	15.925714	-4.920663
C	7.068704	16.412322	-3.886866
H	6.603638	16.363444	-2.897247
H	7.303801	17.465832	-4.085720
H	6.316985	16.110653	-4.627325
C	8.150638	14.416408	-4.872265
H	7.211103	14.445736	-5.438769
H	8.962958	14.400425	-5.610277
H	8.185886	13.465684	-4.331574
N	-4.863556	1.313215	5.986942
N	-6.287541	3.758578	6.691032
C	-5.028732	0.684897	4.649504
H	-4.252871	-0.058245	4.426622
H	-5.990155	0.169809	4.531946
H	-4.985957	1.450331	3.869441
C	-3.693599	0.737848	6.703152
H	-3.147847	-0.002196	6.104596
H	-2.988477	1.530838	6.967216
H	-3.965419	0.234088	7.639234
C	-6.106278	1.170742	6.801263
H	-5.902101	0.642511	7.741317
H	-6.836272	0.530974	6.288770
C	-6.859225	2.469885	7.180746
H	-7.889029	2.340608	6.823777
H	-6.953617	2.470933	8.274381
C	-7.204616	4.413895	5.721538
H	-7.444429	5.449464	5.993215
H	-6.745722	4.429677	4.729187
H	-8.164367	3.893108	5.613970
C	-5.977814	4.666523	7.827981
H	-6.453831	5.650160	7.729623
H	-6.298192	4.267439	8.798607
H	-4.898388	4.829149	7.896936
N	9.348605	1.689894	6.107734
N	10.505338	4.262356	7.159210
N	10.045251	15.743156	-1.512415
N	8.270969	15.549195	-3.925197
N	-7.065967	15.027058	-1.595914
N	-5.389904	14.955121	-4.081203
N	6.570944	12.750545	4.894318
N	6.488533	10.747836	6.232809
N	7.615065	10.731989	4.109856
N	8.294686	6.042488	5.808210
N	3.388956	13.954274	8.611805
N	2.252934	3.379692	-3.633334
N	3.457088	3.095132	1.271243
N	1.057272	2.847110	1.233591
N	2.214834	3.058871	3.331098
N	-2.635695	3.037387	4.675260
N	6.949816	3.709967	4.879844
N	-4.557916	13.274092	-0.264108
N	-3.374846	10.018401	3.399649
N	-1.996585	10.036891	5.372497
N	-2.216128	12.028772	4.039199
N	0.663725	13.480395	7.928894
N	2.184133	5.549500	-5.534479
N	0.669949	10.109952	-3.917306
N	1.760032	12.043541	-2.978102
N	3.070758	10.154571	-3.674242
N	6.484350	13.449665	-1.921577
N	-3.117720	13.259720	-2.726121
Pd	1.583306	14.595801	9.453594
Pd	-4.962518	14.092239	-2.150536
Pd	8.705315	3.987835	5.999635
Pd	8.225958	14.601721	-1.716113
Pd	2.273813	3.444817	-5.751131
Pd	-4.453313	3.381541	5.668864
C	4.863960	6.666601	3.719554
C	4.172389	6.426518	2.554678
C	2.754219	6.396730	2.551681
C	2.046667	6.625807	3.778029
C	2.792946	6.833923	4.968252
C	4.170147	6.850520	4.938714
H	2.525396	5.942708	0.437564
H	5.946441	6.708046	3.700320

H	4.705457	6.257567	1.625333
C	2.014904	6.147465	1.371447
C	0.626996	6.654348	3.755983
H	2.262375	6.969986	5.904204
H	4.737201	6.966241	5.854538
C	-0.070090	6.434375	2.589158
C	0.639821	6.140129	1.386341
H	0.069765	6.860205	4.664511
C	2.712816	8.935238	-0.600125
C	3.415425	7.784143	-1.250084
C	4.647452	7.714265	-0.735919
C	4.794160	8.776208	0.316314
H	2.890703	7.138173	-1.948318
H	5.449486	7.022921	-0.954465
C	3.159788	10.512915	1.288851
C	2.681300	9.800768	2.564390
C	4.255611	11.543508	1.566715
H	2.305461	11.017887	0.828586
C	2.270557	10.808091	3.645638
H	3.494792	9.173717	2.945860
H	1.851399	9.132646	2.314255
C	3.855325	12.517013	2.685816
H	5.164713	11.016032	1.865337
H	4.487210	12.087407	0.642855
C	3.436735	11.760157	3.951369
H	1.967713	10.264886	4.547534
H	1.400203	11.387102	3.309490
H	4.727488	13.152003	2.846808
H	3.036852	13.167048	2.360208
H	3.169326	12.459342	4.751517
H	4.269650	11.137294	4.305619
N	3.597871	9.523202	0.287570
O	5.732038	8.945809	1.066374
O	1.574502	9.340833	-0.803520
O	1.073765	6.043669	-2.307907
H	0.902199	5.670099	-1.431059
H	0.420856	6.770733	-2.305288
O	-0.721717	8.034316	-1.614181
H	-1.154472	8.575964	-2.284246
H	0.055335	8.549252	-1.317584
O	-0.010685	5.834869	0.228127
O	-1.439705	6.475120	2.587823
C	-1.443608	5.718552	0.289533
H	-1.805300	5.923102	-0.716977
H	-1.709910	4.696159	0.584189
C	-1.988268	6.725179	1.277663
H	-3.070669	6.634588	1.368765
H	-1.729729	7.736566	0.944395

Table5_1d_TSi_reactant_1wat

Property	Value
Charge	0
Electronic Energy, BS1 (a.u.)	-1284.590231
Thermal and entropic correction, BS1 (a.u.)	2.656968
Electronic Energy, BS2 (a.u.)	-1282.328912
Number of Imaginary Frequencies	0
Imaginary frequencies (cm-1)	None

Molecular Geometry in Cartesian Coordinates

C	8.540676	0.971399	6.787691
H	8.899372	0.531540	7.726745
H	7.715807	1.639435	7.048136
H	8.119865	0.143861	6.202840
C	9.824887	1.132426	4.708063
H	9.171265	0.276471	4.497771
H	9.639480	1.883766	3.935050
H	10.852980	0.777465	4.563633
C	10.858922	1.773500	6.841200
H	11.669113	1.258853	6.308820
H	10.759574	1.214925	7.780780
C	11.405997	3.172077	7.217128
H	12.430301	3.216991	6.825339
H	11.537046	3.171854	8.306743
C	11.397068	5.189440	5.819561
H	10.921597	5.171292	4.834733
H	11.474236	6.239102	6.129529
H	12.425611	4.834421	5.678922
C	10.173965	5.166274	7.941425
H	9.084537	5.142727	8.020869
H	10.567275	4.796077	8.896528
H	10.476885	6.218486	7.872377
N	3.143665	15.006581	10.437797

N	0.278198	15.338479	10.879320
C	3.903659	13.818338	10.907762
H	3.338230	12.907012	10.695918
H	4.884107	13.719770	10.425377
H	4.092503	13.826402	11.988569
C	3.939776	15.795314	9.460277
H	3.388926	15.893340	8.521295
H	4.157237	16.814649	9.803303
H	4.909004	15.336129	9.229221
C	2.722353	15.858123	11.588755
H	3.132242	15.473210	12.531351
H	3.146216	16.867060	11.505712
C	1.201860	16.041381	11.817056
H	1.014890	17.122916	11.813780
H	1.007382	15.739837	12.854323
C	-0.581305	16.309439	10.150895
H	-1.652480	16.098168	10.260506
H	-0.437377	17.345920	10.480841
H	-0.350804	16.284251	9.081867
C	-0.548713	14.333228	11.598592
H	-0.294852	13.326524	11.254617
H	-0.393669	14.343947	12.684714
H	-1.625506	14.472406	11.439633
N	2.491949	1.352299	-6.411930
N	2.416377	3.930996	-7.777103
C	3.738900	0.752481	-5.866772
H	3.546373	-0.063304	-5.158679
H	4.393791	0.337895	-6.643316
H	4.320606	1.513967	-5.339512
C	1.290209	0.657361	-5.878613
H	1.538522	-0.147889	-5.175882
H	0.651971	1.369884	-5.348177
H	0.671688	0.201919	-6.662043
C	2.500077	1.337479	-7.904172
H	1.652749	0.759331	-8.294484
H	3.382804	0.807570	-8.284441
C	2.467800	2.706197	-8.628624
H	3.342421	2.726859	-9.291324
H	1.612784	2.671676	-9.315918
C	1.168332	4.699684	-8.028207
H	1.357732	5.748282	-8.289823
H	0.561652	4.284550	-8.842708
H	0.539116	4.692869	-7.134042
C	3.617184	4.780874	-7.994140
H	3.366427	5.811397	-8.275095
H	4.213239	4.826006	-7.078413
H	4.279049	4.399735	-8.781778
C	-6.601205	15.848203	-0.850435
H	-7.202048	15.596866	0.032624
H	-5.558930	15.921748	-0.528166
H	-6.908386	16.856943	-1.153753
C	-7.676063	13.764692	-1.562375
H	-8.079340	13.885916	-0.549213
H	-8.542803	13.697005	-2.231555
H	-7.171891	12.794617	-1.600356
C	-7.140783	15.495441	-3.219276
H	-7.354372	16.560707	-3.063834
H	-8.091696	15.082030	-3.579282
C	-6.150778	15.418347	-4.407949
H	-6.712458	14.995181	-5.250555
H	-5.951178	16.453579	-4.713225
C	-4.789978	13.494301	-5.095340
H	-3.871251	13.482044	-5.694919
H	-4.812341	12.571214	-4.509473
H	-5.622743	13.434140	-5.807268
C	-3.700889	15.565070	-4.368562
H	-2.974890	15.176875	-5.093874
H	-3.971220	16.571087	-4.713157
H	-3.179120	15.688846	-3.415414
N	8.118451	13.681579	0.084976
C	7.203444	14.212919	0.922715
H	6.904933	15.234267	0.733197
C	6.646031	13.513516	2.010984
H	5.931759	14.025715	2.641906
C	7.049371	12.181343	2.276419
C	8.007874	11.634074	1.387597
H	8.387275	10.627544	1.509578
C	8.502925	12.410254	0.322370
H	9.237739	11.988701	-0.348526
C	6.544302	11.444283	3.388939
C	5.279276	11.348353	5.269181
C	4.338596	11.963323	6.152024
C	3.837001	13.269789	5.918316
H	4.142300	13.852050	5.058383
C	2.934377	13.853557	6.827540
H	2.557678	14.850854	6.649777
C	2.953818	11.980661	8.172171
H	2.588475	11.489927	9.062885

C	3.859225	11.315860	7.318857
H	4.179642	10.318617	7.579123
C	6.591841	9.587022	4.690990
C	7.090022	8.270003	4.936223
C	6.747402	7.530324	6.097096
H	6.100850	7.943774	6.859319
C	7.970695	7.626616	4.029094
H	8.294924	8.105019	3.113573
C	8.452607	6.333770	4.307529
H	9.121802	5.847991	3.611556
C	7.276672	6.237320	6.291065
H	7.015455	5.672588	7.174798
C	3.521782	2.865464	-2.965983
H	4.450539	2.853376	-3.518310
C	3.550215	2.899829	-1.558473
H	4.513679	2.908811	-1.064899
C	1.220139	2.846244	-3.000779
H	0.309857	2.815010	-3.582796
C	1.147541	2.884361	-1.594417
H	0.171120	2.872570	-1.127720
C	2.337156	2.917146	-0.825381
C	2.316140	2.958225	0.601092
C	1.159682	3.032303	2.553501
C	3.427884	2.985681	2.578301
C	4.661401	3.005054	3.295344
C	4.708888	2.974745	4.710043
H	3.807528	2.906046	5.305107
C	5.914282	3.056606	2.634413
H	5.988838	3.067416	1.554348
C	7.105089	3.093056	3.384901
H	8.057116	3.133367	2.874791
C	5.950199	3.019269	5.374393
H	5.979157	3.001205	6.454304
C	-0.085055	3.137251	3.245186
C	-1.322453	3.169724	2.553680
H	-1.372737	3.109070	1.474161
C	-2.528272	3.267664	3.272793
H	-3.471081	3.286183	2.744509
C	-1.414037	3.321358	5.293470
H	-1.470959	3.389889	6.370507
C	-0.159037	3.222380	4.659015
H	0.732374	3.203883	5.272323
C	-4.061633	13.876002	0.722808
H	-3.843966	14.915618	0.524255
C	-5.017151	11.904834	0.015702
H	-5.565538	11.370855	-0.747468
C	-4.643223	11.239460	1.199173
H	-4.924670	10.200910	1.315309
C	-3.936358	11.936977	2.210042
C	-3.653348	13.299421	1.941009
H	-3.135798	13.923979	2.657560
C	-3.546369	11.308834	3.430877
C	-3.521052	9.485928	4.778940
C	-2.586185	11.429886	5.482844
N	-4.390823	5.343299	5.465566
C	-3.806689	8.105337	5.003501
C	-4.457386	7.303245	4.032552
H	-4.779240	7.712329	3.083244
C	-4.726410	5.947359	4.306414
H	-5.238436	5.341813	3.571515
C	-3.455964	7.454769	6.213621
H	-2.968287	7.985396	7.021430
C	-3.761155	6.091749	6.394354
H	-3.500266	5.602273	7.321260
C	-1.844307	12.156333	6.462068
C	-1.470373	11.574692	7.698601
H	-1.758641	10.565245	7.959766
C	-0.694863	12.308592	8.614903
H	-0.410348	11.856336	9.553688
C	-0.640962	14.141748	7.228452
H	-0.317086	15.159452	7.063088
C	-1.411144	13.487625	6.246671
H	-1.660019	14.023461	5.340323
C	3.427580	6.029444	-4.704792
H	4.371922	5.550623	-4.920846
C	1.122437	5.920980	-4.731870
H	0.232429	5.356756	-4.972163
C	1.006612	7.205779	-4.166577
H	0.014913	7.604854	-3.996968
C	3.409323	7.320288	-4.140747
H	4.354299	7.809058	-3.940204
C	2.172046	7.952548	-3.857825
C	2.102756	9.273090	-3.318943
C	0.868901	11.070899	-2.692094
C	3.132312	11.185769	-2.669732
C	-0.400923	11.712922	-2.570298
C	-1.612148	11.039431	-2.869320
H	-1.623381	10.004834	-3.187467

C	-2.839862	11.721920	-2.774791
H	-3.761005	11.207228	-3.008373
C	-1.797695	13.669768	-2.105153
H	-1.891009	14.704683	-1.807997
C	-0.524926	13.068564	-2.172205
H	0.342361	13.669402	-1.931308
C	4.325041	11.957024	-2.527817
C	4.295632	13.318406	-2.134722
H	3.365851	13.823919	-1.909163
C	5.493101	14.055987	-2.064707
H	5.463938	15.098170	-1.780697
C	5.606077	11.418889	-2.809036
H	5.733093	10.389873	-3.120552
C	6.749852	12.234447	-2.707385
H	7.723415	11.822614	-2.932715
C	11.190886	14.554606	-0.736821
H	10.787072	13.552655	-0.907510
H	12.148190	14.604196	-1.270697
H	11.430556	14.634752	0.330938
C	9.831459	16.479992	-0.069382
H	8.750274	16.439075	0.091144
H	10.314037	16.211022	0.878529
H	10.083968	17.531384	-0.255179
C	10.752600	16.359493	-2.342063
H	10.826276	17.425662	-2.092074
H	11.786366	16.064869	-2.563706
C	9.978049	16.277035	-3.681015
H	9.748386	17.310738	-3.969659
H	10.699932	15.942782	-4.437133
C	7.544863	16.253919	-4.006019
H	6.872403	16.256470	-3.143695
H	7.781208	17.302948	-4.224131
H	6.974550	15.881998	-4.866530
C	8.887603	14.322561	-4.689620
H	8.074235	14.297673	-5.425556
H	9.820666	14.369095	-5.264832
H	8.886912	13.360887	-4.168394
N	-4.439404	1.120426	5.778113
N	-6.297771	3.194030	6.644811
C	-4.323193	0.574916	4.398946
H	-3.379536	0.041898	4.228583
H	-5.123749	-0.131447	4.145587
H	-4.380520	1.385003	3.667585
C	-3.265905	0.731065	6.603881
H	-2.519935	0.154879	6.042238
H	-2.770014	1.625320	6.991452
H	-3.534778	0.119536	7.474284
C	-5.712256	0.674432	6.417209
H	-5.510336	0.066748	7.308610
H	-6.269159	-0.000502	5.754790
C	-6.708498	1.775218	6.856860
H	-7.657264	1.559749	6.348681
H	-6.924615	1.588198	7.916603
C	-7.283692	3.908412	5.790174
H	-7.663036	4.827886	6.253165
H	-6.823393	4.187083	4.838095
H	-8.165348	3.301539	5.548611
C	-6.113918	3.895118	7.942979
H	-6.714251	4.810004	8.023873
H	-6.378450	3.275862	8.809261
H	-5.064761	4.177222	8.068046
N	9.593159	1.723080	6.052999
N	10.616337	4.358492	6.774104
N	10.212779	15.579364	-1.189867
N	8.748255	15.431987	-3.708868
N	-6.727114	14.845318	-1.941460
N	-4.876513	14.672000	-4.191289
N	5.628833	12.056104	4.172270
N	5.721826	10.108919	5.590122
N	7.030112	10.198782	3.569884
N	8.121337	5.647799	5.419098
N	2.503164	13.231603	7.942563
N	2.381972	2.837760	-3.687560
N	3.509559	3.006870	1.230528
N	1.106953	2.999267	1.202253
N	2.286243	3.017001	3.303380
N	-2.581973	3.334755	4.617918
N	7.136141	3.076922	4.733386
N	-4.736639	13.201317	-0.231075
N	-3.848163	9.997893	3.571818
N	-2.908549	10.150640	5.779982
N	-2.866462	12.065158	4.321585
N	-0.274080	13.571301	8.395999
N	2.308157	5.339631	-5.005349
N	0.869137	9.786942	-3.113197
N	1.962272	11.811376	-2.404432
N	3.272604	9.916739	-3.112294
N	6.704279	13.534469	-2.349408

N	-2.937533	13.015500	-2.407970
Pd	1.413242	14.284100	9.411325
Pd	-4.811779	13.937612	-2.191025
Pd	8.860739	3.705760	5.752034
Pd	8.452663	14.546698	-1.793937
Pd	2.407751	3.367669	-5.720661
Pd	-4.425009	3.251716	5.625688
C	2.816628	5.663911	5.866441
C	3.012441	6.015129	4.546519
C	1.902589	6.301263	3.702276
C	0.643044	6.566276	4.316431
C	0.574159	6.543428	5.730423
C	1.544773	5.895554	6.465237
H	2.956576	6.098902	1.822944
H	4.021353	6.005616	4.136993
C	1.988556	6.268959	2.284204
C	-0.499877	6.750662	3.497121
H	-0.318510	6.882724	6.249660
C	-0.407594	6.614693	2.128531
C	0.849478	6.380419	1.518340
H	-1.459231	6.946305	3.968335
C	3.058568	9.003218	3.910353
C	3.123636	8.397221	5.266261
C	2.020348	8.662897	5.927674
C	1.184596	9.560818	5.100646
H	4.084600	7.931809	5.633497
H	1.758195	8.488417	6.985709
C	1.461274	10.602075	2.800178
C	0.042701	10.302302	2.293987
C	1.629955	12.073572	3.208984
H	2.154922	10.397499	1.979987
C	-0.301409	11.228479	1.119528
H	-0.661343	10.446093	3.115213
H	-0.021578	9.253031	1.993504
C	1.274292	12.991432	2.035332
H	0.978431	12.281404	4.070862
H	2.665403	12.238174	3.529733
C	-0.141827	12.702514	1.521546
H	-1.319710	11.027742	0.769015
H	0.382233	10.997772	0.292809
H	1.369108	14.040520	2.328296
H	1.993114	12.823924	1.221658
H	-0.376339	13.365018	0.677937
H	-0.858818	12.940908	2.318560
N	1.851891	9.701566	3.875026
O	0.174786	10.156771	5.420055
O	3.892784	8.972262	3.011464
O	2.539181	9.049563	0.169995
H	3.251550	8.980041	0.824154
H	1.848296	8.499602	0.560043
O	1.295523	5.617449	7.763748
O	3.888797	5.262677	6.618867
C	2.318092	4.851232	8.431971
H	2.131542	4.958054	9.501649
H	2.222688	3.800802	8.151964
C	3.681724	5.387545	8.050953
H	4.475561	4.811706	8.530232
H	3.773850	6.438777	8.335708
H	-1.295604	6.698567	1.513617
H	0.909316	6.281185	0.439730

Table5_1d_TSi_reactant_2wat

Property	Value
Charge	0
Electronic Energy, BS1 (a.u.)	-1361.039395
Thermal and entropic correction, BS1 (a.u.)	2.791547
Electronic Energy, BS2 (a.u.)	-1361.534255
Number of Imaginary Frequencies	0
Imaginary frequencies (cm-1)	None

Molecular Geometry in Cartesian Coordinates

C	8.153923	1.050560	6.706913
H	8.388114	0.509149	7.632280
H	7.403781	1.808747	6.953021
H	7.685709	0.325483	6.029176
C	9.583391	1.285557	4.702200
H	8.870235	0.523411	4.362759
H	9.486680	2.151988	4.041080
H	10.586496	0.867986	4.548277
C	10.556142	1.600888	6.957489
H	11.365943	1.097420	6.413133
H	10.364730	0.955565	7.824844

C	11.139435	2.937943	7.502910
H	12.194289	2.939531	7.200064
H	11.188464	2.811867	8.591867
C	11.427854	5.089715	6.336915
H	10.988565	5.268502	5.351143
H	11.639621	6.069039	6.783937
H	12.400538	4.611844	6.165747
C	10.112624	4.987208	8.397453
H	9.023774	5.072032	8.448516
H	10.430274	4.480532	9.317223
H	10.525745	6.002338	8.449131
N	2.522360	15.737596	11.030760
N	-0.398192	15.214409	10.604373
C	3.397119	14.799606	11.783838
H	3.248931	13.779451	11.419122
H	4.464422	15.032826	11.682262
H	3.188205	14.782294	12.860559
C	3.331772	16.791430	10.362368
H	3.158069	16.764515	9.282434
H	3.084861	17.806518	10.697123
H	4.410307	16.670565	10.522947
C	1.495282	16.356890	11.944476
H	1.705776	16.079971	12.985151
H	1.595015	17.449587	11.945363
C	-0.022335	16.073494	11.747462
H	-0.524952	17.046790	11.674232
H	-0.393524	15.640147	12.685599
C	-1.216534	15.910428	9.584389
H	-2.139444	15.365662	9.348129
H	-1.517856	16.917008	9.902399
H	-0.646675	16.020132	8.656824
C	-0.952048	13.901279	11.006517
H	-0.267237	13.098745	10.714345
H	-1.102054	13.824433	12.091015
H	-1.924002	13.699952	10.538977
N	2.303704	1.318224	-5.980200
N	2.353252	3.519244	-7.881658
C	3.417114	0.799635	-5.140607
H	3.066759	0.197740	-4.293009
H	4.117220	0.164395	-5.697594
H	4.002628	1.628801	-4.734865
C	0.992832	0.807019	-5.501515
H	1.079625	0.177425	-4.607095
H	0.337013	1.646922	-5.253371
H	0.464630	0.206442	-6.252722
C	2.521034	0.971998	-7.414620
H	1.745384	0.280303	-7.767804
H	3.458428	0.417634	-7.550637
C	2.550983	2.143785	-8.425291
H	3.501424	2.065662	-8.968502
H	1.790157	1.915264	-9.182571
C	1.066533	4.097657	-8.349810
H	1.188462	5.071850	-8.839838
H	0.539892	3.453819	-9.065369
H	0.392606	4.237710	-7.499174
C	3.497392	4.397103	-8.248199
H	3.182925	5.314623	-8.761016
H	4.048843	4.694603	-7.352298
H	4.221207	3.908303	-8.912162
C	-6.790254	15.877430	-0.414386
H	-7.310256	15.521629	0.484173
H	-5.717480	15.884955	-0.196763
H	-7.100760	16.919146	-0.566088
C	-7.879905	13.830611	-1.279628
H	-8.209841	13.817169	-0.232934
H	-8.785437	13.764154	-1.896283
H	-7.295962	12.921621	-1.456936
C	-7.553478	15.783695	-2.770136
H	-7.738183	16.830970	-2.496787
H	-8.533488	15.402864	-3.086828
C	-6.639263	15.795873	-4.030751
H	-7.299018	15.527457	-4.865810
H	-6.401129	16.850298	-4.219514
C	-5.548704	13.828257	-5.037165
H	-4.769100	13.806785	-5.808912
H	-5.505388	12.877501	-4.498104
H	-6.509081	13.844842	-5.567041
C	-4.214743	15.792972	-4.441918
H	-3.662282	15.404571	-5.306595
H	-4.480266	16.828429	-4.689210
H	-3.515625	15.839342	-3.601823
N	8.252382	13.905391	0.262718
C	7.617414	14.587824	1.240055
H	7.364956	15.617428	1.032016
C	7.291187	14.030497	2.493761
H	6.797084	14.658802	3.228836
C	7.616836	12.676120	2.765675
C	8.283907	11.974438	1.730813

H	8.582371	10.939830	1.844025
C	8.578825	12.623045	0.518209
H	9.097507	12.089273	-0.265351
C	7.280164	12.034936	3.995082
C	6.192780	12.045312	5.983708
C	5.295110	12.694038	6.883994
C	4.831995	14.014264	6.657782
H	5.175424	14.601462	5.815892
C	3.890438	14.589093	7.532620
H	3.532403	15.591816	7.348223
C	3.841077	12.704771	8.851323
H	3.447822	12.202099	9.723609
C	4.781191	12.046313	8.034142
H	5.090770	11.045785	8.306878
C	7.228778	10.156415	5.267967
C	7.584596	8.785586	5.452750
C	7.205156	8.057088	6.608400
H	6.637697	8.519023	7.406255
C	8.338421	8.071999	4.485448
H	8.681631	8.541952	3.572321
C	8.670639	6.721516	4.706477
H	9.253499	6.181271	3.973362
C	7.570008	6.702551	6.736928
H	7.269648	6.145485	7.613054
C	3.396641	3.474674	-2.924415
H	4.295871	3.687720	-3.484341
C	3.455881	3.315742	-1.524703
H	4.416935	3.407743	-1.036076
C	1.119235	3.110840	-2.952945
H	0.205727	3.064645	-3.527137
C	1.080654	2.943406	-1.552655
H	0.125331	2.744145	-1.085341
C	2.270895	3.057618	-0.789048
C	2.267361	2.956725	0.636670
C	1.100146	2.933852	2.582035
C	3.362365	3.106034	2.622334
C	4.564379	3.279489	3.373229
C	4.551602	3.393865	4.787688
H	3.633150	3.329845	5.356125
C	5.834747	3.377779	2.751874
H	5.944806	3.288969	1.679328
C	6.986174	3.592518	3.536501
H	7.954734	3.671747	3.064024
C	5.752993	3.610324	5.489081
H	5.744852	3.702576	6.565856
C	-0.144977	2.969612	3.277498
C	-1.385855	2.866346	2.601699
H	-1.432211	2.722587	1.530617
C	-2.590312	2.910078	3.332918
H	-3.535825	2.824306	2.819381
C	-1.463037	3.142285	5.331626
H	-1.511624	3.240173	6.406648
C	-0.212744	3.117166	4.686243
H	0.678820	3.199155	5.294327
C	-3.768263	13.926580	0.612787
H	-3.535701	14.957392	0.384968
C	-4.869572	11.992178	0.011073
H	-5.510354	11.484151	-0.695587
C	-4.406805	11.314085	1.156075
H	-4.711224	10.286220	1.303193
C	-3.561223	11.978465	2.080495
C	-3.256909	13.331008	1.782878
H	-2.633201	13.928841	2.434853
C	-3.038658	11.317826	3.233953
C	-2.856807	9.448569	4.510362
C	-1.755046	11.341253	5.108221
N	-4.023435	5.412288	5.347485
C	-3.236986	8.100239	4.785295
C	-4.129058	7.386788	3.944702
H	-4.559912	7.840000	3.061626
C	-4.481180	6.058974	4.256209
H	-5.155497	5.517214	3.609900
C	-2.758896	7.400751	5.922159
H	-2.090578	7.865875	6.634587
C	-3.176637	6.076770	6.159846
H	-2.825181	5.548387	7.034587
C	-0.932034	12.050117	6.036565
C	-0.413747	11.432287	7.203366
H	-0.615597	10.394113	7.432984
C	0.364472	12.178854	8.110551
H	0.748115	11.712376	9.007028
C	0.188977	14.082425	6.819475
H	0.436734	15.126833	6.687603
C	-0.603134	13.418806	5.860627
H	-0.957152	13.982415	5.006729
C	3.281026	6.245112	-5.170448
H	4.227849	5.730442	-5.246998
C	0.998800	6.192689	-5.462651

H	0.122228	5.633927	-5.754707
C	0.861658	7.528228	-5.025118
H	-0.128268	7.963257	-4.998990
C	3.243032	7.577559	-4.713990
H	4.177076	8.051818	-4.443413
C	2.002489	8.256833	-4.602973
C	1.909505	9.579641	-4.069078
C	0.669804	11.357459	-3.386681
C	2.928849	11.394888	-3.157019
C	-0.594329	11.992894	-3.184570
C	-1.821270	11.363459	-3.515375
H	-1.847069	10.382027	-3.969675
C	-3.042847	12.023968	-3.264012
H	-3.977592	11.540233	-3.508333
C	-1.960624	13.876816	-2.417172
H	-2.034251	14.867485	-1.990767
C	-0.696874	13.294187	-2.627743
H	0.178823	13.869357	-2.357273
C	4.113025	12.069710	-2.728690
C	4.072452	13.370898	-2.164819
H	3.139269	13.902909	-2.031306
C	5.267581	14.012642	-1.783997
H	5.238187	15.008123	-1.363911
C	5.400037	11.488688	-2.855765
H	5.537009	10.503791	-3.283094
C	6.540545	12.207903	-2.444879
H	7.523687	11.772413	-2.555101
C	11.075368	14.811563	-0.979591
H	10.677916	13.793263	-0.940628
H	11.983153	14.773147	-1.594700
H	11.404155	15.074031	0.033558
C	9.769159	16.835182	-0.543171
H	8.709449	16.826627	-0.270061
H	10.349291	16.743904	0.383570
H	9.980690	17.832942	-0.947904
C	10.490409	16.306674	-2.836643
H	10.776223	17.358155	-2.704371
H	11.419791	15.827179	-3.169270
C	9.534872	16.303545	-4.065213
H	9.326629	17.352664	-4.313005
H	10.110295	15.925714	-4.920663
C	7.068704	16.412322	-3.886866
H	6.603638	16.363444	-2.897247
H	7.303801	17.465832	-4.085720
H	6.316985	16.110653	-4.627325
C	8.150638	14.416408	-4.872265
H	7.211103	14.445736	-5.438769
H	8.962958	14.400425	-5.610277
H	8.185886	13.465684	-4.331574
N	-4.863556	1.313215	5.986942
N	-6.287541	3.758578	6.691032
C	-5.028732	0.684897	4.649504
H	-4.252871	-0.058245	4.426622
H	-5.990155	0.169809	4.531946
H	-4.985957	1.450331	3.869441
C	-3.693599	0.737848	6.703152
H	-3.147847	-0.002196	6.104596
H	-2.988477	1.530838	6.967216
H	-3.965419	0.234088	7.639234
C	-6.106278	1.170742	6.801263
H	-5.902101	0.642511	7.741317
H	-6.836272	0.530974	6.288770
C	-6.859225	2.469885	7.180746
H	-7.889029	2.340608	6.823777
H	-6.953617	2.470933	8.274381
C	-7.204616	4.413895	5.721538
H	-7.444429	5.449464	5.993215
H	-6.745722	4.429677	4.729187
H	-8.164367	3.893108	5.613970
C	-5.977814	4.666523	7.827981
H	-6.453831	5.650160	7.729623
H	-6.298192	4.267439	8.798607
H	-4.898388	4.829149	7.896936
N	9.348605	1.689894	6.107734
N	10.505338	4.262356	7.159210
N	10.045251	15.743156	-1.512415
N	8.270969	15.549195	-3.925197
N	-7.065967	15.027058	-1.595914
N	-5.389904	14.955121	-4.081203
N	6.570944	12.750545	4.894318
N	6.488533	10.747836	6.232809
N	7.615065	10.731989	4.109856
N	8.294686	6.042488	5.808210
N	3.388956	13.954274	8.611805
N	2.252934	3.379692	-3.633334
N	3.457088	3.095132	1.271243
N	1.057272	2.847110	1.233591
N	2.214834	3.058871	3.331098

N	-2.635695	3.037387	4.675260
N	6.949816	3.709967	4.879844
N	-4.557916	13.274092	-0.264108
N	-3.374846	10.018401	3.399649
N	-1.996585	10.036891	5.372497
N	-2.216128	12.028772	4.039199
N	0.663725	13.480395	7.928894
N	2.184133	5.549500	-5.534479
N	0.669949	10.109952	-3.917306
N	1.760032	12.043541	-2.978102
N	3.070758	10.154571	-3.674242
N	6.484350	13.449665	-1.921577
N	-3.117720	13.259720	-2.726121
Pd	1.583306	14.595801	9.453594
Pd	-4.962518	14.092239	-2.150536
Pd	8.705315	3.987835	5.999635
Pd	8.225958	14.601721	-1.716113
Pd	2.273813	3.444817	-5.751131
Pd	-4.453313	3.381541	5.668864
C	4.863960	6.666601	3.719554
C	4.172389	6.426518	2.554678
C	2.754219	6.396730	2.551681
C	2.046667	6.625807	3.778029
C	2.792946	6.833923	4.968252
C	4.170147	6.850520	4.938714
H	2.525396	5.942708	0.437564
H	5.946441	6.708046	3.700320
H	4.705457	6.257567	1.625333
C	2.014904	6.147465	1.371447
C	0.626996	6.654348	3.755983
H	2.262375	6.969986	5.904204
H	4.737201	6.966241	5.854538
C	-0.070090	6.434375	2.589158
C	0.639821	6.140129	1.386341
H	0.069765	6.860205	4.664511
C	2.712816	8.935238	-0.600125
C	3.415425	7.784143	-1.250084
C	4.647452	7.714265	-0.735919
C	4.794160	8.776208	0.316314
H	2.890703	7.138173	-1.948318
H	5.449486	7.022921	-0.954465
C	3.159788	10.512915	1.288851
C	2.681300	9.800768	2.564390
C	4.255611	11.543508	1.566715
H	2.305461	11.017887	0.828586
C	2.270557	10.808091	3.645638
H	3.494792	9.173717	2.945860
H	1.851399	9.132646	2.314255
C	3.855325	12.517013	2.685816
H	5.164713	11.016032	1.865337
H	4.487210	12.087407	0.642855
C	3.436735	11.760157	3.951369
H	1.967713	10.264886	4.547534
H	1.400203	11.387102	3.309490
H	4.727488	13.152003	2.846808
H	3.036852	13.167048	2.360208
H	3.169326	12.459342	4.751517
H	4.269650	11.137294	4.305619
N	3.597871	9.523202	0.287570
O	5.732038	8.945809	1.066374
O	1.574502	9.340833	-0.803520
O	1.073765	6.043669	-2.307907
H	0.902199	5.670099	-1.431059
H	0.420856	6.770733	-2.305288
O	-0.721717	8.034316	-1.614181
H	-1.154472	8.575964	-2.284246
H	0.055335	8.549252	-1.317584
O	-0.010685	5.834869	0.228127
O	-1.439705	6.475120	2.587823
C	-1.443608	5.718552	0.289533
H	-1.805300	5.923102	-0.716977
H	-1.709910	4.696159	0.584189
C	-1.988268	6.725179	1.277663
H	-3.070669	6.634588	1.368765
H	-1.729729	7.736566	0.944395

Table5_1e_TSi_reactant_2.wat

**Property**	**Value**
Charge	0
Electronic Energy, BS1 (a.u.)	-1477.807242
Thermal and entropic correction, BS1 (a.u.)	2.821705
Electronic Energy, BS2 (a.u.)	-1478.342619

Number of Imaginary Frequencies	0
Imaginary frequencies (cm-1)	None

Molecular Geometry in Cartesian Coordinates

C	-1.224596	-10.795081	-3.100902
H	-1.098679	-11.846934	-2.815052
H	-1.106458	-10.204113	-2.188086
H	-2.266235	-10.687361	-3.428763
C	-0.940109	-9.863388	-5.348821
H	-2.033361	-9.895561	-5.260558
H	-0.658767	-8.827101	-5.554614
H	-0.687083	-10.441740	-6.246255
C	0.721062	-11.433405	-4.456324
H	0.664007	-11.696583	-5.520323
H	0.455622	-12.363150	-3.936885
C	2.216272	-11.172298	-4.151652
H	2.754476	-11.299282	-5.099675
H	2.555767	-12.015739	-3.536892
C	3.516351	-9.101734	-4.353400
H	3.035546	-8.190744	-4.721213
H	4.422550	-8.800932	-3.813037
H	3.855791	-9.656778	-5.236968
C	3.121918	-10.088693	-2.145231
H	2.443077	-9.675249	-1.394848
H	3.253495	-11.149211	-1.896768
H	4.101526	-9.615429	-2.002761
N	7.074738	-3.123949	7.608038
N	5.253608	-1.874506	9.515899
C	6.892305	-4.508362	7.097595
H	5.841263	-4.679371	6.850827
H	7.481069	-4.711824	6.194431
H	7.173567	-5.278246	7.827060
C	8.067047	-2.381662	6.785706
H	7.606122	-1.484452	6.363266
H	8.939629	-2.046563	7.360282
H	8.459868	-2.974989	5.950512
C	7.475370	-3.134413	9.045671
H	7.631987	-4.161293	9.400187
H	8.451860	-2.652720	9.183939
C	6.511312	-2.467575	10.057295
H	7.100016	-1.714021	10.596090
H	6.288674	-3.226587	10.818205
C	5.194542	-0.413929	9.790461
H	4.277303	-0.113942	10.312617
H	6.028857	-0.056410	10.406977
H	5.236698	0.145744	8.851769
C	4.058082	-2.567201	10.065964
H	3.526008	-3.088512	9.265291
H	4.307031	-3.322324	10.822061
H	3.347076	-1.880049	10.541733
N	-6.826428	1.073762	-8.140908
N	-5.593210	3.574348	-9.006572
C	-6.310240	-0.277853	-8.484755
H	-6.716409	-1.067332	-7.840110
H	-6.536228	-0.576826	-9.516057
H	-5.221805	-0.298496	-8.386023
C	-7.824743	0.998843	-7.041524
H	-7.964845	-0.020431	-6.660239
H	-7.504464	1.623514	-6.203182
H	-8.819304	1.355827	-7.337084
C	-7.405785	1.742315	-9.342632
H	-8.468185	1.971378	-9.189562
H	-7.399679	1.066762	-10.207601
C	-6.738317	3.058308	-9.813188
H	-6.442597	2.900857	-10.858345
H	-7.540172	3.805368	-9.872660
C	-5.924408	4.887472	-8.391787
H	-5.194787	5.670213	-8.635131
H	-6.904174	5.273421	-8.699925
H	-5.953313	4.793178	-7.302759
C	-4.361057	3.671003	-9.833853
H	-3.915425	4.673462	-9.821207
H	-3.605571	2.972364	-9.462917
H	-4.529062	3.423414	-10.889419
C	-0.410152	8.730965	5.963572
H	-0.852206	8.011193	6.663761
H	0.360750	8.205212	5.393896
H	0.109013	9.477718	6.577450
C	-2.780762	8.841718	5.349662
H	-2.798684	8.101351	6.159224
H	-3.465100	9.644522	5.651397
H	-3.220308	8.375046	4.464046
C	-1.352009	10.817698	5.083548
H	-0.608070	11.160682	5.813896
H	-2.298605	11.234335	5.450984
C	-1.040633	11.554580	3.757605
H	-1.871336	12.251893	3.589527

H	-0.181676	12.206991	3.960323
C	-1.830955	10.972237	1.506631
H	-1.401466	11.264867	0.540298
H	-2.425185	10.068908	1.341816
H	-2.535058	11.766802	1.783503
C	0.567663	10.949201	2.005862
H	0.566270	11.222804	0.943331
H	1.108831	11.748177	2.528033
H	1.170645	10.042872	2.109454
N	6.672441	1.292567	-3.578401
C	6.742172	1.393993	-2.234887
H	7.238081	2.268110	-1.836971
C	6.214595	0.425243	-1.357795
H	6.327476	0.578232	-0.292513
C	5.572356	-0.726880	-1.877759
C	5.492654	-0.808495	-3.291173
H	5.024291	-1.649972	-3.785213
C	6.047999	0.211477	-4.088551
H	5.986801	0.145876	-5.165734
C	5.047792	-1.750172	-1.030739
C	4.727482	-2.613301	1.043257
C	4.855919	-2.509516	2.462075
C	5.444541	-1.379997	3.085732
H	5.828935	-0.549775	2.507026
C	5.567630	-1.335391	4.489249
H	6.032141	-0.482508	4.964007
C	4.567276	-3.401904	4.724659
H	4.229104	-4.188593	5.383852
C	4.410773	-3.537534	3.331552
H	3.958774	-4.444426	2.951368
C	3.998372	-3.748601	-0.783191
C	3.273128	-4.844031	-1.343860
C	2.775250	-5.903962	-0.543394
H	2.944534	-5.934466	0.525099
C	2.996865	-4.931406	-2.731986
H	3.329718	-4.170964	-3.426263
C	2.269891	-6.026995	-3.238966
H	2.054360	-6.088046	-4.296367
C	2.059073	-6.961000	-1.139120
H	1.680873	-7.768355	-0.528735
C	-3.937784	-0.174126	-6.034307
H	-3.414379	-0.227122	-6.978487
C	-3.779252	-1.202327	-5.086722
H	-3.129422	-2.031231	-5.336106
C	-5.365861	0.992131	-4.648285
H	-5.982980	1.865794	-4.494433
C	-5.262345	0.015654	-3.636704
H	-5.819805	0.157660	-2.720030
C	-4.456622	-1.132497	-3.842769
C	-4.332416	-2.161764	-2.861307
C	-4.796884	-2.996884	-0.802612
C	-3.461270	-4.158276	-2.219753
C	-2.636309	-5.285806	-2.511657
C	-2.371472	-6.301254	-1.559970
H	-2.818516	-6.280572	-0.575140
C	-2.020398	-5.448618	-3.777554
H	-2.164008	-4.736266	-4.579505
C	-1.177265	-6.548174	-4.015325
H	-0.700854	-6.653315	-4.979427
C	-1.515537	-7.371360	-1.894857
H	-1.318599	-8.147854	-1.169919
C	-5.473633	-2.887524	0.450580
C	-6.333268	-1.798908	0.747685
H	-6.508074	-1.002726	0.035519
C	-7.012904	-1.752932	1.981320
H	-7.679610	-0.930442	2.200415
C	-6.058331	-3.737783	2.671424
H	-5.960986	-4.490469	3.440892
C	-5.344811	-3.871751	1.463629
H	-4.723547	-4.747570	1.328470
C	0.046994	5.951895	4.006799
H	0.993752	6.466694	3.927002
C	-2.227140	5.968440	3.630284
H	-3.091229	6.498262	3.256027
C	-2.373305	4.667525	4.155195
H	-3.361564	4.227724	4.186823
C	-1.239031	3.968117	4.638562
C	-0.001567	4.654219	4.548725
H	0.925023	4.206328	4.883266
C	-1.326441	2.645795	5.169674
C	-2.508428	0.749578	5.559869
C	-0.279200	0.796086	5.966864
N	-6.218857	-1.397958	5.358940
C	-3.741663	0.032366	5.495151
C	-4.947298	0.652140	5.081065
H	-4.985352	1.700634	4.814725
C	-6.140784	-0.092921	5.026866
H	-7.057105	0.387889	4.715058

C	-3.837884	-1.339131	5.841019
H	-2.979021	-1.896866	6.190228
C	-5.079454	-2.001523	5.756969
H	-5.151628	-3.044450	6.031509
C	0.933247	0.149598	6.356683
C	0.976971	-1.213883	6.742589
H	0.080672	-1.819977	6.772124
C	2.202491	-1.797264	7.127514
H	2.233373	-2.833602	7.431982
C	3.341735	0.178791	6.793816
H	4.278503	0.716200	6.834684
C	2.170197	0.843059	6.386040
H	2.242741	1.890672	6.121845
C	-2.299290	2.992299	-6.950772
H	-2.153539	2.323844	-7.787716
C	-3.760214	4.183097	-5.628108
H	-4.779518	4.466840	-5.409362
C	-2.709908	4.699783	-4.846178
H	-2.958340	5.382260	-4.043858
C	-1.183641	3.458458	-6.226471
H	-0.197193	3.136974	-6.534283
C	-1.368722	4.339354	-5.132134
C	-0.274293	4.831189	-4.358716
C	0.467416	5.946729	-2.524864
C	1.943559	4.896391	-3.890916
C	0.169358	6.637512	-1.311719
C	-1.154230	7.017482	-0.970202
H	-1.989941	6.826600	-1.630534
C	-1.407471	7.669611	0.251836
H	-2.416427	7.958099	0.509448
C	0.823112	7.612050	0.834736
H	1.588513	7.849806	1.559092
C	1.172173	6.958809	-0.363850
H	2.210905	6.707016	-0.530963
C	3.286342	4.534656	-4.214306
C	4.386823	4.944717	-3.421280
H	4.258071	5.583542	-2.556794
C	5.689179	4.525586	-3.754983
H	6.523785	4.840702	-3.144620
C	3.600112	3.733603	-5.340639
H	2.834811	3.392612	-6.026345
C	4.936674	3.365455	-5.591629
H	5.169082	2.752003	-6.450626
C	9.178288	0.127390	-5.461313
H	8.149061	0.146968	-5.829831
H	9.812919	-0.128130	-6.319078
H	9.262258	-0.709143	-4.756416
C	9.957605	1.267568	-3.437404
H	9.328888	1.869816	-2.776098
H	9.896960	0.227163	-3.094467
H	10.992500	1.587907	-3.263030
C	10.572626	2.137462	-5.646064
H	11.456175	2.351859	-5.031245
H	10.953823	1.487691	-6.444262
C	10.175923	3.471058	-6.325635
H	10.905845	4.219144	-5.990742
H	10.391069	3.348655	-7.394946
C	8.776666	5.292941	-5.464428
H	8.331207	5.221292	-4.468492
H	9.782173	5.711129	-5.330412
H	8.205708	6.042619	-6.026396
C	8.032606	3.994208	-7.403637
H	7.612509	4.983139	-7.626104
H	8.648174	3.719111	-8.269225
H	7.200567	3.284770	-7.371284
N	-9.568045	-3.686167	4.153872
N	-8.865099	-2.365551	6.657130
C	-10.148842	-3.003535	2.966975
H	-10.078600	-3.603996	2.051424
H	-11.210343	-2.754070	3.088830
H	-9.624842	-2.061283	2.783745
C	-9.075515	-5.043154	3.797587
H	-9.183818	-5.270788	2.729717
H	-8.015111	-5.132627	4.048118
H	-9.593671	-5.845913	4.336965
C	-10.560757	-3.755737	5.265890
H	-10.795722	-4.798661	5.514637
H	-11.524945	-3.330435	4.959091
C	-10.180890	-3.067179	6.599620
H	-11.002348	-2.379150	6.837320
H	-10.246341	-3.839265	7.376921
C	-9.049595	-0.914353	6.926428
H	-8.476332	-0.564301	7.794033
H	-8.725583	-0.329169	6.061684
H	-10.094529	-0.639889	7.118256
C	-7.974583	-2.985021	7.674299
H	-7.606475	-2.264784	8.415675
H	-8.460101	-3.789040	8.241521

H	-7.101599	-3.427354	7.185453
N	-0.244973	-10.345549	-4.125398
N	2.566335	-9.872544	-3.507377
N	9.522442	1.437922	-4.849292
N	8.780017	3.957012	-6.118542
N	-1.410416	9.327470	5.038629
N	-0.798757	10.716813	2.546036
N	5.172894	-1.571953	0.304699
N	4.128699	-3.725748	0.562315
N	4.435678	-2.793960	-1.634964
N	1.812417	-7.030933	-2.462994
N	5.142918	-2.326868	5.299727
N	-4.723837	0.901681	-5.831576
N	-3.563473	-3.221711	-3.186925
N	-4.974132	-1.985254	-1.684068
N	-4.010283	-4.083993	-0.985027
N	-6.886142	-2.704694	2.928197
N	-0.916220	-7.501162	-3.098167
N	-1.041092	6.608793	3.559790
N	-2.536023	2.044048	5.162809
N	-1.410314	0.054607	5.947125
N	-0.170576	2.084309	5.581627
N	3.368203	-1.117198	7.162566
N	-3.571233	3.343948	-6.666733
N	-0.581943	5.623947	-3.310618
N	1.755733	5.605301	-2.754155
N	0.966389	4.407177	-4.688333
N	5.975288	3.742594	-4.816356
N	-0.441058	7.968465	1.142810
Pd	5.202998	-2.117317	7.392984
Pd	-0.928721	8.652864	3.066163
Pd	0.807290	-8.684021	-3.297714
Pd	7.742533	2.615732	-4.825479
Pd	-5.173124	2.219516	-7.414792
Pd	-7.878809	-2.539621	4.774314
C	1.159457	-3.297842	3.095657
C	1.302622	-1.933018	2.953054
C	1.565066	-1.343769	1.669903
C	1.274340	-2.165700	0.523689
C	0.789292	-3.495438	0.735557
C	0.906008	-4.104383	1.963030
H	1.071465	-3.745607	4.088402
H	1.282473	-1.304750	3.835943
C	2.086506	-0.046955	1.489694
C	1.490445	-1.671556	-0.766281
H	0.416185	-4.042678	-0.130620
H	0.647808	-5.155868	2.103290
C	1.992060	-0.383691	-0.959064
C	2.280916	0.452259	0.179297
H	1.246464	-2.298193	-1.619644
C	-1.509225	-1.628105	0.272733
C	-1.445229	-2.540795	1.435895
C	-1.224029	-1.823881	2.519019
C	-1.143537	-0.386792	2.142102
H	-1.723073	-3.611486	1.325875
H	-1.249457	-2.108036	3.587948
C	-1.382978	0.834754	-0.140529
C	-2.802461	1.025518	-0.694477
C	-0.859127	2.132112	0.487155
H	-0.712859	0.579888	-0.970262
C	-2.817642	2.151042	-1.736696
H	-3.469702	1.276459	0.137813
H	-3.161849	0.093916	-1.135398
C	-0.854675	3.242784	-0.571409
H	-1.501245	2.421710	1.322257
H	0.143521	1.992893	0.884751
C	-2.262288	3.452169	-1.143410
H	-3.839292	2.303052	-2.099207
H	-2.212802	1.830761	-2.596772
H	-0.456398	4.150837	-0.108105
H	-0.156253	2.970829	-1.376406
H	-2.271023	4.243663	-1.896353
H	-2.928830	3.787117	-0.339166
N	-1.341447	-0.330464	0.757845
O	-0.963803	0.572642	2.878666
O	-1.691475	-1.901941	-0.911029
C	2.717202	1.793380	-0.080462
C	2.196429	0.119394	-2.278838
C	2.612644	1.407715	-2.479348
H	2.744158	1.781663	-3.488526
C	2.857427	2.255263	-1.360306
H	3.143278	3.290065	-1.514625
H	2.851457	2.488786	0.735603
H	2.012889	-0.546390	-3.117596
C	2.459470	0.818152	2.679886
H	3.417445	1.306387	2.495431
H	2.592028	0.195509	3.574007
O	1.531779	1.872239	2.943660

H	0.634945	1.496296	3.008081
O	-0.070913	-3.265727	-2.953197
H	-0.236740	-2.495255	-3.524054
H	-0.749961	-3.144767	-2.272527
O	1.749343	4.422214	1.715309
H	2.662104	4.665366	1.923652
H	1.623209	3.585657	2.200479
O	-1.035228	-0.516076	-3.339345
H	-1.285428	-0.822846	-2.452277
H	-0.233748	0.002123	-3.199607

Table5_1e_TSi_TSi-ii_h2o_2

Property	Value
Charge	0
Electronic Energy, BS1 (a.u.)	-1477.769255
Thermal and entropic correction, BS1 (a.u.)	2.824169
Electronic Energy, BS2 (a.u.)	-1478.300900
Number of Imaginary Frequencies	0
Imaginary frequencies (cm-1)	None

Molecular Geometry in Cartesian Coordinates

C	-1.224596	-10.795081	-3.100902
H	-1.098679	-11.846934	-2.815052
H	-1.106458	-10.204113	-2.188086
H	-2.266235	-10.687361	-3.428763
C	-0.940109	-9.863388	-5.348821
H	-2.033361	-9.895561	-5.260558
H	-0.658767	-8.827101	-5.554614
H	-0.687083	-10.441740	-6.246255
C	0.721062	-11.433405	-4.456324
H	0.664007	-11.696583	-5.520323
H	0.455622	-12.363150	-3.936885
C	2.216272	-11.172298	-4.151652
H	2.754476	-11.299282	-5.099675
H	2.555767	-12.015739	-3.536892
C	3.516351	-9.101734	-4.353400
H	3.035546	-8.190744	-4.721213
H	4.422550	-8.800932	-3.813037
H	3.855791	-9.656778	-5.236968
C	3.121918	-10.088693	-2.145231
H	2.443077	-9.675249	-1.394848
H	3.253495	-11.149211	-1.896768
H	4.101526	-9.615429	-2.002761
N	7.074738	-3.123949	7.608038
N	5.253608	-1.874506	9.515899
C	6.892305	-4.508362	7.097595
H	5.841263	-4.679371	6.850827
H	7.481069	-4.711824	6.194431
H	7.173567	-5.278246	7.827060
C	8.067047	-2.381662	6.785706
H	7.606122	-1.484452	6.363266
H	8.939629	-2.046563	7.360282
H	8.459868	-2.974989	5.950512
C	7.475370	-3.134413	9.045671
H	7.631987	-4.161293	9.400187
H	8.451860	-2.652720	9.183939
C	6.511312	-2.467575	10.057295
H	7.100016	-1.714021	10.596090
H	6.288674	-3.226587	10.818205
C	5.194542	-0.413929	9.790461
H	4.277303	-0.113942	10.312617
H	6.028857	-0.056410	10.406977
H	5.236698	0.145744	8.851769
C	4.058082	-2.567201	10.065964
H	3.526008	-3.088512	9.265291
H	4.307031	-3.322324	10.822061
H	3.347076	-1.880049	10.541733
N	-6.826428	1.073762	-8.140908
N	-5.593210	3.574348	-9.006572
C	-6.310240	-0.277853	-8.484755
H	-6.716409	-1.067332	-7.840110
H	-6.536228	-0.576826	-9.516057
H	-5.221805	-0.298496	-8.386023
C	-7.824743	0.998843	-7.041524
H	-7.964845	-0.020431	-6.660239
H	-7.504464	1.623514	-6.203182
H	-8.819304	1.355827	-7.337084
C	-7.405785	1.742315	-9.342632
H	-8.468185	1.971378	-9.189562
H	-7.399679	1.066762	-10.207601
C	-6.738317	3.058308	-9.813188
H	-6.442597	2.900857	-10.858345

H	-7.540172	3.805368	-9.872660
C	-5.924408	4.887472	-8.391787
H	-5.194787	5.670213	-8.635131
H	-6.904174	5.273421	-8.699925
H	-5.953313	4.793178	-7.302759
C	-4.361057	3.671003	-9.833853
H	-3.915425	4.673462	-9.821207
H	-3.605571	2.972364	-9.462917
H	-4.529062	3.423414	-10.889419
C	-0.410152	8.730965	5.963572
H	-0.852206	8.011193	6.663761
H	0.360750	8.205212	5.393896
H	0.109013	9.477718	6.577450
C	-2.780762	8.841718	5.349662
H	-2.798684	8.101351	6.159224
H	-3.465100	9.644522	5.651397
H	-3.220308	8.375046	4.464046
C	-1.352009	10.817698	5.083548
H	-0.608070	11.160682	5.813896
H	-2.298605	11.234335	5.450984
C	-1.040633	11.554580	3.757605
H	-1.871336	12.251893	3.589527
H	-0.181676	12.206991	3.960323
C	-1.830955	10.972237	1.506631
H	-1.401466	11.264867	0.540298
H	-2.425185	10.068908	1.341816
H	-2.535058	11.766802	1.783503
C	0.567663	10.949201	2.005862
H	0.566270	11.222804	0.943331
H	1.108831	11.748177	2.528033
H	1.170645	10.042872	2.109454
N	6.672441	1.292567	-3.578401
C	6.742172	1.393993	-2.234887
H	7.238081	2.268110	-1.836971
C	6.214595	0.425243	-1.357795
H	6.327476	0.578232	-0.292513
C	5.572356	-0.726880	-1.877759
C	5.492654	-0.808495	-3.291173
H	5.024291	-1.649972	-3.785213
C	6.047999	0.211477	-4.088551
H	5.986801	0.145876	-5.165734
C	5.047792	-1.750172	-1.030739
C	4.727482	-2.613301	1.043257
C	4.855919	-2.509516	2.462075
C	5.444541	-1.379997	3.085732
H	5.828935	-0.549775	2.507026
C	5.567630	-1.335391	4.489249
H	6.032141	-0.482508	4.964007
C	4.567276	-3.401904	4.724659
H	4.229104	-4.188593	5.383852
C	4.410773	-3.537534	3.331552
H	3.958774	-4.444426	2.951368
C	3.998372	-3.748601	-0.783191
C	3.273128	-4.844031	-1.343860
C	2.775250	-5.903962	-0.543394
H	2.944534	-5.934466	0.525099
C	2.996865	-4.931406	-2.731986
H	3.329718	-4.170964	-3.426263
C	2.269891	-6.026995	-3.238966
H	2.054360	-6.088046	-4.296367
C	2.059073	-6.961000	-1.139120
H	1.680873	-7.768355	-0.528735
C	-3.937784	-0.174126	-6.034307
H	-3.414379	-0.227122	-6.978487
C	-3.779252	-1.202327	-5.086722
H	-3.129422	-2.031231	-5.336106
C	-5.365861	0.992131	-4.648285
H	-5.982980	1.865794	-4.494433
C	-5.262345	0.015654	-3.636704
H	-5.819805	0.157660	-2.720030
C	-4.456622	-1.132497	-3.842769
C	-4.332416	-2.161764	-2.861307
C	-4.796884	-2.996884	-0.802612
C	-3.461270	-4.158276	-2.219753
C	-2.636309	-5.285806	-2.511657
C	-2.371472	-6.301254	-1.559970
H	-2.818516	-6.280572	-0.575140
C	-2.020398	-5.448618	-3.777554
H	-2.164008	-4.736266	-4.579505
C	-1.177265	-6.548174	-4.015325
H	-0.700854	-6.653315	-4.979427
C	-1.515537	-7.371360	-1.894857
H	-1.318599	-8.147854	-1.169919
C	-5.473633	-2.887524	0.450580
C	-6.333268	-1.798908	0.747685
H	-6.508074	-1.002726	0.035519
C	-7.012904	-1.752932	1.981320
H	-7.679610	-0.930442	2.200415

C	-6.058331	-3.737783	2.671424
H	-5.960986	-4.490469	3.440892
C	-5.344811	-3.871751	1.463629
H	-4.723547	-4.747570	1.328470
C	0.046994	5.951895	4.006799
H	0.993752	6.466694	3.927002
C	-2.227140	5.968440	3.630284
H	-3.091229	6.498262	3.256027
C	-2.373305	4.667525	4.155195
H	-3.361564	4.227724	4.186823
C	-1.239031	3.968117	4.638562
C	-0.001567	4.654219	4.548725
H	0.925023	4.206328	4.883266
C	-1.326441	2.645795	5.169674
C	-2.508428	0.749578	5.559869
C	-0.279200	0.796086	5.966864
N	-6.218857	-1.397958	5.358940
C	-3.741663	0.032366	5.495151
C	-4.947298	0.652140	5.081065
H	-4.985352	1.700634	4.814725
C	-6.140784	-0.092921	5.026866
H	-7.057105	0.387889	4.715058
C	-3.837884	-1.339131	5.841019
H	-2.979021	-1.896866	6.190228
C	-5.079454	-2.001523	5.756969
H	-5.151628	-3.044450	6.031509
C	0.933247	0.149598	6.356683
C	0.976971	-1.213883	6.742589
H	0.080672	-1.819977	6.772124
C	2.202491	-1.797264	7.127514
H	2.233373	-2.833602	7.431982
C	3.341735	0.178791	6.793816
H	4.278503	0.716200	6.834684
C	2.170197	0.843059	6.386040
H	2.242741	1.890672	6.121845
C	-2.299290	2.992299	-6.950772
H	-2.153539	2.323844	-7.787716
C	-3.760214	4.183097	-5.628108
H	-4.779518	4.466840	-5.409362
C	-2.709908	4.699783	-4.846178
H	-2.958340	5.382260	-4.043858
C	-1.183641	3.458458	-6.226471
H	-0.197193	3.136974	-6.534283
C	-1.368722	4.339354	-5.132134
C	-0.274293	4.831189	-4.358716
C	0.467416	5.946729	-2.524864
C	1.943559	4.896391	-3.890916
C	0.169358	6.637512	-1.311719
C	-1.154230	7.017482	-0.970202
H	-1.989941	6.826600	-1.630534
C	-1.407471	7.669611	0.251836
H	-2.416427	7.958099	0.509448
C	0.823112	7.612050	0.834736
H	1.588513	7.849806	1.559092
C	1.172173	6.958809	-0.363850
H	2.210905	6.707016	-0.530963
C	3.286342	4.534656	-4.214306
C	4.386823	4.944717	-3.421280
H	4.258071	5.583542	-2.556794
C	5.689179	4.525586	-3.754983
H	6.523785	4.840702	-3.144620
C	3.600112	3.733603	-5.340639
H	2.834811	3.392612	-6.026345
C	4.936674	3.365455	-5.591629
H	5.169082	2.752003	-6.450626
C	9.178288	0.127390	-5.461313
H	8.149061	0.146968	-5.829831
H	9.812919	-0.128130	-6.319078
H	9.262258	-0.709143	-4.756416
C	9.957605	1.267568	-3.437404
H	9.328888	1.869816	-2.776098
H	9.896960	0.227163	-3.094467
H	10.992500	1.587907	-3.263030
C	10.572626	2.137462	-5.646064
H	11.456175	2.351859	-5.031245
H	10.953823	1.487691	-6.444262
C	10.175923	3.471058	-6.325635
H	10.905845	4.219144	-5.990742
H	10.391069	3.348655	-7.394946
C	8.776666	5.292941	-5.464428
H	8.331207	5.221292	-4.468492
H	9.782173	5.711129	-5.330412
H	8.205708	6.042619	-6.026396
C	8.032606	3.994208	-7.403637
H	7.612509	4.983139	-7.626104
H	8.648174	3.719111	-8.269225
H	7.200567	3.284770	-7.371284
N	-9.568045	-3.686167	4.153872

N	-8.865099	-2.365551	6.657130
C	-10.148842	-3.003535	2.966975
H	-10.078600	-3.603996	2.051424
H	-11.210343	-2.754070	3.088830
H	-9.624842	-2.061283	2.783745
C	-9.075515	-5.043154	3.797587
H	-9.183818	-5.270788	2.729717
H	-8.015111	-5.132627	4.048118
H	-9.593671	-5.845913	4.336965
C	-10.560757	-3.755737	5.265890
H	-10.795722	-4.798661	5.514637
H	-11.524945	-3.330435	4.959091
C	-10.180890	-3.067179	6.599620
H	-11.002348	-2.379150	6.837320
H	-10.246341	-3.839265	7.376921
C	-9.049595	-0.914353	6.926428
H	-8.476332	-0.564301	7.794033
H	-8.725583	-0.329169	6.061684
H	-10.094529	-0.639889	7.118256
C	-7.974583	-2.985021	7.674299
H	-7.606475	-2.264784	8.415675
H	-8.460101	-3.789040	8.241521
H	-7.101599	-3.427354	7.185453
N	-0.244973	-10.345549	-4.125398
N	2.566335	-9.872544	-3.507377
N	9.522442	1.437922	-4.849292
N	8.780017	3.957012	-6.118542
N	-1.410416	9.327470	5.038629
N	-0.798757	10.716813	2.546036
N	5.172894	-1.571953	0.304699
N	4.128699	-3.725748	0.562315
N	4.435678	-2.793960	-1.634964
N	1.812417	-7.030933	-2.462994
N	5.142918	-2.326868	5.299727
N	-4.723837	0.901681	-5.831576
N	-3.563473	-3.221711	-3.186925
N	-4.974132	-1.985254	-1.684068
N	-4.010283	-4.083993	-0.985027
N	-6.886142	-2.704694	2.928197
N	-0.916220	-7.501162	-3.098167
N	-1.041092	6.608793	3.559790
N	-2.536023	2.044048	5.162809
N	-1.410314	0.054607	5.947125
N	-0.170576	2.084309	5.581627
N	3.368203	-1.117198	7.162566
N	-3.571233	3.343948	-6.666733
N	-0.581943	5.623947	-3.310618
N	1.755733	5.605301	-2.754155
N	0.966389	4.407177	-4.688333
N	5.975288	3.742594	-4.816356
N	-0.441058	7.968465	1.142810
Pd	5.202998	-2.117317	7.392984
Pd	-0.928721	8.652864	3.066163
Pd	0.807290	-8.684021	-3.297714
Pd	7.742533	2.615732	-4.825479
Pd	-5.173124	2.219516	-7.414792
Pd	-7.878809	-2.539621	4.774314
C	1.159457	-3.326703	3.090847
C	1.115027	-1.908967	2.929003
C	1.555446	-1.334149	1.660283
C	1.269530	-2.151270	0.518879
C	0.606507	-3.423286	0.774038
C	0.906008	-4.104383	1.991891
H	1.210959	-3.760037	4.083592
H	1.176650	-1.290320	3.816702
C	2.091316	-0.051765	1.494504
C	1.495255	-1.681176	-0.761471
H	0.319982	-3.999387	-0.101759
H	0.763251	-5.175109	2.079239
C	2.001680	-0.383691	-0.959064
C	2.290536	0.452259	0.174487
H	1.241654	-2.298193	-1.619644
C	-1.489984	-1.637725	0.267923
C	-1.267254	-2.598517	1.378173
C	-1.031623	-1.819071	2.538260
C	-1.124296	-0.391602	2.146912
H	-1.790415	-3.544144	1.364356
H	-1.307179	-2.098416	3.544657
C	-1.387788	0.829944	-0.135719
C	-2.807271	1.025518	-0.694477
C	-0.859127	2.127302	0.487155
H	-0.717669	0.565458	-0.960642
C	-2.817642	2.151042	-1.736696
H	-3.474512	1.276459	0.137813
H	-3.166659	0.093916	-1.135398
C	-0.854675	3.237974	-0.571409
H	-1.501245	2.421710	1.322257
H	0.143521	1.988083	0.884751

C	-2.262288	3.452169	-1.143410
H	-3.839292	2.303052	-2.099207
H	-2.212802	1.830761	-2.596772
H	-0.456398	4.146027	-0.108105
H	-0.156253	2.966019	-1.376406
H	-2.271023	4.243663	-1.896353
H	-2.928830	3.787117	-0.339166
N	-1.351067	-0.344894	0.767465
O	-0.973423	0.577452	2.878666
O	-1.696285	-1.897131	-0.915839
C	2.717202	1.788570	-0.080462
C	2.196429	0.119394	-2.278838
C	2.612644	1.412525	-2.479348
H	2.744158	1.786473	-3.488526
C	2.857427	2.255263	-1.365116
H	3.143278	3.290065	-1.514625
H	2.851457	2.483976	0.735603
H	2.012889	-0.546390	-3.117596
C	2.454660	0.813342	2.684696
H	3.417445	1.296767	2.505051
H	2.582408	0.190699	3.578817
O	1.531779	1.872239	2.943660
H	0.634945	1.496296	3.012891
O	-0.070913	-3.265727	-2.953197
H	-0.236740	-2.495255	-3.524054
H	-0.749961	-3.144767	-2.272527
O	1.749343	4.422214	1.715309
H	2.662104	4.665366	1.918842
H	1.623209	3.585657	2.200479
O	-1.035228	-0.516076	-3.339345
H	-1.290238	-0.822846	-2.452277
H	-0.228938	-0.002687	-3.199607

Table5_1e_TSi_DG_h2o_2

Property	Value
Charge	0
Electronic Energy, BS1 (a.u.)	-1477.812025
Thermal and entropic correction, BS1 (a.u.)	2.828275
Electronic Energy, BS2 (a.u.)	-1478.343008
Number of Imaginary Frequencies	0
Imaginary frequencies (cm-1)	None

Molecular Geometry in Cartesian Coordinates

C	-1.225137	-10.794356	-3.104011
H	-1.099052	-11.846164	-2.818070
H	-1.107724	-10.203324	-2.191140
H	-2.266624	-10.686982	-3.432468
C	-0.939714	-9.862668	-5.351800
H	-2.033007	-9.895141	-5.264121
H	-0.658537	-8.826324	-5.557527
H	-0.686032	-10.441021	-6.249046
C	0.721498	-11.432065	-4.458335
H	0.664587	-11.695751	-5.522216
H	0.456534	-12.361706	-3.938470
C	2.216579	-11.170026	-4.153840
H	2.754761	-11.296607	-5.101928
H	2.556658	-12.013295	-3.539166
C	3.515408	-9.098656	-4.355508
H	3.034017	-8.187934	-4.723221
H	4.421449	-8.797346	-3.815159
H	3.855136	-9.653430	-5.239135
C	3.121664	-10.086071	-2.147413
H	2.442407	-9.673485	-1.396940
H	3.254229	-11.146528	-1.899216
H	4.100819	-9.611923	-2.004803
N	7.074613	-3.124361	7.606264
N	5.254708	-1.874902	9.515287
C	6.891579	-4.508718	7.095890
H	5.840327	-4.679531	6.849901
H	7.479636	-4.712251	6.192282
H	7.173239	-5.278677	7.825122
C	8.066609	-2.382247	6.783401
H	7.605614	-1.484958	6.361210
H	8.939553	-2.047295	7.357513
H	8.458891	-2.975636	5.948001
C	7.476083	-3.134969	9.043661
H	7.632887	-4.161891	9.397974
H	8.452661	-2.653310	9.181418
C	6.512611	-2.468247	10.055916
H	7.101678	-1.714892	10.594590
H	6.290259	-3.227402	10.816767
C	5.195995	-0.414372	9.790187

H	4.279085	-0.114358	10.312902
H	6.030704	-0.057099	10.406312
H	5.237722	0.145496	8.851597
C	4.059380	-2.567564	10.065826
H	3.526789	-3.088590	9.265308
H	4.308639	-3.322924	10.821584
H	3.348737	-1.880438	10.542172
N	-6.827024	1.074769	-8.143199
N	-5.592430	3.575041	-9.007722
C	-6.311300	-0.277026	-8.487040
H	-6.718170	-1.066430	-7.842740
H	-6.536913	-0.575715	-9.518507
H	-5.222925	-0.298202	-8.387774
C	-7.825854	1.000113	-7.044269
H	-7.966555	-0.019159	-6.663192
H	-7.505681	1.624537	-6.205697
H	-8.820130	1.357562	-7.340231
C	-7.405552	1.743785	-9.345063
H	-8.467970	1.973098	-9.192483
H	-7.399181	1.068466	-10.210211
C	-6.737490	3.059740	-9.814880
H	-6.441506	2.902619	-10.860012
H	-7.539072	3.807098	-9.874277
C	-5.923249	4.888136	-8.392680
H	-5.193056	5.670545	-8.635361
H	-6.902635	5.274739	-8.701211
H	-5.952784	4.793481	-7.303699
C	-4.359947	3.671382	-9.834534
H	-3.913866	4.673631	-9.821402
H	-3.604913	2.972276	-9.463549
H	-4.527707	3.424202	-10.890233
C	-0.409248	8.733419	5.963175
H	-0.851230	8.014172	6.663947
H	0.361465	8.207185	5.393688
H	0.110141	9.480522	6.576438
C	-2.779988	8.844031	5.349734
H	-2.797824	8.104312	6.159892
H	-3.464221	9.647122	5.650943
H	-3.219730	8.376685	4.464569
C	-1.351040	10.819685	5.082004
H	-0.606626	11.163046	5.811694
H	-2.297369	11.236669	5.449734
C	-1.040369	11.555685	3.755405
H	-1.871321	12.252645	3.587105
H	-0.181521	12.208480	3.957351
C	-1.831038	10.971753	1.504963
H	-1.401720	11.263722	0.538357
H	-2.425236	10.068269	1.340883
H	-2.535137	11.766480	1.781389
C	0.567654	10.949176	2.003785
H	0.566060	11.221963	0.941044
H	1.108846	11.748596	2.525249
H	1.170723	10.042979	2.107972
N	6.673586	1.292587	-3.578567
C	6.742917	1.394016	-2.235030
H	7.238368	2.268318	-1.836948
C	6.215459	0.425030	-1.358119
H	6.327926	0.578052	-0.292795
C	5.573830	-0.727341	-1.878310
C	5.494579	-0.808963	-3.291740
H	5.026728	-1.650624	-3.785954
C	6.049730	0.211260	-4.088915
H	5.988813	0.145669	-5.166112
C	5.049366	-1.750850	-1.031497
C	4.728641	-2.614064	1.042389
C	4.856340	-2.509929	2.461252
C	5.444190	-1.380012	3.084932
H	5.828532	-0.549763	2.506230
C	5.566570	-1.334997	4.488490
H	6.030522	-0.481818	4.963253
C	4.566886	-3.401843	4.723928
H	4.228638	-4.188472	5.383151
C	4.411171	-3.537900	3.330760
H	3.959685	-4.445026	2.950530
C	4.000246	-3.749497	-0.784281
C	3.274244	-4.844370	-1.345089
C	2.775919	-5.904249	-0.544849
H	2.945859	-5.935398	0.523513
C	2.997128	-4.930917	-2.733097
H	3.330243	-4.170450	-3.427187
C	2.268449	-6.025367	-3.240110
H	2.051879	-6.085521	-4.297346
C	2.058080	-6.960156	-1.140610
H	1.679254	-7.767320	-0.530376
C	-3.939550	-0.174699	-6.035734
H	-3.415836	-0.227727	-6.979742
C	-3.781567	-1.203018	-5.088205
H	-3.131764	-2.032005	-5.337399

C	-5.367802	0.991747	-4.650026
H	-5.984789	1.865512	-4.496283
C	-5.264787	0.015175	-3.638484
H	-5.822489	0.157188	-2.721958
C	-4.459305	-1.133153	-3.844456
C	-4.335585	-2.162571	-2.863101
C	-4.799318	-2.997256	-0.804093
C	-3.465010	-4.159437	-2.221862
C	-2.640190	-5.287000	-2.513950
C	-2.374028	-6.301695	-1.561834
H	-2.820313	-6.280658	-0.576666
C	-2.024971	-5.450015	-3.780170
H	-2.169766	-4.738225	-4.582389
C	-1.180563	-6.548679	-4.017595
H	-0.704213	-6.653652	-4.981742
C	-1.517177	-7.371114	-1.896529
H	-1.319002	-8.146938	-1.171220
C	-5.474680	-2.887019	0.449777
C	-6.333279	-1.797727	0.747433
H	-6.508343	-1.001694	0.035161
C	-7.011528	-1.750874	1.981799
H	-7.677486	-0.927893	2.201355
C	-6.057350	-3.736037	2.671636
H	-5.959506	-4.488421	3.441339
C	-5.345280	-3.870898	1.463090
H	-4.724553	-4.747061	1.327632
C	0.047199	5.952974	4.008028
H	0.993973	6.467707	3.927981
C	-2.226971	5.969507	3.631682
H	-3.091048	6.499288	3.257355
C	-2.373158	4.668749	4.156946
H	-3.361441	4.229014	4.188715
C	-1.238891	3.969432	4.640460
C	-0.001396	4.655453	4.550352
H	0.925187	4.207591	4.884950
C	-1.326380	2.647245	5.171900
C	-2.508518	0.751126	5.562223
C	-0.279304	0.797543	5.969352
N	-6.218969	-1.396357	5.360531
C	-3.741767	0.033952	5.497328
C	-4.947296	0.653721	5.082923
H	-4.985287	1.702214	4.816568
C	-6.140774	-0.091332	5.028432
H	-7.057008	0.389491	4.716379
C	-3.838111	-1.337506	5.843308
H	-2.979378	-1.895227	6.192869
C	-5.079676	-1.999871	5.758959
H	-5.151954	-3.042757	6.033618
C	0.933145	0.150842	6.358813
C	0.976783	-1.212735	6.744397
H	0.080388	-1.818668	6.774222
C	2.202385	-1.796485	7.128484
H	2.233212	-2.832918	7.432625
C	3.341864	0.179428	6.794728
H	4.278769	0.716630	6.835165
C	2.170225	0.844080	6.387865
H	2.242836	1.891764	6.123972
C	-2.299444	2.991138	-6.950910
H	-2.153649	2.322705	-7.787865
C	-3.760397	4.182311	-5.628614
H	-4.779673	4.466377	-5.410172
C	-2.710171	4.698563	-4.846298
H	-2.958638	5.381072	-4.044018
C	-1.183863	3.456882	-6.226218
H	-0.197413	3.135107	-6.533741
C	-1.369008	4.337726	-5.131845
C	-0.274676	4.829199	-4.358053
C	0.466904	5.944753	-2.524148
C	1.943168	4.894618	-3.890234
C	0.168866	6.635790	-1.311145
C	-1.154796	7.015262	-0.969352
H	-1.990638	6.823549	-1.629279
C	-1.407906	7.668006	0.252402
H	-2.416901	7.956226	0.510185
C	0.822907	7.611747	0.834571
H	1.588455	7.850213	1.558532
C	1.171844	6.958012	-0.363771
H	2.210636	6.706616	-0.531093
C	3.286044	4.533326	-4.213748
C	4.386482	4.944048	-3.420999
H	4.257617	5.583025	-2.556639
C	5.688977	4.525494	-3.754880
H	6.523537	4.841142	-3.144733
C	3.599988	3.732187	-5.339975
H	2.834716	3.390631	-6.025430
C	4.936698	3.364702	-5.591190
H	5.169253	2.751249	-6.450149
C	9.180060	0.128874	-5.461645

H	8.150856	0.148038	-5.830244
H	9.814874	-0.126131	-6.319428
H	9.264397	-0.707784	-4.756941
C	9.958609	1.268986	-3.437396
H	9.329583	1.870851	-2.776031
H	9.898361	0.228491	-3.094660
H	10.993360	1.589711	-3.262874
C	10.573477	2.139640	-5.645779
H	11.456701	2.354650	-5.030706
H	10.955338	1.490083	-6.443834
C	10.176064	3.472919	-6.325553
H	10.905643	4.221415	-5.990830
H	10.391200	3.350450	-7.394858
C	8.776009	5.294145	-5.464276
H	8.330603	5.222265	-4.468337
H	9.781337	5.712765	-5.330256
H	8.204716	6.043591	-6.026211
C	8.032473	3.995111	-7.403497
H	7.611917	4.983853	-7.625941
H	8.648133	3.720305	-8.269113
H	7.200754	3.285292	-7.371121
N	-9.566776	-3.685124	4.152619
N	-8.865796	-2.364977	6.656679
C	-10.146573	-3.002841	2.965034
H	-10.075108	-3.603372	2.049624
H	-11.208308	-2.753801	3.085722
H	-9.622778	-2.060397	2.782264
C	-9.073731	-5.042124	3.797108
H	-9.181167	-5.270090	2.729221
H	-8.013506	-5.131335	4.048507
H	-9.592156	-5.844810	4.336333
C	-10.560546	-3.754545	5.263703
H	-10.796157	-4.797441	5.511956
H	-11.524286	-3.328765	4.956160
C	-10.181579	-3.066527	6.597969
H	-11.003176	-2.378558	6.835362
H	-10.247606	-3.838914	7.374923
C	-9.050419	-0.913892	6.926520
H	-8.477745	-0.564262	7.794687
H	-8.725784	-0.328307	6.062283
H	-10.095472	-0.639474	7.117746
C	-7.975973	-2.984932	7.674155
H	-7.608275	-2.265023	8.416057
H	-8.461917	-3.789136	8.240746
H	-7.102716	-3.427133	7.185673
N	-0.245086	-10.344527	-4.127974
N	2.565894	-9.870100	-3.509508
N	9.523524	1.439434	-4.849296
N	8.779940	3.958235	-6.118422
N	-1.409659	9.329435	5.038067
N	-0.798655	10.717099	2.544370
N	5.173991	-1.572606	0.303960
N	4.130544	-3.726827	0.561254
N	4.437602	-2.794734	-1.635907
N	1.810280	-7.029122	-2.464324
N	5.141823	-2.326423	5.299000
N	-4.725433	0.901248	-5.833131
N	-3.567523	-3.222975	-3.189093
N	-4.976813	-1.985737	-1.685650
N	-4.013636	-4.084949	-0.986953
N	-6.884287	-2.702368	2.928891
N	-0.918136	-7.500860	-3.099979
N	-1.040910	6.609781	3.560927
N	-2.535968	2.045514	5.164924
N	-1.410455	0.056111	5.949525
N	-0.170575	2.085727	5.583990
N	3.368266	-1.116694	7.163038
N	-3.571363	3.343195	-6.667249
N	-0.582454	5.621736	-3.309805
N	1.755281	5.603644	-2.753562
N	0.966042	4.405224	-4.687601
N	5.975281	3.742478	-4.816181
N	-0.441301	7.967811	1.142865
Pd	5.202973	-2.117276	7.392333
Pd	-0.928496	8.653499	3.065913
Pd	0.806144	-8.682617	-3.299690
Pd	7.743075	2.616427	-4.825409
Pd	-5.173496	2.219636	-7.416190
Pd	-7.878260	-2.538199	4.774449
C	1.160847	-3.355415	3.089872
C	0.912561	-1.881808	2.906339
C	1.544372	-1.322985	1.653113
C	1.266850	-2.136352	0.516890
C	0.422326	-3.350153	0.817221
C	0.912267	-4.104533	2.026512
H	1.359230	-3.772594	4.083009
H	1.059444	-1.272571	3.798658
C	2.095497	-0.056040	1.502746

C	1.503447	-1.691533	-0.753361
H	0.227082	-3.957462	-0.068151
H	0.900207	-5.196037	2.058680
C	2.013660	-0.383480	-0.955941
C	2.301645	0.452613	0.172611
H	1.241832	-2.299559	-1.616333
C	-1.471275	-1.651453	0.269237
C	-1.080980	-2.659962	1.324595
C	-0.824236	-1.814067	2.563978
C	-1.102662	-0.396498	2.155254
H	-1.858536	-3.475916	1.413993
H	-1.365809	-2.086093	3.504550
C	-1.394629	0.820917	-0.128519
C	-2.814388	1.020834	-0.691686
C	-0.860114	2.119263	0.486578
H	-0.725198	0.544646	-0.948444
C	-2.819761	2.144173	-1.736297
H	-3.481013	1.273704	0.140508
H	-3.174361	0.088475	-1.130483
C	-0.855607	3.227739	-0.574306
H	-1.501810	2.420645	1.321314
H	0.142641	1.980485	0.884082
C	-2.263349	3.446323	-1.146219
H	-3.841532	2.296024	-2.098524
H	-2.215612	1.821657	-2.596037
H	-0.456875	4.136618	-0.113005
H	-0.157569	2.953873	-1.378983
H	-2.271901	4.236093	-1.900964
H	-2.929384	3.783540	-0.342506
N	-1.362311	-0.362370	0.781752
O	-0.982683	0.583497	2.880779
O	-1.704599	-1.896953	-0.909085
C	2.718715	1.783705	-0.077176
C	2.198636	0.119552	-2.275626
C	2.615042	1.417669	-2.476082
H	2.746863	1.791578	-3.485232
C	2.859632	2.255332	-1.366865
H	3.145749	3.290069	-1.511333
H	2.852795	2.479134	0.738883
H	2.015232	-0.546212	-3.114451
C	2.447448	0.809475	2.693103
H	3.416205	1.286554	2.519971
H	2.567674	0.187120	3.587787
O	1.530504	1.874717	2.944701
H	0.633506	1.499422	3.019995
O	-0.072228	-3.278200	-2.935671
H	-0.237674	-2.512066	-3.512375
H	-0.745573	-3.146382	-2.251200
O	1.748801	4.423714	1.714919
H	2.661434	4.666917	1.913964
H	1.622458	3.587253	2.200172
O	-1.036843	-0.527816	-3.335499
H	-1.298224	-0.831548	-2.447814
H	-0.225701	-0.019066	-3.196409

Table5_1e_TSiii_reactant_1wat

Property	Value
Charge	0
Electronic Energy, BS1 (a.u.)	-1477.807226
Thermal and entropic correction, BS1 (a.u.)	2.820680
Electronic Energy, BS2 (a.u.)	-1478.342605
Number of Imaginary Frequencies	0
Imaginary frequencies (cm-1)	None

Molecular Geometry in Cartesian Coordinates

C	-1.109959	-10.862139	-3.131281
H	-0.993562	-11.914160	-2.841896
H	-1.014660	-10.269922	-2.216711
H	-2.142909	-10.754185	-3.485424
C	-0.770098	-9.940855	-5.375439
H	-1.865282	-9.969930	-5.313626
H	-0.479140	-8.907864	-5.582303
H	-0.496549	-10.525714	-6.262498
C	0.869082	-11.504503	-4.435209
H	0.795962	-11.810822	-5.486700
H	0.627566	-12.416302	-3.873986
C	2.366202	-11.208009	-4.176322
H	2.877960	-11.305763	-5.142246
H	2.745773	-12.052141	-3.586349
C	3.631411	-9.113626	-4.364325
H	3.137366	-8.197656	-4.702171
H	4.544424	-8.817801	-3.832728

H	3.960137	-9.646234	-5.265694
C	3.276159	-10.137697	-2.166855
H	2.591273	-9.761844	-1.403422
H	3.441421	-11.198609	-1.940962
H	4.242585	-9.638630	-2.022254
N	7.068695	-3.144685	7.472979
N	5.565776	-1.831618	9.601168
C	6.719500	-4.515207	7.015885
H	5.635357	-4.606338	6.909064
H	7.172422	-4.772212	6.050260
H	7.031585	-5.297029	7.719658
C	7.995793	-2.483632	6.515780
H	7.566629	-1.541163	6.164865
H	8.969465	-2.238415	6.958457
H	8.207889	-3.099133	5.632690
C	7.656334	-3.174150	8.844477
H	7.822903	-4.207583	9.174658
H	8.658311	-2.726311	8.853227
C	6.855642	-2.482293	9.974829
H	7.538048	-1.761386	10.443143
H	6.697388	-3.242680	10.750438
C	5.591816	-0.379727	9.923904
H	4.755232	-0.068866	10.562237
H	6.505969	-0.069505	10.445524
H	5.539277	0.210337	9.004945
C	4.417104	-2.502429	10.265190
H	3.772945	-2.967416	9.513591
H	4.722568	-3.298519	10.955666
H	3.796303	-1.810592	10.848334
N	-6.774974	1.248976	-8.496130
N	-5.178883	3.671782	-8.822379
C	-6.345675	-0.085302	-8.993417
H	-6.912320	-0.911053	-8.545364
H	-6.450399	-0.195321	-10.080005
H	-5.289247	-0.247955	-8.764473
C	-7.912389	1.122181	-7.546803
H	-8.210340	0.080414	-7.374264
H	-7.639305	1.549775	-6.577606
H	-8.815088	1.648045	-7.882187
C	-7.123724	2.151732	-9.632064
H	-8.160632	2.502231	-9.551290
H	-7.099555	1.606587	-10.584300
C	-6.245599	3.411608	-9.833328
H	-5.821436	3.340215	-10.843082
H	-6.938886	4.260302	-9.894369
C	-5.453134	4.928216	-8.075756
H	-4.608196	5.628017	-8.091290
H	-6.317587	5.481149	-8.464116
H	-5.673922	4.700525	-7.029820
C	-3.837353	3.715744	-9.462622
H	-3.294753	4.646407	-9.254423
H	-3.221942	2.888773	-9.096698
H	-3.876853	3.621321	-10.555147
C	-0.204010	9.342974	5.972142
H	-0.573471	8.671480	6.757244
H	0.583382	8.813832	5.428829
H	0.280901	10.181372	6.487596
C	-2.593492	9.233928	5.439597
H	-2.536757	8.592812	6.328235
H	-3.326118	10.017058	5.671713
H	-3.020346	8.636355	4.629334
C	-1.312605	11.261628	4.919137
H	-0.613735	11.726046	5.626647
H	-2.294371	11.649384	5.219664
C	-1.001631	11.879186	3.533364
H	-1.850898	12.529769	3.288054
H	-0.167285	12.576012	3.684647
C	-1.723073	11.075079	1.329472
H	-1.274787	11.271582	0.347436
H	-2.307455	10.154934	1.244959
H	-2.439150	11.886873	1.508088
C	0.662505	11.147300	1.882718
H	0.680339	11.342346	0.803153
H	1.183944	11.988791	2.355711
H	1.271738	10.257257	2.064911
N	6.974178	1.092426	-3.459207
C	7.017447	1.204885	-2.115905
H	7.579649	2.035298	-1.712115
C	6.383694	0.296514	-1.245182
H	6.487581	0.449964	-0.178816
C	5.656755	-0.801230	-1.771735
C	5.609660	-0.895043	-3.185718
H	5.089354	-1.703208	-3.683756
C	6.274014	0.062493	-3.977679
H	6.243458	-0.016037	-5.055199
C	5.035820	-1.771016	-0.927716
C	4.640640	-2.595169	1.150129
C	4.733514	-2.451227	2.569364

C	5.336661	-1.315009	3.166758
H	5.761090	-0.517981	2.570193
C	5.417433	-1.211133	4.568819
H	5.885199	-0.346925	5.019868
C	4.359321	-3.240235	4.859513
H	3.977528	-3.986870	5.540894
C	4.239164	-3.431853	3.466289
H	3.777449	-4.341570	3.104000
C	3.936522	-3.739052	-0.681202
C	3.267803	-4.858253	-1.264609
C	2.861427	-5.973605	-0.492036
H	3.060091	-6.025631	0.570945
C	2.968735	-4.921324	-2.650117
H	3.237262	-4.119733	-3.325917
C	2.296072	-6.042093	-3.177870
H	2.061429	-6.083530	-4.232502
C	2.197556	-7.053725	-1.106851
H	1.885179	-7.898694	-0.510994
C	-4.287654	-0.570793	-6.212772
H	-3.790544	-0.640049	-7.169580
C	-4.111767	-1.590747	-5.258516
H	-3.479541	-2.430656	-5.516225
C	-5.677256	0.618787	-4.804979
H	-6.293391	1.492764	-4.649337
C	-5.552266	-0.346480	-3.786033
H	-6.090288	-0.191636	-2.859909
C	-4.757055	-1.501330	-3.999212
C	-4.607333	-2.513197	-3.004101
C	-4.948490	-3.251630	-0.885717
C	-3.652645	-4.453890	-2.309977
C	-2.737166	-5.522160	-2.558417
C	-2.373201	-6.465169	-1.564182
H	-2.817914	-6.441908	-0.578074
C	-2.112724	-5.684176	-3.820417
H	-2.321449	-5.023256	-4.651912
C	-1.166835	-6.705238	-4.016358
H	-0.681636	-6.800732	-4.976868
C	-1.422440	-7.466784	-1.858814
H	-1.150570	-8.190754	-1.104253
C	-5.492276	-3.032676	0.417102
C	-6.275671	-1.888575	0.717291
H	-6.499083	-1.137953	-0.030013
C	-6.805753	-1.715940	2.010067
H	-7.411300	-0.848950	2.234444
C	-5.854977	-3.683828	2.750698
H	-5.698827	-4.377446	3.564533
C	-5.285070	-3.940855	1.486303
H	-4.710291	-4.847714	1.352383
C	0.189907	6.337443	4.329787
H	1.106175	6.909694	4.354677
C	-2.042405	6.234947	3.752305
H	-2.902577	6.729376	3.323962
C	-2.155588	4.914584	4.230609
H	-3.117053	4.422503	4.163649
C	-1.029908	4.268481	4.802524
C	0.169270	5.024019	4.838483
H	1.080989	4.617495	5.255845
C	-1.100617	2.937242	5.314783
C	-2.294792	1.046349	5.708923
C	-0.105420	1.150238	6.303619
N	-5.950523	-1.170229	5.376753
C	-3.511667	0.307703	5.597192
C	-4.679872	0.867334	5.021424
H	-4.697823	1.885495	4.655282
C	-5.859181	0.101528	4.936140
H	-6.752675	0.535745	4.509985
C	-3.624097	-1.029369	6.054355
H	-2.793524	-1.537655	6.525424
C	-4.844882	-1.719540	5.919815
H	-4.928307	-2.736400	6.274083
C	1.082779	0.490627	6.745540
C	1.077026	-0.856653	7.187076
H	0.157504	-1.422208	7.261016
C	2.286016	-1.487224	7.543001
H	2.276367	-2.513734	7.879396
C	3.502647	0.416460	7.101954
H	4.464670	0.908974	7.084397
C	2.347666	1.129043	6.724011
H	2.454941	2.162239	6.420065
C	-2.278779	2.495896	-6.521655
H	-2.118746	1.794190	-7.327928
C	-3.769300	3.750919	-5.284588
H	-4.793054	4.045306	-5.104077
C	-2.736607	4.281298	-4.487239
H	-2.997802	4.994328	-3.716612
C	-1.184056	2.967238	-5.771414
H	-0.195095	2.612075	-6.029504
C	-1.392498	3.892294	-4.716864

C	-0.312200	4.401973	-3.934874
C	0.435444	5.627994	-2.174249
C	1.909067	4.504604	-3.484255
C	0.165355	6.441097	-1.031615
C	-1.149501	6.850207	-0.693707
H	-2.003331	6.575374	-1.298764
C	-1.367051	7.652297	0.443616
H	-2.369305	7.968011	0.694730
C	0.881515	7.690190	0.949073
H	1.670171	8.035711	1.602400
C	1.195808	6.888130	-0.165929
H	2.234638	6.641736	-0.343877
C	3.253464	4.168920	-3.826451
C	4.358916	4.619299	-3.062958
H	4.228153	5.246500	-2.190497
C	5.667939	4.261233	-3.440582
H	6.506277	4.607980	-2.853309
C	3.564516	3.377660	-4.960347
H	2.793948	3.009031	-5.624862
C	4.906814	3.076719	-5.262063
H	5.139286	2.480253	-6.133018
C	9.431060	0.252086	-5.516519
H	8.375661	0.170657	-5.790800
H	10.008239	0.083504	-6.434240
H	9.663942	-0.587064	-4.849175
C	10.252771	1.419098	-3.525814
H	9.607407	1.919790	-2.798609
H	10.342017	0.369464	-3.219233
H	11.252511	1.857060	-3.414395
C	10.602028	2.396191	-5.750146
H	11.521427	2.655112	-5.209493
H	10.954066	1.816444	-6.613065
C	10.039484	3.723023	-6.317488
H	10.739665	4.511064	-6.011956
H	10.157434	3.669196	-7.407338
C	8.603553	5.358559	-5.183616
H	8.250949	5.170904	-4.165659
H	9.587251	5.835929	-5.092752
H	7.938241	6.108679	-5.629273
C	7.778038	4.172174	-7.161374
H	7.267652	5.139099	-7.254494
H	8.332155	4.026447	-8.097064
H	7.007040	3.397184	-7.128476
N	-9.256288	-3.595256	4.317395
N	-8.581406	-2.110364	6.731864
C	-9.814426	-3.055801	3.048485
H	-9.687442	-3.741004	2.201184
H	-10.888511	-2.839008	3.107604
H	-9.320911	-2.115745	2.788868
C	-8.737971	-4.975423	4.126805
H	-8.827998	-5.327356	3.091430
H	-7.679719	-5.015536	4.399842
H	-9.250254	-5.718536	4.750788
C	-10.280023	-3.555054	5.402325
H	-10.540848	-4.570127	5.728431
H	-11.227719	-3.139872	5.036028
C	-9.920390	-2.767400	6.685804
H	-10.723903	-2.035895	6.839845
H	-10.036164	-3.471147	7.520111
C	-8.718961	-0.646789	6.961367
H	-8.138705	-0.293828	7.823050
H	-8.372104	-0.092816	6.084735
H	-9.754999	-0.333450	7.141727
C	-7.718039	-2.732166	7.770124
H	-7.327244	-2.005415	8.493402
H	-8.235120	-3.502487	8.355706
H	-6.860991	-3.220337	7.297435
N	-0.105382	-10.415862	-4.132854
N	2.702510	-9.910989	-3.519599
N	9.693533	1.577353	-4.894634
N	8.638717	4.085148	-5.951324
N	-1.270071	9.774174	5.029466
N	-0.711628	10.938200	2.411394
N	5.145387	-1.586519	0.405837
N	4.034281	-3.706006	0.670139
N	4.379780	-2.785859	-1.530901
N	1.918123	-7.095978	-2.425617
N	4.942974	-2.153166	5.407829
N	-5.062972	0.511897	-6.000846
N	-3.837459	-3.575730	-3.320302
N	-5.193065	-2.293382	-1.806976
N	-4.167339	-4.345101	-1.061229
N	-6.609551	-2.596150	3.011866
N	-0.819483	-7.590179	-3.061171
N	-0.894808	6.941474	3.803067
N	-2.288233	2.300980	5.205945
N	-1.237803	0.410191	6.264173
N	0.029676	2.414864	5.843502

N	3.486369	-0.872259	7.502603
N	-3.550081	2.880529	-6.291126
N	-0.624161	5.235792	-2.917549
N	1.723116	5.278877	-2.392276
N	0.930150	3.972463	-4.249181
N	5.950488	3.504691	-4.521983
N	-0.374904	8.074228	1.254041
Pd	5.260939	-2.004203	7.492829
Pd	-0.810254	8.929480	3.120634
Pd	0.926616	-8.749795	-3.281817
Pd	7.816929	2.567941	-4.698383
Pd	-5.139412	2.079305	-7.401989
Pd	-7.596071	-2.364886	4.856938
C	0.872987	-5.803518	3.794403
C	1.010608	-4.437085	3.782167
C	0.859195	-3.685175	2.578924
C	0.551789	-4.403591	1.373614
C	0.310543	-5.805926	1.451177
C	0.497914	-6.497669	2.620043
H	1.022191	-6.359561	4.716067
H	1.268600	-3.927395	4.705854
C	0.987799	-2.266197	2.516198
C	0.478905	-3.697771	0.156873
H	0.007913	-6.326340	0.540975
H	0.348350	-7.570775	2.661905
C	0.955895	-2.380797	0.062292
C	1.237574	-1.647557	1.266273
H	0.117773	-4.196236	-0.748191
C	-1.877999	-1.893583	-0.175032
C	-1.823202	-2.946751	0.871017
C	-1.694936	-2.369835	2.051233
C	-1.815018	-0.905983	1.866564
H	-1.978137	-4.026042	0.612060
H	-1.700290	-2.810945	3.044648
C	-1.877926	0.599089	-0.244431
C	-3.280632	0.878755	-0.797042
C	-1.335206	1.774180	0.576307
H	-1.203727	0.446828	-1.094120
C	-3.251332	2.141539	-1.667401
H	-3.973014	1.011933	0.042684
H	-3.625025	0.018502	-1.377580
C	-1.307218	3.028604	-0.306208
H	-1.975793	1.954052	1.438383
H	-0.337446	1.555394	0.959403
C	-2.700916	3.333516	-0.871619
H	-4.256636	2.362895	-2.040448
H	-2.614963	1.937310	-2.540248
H	-0.924246	3.866388	0.286637
H	-0.597894	2.875163	-1.133547
H	-2.682068	4.231965	-1.494147
H	-3.384995	3.548366	-0.041668
N	-1.856772	-0.673305	0.486048
O	-1.937499	-0.055291	2.743312
O	-1.961975	-2.013723	-1.399920
C	1.804993	-0.341005	1.129759
C	1.198539	-1.782933	-1.211222
C	1.773890	-0.542652	-1.290228
H	2.013890	-0.116283	-2.258317
C	2.083889	0.183582	-0.106929
H	2.538394	1.165075	-0.177962
H	2.026176	0.249708	2.005442
H	0.956038	-2.348595	-2.106733
C	1.023822	-1.473980	3.815998
H	2.053186	-1.434497	4.194142
H	0.427441	-2.005125	4.571839
O	0.588226	-0.129031	3.720097
H	-0.369180	-0.114214	3.542177
O	-0.395723	-3.229774	-3.500402
H	-0.500471	-2.471832	-4.091515
H	-1.081312	-3.062069	-2.835375
O	1.052262	2.512130	2.650211
H	1.942784	2.851910	2.798042
H	1.008782	1.698961	3.173355
O	-1.295239	-0.301682	-3.579625
H	-1.629360	-0.777903	-2.797171
H	-0.371683	-0.135683	-3.351268

Table5_1e_TSiii_TSi-ii_h2o_2

Property	Value
Charge	0
Electronic Energy, BS1 (a.u.)	-1477.762572
Thermal and entropic correction, BS1 (a.u.)	2.821968

Electronic Energy, BS2 (a.u.)	-1478.296498
Number of Imaginary Frequencies	0
Imaginary frequencies (cm-1)	None

Molecular Geometry in Cartesian Coordinates

C	-1.103449	-10.867037	-3.120494
H	-0.980141	-11.918146	-2.830616
H	-1.005795	-10.273416	-2.207056
H	-2.138874	-10.764322	-3.468963
C	-0.780380	-9.940900	-5.365188
H	-1.875072	-9.971008	-5.295543
H	-0.491877	-8.907467	-5.573094
H	-0.512884	-10.524591	-6.254872
C	0.868153	-11.502845	-4.439113
H	0.795792	-11.800384	-5.493156
H	0.625861	-12.419279	-3.885824
C	2.365136	-11.209320	-4.176073
H	2.879073	-11.306251	-5.140953
H	2.741958	-12.055119	-3.586728
C	3.634462	-9.117017	-4.357580
H	3.142686	-8.200062	-4.696009
H	4.546396	-8.822980	-3.823155
H	3.964951	-9.649165	-5.258551
C	3.272154	-10.144056	-2.162562
H	2.587448	-9.765622	-1.400208
H	3.432300	-11.205801	-1.936960
H	4.240473	-9.649285	-2.015811
N	7.067562	-3.145042	7.472824
N	5.566426	-1.832205	9.602330
C	6.716810	-4.515433	7.016490
H	5.632544	-4.605552	6.910047
H	7.169144	-4.773329	6.050828
H	7.028364	-5.297258	7.720493
C	7.994331	-2.485106	6.514549
H	7.565750	-1.542310	6.163853
H	8.968679	-2.240750	6.956204
H	8.204946	-3.100993	5.631373
C	7.656396	-3.174576	8.843825
H	7.821363	-4.208045	9.174684
H	8.659211	-2.728591	8.851292
C	6.858102	-2.480182	9.974344
H	7.540925	-1.757172	10.438786
H	6.702685	-3.238508	10.752553
C	5.589483	-0.380580	9.926573
H	4.752135	-0.072127	10.565073
H	6.502899	-0.069097	10.448748
H	5.535932	0.210460	9.008305
C	4.419774	-2.506332	10.266434
H	3.775506	-2.970903	9.514644
H	4.727566	-3.303355	10.954809
H	3.798501	-1.816766	10.851771
N	-6.779351	1.251136	-8.498272
N	-5.182063	3.673484	-8.822364
C	-6.350431	-0.082943	-8.996439
H	-6.917816	-0.908784	-8.549475
H	-6.454542	-0.191985	-10.083184
H	-5.294249	-0.246383	-8.766932
C	-7.917043	1.124024	-7.549325
H	-8.215268	0.082225	-7.377447
H	-7.644077	1.550970	-6.579808
H	-8.819523	1.650325	-7.884619
C	-7.127434	2.154885	-9.633625
H	-8.164494	2.505037	-9.553268
H	-7.102434	1.610661	-10.586363
C	-6.249558	3.415249	-9.832997
H	-5.826110	3.345872	-10.843195
H	-6.942955	4.264002	-9.891884
C	-5.454677	4.929647	-8.074695
H	-4.608819	5.628338	-8.089649
H	-6.318380	5.484073	-8.462596
H	-5.675779	4.701352	-7.028957
C	-3.840764	3.716494	-9.463145
H	-3.297168	4.646516	-9.254674
H	-3.226021	2.888701	-9.097953
H	-3.880825	3.622716	-10.555705
C	-0.203093	9.335268	5.969233
H	-0.571397	8.662451	6.753749
H	0.584803	8.807821	5.425033
H	0.280991	10.173731	6.485371
C	-2.592227	9.221650	5.435822
H	-2.533821	8.576735	6.321596
H	-3.326157	10.002423	5.671766
H	-3.018395	8.626874	4.623145
C	-1.316332	11.253675	4.920684
H	-0.622064	11.718101	5.632709
H	-2.300555	11.637992	5.217577
C	-1.000210	11.875627	3.538065

H	-1.847437	12.528945	3.292973
H	-0.164746	12.570069	3.694032
C	-1.720591	11.078114	1.331422
H	-1.272001	11.278343	0.350268
H	-2.304325	10.157891	1.243491
H	-2.437308	11.888776	1.512594
C	0.664718	11.149055	1.885898
H	0.683003	11.346782	0.806822
H	1.185558	11.989612	2.361198
H	1.274189	10.258758	2.066174
N	6.974271	1.088935	-3.460794
C	7.017394	1.203451	-2.117591
H	7.579525	2.034359	-1.714749
C	6.383516	0.296146	-1.245696
H	6.487136	0.450865	-0.179476
C	5.656643	-0.802307	-1.770939
C	5.609786	-0.898099	-3.184761
H	5.089626	-1.706848	-3.681998
C	6.274265	0.058241	-3.977793
H	6.243874	-0.021762	-5.055223
C	5.035351	-1.771150	-0.926163
C	4.638759	-2.593887	1.151883
C	4.731239	-2.449478	2.571071
C	5.335138	-1.313553	3.168245
H	5.760095	-0.516932	2.571517
C	5.416130	-1.209596	4.570280
H	5.884544	-0.345667	5.021200
C	4.356376	-3.237804	4.861299
H	3.973973	-3.983977	5.542843
C	4.235929	-3.429490	3.468140
H	3.773319	-4.338836	3.106060
C	3.935632	-3.738868	-0.679087
C	3.267852	-4.858525	-1.262633
C	2.861119	-5.973711	-0.490037
H	3.058534	-6.025111	0.573211
C	2.970542	-4.922332	-2.648500
H	3.239483	-4.120911	-3.324352
C	2.299438	-6.043792	-3.176675
H	2.066373	-6.085937	-4.231642
C	2.199011	-7.054653	-1.105319
H	1.886768	-7.899728	-0.509547
C	-4.291520	-0.570249	-6.215116
H	-3.795314	-0.639529	-7.172388
C	-4.113118	-1.589213	-5.260282
H	-3.479970	-2.428408	-5.518043
C	-5.681678	0.618185	-4.806986
H	-6.299087	1.491274	-4.651416
C	-5.554303	-0.346167	-3.787466
H	-6.091803	-0.191494	-2.861015
C	-4.757536	-1.499967	-4.000534
C	-4.605807	-2.511170	-3.005056
C	-4.946614	-3.250120	-0.886830
C	-3.649306	-4.450989	-2.310964
C	-2.733366	-5.518948	-2.559055
C	-2.372049	-6.463708	-1.565491
H	-2.818787	-6.441788	-0.580268
C	-2.106183	-5.679249	-3.819909
H	-2.312609	-5.016762	-4.650748
C	-1.160802	-6.700882	-4.015513
H	-0.673649	-6.795418	-4.975160
C	-1.421744	-7.465812	-1.859782
H	-1.152175	-8.191282	-1.105878
C	-5.489686	-3.031453	0.416327
C	-6.274583	-1.888473	0.716794
H	-6.499999	-1.138627	-0.030692
C	-6.803484	-1.716060	2.010107
H	-7.410138	-0.849908	2.234778
C	-5.848443	-3.681836	2.750726
H	-5.689807	-4.374506	3.564904
C	-5.279828	-3.938737	1.485760
H	-4.703561	-4.844622	1.351725
C	0.192288	6.334083	4.323048
H	1.108685	6.906102	4.347746
C	-2.038952	6.230536	3.741866
H	-2.898076	6.723931	3.310279
C	-2.153343	4.911511	4.223640
H	-3.114672	4.419257	4.156114
C	-1.028740	4.266620	4.799005
C	0.170492	5.022044	4.835138
H	1.081525	4.616296	5.254747
C	-1.100198	2.936423	5.313840
C	-2.294381	1.045869	5.709573
C	-0.105266	1.150517	6.304853
N	-5.949805	-1.171607	5.379208
C	-3.511098	0.306905	5.598361
C	-4.679590	0.866128	5.022851
H	-4.697870	1.884237	4.656621
C	-5.858775	0.100071	4.938256

H	-6.752525	0.534084	4.512435
C	-3.623078	-1.030099	6.055840
H	-2.792157	-1.538217	6.526510
C	-4.843792	-1.720527	5.921958
H	-4.926802	-2.737331	6.276520
C	1.082995	0.491324	6.747095
C	1.077242	-0.855439	7.190146
H	0.157708	-1.420852	7.264978
C	2.286248	-1.485580	7.546692
H	2.276596	-2.511652	7.884394
C	3.502903	0.417436	7.102935
H	4.464983	0.909820	7.084517
C	2.347900	1.129663	6.724442
H	2.455173	2.162455	6.419105
C	-2.282685	2.494522	-6.523337
H	-2.123385	1.792321	-7.329329
C	-3.772186	3.749670	-5.285093
H	-4.795845	4.043744	-5.103513
C	-2.738736	4.280779	-4.489207
H	-2.999249	4.994113	-3.718607
C	-1.187275	2.966408	-5.774476
H	-0.198525	2.611249	-6.033381
C	-1.394799	3.891835	-4.720084
C	-0.313778	4.401671	-3.939223
C	0.435208	5.628435	-2.179681
C	1.907664	4.502916	-3.489215
C	0.165899	6.442885	-1.037812
C	-1.148556	6.853478	-0.700162
H	-2.002584	6.579299	-1.305272
C	-1.365357	7.656236	0.436826
H	-2.367381	7.972870	0.687717
C	0.883219	7.692939	0.941853
H	1.672289	8.038398	1.594728
C	1.196773	6.889887	-0.172663
H	2.235355	6.642682	-0.350800
C	3.251722	4.165628	-3.831136
C	4.357395	4.615187	-3.067467
H	4.227145	5.242976	-2.195334
C	5.666107	4.255546	-3.444484
H	6.504541	4.601367	-2.856843
C	3.562222	3.373654	-4.964771
H	2.791463	3.005708	-5.629529
C	4.904378	3.071438	-5.266080
H	5.136857	2.474756	-6.136872
C	9.432607	0.247678	-5.515582
H	8.377616	0.164764	-5.790950
H	10.011169	0.077155	-6.432061
H	9.665228	-0.589551	-4.845740
C	10.252630	1.420477	-3.527609
H	9.606322	1.921355	-2.801373
H	10.344149	0.371670	-3.218860
H	11.251409	1.860845	-3.417127
C	10.600316	2.392874	-5.754357
H	11.522081	2.649001	-5.216433
H	10.948270	1.813900	-6.619444
C	10.038858	3.722136	-6.317030
H	10.740718	4.508185	-6.010199
H	10.155072	3.671312	-7.407204
C	8.607527	5.354318	-5.172267
H	8.255968	5.161324	-4.155011
H	9.592203	5.829422	-5.080161
H	7.942897	6.108158	-5.612612
C	7.777801	4.181340	-7.156311
H	7.268371	5.149393	-7.242685
H	8.331022	4.041164	-8.093399
H	7.005945	3.406885	-7.128049
N	-9.251656	-3.599375	4.313622
N	-8.580876	-2.116518	6.730735
C	-9.811956	-3.056348	3.047163
H	-9.687166	-3.739453	2.197842
H	-10.885722	-2.838741	3.108914
H	-9.318274	-2.115998	2.788982
C	-8.730522	-4.977715	4.117340
H	-8.817955	-5.324777	3.080107
H	-7.672700	-5.017255	4.392061
H	-9.242603	-5.724863	4.736643
C	-10.274458	-3.565754	5.399747
H	-10.527566	-4.582286	5.727327
H	-11.225625	-3.158140	5.033983
C	-9.919632	-2.773746	6.681912
H	-10.723823	-2.041948	6.830959
H	-10.038033	-3.474959	7.518010
C	-8.719263	-0.653331	6.962366
H	-8.140704	-0.301632	7.825716
H	-8.370954	-0.097723	6.087362
H	-9.755696	-0.340588	7.141462
C	-7.718622	-2.739617	7.769140
H	-7.329682	-2.014002	8.494552

H	-8.235970	-3.511663	8.352211
H	-6.860327	-3.226001	7.296873
N	-0.106491	-10.416840	-4.127846
N	2.702154	-9.914051	-3.516297
N	9.693111	1.574729	-4.896814
N	8.639291	4.085418	-5.947417
N	-1.270019	9.766092	5.027374
N	-0.709597	10.938113	2.413354
N	5.144438	-1.585838	0.407302
N	4.032355	-3.704905	0.672264
N	4.379470	-2.786256	-1.528977
N	1.921410	-7.097665	-2.424474
N	4.941036	-2.151203	5.409422
N	-5.068340	0.511397	-6.003349
N	-3.834007	-3.572450	-3.320978
N	-5.192021	-2.292033	-1.808012
N	-4.165099	-4.343240	-1.062629
N	-6.604542	-2.595309	3.012226
N	-0.816530	-7.587884	-3.061084
N	-0.891260	6.936922	3.792660
N	-2.287791	2.300021	5.205299
N	-1.237469	0.410263	6.265582
N	0.029649	2.414952	5.844344
N	3.486639	-0.870788	7.505180
N	-3.553823	2.878855	-6.291462
N	-0.624867	5.236110	-2.922180
N	1.722541	5.277985	-2.397613
N	0.928250	3.971594	-4.254059
N	5.948136	3.498562	-4.525686
N	-0.372886	8.077949	1.247019
Pd	5.260646	-2.003229	7.494104
Pd	-0.807854	8.927175	3.116443
Pd	0.927818	-8.750214	-3.280380
Pd	7.814760	2.562595	-4.703452
Pd	-5.143763	2.079954	-7.403117
Pd	-7.593354	-2.367623	4.856497
C	0.873168	-5.807094	3.797769
C	1.018988	-4.431757	3.780318
C	0.851444	-3.698598	2.580613
C	0.528314	-4.409252	1.393047
C	0.314389	-5.798501	1.449237
C	0.504760	-6.497983	2.627776
H	1.016760	-6.357969	4.720082
H	1.266372	-3.921186	4.704013
C	0.878221	-2.253126	2.508065
C	0.249749	-3.620668	0.215138
H	-0.004074	-6.315439	0.547728
H	0.355875	-7.571160	2.665844
C	0.937763	-2.360892	0.063117
C	1.231495	-1.635470	1.253660
H	0.003477	-4.150464	-0.702946
C	-1.851340	-1.889106	-0.188323
C	-1.609000	-2.978237	0.804465
C	-1.546868	-2.344854	2.064254
C	-1.802428	-0.920327	1.875896
H	-2.063051	-3.947403	0.645906
H	-1.712240	-2.812684	3.022043
C	-1.880700	0.599372	-0.238142
C	-3.283212	0.884609	-0.795190
C	-1.333501	1.773677	0.579375
H	-1.206368	0.443129	-1.087787
C	-3.248294	2.147486	-1.665330
H	-3.975440	1.018286	0.044567
H	-3.628427	0.024754	-1.375884
C	-1.304255	3.028475	-0.302508
H	-1.974809	1.953366	1.445372
H	-0.336185	1.554308	0.963393
C	-2.697226	3.338886	-0.869217
H	-4.253271	2.369718	-2.038734
H	-2.611789	1.942757	-2.537928
H	-0.921487	3.865912	0.290991
H	-0.594202	2.875165	-1.129252
H	-2.676082	4.236966	-1.492057
H	-3.381893	3.554987	-0.040062
N	-1.860244	-0.686782	0.486818
O	-1.947046	-0.057013	2.739943
O	-1.965593	-2.003774	-1.408923
C	1.812780	-0.346844	1.129399
C	1.202938	-1.794921	-1.202047
C	1.782353	-0.541354	-1.286267
H	2.018080	-0.119361	-2.258756
C	2.087740	0.181047	-0.116507
H	2.533926	1.166643	-0.184191
H	2.025351	0.244385	2.004658
H	0.943665	-2.353034	-2.097050
C	0.987051	-1.469652	3.808100
H	2.037064	-1.466256	4.133595
H	0.411248	-1.989274	4.583531

O	0.585201	-0.118319	3.723722
H	-0.376006	-0.088994	3.539296
O	-0.396982	-3.217009	-3.507001
H	-0.504345	-2.458212	-4.100887
H	-1.081556	-3.051995	-2.840327
O	1.057554	2.518225	2.656206
H	1.948600	2.860862	2.794069
H	1.022902	1.704445	3.183408
O	-1.298650	-0.290611	-3.577842
H	-1.636667	-0.768668	-2.800669
H	-0.374302	-0.127412	-3.350538

Table5_1e_TSiii_DG_h2o_2

Property	Value
Charge	0
Electronic Energy, BS1 (a.u.)	-1477.811419
Thermal and entropic correction, BS1 (a.u.)	2.828026
Electronic Energy, BS2 (a.u.)	-1478.343009
Number of Imaginary Frequencies	0
Imaginary frequencies (cm-1)	None

Molecular Geometry in Cartesian Coordinates

C	-1.103449	-10.867037	-3.120494
H	-0.980141	-11.918146	-2.830616
H	-1.005795	-10.273416	-2.207056
H	-2.138874	-10.764322	-3.468963
C	-0.780380	-9.940900	-5.365188
H	-1.875072	-9.971008	-5.295543
H	-0.491877	-8.907467	-5.573094
H	-0.512884	-10.524591	-6.254872
C	0.868153	-11.502845	-4.439113
H	0.795792	-11.800384	-5.493156
H	0.625861	-12.419279	-3.885824
C	2.365136	-11.209320	-4.176073
H	2.879073	-11.306251	-5.140953
H	2.741958	-12.055119	-3.586728
C	3.634462	-9.117017	-4.357580
H	3.142686	-8.200062	-4.696009
H	4.546396	-8.822980	-3.823155
H	3.964951	-9.649165	-5.258551
C	3.272154	-10.144056	-2.162562
H	2.587448	-9.765622	-1.400208
H	3.432300	-11.205801	-1.936960
H	4.240473	-9.649285	-2.015811
N	7.067562	-3.145042	7.472824
N	5.566426	-1.832205	9.602330
C	6.716810	-4.515433	7.016490
H	5.632544	-4.605552	6.910047
H	7.169144	-4.773329	6.050828
H	7.028364	-5.297258	7.720493
C	7.994331	-2.485106	6.514549
H	7.565750	-1.542310	6.163853
H	8.968679	-2.240750	6.956204
H	8.204946	-3.100993	5.631373
C	7.656396	-3.174576	8.843825
H	7.821363	-4.208045	9.174684
H	8.659211	-2.728591	8.851292
C	6.858102	-2.480182	9.974344
H	7.540925	-1.757172	10.438786
H	6.702685	-3.238508	10.752553
C	5.589483	-0.380580	9.926573
H	4.752135	-0.072127	10.565073
H	6.502899	-0.069097	10.448748
H	5.535932	0.210460	9.008305
C	4.419774	-2.506332	10.266434
H	3.775506	-2.970903	9.514644
H	4.727566	-3.303355	10.954809
H	3.798501	-1.816766	10.851771
N	-6.779351	1.251136	-8.498272
N	-5.182063	3.673484	-8.822364
C	-6.350431	-0.082943	-8.996439
H	-6.917816	-0.908784	-8.549475
H	-6.454542	-0.191985	-10.083184
H	-5.294249	-0.246383	-8.766932
C	-7.917043	1.124024	-7.549325
H	-8.215268	0.082225	-7.377447
H	-7.644077	1.550970	-6.579808
H	-8.819523	1.650325	-7.884619
C	-7.127434	2.154885	-9.633625
H	-8.164494	2.505037	-9.553268
H	-7.102434	1.610661	-10.586363
C	-6.249558	3.415249	-9.832997

H	-5.826110	3.345872	-10.843195
H	-6.942955	4.264002	-9.891884
C	-5.454677	4.929647	-8.074695
H	-4.608819	5.628338	-8.089649
H	-6.318380	5.484073	-8.462596
H	-5.675779	4.701352	-7.028957
C	-3.840764	3.716494	-9.463145
H	-3.297168	4.646516	-9.254674
H	-3.226021	2.888701	-9.097953
H	-3.880825	3.622716	-10.555705
C	-0.203093	9.335268	5.969233
H	-0.571397	8.662451	6.753749
H	0.584803	8.807821	5.425033
H	0.280991	10.173731	6.485371
C	-2.592227	9.221650	5.435822
H	-2.533821	8.576735	6.321596
H	-3.326157	10.002423	5.671766
H	-3.018395	8.626874	4.623145
C	-1.316332	11.253675	4.920684
H	-0.622064	11.718101	5.632709
H	-2.300555	11.637992	5.217577
C	-1.000210	11.875627	3.538065
H	-1.847437	12.528945	3.292973
H	-0.164746	12.570069	3.694032
C	-1.720591	11.078114	1.331422
H	-1.272001	11.278343	0.350268
H	-2.304325	10.157891	1.243491
H	-2.437308	11.888776	1.512594
C	0.664718	11.149055	1.885898
H	0.683003	11.346782	0.806822
H	1.185558	11.989612	2.361198
H	1.274189	10.258758	2.066174
N	6.974271	1.088935	-3.460794
C	7.017394	1.203451	-2.117591
H	7.579525	2.034359	-1.714749
C	6.383516	0.296146	-1.245696
H	6.487136	0.450865	-0.179476
C	5.656643	-0.802307	-1.770939
C	5.609786	-0.898099	-3.184761
H	5.089626	-1.706848	-3.681998
C	6.274265	0.058241	-3.977793
H	6.243874	-0.021762	-5.055223
C	5.035351	-1.771150	-0.926163
C	4.638759	-2.593887	1.151883
C	4.731239	-2.449478	2.571071
C	5.335138	-1.313553	3.168245
H	5.760095	-0.516932	2.571517
C	5.416130	-1.209596	4.570280
H	5.884544	-0.345667	5.021200
C	4.356376	-3.237804	4.861299
H	3.973973	-3.983977	5.542843
C	4.235929	-3.429490	3.468140
H	3.773319	-4.338836	3.106060
C	3.935632	-3.738868	-0.679087
C	3.267852	-4.858525	-1.262633
C	2.861119	-5.973711	-0.490037
H	3.058534	-6.025111	0.573211
C	2.970542	-4.922332	-2.648500
H	3.239483	-4.120911	-3.324352
C	2.299438	-6.043792	-3.176675
H	2.066373	-6.085937	-4.231642
C	2.199011	-7.054653	-1.105319
H	1.886768	-7.899728	-0.509547
C	-4.291520	-0.570249	-6.215116
H	-3.795314	-0.639529	-7.172388
C	-4.113118	-1.589213	-5.260282
H	-3.479970	-2.428408	-5.518043
C	-5.681678	0.618185	-4.806986
H	-6.299087	1.491274	-4.651416
C	-5.554303	-0.346167	-3.787466
H	-6.091803	-0.191494	-2.861015
C	-4.757536	-1.499967	-4.000534
C	-4.605807	-2.511170	-3.005056
C	-4.946614	-3.250120	-0.886830
C	-3.649306	-4.450989	-2.310964
C	-2.733366	-5.518948	-2.559055
C	-2.372049	-6.463708	-1.565491
H	-2.818787	-6.441788	-0.580268
C	-2.106183	-5.679249	-3.819909
H	-2.312609	-5.016762	-4.650748
C	-1.160802	-6.700882	-4.015513
H	-0.673649	-6.795418	-4.975160
C	-1.421744	-7.465812	-1.859782
H	-1.152175	-8.191282	-1.105878
C	-5.489686	-3.031453	0.416327
C	-6.274583	-1.888473	0.716794
H	-6.499999	-1.138627	-0.030692
C	-6.803484	-1.716060	2.010107

H	-7.410138	-0.849908	2.234778
C	-5.848443	-3.681836	2.750726
H	-5.689807	-4.374506	3.564904
C	-5.279828	-3.938737	1.485760
H	-4.703561	-4.844622	1.351725
C	0.192288	6.334083	4.323048
H	1.108685	6.906102	4.347746
C	-2.038952	6.230536	3.741866
H	-2.898076	6.723931	3.310279
C	-2.153343	4.911511	4.223640
H	-3.114672	4.419257	4.156114
C	-1.028740	4.266620	4.799005
C	0.170492	5.022044	4.835138
H	1.081525	4.616296	5.254747
C	-1.100198	2.936423	5.313840
C	-2.294381	1.045869	5.709573
C	-0.105266	1.150517	6.304853
N	-5.949805	-1.171607	5.379208
C	-3.511098	0.306905	5.598361
C	-4.679590	0.866128	5.022851
H	-4.697870	1.884237	4.656621
C	-5.858775	0.100071	4.938256
H	-6.752525	0.534084	4.512435
C	-3.623078	-1.030099	6.055840
H	-2.792157	-1.538217	6.526510
C	-4.843792	-1.720527	5.921958
H	-4.926802	-2.737331	6.276520
C	1.082995	0.491324	6.747095
C	1.077242	-0.855439	7.190146
H	0.157708	-1.420852	7.264978
C	2.286248	-1.485580	7.546692
H	2.276596	-2.511652	7.884394
C	3.502903	0.417436	7.102935
H	4.464983	0.909820	7.084517
C	2.347900	1.129663	6.724442
H	2.455173	2.162455	6.419105
C	-2.282685	2.494522	-6.523337
H	-2.123385	1.792321	-7.329329
C	-3.772186	3.749670	-5.285093
H	-4.795845	4.043744	-5.103513
C	-2.738736	4.280779	-4.489207
H	-2.999249	4.994113	-3.718607
C	-1.187275	2.966408	-5.774476
H	-0.198525	2.611249	-6.033381
C	-1.394799	3.891835	-4.720084
C	-0.313778	4.401671	-3.939223
C	0.435208	5.628435	-2.179681
C	1.907664	4.502916	-3.489215
C	0.165899	6.442885	-1.037812
C	-1.148556	6.853478	-0.700162
H	-2.002584	6.579299	-1.305272
C	-1.365357	7.656236	0.436826
H	-2.367381	7.972870	0.687717
C	0.883219	7.692939	0.941853
H	1.672289	8.038398	1.594728
C	1.196773	6.889887	-0.172663
H	2.235355	6.642682	-0.350800
C	3.251722	4.165628	-3.831136
C	4.357395	4.615187	-3.067467
H	4.227145	5.242976	-2.195334
C	5.666107	4.255546	-3.444484
H	6.504541	4.601367	-2.856843
C	3.562222	3.373654	-4.964771
H	2.791463	3.005708	-5.629529
C	4.904378	3.071438	-5.266080
H	5.136857	2.474756	-6.136872
C	9.432607	0.247678	-5.515582
H	8.377616	0.164764	-5.790950
H	10.011169	0.077155	-6.432061
H	9.665228	-0.589551	-4.845740
C	10.252630	1.420477	-3.527609
H	9.606322	1.921355	-2.801373
H	10.344149	0.371670	-3.218860
H	11.251409	1.860845	-3.417127
C	10.600316	2.392874	-5.754357
H	11.522081	2.649001	-5.216433
H	10.948270	1.813900	-6.619444
C	10.038858	3.722136	-6.317030
H	10.740718	4.508185	-6.010199
H	10.155072	3.671312	-7.407204
C	8.607527	5.354318	-5.172267
H	8.255968	5.161324	-4.155011
H	9.592203	5.829422	-5.080161
H	7.942897	6.108158	-5.612612
C	7.777801	4.181340	-7.156311
H	7.268371	5.149393	-7.242685
H	8.331022	4.041164	-8.093399
H	7.005945	3.406885	-7.128049

N	-9.251656	-3.599375	4.313622
N	-8.580876	-2.116518	6.730735
C	-9.811956	-3.056348	3.047163
H	-9.687166	-3.739453	2.197842
H	-10.885722	-2.838741	3.108914
H	-9.318274	-2.115998	2.788982
C	-8.730522	-4.977715	4.117340
H	-8.817955	-5.324777	3.080107
H	-7.672700	-5.017255	4.392061
H	-9.242603	-5.724863	4.736643
C	-10.274458	-3.565754	5.399747
H	-10.527566	-4.582286	5.727327
H	-11.225625	-3.158140	5.033983
C	-9.919632	-2.773746	6.681912
H	-10.723823	-2.041948	6.830959
H	-10.038033	-3.474959	7.518010
C	-8.719263	-0.653331	6.962366
H	-8.140704	-0.301632	7.825716
H	-8.370954	-0.097723	6.087362
H	-9.755696	-0.340588	7.141462
C	-7.718622	-2.739617	7.769140
H	-7.329682	-2.014002	8.494552
H	-8.235970	-3.511663	8.352211
H	-6.860327	-3.226001	7.296873
N	-0.106491	-10.416840	-4.127846
N	2.702154	-9.914051	-3.516297
N	9.693111	1.574729	-4.896814
N	8.639291	4.085418	-5.947417
N	-1.270019	9.766092	5.027374
N	-0.709597	10.938113	2.413354
N	5.144438	-1.585838	0.407302
N	4.032355	-3.704905	0.672264
N	4.379470	-2.786256	-1.528977
N	1.921410	-7.097665	-2.424474
N	4.941036	-2.151203	5.409422
N	-5.068340	0.511397	-6.003349
N	-3.834007	-3.572450	-3.320978
N	-5.192021	-2.292033	-1.808012
N	-4.165099	-4.343240	-1.062629
N	-6.604542	-2.595309	3.012226
N	-0.816530	-7.587884	-3.061084
N	-0.891260	6.936922	3.792660
N	-2.287791	2.300021	5.205299
N	-1.237469	0.410263	6.265582
N	0.029649	2.414952	5.844344
N	3.486639	-0.870788	7.505180
N	-3.553823	2.878855	-6.291462
N	-0.624867	5.236110	-2.922180
N	1.722541	5.277985	-2.397613
N	0.928250	3.971594	-4.254059
N	5.948136	3.498562	-4.525686
N	-0.372886	8.077949	1.247019
Pd	5.260646	-2.003229	7.494104
Pd	-0.807854	8.927175	3.116443
Pd	0.927818	-8.750214	-3.280380
Pd	7.814760	2.562595	-4.703452
Pd	-5.143763	2.079954	-7.403117
Pd	-7.593354	-2.367623	4.856497
C	0.877938	-5.816633	3.797769
C	1.033297	-4.431757	3.775548
C	0.846674	-3.717677	2.580613
C	0.504466	-4.418791	1.412126
C	0.319159	-5.793731	1.444467
C	0.514299	-6.502753	2.632546
H	1.016760	-6.362739	4.720082
H	1.271142	-3.921186	4.699243
C	0.763750	-2.243587	2.498526
C	0.001728	-3.539584	0.277143
H	-0.018383	-6.305900	0.552498
H	0.365414	-7.575930	2.665844
C	0.918684	-2.341813	0.063117
C	1.226725	-1.625931	1.239351
H	-0.120533	-4.102768	-0.655250
C	-1.822722	-1.889106	-0.202632
C	-1.375288	-3.016394	0.737690
C	-1.384700	-2.321006	2.078563
C	-1.788119	-0.939406	1.885435
H	-2.153674	-3.866319	0.684063
H	-1.717010	-2.817454	2.998195
C	-1.885470	0.594602	-0.233372
C	-3.287982	0.884609	-0.795190
C	-1.333501	1.768907	0.579375
H	-1.211138	0.433590	-1.083017
C	-3.248294	2.147486	-1.665330
H	-3.980210	1.018286	0.044567
H	-3.633197	0.024754	-1.375884
C	-1.304255	3.023705	-0.302508
H	-1.974809	1.948596	1.450142

H	-0.336185	1.549538	0.963393
C	-2.697226	3.338886	-0.869217
H	-4.253271	2.369718	-2.038734
H	-2.611789	1.942757	-2.537928
H	-0.921487	3.861142	0.290991
H	-0.594202	2.870395	-1.129252
H	-2.676082	4.236966	-1.492057
H	-3.381893	3.554987	-0.040062
N	-1.865014	-0.705861	0.486818
O	-1.956585	-0.061783	2.735173
O	-1.970363	-1.999004	-1.418462
C	1.822319	-0.356383	1.129399
C	1.207708	-1.809230	-1.192508
C	1.791892	-0.541354	-1.281497
H	2.022850	-0.124131	-2.258756
C	2.092510	0.176277	-0.126046
H	2.529156	1.166643	-0.188961
H	2.025351	0.234846	2.004658
H	0.929356	-2.357804	-2.087511
C	0.953664	-1.469652	3.798561
H	2.027525	-1.499643	4.062051
H	0.411248	-1.979735	4.597840
O	0.580431	-0.113549	3.728492
H	-0.385545	-0.074685	3.539296
O	-0.396982	-3.217009	-3.507001
H	-0.504345	-2.458212	-4.105657
H	-1.081556	-3.051995	-2.840327
O	1.057554	2.518225	2.656206
H	1.948600	2.860862	2.794069
H	1.022902	1.704445	3.188178
O	-1.298650	-0.290611	-3.577842
H	-1.641437	-0.768668	-2.805439
H	-0.374302	-0.127412	-3.350538

Table5_1a_reactant_0wat

Property	Value
Charge	0
Electronic Energy, BS1 (a.u.)	-980.278057
Thermal and entropic correction, BS1 (a.u.)	2.693352
Electronic Energy, BS2 (a.u.)	-980.613279
Number of Imaginary Frequencies	0
Imaginary frequencies (cm-1)	None

Molecular Geometry in Cartesian Coordinates

C	-8.073030	2.835865	5.886800
C	-8.640625	3.013763	4.643638
C	-8.030456	3.885510	3.687938
C	-7.067521	4.819276	4.160682
C	-6.770665	4.825639	5.561233
C	-7.111398	3.756522	6.353795
H	-9.039791	3.102602	1.934381
H	-8.475979	2.090130	6.581745
H	-9.518513	2.436843	4.349057
C	-8.301134	3.809935	2.296290
C	-6.408104	5.666268	3.234543
H	-6.192490	5.649150	5.974633
H	-6.786881	3.707077	7.395673
C	-6.649069	5.534162	1.883397
C	-7.601399	4.602211	1.413395
H	-5.679730	6.385225	3.600727
H	-7.785686	4.522236	0.349078
H	-6.109226	6.154644	1.173346
C	-10.432451	4.284744	6.702461
C	-9.924062	5.025565	5.510335
C	-9.030693	5.899305	5.897883
C	-8.868882	5.825498	7.374871
H	-10.396115	4.878154	4.507328
H	-8.526598	6.702481	5.315942
C	-9.956384	4.403966	9.182165
C	-8.682170	3.823723	9.801510
C	-10.507371	5.563675	10.030903
H	-10.716438	3.618149	9.118249
C	-8.938403	3.371166	11.247529
H	-7.903231	4.593492	9.778974
H	-8.332726	2.980564	9.189578
C	-10.775601	5.100179	11.469445
H	-9.768448	6.372132	10.029543
H	-11.419158	5.942941	9.547222
C	-9.502177	4.514771	12.098312
H	-8.008202	2.992045	11.681724
H	-9.648701	2.533890	11.247442
H	-11.145925	5.937117	12.071596

H	-11.564259	4.335662	11.467456
H	-9.714801	4.159983	13.111476
H	-8.748620	5.307942	12.191155
N	-9.747429	4.824932	7.796190
O	-8.175722	6.507962	8.115019
O	-11.292769	3.427705	6.771316
C	-12.403174	-3.773593	7.638379
H	-12.259183	-4.442326	8.496196
H	-11.780608	-2.892599	7.818949
H	-13.450930	-3.449711	7.672941
C	-13.201453	-4.452664	5.423738
H	-14.101727	-3.994341	5.851779
H	-12.974911	-3.923461	4.494367
H	-13.482240	-5.475368	5.142532
C	-11.455567	-5.758773	6.547439
H	-12.037059	-6.517881	6.008756
H	-11.534695	-6.059261	7.600119
C	-9.975968	-5.971341	6.144066
H	-9.962239	-6.810181	5.436421
H	-9.469078	-6.365006	7.034434
C	-8.728816	-5.082979	4.227395
H	-9.223216	-4.436108	3.497131
H	-7.647788	-4.920774	4.132875
H	-8.912248	-6.116055	3.906190
C	-8.134838	-4.388450	6.501235
H	-8.343770	-3.401542	6.922680
H	-7.999739	-5.070255	7.350283
H	-7.163910	-4.333015	5.993324
N	0.180147	5.665912	6.791635
N	-0.768908	8.284699	7.652065
C	-0.153495	4.444212	7.570124
H	-1.213525	4.453984	7.840146
H	0.037690	3.517238	7.014902
H	0.409806	4.363792	8.508190
C	0.719319	5.312090	5.451063
H	0.110494	5.770913	4.667389
H	1.747716	5.660626	5.293409
H	0.731130	4.230426	5.267689
C	1.141082	6.530955	7.537163
H	1.494642	6.026354	8.445515
H	2.054595	6.699023	6.952402
C	0.641738	7.927583	7.981646
H	1.342624	8.659987	7.561021
H	0.813556	7.984168	9.064266
C	-0.823663	9.529447	6.839411
H	-1.479946	10.294013	7.273729
H	0.157114	10.004715	6.712044
H	-1.197465	9.307971	5.835998
C	-1.586427	8.417832	8.886477
H	-2.350937	7.636260	8.911763
H	-0.998491	8.310819	9.806674
H	-2.098388	9.385663	8.958120
N	-20.157153	3.698347	-1.939616
N	-18.888535	4.953663	-4.248571
C	-19.998976	2.254657	-1.620603
H	-20.308269	2.006314	-0.597591
H	-20.576501	1.599663	-2.284854
H	-18.950029	1.963436	-1.726227
C	-20.728417	4.439150	-0.783470
H	-20.898554	3.799730	0.091851
H	-20.046537	5.237812	-0.477915
H	-21.690916	4.915141	-1.008800
C	-20.993308	3.887670	-3.161068
H	-21.886283	4.484631	-2.935654
H	-21.394644	2.928110	-3.511456
C	-20.319481	4.552480	-4.387236
H	-20.450287	3.856998	-5.226152
H	-20.943919	5.414653	-4.654387
C	-18.728896	6.421272	-4.428260
H	-17.989347	6.680111	-5.196374
H	-19.660059	6.924701	-4.716603
H	-18.402187	6.878876	-3.490372
C	-18.030543	4.204836	-5.204869
H	-17.419163	4.862116	-5.835856
H	-17.348676	3.547127	-4.658518
H	-18.602958	3.566522	-5.889364
C	-7.384892	16.150521	-0.040323
H	-7.300381	16.279821	1.046030
H	-6.974137	15.167352	-0.286745
H	-6.715352	16.893505	-0.491474
C	-9.719205	16.546755	0.591118
H	-9.215082	16.602849	1.563954
H	-10.255467	17.495893	0.467856
H	-10.479292	15.763595	0.660109
C	-8.902909	17.279639	-1.604378
H	-7.947871	17.799279	-1.754479
H	-9.597410	18.076892	-1.310166
C	-9.361227	16.794628	-3.002017

H	-10.244972	17.390811	-3.263028
H	-8.593967	17.129676	-3.711645
C	-11.051359	15.102544	-3.555824
H	-11.149911	14.509723	-4.473898
H	-11.572950	14.563481	-2.759940
H	-11.611523	16.030322	-3.727086
C	-8.705266	14.728700	-4.155020
H	-9.226057	14.200000	-4.963164
H	-8.049737	15.462986	-4.639583
H	-8.052649	14.005783	-3.657422
N	-5.429233	2.143156	-3.410757
C	-4.670236	2.943785	-2.634027
H	-4.025838	3.649652	-3.138415
C	-4.683631	2.889574	-1.225749
H	-4.032427	3.557807	-0.677047
C	-5.528142	1.965038	-0.560707
C	-6.329006	1.142804	-1.393539
H	-7.002486	0.401601	-0.982703
C	-6.245497	1.265011	-2.793824
H	-6.851168	0.631713	-3.426636
C	-5.563627	1.858411	0.862459
C	-4.818145	2.505406	2.905917
C	-4.029967	3.387053	3.707302
C	-3.222393	4.401573	3.133089
H	-3.148926	4.528675	2.060780
C	-2.494642	5.276801	3.962324
H	-1.878747	6.047742	3.521389
C	-3.272096	4.243966	5.872368
H	-3.278699	4.194516	6.951359
C	-4.028897	3.320500	5.123280
H	-4.598662	2.570991	5.651725
C	-6.396034	0.882664	2.734219
C	-7.310228	-0.031977	3.341023
C	-7.379481	-0.210288	4.745484
H	-6.724778	0.333879	5.413825
C	-8.205741	-0.817469	2.570625
H	-8.226211	-0.762278	1.489683
C	-9.091667	-1.707786	3.207277
H	-9.768126	-2.309499	2.616485
C	-8.305940	-1.119549	5.295860
H	-8.360792	-1.249902	6.366576
C	-16.828911	2.891494	-0.116166
H	-16.842554	2.141431	-0.893804
C	-16.128032	2.644358	1.080112
H	-15.618048	1.696151	1.191745
C	-17.488135	4.980051	0.606415
H	-18.028474	5.893782	0.404216
C	-16.814605	4.823200	1.833316
H	-16.857908	5.631284	2.552241
C	-16.107163	3.624818	2.104043
C	-15.416281	3.416594	3.335617
C	-14.805378	4.147539	5.395703
C	-14.153729	2.094333	4.680821
C	-13.399696	0.896878	4.875269
C	-12.706866	0.628845	6.082651
H	-12.737388	1.320013	6.915168
C	-13.292831	-0.093009	3.865442
H	-13.788141	0.015677	2.908966
C	-12.529643	-1.253883	4.094189
H	-12.451745	-2.006190	3.322077
C	-11.965209	-0.561367	6.220820
H	-11.438877	-0.762802	7.141419
C	-14.774971	5.182723	6.379420
C	-15.385449	6.443422	6.157608
H	-15.901572	6.669423	5.233243
C	-15.326681	7.439758	7.150799
H	-15.791263	8.399807	6.975543
C	-14.135952	6.066644	8.571082
H	-13.663085	5.929643	9.532072
C	-14.131952	5.015240	7.632180
H	-13.643369	4.085012	7.894492
C	-7.841218	12.748822	0.792783
H	-7.026232	12.927977	0.106244
C	-10.048233	13.115700	1.355568
H	-10.990811	13.587103	1.117089
C	-9.939441	12.313335	2.507669
H	-10.813587	12.192164	3.134471
C	-8.704521	11.693032	2.825054
C	-7.638043	11.931164	1.921740
H	-6.656173	11.504008	2.078455
C	-8.544141	10.883026	3.989490
C	-9.398824	10.003859	5.900769
C	-7.226243	9.632561	5.350519
N	-12.758847	9.326191	8.474644
C	-10.514434	9.786711	6.766093
C	-11.808649	10.278247	6.456917
H	-12.000656	10.846823	5.555828
C	-12.886680	10.020754	7.325940

H	-13.873026	10.391289	7.084822
C	-10.398638	9.063092	7.979739
H	-9.446217	8.675519	8.316013
C	-11.533719	8.857322	8.789349
H	-11.440899	8.307793	9.714207
C	-5.966530	9.015693	5.622579
C	-5.723865	8.295919	6.819228
H	-6.478974	8.211313	7.589396
C	-4.471602	7.687770	7.037817
H	-4.296905	7.135713	7.949921
C	-3.661067	8.453585	5.017726
H	-2.833285	8.513475	4.324964
C	-4.881559	9.087739	4.711894
H	-4.961195	9.628015	3.777076
C	-15.278047	4.406976	-3.037095
H	-15.544942	3.384887	-3.264474
C	-15.945748	6.490872	-2.301915
H	-16.743583	7.126117	-1.944344
C	-14.642745	7.008797	-2.433704
H	-14.472028	8.045920	-2.174899
C	-13.946664	4.836327	-3.199597
H	-13.215304	4.123202	-3.557730
C	-13.593508	6.176654	-2.900395
C	-12.260601	6.661035	-3.063256
C	-10.758860	8.358488	-2.944411
C	-10.102603	6.311296	-3.671621
C	-10.451258	9.718648	-2.639382
C	-11.434250	10.622722	-2.163180
H	-12.460595	10.314872	-2.009416
C	-11.085490	11.958845	-1.885686
H	-11.838082	12.646393	-1.526534
C	-8.889244	11.596351	-2.491282
H	-7.892110	11.994431	-2.612443
C	-9.144462	10.245594	-2.796677
H	-8.325214	9.634586	-3.153161
C	-9.078531	5.442275	-4.155953
C	-7.750610	5.891839	-4.366588
H	-7.460398	6.916211	-4.170596
C	-6.773906	5.000890	-4.851829
H	-5.763631	5.348829	-5.012690
C	-9.329134	4.080456	-4.459821
H	-10.312047	3.643781	-4.336171
C	-8.287551	3.264089	-4.942467
H	-8.483715	2.227320	-5.175840
C	-4.223220	-0.289003	-5.226034
H	-5.252850	-0.247193	-4.859817
H	-4.174472	-1.127179	-5.932322
H	-3.583265	-0.565217	-4.378712
C	-2.648119	1.586686	-5.166530
H	-2.885197	2.574472	-4.761383
H	-2.285415	0.967838	-4.336302
H	-1.796390	1.721221	-5.844988
C	-3.616141	0.866520	-7.303822
H	-2.580629	1.120320	-7.564359
H	-3.720698	-0.181008	-7.614032
C	-4.518888	1.695418	-8.250601
H	-3.842898	2.277760	-8.889756
H	-4.986820	0.980225	-8.939055
C	-5.278021	4.007741	-7.929400
H	-5.027333	4.547606	-7.011782
H	-4.436734	4.150764	-8.618809
H	-6.138223	4.514288	-8.384867
C	-6.914512	2.188818	-8.048396
H	-7.481125	3.023971	-8.478918
H	-6.922385	1.391139	-8.801827
H	-7.481715	1.816235	-7.190933
N	-16.156261	8.417240	10.835906
N	-14.148533	10.523955	10.982613
C	-17.323519	8.374354	9.914461
H	-17.766950	7.374058	9.833057
H	-18.136947	9.046630	10.215113
H	-17.020397	8.681485	8.909889
C	-15.919500	7.087019	11.456535
H	-16.618560	6.319308	11.101443
H	-14.906619	6.743452	11.225929
H	-16.006441	7.099827	12.550275
C	-16.343743	9.468206	11.878638
H	-16.391745	9.019451	12.879092
H	-17.313887	9.968431	11.763513
C	-15.277737	10.588462	11.955950
H	-15.817773	11.540068	11.870638
H	-14.903144	10.585214	12.987656
C	-14.098026	11.754635	10.148446
H	-13.117493	12.246505	10.171469
H	-14.319318	11.511960	9.105383
H	-14.827780	12.514065	10.455986
C	-12.855438	10.302277	11.681759
H	-12.093564	11.046333	11.417211

H	-12.946925	10.330826	12.774631
H	-12.457640	9.315853	11.425568
N	-12.031747	-4.397982	6.341027
N	-9.240551	-4.794851	5.594324
N	-3.845735	1.013980	-5.836514
N	-5.544216	2.577651	-7.620034
N	-8.785903	16.252178	-0.528641
N	-9.635162	15.336661	-3.166453
N	-4.748100	2.673904	1.565701
N	-5.605984	1.614352	3.553129
N	-6.431210	0.966118	1.386514
N	-9.144859	-1.863654	4.545213
N	-2.512975	5.206515	5.309130
N	-17.504846	4.034094	-0.355215
N	-14.766766	2.241085	3.485267
N	-15.450927	4.420500	4.239778
N	-14.168589	2.992718	5.690228
N	-14.720458	7.261095	8.342264
N	-11.877473	-1.493427	5.249955
N	-9.020629	13.339982	0.511343
N	-9.625190	10.731740	4.785618
N	-8.221856	9.431902	6.244243
N	-7.329911	10.323985	4.193909
N	-3.451632	7.764466	6.158013
N	-16.265900	5.216053	-2.603487
N	-12.035541	7.957716	-2.754330
N	-9.746535	7.585799	-3.397872
N	-11.337203	5.783667	-3.517350
N	-7.029493	3.708872	-5.142319
N	-9.838387	12.446482	-2.049224
Pd	-1.644818	6.727232	6.479162
Pd	-9.321182	14.339030	-1.309854
Pd	-10.570369	-3.135027	5.429597
Pd	-5.461670	2.362990	-5.500684
Pd	-18.204286	4.481879	-2.285230
Pd	-14.439515	8.877066	9.659007

Table5_1a_TSiii_TSi-ii_0wat

Property	Value
Charge	0
Electronic Energy, BS1 (a.u.)	-980.236305
Thermal and entropic correction, BS1 (a.u.)	2.698789
Electronic Energy, BS2 (a.u.)	-980.567366
Number of Imaginary Frequencies	0
Imaginary frequencies (cm-1)	None

Molecular Geometry in Cartesian Coordinates

C	-9.159326	8.950264	4.605105
H	-9.989022	8.772934	5.300709
H	-8.266989	8.518705	5.066884
H	-9.018910	10.037701	4.564584
C	-9.401686	9.387304	2.206152
H	-9.211774	10.395320	2.595703
H	-8.635587	9.176588	1.455038
H	-10.359037	9.440241	1.672756
C	-10.635407	7.538775	3.244510
H	-11.320495	7.898381	2.466040
H	-11.197864	7.671386	4.177645
C	-10.494669	6.010956	3.037458
H	-11.143038	5.752319	2.190506
H	-10.980108	5.536315	3.899898
C	-8.998866	4.795994	1.519204
H	-8.307292	5.347014	0.876139
H	-8.627340	3.766456	1.596710
H	-9.951059	4.737242	0.977322
C	-8.745398	4.545031	3.942989
H	-7.917731	4.968028	4.519786
H	-9.564284	4.365176	4.650728
H	-8.426724	3.558251	3.584471
N	2.659885	-1.134187	7.376387
N	4.636645	0.528958	8.729382
C	1.231080	-1.117507	7.786976
H	0.899088	-0.086185	7.935847
H	0.566384	-1.574812	7.043175
H	1.047445	-1.646218	8.730603
C	2.826702	-1.805017	6.059290
H	3.299168	-1.122640	5.347296
H	3.460810	-2.699150	6.107240
H	1.874143	-2.128365	5.621298
C	3.507382	-1.787585	8.416459
H	2.883823	-2.232353	9.202621
H	4.057470	-2.639916	7.997503

C	4.551115	-0.902731	9.140812
H	5.520164	-1.404263	9.022476
H	4.335407	-0.987636	10.213653
C	5.998027	0.854694	8.226878
H	6.446352	1.716650	8.736559
H	6.709484	0.027015	8.340064
H	5.957850	1.088703	7.159704
C	4.252864	1.428879	9.848646
H	3.337009	1.969189	9.593639
H	4.051311	0.891923	10.784044
H	5.023279	2.174704	10.081364
N	-1.670016	17.637765	-3.488736
N	0.305553	16.921403	-5.514252
C	-3.034880	17.050135	-3.431253
H	-3.531446	17.215812	-2.466938
H	-3.709929	17.451450	-4.197423
H	-2.983941	15.970049	-3.593675
C	-1.341862	18.345474	-2.222899
H	-2.140362	18.273399	-1.473956
H	-0.436923	17.919174	-1.780937
H	-1.149236	19.416333	-2.364248
C	-1.530866	18.550107	-4.661362
H	-1.282125	19.568875	-4.337417
H	-2.488773	18.662717	-5.185108
C	-0.486976	18.166093	-5.739305
H	-1.029510	18.117056	-6.692191
H	0.174071	19.034889	-5.852607
C	1.756264	17.227927	-5.400581
H	2.371515	16.649626	-6.101203
H	1.990376	18.283687	-5.586059
H	2.107719	17.000802	-4.390057
C	0.055093	15.928864	-6.593106
H	0.975692	15.590638	-7.084893
H	-0.439543	15.045059	-6.180458
H	-0.595797	16.311527	-7.389093
C	12.378385	8.508267	2.650697
H	12.192895	8.496938	3.732035
H	11.626532	7.867165	2.182055
H	13.348453	8.018385	2.499993
C	12.053416	10.891145	3.118828
H	11.916464	10.450599	4.114307
H	12.859785	11.628681	3.217597
H	11.145004	11.450143	2.879331
C	13.566652	10.193023	1.317898
H	14.304013	9.387536	1.427859
H	14.065176	11.072520	1.745166
C	13.435838	10.460709	-0.201867
H	13.874350	11.450639	-0.381648
H	14.128220	9.768161	-0.697420
C	11.665163	11.662537	-1.404232
H	11.363829	11.568659	-2.454994
H	10.816245	12.080998	-0.856193
H	12.459969	12.418459	-1.379473
C	12.006365	9.268840	-1.802851
H	11.646754	9.606270	-2.783020
H	12.976167	8.787526	-1.980589
H	11.326588	8.485541	-1.455453
N	0.726266	3.134881	-3.877019
C	1.454478	2.647975	-2.851561
H	2.429779	2.247573	-3.090124
C	0.999864	2.642277	-1.518036
H	1.644227	2.221799	-0.756624
C	-0.279102	3.167398	-1.202295
C	-1.025846	3.683360	-2.291934
H	-2.013906	4.103688	-2.154718
C	-0.488525	3.647711	-3.593889
H	-1.058368	4.043613	-4.422712
C	-0.782970	3.179868	0.133837
C	-0.496839	2.759929	2.345276
C	0.313471	2.262396	3.410389
C	1.609549	1.733657	3.184029
H	2.033203	1.674798	2.189706
C	2.374413	1.255811	4.265996
H	3.358764	0.845383	4.088503
C	0.709143	1.769454	5.775097
H	0.367202	1.773524	6.799600
C	-0.129175	2.264661	4.756597
H	-1.107993	2.638109	5.028598
C	-2.396498	3.765759	1.618665
C	-3.621662	4.445561	1.898016
C	-4.109560	4.612024	3.219243
H	-3.579628	4.206146	4.071340
C	-4.411593	5.016282	0.867830
H	-4.128812	4.938491	-0.174096
C	-5.602177	5.697148	1.186524
H	-6.202798	6.127985	0.397853
C	-5.314509	5.309750	3.442465
H	-5.689344	5.431099	4.448511

C	-1.846396	14.172862	-1.847271
H	-2.334240	13.946556	-2.784739
C	-2.250000	13.514664	-0.669722
H	-3.056484	12.795133	-0.734489
C	-0.237955	15.382463	-0.715372
H	0.549944	16.121072	-0.753912
C	-0.572763	14.773464	0.510312
H	-0.030908	15.071118	1.398929
C	-1.611767	13.809207	0.562026
C	-2.004273	13.181894	1.783286
C	-1.793987	12.948002	4.027840
C	-3.381945	11.777946	2.912860
C	-4.366740	10.743365	2.904256
C	-4.772962	10.085058	4.092432
H	-4.359457	10.355422	5.055107
C	-4.976439	10.295488	1.704869
H	-4.731708	10.736134	0.746887
C	-5.929099	9.258365	1.743640
H	-6.396055	8.921417	0.828919
C	-5.730042	9.052862	4.034271
H	-6.034316	8.550222	4.940540
C	-1.089074	13.214965	5.240514
C	0.033564	14.080232	5.284181
H	0.398002	14.581044	4.396403
C	0.683191	14.328079	6.509724
H	1.527713	15.001576	6.545764
C	-0.754795	12.928950	7.648669
H	-1.051428	12.483864	8.587361
C	-1.471877	12.629214	6.473932
H	-2.317909	11.957483	6.543731
C	8.886566	8.175104	2.465201
H	9.407769	7.402733	1.918010
C	8.593617	10.425881	2.882091
H	8.884336	11.444724	2.668532
C	7.579323	10.169111	3.825465
H	7.115180	11.009742	4.324567
C	7.196027	8.833505	4.109135
C	7.886161	7.822975	3.392254
H	7.666051	6.773302	3.538373
C	6.176705	8.525044	5.060117
C	4.636083	9.207077	6.578230
C	4.920405	6.996108	6.168294
N	2.608791	12.399950	8.595148
C	3.947521	10.268135	7.241403
C	4.266788	11.628947	7.002928
H	5.046705	11.916187	6.309596
C	3.581294	12.645641	7.694350
H	3.835895	13.681214	7.518004
C	2.913770	10.028121	8.182008
H	2.607169	9.023185	8.439794
C	2.280534	11.111639	8.824792
H	1.500471	10.928058	9.549650
C	4.558802	5.633555	6.400520
C	3.578514	5.262946	7.356352
H	3.071214	6.003501	7.961025
C	3.264544	3.902248	7.552813
H	2.521953	3.620898	8.285679
C	4.786559	3.237197	5.950946
H	5.254854	2.427438	5.409048
C	5.165490	4.567611	5.688354
H	5.931780	4.744615	4.944212
C	0.602982	13.211709	-4.324036
H	-0.357167	13.198699	-4.820180
C	2.230860	14.427123	-3.236412
H	2.569439	15.384968	-2.868164
C	3.040971	13.286916	-3.068706
H	3.997754	13.402661	-2.575918
C	1.343042	12.019309	-4.204395
H	0.925919	11.111604	-4.621633
C	2.606106	12.030535	-3.560895
C	3.395707	10.848775	-3.425409
C	5.268368	9.809669	-2.672786
C	3.689463	8.628721	-3.800049
C	6.513449	9.852176	-1.975521
C	7.004422	11.043246	-1.383207
H	6.457111	11.974909	-1.445865
C	8.237440	11.032926	-0.700987
H	8.611976	11.942375	-0.253235
C	8.547892	8.787539	-1.126960
H	9.166204	7.908121	-1.017148
C	7.329577	8.701836	-1.828089
H	7.044084	7.744622	-2.245517
C	3.222999	7.391572	-4.339658
C	3.967786	6.191245	-4.219020
H	4.926145	6.166031	-3.716105
C	3.468825	4.994108	-4.768721
H	4.042268	4.082589	-4.677172
C	1.989708	7.289331	-5.031270

H	1.351572	8.150676	-5.182661
C	1.571203	6.046531	-5.546296
H	0.630373	5.974583	-6.073312
C	-0.883785	1.254632	-6.071241
H	-1.150224	2.297966	-5.881001
H	-1.459278	0.938496	-6.950189
H	-1.257875	0.659431	-5.228960
C	1.169018	0.175659	-5.281409
H	1.936564	0.674941	-4.683704
H	0.424197	-0.233592	-4.587435
H	1.653013	-0.686854	-5.756621
C	0.911167	0.727694	-7.658504
H	1.438285	-0.234840	-7.675005
H	-0.003905	0.541045	-8.235084
C	1.765313	1.707130	-8.500669
H	2.636113	1.137215	-8.849173
H	1.193919	1.907710	-9.416031
C	3.672017	3.065246	-7.762835
H	3.987411	3.046403	-6.715768
H	4.181579	2.230576	-8.260233
H	4.074825	3.981988	-8.211470
C	1.631690	4.156632	-8.567940
H	2.401654	4.876208	-8.873363
H	1.084215	3.885856	-9.479433
H	0.924463	4.686395	-7.923528
N	0.322294	15.582927	10.193570
N	2.674951	14.155675	11.157548
C	0.327053	16.664928	9.172920
H	-0.667850	16.861686	8.754456
H	0.691192	17.624071	9.561995
H	0.984261	16.393427	8.341858
C	-1.055994	15.073129	10.420675
H	-1.802932	15.557713	9.779304
H	-1.094316	13.999414	10.218119
H	-1.401998	15.209644	11.452884
C	0.932906	16.055097	11.470755
H	0.197115	16.035863	12.284946
H	1.225636	17.110559	11.400268
C	2.173533	15.288117	11.989927
H	2.959208	16.038619	12.144947
H	1.925179	14.947940	13.003468
C	4.071794	14.404714	10.711516
H	4.753917	13.583084	10.963584
H	4.100623	14.534364	9.626317
H	4.509433	15.311248	11.148072
C	2.573937	12.867926	11.894077
H	3.530585	12.334295	11.955459
H	2.222138	12.987354	12.926463
H	1.860142	12.207560	11.393343
N	-9.379364	8.343567	3.265638
N	-9.117654	5.470139	2.840095
N	0.586224	1.132603	-6.259236
N	2.189608	2.980716	-7.848524
N	12.315626	9.874110	2.066110
N	12.071806	10.365538	-0.800384
N	0.027799	2.698049	1.101130
N	-1.696932	3.290668	2.674525
N	-2.007174	3.718795	0.324572
N	-6.055200	5.842071	2.448148
N	1.939730	1.267947	5.542930
N	-0.864587	15.096317	-1.874848
N	-3.015303	12.288407	1.715532
N	-1.306935	13.506229	2.895806
N	-2.869094	12.135188	4.112321
N	0.299363	13.769693	7.676047
N	-6.306370	8.646048	2.884770
N	9.243931	9.451143	2.214133
N	5.595364	9.565637	5.696118
N	4.257787	7.941141	6.875162
N	5.882396	7.219466	5.246226
N	3.857602	2.901909	6.868723
N	1.032508	14.401710	-3.855524
N	4.565515	10.962126	-2.755918
N	4.879650	8.610864	-3.160837
N	2.888849	9.707987	-3.944642
N	2.293368	4.913181	-5.425863
N	9.004008	9.929931	-0.574174
Pd	3.268196	0.896457	7.129341
Pd	10.653859	9.909855	0.724389
Pd	-7.710073	7.072215	2.863632
Pd	1.436119	3.046165	-5.855437
Pd	-0.291905	16.017911	-3.678783
Pd	1.473995	13.970696	9.404064
C	0.309619	5.302512	5.608337
C	1.197960	5.776561	4.595066
C	0.651315	5.987684	3.268767
C	-0.688867	6.438744	3.217353
C	-1.351472	6.611749	4.498980

C	-0.996030	5.710427	5.555070
H	2.412619	5.495315	2.118005
H	0.695282	4.779271	6.476724
H	2.257126	5.543569	4.659391
C	1.381716	5.830116	2.077643
C	-1.262852	6.778837	1.980750
H	-2.353362	7.031656	4.480671
H	-1.686186	5.520270	6.369408
C	-0.546697	6.578368	0.809459
C	0.771443	6.088664	0.859021
H	-2.268417	7.186689	1.951967
H	1.325556	5.939274	-0.061714
H	-1.004070	6.805883	-0.147083
C	-0.368922	9.169883	4.024094
C	-0.244664	8.245478	5.193033
C	1.117318	7.874257	5.283456
C	1.857159	8.608289	4.215555
H	-0.887553	8.414000	6.045314
H	1.630423	7.656406	6.208252
C	1.176410	10.194540	2.327064
C	1.950675	11.444259	2.779201
C	1.898697	9.456412	1.193550
H	0.183912	10.500181	1.977707
C	2.214495	12.374395	1.587388
H	2.901206	11.125772	3.222232
H	1.381203	11.963129	3.559603
C	2.187957	10.398467	0.017848
H	2.833927	9.048899	1.585902
H	1.287140	8.613920	0.868359
C	2.966323	11.640475	0.468738
H	2.771033	13.258038	1.911205
H	1.251393	12.729790	1.194110
H	2.741397	9.846567	-0.750078
H	1.240783	10.712571	-0.442156
H	3.140735	12.316776	-0.374730
H	3.955225	11.337856	0.837080
N	0.903000	9.313447	3.469721
O	3.058046	8.616533	4.004056
O	-1.382751	9.679011	3.583811

Table5_1a_TSiii_DG_0wat

Property	Value
Charge	0
Electronic Energy, BS1 (a.u.)	-980.286025
Thermal and entropic correction, BS1 (a.u.)	2.702273
Electronic Energy, BS2 (a.u.)	-980.613855
Number of Imaginary Frequencies	0
Imaginary frequencies (cm-1)	None

Molecular Geometry in Cartesian Coordinates

C	-9.215250	8.894920	4.668385
H	-10.032584	8.681997	5.368615
H	-8.304169	8.489330	5.117236
H	-9.111781	9.986821	4.639199
C	-9.482477	9.350092	2.275602
H	-9.316741	10.358565	2.674779
H	-8.715793	9.167456	1.517840
H	-10.443985	9.383390	1.748134
C	-10.659594	7.457233	3.299637
H	-11.361655	7.811812	2.534105
H	-11.216801	7.557045	4.239996
C	-10.478796	5.938177	3.060990
H	-11.109372	5.681474	2.200160
H	-10.963269	5.433775	3.906937
C	-8.939793	4.765866	1.552784
H	-8.257932	5.335464	0.915275
H	-8.541945	3.746417	1.632704
H	-9.886107	4.682048	1.003888
C	-8.696277	4.521087	3.978617
H	-7.887354	4.968008	4.563689
H	-9.516023	4.316978	4.678660
H	-8.345595	3.544381	3.622302
N	2.663629	-1.190429	7.535963
N	4.589052	0.601992	8.793108
C	1.243749	-1.205568	7.976116
H	0.879541	-0.180941	8.091176
H	0.580478	-1.714008	7.265053
H	1.097546	-1.701552	8.943796
C	2.827534	-1.910639	6.244500
H	3.251553	-1.240671	5.491066
H	3.501030	-2.774087	6.314249
H	1.879575	-2.293534	5.845984

C	3.554294	-1.769393	8.583859
H	2.961513	-2.218485	9.391116
H	4.140061	-2.607155	8.184357
C	4.559981	-0.812014	9.268833
H	5.548359	-1.279489	9.172802
H	4.346258	-0.857431	10.344459
C	5.934602	0.954683	8.266633
H	6.349750	1.859302	8.728143
H	6.678918	0.163039	8.419087
H	5.881994	1.129594	7.188801
C	4.178255	1.536608	9.873716
H	3.242426	2.032275	9.600861
H	4.000030	1.034840	10.833065
H	4.922439	2.319064	10.068357
N	-1.668204	17.601510	-3.521626
N	0.327839	16.865352	-5.519063
C	-3.031575	17.011024	-3.456656
H	-3.529444	17.189610	-2.495316
H	-3.707184	17.399593	-4.228909
H	-2.977774	15.928686	-3.602928
C	-1.345060	18.332403	-2.267931
H	-2.145239	18.272064	-1.519670
H	-0.440181	17.916131	-1.816542
H	-1.154339	19.400896	-2.428329
C	-1.529148	18.493128	-4.710022
H	-1.298301	19.521129	-4.402416
H	-2.482366	18.582223	-5.246688
C	-0.466686	18.104278	-5.767918
H	-0.994659	18.043467	-6.728254
H	0.192039	18.975083	-5.879539
C	1.775971	17.178683	-5.392308
H	2.401148	16.596643	-6.080903
H	2.007860	18.233748	-5.584618
H	2.117148	16.961471	-4.376002
C	0.093547	15.859451	-6.589130
H	1.021028	15.517839	-7.065327
H	-0.403984	14.979350	-6.172234
H	-0.548144	16.230836	-7.397831
C	12.399661	8.419886	2.693386
H	12.225886	8.419347	3.776671
H	11.634109	7.784825	2.238588
H	13.361399	7.915651	2.536317
C	12.117012	10.811118	3.148121
H	11.990891	10.380119	4.149222
H	12.934153	11.538954	3.227624
H	11.211860	11.379905	2.919310
C	13.594254	10.076789	1.331380
H	14.315013	9.254367	1.424442
H	14.119544	10.942296	1.755252
C	13.443507	10.358128	-0.184269
H	13.909881	11.334971	-0.365311
H	14.104002	9.647946	-0.698003
C	11.674053	11.638706	-1.306262
H	11.360027	11.590572	-2.356448
H	10.837294	12.047920	-0.733005
H	12.481062	12.380544	-1.260458
C	11.956922	9.255157	-1.796634
H	11.582189	9.634911	-2.755391
H	12.913035	8.763155	-2.014681
H	11.269758	8.472564	-1.463526
N	0.704645	3.057390	-3.813772
C	1.441648	2.594897	-2.783097
H	2.407178	2.171848	-3.021965
C	1.005479	2.637310	-1.444105
H	1.653018	2.231192	-0.677701
C	-0.263577	3.185746	-1.129247
C	-1.016568	3.681854	-2.223743
H	-1.998491	4.116473	-2.087271
C	-0.498220	3.597258	-3.530681
H	-1.075711	3.971291	-4.364217
C	-0.759425	3.225221	0.208975
C	-0.513133	2.741474	2.411795
C	0.262116	2.174217	3.468616
C	1.561534	1.652641	3.248553
H	2.008344	1.639658	2.262900
C	2.303577	1.130588	4.325461
H	3.292510	0.730139	4.150854
C	0.605129	1.569125	5.815736
H	0.237434	1.526068	6.830373
C	-0.216007	2.100176	4.800636
H	-1.206309	2.447490	5.064308
C	-2.385134	3.796652	1.684333
C	-3.603972	4.488834	1.959473
C	-4.105831	4.642492	3.276796
H	-3.591075	4.220572	4.130142
C	-4.376977	5.079126	0.927122
H	-4.083819	5.010642	-0.112583
C	-5.567412	5.763117	1.239564

H	-6.157614	6.206318	0.449669
C	-5.307158	5.347852	3.494651
H	-5.690292	5.462593	4.498367
C	-1.842885	14.139592	-1.845289
H	-2.316191	13.895939	-2.785919
C	-2.249052	13.486847	-0.665061
H	-3.041630	12.752109	-0.730499
C	-0.268614	15.388389	-0.708440
H	0.506629	16.140398	-0.745591
C	-0.608672	14.787674	0.519292
H	-0.088259	15.110653	1.410928
C	-1.628375	13.803380	0.570176
C	-2.015642	13.175734	1.793056
C	-1.779588	12.925133	4.034158
C	-3.371959	11.752404	2.925630
C	-4.375006	10.735813	2.925593
C	-4.777173	10.078980	4.116176
H	-4.343714	10.332312	5.074745
C	-5.014997	10.311035	1.734000
H	-4.778128	10.754305	0.775365
C	-5.993343	9.298629	1.782993
H	-6.485502	8.981637	0.874567
C	-5.761920	9.072666	4.068114
H	-6.065410	8.573532	4.976863
C	-1.066594	13.188443	5.242756
C	0.051095	14.060352	5.285378
H	0.406971	14.569406	4.398834
C	0.706224	14.304086	6.509310
H	1.546268	14.982796	6.545697
C	-0.718346	12.889933	7.647018
H	-1.007559	12.437217	8.584541
C	-1.438562	12.592114	6.474444
H	-2.280525	11.915356	6.546537
C	8.885274	8.096628	2.499622
H	9.403491	7.323485	1.950490
C	8.636404	10.343513	2.959136
H	8.954496	11.359823	2.775314
C	7.593156	10.091618	3.870913
H	7.137985	10.933592	4.375708
C	7.169269	8.760528	4.114229
C	7.851936	7.749116	3.392356
H	7.603661	6.702468	3.511420
C	6.120741	8.458846	5.034955
C	4.568500	9.156951	6.533868
C	4.819201	6.942800	6.109829
N	2.627445	12.363776	8.614831
C	3.904041	10.223689	7.212464
C	4.268968	11.578979	7.011027
H	5.063971	11.857839	6.331516
C	3.613238	12.599436	7.725735
H	3.905393	13.629819	7.580427
C	2.854749	9.994201	8.138304
H	2.513119	8.993084	8.365826
C	2.254874	11.081253	8.806887
H	1.465616	10.905167	9.524055
C	4.445407	5.583936	6.347822
C	3.446636	5.227186	7.290157
H	2.931211	5.975340	7.878169
C	3.120036	3.870307	7.493952
H	2.360655	3.600450	8.214021
C	4.662974	3.181847	5.922263
H	5.132604	2.364467	5.393096
C	5.057739	4.507591	5.656624
H	5.838019	4.673489	4.924581
C	0.611300	13.170119	-4.282597
H	-0.341083	13.154950	-4.793429
C	2.230271	14.392429	-3.190441
H	2.569325	15.353374	-2.830903
C	3.030510	13.249668	-2.992172
H	3.977578	13.364660	-2.480847
C	1.339939	11.975075	-4.130124
H	0.921183	11.064458	-4.539043
C	2.592514	11.987895	-3.466432
C	3.367443	10.801143	-3.294936
C	5.237434	9.760204	-2.540041
C	3.651845	8.578754	-3.657175
C	6.491603	9.799034	-1.859015
C	7.002217	10.990204	-1.284198
H	6.462323	11.926025	-1.348280
C	8.245121	10.973514	-0.619010
H	8.634261	11.882920	-0.183786
C	8.528143	8.722053	-1.031385
H	9.139556	7.837568	-0.922878
C	7.299611	8.642503	-1.714026
H	7.000844	7.684114	-2.119013
C	3.186016	7.342951	-4.199294
C	3.941797	6.147487	-4.099244
H	4.906202	6.124108	-3.607866

C	3.448983	4.953962	-4.661410
H	4.030646	4.045802	-4.588025
C	1.946355	7.239664	-4.879299
H	1.302617	8.098617	-5.019279
C	1.535612	6.000747	-5.409578
H	0.592253	5.927215	-5.931759
C	-0.864453	1.198696	-6.049093
H	-1.142902	2.234049	-5.834165
H	-1.429365	0.900430	-6.941038
H	-1.240080	0.578500	-5.225711
C	1.193288	0.126996	-5.261902
H	1.950174	0.623307	-4.648278
H	0.447211	-0.302146	-4.581506
H	1.689942	-0.721774	-5.748486
C	0.949194	0.717490	-7.630694
H	1.504891	-0.228530	-7.658297
H	0.041098	0.510553	-8.211403
C	1.773695	1.733418	-8.459238
H	2.657351	1.192558	-8.821523
H	1.192513	1.935286	-9.368202
C	3.647672	3.133907	-7.717217
H	3.977610	3.106800	-6.674953
H	4.175526	2.323701	-8.235556
H	4.017263	4.069795	-8.154999
C	1.565876	4.179141	-8.474464
H	2.310377	4.926945	-8.775013
H	1.014946	3.910505	-9.384439
H	0.852362	4.675116	-7.810403
N	0.351779	15.571791	10.176557
N	2.690375	14.136429	11.164977
C	0.388993	16.655465	9.158430
H	-0.596338	16.869460	8.725951
H	0.763160	17.607712	9.554811
H	1.053479	16.374426	8.336429
C	-1.037185	15.079357	10.376179
H	-1.764188	15.569071	9.716132
H	-1.084662	14.005225	10.178300
H	-1.404025	15.226048	11.399716
C	0.943353	16.033500	11.466485
H	0.194779	16.011373	12.268875
H	1.240435	17.088451	11.407279
C	2.173503	15.259679	12.000373
H	2.956394	16.008304	12.176967
H	1.907222	14.909368	13.005837
C	4.086619	14.400805	10.726352
H	4.775742	13.585052	10.978471
H	4.118701	14.533896	9.641734
H	4.513012	15.310375	11.167621
C	2.599021	12.844232	11.895625
H	3.561881	12.322526	11.963396
H	2.237551	12.954835	12.925729
H	1.898179	12.176244	11.386687
N	-9.426119	8.296357	3.323827
N	-9.086106	5.435634	2.873114
N	0.608347	1.095099	-6.227490
N	2.167299	3.006046	-7.786297
N	12.349208	9.782561	2.099370
N	12.065810	10.313378	-0.757046
N	0.041275	2.732174	1.178869
N	-1.712330	3.278520	2.737381
N	-1.982751	3.766843	0.394637
N	-6.032813	5.895987	2.497799
N	1.847515	1.089595	5.594387
N	-0.875524	15.078634	-1.871736
N	-3.017351	12.271447	1.727875
N	-1.315696	13.504554	2.903276
N	-2.835088	12.088344	4.120370
N	0.330272	13.736940	7.674282
N	-6.368495	8.688952	2.926431
N	9.280249	9.368716	2.285635
N	5.554449	9.503247	5.678550
N	4.148346	7.897100	6.796035
N	5.791589	7.156953	5.196246
N	3.715981	2.860200	6.827042
N	1.040837	14.364821	-3.827216
N	4.532886	10.912976	-2.616760
N	4.846999	8.562490	-3.029681
N	2.849171	9.656419	-3.793875
N	2.269914	4.873233	-5.311550
N	9.003062	9.863968	-0.494494
Pd	3.196980	0.846173	7.189008
Pd	10.670370	9.833204	0.780989
Pd	-7.725132	7.076410	2.902675
Pd	1.436852	3.007930	-5.783089
Pd	-0.286232	15.981883	-3.679929
Pd	1.498505	13.946851	9.405537
C	0.729538	5.216208	4.930017
C	1.568695	6.178721	4.110329

C	0.993048	6.252429	2.706465
C	-0.382168	6.535234	2.694101
C	-0.970507	6.682606	4.084787
C	-0.581206	5.476907	4.921645
H	2.769618	5.961535	1.527701
H	1.191283	4.420797	5.502723
H	2.635892	5.953627	4.104235
C	1.703759	6.160590	1.514916
C	-1.041026	6.764663	1.491626
H	-2.041240	6.887237	4.066602
H	-1.322601	4.927272	5.489496
C	-0.331689	6.658091	0.289120
C	1.029509	6.349087	0.302135
H	-2.091304	7.040229	1.488739
H	1.576285	6.274224	-0.632667
H	-0.844878	6.825686	-0.651307
C	-0.318624	9.120475	3.840698
C	-0.201395	7.895599	4.726150
C	1.305718	7.589483	4.766885
C	1.959764	8.701676	3.958158
H	-0.622859	8.111680	5.709113
H	1.705204	7.594933	5.782348
C	1.147976	10.638874	2.517051
C	2.255554	11.624846	2.909626
C	1.369202	10.056733	1.112393
H	0.188068	11.165852	2.516747
C	2.330913	12.756884	1.874815
H	3.210252	11.094641	2.959014
H	2.055098	12.028431	3.909830
C	1.444942	11.189257	0.080189
H	2.299491	9.477186	1.116257
H	0.556683	9.364670	0.872868
C	2.525371	12.214759	0.450363
H	3.141547	13.444861	2.128222
H	1.401949	13.340228	1.917657
H	1.626829	10.767904	-0.914461
H	0.467745	11.689676	0.026634
H	2.522915	13.047189	-0.261826
H	3.512878	11.741604	0.375209
N	0.961736	9.558825	3.498580
O	3.148286	8.805510	3.721612
O	-1.349403	9.621938	3.439782

Table5_1a_TSiv_reactant_0wat

Property	Value
Charge	0
Electronic Energy, BS1 (a.u.)	-980.278150
Thermal and entropic correction, BS1 (a.u.)	2.695665
Electronic Energy, BS2 (a.u.)	-980.613503
Number of Imaginary Frequencies	0
Imaginary frequencies (cm-1)	None

Molecular Geometry in Cartesian Coordinates

C	-9.157439	8.951766	4.604429
H	-9.987561	8.775738	5.299857
H	-8.265879	8.518865	5.066449
H	-9.015367	10.038987	4.563855
C	-9.398751	9.389012	2.205409
H	-9.207303	10.396758	2.594909
H	-8.632962	9.177086	1.454326
H	-10.356005	9.443408	1.671980
C	-10.635251	7.542294	3.243626
H	-11.319531	7.902558	2.464751
H	-11.197870	7.676056	4.176500
C	-10.496568	6.014202	3.037261
H	-11.145247	5.756072	2.190394
H	-10.982704	5.540599	3.899878
C	-9.002356	4.796574	1.519574
H	-8.309775	5.346197	0.876407
H	-8.632531	3.766457	1.597530
H	-9.954546	4.739162	0.977543
C	-8.749392	4.546208	3.943479
H	-7.920989	4.968154	4.520003
H	-9.568494	4.367946	4.651371
H	-8.432318	3.558762	3.585384
N	2.658859	-1.133366	7.374967
N	4.636784	0.527367	8.729324
C	1.229921	-1.115858	7.785078
H	0.898710	-0.084394	7.934683
H	0.565117	-1.572023	7.040673
H	1.045520	-1.645176	8.728217
C	2.825613	-1.803535	6.057515

H	3.298856	-1.121109	5.346077
H	3.459009	-2.698186	6.105161
H	1.872957	-2.125897	5.619005
C	3.505496	-1.788021	8.414953
H	2.881343	-2.232619	9.200738
H	4.054937	-2.640637	7.995726
C	4.549829	-0.904452	9.140016
H	5.518456	-1.406805	9.021720
H	4.333732	-0.989691	10.212753
C	5.998527	0.852061	8.227102
H	6.447854	1.712999	8.737625
H	6.709053	0.023466	8.339423
H	5.958597	1.087171	7.160171
C	4.253793	1.427101	9.849010
H	3.338371	1.968288	9.594285
H	4.051878	0.889891	10.784183
H	5.024819	2.172204	10.082005
N	-1.670533	17.636928	-3.488498
N	0.305041	16.921137	-5.514213
C	-3.035313	17.049103	-3.431040
H	-3.531855	17.214543	-2.466675
H	-3.710451	17.450461	-4.197110
H	-2.984221	15.969049	-3.593635
C	-1.342423	18.344514	-2.222579
H	-2.140854	18.272180	-1.473589
H	-0.437366	17.918332	-1.780745
H	-1.150034	19.415434	-2.363784
C	-1.531532	18.549475	-4.660980
H	-1.282666	19.568171	-4.336908
H	-2.489534	18.662222	-5.184525
C	-0.487871	18.165593	-5.739185
H	-1.030656	18.116341	-6.691918
H	0.172942	19.034532	-5.852744
C	1.755657	17.228096	-5.400469
H	2.371102	16.650190	-6.101248
H	1.989413	18.283988	-5.585642
H	2.107190	17.000800	-4.390008
C	0.054924	15.928622	-6.593175
H	0.975670	15.590513	-7.084763
H	-0.439718	15.044744	-6.180699
H	-0.595799	16.311262	-7.389309
C	12.378824	8.509137	2.650206
H	12.193202	8.497580	3.731519
H	11.627196	7.867931	2.181354
H	13.349033	8.019520	2.499539
C	12.053134	10.891857	3.118665
H	11.916075	10.451120	4.114043
H	12.859319	11.629556	3.217716
H	11.144646	11.450699	2.879075
C	13.566871	10.194430	1.317902
H	14.304475	9.389179	1.427957
H	14.065024	11.074061	1.745329
C	13.436278	10.462184	-0.201867
H	13.874258	11.452388	-0.381448
H	14.129183	9.770079	-0.697312
C	11.665505	11.662854	-1.405194
H	11.364393	11.568431	-2.455970
H	10.816368	12.081299	-0.857490
H	12.460071	12.419036	-1.380635
C	12.007679	9.269119	-1.802782
H	11.648388	9.606065	-2.783236
H	12.977663	8.787945	-1.979906
H	11.327925	8.485813	-1.455339
N	0.726534	3.134614	-3.876825
C	1.454789	2.648156	-2.851196
H	2.430157	2.247827	-3.089606
C	1.000128	2.642808	-1.517694
H	1.644554	2.222674	-0.756144
C	-0.278963	3.167765	-1.202170
C	-1.025752	3.683278	-2.291984
H	-2.013888	4.103474	-2.154906
C	-0.488355	3.647346	-3.593906
H	-1.058229	4.042918	-4.422862
C	-0.782908	3.180429	0.133929
C	-0.496619	2.760988	2.345440
C	0.313991	2.264048	3.410596
C	1.610116	1.735437	3.184156
H	2.033596	1.676248	2.189777
C	2.375270	1.258087	4.266129
H	3.359647	0.847733	4.088617
C	0.710221	1.772124	5.775418
H	0.368558	1.776610	6.800008
C	-0.128385	2.266797	4.756893
H	-1.107194	2.640243	5.028931
C	-2.396820	3.765643	1.618617
C	-3.622529	4.444516	1.897847
C	-4.110749	4.610541	3.219007
H	-3.580625	4.205031	4.071159

C	-4.412802	5.014630	0.867601
H	-4.129855	4.937059	-0.174296
C	-5.604008	5.694464	1.186184
H	-6.204892	6.124743	0.397414
C	-5.316345	5.307212	3.442091
H	-5.691452	5.428074	4.448099
C	-1.846411	14.171911	-1.847394
H	-2.334126	13.945491	-2.784901
C	-2.250131	13.513828	-0.669816
H	-3.056580	12.794258	-0.734585
C	-0.238148	15.381695	-0.715432
H	0.549746	16.120311	-0.753965
C	-0.573111	14.772854	0.510289
H	-0.031373	15.070653	1.398930
C	-1.612096	13.808575	0.561986
C	-2.004781	13.181431	1.783274
C	-1.795201	12.948218	4.027962
C	-3.382503	11.777526	2.912806
C	-4.366570	10.742253	2.904061
C	-4.772884	10.083964	4.092217
H	-4.360013	10.354875	5.055017
C	-4.975316	10.293578	1.704483
H	-4.730432	10.734151	0.746505
C	-5.927196	9.255737	1.743037
H	-6.393405	8.918151	0.828165
C	-5.729142	9.051012	4.033840
H	-6.033468	8.548330	4.940073
C	-1.090442	13.215362	5.240691
C	0.032336	14.080446	5.284283
H	0.396960	14.581005	4.396440
C	0.681873	14.328461	6.509829
H	1.526477	15.001858	6.545793
C	-0.756437	12.929854	7.648962
H	-1.053294	12.485047	8.587718
C	-1.473472	12.629977	6.474213
H	-2.319628	11.958405	6.544051
C	8.887155	8.175699	2.464738
H	9.408517	7.403363	1.917651
C	8.593526	10.426491	2.881112
H	8.883924	11.445370	2.667281
C	7.579354	10.169623	3.824590
H	7.114957	11.010234	4.323489
C	7.196495	8.833960	4.108602
C	7.886946	7.823472	3.391964
H	7.667158	6.773765	3.538338
C	6.177257	8.525411	5.059649
C	4.636427	9.207360	6.577601
C	4.921561	6.996356	6.168346
N	2.607626	12.399870	8.593523
C	3.947464	10.268317	7.240524
C	4.266100	11.629214	7.001656
H	5.045898	11.916609	6.308252
C	3.580052	12.645794	7.692713
H	3.834063	13.681451	7.515995
C	2.913876	10.028090	8.181261
H	2.607784	9.023094	8.439415
C	2.280058	11.111488	8.823655
H	1.500044	10.927787	9.543594
C	4.560343	5.633781	6.401019
C	3.580223	5.263236	7.357051
H	3.072839	6.003835	7.961602
C	3.266586	3.902534	7.553957
H	2.524183	3.621234	8.287033
C	4.788643	3.237347	5.952200
H	5.257123	2.427547	5.410527
C	5.167231	4.567769	5.689139
H	5.933410	4.744743	4.944872
C	0.603029	13.211299	-4.324435
H	-0.357051	13.198232	-4.820712
C	2.230713	14.426776	-3.236610
H	2.569210	15.384635	-2.868323
C	3.040845	13.286602	-3.068801
H	3.997502	13.402313	-2.575777
C	1.343128	12.018932	-4.204721
H	0.926108	11.111221	-4.622049
C	2.606120	12.030216	-3.561081
C	3.395822	10.848528	-3.425561
C	5.268705	9.809661	-2.673147
C	3.689783	8.628500	-3.800161
C	6.513880	9.852370	-1.976062
C	7.004671	11.043500	-1.383720
H	6.457155	11.975052	-1.446233
C	8.237772	11.033390	-0.701654
H	8.612160	11.942887	-0.253878
C	8.548685	8.788122	-1.127882
H	9.167226	7.908844	-1.018259
C	7.330290	8.702202	-1.828849
H	7.044962	7.744967	-2.246341

C	3.223326	7.391277	-4.339595
C	3.968222	6.191018	-4.218972
H	4.926648	6.165911	-3.716181
C	3.469285	4.993816	-4.768552
H	4.042789	4.082336	-4.677000
C	1.989950	7.288894	-5.031035
H	1.351731	8.150181	-5.182398
C	1.571478	6.046050	-5.545958
H	0.630585	5.973989	-6.072844
C	-0.883100	1.254038	-6.071084
H	-1.149628	2.297347	-5.880852
H	-1.458461	0.937885	-6.950111
H	-1.257279	0.658802	-5.228867
C	1.169695	0.175289	-5.280883
H	1.937048	0.674724	-4.683056
H	0.424809	-0.234039	-4.587024
H	1.653920	-0.687174	-5.755950
C	0.912104	0.727089	-7.658071
H	1.439471	-0.235311	-7.674411
H	-0.002870	0.540134	-8.234707
C	1.766054	1.706650	-8.500291
H	2.636937	1.136872	-8.848811
H	1.194607	1.907103	-9.415647
C	3.672521	3.065132	-7.762583
H	3.987988	3.046388	-6.715541
H	4.182212	2.230513	-8.259931
H	4.075164	3.981898	-8.211324
C	1.631961	4.156137	-8.567637
H	2.401764	4.875929	-8.872958
H	1.084615	3.885238	-9.479173
H	0.924559	4.685704	-7.923246
N	0.322249	15.582220	10.194797
N	2.675351	14.154407	11.156754
C	0.325473	16.664288	9.174212
H	-0.669933	16.860528	8.756697
H	0.689435	17.623607	9.563021
H	0.982064	16.393233	8.342521
C	-1.055599	15.071987	10.423558
H	-1.803554	15.556765	9.783514
H	-1.093926	13.998365	10.220447
H	-1.400161	15.207808	11.456346
C	0.934315	16.054538	11.471223
H	0.199178	16.036176	12.286025
H	1.227746	17.109762	11.400047
C	2.174775	15.286816	11.989695
H	2.960847	16.036915	12.144651
H	1.926649	14.946436	13.003225
C	4.072078	14.403040	10.710117
H	4.754027	13.581120	10.961710
H	4.100459	14.532935	9.624939
H	4.510255	15.309326	11.146647
C	2.574193	12.866498	11.892961
H	3.530610	12.332362	11.953529
H	2.223159	12.985829	12.925614
H	1.859716	12.206650	11.392510
N	-9.378078	8.345299	3.264959
N	-9.120282	5.471411	2.840198
N	0.586945	1.132094	-6.258878
N	2.190115	2.980347	-7.848206
N	12.315795	9.875057	2.065835
N	12.072454	10.366234	-0.800728
N	0.027995	2.699045	1.101307
N	-1.696990	3.291152	2.674582
N	-2.007329	3.718921	0.324547
N	-6.057416	5.838956	2.447732
N	1.940796	1.270668	5.543111
N	-0.864631	15.095400	-1.874952
N	-3.015555	12.287671	1.715433
N	-1.307783	13.506090	2.895902
N	-2.870485	12.135610	4.112380
N	0.297883	13.770420	7.676265
N	-6.304557	8.643445	2.884147
N	9.244084	9.451795	2.213332
N	5.595457	9.566004	5.695261
N	4.258591	7.941383	6.874885
N	5.883426	7.219775	5.246157
N	3.859841	2.902109	6.870168
N	1.032440	14.401326	-3.855869
N	4.565650	10.962009	-2.756132
N	4.880089	8.610801	-3.161154
N	2.889064	9.707686	-3.944750
N	2.293758	4.912780	-5.425560
N	9.004609	9.930558	-0.575028
Pd	3.268791	0.896973	7.129369
Pd	10.654258	9.910589	0.723819
Pd	-7.710468	7.071559	2.863285
Pd	1.436685	3.045710	-5.855133
Pd	-0.292217	16.017284	-3.678839

Pd	1.473453	13.970383	9.403815
C	0.327900	5.309858	5.595654
C	1.181601	5.593301	4.543525
C	0.654855	5.974598	3.280692
C	-0.700687	6.428936	3.228381
C	-1.453695	6.432061	4.434957
C	-1.012950	5.725759	5.536579
H	2.412462	5.493896	2.116378
H	0.714005	4.896722	6.528928
H	2.250726	5.434650	4.632653
C	1.386103	5.842208	2.075281
C	-1.264232	6.788390	1.976492
H	-2.410940	6.946232	4.451278
H	-1.648788	5.624126	6.419964
C	-0.552052	6.588576	0.815617
C	0.776312	6.094919	0.866089
H	-2.270274	7.185188	1.947022
H	1.326202	5.940981	-0.059206
H	-1.004003	6.810827	-0.141250
C	-0.357711	9.190783	4.035533
C	-0.144514	8.406048	5.283876
C	1.112577	8.045669	5.362623
C	1.857797	8.626121	4.214793
H	-0.970844	8.330940	6.037052
H	1.660968	7.567548	6.198536
C	1.177459	10.193421	2.316985
C	1.950921	11.443560	2.774299
C	1.900308	9.455818	1.188439
H	0.184870	10.498618	1.967515
C	2.214429	12.373912	1.587495
H	2.901569	11.125598	3.222446
H	1.380951	11.957099	3.559536
C	2.189488	10.398150	0.012937
H	2.835584	9.048539	1.580903
H	1.289143	8.613169	0.862958
C	2.967131	11.640476	0.469122
H	2.770126	13.258056	1.911377
H	1.251140	12.728445	1.193877
H	2.748312	9.851487	-0.754878
H	1.242316	10.711902	-0.447310
H	3.141635	12.316918	-0.374211
H	3.956008	11.338284	0.837885
N	0.904186	9.312234	3.449764
O	3.058589	8.607747	4.002921
O	-1.380883	9.671780	3.590772

Table5_1a_TSiv_TSi-ii_0wat

Property	Value
Charge	0
Electronic Energy, BS1 (a.u.)	-980.232763
Thermal and entropic correction, BS1 (a.u.)	2.697614
Electronic Energy, BS2 (a.u.)	-980.564651
Number of Imaginary Frequencies	0
Imaginary frequencies (cm-1)	None

Molecular Geometry in Cartesian Coordinates

C	-8.054202	2.837918	5.907052
C	-8.763269	3.173007	4.709954
C	-8.036679	3.903006	3.678558
C	-7.077273	4.830434	4.145864
C	-6.943036	4.925545	5.590992
C	-7.121254	3.732356	6.358740
H	-9.047001	3.110531	1.940508
H	-8.387562	2.012196	6.526829
H	-9.576044	2.525957	4.390246
C	-8.297350	3.806978	2.302015
C	-6.401580	5.656006	3.234209
H	-6.285548	5.694393	5.984708
H	-6.681052	3.641881	7.345241
C	-6.642725	5.528519	1.873163
C	-7.591323	4.602610	1.408645
H	-5.681978	6.383926	3.600045
H	-7.785572	4.522177	0.344375
H	-6.106934	6.152858	1.167774
C	-10.423531	4.267398	6.692738
C	-9.820365	4.858203	5.459759
C	-8.856047	5.805756	5.882587
C	-8.858225	5.822121	7.373897
H	-10.429141	4.966129	4.572872
H	-8.621243	6.712897	5.344364
C	-9.964075	4.410238	9.177153
C	-8.684755	3.824041	9.795264

C	-10.508156	5.565465	10.031445
H	-10.724806	3.625010	9.114065
C	-8.940005	3.371396	11.241422
H	-7.905333	4.593328	9.777036
H	-8.336450	2.985726	9.182843
C	-10.775854	5.101686	11.470011
H	-9.768311	6.373040	10.029864
H	-11.419746	5.951148	9.553509
C	-9.502425	4.515141	12.097846
H	-8.009650	2.991671	11.674763
H	-9.650757	2.534505	11.241758
H	-11.145110	5.938691	12.072713
H	-11.565094	4.337765	11.468328
H	-9.714620	4.160200	13.111046
H	-8.748261	5.307763	12.190430
N	-9.755922	4.835623	7.785910
O	-8.186027	6.515516	8.119127
O	-11.296771	3.423272	6.776490
C	-12.397189	-3.786167	7.641591
H	-12.247472	-4.455693	8.497815
H	-11.778785	-2.902207	7.822021
H	-13.446522	-3.467751	7.679469
C	-13.197567	-4.464999	5.427686
H	-14.099284	-4.012821	5.859221
H	-12.976329	-3.932066	4.499173
H	-13.473445	-5.488496	5.144519
C	-11.442359	-5.764566	6.544455
H	-12.021476	-6.525523	6.005819
H	-11.517257	-6.067422	7.596767
C	-9.962792	-5.969062	6.136768
H	-9.946870	-6.805611	5.426463
H	-9.451899	-6.363058	7.024707
C	-8.722725	-5.069544	4.220754
H	-9.222386	-4.424503	3.492450
H	-7.642894	-4.900204	4.124903
H	-8.899987	-6.103181	3.897911
C	-8.129692	-4.377307	6.495585
H	-8.343968	-3.393124	6.920719
H	-7.989311	-5.061131	7.342136
H	-7.159873	-4.314331	5.986435
N	0.179559	5.664611	6.792555
N	-0.770799	8.283138	7.652426
C	-0.153912	4.442699	7.570783
H	-1.214074	4.451984	7.840312
H	0.037922	3.515853	7.015568
H	0.409005	4.362439	8.509091
C	0.719419	5.311118	5.452173
H	0.110794	5.769874	4.668307
H	1.747779	5.659982	5.295004
H	0.731632	4.229481	5.268656
C	1.139814	6.529991	7.538568
H	1.492957	6.025597	8.447198
H	2.053633	6.698225	6.954333
C	0.639861	7.926566	7.982547
H	1.340644	8.659103	7.561980
H	0.811307	7.983455	9.065210
C	-0.825722	9.527722	6.839533
H	-1.482464	10.292114	7.273466
H	0.154924	10.003333	6.712437
H	-1.199068	9.305912	5.836022
C	-1.588788	8.416204	8.886536
H	-2.352800	7.634149	8.911887
H	-1.001066	8.309911	9.806954
H	-2.101391	9.383736	8.957657
N	-20.156122	3.698349	-1.937640
N	-18.890063	4.953355	-4.248149
C	-19.997226	2.254626	-1.619151
H	-20.305131	2.005983	-0.595795
H	-20.575467	1.599662	-2.282804
H	-18.948349	1.963661	-1.726220
C	-20.726312	4.438725	-0.780679
H	-20.895561	3.798993	0.094586
H	-20.044225	5.237338	-0.475493
H	-21.689071	4.914711	-1.004897
C	-20.993703	3.887729	-3.158108
H	-21.886300	4.484886	-2.931723
H	-21.395634	2.928202	-3.507911
C	-20.321167	4.552184	-4.385171
H	-20.452884	3.856484	-5.223764
H	-20.945867	5.414299	-4.651890
C	-18.730559	6.420871	-4.428749
H	-17.991776	6.679290	-5.197740
H	-19.661988	6.924190	-4.716434
H	-18.402882	6.878964	-3.491441
C	-18.033054	4.204019	-5.204924
H	-17.422493	4.860966	-5.837048
H	-17.350449	3.546803	-4.658898
H	-18.606147	3.565152	-5.888331

C	-7.382549	16.150281	-0.041485
H	-7.298047	16.280338	1.044779
H	-6.972104	15.166798	-0.287175
H	-6.712748	16.892724	-0.493137
C	-9.716760	16.547666	0.589578
H	-9.212641	16.604375	1.562380
H	-10.252798	17.496838	0.465602
H	-10.477026	15.764736	0.659145
C	-8.900145	17.278817	-1.606381
H	-7.944894	17.797942	-1.756919
H	-9.594276	18.076570	-1.312657
C	-9.358788	16.793051	-3.003653
H	-10.242329	17.389395	-3.264989
H	-8.591461	17.127336	-3.713567
C	-11.049644	15.101205	-3.556037
H	-11.148571	14.507897	-4.473756
H	-11.571249	14.562766	-2.759736
H	-11.609538	16.029069	-3.727723
C	-8.703798	14.726156	-4.155443
H	-9.224922	14.196932	-4.963030
H	-8.048247	15.459923	-4.640763
H	-8.051184	14.003500	-3.657464
N	-5.429377	2.142038	-3.411514
C	-4.669970	2.942231	-2.634721
H	-4.024942	3.647572	-3.139037
C	-4.683730	2.888245	-1.226436
H	-4.032186	3.556101	-0.677677
C	-5.529182	1.964533	-0.561453
C	-6.330442	1.142747	-1.394347
H	-7.004571	0.402103	-0.983572
C	-6.246424	1.264592	-2.794628
H	-6.852359	0.631586	-3.427478
C	-5.565220	1.858279	0.861726
C	-4.819381	2.504658	2.905241
C	-4.030641	3.385630	3.706821
C	-3.222178	4.399579	3.132869
H	-3.148327	4.526628	2.060579
C	-2.494097	5.274317	3.962353
H	-1.877553	6.044839	3.521593
C	-3.272734	4.241932	5.872121
H	-3.279596	4.192371	6.951103
C	-4.029964	3.319018	5.122797
H	-4.605296	2.569865	5.651077
C	-6.399622	0.884224	2.733438
C	-7.316040	-0.028078	3.340393
C	-7.385312	-0.206145	4.744880
H	-6.728818	0.336156	5.412982
C	-8.214133	-0.810926	2.570278
H	-8.234889	-0.755533	1.489353
C	-9.102713	-1.698373	3.207262
H	-9.781528	-2.297830	2.616831
C	-8.314473	-1.112367	5.295612
H	-8.369838	-1.242140	6.366357
C	-16.827787	2.892734	-0.116089
H	-16.841914	2.142428	-0.893481
C	-16.127461	2.645386	1.080467
H	-15.618414	1.696732	1.192582
C	-17.485134	4.982273	0.605521
H	-18.024565	5.896432	0.402794
C	-16.811975	4.825271	1.832610
H	-16.854605	5.633697	2.551190
C	-16.105871	3.626244	2.104013
C	-15.415605	3.417751	3.335888
C	-14.804263	4.148917	5.395775
C	-14.155589	2.094261	4.682308
C	-13.404532	0.895138	4.877969
C	-12.712786	0.626213	6.085763
H	-12.741707	1.318140	6.917707
C	-13.300052	-0.095909	3.869061
H	-13.794853	0.013206	2.912372
C	-12.540198	-1.258698	4.099148
H	-12.464297	-2.011906	3.327712
C	-11.974597	-0.566021	6.225226
H	-11.449478	-0.768194	7.146381
C	-14.773131	5.184469	6.379087
C	-15.382356	6.445662	6.156574
H	-15.897850	6.671847	5.231902
C	-15.323165	7.442289	7.149455
H	-15.786771	8.402725	6.973704
C	-14.134343	6.068526	8.570768
H	-13.662043	5.931400	9.532007
C	-14.130801	5.016817	7.632185
H	-13.643149	4.086236	7.894999
C	-7.839905	12.748691	0.793357
H	-7.024892	12.927138	0.106662
C	-10.046574	13.117205	1.356386
H	-10.988884	13.589141	1.117924
C	-9.938127	12.315165	2.508749

H	-10.812240	12.194799	3.135754
C	-8.703566	11.694157	2.826131
C	-7.637101	11.931232	1.922526
H	-6.655483	11.503489	2.079217
C	-8.543473	10.884494	3.990845
C	-9.398135	10.006829	5.902806
C	-7.226140	9.633249	5.351716
N	-12.758176	9.330478	8.477014
C	-10.513652	9.790652	6.768493
C	-11.807679	10.282824	6.459533
H	-11.999505	10.851565	5.558511
C	-12.885786	10.025391	7.328482
H	-13.872033	10.396202	7.087387
C	-10.398027	9.067001	7.982128
H	-9.445709	8.679180	8.318370
C	-11.533146	8.861325	8.791680
H	-11.440374	8.311660	9.716478
C	-5.966902	9.015202	5.623330
C	-5.724482	8.295065	6.819823
H	-6.479503	8.210859	7.590143
C	-4.472689	7.685731	7.037881
H	-4.298222	7.133266	7.949780
C	-3.662165	8.451068	5.017595
H	-2.834546	8.510296	4.324596
C	-4.882192	9.086331	4.712252
H	-4.961667	9.626736	3.777493
C	-15.278068	4.407657	-3.038889
H	-15.545161	3.385675	-3.266509
C	-15.945482	6.491695	-2.303917
H	-16.743281	7.127224	-1.946775
C	-14.642251	7.009222	-2.435019
H	-14.471344	8.046291	-2.176122
C	-13.946472	4.836600	-3.200718
H	-13.215179	4.123273	-3.558581
C	-13.593051	6.176805	-2.901296
C	-12.259954	6.660840	-3.063640
C	-10.757822	8.357905	-2.944149
C	-10.101921	6.310695	-3.671650
C	-10.449963	9.717950	-2.638853
C	-11.432848	10.622213	-2.162768
H	-12.459296	10.314584	-2.009252
C	-11.083831	11.958228	-1.885044
H	-11.836331	12.645926	-1.525983
C	-8.887510	11.595234	-2.490152
H	-7.890242	11.993075	-2.610999
C	-9.142993	10.244580	-2.795761
H	-8.323808	9.633394	-3.152088
C	-9.078017	5.441580	-4.156174
C	-7.750033	5.890965	-4.366779
H	-7.459649	6.915252	-4.170603
C	-6.773504	4.999972	-4.852291
H	-5.763187	5.347781	-5.013168
C	-9.328863	4.079874	-4.460348
H	-10.311851	3.643341	-4.336785
C	-8.287438	3.263450	-4.943252
H	-8.483797	2.226776	-5.176885
C	-4.223603	-0.290200	-5.226562
H	-5.253215	-0.248191	-4.860302
H	-4.175006	-1.128469	-5.932752
H	-3.583652	-0.566412	-4.379238
C	-2.648179	1.585241	-5.167381
H	-2.885059	2.573128	-4.762377
H	-2.285528	0.966454	-4.337083
H	-1.796469	1.719553	-5.845908
C	-3.616487	0.865004	-7.304518
H	-2.580878	1.118343	-7.565119
H	-3.721481	-0.182501	-7.614655
C	-4.518889	1.694256	-8.251318
H	-3.842661	2.276518	-8.890291
H	-4.986892	0.979276	-8.939947
C	-5.277718	4.006688	-7.930129
H	-5.027000	4.546510	-7.012492
H	-4.436383	4.149616	-8.619501
H	-6.137830	4.513356	-8.385629
C	-6.914462	2.188005	-8.049216
H	-7.480947	3.023253	-8.479721
H	-6.922431	1.390342	-8.802668
H	-7.481722	1.815478	-7.191765
N	-16.156880	8.416817	10.834591
N	-14.150723	10.524768	10.984744
C	-17.323238	8.373572	9.912023
H	-17.765921	7.373016	9.829761
H	-18.137393	9.045176	10.212209
H	-17.019380	8.681365	8.907879
C	-15.919646	7.086422	11.454660
H	-16.617615	6.318315	11.098281
H	-14.906201	6.743931	11.224983
H	-16.007808	7.098446	12.548317

C	-16.346176	9.466977	11.877807
H	-16.394971	9.017537	12.877916
H	-17.316516	9.966659	11.761978
C	-15.280916	10.587808	11.957036
H	-15.821464	11.539140	11.871956
H	-14.907309	10.583983	12.989097
C	-14.100194	11.756171	10.151626
H	-13.119960	12.248570	10.175915
H	-14.320437	11.514254	9.108169
H	-14.830625	12.514958	10.459144
C	-12.858122	10.303357	11.684881
H	-12.096553	11.048254	11.421812
H	-12.950673	10.330704	12.777690
H	-12.459312	9.317515	11.428012
N	-12.025861	-4.406257	6.342152
N	-9.234286	-4.787394	5.588999
N	-3.845941	1.012654	-5.837207
N	-5.544127	2.576626	-7.620798
N	-8.783505	16.252044	-0.529944
N	-9.633293	15.335081	-3.167091
N	-4.748882	2.672940	1.565020
N	-5.608319	1.614529	3.552354
N	-6.434227	0.967311	1.385706
N	-9.155937	-1.853779	4.545228
N	-2.512828	5.204024	5.309170
N	-17.502566	4.035892	-0.355671
N	-14.767884	2.241382	3.486417
N	-15.449173	4.422127	4.239553
N	-14.168968	2.993448	5.691049
N	-14.717682	7.263424	8.341251
N	-11.889034	-1.499307	5.255297
N	-9.018961	13.340561	0.511918
N	-9.624345	10.734465	4.787458
N	-8.221539	9.433894	6.245949
N	-7.329632	10.324525	4.195002
N	-3.452964	7.761624	6.157711
N	-16.265909	5.216992	-2.605738
N	-12.034653	7.957421	-2.754462
N	-9.745589	7.585049	-3.397546
N	-11.336663	5.783319	-3.517660
N	-7.029314	3.708069	-5.143074
N	-9.836558	12.445545	-2.048238
Pd	-1.645740	6.725188	6.479431
Pd	-9.319327	14.338510	-1.309902
Pd	-10.573086	-3.134265	5.429719
Pd	-5.461682	2.361934	-5.501420
Pd	-18.203814	4.482494	-2.285280
Pd	-14.439408	8.878782	9.659552

Table5_1a_TSiv_DG_0wat

Property	Value
Charge	0
Electronic Energy, BS1 (a.u.)	-980.279646
Thermal and entropic correction, BS1 (a.u.)	2.702191
Electronic Energy, BS2 (a.u.)	-980.609320
Number of Imaginary Frequencies	0
Imaginary frequencies (cm-1)	None

Molecular Geometry in Cartesian Coordinates

C	-8.033232	2.835865	5.926598
C	-8.879419	3.322205	4.772985
C	-8.040406	3.915359	3.668039
C	-7.087420	4.839175	4.130833
C	-7.118905	5.024633	5.620932
C	-7.131297	3.706773	6.363745
H	-9.049741	3.112552	1.944331
H	-8.296884	1.930934	6.472298
H	-9.627960	2.605988	4.428655
C	-8.291184	3.799985	2.306240
C	-6.398154	5.646369	3.234543
H	-6.381534	5.738697	5.994532
H	-6.577937	3.577730	7.296176
C	-6.639119	5.524212	1.863498
C	-7.581500	4.602211	1.403445
H	-5.689680	6.385225	3.600727
H	-7.785686	4.522236	0.339128
H	-6.109226	6.154644	1.163396
C	-10.412552	4.254895	6.682562
C	-9.715118	4.697224	5.410838
C	-8.672503	5.710261	5.868034
C	-8.839033	5.815548	7.374871
H	-10.455814	5.057249	4.636675

H	-8.705693	6.722380	5.375641
C	-9.966334	4.413916	9.172215
C	-8.682170	3.823723	9.791560
C	-10.507371	5.563675	10.030903
H	-10.726388	3.628099	9.108299
C	-8.938403	3.371166	11.237579
H	-7.903231	4.593492	9.778974
H	-8.332726	2.990514	9.179628
C	-10.775601	5.100179	11.469445
H	-9.768448	6.372132	10.029543
H	-11.419158	5.952891	9.557172
C	-9.502177	4.514771	12.098312
H	-8.008202	2.992045	11.671774
H	-9.648701	2.533890	11.237492
H	-11.145925	5.937117	12.071596
H	-11.564259	4.335662	11.467456
H	-9.714801	4.159983	13.111476
H	-8.748620	5.307942	12.191155
N	-9.757379	4.844831	7.776291
O	-8.185672	6.517912	8.124969
O	-11.302719	3.427705	6.781266
C	-12.403174	-3.773593	7.638379
H	-12.259183	-4.442326	8.496196
H	-11.780608	-2.892599	7.818949
H	-13.450930	-3.449711	7.672941
C	-13.201453	-4.452664	5.423738
H	-14.101727	-3.994341	5.851779
H	-12.974911	-3.923461	4.494367
H	-13.482240	-5.475368	5.142532
C	-11.455567	-5.758773	6.547439
H	-12.037059	-6.517881	6.008756
H	-11.534695	-6.059261	7.600119
C	-9.975968	-5.971341	6.144066
H	-9.962239	-6.810181	5.436421
H	-9.469078	-6.365006	7.034434
C	-8.728816	-5.082979	4.227395
H	-9.223216	-4.436108	3.497131
H	-7.647788	-4.920774	4.132875
H	-8.912248	-6.116055	3.906190
C	-8.134838	-4.388450	6.501235
H	-8.343770	-3.401542	6.922680
H	-7.999739	-5.070255	7.350283
H	-7.163910	-4.333015	5.993324
N	0.180147	5.665912	6.791635
N	-0.768908	8.284699	7.652065
C	-0.153495	4.444212	7.570124
H	-1.213525	4.453984	7.840146
H	0.037690	3.517238	7.014902
H	0.409806	4.363792	8.508190
C	0.719319	5.312090	5.451063
H	0.110494	5.770913	4.667389
H	1.747716	5.660626	5.293409
H	0.731130	4.230426	5.267689
C	1.141082	6.530955	7.537163
H	1.494642	6.026354	8.445515
H	2.054595	6.699023	6.952402
C	0.641738	7.927583	7.981646
H	1.342624	8.659987	7.561021
H	0.813556	7.984168	9.064266
C	-0.823663	9.529447	6.839411
H	-1.479946	10.294013	7.273729
H	0.157114	10.004715	6.712044
H	-1.197465	9.307971	5.835998
C	-1.586427	8.417832	8.886477
H	-2.350937	7.636260	8.911763
H	-0.998491	8.310819	9.806674
H	-2.098388	9.385663	8.958120
N	-20.157153	3.698347	-1.939616
N	-18.888535	4.953663	-4.248571
C	-19.998976	2.254657	-1.620603
H	-20.308269	2.006314	-0.597591
H	-20.576501	1.599663	-2.284854
H	-18.950029	1.963436	-1.726227
C	-20.728417	4.439150	-0.783470
H	-20.898554	3.799730	0.091851
H	-20.046537	5.237812	-0.477915
H	-21.690916	4.915141	-1.008800
C	-20.993308	3.887670	-3.161068
H	-21.886283	4.484631	-2.935654
H	-21.394644	2.928110	-3.511456
C	-20.319481	4.552480	-4.387236
H	-20.450287	3.856998	-5.226152
H	-20.943919	5.414653	-4.654387
C	-18.728896	6.421272	-4.428260
H	-17.989347	6.680111	-5.196374
H	-19.660059	6.924701	-4.716603
H	-18.402187	6.878876	-3.490372
C	-18.030543	4.204836	-5.204869

H	-17.419163	4.862116	-5.835856
H	-17.348676	3.547127	-4.658518
H	-18.602958	3.566522	-5.889364
C	-7.384892	16.150521	-0.040323
H	-7.300381	16.279821	1.046030
H	-6.974137	15.167352	-0.286745
H	-6.715352	16.893505	-0.491474
C	-9.719205	16.546755	0.591118
H	-9.215082	16.602849	1.563954
H	-10.255467	17.495893	0.467856
H	-10.479292	15.763595	0.660109
C	-8.902909	17.279639	-1.604378
H	-7.947871	17.799279	-1.754479
H	-9.597410	18.076892	-1.310166
C	-9.361227	16.794628	-3.002017
H	-10.244972	17.390811	-3.263028
H	-8.593967	17.129676	-3.711645
C	-11.051359	15.102544	-3.555824
H	-11.149911	14.509723	-4.473898
H	-11.572950	14.563481	-2.759940
H	-11.611523	16.030322	-3.727086
C	-8.705266	14.728700	-4.155020
H	-9.226057	14.200000	-4.963164
H	-8.049737	15.462986	-4.639583
H	-8.052649	14.005783	-3.657422
N	-5.429233	2.143156	-3.410757
C	-4.670236	2.943785	-2.634027
H	-4.025838	3.649652	-3.138415
C	-4.683631	2.889574	-1.225749
H	-4.032427	3.557807	-0.677047
C	-5.528142	1.965038	-0.560707
C	-6.329006	1.142804	-1.393539
H	-7.002486	0.401601	-0.982703
C	-6.245497	1.265011	-2.793824
H	-6.851168	0.631713	-3.426636
C	-5.563627	1.858411	0.862459
C	-4.818145	2.505406	2.905917
C	-4.029967	3.387053	3.707302
C	-3.222393	4.401573	3.133089
H	-3.148926	4.528675	2.060780
C	-2.494642	5.276801	3.962324
H	-1.878747	6.047742	3.521389
C	-3.272096	4.243966	5.872368
H	-3.278699	4.194516	6.951359
C	-4.028897	3.320500	5.123280
H	-4.608612	2.570991	5.651725
C	-6.396034	0.882664	2.734219
C	-7.310228	-0.031977	3.341023
C	-7.379481	-0.210288	4.745484
H	-6.724778	0.333879	5.413825
C	-8.205741	-0.817469	2.570625
H	-8.226211	-0.762278	1.489683
C	-9.091667	-1.707786	3.207277
H	-9.768126	-2.309499	2.616485
C	-8.305940	-1.119549	5.295860
H	-8.360792	-1.249902	6.366576
C	-16.828911	2.891494	-0.116166
H	-16.842554	2.141431	-0.893804
C	-16.128032	2.644358	1.080112
H	-15.618048	1.696151	1.191745
C	-17.488135	4.980051	0.606415
H	-18.028474	5.893782	0.404216
C	-16.814605	4.823200	1.833316
H	-16.857908	5.631284	2.552241
C	-16.107163	3.624818	2.104043
C	-15.416281	3.416594	3.335617
C	-14.805378	4.147539	5.395703
C	-14.153729	2.094333	4.680821
C	-13.399696	0.896878	4.875269
C	-12.706866	0.628845	6.082651
H	-12.737388	1.320013	6.915168
C	-13.292831	-0.093009	3.865442
H	-13.788141	0.015677	2.908966
C	-12.529643	-1.253883	4.094189
H	-12.451745	-2.006190	3.322077
C	-11.965209	-0.561367	6.220820
H	-11.438877	-0.762802	7.141419
C	-14.774971	5.182723	6.379420
C	-15.385449	6.443422	6.157608
H	-15.901572	6.669423	5.233243
C	-15.326681	7.439758	7.150799
H	-15.791263	8.399807	6.975543
C	-14.135952	6.066644	8.571082
H	-13.663085	5.929643	9.532072
C	-14.131952	5.015240	7.632180
H	-13.643369	4.085012	7.894492
C	-7.841218	12.748822	0.792783
H	-7.026232	12.927977	0.106244

C	-10.048233	13.115700	1.355568
H	-10.990811	13.587103	1.117089
C	-9.939441	12.313335	2.507669
H	-10.813587	12.192164	3.134471
C	-8.704521	11.693032	2.825054
C	-7.638043	11.931164	1.921740
H	-6.656173	11.504008	2.078455
C	-8.544141	10.883026	3.989490
C	-9.398824	10.003859	5.900769
C	-7.226243	9.632561	5.350519
N	-12.758847	9.326191	8.474644
C	-10.514434	9.786711	6.766093
C	-11.808649	10.278247	6.456917
H	-12.000656	10.846823	5.555828
C	-12.886680	10.020754	7.325940
H	-13.873026	10.391289	7.084822
C	-10.398638	9.063092	7.979739
H	-9.446217	8.675519	8.316013
C	-11.533719	8.857322	8.789349
H	-11.440899	8.307793	9.714207
C	-5.966530	9.015693	5.622579
C	-5.723865	8.295919	6.819228
H	-6.478974	8.211313	7.589396
C	-4.471602	7.687770	7.037817
H	-4.296905	7.135713	7.949921
C	-3.661067	8.453585	5.017726
H	-2.833285	8.513475	4.324964
C	-4.881559	9.087739	4.711894
H	-4.961195	9.628015	3.777076
C	-15.278047	4.406976	-3.037095
H	-15.544942	3.384887	-3.264474
C	-15.945748	6.490872	-2.301915
H	-16.743583	7.126117	-1.944344
C	-14.642745	7.008797	-2.433704
H	-14.472028	8.045920	-2.174899
C	-13.946664	4.836327	-3.199597
H	-13.215304	4.123202	-3.557730
C	-13.593508	6.176654	-2.900395
C	-12.260601	6.661035	-3.063256
C	-10.758860	8.358488	-2.944411
C	-10.102603	6.311296	-3.671621
C	-10.451258	9.718648	-2.639382
C	-11.434250	10.622722	-2.163180
H	-12.460595	10.314872	-2.009416
C	-11.085490	11.958845	-1.885686
H	-11.838082	12.646393	-1.526534
C	-8.889244	11.596351	-2.491282
H	-7.892110	11.994431	-2.612443
C	-9.144462	10.245594	-2.796677
H	-8.325214	9.634586	-3.153161
C	-9.078531	5.442275	-4.155953
C	-7.750610	5.891839	-4.366588
H	-7.460398	6.916211	-4.170596
C	-6.773906	5.000890	-4.851829
H	-5.763631	5.348829	-5.012690
C	-9.329134	4.080456	-4.459821
H	-10.312047	3.643781	-4.336171
C	-8.287551	3.264089	-4.942467
H	-8.483715	2.227320	-5.175840
C	-4.223220	-0.289003	-5.226034
H	-5.252850	-0.247193	-4.859817
H	-4.174472	-1.127179	-5.932322
H	-3.583265	-0.565217	-4.378712
C	-2.648119	1.586686	-5.166530
H	-2.885197	2.574472	-4.761383
H	-2.285415	0.967838	-4.336302
H	-1.796390	1.721221	-5.844988
C	-3.616141	0.866520	-7.303822
H	-2.580629	1.120320	-7.564359
H	-3.720698	-0.181008	-7.614032
C	-4.518888	1.695418	-8.250601
H	-3.842898	2.277760	-8.889756
H	-4.986820	0.980225	-8.939055
C	-5.278021	4.007741	-7.929400
H	-5.027333	4.547606	-7.011782
H	-4.436734	4.150764	-8.618809
H	-6.138223	4.514288	-8.384867
C	-6.914512	2.188818	-8.048396
H	-7.481125	3.023971	-8.478918
H	-6.922385	1.391139	-8.801827
H	-7.481715	1.816235	-7.190933
N	-16.156261	8.417240	10.835906
N	-14.148533	10.523955	10.982613
C	-17.323519	8.374354	9.914461
H	-17.766950	7.374058	9.833057
H	-18.136947	9.046630	10.215113
H	-17.020397	8.681485	8.909889
C	-15.919500	7.087019	11.456535

H	-16.618560	6.319308	11.101443
H	-14.906619	6.743452	11.225929
H	-16.006441	7.099827	12.550275
C	-16.343743	9.468206	11.878638
H	-16.391745	9.019451	12.879092
H	-17.313887	9.968431	11.763513
C	-15.277737	10.588462	11.955950
H	-15.817773	11.540068	11.870638
H	-14.903144	10.585214	12.987656
C	-14.098026	11.754635	10.148446
H	-13.117493	12.246505	10.171469
H	-14.319318	11.511960	9.105383
H	-14.827780	12.514065	10.455986
C	-12.855438	10.302277	11.681759
H	-12.093564	11.046333	11.417211
H	-12.946925	10.330826	12.774631
H	-12.457640	9.315853	11.425568
N	-12.031747	-4.397982	6.341027
N	-9.240551	-4.794851	5.594324
N	-3.845735	1.013980	-5.836514
N	-5.544216	2.577651	-7.620034
N	-8.785903	16.252178	-0.528641
N	-9.635162	15.336661	-3.166453
N	-4.748100	2.673904	1.565701
N	-5.605984	1.614352	3.553129
N	-6.431210	0.966118	1.386514
N	-9.144859	-1.863654	4.545213
N	-2.512975	5.206515	5.309130
N	-17.504846	4.034094	-0.355215
N	-14.766766	2.241085	3.485267
N	-15.450927	4.420500	4.239778
N	-14.168589	2.992718	5.690228
N	-14.720458	7.261095	8.342264
N	-11.877473	-1.493427	5.249955
N	-9.020629	13.339982	0.511343
N	-9.625190	10.731740	4.785618
N	-8.221856	9.431902	6.244243
N	-7.329911	10.323985	4.193909
N	-3.451632	7.764466	6.158013
N	-16.265900	5.216053	-2.603487
N	-12.035541	7.957716	-2.754330
N	-9.746535	7.585799	-3.397872
N	-11.337203	5.783667	-3.517350
N	-7.029493	3.708872	-5.142319
N	-9.838387	12.446482	-2.049224
Pd	-1.644818	6.727232	6.479162
Pd	-9.321182	14.339030	-1.309854
Pd	-10.570369	-3.135027	5.429597
Pd	-5.461670	2.362990	-5.500684
Pd	-18.204286	4.481879	-2.285230
Pd	-14.439515	8.877066	9.659007

Table5_1a_reactant_1wat

**Property**	**Value**
Charge	0
Electronic Energy, BS1 (a.u.)	-1056.725729
Thermal and entropic correction, BS1 (a.u.)	2.716658
Electronic Energy, BS2 (a.u.)	-1057.098036
Number of Imaginary Frequencies	0
Imaginary frequencies (cm-1)	None

Molecular Geometry in Cartesian Coordinates

C	8.432847	1.055238	6.907828
H	8.821541	0.609244	7.831874
H	7.673384	1.783479	7.206144
H	7.921538	0.248409	6.367933
C	9.590837	1.096013	4.748829
H	8.865168	0.287466	4.594971
H	9.416932	1.843799	3.970054
H	10.579324	0.662026	4.553196
C	10.798414	1.702464	6.797264
H	11.558049	1.188320	6.194644
H	10.739111	1.106642	7.717137
C	11.408635	3.066904	7.200787
H	12.412079	3.101794	6.757436
H	11.595460	3.013145	8.280968
C	11.416763	5.187497	5.967130
H	10.936450	5.256576	4.987424
H	11.510478	6.207681	6.359810
H	12.439609	4.831490	5.791305
C	10.201926	5.012412	8.087301
H	9.111802	4.989202	8.170105

H	10.597727	4.566741	9.008432
H	10.511207	6.065111	8.096834
N	3.186873	15.019034	10.411635
N	0.322424	15.348895	10.859201
C	3.933357	13.833795	10.909714
H	3.358772	12.924087	10.715539
H	4.914289	13.714943	10.432983
H	4.118420	13.863067	11.990800
C	3.995401	15.780667	9.422560
H	3.449723	15.868863	8.479593
H	4.224023	16.803245	9.748371
H	4.960091	15.306124	9.203550
C	2.767556	15.897919	11.542555
H	3.186382	15.539704	12.491714
H	3.183707	16.907305	11.430896
C	1.247374	16.074815	11.777955
H	1.052804	17.154820	11.757511
H	1.062037	15.789612	12.821495
C	-0.557741	16.300759	10.129881
H	-1.624687	16.074848	10.250928
H	-0.426241	17.342031	10.449728
H	-0.336827	16.270054	9.059099
C	-0.483332	14.341557	11.598894
H	-0.222681	13.334606	11.260510
H	-0.313806	14.364553	12.682613
H	-1.563612	14.467248	11.453165
N	2.422298	1.262234	-6.204836
N	2.387980	3.661127	-7.865263
C	3.656517	0.716304	-5.580675
H	3.447989	-0.003887	-4.779642
H	4.310088	0.201113	-6.295592
H	4.245271	1.529295	-5.146379
C	1.207073	0.650069	-5.604961
H	1.438320	-0.058270	-4.799578
H	0.566080	1.429460	-5.183220
H	0.596943	0.100156	-6.332335
C	2.441651	1.070186	-7.684535
H	1.591820	0.457011	-8.010876
H	3.322536	0.492166	-7.992048
C	2.425731	2.343667	-8.565759
H	3.301730	2.275503	-9.223517
H	1.571394	2.237984	-9.246577
C	1.152858	4.412957	-8.213338
H	1.359391	5.420650	-8.595076
H	0.544857	3.913819	-8.978102
H	0.518372	4.520478	-7.329073
C	3.602485	4.461796	-8.173409
H	3.368539	5.458992	-8.566709
H	4.199550	4.597234	-7.267140
H	4.257363	3.985558	-8.913677
C	-6.605365	15.823022	-0.762554
H	-7.190938	15.554384	0.125520
H	-5.557784	15.890651	-0.456853
H	-6.917525	16.837451	-1.040798
C	-7.692650	13.753546	-1.496511
H	-8.082148	13.857494	-0.476130
H	-8.568383	13.696098	-2.154840
H	-7.188481	12.784601	-1.557711
C	-7.185696	15.516923	-3.127789
H	-7.392711	16.579727	-2.948426
H	-8.144809	15.113435	-3.477235
C	-6.219365	15.459511	-4.336699
H	-6.800211	15.057408	-5.176620
H	-6.019688	16.499737	-4.624362
C	-4.890590	13.536172	-5.083842
H	-3.988236	13.527640	-5.707773
H	-4.904440	12.604913	-4.511115
H	-5.741878	13.491352	-5.774634
C	-3.768938	15.589060	-4.354366
H	-3.063632	15.206729	-5.102750
H	-4.040563	16.601760	-4.677790
H	-3.223285	15.695948	-3.412742
N	8.030869	13.630375	-0.052697
C	7.138552	14.177976	0.798431
H	6.817222	15.187662	0.585997
C	6.631816	13.504847	1.927746
H	5.928933	14.024275	2.565722
C	7.062290	12.185826	2.218056
C	7.995563	11.623234	1.311043
H	8.387573	10.623307	1.446194
C	8.441213	12.371524	0.204375
H	9.152772	11.938322	-0.483838
C	6.593878	11.469951	3.360816
C	5.362111	11.395123	5.265042
C	4.410901	11.999127	6.143758
C	3.870191	13.285802	5.891513
H	4.154864	13.861458	5.020226
C	2.953162	13.855614	6.795320

H	2.547073	14.838663	6.603725
C	3.032049	12.005095	8.168056
H	2.684140	11.514460	9.065870
C	3.955250	11.355912	7.322607
H	4.310103	10.372733	7.602764
C	6.688213	9.641739	4.700563
C	7.198603	8.335053	4.966777
C	6.901521	7.636962	6.164646
H	6.292928	8.080652	6.940999
C	8.038518	7.659344	4.045284
H	8.323034	8.105805	3.101478
C	8.518409	6.370000	4.343394
H	9.152753	5.855000	3.636403
C	7.430093	6.348069	6.378055
H	7.206552	5.818161	7.292328
C	3.492306	3.091573	-2.960223
H	4.416242	3.116734	-3.520210
C	3.527030	3.085317	-1.552057
H	4.491888	3.100217	-1.061695
C	1.185941	3.037147	-2.983562
H	0.273962	3.016457	-3.562845
C	1.122454	3.030792	-1.576041
H	0.148556	2.997830	-1.104950
C	2.317224	3.058296	-0.812707
C	2.303431	3.062975	0.614899
C	1.150377	3.068900	2.570250
C	3.419328	3.077480	2.591210
C	4.650348	3.116338	3.312227
C	4.696070	3.056470	4.727487
H	3.794476	2.953023	5.316991
C	5.901121	3.221737	2.653641
H	5.973612	3.267864	1.575027
C	7.090002	3.272168	3.405820
H	8.041360	3.355442	2.900068
C	5.935990	3.113789	5.394245
H	5.968363	3.064718	6.472881
C	-0.093298	3.165178	3.265081
C	-1.331462	3.212349	2.575946
H	-1.382931	3.162674	1.496075
C	-2.535070	3.320214	3.297311
H	-3.477585	3.355002	2.769505
C	-1.417783	3.348337	5.315743
H	-1.470850	3.413214	6.392813
C	-0.164604	3.240234	4.679354
H	0.728186	3.220718	5.291108
C	-4.022392	13.856301	0.726230
H	-3.791346	14.891600	0.520626
C	-5.012409	11.895700	0.034318
H	-5.572668	11.365977	-0.723071
C	-4.641167	11.230971	1.219193
H	-4.936962	10.197082	1.342399
C	-3.918448	11.923403	2.222567
C	-3.611486	13.278577	1.942902
H	-3.076166	13.897518	2.651208
C	-3.536668	11.298413	3.447544
C	-3.544953	9.489412	4.814569
C	-2.574541	11.423319	5.498492
N	-4.435937	5.351398	5.508171
C	-3.851970	8.115034	5.048742
C	-4.490830	7.310000	4.072318
H	-4.796233	7.714383	3.115644
C	-4.758379	5.953697	4.344491
H	-5.256289	5.344914	3.602774
C	-3.522007	7.469321	6.267461
H	-3.044531	8.002092	7.080069
C	-3.825311	6.105231	6.445637
H	-3.576256	5.618738	7.377171
C	-1.824769	12.152438	6.469512
C	-1.450693	11.579162	7.709801
H	-1.743065	10.572839	7.978117
C	-0.662804	12.314011	8.615054
H	-0.374423	11.865899	9.554596
C	-0.603993	14.135558	7.213081
H	-0.274108	15.149399	7.037233
C	-1.382340	13.478480	6.240326
H	-1.629724	14.008934	5.330302
C	3.387118	6.103720	-5.047934
H	4.330706	5.612689	-5.238370
C	1.082853	5.992221	-5.048377
H	0.191582	5.412661	-5.242601
C	0.969546	7.311767	-4.568954
H	-0.021213	7.717516	-4.409066
C	3.371895	7.429906	-4.571935
H	4.318053	7.930194	-4.409055
C	2.136215	8.076537	-4.315299
C	2.069917	9.415654	-3.824368
C	0.843760	11.187286	-3.107549
C	3.108202	11.317822	-3.148347

C	-0.425577	11.792016	-2.859682
C	-1.641908	11.106464	-3.106921
H	-1.656324	10.090438	-3.480746
C	-2.872170	11.753596	-2.883071
H	-3.798273	11.230863	-3.075566
C	-1.821046	13.684361	-2.177013
H	-1.912520	14.697512	-1.811913
C	-0.545350	13.118000	-2.370135
H	0.325542	13.723760	-2.155357
C	4.306195	12.066381	-2.943513
C	4.284189	13.406686	-2.482223
H	3.354676	13.920009	-2.273884
C	5.491603	14.110247	-2.307615
H	5.470818	15.135522	-1.967012
C	5.588604	11.515261	-3.192214
H	5.710591	10.499776	-3.547182
C	6.742784	12.294566	-2.980605
H	7.718198	11.869800	-3.170461
C	11.143624	14.481980	-0.732721
H	10.737678	13.490154	-0.950522
H	12.124892	14.534577	-1.220843
H	11.334763	14.529827	0.346633
C	9.784901	16.412069	-0.076586
H	8.697149	16.388700	0.035170
H	10.220359	16.111862	0.884688
H	10.063606	17.462625	-0.226088
C	10.796865	16.328413	-2.311735
H	10.882876	17.386004	-2.031181
H	11.831959	16.017899	-2.503535
C	10.070638	16.297343	-3.679294
H	9.868955	17.342723	-3.945961
H	10.814292	15.971888	-4.417970
C	7.650580	16.326921	-4.089492
H	6.948224	16.314412	-3.251383
H	7.911630	17.377841	-4.266290
H	7.105054	15.991504	-4.980378
C	8.984720	14.394406	-4.785782
H	8.195869	14.404645	-5.548371
H	9.936620	14.444696	-5.328925
H	8.953776	13.417195	-4.295421
N	-4.402722	1.125119	5.792713
N	-6.298024	3.157802	6.674048
C	-4.260724	0.590184	4.411673
H	-3.302408	0.081981	4.247109
H	-5.041072	-0.134641	4.147547
H	-4.332157	1.402222	3.683741
C	-3.231209	0.753647	6.629524
H	-2.467161	0.196698	6.072755
H	-2.757991	1.655165	7.028742
H	-3.497772	0.131137	7.492831
C	-5.673989	0.650646	6.414169
H	-5.469867	0.033312	7.298390
H	-6.215373	-0.022799	5.737552
C	-6.689704	1.730290	6.862077
H	-7.631996	1.508871	6.344611
H	-6.909916	1.524865	7.917567
C	-7.294808	3.874019	5.833333
H	-7.687281	4.778958	6.313915
H	-6.839266	4.177745	4.886591
H	-8.167659	3.259000	5.580612
C	-6.120703	3.838677	7.983651
H	-6.730872	4.745416	8.080697
H	-6.376579	3.201690	8.839589
H	-5.074499	4.130382	8.111451
N	9.482860	1.722941	6.093628
N	10.630265	4.293319	6.858484
N	10.198810	15.530887	-1.201030
N	8.828801	15.475345	-3.775986
N	-6.750013	14.841440	-1.870492
N	-4.945945	14.701055	-4.161048
N	5.692238	12.091285	4.154261
N	5.831971	10.172825	5.605992
N	7.091997	10.230180	3.553730
N	8.228993	5.722343	5.489584
N	2.542979	13.238203	7.921294
N	2.346354	3.063365	-3.670763
N	3.498355	3.123863	1.242245
N	1.095231	3.058262	1.218981
N	2.278187	3.045382	3.317015
N	-2.587099	3.380387	4.643029
N	7.118640	3.217122	4.753068
N	-4.715946	13.187527	-0.218273
N	-3.863046	9.995490	3.602330
N	-2.916131	10.151774	5.808006
N	-2.845908	12.051743	4.331717
N	-0.234803	13.572204	8.383076
N	2.266455	5.392520	-5.290061
N	0.839929	9.922950	-3.584045

N	1.941500	11.928180	-2.837752
N	3.241429	10.057802	-3.619176
N	6.704588	13.573411	-2.552472
N	-2.966981	13.021707	-2.435383
Pd	1.454895	14.291625	9.391109
Pd	-4.838639	13.938757	-2.170937
Pd	8.862724	3.743785	5.800676
Pd	8.449005	14.537407	-1.899770
Pd	2.361488	3.346865	-5.756760
Pd	-4.428530	3.257248	5.653413
C	2.774258	5.602185	6.111981
C	2.956161	5.944267	4.783425
C	1.831946	6.284673	3.979195
C	0.599010	6.590254	4.634768
C	0.583367	6.589220	6.056640
C	1.576382	5.936721	6.766471
H	2.839041	6.096433	2.082054
H	3.603520	5.207307	6.701161
H	3.924654	5.869760	4.298706
C	1.876594	6.259321	2.560215
C	-0.566463	6.788431	3.851403
H	-0.279493	7.009891	6.572307
H	1.492671	5.805309	7.848233
C	-0.516901	6.641043	2.482428
C	0.717700	6.394563	1.830167
H	-1.498717	7.006654	4.353230
H	0.739653	6.313303	0.749596
H	-1.421896	6.744012	1.889624
C	3.118926	8.924615	4.125251
C	3.175061	8.340699	5.486624
C	2.080452	8.658990	6.142802
C	1.239582	9.515775	5.272602
H	4.126327	7.894274	5.863216
H	1.836407	8.554618	7.216161
C	1.524423	10.440827	2.910949
C	0.077798	10.188333	2.457936
C	1.772541	11.922824	3.242424
H	2.204739	10.149155	2.101738
C	-0.287299	11.103210	1.285792
H	-0.586616	10.380718	3.301518
H	-0.031434	9.138779	2.182247
C	1.386155	12.824839	2.066382
H	1.175069	12.181938	4.130138
H	2.828496	12.057251	3.503004
C	-0.064688	12.580544	1.632477
H	-1.333055	10.926563	0.989131
H	0.328391	10.841884	0.414410
H	1.535668	13.875193	2.331764
H	2.053295	12.615756	1.218752
H	-0.314862	13.220481	0.778665
H	-0.734921	12.868086	2.453897
N	1.902817	9.587987	4.038468
O	0.228705	10.127772	5.568318
O	3.976828	8.924440	3.245489
O	5.287617	6.496064	2.638893
H	5.429039	6.473604	1.684122
H	4.959378	7.400410	2.806544

Table5_1a_TSiii_TSi-ii_h2o_1wat

Property	Value
Charge	0
Electronic Energy, BS1 (a.u.)	-1056.687167
Thermal and entropic correction, BS1 (a.u.)	2.717515
Electronic Energy, BS2 (a.u.)	-1057.055162
Number of Imaginary Frequencies	0
Imaginary frequencies (cm-1)	None

Molecular Geometry in Cartesian Coordinates

C	8.432924	1.055202	6.908719
H	8.821782	0.609383	7.832781
H	7.673419	1.783414	7.207013
H	7.921627	0.248245	6.369002
C	9.590577	1.095846	4.749539
H	8.864922	0.287251	4.595863
H	9.416532	1.843546	3.970716
H	10.579053	0.661880	4.553805
C	10.798442	1.702591	6.797705
H	11.558034	1.188517	6.194972
H	10.739360	1.106792	7.717607
C	11.408573	3.067117	7.201074
H	12.411946	3.102113	6.757569
H	11.595565	3.013424	8.281229

C	11.416286	5.187675	5.967365
H	10.935799	5.256754	4.987741
H	11.510005	6.207853	6.360058
H	12.439121	4.831730	5.791353
C	10.201746	5.012482	8.087695
H	9.111649	4.988995	8.170739
H	10.597853	4.566962	9.008768
H	10.510778	6.065256	8.097097
N	3.186223	15.018785	10.412553
N	0.321698	15.349003	10.859293
C	3.931968	13.833456	10.911519
H	3.357165	12.923888	10.717301
H	4.913157	13.714075	10.435450
H	4.116353	13.863051	11.992711
C	3.995568	15.779655	9.423551
H	3.450325	15.867873	8.480335
H	4.224653	16.802203	9.749137
H	4.960075	15.304496	9.205069
C	2.766693	15.898354	11.542861
H	3.185386	15.540704	12.492291
H	3.182814	16.907693	11.430675
C	1.246465	16.075294	11.777938
H	1.051862	17.155285	11.757071
H	1.060960	15.790460	12.821549
C	-0.558609	16.300553	10.129719
H	-1.625512	16.074380	10.250644
H	-0.427420	17.341901	10.449448
H	-0.337558	16.269762	9.058968
C	-0.483903	14.341676	11.599168
H	-0.223121	13.334704	11.260952
H	-0.314344	14.364878	12.682876
H	-1.564205	14.467204	11.453460
N	2.422213	1.262217	-6.204551
N	2.388142	3.661605	-7.864292
C	3.656448	0.716135	-5.580545
H	3.447936	-0.004219	-4.779651
H	4.309986	0.201065	-6.295581
H	4.245241	1.529021	-5.146105
C	1.207004	0.649828	-5.604869
H	1.438276	-0.058856	-4.799798
H	0.566065	1.429056	-5.182746
H	0.596807	0.100252	-6.332444
C	2.441603	1.070606	-7.684306
H	1.591927	0.457300	-8.010805
H	3.322628	0.492900	-7.992007
C	2.425308	2.344329	-8.565169
H	3.300941	2.276239	-9.223421
H	1.570593	2.238919	-9.245554
C	1.153380	4.414087	-8.212261
H	1.360381	5.421893	-8.593448
H	0.545363	3.915585	-8.977426
H	0.518725	4.521407	-7.328099
C	3.603020	4.461838	-8.172086
H	3.369551	5.459191	-8.565265
H	4.199949	4.596884	-7.265670
H	4.257860	3.985458	-8.912293
C	-6.604977	15.821860	-0.760172
H	-7.189414	15.552805	0.128520
H	-5.557051	15.889764	-0.455733
H	-6.917776	16.836271	-1.037783
C	-7.692159	13.752008	-1.493262
H	-8.080192	13.855372	-0.472264
H	-8.568856	13.694455	-2.150300
H	-7.187668	12.783297	-1.555627
C	-7.188343	15.516103	-3.124701
H	-7.396293	16.578601	-2.944602
H	-8.147404	15.111782	-3.473324
C	-6.223221	15.460315	-4.334655
H	-6.804690	15.058578	-5.174322
H	-6.024496	16.500902	-4.621653
C	-4.894244	13.538226	-5.084626
H	-3.992780	13.530784	-5.709851
H	-4.906598	12.606582	-4.512496
H	-5.746477	13.493208	-5.774239
C	-3.772873	15.591261	-4.355101
H	-3.068290	15.209840	-5.104625
H	-4.045423	16.604038	-4.677494
H	-3.226095	15.697757	-3.414079
N	8.030815	13.630533	-0.052022
C	7.138622	14.178182	0.799205
H	6.817345	15.187893	0.586812
C	6.631938	13.505073	1.928561
H	5.929186	14.024578	2.566610
C	7.062326	12.186009	2.218804
C	7.995504	11.623388	1.311711
H	8.387467	10.623434	1.446799
C	8.441098	12.371655	0.205006
H	9.152542	11.938399	-0.483288

C	6.593918	11.470089	3.361531
C	5.362091	11.395185	5.265718
C	4.410854	11.999152	6.144427
C	3.870003	13.285743	5.892078
H	4.154570	13.861361	5.020736
C	2.952942	13.855548	6.795841
H	2.546778	14.838550	6.604146
C	3.032119	12.005210	8.168811
H	2.684344	11.514665	9.066727
C	3.955321	11.356008	7.323367
H	4.310328	10.372919	7.603654
C	6.688161	9.641782	4.701160
C	7.198431	8.335034	4.967307
C	6.901640	7.637108	6.165347
H	6.293532	8.081031	6.941960
C	8.037903	7.659080	4.045583
H	8.322154	8.105366	3.101614
C	8.517596	6.369643	4.343606
H	9.151547	5.854435	3.636414
C	7.429993	6.348106	6.378657
H	7.206651	5.818328	7.293058
C	3.492077	3.090875	-2.959372
H	4.416036	3.115943	-3.519325
C	3.526751	3.084844	-1.551204
H	4.491588	3.099856	-1.060803
C	1.185730	3.036384	-2.982793
H	0.273769	3.015574	-3.562104
C	1.122190	3.030236	-1.575272
H	0.148268	2.997274	-1.104229
C	2.316922	3.057902	-0.811889
C	2.303076	3.062766	0.615715
C	1.149957	3.069103	2.571035
C	3.418908	3.077602	2.592056
C	4.649949	3.116197	3.313038
C	4.695726	3.056294	4.728286
H	3.794150	2.952882	5.317809
C	5.900711	3.221304	2.654396
H	5.973166	3.267402	1.575782
C	7.089624	3.271626	3.406522
H	8.040960	3.354787	2.900717
C	5.935687	3.113445	5.394994
H	5.968104	3.064340	6.473626
C	-0.093792	3.165349	3.265746
C	-1.331911	3.211929	2.576486
H	-1.383282	3.161900	1.496624
C	-2.535620	3.319579	3.297711
H	-3.478100	3.353841	2.769808
C	-1.418521	3.348691	5.316244
H	-1.471702	3.413852	6.393296
C	-0.165245	3.240853	4.679989
H	0.727507	3.221825	5.291814
C	-4.020404	13.855320	0.725088
H	-3.789822	14.890765	0.519697
C	-5.009535	11.894363	0.032780
H	-5.569518	11.364539	-0.724734
C	-4.637967	11.229574	1.217523
H	-4.933205	10.195500	1.340513
C	-3.915648	11.922175	2.221072
C	-3.609260	13.277534	1.941648
H	-3.074309	13.896595	2.650113
C	-3.533823	11.297192	3.446042
C	-3.542746	9.488424	4.813363
C	-2.572150	11.422283	5.497202
N	-4.436703	5.351253	5.508245
C	-3.850848	8.114371	5.047988
C	-4.490091	7.309429	4.071737
H	-4.795032	7.713676	3.114856
C	-4.758569	5.953389	4.344322
H	-5.256733	5.344668	3.602726
C	-3.521615	7.468876	6.267015
H	-3.044002	8.001594	7.079578
C	-3.825861	6.105057	6.445581
H	-3.577401	5.618732	7.377353
C	-1.823290	12.151752	6.468646
C	-1.450095	11.578838	7.709370
H	-1.742566	10.572564	7.977778
C	-0.663046	12.314065	8.615049
H	-0.375401	11.866264	9.554965
C	-0.603448	14.135245	7.212680
H	-0.273612	15.149091	7.036819
C	-1.380955	13.477826	6.239498
H	-1.627686	14.008026	5.329147
C	3.386946	6.103575	-5.046752
H	4.330534	5.612521	-5.237136
C	1.082669	5.991947	-5.046850
H	0.191411	5.412260	-5.240783
C	0.969355	7.311644	-4.567835
H	-0.021403	7.717409	-4.407993

C	3.371707	7.429907	-4.571157
H	4.317862	7.930298	-4.408573
C	2.136020	8.076547	-4.314550
C	2.069704	9.415814	-3.824018
C	0.843489	11.187671	-3.107840
C	3.107940	11.318103	-3.148247
C	-0.425886	11.792659	-2.860806
C	-1.642180	11.107184	-3.108434
H	-1.656543	10.090996	-3.481816
C	-2.872478	11.754671	-2.885792
H	-3.798549	11.232062	-3.078781
C	-1.821470	13.685594	-2.179997
H	-1.912989	14.698988	-1.815609
C	-0.545741	13.118891	-2.371947
H	0.325115	13.724632	-2.156967
C	4.305926	12.066617	-2.943242
C	4.283907	13.406884	-2.481836
H	3.354388	13.920237	-2.273593
C	5.491335	14.110360	-2.306962
H	5.470561	15.135602	-1.966257
C	5.588344	11.515447	-3.191787
H	5.710343	10.499985	-3.546816
C	6.742533	12.294658	-2.979900
H	7.717956	11.869845	-3.169601
C	11.143622	14.481880	-0.732681
H	10.737623	13.490093	-0.950564
H	12.124800	14.534538	-1.220978
H	11.334965	14.529552	0.346645
C	9.785083	16.411911	-0.075999
H	8.697342	16.388665	0.035897
H	10.220629	16.111476	0.885162
H	10.063912	17.462457	-0.225339
C	10.796578	16.328545	-2.311372
H	10.882490	17.386133	-2.030780
H	11.831687	16.018161	-2.503298
C	10.070224	16.297458	-3.678863
H	9.868421	17.342828	-3.945473
H	10.813837	15.972095	-4.417620
C	7.650165	16.326792	-4.089021
H	6.947865	16.314381	-3.250867
H	7.911137	17.377701	-4.266004
H	7.104601	15.991204	-4.979818
C	8.984458	14.394322	-4.785163
H	8.195616	14.404441	-5.547763
H	9.936360	14.444633	-5.328302
H	8.953580	13.417150	-4.294722
N	-4.403313	1.125083	5.793598
N	-6.299029	3.157810	6.673888
C	-4.260935	0.589734	4.412757
H	-3.302482	0.081676	4.248541
H	-5.041085	-0.135336	4.148719
H	-4.332394	1.401527	3.684550
C	-3.231947	0.754017	6.630794
H	-2.467796	0.196803	6.074432
H	-2.758791	1.655731	7.029657
H	-3.498676	0.131924	7.494350
C	-5.674682	0.650648	6.414878
H	-5.470697	0.033533	7.299283
H	-6.215861	-0.022995	5.738294
C	-6.690584	1.730318	6.862314
H	-7.632777	1.508649	6.344774
H	-6.910917	1.525190	7.917836
C	-7.295660	3.873671	5.832697
H	-7.688332	4.778729	6.312892
H	-6.839866	4.177159	4.885993
H	-8.168382	3.258484	5.579939
C	-6.122112	3.839127	7.983317
H	-6.732392	4.745839	8.079914
H	-6.378163	3.202395	8.839392
H	-5.075967	4.130961	8.111300
N	9.482766	1.722897	6.094294
N	10.630004	4.293421	6.858834
N	10.198734	15.530873	-1.200654
N	8.828462	15.475337	-3.775460
N	-6.750589	14.840508	-1.868183
N	-4.949160	14.702467	-4.160992
N	5.692254	12.091376	4.154971
N	5.831816	10.172800	5.606545
N	7.091973	10.230283	3.554374
N	8.228442	5.722136	5.489946
N	2.542898	13.238232	7.921919
N	2.346160	3.062539	-3.669968
N	3.497973	3.123869	1.243084
N	1.094866	3.058020	1.219765
N	2.277745	3.045888	3.317856
N	-2.587787	3.380120	4.643405
N	7.118326	3.216639	4.753771
N	-4.713726	13.186407	-0.219480

N	-3.860352	9.994320	3.600927
N	-2.913464	10.150638	5.806620
N	-2.843252	12.050586	4.330299
N	-0.235009	13.572254	8.383084
N	2.266280	5.392247	-5.288481
N	0.839708	9.923169	-3.583900
N	1.941213	11.928527	-2.837858
N	3.241193	10.058044	-3.618964
N	6.704326	13.573462	-2.551659
N	-2.967357	13.023036	-2.438844
Pd	1.454468	14.291635	9.391505
Pd	-4.839122	13.938924	-2.171524
Pd	8.862418	3.743647	5.801217
Pd	8.448781	14.537523	-1.899163
Pd	2.361336	3.346719	-5.755873
Pd	-4.429336	3.257135	5.653647
C	2.763446	5.602815	6.133776
C	2.985009	6.145586	4.825043
C	1.821728	6.300777	3.967114
C	0.603363	6.601734	4.617706
C	0.674821	6.760335	6.059075
C	1.594715	5.922666	6.768695
H	2.838347	6.095877	2.084804
H	3.567890	5.104669	6.664260
H	3.933771	5.973143	4.325544
C	1.875903	6.249533	2.562865
C	-0.562241	6.779340	3.853749
H	-0.216777	7.113937	6.569961
H	1.432527	5.692778	7.816056
C	-0.517669	6.635416	2.475152
C	0.707148	6.388472	1.827868
H	-1.494506	7.012061	4.355479
H	0.733812	6.310666	0.747393
H	-1.422807	6.742485	1.887246
C	3.121605	8.899536	4.117397
C	3.147771	8.159716	5.415590
C	1.966914	8.515015	6.116280
C	1.234513	9.499362	5.276010
H	4.103936	8.014861	5.894302
H	1.898459	8.613972	7.189700
C	1.533437	10.442105	2.913794
C	0.086844	10.188492	2.461250
C	1.776062	11.924352	3.244532
H	2.213785	10.145546	2.109448
C	-0.283986	11.102623	1.284029
H	-0.582264	10.380895	3.304931
H	-0.026564	9.138714	2.186095
C	1.388779	12.825546	2.063361
H	1.178735	12.183573	4.127480
H	2.832019	12.064315	3.499887
C	-0.062075	12.580214	1.630059
H	-1.324938	10.920436	0.992589
H	0.331568	10.841315	0.412549
H	1.537646	13.876075	2.328388
H	2.055820	12.616597	1.215597
H	-0.312954	13.219672	0.776100
H	-0.732107	12.867687	2.451660
N	1.916864	9.589717	4.051180
O	0.239949	10.138213	5.567334
O	3.979171	8.926906	3.242118
O	5.289565	6.493466	2.637091
H	5.428563	6.464338	1.682015
H	4.962073	7.398248	2.799034

Table5_1a_TSiii_DG_h2o_1wat

Property	Value
Charge	0
Electronic Energy, BS1 (a.u.)	-1056.732799
Thermal and entropic correction, BS1 (a.u.)	2.728616
Electronic Energy, BS2 (a.u.)	-1057.098456
Number of Imaginary Frequencies	0
Imaginary frequencies (cm-1)	None

Molecular Geometry in Cartesian Coordinates

C	8.432847	1.055238	6.907828
H	8.821541	0.609244	7.831874
H	7.673384	1.783479	7.206144
H	7.921538	0.248409	6.367933
C	9.590837	1.096013	4.748829
H	8.865168	0.287466	4.594971
H	9.416932	1.843799	3.970054
H	10.579324	0.662026	4.553196

C	10.798414	1.702464	6.797264
H	11.558049	1.188320	6.194644
H	10.739111	1.106642	7.717137
C	11.408635	3.066904	7.200787
H	12.412079	3.101794	6.757436
H	11.595460	3.013145	8.280968
C	11.416763	5.187497	5.967130
H	10.936450	5.256576	4.987424
H	11.510478	6.207681	6.359810
H	12.439609	4.831490	5.791305
C	10.201926	5.012412	8.087301
H	9.111802	4.989202	8.170105
H	10.597727	4.566741	9.008432
H	10.511207	6.065111	8.096834
N	3.186873	15.019034	10.411635
N	0.322424	15.348895	10.859201
C	3.933357	13.833795	10.909714
H	3.358772	12.924087	10.715539
H	4.914289	13.714943	10.432983
H	4.118420	13.863067	11.990800
C	3.995401	15.780667	9.422560
H	3.449723	15.868863	8.479593
H	4.224023	16.803245	9.748371
H	4.960091	15.306124	9.203550
C	2.767556	15.897919	11.542555
H	3.186382	15.539704	12.491714
H	3.183707	16.907305	11.430896
C	1.247374	16.074815	11.777955
H	1.052804	17.154820	11.757511
H	1.062037	15.789612	12.821495
C	-0.557741	16.300759	10.129881
H	-1.624687	16.074848	10.250928
H	-0.426241	17.342031	10.449728
H	-0.336827	16.270054	9.059099
C	-0.483332	14.341557	11.598894
H	-0.222681	13.334606	11.260510
H	-0.313806	14.364553	12.682613
H	-1.563612	14.467248	11.453165
N	2.422298	1.262234	-6.204836
N	2.387980	3.661127	-7.865263
C	3.656517	0.716304	-5.580675
H	3.447989	-0.003887	-4.779642
H	4.310088	0.201113	-6.295592
H	4.245271	1.529295	-5.146379
C	1.207073	0.650069	-5.604961
H	1.438320	-0.058270	-4.799578
H	0.566080	1.429460	-5.183220
H	0.596943	0.100156	-6.332335
C	2.441651	1.070186	-7.684535
H	1.591820	0.457011	-8.010876
H	3.322536	0.492166	-7.992048
C	2.425731	2.343667	-8.565759
H	3.301730	2.275503	-9.223517
H	1.571394	2.237984	-9.246577
C	1.152858	4.412957	-8.213338
H	1.359391	5.420650	-8.595076
H	0.544857	3.913819	-8.978102
H	0.518372	4.520478	-7.329073
C	3.602485	4.461796	-8.173409
H	3.368539	5.458992	-8.566709
H	4.199550	4.597234	-7.267140
H	4.257363	3.985558	-8.913677
C	-6.605365	15.823022	-0.762554
H	-7.190938	15.554384	0.125520
H	-5.557784	15.890651	-0.456853
H	-6.917525	16.837451	-1.040798
C	-7.692650	13.753546	-1.496511
H	-8.082148	13.857494	-0.476130
H	-8.568383	13.696098	-2.154840
H	-7.188481	12.784601	-1.557711
C	-7.185696	15.516923	-3.127789
H	-7.392711	16.579727	-2.948426
H	-8.144809	15.113435	-3.477235
C	-6.219365	15.459511	-4.336699
H	-6.800211	15.057408	-5.176620
H	-6.019688	16.499737	-4.624362
C	-4.890590	13.536172	-5.083842
H	-3.988236	13.527640	-5.707773
H	-4.904440	12.604913	-4.511115
H	-5.741878	13.491352	-5.774634
C	-3.768938	15.589060	-4.354366
H	-3.063632	15.206729	-5.102750
H	-4.040563	16.601760	-4.677790
H	-3.223285	15.695948	-3.412742
N	8.030869	13.630375	-0.052697
C	7.138552	14.177976	0.798431
H	6.817222	15.187662	0.585997
C	6.631816	13.504847	1.927746

H	5.928933	14.024275	2.565722
C	7.062290	12.185826	2.218056
C	7.995563	11.623234	1.311043
H	8.387573	10.623307	1.446194
C	8.441213	12.371524	0.204375
H	9.152772	11.938322	-0.483838
C	6.593878	11.469951	3.360816
C	5.362111	11.395123	5.265042
C	4.410901	11.999127	6.143758
C	3.870191	13.285802	5.891513
H	4.154864	13.861458	5.020226
C	2.953162	13.855614	6.795320
H	2.547073	14.838663	6.603725
C	3.032049	12.005095	8.168056
H	2.684140	11.514460	9.065870
C	3.955250	11.355912	7.322607
H	4.310103	10.372733	7.602764
C	6.688213	9.641739	4.700563
C	7.198603	8.335053	4.966777
C	6.901521	7.636962	6.164646
H	6.292928	8.080652	6.940999
C	8.038518	7.659344	4.045284
H	8.323034	8.105805	3.101478
C	8.518409	6.370000	4.343394
H	9.152753	5.855000	3.636403
C	7.430093	6.348069	6.378055
H	7.206552	5.818161	7.292328
C	3.492306	3.091573	-2.960223
H	4.416242	3.116734	-3.520210
C	3.527030	3.085317	-1.552057
H	4.491888	3.100217	-1.061695
C	1.185941	3.037147	-2.983562
H	0.273962	3.016457	-3.562845
C	1.122454	3.030792	-1.576041
H	0.148556	2.997830	-1.104950
C	2.317224	3.058296	-0.812707
C	2.303431	3.062975	0.614899
C	1.150377	3.068900	2.570250
C	3.419328	3.077480	2.591210
C	4.650348	3.116338	3.312227
C	4.696070	3.056470	4.727487
H	3.794476	2.953023	5.316991
C	5.901121	3.221737	2.653641
H	5.973612	3.267864	1.575027
C	7.090002	3.272168	3.405820
H	8.041360	3.355442	2.900068
C	5.935990	3.113789	5.394245
H	5.968363	3.064718	6.472881
C	-0.093298	3.165178	3.265081
C	-1.331462	3.212349	2.575946
H	-1.382931	3.162674	1.496075
C	-2.535070	3.320214	3.297311
H	-3.477585	3.355002	2.769505
C	-1.417783	3.348337	5.315743
H	-1.470850	3.413214	6.392813
C	-0.164604	3.240234	4.679354
H	0.728186	3.220718	5.291108
C	-4.022392	13.856301	0.726230
H	-3.791346	14.891600	0.520626
C	-5.012409	11.895700	0.034318
H	-5.572668	11.365977	-0.723071
C	-4.641167	11.230971	1.219193
H	-4.936962	10.197082	1.342399
C	-3.918448	11.923403	2.222567
C	-3.611486	13.278577	1.942902
H	-3.076166	13.897518	2.651208
C	-3.536668	11.298413	3.447544
C	-3.544953	9.489412	4.814569
C	-2.574541	11.423319	5.498492
N	-4.435937	5.351398	5.508171
C	-3.851970	8.115034	5.048742
C	-4.490830	7.310000	4.072318
H	-4.796233	7.714383	3.115644
C	-4.758379	5.953697	4.344491
H	-5.256289	5.344914	3.602774
C	-3.522007	7.469321	6.267461
H	-3.044531	8.002092	7.080069
C	-3.825311	6.105231	6.445637
H	-3.576256	5.618738	7.377171
C	-1.824769	12.152438	6.469512
C	-1.450693	11.579162	7.709801
H	-1.743065	10.572839	7.978117
C	-0.662804	12.314011	8.615054
H	-0.374423	11.865899	9.554596
C	-0.603993	14.135558	7.213081
H	-0.274108	15.149399	7.037233
C	-1.382340	13.478480	6.240326
H	-1.629724	14.008934	5.330302

C	3.387118	6.103720	-5.047934
H	4.330706	5.612689	-5.238370
C	1.082853	5.992221	-5.048377
H	0.191582	5.412661	-5.242601
C	0.969546	7.311767	-4.568954
H	-0.021213	7.717516	-4.409066
C	3.371895	7.429906	-4.571935
H	4.318053	7.930194	-4.409055
C	2.136215	8.076537	-4.315299
C	2.069917	9.415654	-3.824368
C	0.843760	11.187286	-3.107549
C	3.108202	11.317822	-3.148347
C	-0.425577	11.792016	-2.859682
C	-1.641908	11.106464	-3.106921
H	-1.656324	10.090438	-3.480746
C	-2.872170	11.753596	-2.883071
H	-3.798273	11.230863	-3.075566
C	-1.821046	13.684361	-2.177013
H	-1.912520	14.697512	-1.811913
C	-0.545350	13.118000	-2.370135
H	0.325542	13.723760	-2.155357
C	4.306195	12.066381	-2.943513
C	4.284189	13.406686	-2.482223
H	3.354676	13.920009	-2.273884
C	5.491603	14.110247	-2.307615
H	5.470818	15.135522	-1.967012
C	5.588604	11.515261	-3.192214
H	5.710591	10.499776	-3.547182
C	6.742784	12.294566	-2.980605
H	7.718198	11.869800	-3.170461
C	11.143624	14.481980	-0.732721
H	10.737678	13.490154	-0.950522
H	12.124892	14.534577	-1.220843
H	11.334763	14.529827	0.346633
C	9.784901	16.412069	-0.076586
H	8.697149	16.388700	0.035170
H	10.220359	16.111862	0.884688
H	10.063606	17.462625	-0.226088
C	10.796865	16.328413	-2.311735
H	10.882876	17.386004	-2.031181
H	11.831959	16.017899	-2.503535
C	10.070638	16.297343	-3.679294
H	9.868955	17.342723	-3.945961
H	10.814292	15.971888	-4.417970
C	7.650580	16.326921	-4.089492
H	6.948224	16.314412	-3.251383
H	7.911630	17.377841	-4.266290
H	7.105054	15.991504	-4.980378
C	8.984720	14.394406	-4.785782
H	8.195869	14.404645	-5.548371
H	9.936620	14.444696	-5.328925
H	8.953776	13.417195	-4.295421
N	-4.402722	1.125119	5.792713
N	-6.298024	3.157802	6.674048
C	-4.260724	0.590184	4.411673
H	-3.302408	0.081981	4.247109
H	-5.041072	-0.134641	4.147547
H	-4.332157	1.402222	3.683741
C	-3.231209	0.753647	6.629524
H	-2.467161	0.196698	6.072755
H	-2.757991	1.655165	7.028742
H	-3.497772	0.131137	7.492831
C	-5.673989	0.650646	6.414169
H	-5.469867	0.033312	7.298390
H	-6.215373	-0.022799	5.737552
C	-6.689704	1.730290	6.862077
H	-7.631996	1.508871	6.344611
H	-6.909916	1.524865	7.917567
C	-7.294808	3.874019	5.833333
H	-7.687281	4.778958	6.313915
H	-6.839266	4.177745	4.886591
H	-8.167659	3.259000	5.580612
C	-6.120703	3.838677	7.983651
H	-6.730872	4.745416	8.080697
H	-6.376579	3.201690	8.839589
H	-5.074499	4.130382	8.111451
N	9.482860	1.722941	6.093628
N	10.630265	4.293319	6.858484
N	10.198810	15.530887	-1.201030
N	8.828801	15.475345	-3.775986
N	-6.750013	14.841440	-1.870492
N	-4.945945	14.701055	-4.161048
N	5.692238	12.091285	4.154261
N	5.831971	10.172825	5.605992
N	7.091997	10.230180	3.553730
N	8.228993	5.722343	5.489584
N	2.542979	13.238203	7.921294
N	2.346354	3.063365	-3.670763

N	3.498355	3.123863	1.242245
N	1.095231	3.058262	1.218981
N	2.278187	3.045382	3.317015
N	-2.587099	3.380387	4.643029
N	7.118640	3.217122	4.753068
N	-4.715946	13.187527	-0.218273
N	-3.863046	9.995490	3.602330
N	-2.916131	10.151774	5.808006
N	-2.845908	12.051743	4.331717
N	-0.234803	13.572204	8.383076
N	2.266455	5.392520	-5.290061
N	0.839929	9.922950	-3.584045
N	1.941500	11.928180	-2.837752
N	3.241429	10.057802	-3.619176
N	6.704588	13.573411	-2.552472
N	-2.966981	13.021707	-2.435383
Pd	1.454895	14.291625	9.391109
Pd	-4.838639	13.938757	-2.170937
Pd	8.862724	3.743785	5.800676
Pd	8.449005	14.537407	-1.899770
Pd	2.361488	3.346865	-5.756760
Pd	-4.428530	3.257248	5.653413
C	2.756073	5.602185	6.157444
C	3.010717	6.317070	4.865260
C	1.813761	6.311951	3.961010
C	0.608103	6.608439	4.607490
C	0.756129	6.907466	6.065733
C	1.612753	5.909443	6.775564
H	2.839041	6.096433	2.091147
H	3.539871	5.016359	6.637512
H	3.942839	6.060708	4.353262
C	1.876594	6.241136	2.569308
C	-0.557370	6.770246	3.860496
H	-0.161287	7.200839	6.572307
H	1.383558	5.596176	7.793677
C	-0.516901	6.631950	2.473335
C	0.699515	6.385470	1.830167
H	-1.489624	7.015747	4.362323
H	0.730560	6.313303	0.749596
H	-1.421896	6.744012	1.889624
C	3.118926	8.879152	4.116158
C	3.120505	8.004267	5.359325
C	1.862226	8.386207	6.097339
C	1.221397	9.479404	5.281695
H	4.080864	8.121593	5.926865
H	1.945520	8.663731	7.170698
C	1.533516	10.440827	2.920042
C	0.086891	10.188333	2.467029
C	1.772541	11.922824	3.251517
H	2.213832	10.140062	2.119923
C	-0.287299	11.103210	1.285792
H	-0.586616	10.380718	3.310611
H	-0.031434	9.138779	2.191340
C	1.386155	12.824839	2.066382
H	1.175069	12.181938	4.130138
H	2.828496	12.066344	3.503004
C	-0.064688	12.580544	1.632477
H	-1.323962	10.917470	0.998224
H	0.328391	10.841884	0.414410
H	1.535668	13.875193	2.331764
H	2.053295	12.615756	1.218752
H	-0.314862	13.220481	0.778665
H	-0.734921	12.868086	2.453897
N	1.921002	9.587987	4.065746
O	0.237798	10.136865	5.568318
O	3.976828	8.933533	3.245489
O	5.287617	6.496064	2.638893
H	5.429039	6.464511	1.684122
H	4.959378	7.400410	2.797451

Table5_1a_reactant_2wat

Property	Value
Charge	0
Electronic Energy, BS1 (a.u.)	-1133.163488
Thermal and entropic correction, BS1 (a.u.)	2.746376
Electronic Energy, BS2 (a.u.)	-1133.572562
Number of Imaginary Frequencies	0
Imaginary frequencies (cm-1)	None

Molecular Geometry in Cartesian Coordinates

C	8.468015	1.050094	6.961471
H	8.839052	0.621212	7.900782

H	7.677442	1.756332	7.229820
H	7.999050	0.224877	6.411026
C	9.693428	1.112325	4.840584
H	8.998311	0.280849	4.669162
H	9.519637	1.851732	4.053732
H	10.700972	0.709663	4.677612
C	10.815495	1.765970	6.923777
H	11.600833	1.243415	6.362730
H	10.735304	1.200052	7.860735
C	11.395173	3.151720	7.298434
H	12.412901	3.181763	6.888648
H	11.545603	3.140715	8.385514
C	11.407483	5.197163	5.942654
H	10.934516	5.203184	4.956679
H	11.493778	6.239872	6.272977
H	12.432954	4.835507	5.796729
C	10.183569	5.143808	8.064166
H	9.093495	5.125654	8.145319
H	10.576018	4.752718	9.011233
H	10.493047	6.195192	8.014142
N	3.161892	15.056809	10.444895
N	0.287930	15.357240	10.849382
C	3.922630	13.878114	10.937061
H	3.367110	12.961121	10.723076
H	4.911503	13.782555	10.471514
H	4.093383	13.896113	12.020750
C	3.967437	15.842231	9.472378
H	3.432056	15.925507	8.523007
H	4.170619	16.866856	9.808413
H	4.944200	15.388964	9.262340
C	2.716608	15.915617	11.581331
H	3.121708	15.546757	12.532364
H	3.127459	16.929333	11.491397
C	1.191348	16.079512	11.792065
H	0.989670	17.158325	11.778961
H	0.989628	15.782533	12.829277
C	-0.579316	16.311180	10.107507
H	-1.647943	16.082250	10.205551
H	-0.456781	17.351032	10.435431
H	-0.336507	16.286761	9.041217
C	-0.530352	14.342880	11.565800
H	-0.259901	13.338500	11.227420
H	-0.383651	14.359699	12.652952
H	-1.607790	14.466565	11.398403
N	2.445333	1.353811	-6.352182
N	2.379728	3.957373	-7.670721
C	3.692260	0.743474	-5.818885
H	3.500021	-0.083930	-5.124236
H	4.345842	0.341452	-6.603110
H	4.275193	1.495485	-5.279944
C	1.243847	0.649889	-5.830128
H	1.492630	-0.169621	-5.144191
H	0.607855	1.352353	-5.283497
H	0.623280	0.210837	-6.621224
C	2.452573	1.366099	-7.844427
H	1.598680	0.804848	-8.245017
H	3.329251	0.833101	-8.234292
C	2.437656	2.748136	-8.543894
H	3.320949	2.776709	-9.194689
H	1.591575	2.730127	-9.242772
C	1.132541	4.729106	-7.916649
H	1.322078	5.783882	-8.152077
H	0.534711	4.331435	-8.746328
H	0.494308	4.699880	-7.029667
C	3.581047	4.812087	-7.864368
H	3.331004	5.846025	-8.133057
H	4.168761	4.845096	-6.942592
H	4.250448	4.443351	-8.651569
C	-6.556807	15.795018	-0.662947
H	-7.070817	15.503695	0.261652
H	-5.491826	15.884186	-0.432329
H	-6.911653	16.804156	-0.907399
C	-7.643447	13.706563	-1.341740
H	-7.965905	13.793578	-0.296647
H	-8.559896	13.631506	-1.940292
H	-7.121398	12.751259	-1.445336
C	-7.293557	15.495305	-2.985565
H	-7.519129	16.549539	-2.779742
H	-8.261330	15.066558	-3.275701
C	-6.408396	15.478684	-4.256437
H	-7.024536	15.050048	-5.057200
H	-6.275125	16.526734	-4.553948
C	-5.026279	13.644730	-5.124387
H	-4.158546	13.699267	-5.793597
H	-4.960192	12.699101	-4.579006
H	-5.909487	13.574019	-5.771500
C	-3.969609	15.730122	-4.392342
H	-3.292259	15.408617	-5.193354

H	-4.305522	16.739453	-4.660961
H	-3.374508	15.829185	-3.480111
N	8.133626	13.712704	0.058183
C	7.207176	14.232967	0.889972
H	6.890031	15.246963	0.690491
C	6.660927	13.532109	1.982949
H	5.934845	14.036326	2.606934
C	7.087496	12.209389	2.260112
C	8.066013	11.677677	1.384102
H	8.471113	10.683194	1.521249
C	8.545720	12.452883	0.311048
H	9.292163	12.040072	-0.352248
C	6.582977	11.468472	3.370717
C	5.307345	11.373886	5.244308
C	4.374277	11.991797	6.132129
C	3.863502	13.293571	5.893869
H	4.150982	13.867824	5.022572
C	2.980042	13.884662	6.817285
H	2.600287	14.880863	6.639788
C	3.030291	12.023218	8.179320
H	2.686648	11.538515	9.082166
C	3.919288	11.353346	7.313690
H	4.254290	10.360653	7.583765
C	6.640924	9.621406	4.686400
C	7.151551	8.315711	4.959281
C	6.822514	7.608794	6.143925
H	6.189077	8.044280	6.904988
C	8.024215	7.650056	4.061016
H	8.338175	8.105462	3.130894
C	8.498848	6.359233	4.364969
H	9.155680	5.850703	3.674145
C	7.348709	6.320241	6.365130
H	7.100267	5.782389	7.268629
C	3.487687	2.813127	-2.886630
H	4.413640	2.787335	-3.443146
C	3.524781	2.857543	-1.479560
H	4.490069	2.857742	-0.989920
C	1.186871	2.813007	-2.908893
H	0.272999	2.784151	-3.485488
C	1.122674	2.862941	-1.502898
H	0.148339	2.863838	-1.031722
C	2.316742	2.893055	-0.740455
C	2.303034	2.948168	0.685383
C	1.145579	3.038999	2.634732
C	3.414709	2.994908	2.662733
C	4.645973	3.031791	3.384049
C	4.690088	3.023201	4.800421
H	3.786557	2.953023	5.391541
C	5.899543	3.087521	2.724493
H	5.975017	3.085843	1.645133
C	7.088148	3.151897	3.476286
H	8.040817	3.199866	2.968848
C	5.930202	3.089587	5.466499
H	5.960576	3.080109	6.546521
C	-0.101975	3.156773	3.318834
C	-1.334088	3.203577	2.618561
H	-1.377955	3.148069	1.538674
C	-2.543993	3.310380	3.328589
H	-3.482346	3.339455	2.793062
C	-1.444282	3.347246	5.356954
H	-1.508026	3.412464	6.433456
C	-0.185098	3.240151	4.731778
H	0.702483	3.217524	5.350819
C	-3.899814	13.834387	0.650958
H	-3.683845	14.874229	0.451632
C	-4.866698	11.863490	-0.051922
H	-5.421790	11.332444	-0.812454
C	-4.479849	11.195233	1.125941
H	-4.760300	10.156437	1.243382
C	-3.763890	11.890917	2.132138
C	-3.474447	13.251962	1.860598
H	-2.945115	13.872896	2.571756
C	-3.378881	11.264884	3.355727
C	-3.405862	9.461954	4.730534
C	-2.436284	11.395756	5.415089
N	-4.440352	5.369606	5.497909
C	-3.752394	8.100657	4.984644
C	-4.416700	7.300281	4.021912
H	-4.705629	7.697235	3.057060
C	-4.735758	5.960726	4.320930
H	-5.255022	5.357144	3.589562
C	-3.449183	7.467145	6.216309
H	-2.960324	7.998420	7.022986
C	-3.804860	6.120010	6.421239
H	-3.579746	5.644733	7.364789
C	-1.718826	12.132967	6.403909
C	-1.358467	11.559288	7.647967
H	-1.636483	10.546423	7.906203

C	-0.612887	12.307784	8.577280
H	-0.338131	11.862257	9.522080
C	-0.564939	14.139376	7.189573
H	-0.256503	15.162485	7.028806
C	-1.303277	13.470458	6.193793
H	-1.544561	14.001188	5.282401
C	3.373659	5.999502	-4.550692
H	4.318390	5.509072	-4.738398
C	1.069050	5.911022	-4.636606
H	0.180122	5.349894	-4.888192
C	0.950352	7.207037	-4.097442
H	-0.042059	7.617200	-3.960815
C	3.352226	7.297907	-4.003824
H	4.297099	7.782236	-3.794388
C	2.114069	7.948121	-3.768591
C	2.043072	9.281649	-3.262399
C	0.814475	11.121335	-2.753170
C	3.078432	11.208525	-2.658909
C	-0.450878	11.778489	-2.684322
C	-1.661744	11.105627	-2.984912
H	-1.677078	10.061483	-3.269880
C	-2.884088	11.802840	-2.939860
H	-3.804417	11.289005	-3.179292
C	-1.838244	13.761545	-2.317984
H	-1.924953	14.808272	-2.063332
C	-0.569927	13.148396	-2.339175
H	0.297761	13.751905	-2.106621
C	4.277376	11.971987	-2.522775
C	4.257072	13.347592	-2.180674
H	3.329441	13.872539	-1.992487
C	5.462363	14.073224	-2.108829
H	5.440594	15.124517	-1.859105
C	5.557541	11.410659	-2.760784
H	5.680150	10.373021	-3.043416
C	6.709250	12.217024	-2.667251
H	7.682303	11.788858	-2.863864
C	11.184776	14.595336	-0.817724
H	10.790635	13.587904	-0.976547
H	12.137765	14.649540	-1.358547
H	11.430870	14.688523	0.247534
C	9.820321	16.521480	-0.164302
H	8.742015	16.471632	0.013406
H	10.319972	16.273753	0.780644
H	10.059670	17.572009	-0.371460
C	10.713545	16.369880	-2.446575
H	10.758903	17.443919	-2.225480
H	11.755139	16.096793	-2.659048
C	9.945954	16.227429	-3.784409
H	9.705930	17.246957	-4.112409
H	10.674355	15.873124	-4.525030
C	7.515982	16.165858	-4.120012
H	6.845541	16.208014	-3.257371
H	7.743744	17.204376	-4.391497
H	6.946580	15.746755	-4.959048
C	8.881180	14.222070	-4.719569
H	8.076729	14.164863	-5.463454
H	9.820915	14.250265	-5.285196
H	8.877948	13.281775	-4.160509
N	-4.458447	1.148427	5.823959
N	-6.340536	3.210671	6.664994
C	-4.317246	0.599925	4.448156
H	-3.367042	0.073678	4.293637
H	-5.108698	-0.113008	4.184725
H	-4.369704	1.407420	3.713409
C	-3.294552	0.770444	6.668634
H	-2.534424	0.200912	6.119308
H	-2.812827	1.669265	7.063714
H	-3.571636	0.157139	7.535189
C	-5.737344	0.694541	6.445204
H	-5.543970	0.088790	7.339839
H	-6.280543	0.015680	5.775490
C	-6.746338	1.789510	6.870506
H	-7.686482	1.569030	6.348744
H	-6.976504	1.601106	7.927044
C	-7.320843	3.921863	5.801227
H	-7.709250	4.838871	6.261620
H	-6.851272	4.204805	4.854924
H	-8.196993	3.311263	5.549204
C	-6.173290	3.910983	7.965719
H	-6.778543	4.823069	8.041823
H	-6.443422	3.289153	8.828354
H	-5.126704	4.197888	8.101132
N	9.524151	1.743530	6.176698
N	10.617840	4.355224	6.880575
N	10.193566	15.605597	-1.274919
N	8.725765	15.368157	-3.784713
N	-6.755193	14.820564	-1.768451
N	-5.091971	14.780835	-4.166706

N	5.652702	12.074000	4.141677
N	5.753142	10.138504	5.569427
N	7.068504	10.221696	3.553818
N	8.175792	5.702680	5.497340
N	2.573797	13.271439	7.946769
N	2.343938	2.791211	-3.602868
N	3.497865	3.001391	1.313077
N	1.094666	2.997488	1.284806
N	2.270798	3.014400	3.385968
N	-2.607417	3.373043	4.673224
N	7.115657	3.152849	4.824583
N	-4.590926	13.162012	-0.292398
N	-3.706291	9.961648	3.511233
N	-2.766259	10.120058	5.719162
N	-2.699158	12.022291	4.245225
N	-0.210152	13.577423	8.364681
N	2.256361	5.315586	-4.870832
N	0.812749	9.823081	-3.127222
N	1.909684	11.856386	-2.454530
N	3.213570	9.903286	-2.987741
N	6.672318	13.528552	-2.353123
N	-2.980102	13.109119	-2.618865
Pd	1.453822	14.312939	9.398840
Pd	-4.847543	13.970312	-2.210658
Pd	8.854996	3.743222	5.848177
Pd	8.429431	14.554585	-1.837805
Pd	2.360664	3.356289	-5.624462
Pd	-4.458749	3.279116	5.665207
C	2.655660	5.620349	5.897575
C	2.908509	5.906434	4.566607
C	1.842273	6.281570	3.698713
C	0.596236	6.654131	4.284976
C	0.504126	6.687743	5.702877
C	1.440051	6.027007	6.480035
H	2.944867	6.027058	1.859863
H	3.441484	5.221227	6.538194
H	3.901971	5.778387	4.136379
C	1.966063	6.237615	2.284693
C	-0.514155	6.881076	3.434071
H	-0.361073	7.154541	6.161208
H	1.304376	5.930898	7.556261
C	-0.398025	6.716962	2.071334
C	0.861267	6.424093	1.487459
H	-1.470462	7.139370	3.877380
H	-1.266231	6.846834	1.431388
H	0.956116	6.378569	0.409464
C	3.166199	8.878908	3.810215
C	3.212638	8.319797	5.183401
C	2.150091	8.693793	5.852638
C	1.358611	9.604971	4.994373
H	4.147485	7.805845	5.542710
H	1.897615	8.588524	6.916947
C	1.664174	10.526571	2.635751
C	0.242072	10.243237	2.133879
C	1.867135	12.007078	2.997191
H	2.359093	10.274118	1.829138
C	-0.093519	11.144203	0.942690
H	-0.464538	10.408441	2.952655
H	0.173004	9.189205	1.844584
C	1.521349	12.897134	1.799487
H	1.222047	12.255335	3.852430
H	2.905771	12.163768	3.312303
C	0.095517	12.624102	1.304390
H	-1.117538	10.953214	0.603813
H	0.578182	10.876074	0.117715
H	1.638619	13.951095	2.063707
H	2.229924	12.694003	0.983603
H	-0.132865	13.265430	0.446988
H	-0.611225	12.896619	2.099967
N	2.025760	9.655869	3.751611
O	0.394039	10.286871	5.290469
O	3.983872	8.762503	2.889356
O	5.397508	6.326791	2.646400
H	5.673856	6.212304	1.729150
H	5.046924	7.237744	2.694708
O	2.695117	8.687101	0.102851
H	3.127362	8.555470	0.963724
H	3.421249	8.907739	-0.494456

Table5_1a_TSiii_TSi-ii_h2o_2wat

Property	Value
Charge	0
Electronic Energy, BS1 (a.u.)	-1133.121908

Thermal and entropic correction, BS1 (a.u.)	2.742678
Electronic Energy, BS2 (a.u.)	-1133.527326
Number of Imaginary Frequencies	0
Imaginary frequencies (cm-1)	None

Molecular Geometry in Cartesian Coordinates

C	8.467905	1.049836	6.961078
H	8.838663	0.620903	7.900475
H	7.677180	1.755958	7.229261
H	7.999198	0.224631	6.410395
C	9.693920	1.112494	4.840539
H	8.999085	0.280831	4.668885
H	9.519972	1.851991	4.053807
H	10.701622	0.710200	4.677619
C	10.815349	1.765861	6.924210
H	11.600740	1.242861	6.363652
H	10.734734	1.200336	7.861367
C	11.395278	3.151602	7.298513
H	12.412986	3.181395	6.888667
H	11.545798	3.140765	8.385577
C	11.407753	5.196854	5.942364
H	10.934667	5.202814	4.956433
H	11.494228	6.239602	6.272520
H	12.433160	4.835037	5.796416
C	10.183998	5.143984	8.063948
H	9.093925	5.125953	8.145195
H	10.576476	4.753037	9.011062
H	10.493557	6.195332	8.013720
N	3.161809	15.056540	10.445162
N	0.287795	15.357068	10.849464
C	3.922644	13.877816	10.937080
H	3.367122	12.960855	10.722922
H	4.911474	13.782338	10.471429
H	4.093465	13.895662	12.020762
C	3.967322	15.842258	9.472829
H	3.432035	15.925689	8.523424
H	4.170361	16.866847	9.809051
H	4.944177	15.389191	9.262787
C	2.716440	15.915097	11.581765
H	3.121397	15.545942	12.532744
H	3.127379	16.928806	11.492196
C	1.191174	16.079075	11.792386
H	0.989602	17.157911	11.779527
H	0.989303	15.781889	12.829510
C	-0.579187	16.311223	10.107541
H	-1.647884	16.082677	10.205698
H	-0.456268	17.351074	10.435313
H	-0.336456	16.286657	9.041247
C	-0.530744	14.342718	11.565587
H	-0.260184	13.338338	11.227266
H	-0.384398	14.359537	12.652783
H	-1.608119	14.466403	11.397787
N	2.444997	1.353599	-6.351988
N	2.379854	3.957068	-7.670759
C	3.691864	0.743026	-5.818783
H	3.499514	-0.084303	-5.124075
H	4.345294	0.340826	-6.603046
H	4.275021	1.494918	-5.279909
C	1.243427	0.649989	-5.829728
H	1.492098	-0.169562	-5.143796
H	0.607683	1.352637	-5.283049
H	0.622673	0.211062	-6.620739
C	2.452082	1.365745	-7.844230
H	1.597974	0.804707	-8.244660
H	3.328559	0.832465	-8.234152
C	2.437511	2.747728	-8.543819
H	3.320824	2.776026	-9.194601
H	1.591435	2.729842	-9.242705
C	1.132776	4.728989	-7.916731
H	1.322481	5.783687	-8.152374
H	0.534780	4.331297	-8.746283
H	0.494634	4.700053	-7.029679
C	3.581299	4.811549	-7.864561
H	3.331439	5.845466	-8.133501
H	4.168969	4.844704	-6.942761
H	4.250653	4.442500	-8.651649
C	-6.556837	15.794963	-0.662992
H	-7.070969	15.503661	0.261545
H	-5.491853	15.883925	-0.432280
H	-6.911533	16.804153	-0.907465
C	-7.643480	13.706561	-1.341929
H	-7.965979	13.793517	-0.296847
H	-8.559879	13.631467	-1.940557
H	-7.121370	12.751301	-1.445539
C	-7.293474	15.495321	-2.985662
H	-7.519300	16.549482	-2.779737

H	-8.261106	15.066410	-3.276018
C	-6.408117	15.478944	-4.256383
H	-7.024220	15.050498	-5.057271
H	-6.274807	16.527032	-4.553736
C	-5.026007	13.645043	-5.124447
H	-4.158088	13.699435	-5.793429
H	-4.960304	12.699329	-4.579180
H	-5.909043	13.574546	-5.771811
C	-3.969318	15.730373	-4.392179
H	-3.292006	15.408987	-5.193271
H	-4.305241	16.739740	-4.660634
H	-3.374153	15.829270	-3.479949
N	8.133566	13.712877	0.058217
C	7.207143	14.233077	0.890068
H	6.889953	15.247067	0.690618
C	6.660981	13.532171	1.983059
H	5.934938	14.036356	2.607113
C	7.087564	12.209443	2.260137
C	8.066059	11.677788	1.384064
H	8.471208	10.683319	1.521159
C	8.545733	12.453070	0.311045
H	9.292198	12.040324	-0.352260
C	6.583083	11.468505	3.370745
C	5.307484	11.373908	5.244346
C	4.374409	11.991789	6.132187
C	3.863700	13.293602	5.894003
H	4.151250	13.867896	5.022756
C	2.980266	13.884693	6.817458
H	2.600603	14.880947	6.640047
C	3.030334	12.023115	8.179314
H	2.686638	11.538343	9.082098
C	3.919346	11.353276	7.313690
H	4.254279	10.360553	7.583735
C	6.641048	9.621412	4.686401
C	7.151666	8.315711	4.959305
C	6.822648	7.608815	6.143976
H	6.189240	8.044333	6.905046
C	8.024325	7.650031	4.061051
H	8.338321	8.105432	3.130939
C	8.499016	6.359246	4.365068
H	9.155922	5.850742	3.674296
C	7.348865	6.320274	6.365217
H	7.100478	5.782433	7.268739
C	3.487819	2.813288	-2.886588
H	4.413756	2.787540	-3.443136
C	3.524906	2.857712	-1.479513
H	4.490193	2.857962	-0.989869
C	1.186997	2.813032	-2.908838
H	0.273120	2.784085	-3.485428
C	1.122794	2.862994	-1.502853
H	0.148460	2.863794	-1.031679
C	2.316861	2.893161	-0.740407
C	2.303127	2.948275	0.685435
C	1.145604	3.039156	2.634753
C	3.414733	2.995015	2.662809
C	4.645964	3.031955	3.384166
C	4.690024	3.023496	4.800541
H	3.786474	2.953426	5.391663
C	5.899551	3.087583	2.724649
H	5.975071	3.085798	1.645291
C	7.088138	3.151933	3.476476
H	8.040830	3.199774	2.969073
C	5.930122	3.089792	5.466648
H	5.960481	3.080421	6.546672
C	-0.101973	3.156907	3.318812
C	-1.334054	3.203706	2.618490
H	-1.377875	3.148199	1.538602
C	-2.543991	3.310456	3.328465
H	-3.482321	3.339491	2.792898
C	-1.444372	3.347338	5.356873
H	-1.508172	3.412581	6.433371
C	-0.185156	3.240274	4.731753
H	0.702396	3.217644	5.350834
C	-3.899880	13.834356	0.650957
H	-3.683871	14.874181	0.451592
C	-4.866666	11.863436	-0.051998
H	-5.421716	11.332392	-0.812571
C	-4.479827	11.195180	1.125879
H	-4.760261	10.156379	1.243317
C	-3.763936	11.890879	2.132118
C	-3.474578	13.251947	1.860620
H	-2.945244	13.872877	2.571768
C	-3.378900	11.264830	3.355692
C	-3.405771	9.461837	4.730423
C	-2.436239	11.395635	5.415032
N	-4.440514	5.369547	5.497816
C	-3.752328	8.100538	4.984510
C	-4.416730	7.300208	4.021802

H	-4.705704	7.697174	3.056969
C	-4.735904	5.960695	4.320851
H	-5.255283	5.357168	3.589519
C	-3.449110	7.466994	6.216160
H	-2.960213	7.998239	7.022834
C	-3.804898	6.119894	6.421111
H	-3.579811	5.644614	7.364671
C	-1.718782	12.132816	6.403871
C	-1.358365	11.559075	7.647880
H	-1.636357	10.546188	7.906058
C	-0.612781	12.307542	8.577221
H	-0.337992	11.861999	9.522004
C	-0.564917	14.139201	7.189603
H	-0.256493	15.162314	7.028883
C	-1.303274	13.470326	6.193814
H	-1.544598	14.001108	5.282463
C	3.373719	5.999368	-4.550580
H	4.318462	5.508917	-4.738170
C	1.069133	5.911011	-4.636945
H	0.180225	5.349964	-4.888780
C	0.950417	7.206989	-4.097674
H	-0.041990	7.617219	-3.961230
C	3.352274	7.297722	-4.003616
H	4.297153	7.781958	-3.794008
C	2.114113	7.948005	-3.768574
C	2.043090	9.281526	-3.262334
C	0.814482	11.121227	-2.753209
C	3.078418	11.208385	-2.658703
C	-0.450859	11.778395	-2.684369
C	-1.661743	11.105554	-2.984897
H	-1.677094	10.061413	-3.269882
C	-2.884058	11.802819	-2.939888
H	-3.804403	11.289041	-3.179390
C	-1.838172	13.761501	-2.318052
H	-1.924864	14.808224	-2.063381
C	-0.569880	13.148308	-2.339244
H	0.297817	13.751791	-2.106658
C	4.277348	11.971859	-2.522546
C	4.256993	13.347499	-2.180610
H	3.329337	13.872437	-1.992510
C	5.462256	14.073186	-2.108835
H	5.440452	15.124497	-1.859213
C	5.557532	11.410532	-2.760440
H	5.680181	10.372865	-3.042942
C	6.709206	12.216958	-2.667018
H	7.682286	11.788829	-2.863606
C	11.184538	14.595592	-0.817505
H	10.790435	13.588151	-0.976378
H	12.137671	14.649810	-1.358078
H	11.430361	14.688819	0.247809
C	9.820086	16.521909	-0.164605
H	8.741757	16.472193	0.013045
H	10.319624	16.274359	0.780446
H	10.059573	17.572363	-0.371956
C	10.713494	16.369805	-2.446768
H	10.758857	17.443900	-2.225974
H	11.755102	16.096620	-2.659049
C	9.946101	16.226945	-3.784680
H	9.706469	17.246398	-4.113204
H	10.674504	15.872056	-4.525013
C	7.516025	16.166078	-4.119950
H	6.845743	16.208410	-3.257179
H	7.744091	17.204515	-4.391500
H	6.946359	15.747093	-4.958869
C	8.880568	14.221853	-4.719538
H	8.075900	14.164724	-5.463189
H	9.820140	14.249852	-5.285448
H	8.877379	13.281602	-4.160402
N	-4.458371	1.148368	5.823846
N	-6.340564	3.210497	6.664916
C	-4.317318	0.599826	4.448044
H	-3.367135	0.073565	4.293444
H	-5.108797	-0.113110	4.184708
H	-4.369845	1.407308	3.713291
C	-3.294331	0.770492	6.668372
H	-2.534159	0.201145	6.118914
H	-2.812709	1.669358	7.063480
H	-3.571220	0.157055	7.534895
C	-5.737152	0.694416	6.445290
H	-5.543628	0.088799	7.339982
H	-6.280354	0.015412	5.775723
C	-6.746217	1.789318	6.870580
H	-7.686387	1.568710	6.348915
H	-6.976272	1.600969	7.927151
C	-7.320962	3.921526	5.801119
H	-7.709441	4.838533	6.261454
H	-6.851439	4.204445	4.854781
H	-8.197054	3.310818	5.549163

C	-6.173334	3.910955	7.965565
H	-6.778649	4.823007	8.041583
H	-6.443392	3.289210	8.828284
H	-5.126765	4.197947	8.100908
N	9.524233	1.743456	6.176711
N	10.618083	4.355134	6.880454
N	10.193393	15.605777	-1.275003
N	8.725622	15.368077	-3.784764
N	-6.755192	14.820569	-1.768538
N	-5.091688	14.781072	-4.166639
N	5.652824	12.074038	4.141723
N	5.753299	10.138531	5.569439
N	7.068602	10.221719	3.553813
N	8.175966	5.702710	5.497446
N	2.573927	13.271384	7.946865
N	2.344067	2.791273	-3.602810
N	3.497939	3.001472	1.313155
N	1.094741	2.997624	1.284825
N	2.270807	3.014606	3.386016
N	-2.607479	3.373111	4.673097
N	7.115603	3.152946	4.824767
N	-4.590913	13.161969	-0.292436
N	-3.706222	9.961568	3.511141
N	-2.766104	10.119894	5.719043
N	-2.699202	12.022224	4.245216
N	-0.210091	13.577198	8.364671
N	2.256458	5.315535	-4.871010
N	0.812757	9.822976	-3.127272
N	1.909670	11.856275	-2.454477
N	3.213570	9.903128	-2.987475
N	6.672225	13.528519	-2.353046
N	-2.980044	13.109115	-2.618926
Pd	1.453834	14.312793	9.398913
Pd	-4.847433	13.970361	-2.210691
Pd	8.855075	3.743233	5.848290
Pd	8.429293	14.554705	-1.837770
Pd	2.360766	3.356165	-5.624456
Pd	-4.458856	3.279036	5.665018
C	2.645677	5.625673	5.917531
C	2.948370	6.116479	4.606506
C	1.837132	6.296969	3.688774
C	0.601169	6.664605	4.270173
C	0.604105	6.848044	5.708051
C	1.460113	6.007436	6.485131
H	2.944502	6.027324	1.864787
H	3.406541	5.121529	6.503092
H	3.911812	5.888426	4.161254
C	1.965757	6.227997	2.289712
C	-0.514286	6.871629	3.439347
H	-0.290984	7.259984	6.166454
H	1.244622	5.821422	7.531405
C	-0.398327	6.712444	2.066613
C	0.850892	6.419499	1.487595
H	-1.465535	7.144989	3.882755
H	-1.266602	6.847351	1.431764
H	0.950615	6.373942	0.409587
C	3.161285	8.854201	3.805107
C	3.172829	8.135325	5.118394
C	2.020491	8.559371	5.822667
C	1.348841	9.590394	4.999377
H	4.127682	7.955917	5.587491
H	1.942920	8.628909	6.896971
C	1.669205	10.526877	2.640706
C	0.247100	10.238474	2.138879
C	1.867134	12.007403	3.002102
H	2.364104	10.269490	1.839053
C	-0.088539	11.144354	0.947649
H	-0.459507	10.408646	2.957662
H	0.178091	9.189425	1.849608
C	1.521248	12.897403	1.799375
H	1.222088	12.255676	3.852371
H	2.905786	12.169123	3.312160
C	0.095417	12.624279	1.304311
H	-1.112527	10.948279	0.608740
H	0.578229	10.876249	0.117728
H	1.638472	13.951386	2.063526
H	2.229816	12.694268	0.983480
H	-0.133030	13.265560	0.446889
H	-0.611341	12.896765	2.099884
N	2.035870	9.651201	3.766540
O	0.399283	10.292310	5.290496
O	3.983851	8.767632	2.889165
O	5.397230	6.326769	2.645979
H	5.673330	6.212269	1.728665
H	5.051682	7.237726	2.689403
O	2.694494	8.687177	0.102720
H	3.131639	8.550437	0.958614
H	3.420725	8.907590	-0.494544

Table5_1a_TSiii_DG_h2o_2wat

Property	Value
Charge	0
Electronic Energy, BS1 (a.u.)	-1133.166835
Thermal and entropic correction, BS1 (a.u.)	2.750123
Electronic Energy, BS2 (a.u.)	-1133.570195
Number of Imaginary Frequencies	0
Imaginary frequencies (cm-1)	None

Molecular Geometry in Cartesian Coordinates

C	8.468015	1.050094	6.961471
H	8.839052	0.621212	7.900782
H	7.677442	1.756332	7.229820
H	7.999050	0.224877	6.411026
C	9.693428	1.112325	4.840584
H	8.998311	0.280849	4.669162
H	9.519637	1.851732	4.053732
H	10.700972	0.709663	4.677612
C	10.815495	1.765970	6.923777
H	11.600833	1.243415	6.362730
H	10.735304	1.200052	7.860735
C	11.395173	3.151720	7.298434
H	12.412901	3.181763	6.888648
H	11.545603	3.140715	8.385514
C	11.407483	5.197163	5.942654
H	10.934516	5.203184	4.956679
H	11.493778	6.239872	6.272977
H	12.432954	4.835507	5.796729
C	10.183569	5.143808	8.064166
H	9.093495	5.125654	8.145319
H	10.576018	4.752718	9.011233
H	10.493047	6.195192	8.014142
N	3.161892	15.056809	10.444895
N	0.287930	15.357240	10.849382
C	3.922630	13.878114	10.937061
H	3.367110	12.961121	10.723076
H	4.911503	13.782555	10.471514
H	4.093383	13.896113	12.020750
C	3.967437	15.842231	9.472378
H	3.432056	15.925507	8.523007
H	4.170619	16.866856	9.808413
H	4.944200	15.388964	9.262340
C	2.716608	15.915617	11.581331
H	3.121708	15.546757	12.532364
H	3.127459	16.929333	11.491397
C	1.191348	16.079512	11.792065
H	0.989670	17.158325	11.778961
H	0.989628	15.782533	12.829277
C	-0.579316	16.311180	10.107507
H	-1.647943	16.082250	10.205551
H	-0.456781	17.351032	10.435431
H	-0.336507	16.286761	9.041217
C	-0.530352	14.342880	11.565800
H	-0.259901	13.338500	11.227420
H	-0.383651	14.359699	12.652952
H	-1.607790	14.466565	11.398403
N	2.445333	1.353811	-6.352182
N	2.379728	3.957373	-7.670721
C	3.692260	0.743474	-5.818885
H	3.500021	-0.083930	-5.124236
H	4.345842	0.341452	-6.603110
H	4.275193	1.495485	-5.279944
C	1.243847	0.649889	-5.830128
H	1.492630	-0.169621	-5.144191
H	0.607855	1.352353	-5.283497
H	0.623280	0.210837	-6.621224
C	2.452573	1.366099	-7.844427
H	1.598680	0.804848	-8.245017
H	3.329251	0.833101	-8.234292
C	2.437656	2.748136	-8.543894
H	3.320949	2.776709	-9.194689
H	1.591575	2.730127	-9.242772
C	1.132541	4.729106	-7.916649
H	1.322078	5.783882	-8.152077
H	0.534711	4.331435	-8.746328
H	0.494308	4.699880	-7.029667
C	3.581047	4.812087	-7.864368
H	3.331004	5.846025	-8.133057
H	4.168761	4.845096	-6.942592
H	4.250448	4.443351	-8.651569
C	-6.556807	15.795018	-0.662947

H	-7.070817	15.503695	0.261652
H	-5.491826	15.884186	-0.432329
H	-6.911653	16.804156	-0.907399
C	-7.643447	13.706563	-1.341740
H	-7.965905	13.793578	-0.296647
H	-8.559896	13.631506	-1.940292
H	-7.121398	12.751259	-1.445336
C	-7.293557	15.495305	-2.985565
H	-7.519129	16.549539	-2.779742
H	-8.261330	15.066558	-3.275701
C	-6.408396	15.478684	-4.256437
H	-7.024536	15.050048	-5.057200
H	-6.275125	16.526734	-4.553948
C	-5.026279	13.644730	-5.124387
H	-4.158546	13.699267	-5.793597
H	-4.960192	12.699101	-4.579006
H	-5.909487	13.574019	-5.771500
C	-3.969609	15.730122	-4.392342
H	-3.292259	15.408617	-5.193354
H	-4.305522	16.739453	-4.660961
H	-3.374508	15.829185	-3.480111
N	8.133626	13.712704	0.058183
C	7.207176	14.232967	0.889972
H	6.890031	15.246963	0.690491
C	6.660927	13.532109	1.982949
H	5.934845	14.036326	2.606934
C	7.087496	12.209389	2.260112
C	8.066013	11.677677	1.384102
H	8.471113	10.683194	1.521249
C	8.545720	12.452883	0.311048
H	9.292163	12.040072	-0.352248
C	6.582977	11.468472	3.370717
C	5.307345	11.373886	5.244308
C	4.374277	11.991797	6.132129
C	3.863502	13.293571	5.893869
H	4.150982	13.867824	5.022572
C	2.980042	13.884662	6.817285
H	2.600287	14.880863	6.639788
C	3.030291	12.023218	8.179320
H	2.686648	11.538515	9.082166
C	3.919288	11.353346	7.313690
H	4.254290	10.360653	7.583765
C	6.640924	9.621406	4.686400
C	7.151551	8.315711	4.959281
C	6.822514	7.608794	6.143925
H	6.189077	8.044280	6.904988
C	8.024215	7.650056	4.061016
H	8.338175	8.105462	3.130894
C	8.498848	6.359233	4.364969
H	9.155680	5.850703	3.674145
C	7.348709	6.320241	6.365130
H	7.100267	5.782389	7.268629
C	3.487687	2.813127	-2.886630
H	4.413640	2.787335	-3.443146
C	3.524781	2.857543	-1.479560
H	4.490069	2.857742	-0.989920
C	1.186871	2.813007	-2.908893
H	0.272999	2.784151	-3.485488
C	1.122674	2.862941	-1.502898
H	0.148339	2.863838	-1.031722
C	2.316742	2.893055	-0.740455
C	2.303034	2.948168	0.685383
C	1.145579	3.038999	2.634732
C	3.414709	2.994908	2.662733
C	4.645973	3.031791	3.384049
C	4.690088	3.023201	4.800421
H	3.786557	2.953023	5.391541
C	5.899543	3.087521	2.724493
H	5.975017	3.085843	1.645133
C	7.088148	3.151897	3.476286
H	8.040817	3.199866	2.968848
C	5.930202	3.089587	5.466499
H	5.960576	3.080109	6.546521
C	-0.101975	3.156773	3.318834
C	-1.334088	3.203577	2.618561
H	-1.377955	3.148069	1.538674
C	-2.543993	3.310380	3.328589
H	-3.482346	3.339455	2.793062
C	-1.444282	3.347246	5.356954
H	-1.508026	3.412464	6.433456
C	-0.185098	3.240151	4.731778
H	0.702483	3.217524	5.350819
C	-3.899814	13.834387	0.650958
H	-3.683845	14.874229	0.451632
C	-4.866698	11.863490	-0.051922
H	-5.421790	11.332444	-0.812454
C	-4.479849	11.195233	1.125941
H	-4.760300	10.156437	1.243382

C	-3.763890	11.890917	2.132138
C	-3.474447	13.251962	1.860598
H	-2.945115	13.872896	2.571756
C	-3.378881	11.264884	3.355727
C	-3.405862	9.461954	4.730534
C	-2.436284	11.395756	5.415089
N	-4.440352	5.369606	5.497909
C	-3.752394	8.100657	4.984644
C	-4.416700	7.300281	4.021912
H	-4.705629	7.697235	3.057060
C	-4.735758	5.960726	4.320930
H	-5.255022	5.357144	3.589562
C	-3.449183	7.467145	6.216309
H	-2.960324	7.998420	7.022986
C	-3.804860	6.120010	6.421239
H	-3.579746	5.644733	7.364789
C	-1.718826	12.132967	6.403909
C	-1.358467	11.559288	7.647967
H	-1.636483	10.546423	7.906203
C	-0.612887	12.307784	8.577280
H	-0.338131	11.862257	9.522080
C	-0.564939	14.139376	7.189573
H	-0.256503	15.162485	7.028806
C	-1.303277	13.470458	6.193793
H	-1.544561	14.001188	5.282401
C	3.373659	5.999502	-4.550692
H	4.318390	5.509072	-4.738398
C	1.069050	5.911022	-4.636606
H	0.180122	5.349894	-4.888192
C	0.950352	7.207037	-4.097442
H	-0.042059	7.617200	-3.960815
C	3.352226	7.297907	-4.003824
H	4.297099	7.782236	-3.794388
C	2.114069	7.948121	-3.768591
C	2.043072	9.281649	-3.262399
C	0.814475	11.121335	-2.753170
C	3.078432	11.208525	-2.658909
C	-0.450878	11.778489	-2.684322
C	-1.661744	11.105627	-2.984912
H	-1.677078	10.061483	-3.269880
C	-2.884088	11.802840	-2.939860
H	-3.804417	11.289005	-3.179292
C	-1.838244	13.761545	-2.317984
H	-1.924953	14.808272	-2.063332
C	-0.569927	13.148396	-2.339175
H	0.297761	13.751905	-2.106621
C	4.277376	11.971987	-2.522775
C	4.257072	13.347592	-2.180674
H	3.329441	13.872539	-1.992487
C	5.462363	14.073224	-2.108829
H	5.440594	15.124517	-1.859105
C	5.557541	11.410659	-2.760784
H	5.680150	10.373021	-3.043416
C	6.709250	12.217024	-2.667251
H	7.682303	11.788858	-2.863864
C	11.184776	14.595336	-0.817724
H	10.790635	13.587904	-0.976547
H	12.137765	14.649540	-1.358547
H	11.430870	14.688523	0.247534
C	9.820321	16.521480	-0.164302
H	8.742015	16.471632	0.013406
H	10.319972	16.273753	0.780644
H	10.059670	17.572009	-0.371460
C	10.713545	16.369880	-2.446575
H	10.758903	17.443919	-2.225480
H	11.755139	16.096793	-2.659048
C	9.945954	16.227429	-3.784409
H	9.705930	17.246957	-4.112409
H	10.674355	15.873124	-4.525030
C	7.515982	16.165858	-4.120012
H	6.845541	16.208014	-3.257371
H	7.743744	17.204376	-4.391497
H	6.946580	15.746755	-4.959048
C	8.881180	14.222070	-4.719569
H	8.076729	14.164863	-5.463454
H	9.820915	14.250265	-5.285196
H	8.877948	13.281775	-4.160509
N	-4.458447	1.148427	5.823959
N	-6.340536	3.210671	6.664994
C	-4.317246	0.599925	4.448156
H	-3.367042	0.073678	4.293637
H	-5.108698	-0.113008	4.184725
H	-4.369704	1.407420	3.713409
C	-3.294552	0.770444	6.668634
H	-2.534424	0.200912	6.119308
H	-2.812827	1.669265	7.063714
H	-3.571636	0.157139	7.535189
C	-5.737344	0.694541	6.445204

H	-5.543970	0.088790	7.339839
H	-6.280543	0.015680	5.775490
C	-6.746338	1.789510	6.870506
H	-7.686482	1.569030	6.348744
H	-6.976504	1.601106	7.927044
C	-7.320843	3.921863	5.801227
H	-7.709250	4.838871	6.261620
H	-6.851272	4.204805	4.854924
H	-8.196993	3.311263	5.549204
C	-6.173290	3.910983	7.965719
H	-6.778543	4.823069	8.041823
H	-6.443422	3.289153	8.828354
H	-5.126704	4.197888	8.101132
N	9.524151	1.743530	6.176698
N	10.617840	4.355224	6.880575
N	10.193566	15.605597	-1.274919
N	8.725765	15.368157	-3.784713
N	-6.755193	14.820564	-1.768451
N	-5.091971	14.780835	-4.166706
N	5.652702	12.074000	4.141677
N	5.753142	10.138504	5.569427
N	7.068504	10.221696	3.553818
N	8.175792	5.702680	5.497340
N	2.573797	13.271439	7.946769
N	2.343938	2.791211	-3.602868
N	3.497865	3.001391	1.313077
N	1.094666	2.997488	1.284806
N	2.270798	3.014400	3.385968
N	-2.607417	3.373043	4.673224
N	7.115657	3.152849	4.824583
N	-4.590926	13.162012	-0.292398
N	-3.706291	9.961648	3.511233
N	-2.766259	10.120058	5.719162
N	-2.699158	12.022291	4.245225
N	-0.210152	13.577423	8.364681
N	2.256361	5.315586	-4.870832
N	0.812749	9.823081	-3.127222
N	1.909684	11.856386	-2.454530
N	3.213570	9.903286	-2.987741
N	6.672318	13.528552	-2.353123
N	-2.980102	13.109119	-2.618865
Pd	1.453822	14.312939	9.398840
Pd	-4.847543	13.970312	-2.210658
Pd	8.854996	3.743222	5.848177
Pd	8.429431	14.554585	-1.837805
Pd	2.360664	3.356289	-5.624462
Pd	-4.458749	3.279116	5.665207
C	2.635957	5.630201	5.936983
C	2.987323	6.320212	4.645421
C	1.832421	6.311125	3.679010
C	0.606088	6.673834	4.255421
C	0.701163	7.003002	5.712729
C	1.479459	5.987599	6.489887
H	2.944867	6.027058	1.869715
H	3.372521	5.024190	6.469231
H	3.921674	5.995127	4.185638
C	1.966063	6.217912	2.294545
C	-0.514155	6.861373	3.443923
H	-0.223147	7.361429	6.171060
H	1.186154	5.714158	7.507002
C	-0.398025	6.707110	2.061482
C	0.841564	6.414241	1.487459
H	-1.460610	7.149222	3.887232
H	-1.266231	6.846834	1.431388
H	0.946264	6.368717	0.409464
C	3.156347	8.829649	3.800363
C	3.133824	7.955279	5.055327
C	1.893943	8.427794	5.793527
C	1.338908	9.575416	5.004225
H	4.108077	8.101401	5.631376
H	1.986281	8.667338	6.877539
C	1.674026	10.526571	2.645603
C	0.251924	10.233385	2.143731
C	1.867135	12.007078	3.007043
H	2.368945	10.264266	1.848841
C	-0.083667	11.144203	0.952542
H	-0.454686	10.408441	2.962507
H	0.182856	9.189205	1.854436
C	1.521349	12.897134	1.799487
H	1.222047	12.255335	3.852430
H	2.905771	12.173620	3.312303
C	0.095517	12.624102	1.304390
H	-1.107686	10.943362	0.613665
H	0.578182	10.876074	0.117715
H	1.638619	13.951095	2.063707
H	2.229924	12.694003	0.983603
H	-0.132865	13.265430	0.446988
H	-0.611225	12.896619	2.099967

N	2.045463	9.646017	3.781166
O	0.403891	10.296723	5.290469
O	3.983872	8.772355	2.889356
O	5.397508	6.326791	2.646400
H	5.673856	6.212304	1.729150
H	5.056776	7.237744	2.684856
O	2.695117	8.687101	0.102851
H	3.137214	8.545618	0.953872
H	3.421249	8.907739	-0.494456

Table5_1b_reactant_0wat

Property	Value
Charge	0
Electronic Energy, BS1 (a.u.)	-1058.933618
Thermal and entropic correction, BS1 (a.u.)	2.751245
Electronic Energy, BS2 (a.u.)	-1059.289850
Number of Imaginary Frequencies	0
Imaginary frequencies (cm-1)	None

Molecular Geometry in Cartesian Coordinates

C	-9.246303	8.904783	4.692417
H	-10.060227	8.677131	5.392017
H	-8.330363	8.504476	5.136021
H	-9.153666	9.997918	4.673176
C	-9.520386	9.378902	2.304116
H	-9.364971	10.385395	2.712308
H	-8.751490	9.210630	1.545233
H	-10.481997	9.407111	1.776547
C	-10.679660	7.466724	3.312660
H	-11.387124	7.823758	2.553274
H	-11.234940	7.550810	4.255708
C	-10.486330	5.952297	3.055954
H	-11.111595	5.700985	2.189670
H	-10.970049	5.434278	3.894053
C	-8.934769	4.802864	1.543561
H	-8.264247	5.387727	0.907863
H	-8.519131	3.790134	1.617267
H	-9.880946	4.706259	0.996412
C	-8.693568	4.545319	3.968328
H	-7.890028	4.995741	4.557930
H	-9.512758	4.330740	4.665923
H	-8.334328	3.573592	3.607038
N	2.651280	-1.122825	7.474796
N	4.603688	0.611696	8.772362
C	1.227745	-1.124215	7.903200
H	0.877945	-0.096404	8.032890
H	0.562950	-1.610892	7.178486
H	1.065972	-1.634501	8.860914
C	2.814897	-1.820824	6.171427
H	3.258447	-1.144055	5.435696
H	3.471996	-2.697573	6.230487
H	1.864128	-2.178214	5.756605
C	3.523892	-1.735400	8.518922
H	2.917897	-2.182793	9.317249
H	4.093319	-2.580092	8.110483
C	4.547710	-0.810266	9.221105
H	5.527115	-1.294372	9.115972
H	4.333154	-0.871951	10.295756
C	5.956148	0.949314	8.253848
H	6.389683	1.834149	8.736339
H	6.684250	0.139384	8.387423
H	5.907176	1.150950	7.180592
C	4.209036	1.533077	9.870180
H	3.282455	2.050404	9.606143
H	4.021452	1.016872	10.820040
H	4.967015	2.298254	10.079668
N	-1.671911	17.624062	-3.471684
N	0.283018	16.900516	-5.514512
C	-3.037553	17.039932	-3.398737
H	-3.521329	17.203497	-2.427587
H	-3.721248	17.445993	-4.154681
H	-2.991889	15.960475	-3.566334
C	-1.329052	18.333698	-2.210852
H	-2.120957	18.267170	-1.454449
H	-0.421929	17.904468	-1.775980
H	-1.133369	19.403370	-2.356952
C	-1.543252	18.533253	-4.647932
H	-1.281483	19.550683	-4.330036
H	-2.508231	18.652355	-5.157052
C	-0.520397	18.137753	-5.741784
H	-1.081338	18.072956	-6.683026
H	0.134338	19.007664	-5.880275

C	1.731982	17.218805	-5.411646
H	2.347775	16.639864	-6.111324
H	1.957161	18.274959	-5.606054
H	2.090340	17.002419	-4.401358
C	0.033590	15.899751	-6.585984
H	0.953723	15.566133	-7.081729
H	-0.450924	15.014229	-6.164738
H	-0.625203	16.272564	-7.380172
C	12.329202	8.508853	2.708986
H	12.117343	8.518742	3.785443
H	11.585389	7.863539	2.233573
H	13.299382	8.010199	2.591399
C	12.021227	10.903470	3.127515
H	11.870748	10.483757	4.130079
H	12.829736	11.639527	3.220221
H	11.118181	11.461495	2.866094
C	13.556500	10.155086	1.365103
H	14.279726	9.339725	1.494582
H	14.062492	11.031391	1.790131
C	13.451638	10.407120	-0.159307
H	13.916336	11.384027	-0.344211
H	14.133324	9.692685	-0.638323
C	11.714619	11.647677	-1.372224
H	11.423928	11.567221	-2.427151
H	10.866296	12.074986	-0.830152
H	12.521038	12.390454	-1.331394
C	12.021343	9.252048	-1.786015
H	11.679527	9.603056	-2.767742
H	12.984781	8.755256	-1.955756
H	11.323681	8.478364	-1.452771
N	0.529470	2.766903	-3.865157
C	1.281378	2.348487	-2.826197
H	2.238187	1.905299	-3.063814
C	0.872771	2.452183	-1.482349
H	1.534222	2.075556	-0.712668
C	-0.387614	3.019378	-1.167420
C	-1.165095	3.456765	-2.269058
H	-2.147591	3.890661	-2.133468
C	-0.669761	3.315463	-3.580014
H	-1.268127	3.645848	-4.417258
C	-0.851340	3.130976	0.177819
C	-0.558208	2.735588	2.391519
C	0.246510	2.223956	3.454457
C	1.548964	1.711024	3.229369
H	1.979436	1.671402	2.237308
C	2.312318	1.225841	4.308982
H	3.301647	0.827570	4.131427
C	0.632871	1.697985	5.813759
H	0.283465	1.683598	6.835650
C	-0.206609	2.194964	4.796613
H	-1.193171	2.548968	5.065978
C	-2.443643	3.767329	1.662402
C	-3.647764	4.483844	1.942284
C	-4.141444	4.644084	3.262084
H	-3.633746	4.205355	4.111429
C	-4.410864	5.096063	0.915035
H	-4.123293	5.024588	-0.126015
C	-5.581945	5.809860	1.235950
H	-6.165020	6.271342	0.451246
C	-5.322325	5.380362	3.488722
H	-5.696765	5.502799	4.494880
C	-1.854020	14.165931	-1.827474
H	-2.351951	13.944449	-2.760814
C	-2.254933	13.511301	-0.646750
H	-3.069002	12.799824	-0.705373
C	-0.225567	15.359851	-0.708222
H	0.567759	16.092274	-0.753900
C	-0.555905	14.752476	0.519854
H	-0.004403	15.044933	1.404252
C	-1.602229	13.796338	0.579656
C	-1.986820	13.166844	1.802803
C	-1.743213	12.911430	4.042564
C	-3.357759	11.760406	2.940613
C	-4.378880	10.761337	2.942010
C	-4.784402	10.103830	4.131134
H	-4.343222	10.347814	5.088415
C	-5.033470	10.353887	1.752317
H	-4.797402	10.801323	0.795623
C	-6.024925	9.354189	1.801110
H	-6.527480	9.050309	0.893877
C	-5.783953	9.111900	4.082936
H	-6.092028	8.614129	4.990798
C	-1.048863	13.204248	5.255395
C	0.054731	14.093627	5.296825
H	0.414888	14.591341	4.405697
C	0.688813	14.371417	6.524416
H	1.518721	15.063109	6.558022
C	-0.731085	12.961548	7.670334

H	-1.028816	12.521965	8.612852
C	-1.430552	12.629204	6.494062
H	-2.264966	11.943584	6.568738
C	8.856946	8.165619	2.455415
H	9.379639	7.390792	1.913008
C	8.565168	10.417912	2.863737
H	8.858203	11.435636	2.648142
C	7.547292	10.164821	3.804367
H	7.083506	11.007627	4.299892
C	7.159105	8.830738	4.089266
C	7.853507	7.817423	3.381174
H	7.634311	6.768436	3.532689
C	6.130875	8.527117	5.032701
C	4.583796	9.220960	6.539255
C	4.856990	7.007553	6.135003
N	2.609793	12.429017	8.583107
C	3.910659	10.287419	7.209159
C	4.249030	11.645026	6.978458
H	5.030639	11.925733	6.284424
C	3.581239	12.667020	7.679126
H	3.849753	13.699930	7.508039
C	2.876757	10.055900	8.151600
H	2.552645	9.053899	8.403688
C	2.263301	11.144175	8.805092
H	1.482981	10.966948	9.531523
C	4.491705	5.647327	6.376460
C	3.501388	5.286802	7.326255
H	2.990764	6.033470	7.920568
C	3.177628	3.928940	7.527462
H	2.425127	3.655206	8.253326
C	4.710719	3.247135	5.943126
H	5.178511	2.431643	5.409424
C	5.099867	4.574015	5.676629
H	5.873075	4.742734	4.937724
C	0.577858	13.183129	-4.286492
H	-0.392368	13.164668	-4.762655
C	2.237840	14.415867	-3.270734
H	2.591355	15.380174	-2.934737
C	3.046488	13.275476	-3.094754
H	4.016047	13.398149	-2.629251
C	1.311955	11.988912	-4.149365
H	0.880138	11.074467	-4.535037
C	2.589267	12.007871	-3.534957
C	3.366446	10.821205	-3.371114
C	5.238249	9.781543	-2.613044
C	3.593198	8.574507	-3.610894
C	6.502272	9.831493	-1.950809
C	7.021208	11.033035	-1.406041
H	6.483996	11.969370	-1.485049
C	8.265127	11.025196	-0.743085
H	8.656842	11.942368	-0.326822
C	8.541938	8.766920	-1.114201
H	9.153690	7.883961	-0.994874
C	7.310159	8.677318	-1.790890
H	7.009098	7.713928	-2.181692
C	3.081402	7.320094	-4.062304
C	3.809430	6.112728	-3.906965
H	4.782281	6.093072	-3.432387
C	3.279088	4.902846	-4.396116
H	3.838051	3.984461	-4.284140
C	1.821401	7.209525	-4.703875
H	1.192953	8.074593	-4.873281
C	1.372075	5.955014	-5.161425
H	0.415997	5.872510	-5.658691
C	-0.830598	1.163229	-6.419191
H	-1.159081	2.157922	-6.105393
H	-1.314727	0.961639	-7.382937
H	-1.248244	0.439006	-5.708572
C	1.194447	0.062483	-5.590073
H	1.878345	0.505678	-4.860769
H	0.411763	-0.462597	-5.028433
H	1.760365	-0.711031	-6.123993
C	1.115687	0.910030	-7.892629
H	1.698799	-0.015727	-7.980146
H	0.262611	0.752857	-8.565127
C	1.973917	2.029275	-8.532249
H	2.896301	1.548768	-8.882843
H	1.459290	2.324644	-9.455564
C	3.747616	3.354761	-7.475305
H	3.986391	3.203322	-6.419262
H	4.334885	2.618754	-8.038582
H	4.133802	4.340541	-7.762985
C	1.719446	4.458310	-8.294167
H	2.470347	5.245886	-8.434644
H	1.263969	4.289212	-9.277938
H	0.933929	4.863968	-7.650573
N	0.371226	15.625281	10.217127
N	2.724437	14.177637	11.148780

C	0.376865	16.713369	9.203024
H	-0.620092	16.922745	8.795601
H	0.754624	17.666349	9.594211
H	1.022908	16.440062	8.363843
C	-1.009440	15.125276	10.451693
H	-1.757300	15.620292	9.819517
H	-1.058494	14.053400	10.241437
H	-1.346051	15.257167	11.487582
C	0.995847	16.084054	11.492433
H	0.267289	16.062480	12.313061
H	1.293567	17.138470	11.427330
C	2.237158	15.306554	11.994232
H	3.028369	16.051685	12.147005
H	1.996762	14.960348	13.007650
C	4.116767	14.424125	10.687616
H	4.798621	13.598493	10.926882
H	4.133134	14.559798	9.602876
H	4.562901	15.326543	11.124094
C	2.627291	12.885475	11.878219
H	3.582499	12.347592	11.924409
H	2.288689	12.999674	12.915614
H	1.904729	12.231093	11.382162
N	-9.453566	8.316423	3.342788
N	-9.089085	5.463080	2.867553
N	0.654322	1.117770	-6.488464
N	2.281968	3.221709	-7.689267
N	12.290001	9.864236	2.098460
N	12.093222	10.339665	-0.774229
N	-0.027916	2.682811	1.149562
N	-1.761067	3.263745	2.716681
N	-2.056234	3.711257	0.368196
N	-6.037401	5.951004	2.497075
N	1.872445	1.216460	5.584147
N	-0.863299	15.079863	-1.862926
N	-3.012356	12.289093	1.744330
N	-1.272058	13.478385	2.908222
N	-2.796596	12.071434	4.131563
N	0.306191	13.822791	7.696011
N	-6.401492	8.741866	2.942523
N	9.215864	9.440668	2.200234
N	5.552514	9.571520	5.664439
N	4.189028	7.958362	6.828647
N	5.823304	7.223373	5.214740
N	3.770945	2.921922	6.853693
N	1.023896	14.382282	-3.859061
N	4.559716	10.944212	-2.745336
N	4.800103	8.563530	-3.004160
N	2.812161	9.661914	-3.793820
N	2.086844	4.820194	-5.020866
N	9.020994	9.916637	-0.597169
Pd	3.218478	0.912333	7.171867
Pd	10.650350	9.893589	0.729313
Pd	-7.742153	7.115575	2.908516
Pd	1.377820	2.989305	-5.770026
Pd	-0.297899	16.001326	-3.671277
Pd	1.502011	14.008694	9.409020
C	0.388430	5.244533	5.328400
C	1.322636	5.666463	4.382085
C	0.897511	5.957066	3.049554
C	-0.462231	6.324645	2.866873
C	-1.298372	6.343626	4.027657
C	-0.952111	5.575313	5.144422
H	2.787539	5.610724	2.074130
H	0.710201	4.809280	6.269435
H	2.385773	5.544565	4.557893
C	1.748278	5.883722	1.925957
C	-0.913120	6.640610	1.565811
H	-2.296613	6.764911	3.933374
H	-1.677002	5.396220	5.934056
C	-0.086898	6.515426	0.464815
C	1.274122	6.119056	0.648680
H	-1.940185	6.972425	1.432218
C	2.200240	6.034534	-0.539421
H	1.832217	5.329970	-1.293129
H	2.283435	7.011085	-1.032904
H	3.201628	5.721757	-0.241640
C	-0.589081	6.847331	-0.916869
H	-0.106720	7.755231	-1.298756
H	-0.368116	6.054827	-1.635935
H	-1.666145	7.018866	-0.923602
C	-0.437810	9.096283	3.778092
C	-0.289024	8.230373	4.978663
C	1.024282	7.957899	5.151821
C	1.798936	8.669459	4.105128
H	-1.093986	8.173330	5.714707
H	1.522806	7.606841	6.053394
C	1.150713	10.248191	2.205591
C	1.962148	11.465943	2.679740

C	1.846676	9.497455	1.067646
H	0.167897	10.589384	1.861426
C	2.274529	12.390934	1.499424
H	2.895150	11.114549	3.135666
H	1.397496	11.996832	3.458259
C	2.206884	10.432593	-0.090746
H	2.751404	9.030186	1.465753
H	1.191304	8.696055	0.725632
C	3.025445	11.636469	0.394233
H	2.852311	13.255162	1.836544
H	1.331720	12.777738	1.087319
H	2.761402	9.858524	-0.841257
H	1.288310	10.788927	-0.577796
H	3.253098	12.311768	-0.436385
H	3.989021	11.287745	0.787701
N	0.856786	9.347897	3.318013
O	3.007390	8.716674	3.946876
O	-1.455733	9.527305	3.266460

Table5_1b_TSi_DDG_0wat

Property	Value
Charge	0
Electronic Energy, BS1 (a.u.)	-1058.927815
Thermal and entropic correction, BS1 (a.u.)	2.750151
Electronic Energy, BS2 (a.u.)	-1059.282491
Number of Imaginary Frequencies	0
Imaginary frequencies (cm-1)	None

Molecular Geometry in Cartesian Coordinates

C	-9.170285	8.884582	4.630921
H	-9.983154	8.747544	5.354958
H	-8.275898	8.439278	5.075037
H	-9.000712	9.966432	4.561853
C	-9.483692	9.280306	2.233655
H	-9.272013	10.293354	2.598267
H	-8.737711	9.043627	1.470108
H	-10.451791	9.334834	1.720129
C	-10.723556	7.481033	3.350392
H	-11.448713	7.883782	2.631663
H	-11.221153	7.592431	4.322277
C	-10.636373	5.960052	3.076047
H	-11.322063	5.755994	2.243682
H	-11.105248	5.463782	3.935426
C	-9.223310	4.793462	1.442651
H	-8.509543	5.345772	0.824778
H	-8.908035	3.742527	1.448572
H	-10.184875	4.821813	0.915194
C	-8.916262	4.384039	3.838042
H	-8.058605	4.740619	4.414564
H	-9.720887	4.188648	4.558078
H	-8.644207	3.410529	3.410850
N	2.550223	-0.986426	7.210878
N	4.623699	0.453197	8.675282
C	1.111421	-0.893372	7.573394
H	0.841799	0.149544	7.762200
H	0.446693	-1.268471	6.785124
H	0.858299	-1.454162	8.481818
C	2.720700	-1.601722	5.867183
H	3.272065	-0.923704	5.210384
H	3.285444	-2.542112	5.891552
H	1.765888	-1.830729	5.377624
C	3.314114	-1.747103	8.243078
H	2.639174	-2.155802	9.006042
H	3.789360	-2.633935	7.804590
C	4.427836	-0.988613	9.005939
H	5.354486	-1.556580	8.852980
H	4.209578	-1.116593	10.073986
C	6.008268	0.704386	8.192947
H	6.524874	1.484897	8.765338
H	6.649308	-0.184923	8.239179
H	5.989743	1.024445	7.147395
C	4.303778	1.314693	9.843949
H	3.429352	1.932176	9.622690
H	4.063661	0.742674	10.748902
H	5.125145	1.989553	10.115671
N	-1.798022	17.552982	-3.196017
N	0.087478	16.916368	-5.330799
C	-3.164673	16.973096	-3.111882
H	-3.616546	17.085628	-2.118449
H	-3.868912	17.422876	-3.822901
H	-3.131152	15.904264	-3.339983
C	-1.413526	18.198967	-1.913017

H	-2.187440	18.108207	-1.140600
H	-0.501816	17.738520	-1.521422
H	-1.206813	19.271723	-2.014877
C	-1.697979	18.517124	-4.330611
H	-1.411936	19.514846	-3.973719
H	-2.678518	18.671725	-4.798832
C	-0.720309	18.162713	-5.478496
H	-1.317051	18.136264	-6.399322
H	-0.068411	19.036178	-5.607548
C	1.539364	17.229945	-5.257176
H	2.133122	16.684921	-6.001748
H	1.759480	18.294214	-5.407857
H	1.928485	16.966610	-4.269902
C	-0.194608	15.964223	-6.437726
H	0.710012	15.653706	-6.975791
H	-0.666050	15.060448	-6.040931
H	-0.877330	16.371453	-7.193919
C	12.119577	8.570363	2.666369
H	11.837352	8.590828	3.726321
H	11.401801	7.929880	2.146484
H	13.089087	8.059034	2.616809
C	11.826597	10.973163	3.050442
H	11.597410	10.562792	4.041888
H	12.645588	11.688891	3.195571
H	10.959150	11.553201	2.724767
C	13.451813	10.186860	1.389275
H	14.150484	9.357994	1.560989
H	13.948685	11.053824	1.843283
C	13.441818	10.440342	-0.138342
H	13.933349	11.409204	-0.294072
H	14.138593	9.714611	-0.576984
C	11.799492	11.713017	-1.446677
H	11.572154	11.641217	-2.517694
H	10.926190	12.151057	-0.954943
H	12.613115	12.443128	-1.354036
C	12.093592	9.315172	-1.853774
H	11.822627	9.676950	-2.853621
H	13.056009	8.800059	-1.964075
H	11.360698	8.554239	-1.570778
N	0.624390	3.079960	-3.934517
C	1.361180	2.605746	-2.909094
H	2.318875	2.169141	-3.155105
C	0.935290	2.654761	-1.566700
H	1.583574	2.240297	-0.805266
C	-0.323285	3.222431	-1.242251
C	-1.078955	3.723861	-2.332447
H	-2.053633	4.172714	-2.189469
C	-0.570121	3.634275	-3.643006
H	-1.148322	4.017493	-4.472127
C	-0.801940	3.284857	0.101763
C	-0.482454	2.924822	2.319802
C	0.353584	2.485499	3.390483
C	1.633411	1.922489	3.156445
H	2.026211	1.801539	2.155090
C	2.418607	1.485322	4.239941
H	3.389828	1.047278	4.057779
C	0.810489	2.123089	5.768711
H	0.503821	2.192964	6.802518
C	-0.044808	2.584532	4.747912
H	-1.004924	2.997156	5.024550
C	-2.402314	3.888535	1.592263
C	-3.668996	4.485661	1.869897
C	-4.194378	4.555765	3.183914
H	-3.658556	4.142252	4.028057
C	-4.480591	5.037960	0.847393
H	-4.174953	5.022090	-0.191010
C	-5.725882	5.612205	1.169702
H	-6.344714	6.025751	0.385736
C	-5.451654	5.150293	3.409695
H	-5.851714	5.194094	4.412427
C	-1.951904	14.042049	-1.690192
H	-2.497178	13.876193	-2.608330
C	-2.311828	13.338158	-0.524768
H	-3.142756	12.646163	-0.577752
C	-0.242918	15.141876	-0.594152
H	0.563663	15.859678	-0.641118
C	-0.524332	14.476167	0.615619
H	0.078576	14.707641	1.484003
C	-1.595231	13.548167	0.680950
C	-1.943777	12.876521	1.892002
C	-1.618759	12.529654	4.109571
C	-3.267704	11.419301	3.019027
C	-4.293904	10.425827	2.999648
C	-4.678102	9.710912	4.162646
H	-4.211320	9.900157	5.120357
C	-4.988976	10.095585	1.808252
H	-4.767058	10.582486	0.866991
C	-6.002717	9.119432	1.830196

H	-6.532145	8.873457	0.920478
C	-5.708397	8.750822	4.089791
H	-6.009919	8.214019	4.977915
C	-0.906240	12.830408	5.310625
C	0.175036	13.748643	5.330727
H	0.508610	14.253201	4.433256
C	0.824396	14.047978	6.544218
H	1.638355	14.759297	6.560745
C	-0.539151	12.604593	7.721350
H	-0.805577	12.164924	8.671711
C	-1.249770	12.248343	6.557105
H	-2.067752	11.545813	6.646223
C	8.716722	8.258833	2.240453
H	9.194419	7.485216	1.655885
C	8.451058	10.510615	2.666459
H	8.717145	11.528600	2.420177
C	7.520263	10.256638	3.694171
H	7.096784	11.097546	4.228043
C	7.168869	8.921408	4.017962
C	7.796802	7.909606	3.247628
H	7.598215	6.859366	3.418382
C	6.244981	8.610532	5.062034
C	4.847719	9.276740	6.719172
C	5.089778	7.071157	6.261954
N	2.856502	12.431819	8.838001
C	4.184669	10.326103	7.424958
C	4.443891	11.693165	7.154652
H	5.169344	11.996204	6.410892
C	3.766307	12.694286	7.876971
H	3.976258	13.735344	7.675815
C	3.225780	10.064943	8.435619
H	2.969835	9.054088	8.724099
C	2.596625	11.134699	9.102837
H	1.873456	10.931413	9.879188
C	4.722723	5.709716	6.488615
C	3.783748	5.336703	7.483460
H	3.310254	6.075348	8.117239
C	3.466182	3.976984	7.677006
H	2.755280	3.694692	8.439995
C	4.912206	3.317707	6.005286
H	5.355698	2.509481	5.440718
C	5.284031	4.648168	5.734191
H	6.019002	4.827375	4.959755
C	0.485900	13.172470	-4.278198
H	-0.487773	13.156496	-4.747394
C	2.121991	14.384464	-3.199290
H	2.453794	15.336516	-2.811566
C	2.955551	13.255046	-3.084835
H	3.921079	13.373198	-2.610335
C	1.249615	11.990796	-4.213174
H	0.836653	11.087420	-4.643809
C	2.529067	12.006376	-3.602591
C	3.341264	10.835729	-3.518058
C	5.255375	9.815570	-2.848995
C	3.634185	8.615888	-3.891940
C	6.520975	9.873818	-2.190440
C	7.046411	11.085753	-1.675772
H	6.518851	12.024552	-1.783550
C	8.292231	11.090687	-1.018749
H	8.690380	12.018274	-0.632728
C	8.559474	8.825327	-1.334863
H	9.169913	7.943797	-1.199278
C	7.324988	8.721481	-2.005041
H	7.018674	7.750010	-2.371670
C	3.149723	7.375216	-4.406835
C	3.912523	6.182322	-4.335412
H	4.900248	6.166321	-3.892689
C	3.394138	4.982148	-4.860255
H	3.979568	4.075514	-4.804674
C	1.878600	7.263453	-5.025270
H	1.223159	8.118167	-5.134556
C	1.444413	6.019543	-5.523652
H	0.476054	5.939079	-5.996920
C	-0.955212	1.208271	-6.120040
H	-1.233982	2.241723	-5.897133
H	-1.544618	0.906217	-6.994698
H	-1.302511	0.585639	-5.286163
C	1.129961	0.142916	-5.399680
H	1.902518	0.641102	-4.807042
H	0.406648	-0.292791	-4.699163
H	1.615671	-0.701064	-5.905313
C	0.812325	0.746241	-7.757409
H	1.360053	-0.203489	-7.808016
H	-0.112208	0.551281	-8.315829
C	1.622840	1.763433	-8.597933
H	2.485461	1.214702	-8.997170
H	1.015966	1.988497	-9.484286
C	3.544282	3.099999	-7.860175

H	3.880159	3.048969	-6.820682
H	4.043454	2.280356	-8.391957
H	3.938796	4.029773	-8.288917
C	1.489103	4.214237	-8.592828
H	2.253445	4.942373	-8.892036
H	0.924709	3.969700	-9.501436
H	0.794965	4.725895	-7.920183
N	0.196720	15.465821	10.110537
N	2.632654	14.353684	11.261958
C	0.111747	16.457881	9.005126
H	-0.886067	16.506978	8.551533
H	0.353981	17.479890	9.322831
H	0.820006	16.200820	8.212866
C	-1.123224	14.826050	10.353329
H	-1.900719	15.172943	9.661283
H	-1.038421	13.742051	10.237813
H	-1.509253	15.004031	11.364778
C	0.716674	16.103565	11.355467
H	-0.041532	16.083047	12.148892
H	0.911296	17.172394	11.199459
C	2.004889	15.506017	11.972516
H	2.714411	16.336717	12.077784
H	1.754939	15.236031	13.006611
C	4.026753	14.678042	10.856656
H	4.758646	13.946032	11.220489
H	4.104759	14.704755	9.766404
H	4.365789	15.656047	11.220515
C	2.595009	13.125707	12.099415
H	3.585653	12.677426	12.246678
H	2.182506	13.299357	13.101120
H	1.960632	12.371232	11.625039
N	-9.449661	8.256766	3.312205
N	-9.284901	5.388046	2.804630
N	0.512341	1.113015	-6.342141
N	2.060382	3.018021	-7.918483
N	12.138616	9.921930	2.046291
N	12.121611	10.396951	-0.833455
N	0.010964	2.801966	1.067182
N	-1.684467	3.448627	2.650487
N	-2.008010	3.862070	0.299701
N	-6.214485	5.670295	2.425942
N	2.018417	1.574504	5.524324
N	-0.943939	14.936904	-1.728018
N	-3.001646	12.039112	1.847346
N	-1.192898	13.145317	2.983611
N	-2.637055	11.641937	4.198006
N	0.474307	13.494682	7.722738
N	-6.366870	8.460513	2.948186
N	9.049405	9.533465	1.953944
N	5.719521	9.647099	5.751933
N	4.513134	8.006793	7.049831
N	5.969105	7.303284	5.262454
N	4.023167	2.978308	6.960421
N	0.907895	14.355781	-3.786761
N	4.544583	10.963781	-2.915535
N	4.846931	8.606192	-3.294383
N	2.823448	9.690143	-4.012958
N	2.184356	4.894219	-5.450462
N	9.046070	9.985211	-0.846212
Pd	3.297457	1.010985	7.094101
Pd	10.583947	9.966804	0.578732
Pd	-7.823428	6.939786	2.880972
Pd	1.345574	3.026800	-5.909552
Pd	-0.441319	15.935217	-3.513579
Pd	1.539350	13.931123	9.481381
C	-0.466570	5.875283	5.742738
C	0.740599	5.999911	5.044861
C	0.764868	6.025221	3.615594
C	-0.413703	6.478755	2.965352
C	-1.510601	6.841896	3.805309
C	-1.635498	6.311395	5.100762
H	2.778315	5.291823	3.351137
H	2.656048	5.315133	0.881892
H	1.679255	5.805028	5.561657
C	1.881499	5.630686	2.846380
C	-0.433788	6.576436	1.555110
H	-2.370781	7.322142	3.339686
H	0.627847	6.193423	-0.264552
C	0.655066	6.146538	0.819088
C	1.811182	5.655204	1.466645
H	-1.312929	6.972982	1.059586
C	-0.478169	5.430275	7.182765
H	0.509895	5.097678	7.505610
H	-0.794412	6.230074	7.865965
H	-1.183224	4.608060	7.340051
C	-2.942646	6.394878	5.843408
H	-2.883698	7.063027	6.710531
H	-3.743090	6.761407	5.193134

H	-3.244818	5.416089	6.235449
C	-0.143171	9.333214	3.265775
C	-0.472933	8.774608	4.605643
C	0.681734	8.391638	5.202830
C	1.810465	8.720190	4.292241
H	-1.429647	9.008410	5.074220
H	0.872828	8.226102	6.264938
C	1.946478	9.879492	2.017377
C	2.365121	11.305108	2.418052
C	3.137793	9.063344	1.500510
H	1.197070	9.944432	1.222123
C	3.038838	12.025167	1.243362
H	3.059550	11.236392	3.265036
H	1.482600	11.857044	2.757043
C	3.797929	9.789654	0.321938
H	3.863734	8.932378	2.307170
H	2.802503	8.067545	1.205759
C	4.224040	11.213464	0.703422
H	3.366379	13.022880	1.549975
H	2.301704	12.162970	0.438496
H	4.658705	9.209225	-0.026361
H	3.084881	9.836994	-0.515688
H	4.665462	11.729122	-0.155422
H	5.002513	11.159282	1.477119
N	1.241615	9.234189	3.125111
O	3.007339	8.613784	4.504501
O	-0.889268	9.808718	2.422544

Table5_1b_TSi_TSi-ii_0wat

**Property**	**Value**
Charge	0
Electronic Energy, BS1 (a.u.)	-1058.886631
Thermal and entropic correction, BS1 (a.u.)	2.751577
Electronic Energy, BS2 (a.u.)	-1059.239409
Number of Imaginary Frequencies	0
Imaginary frequencies (cm-1)	None

Molecular Geometry in Cartesian Coordinates

C	-9.174666	8.881470	4.632239
H	-9.987641	8.743367	5.355954
H	-8.279983	8.436896	5.076488
H	-9.006124	9.963511	4.563622
C	-9.487500	9.277775	2.235012
H	-9.276816	10.290870	2.600074
H	-8.741032	9.042021	1.471657
H	-10.455454	9.331674	1.721147
C	-10.726212	7.476991	3.350598
H	-11.451388	7.879243	2.631614
H	-11.224358	7.587737	4.322277
C	-10.637544	5.956143	3.075979
H	-11.322506	5.751640	2.243125
H	-11.106559	5.459328	3.934970
C	-9.222480	4.790524	1.443592
H	-8.508845	5.343330	0.825999
H	-8.906229	3.739894	1.449945
H	-10.183773	4.817856	0.915585
C	-8.916402	4.381894	3.839236
H	-8.059642	4.739556	4.416404
H	-9.721378	4.185656	4.558649
H	-8.642879	3.408642	3.412393
N	2.556099	-0.988640	7.218766
N	4.628139	0.461179	8.675272
C	1.117880	-0.898306	7.584355
H	0.845668	0.144550	7.769738
H	0.452348	-1.278327	6.799129
H	0.868514	-1.456317	8.495526
C	2.725074	-1.607562	5.876507
H	3.272119	-0.929735	5.215891
H	3.293191	-2.545879	5.902251
H	1.769730	-1.841562	5.390408
C	3.324414	-1.744211	8.251448
H	2.652257	-2.152755	9.016949
H	3.801331	-2.630883	7.814456
C	4.437515	-0.980411	9.009884
H	5.365577	-1.545888	8.856254
H	4.222011	-1.106429	10.078725
C	6.011265	0.715659	8.190521
H	6.526145	1.499096	8.760453
H	6.655187	-0.171501	8.237993
H	5.990410	1.033206	7.144263
C	4.306957	1.324589	9.842165
H	3.430249	1.938668	9.620488

H	4.069949	0.754114	10.748911
H	5.126498	2.002759	10.111084
N	-1.791248	17.561653	-3.198917
N	0.101667	16.914180	-5.323763
C	-3.159662	16.985335	-3.119242
H	-3.616126	17.102779	-2.128488
H	-3.859343	17.434037	-3.835411
H	-3.127528	15.915540	-3.343015
C	-1.410852	18.210175	-1.915968
H	-2.188355	18.123302	-1.146723
H	-0.502012	17.748543	-1.519118
H	-1.201098	19.282160	-2.019748
C	-1.683707	18.522459	-4.335647
H	-1.396865	19.520438	-3.980121
H	-2.661733	18.678208	-4.808714
C	-0.701653	18.162601	-5.478083
H	-1.294165	18.135789	-6.401637
H	-0.046579	19.033838	-5.606083
C	1.554224	17.223424	-5.245209
H	2.149281	16.674090	-5.985549
H	1.778332	18.286486	-5.398525
H	1.938459	16.962114	-4.255452
C	-0.179063	15.959933	-6.429213
H	0.726771	15.644638	-6.962400
H	-0.655570	15.059055	-6.031940
H	-0.856980	16.367473	-7.189562
C	12.132569	8.568290	2.662100
H	11.853799	8.588408	3.722974
H	11.412974	7.928147	2.144300
H	13.101827	8.056816	2.609269
C	11.839626	10.970854	3.047711
H	11.613854	10.560133	4.039805
H	12.658234	11.687490	3.190510
H	10.970548	11.549949	2.724715
C	13.461518	10.185724	1.382704
H	14.160920	9.357140	1.552799
H	13.958993	11.052803	1.835831
C	13.448215	10.439459	-0.144847
H	13.938869	11.408618	-0.301479
H	14.144484	9.714173	-0.585023
C	11.802677	11.711132	-1.450117
H	11.573219	11.638955	-2.520651
H	10.930159	12.148916	-0.956752
H	12.616162	12.441625	-1.359291
C	12.096899	9.313286	-1.857180
H	11.823309	9.674599	-2.856477
H	13.059391	8.798776	-1.969628
H	11.365203	8.551944	-1.572182
N	0.626677	3.087335	-3.931908
C	1.362843	2.611460	-2.906814
H	2.320783	2.175433	-3.152936
C	0.936051	2.658246	-1.564625
H	1.583867	2.242609	-0.803431
C	-0.322755	3.225361	-1.240081
C	-1.077746	3.728520	-2.329947
H	-2.052535	4.177099	-2.186882
C	-0.568056	3.641096	-3.640307
H	-1.145684	4.025713	-4.469183
C	-0.802305	3.285609	0.103705
C	-0.484687	2.921109	2.321281
C	0.350614	2.479567	3.391630
C	1.630206	1.916178	3.157246
H	2.023204	1.796191	2.155857
C	2.414885	1.477211	4.240395
H	3.385794	1.038615	4.057874
C	0.806828	2.113974	5.769522
H	0.499787	2.182709	6.803295
C	-0.048146	2.576829	4.749061
H	-1.008235	2.992064	5.025938
C	-2.403564	3.887157	1.594154
C	-3.670026	4.484807	1.871752
C	-4.196070	4.554118	3.185558
H	-3.660810	4.139769	4.029657
C	-4.480675	5.038621	0.849297
H	-4.174471	5.023558	-0.188950
C	-5.725840	5.613241	1.171360
H	-6.344017	6.027837	0.387425
C	-5.453100	5.149294	3.411166
H	-5.853596	5.192619	4.413743
C	-1.956909	14.052091	-1.684561
H	-2.501792	13.887895	-2.603228
C	-2.316522	13.344982	-0.520900
H	-3.146693	12.652196	-0.575551
C	-0.249751	15.151463	-0.585256
H	0.555661	15.870700	-0.630208
C	-0.530722	14.482480	0.622687
H	0.071607	14.712927	1.491732
C	-1.600565	13.553065	0.685467

C	-1.948511	12.878514	1.895011
C	-1.622224	12.529094	4.111994
C	-3.270843	11.418910	3.020761
C	-4.297168	10.425576	3.001183
C	-4.681589	9.710760	4.164172
H	-4.215025	9.900020	5.121983
C	-4.992240	10.095488	1.809755
H	-4.770079	10.582304	0.868498
C	-6.006085	9.119406	1.831665
H	-6.535452	8.873438	0.921910
C	-5.712089	8.750939	4.091303
H	-6.013878	8.214339	4.979455
C	-0.909828	12.830649	5.312860
C	0.169272	13.751399	5.332635
H	0.501250	14.257172	4.435247
C	0.818540	14.051905	6.545769
H	1.630814	14.765177	6.561968
C	-0.540831	12.604947	7.723315
H	-0.805707	12.164413	8.673715
C	-1.251324	12.247405	6.559291
H	-2.067703	11.543073	6.648260
C	8.725327	8.257173	2.240822
H	9.205174	7.483335	1.658330
C	8.459979	10.509143	2.666637
H	8.728614	11.526929	2.422366
C	7.523939	10.255676	3.689674
H	7.099145	11.096854	4.222103
C	7.169663	8.920645	4.011221
C	7.800164	7.908518	3.243397
H	7.599858	6.858413	3.412981
C	6.241489	8.610263	5.051628
C	4.843656	9.277091	6.707981
C	5.085425	7.071423	6.251385
N	2.855478	12.431682	8.829906
C	4.181202	10.326420	7.414299
C	4.436813	11.693510	7.140746
H	5.157905	11.996608	6.392774
C	3.760662	12.694508	7.864617
H	3.967874	13.735680	7.661201
C	3.226659	10.065013	8.428979
H	2.973654	9.054129	8.719913
C	2.598616	11.134574	9.097449
H	1.878820	10.931089	9.876856
C	4.718026	5.710221	6.478895
C	3.780455	5.337967	7.475398
H	3.308213	6.077014	8.109673
C	3.462633	3.978466	7.669996
H	2.752799	3.696756	8.434176
C	4.905742	3.317996	5.996100
H	5.347884	2.509365	5.431052
C	5.277658	4.648222	5.723884
H	6.011365	4.826951	4.948128
C	0.488933	13.174567	-4.265150
H	-0.484117	13.159641	-4.735679
C	2.122386	14.383789	-3.178368
H	2.452479	15.334345	-2.785246
C	2.956865	13.254609	-3.067771
H	3.921504	13.371622	-2.591173
C	1.253503	11.993321	-4.203504
H	0.841865	11.091311	-4.638251
C	2.532180	12.007667	-3.591386
C	3.345048	10.837216	-3.510801
C	5.259521	9.816125	-2.844374
C	3.638110	8.618023	-3.888709
C	6.525194	9.873682	-2.185958
C	7.050336	11.085055	-1.669640
H	6.522330	12.023802	-1.775661
C	8.296673	11.089572	-1.013632
H	8.694737	12.016794	-0.626572
C	8.564484	8.824645	-1.332994
H	9.175254	7.943123	-1.198884
C	7.329623	8.721319	-2.002575
H	7.023333	7.750271	-2.370358
C	3.153317	7.378047	-4.404991
C	3.915741	6.184852	-4.334768
H	4.903434	6.168086	-3.891992
C	3.396994	4.985360	-4.860830
H	3.982191	4.078525	-4.806134
C	1.882193	7.267253	-5.023596
H	1.227025	8.122272	-5.132119
C	1.447707	6.023972	-5.523320
H	0.479411	5.944346	-5.996857
C	-0.956618	1.214464	-6.113979
H	-1.234311	2.248480	-5.892356
H	-1.547048	0.911658	-6.987685
H	-1.303715	0.593327	-5.278906
C	1.128220	0.148007	-5.394254
H	1.901778	0.646140	-4.802885

H	0.405125	-0.286257	-4.692616
H	1.612708	-0.696990	-5.899359
C	0.809036	0.749009	-7.752360
H	1.356062	-0.201153	-7.802424
H	-0.116165	0.554054	-8.309672
C	1.619398	1.764714	-8.594815
H	2.480946	1.214798	-8.994742
H	1.011571	1.989635	-9.480549
C	3.543161	3.099579	-7.860086
H	3.879900	3.049483	-6.820825
H	4.040811	2.278605	-8.391243
H	3.938466	4.028277	-8.290409
C	1.488727	4.215690	-8.591998
H	2.253696	4.942664	-8.892441
H	0.923339	3.970990	-9.499947
H	0.795720	4.728774	-7.919262
N	0.201043	15.464088	10.116714
N	2.641129	14.349956	11.257365
C	0.112868	16.458412	9.013589
H	-0.886310	16.508595	8.563121
H	0.356244	17.479738	9.332598
H	0.818696	16.202842	8.218683
C	-1.118344	14.824173	10.362237
H	-1.897930	15.173037	9.673556
H	-1.034380	13.740412	10.243741
H	-1.501099	14.999920	11.375320
C	0.725093	16.099136	11.361338
H	-0.029864	16.075483	12.157769
H	0.917636	17.168639	11.207389
C	2.016770	15.501917	11.971477
H	2.726595	16.332789	12.073359
H	1.772223	15.231463	13.006739
C	4.033861	14.673925	10.847078
H	4.766745	13.941156	11.207408
H	4.107680	14.701650	9.756526
H	4.374834	15.651372	11.210638
C	2.605971	13.121113	12.093637
H	3.596758	12.671469	12.235701
H	2.198390	13.294136	13.097464
H	1.968401	12.367974	11.621461
N	-9.452989	8.253864	3.313198
N	-9.285392	5.385340	2.805406
N	0.510652	1.117631	-6.337239
N	2.059116	3.019422	-7.916992
N	12.149833	9.920092	2.042478
N	12.126605	10.395356	-0.837224
N	0.009795	2.800877	1.068837
N	-1.686681	3.444936	2.652112
N	-2.008441	3.862565	0.301806
N	-6.215079	5.670509	2.427367
N	2.014611	1.565126	5.524842
N	-0.950000	14.948352	-1.719825
N	-3.005500	12.040067	1.849499
N	-1.197488	13.146200	2.986596
N	-2.639334	11.640039	4.199668
N	0.470502	13.497547	7.724345
N	-6.370451	8.460604	2.949679
N	9.060653	9.531627	1.956640
N	5.714651	9.647171	5.739936
N	4.510530	8.007218	7.040215
N	5.964175	7.303154	5.251256
N	4.018148	2.979384	6.952866
N	0.909391	14.356107	-3.768298
N	4.548808	10.964434	-2.909225
N	4.850968	8.607375	-3.291365
N	2.827324	9.692487	-4.007833
N	2.187230	4.898282	-5.451234
N	9.050998	9.984100	-0.843244
Pd	3.297821	1.010409	7.094316
Pd	10.592068	9.965033	0.578216
Pd	-7.825427	6.938468	2.882118
Pd	1.346079	3.031169	-5.907485
Pd	-0.437272	15.939726	-3.505944
Pd	1.541154	13.930237	9.480321
C	-0.489238	5.872982	5.745729
C	0.741237	6.086327	5.051228
C	0.758996	6.017507	3.607506
C	-0.409394	6.474868	2.957977
C	-1.467754	6.941234	3.835184
C	-1.638236	6.308313	5.111504
H	2.773412	5.284502	3.355731
H	2.659084	5.317803	0.881408
H	1.670818	5.852601	5.564929
C	1.873105	5.618735	2.849812
C	-0.435568	6.570528	1.555654
H	-2.344974	7.366470	3.351416
H	0.634046	6.197850	-0.264778
C	0.660457	6.143691	0.818716

C	1.809737	5.652408	1.465226
H	-1.310416	6.977703	1.059415
C	-0.492534	5.424090	7.183487
H	0.498647	5.092098	7.503387
H	-0.804772	6.226233	7.862143
H	-1.195487	4.600619	7.347431
C	-2.954073	6.396129	5.837297
H	-2.897429	7.077217	6.694903
H	-3.748137	6.757950	5.178207
H	-3.261065	5.427120	6.244207
C	-0.162349	9.315595	3.252220
C	-0.530429	8.669303	4.550024
C	0.676830	8.290808	5.180963
C	1.796698	8.709853	4.290655
H	-1.404094	9.036144	5.073115
H	0.846401	8.276721	6.247908
C	1.929862	9.868987	2.007973
C	2.331435	11.302491	2.395207
C	3.132462	9.061605	1.503378
H	1.180843	9.919126	1.211175
C	3.002699	12.019234	1.217195
H	3.022740	11.248742	3.245822
H	1.441557	11.850510	2.725378
C	3.787770	9.784792	0.318960
H	3.856064	8.945467	2.314318
H	2.806883	8.059645	1.215810
C	4.199674	11.216092	0.690856
H	3.317687	13.023307	1.515813
H	2.267587	12.140730	0.407944
H	4.656352	9.207884	-0.017486
H	3.081168	9.817563	-0.522601
H	4.641300	11.729337	-0.169302
H	4.973869	11.176721	1.469669
N	1.224507	9.231463	3.126769
O	2.993734	8.621590	4.501708
O	-0.902920	9.797570	2.414181

Table5_1b_TSi_DG_0wat

Property	Value
Charge	0
Electronic Energy, BS1 (a.u.)	-1058.935925
Thermal and entropic correction, BS1 (a.u.)	2.758229
Electronic Energy, BS2 (a.u.)	-1059.283070
Number of Imaginary Frequencies	0
Imaginary frequencies (cm-1)	None

Molecular Geometry in Cartesian Coordinates

C	-9.170285	8.884582	4.630921
H	-9.983154	8.747544	5.354958
H	-8.275898	8.439278	5.075037
H	-9.000712	9.966432	4.561853
C	-9.483692	9.280306	2.233655
H	-9.272013	10.293354	2.598267
H	-8.737711	9.043627	1.470108
H	-10.451791	9.334834	1.720129
C	-10.723556	7.481033	3.350392
H	-11.448713	7.883782	2.631663
H	-11.221153	7.592431	4.322277
C	-10.636373	5.960052	3.076047
H	-11.322063	5.755994	2.243682
H	-11.105248	5.463782	3.935426
C	-9.223310	4.793462	1.442651
H	-8.509543	5.345772	0.824778
H	-8.908035	3.742527	1.448572
H	-10.184875	4.821813	0.915194
C	-8.916262	4.384039	3.838042
H	-8.058605	4.740619	4.414564
H	-9.720887	4.188648	4.558078
H	-8.644207	3.410529	3.410850
N	2.550223	-0.986426	7.210878
N	4.623699	0.453197	8.675282
C	1.111421	-0.893372	7.573394
H	0.841799	0.149544	7.762200
H	0.446693	-1.268471	6.785124
H	0.858299	-1.454162	8.481818
C	2.720700	-1.601722	5.867183
H	3.272065	-0.923704	5.210384
H	3.285444	-2.542112	5.891552
H	1.765888	-1.830729	5.377624
C	3.314114	-1.747103	8.243078
H	2.639174	-2.155802	9.006042
H	3.789360	-2.633935	7.804590

C	4.427836	-0.988613	9.005939
H	5.354486	-1.556580	8.852980
H	4.209578	-1.116593	10.073986
C	6.008268	0.704386	8.192947
H	6.524874	1.484897	8.765338
H	6.649308	-0.184923	8.239179
H	5.989743	1.024445	7.147395
C	4.303778	1.314693	9.843949
H	3.429352	1.932176	9.622690
H	4.063661	0.742674	10.748902
H	5.125145	1.989553	10.115671
N	-1.798022	17.552982	-3.196017
N	0.087478	16.916368	-5.330799
C	-3.164673	16.973096	-3.111882
H	-3.616546	17.085628	-2.118449
H	-3.868912	17.422876	-3.822901
H	-3.131152	15.904264	-3.339983
C	-1.413526	18.198967	-1.913017
H	-2.187440	18.108207	-1.140600
H	-0.501816	17.738520	-1.521422
H	-1.206813	19.271723	-2.014877
C	-1.697979	18.517124	-4.330611
H	-1.411936	19.514846	-3.973719
H	-2.678518	18.671725	-4.798832
C	-0.720309	18.162713	-5.478496
H	-1.317051	18.136264	-6.399322
H	-0.068411	19.036178	-5.607548
C	1.539364	17.229945	-5.257176
H	2.133122	16.684921	-6.001748
H	1.759480	18.294214	-5.407857
H	1.928485	16.966610	-4.269902
C	-0.194608	15.964223	-6.437726
H	0.710012	15.653706	-6.975791
H	-0.666050	15.060448	-6.040931
H	-0.877330	16.371453	-7.193919
C	12.119577	8.570363	2.666369
H	11.837352	8.590828	3.726321
H	11.401801	7.929880	2.146484
H	13.089087	8.059034	2.616809
C	11.826597	10.973163	3.050442
H	11.597410	10.562792	4.041888
H	12.645588	11.688891	3.195571
H	10.959150	11.553201	2.724767
C	13.451813	10.186860	1.389275
H	14.150484	9.357994	1.560989
H	13.948685	11.053824	1.843283
C	13.441818	10.440342	-0.138342
H	13.933349	11.409204	-0.294072
H	14.138593	9.714611	-0.576984
C	11.799492	11.713017	-1.446677
H	11.572154	11.641217	-2.517694
H	10.926190	12.151057	-0.954943
H	12.613115	12.443128	-1.354036
C	12.093592	9.315172	-1.853774
H	11.822627	9.676950	-2.853621
H	13.056009	8.800059	-1.964075
H	11.360698	8.554239	-1.570778
N	0.624390	3.079960	-3.934517
C	1.361180	2.605746	-2.909094
H	2.318875	2.169141	-3.155105
C	0.935290	2.654761	-1.566700
H	1.583574	2.240297	-0.805266
C	-0.323285	3.222431	-1.242251
C	-1.078955	3.723861	-2.332447
H	-2.053633	4.172714	-2.189469
C	-0.570121	3.634275	-3.643006
H	-1.148322	4.017493	-4.472127
C	-0.801940	3.284857	0.101763
C	-0.482454	2.924822	2.319802
C	0.353584	2.485499	3.390483
C	1.633411	1.922489	3.156445
H	2.026211	1.801539	2.155090
C	2.418607	1.485322	4.239941
H	3.389828	1.047278	4.057779
C	0.810489	2.123089	5.768711
H	0.503821	2.192964	6.802518
C	-0.044808	2.584532	4.747912
H	-1.004924	3.002889	5.024550
C	-2.402314	3.888535	1.592263
C	-3.668996	4.485661	1.869897
C	-4.194378	4.555765	3.183914
H	-3.658556	4.142252	4.028057
C	-4.480591	5.037960	0.847393
H	-4.174953	5.022090	-0.191010
C	-5.725882	5.612205	1.169702
H	-6.344714	6.025751	0.385736
C	-5.451654	5.150293	3.409695
H	-5.851714	5.194094	4.412427

C	-1.951904	14.042049	-1.690192
H	-2.497178	13.876193	-2.608330
C	-2.311828	13.338158	-0.524768
H	-3.142756	12.646163	-0.577752
C	-0.242918	15.141876	-0.594152
H	0.563663	15.859678	-0.641118
C	-0.524332	14.476167	0.615619
H	0.078576	14.707641	1.484003
C	-1.595231	13.548167	0.680950
C	-1.943777	12.876521	1.892002
C	-1.618759	12.529654	4.109571
C	-3.267704	11.419301	3.019027
C	-4.293904	10.425827	2.999648
C	-4.678102	9.710912	4.162646
H	-4.211320	9.900157	5.120357
C	-4.988976	10.095585	1.808252
H	-4.767058	10.582486	0.866991
C	-6.002717	9.119432	1.830196
H	-6.532145	8.873457	0.920478
C	-5.708397	8.750822	4.089791
H	-6.009919	8.214019	4.977915
C	-0.906240	12.830408	5.310625
C	0.175036	13.748643	5.330727
H	0.508610	14.253201	4.433256
C	0.824396	14.047978	6.544218
H	1.638355	14.759297	6.560745
C	-0.539151	12.604593	7.721350
H	-0.805577	12.164924	8.671711
C	-1.249770	12.248343	6.557105
H	-2.067752	11.545813	6.646223
C	8.716722	8.258833	2.240453
H	9.194419	7.485216	1.655885
C	8.451058	10.510615	2.666459
H	8.717145	11.528600	2.420177
C	7.520263	10.256638	3.694171
H	7.096784	11.097546	4.228043
C	7.168869	8.921408	4.017962
C	7.796802	7.909606	3.247628
H	7.598215	6.859366	3.418382
C	6.244981	8.610532	5.062034
C	4.847719	9.276740	6.719172
C	5.089778	7.071157	6.261954
N	2.856502	12.431819	8.838001
C	4.184669	10.326103	7.424958
C	4.443891	11.693165	7.154652
H	5.169344	11.996204	6.410892
C	3.766307	12.694286	7.876971
H	3.976258	13.735344	7.675815
C	3.225780	10.064943	8.435619
H	2.969835	9.054088	8.724099
C	2.596625	11.134699	9.102837
H	1.873456	10.931413	9.879188
C	4.722723	5.709716	6.488615
C	3.783748	5.336703	7.483460
H	3.310254	6.075348	8.117239
C	3.466182	3.976984	7.677006
H	2.755280	3.694692	8.439995
C	4.912206	3.317707	6.005286
H	5.355698	2.509481	5.440718
C	5.284031	4.648168	5.734191
H	6.019002	4.827375	4.959755
C	0.485900	13.172470	-4.278198
H	-0.487773	13.156496	-4.747394
C	2.121991	14.384464	-3.199290
H	2.453794	15.336516	-2.811566
C	2.955551	13.255046	-3.084835
H	3.921079	13.373198	-2.610335
C	1.249615	11.990796	-4.213174
H	0.836653	11.087420	-4.643809
C	2.529067	12.006376	-3.602591
C	3.341264	10.835729	-3.518058
C	5.255375	9.815570	-2.848995
C	3.634185	8.615888	-3.891940
C	6.520975	9.873818	-2.190440
C	7.046411	11.085753	-1.675772
H	6.518851	12.024552	-1.783550
C	8.292231	11.090687	-1.018749
H	8.690380	12.018274	-0.632728
C	8.559474	8.825327	-1.334863
H	9.169913	7.943797	-1.199278
C	7.324988	8.721481	-2.005041
H	7.018674	7.750010	-2.371670
C	3.149723	7.375216	-4.406835
C	3.912523	6.182322	-4.335412
H	4.900248	6.166321	-3.892689
C	3.394138	4.982148	-4.860255
H	3.979568	4.075514	-4.804674
C	1.878600	7.263453	-5.025270

H	1.223159	8.118167	-5.134556
C	1.444413	6.019543	-5.523652
H	0.476054	5.939079	-5.996920
C	-0.955212	1.208271	-6.120040
H	-1.233982	2.241723	-5.897133
H	-1.544618	0.906217	-6.994698
H	-1.302511	0.585639	-5.286163
C	1.129961	0.142916	-5.399680
H	1.902518	0.641102	-4.807042
H	0.406648	-0.292791	-4.699163
H	1.615671	-0.701064	-5.905313
C	0.812325	0.746241	-7.757409
H	1.360053	-0.203489	-7.808016
H	-0.112208	0.551281	-8.315829
C	1.622840	1.763433	-8.597933
H	2.485461	1.214702	-8.997170
H	1.015966	1.988497	-9.484286
C	3.544282	3.099999	-7.860175
H	3.880159	3.048969	-6.820682
H	4.043454	2.280356	-8.391957
H	3.938796	4.029773	-8.288917
C	1.489103	4.214237	-8.592828
H	2.253445	4.942373	-8.892036
H	0.924709	3.969700	-9.501436
H	0.794965	4.725895	-7.920183
N	0.196720	15.465821	10.110537
N	2.632654	14.353684	11.261958
C	0.111747	16.457881	9.005126
H	-0.886067	16.506978	8.551533
H	0.353981	17.479890	9.322831
H	0.820006	16.200820	8.212866
C	-1.123224	14.826050	10.353329
H	-1.900719	15.172943	9.661283
H	-1.038421	13.742051	10.237813
H	-1.509253	15.004031	11.364778
C	0.716674	16.103565	11.355467
H	-0.041532	16.083047	12.148892
H	0.911296	17.172394	11.199459
C	2.004889	15.506017	11.972516
H	2.714411	16.336717	12.077784
H	1.754939	15.236031	13.006611
C	4.026753	14.678042	10.856656
H	4.758646	13.946032	11.220489
H	4.104759	14.704755	9.766404
H	4.365789	15.656047	11.220515
C	2.595009	13.125707	12.099415
H	3.585653	12.677426	12.246678
H	2.182506	13.299357	13.101120
H	1.960632	12.371232	11.625039
N	-9.449661	8.256766	3.312205
N	-9.284901	5.388046	2.804630
N	0.512341	1.113015	-6.342141
N	2.060382	3.018021	-7.918483
N	12.138616	9.921930	2.046291
N	12.121611	10.396951	-0.833455
N	0.010964	2.801966	1.067182
N	-1.684467	3.448627	2.650487
N	-2.008010	3.862070	0.299701
N	-6.214485	5.670295	2.425942
N	2.018417	1.574504	5.524324
N	-0.943939	14.936904	-1.728018
N	-3.001646	12.039112	1.847346
N	-1.192898	13.145317	2.983611
N	-2.637055	11.641937	4.198006
N	0.474307	13.494682	7.722738
N	-6.366870	8.460513	2.948186
N	9.049405	9.533465	1.953944
N	5.719521	9.647099	5.751933
N	4.513134	8.006793	7.049831
N	5.969105	7.303284	5.262454
N	4.023167	2.978308	6.960421
N	0.907895	14.355781	-3.786761
N	4.544583	10.963781	-2.915535
N	4.846931	8.606192	-3.294383
N	2.823448	9.690143	-4.012958
N	2.184356	4.894219	-5.450462
N	9.046070	9.985211	-0.846212
Pd	3.297457	1.010985	7.094101
Pd	10.583947	9.966804	0.578732
Pd	-7.823428	6.939786	2.880972
Pd	1.345574	3.026800	-5.909552
Pd	-0.441319	15.935217	-3.513579
Pd	1.539350	13.931123	9.481381
C	-0.495238	5.886750	5.754205
C	0.763534	6.234992	5.062062
C	0.764868	6.030954	3.598393
C	-0.396502	6.484488	2.953885
C	-1.407395	7.071243	3.879847

C	-1.624031	6.311395	5.135164
H	2.778315	5.297556	3.356870
H	2.656048	5.315133	0.876159
H	1.684988	5.954103	5.567390
C	1.870032	5.619219	2.852113
C	-0.439521	6.564969	1.560843
H	-2.307711	7.431082	3.379821
H	0.627847	6.187690	-0.264552
C	0.660799	6.135071	0.819088
C	1.805449	5.649471	1.460912
H	-1.312929	6.978715	1.065319
C	-0.478169	5.413074	7.182765
H	0.515628	5.063276	7.482675
H	-0.765744	6.212873	7.871698
H	-1.188957	4.596593	7.351518
C	-2.954113	6.377677	5.837675
H	-2.912366	7.063027	6.693330
H	-3.743090	6.732739	5.170199
H	-3.256285	5.410356	6.246916
C	-0.154638	9.316013	3.254308
C	-0.570406	8.562462	4.496703
C	0.698935	8.162291	5.162695
C	1.810465	8.697255	4.297974
H	-1.337909	9.094415	5.091421
H	0.844160	8.352243	6.236270
C	1.946478	9.879492	2.023110
C	2.365121	11.305108	2.418052
C	3.137793	9.063344	1.506243
H	1.191337	9.944432	1.233590
C	3.038838	12.025167	1.243362
H	3.059550	11.236392	3.265036
H	1.482600	11.862777	2.757043
C	3.792196	9.789654	0.321938
H	3.863734	8.932378	2.312903
H	2.796770	8.067545	1.211492
C	4.224040	11.213464	0.703422
H	3.366379	13.022880	1.549975
H	2.301704	12.162970	0.438496
H	4.652972	9.203492	-0.020628
H	3.084881	9.836994	-0.515688
H	4.665462	11.729122	-0.155422
H	5.002513	11.159282	1.477119
N	1.235882	9.245656	3.148046
O	3.007339	8.619517	4.504501
O	-0.883535	9.814451	2.422544

Table5_1b_TSii_reactant_0wat

Property	Value
Charge	0
Electronic Energy, BS1 (a.u.)	-1058.925701
Thermal and entropic correction, BS1 (a.u.)	2.746485
Electronic Energy, BS2 (a.u.)	-1059.282491
Number of Imaginary Frequencies	0
Imaginary frequencies (cm-1)	None

Molecular Geometry in Cartesian Coordinates

C	-8.123722	2.421941	6.824114
C	-8.972021	2.506051	5.713877
C	-8.578702	3.193274	4.521757
C	-7.598875	4.207762	4.668276
C	-7.100970	4.428220	5.993526
C	-7.144073	3.424499	6.965265
H	-9.883480	2.159193	3.141696
H	-9.077517	3.403316	1.163316
H	-9.822715	1.832745	5.657514
C	-9.124813	2.927257	3.246604
C	-7.185349	4.943649	3.534866
H	-6.457593	5.288545	6.163547
H	-7.367041	5.188758	1.413898
C	-7.698488	4.639611	2.287406
C	-8.672767	3.624125	2.141669
H	-6.447792	5.733451	3.650507
C	-8.358138	1.355913	7.865389
H	-7.440965	0.796073	8.077467
H	-8.709064	1.767168	8.814431
H	-9.120453	0.649110	7.526556
C	-6.212169	3.519481	8.147077
H	-6.289911	4.507481	8.614560
H	-6.413041	2.774352	8.916387
H	-5.174704	3.377075	7.837489
C	-10.449177	3.928242	7.811223
C	-10.122081	4.456167	6.461401

C	-9.187584	5.422150	6.594473
C	-8.870271	5.581227	8.038015
H	-10.783073	4.261678	5.612818
H	-8.910531	6.199763	5.878400
C	-9.769965	4.509015	10.173210
C	-8.441187	4.182017	10.862375
C	-10.406501	5.780274	10.762320
H	-10.461666	3.671901	10.316810
C	-8.636327	4.052995	12.380924
H	-7.721607	4.977754	10.645176
H	-8.039168	3.251035	10.451914
C	-10.591764	5.653480	12.281109
H	-9.748908	6.631152	10.536989
H	-11.371542	5.954798	10.262825
C	-9.261228	5.322586	12.971868
H	-7.672580	3.842519	12.854751
H	-9.285737	3.193018	12.592850
H	-11.010158	6.580868	12.687204
H	-11.318345	4.857920	12.491603
H	-9.419718	5.200508	14.047846
H	-8.567359	6.164416	12.846347
N	-9.636783	4.631914	8.716709
O	-8.117966	6.388747	8.567456
O	-11.261152	3.076604	8.131744
C	-12.444459	-3.627846	7.438912
H	-12.370888	-4.287261	8.312771
H	-11.785098	-2.777240	7.631648
H	-13.474651	-3.250289	7.424344
C	-13.186314	-4.306793	5.204708
H	-14.079578	-3.798482	5.588755
H	-12.896592	-3.804123	4.277833
H	-13.505813	-5.318961	4.926601
C	-11.557270	-5.678638	6.422516
H	-12.157448	-6.417523	5.876167
H	-11.690933	-5.953145	7.476758
C	-10.076936	-5.975701	6.080276
H	-10.081817	-6.837568	5.400821
H	-9.620129	-6.364129	6.999628
C	-8.747603	-5.205590	4.166695
H	-9.189782	-4.547551	3.413631
H	-7.657746	-5.107076	4.088525
H	-8.984649	-6.233518	3.864750
C	-8.152901	-4.493138	6.433313
H	-8.298968	-3.477304	6.809146
H	-8.084769	-5.147411	7.311376
H	-7.169657	-4.526916	5.947399
N	0.232906	5.794506	6.602359
N	-0.835952	8.379033	7.437516
C	-0.043089	4.521947	7.320501
H	-1.105301	4.457201	7.570223
H	0.211138	3.633328	6.729138
H	0.508567	4.430423	8.264467
C	0.816245	5.530098	5.259943
H	0.181422	5.966890	4.483807
H	1.816117	5.962840	5.130265
H	0.913666	4.459761	5.039330
C	1.126144	6.678390	7.407498
H	1.461835	6.167720	8.319139
H	2.055697	6.893853	6.865126
C	0.555781	8.045156	7.859358
H	1.263961	8.806953	7.508935
H	0.651233	8.074605	8.952393
C	-0.853028	9.585772	6.568269
H	-1.526483	10.369377	6.937363
H	0.133189	10.054405	6.460532
H	-1.184055	9.316740	5.561318
C	-1.719815	8.563907	8.618647
H	-2.483307	7.782252	8.638480
H	-1.182543	8.504577	9.573492
H	-2.238514	9.530791	8.617595
N	-20.018297	3.724508	-1.877936
N	-19.017552	4.902950	-4.353377
C	-19.793482	2.310031	-1.477109
H	-19.990757	2.131196	-0.412746
H	-20.421046	1.599696	-2.029773
H	-18.754516	2.027387	-1.670034
C	-20.502471	4.533061	-0.727566
H	-20.576031	3.953570	0.201193
H	-19.818060	5.365617	-0.542173
H	-21.493744	4.972397	-0.895337
C	-20.966532	3.810119	-3.027007
H	-21.862923	4.380755	-2.752190
H	-21.352242	2.817078	-3.290871
C	-20.435628	4.437298	-4.340197
H	-20.611030	3.695952	-5.130227
H	-21.118783	5.259528	-4.588831
C	-18.940362	6.369555	-4.587079
H	-18.300699	6.633947	-5.438493

H	-19.917900	6.827494	-4.782788
H	-18.530855	6.866908	-3.703245
C	-18.223799	4.160834	-5.368662
H	-17.710678	4.822792	-6.077576
H	-17.460249	3.554145	-4.873912
H	-18.829305	3.473552	-5.972728
C	-7.417848	16.111194	0.025034
H	-7.359030	16.181611	1.118420
H	-7.006218	15.139914	-0.263593
H	-6.734152	16.873158	-0.369553
C	-9.763621	16.494866	0.619908
H	-9.282929	16.497044	1.606164
H	-10.288471	17.454267	0.531802
H	-10.532003	15.716919	0.630215
C	-8.890826	17.326584	-1.517703
H	-7.927295	17.840935	-1.625471
H	-9.581824	18.117116	-1.198178
C	-9.329482	16.914426	-2.944776
H	-10.207358	17.526383	-3.188578
H	-8.550370	17.281588	-3.625027
C	-11.013608	15.259124	-3.616884
H	-11.094374	14.708088	-4.562224
H	-11.555071	14.687089	-2.858069
H	-11.565544	16.196782	-3.758734
C	-8.656702	14.904376	-4.184082
H	-9.162237	14.424596	-5.031430
H	-7.983427	15.656952	-4.613174
H	-8.022679	14.149276	-3.710882
N	-5.546440	2.321190	-3.560678
C	-4.793262	3.119100	-2.776593
H	-4.164327	3.843298	-3.274754
C	-4.798251	3.043868	-1.370027
H	-4.155864	3.717006	-0.817097
C	-5.627555	2.099223	-0.713374
C	-6.420355	1.276777	-1.553748
H	-7.085615	0.525258	-1.148385
C	-6.348129	1.422774	-2.953135
H	-6.955898	0.797512	-3.591513
C	-5.657710	1.980104	0.709093
C	-4.907299	2.627127	2.750679
C	-4.090341	3.486029	3.546888
C	-3.261309	4.479298	2.967803
H	-3.207954	4.621107	1.895965
C	-2.465720	5.300003	3.789813
H	-1.827552	6.048624	3.341596
C	-3.228954	4.266411	5.700749
H	-3.195857	4.191117	6.777877
C	-4.055316	3.396525	4.960582
H	-4.644153	2.659556	5.490746
C	-6.437937	0.961973	2.581598
C	-7.290574	-0.006702	3.193156
C	-7.341490	-0.188590	4.598075
H	-6.726947	0.399843	5.264181
C	-8.134494	-0.849480	2.426992
H	-8.161784	-0.795877	1.346200
C	-8.949452	-1.801222	3.068407
H	-9.582758	-2.449657	2.479378
C	-8.194357	-1.165100	5.151804
H	-8.225680	-1.306216	6.222736
C	-16.512203	3.037841	-0.372338
H	-16.528614	2.288472	-1.150684
C	-15.775645	2.809789	0.806125
H	-15.241314	1.873831	0.906062
C	-17.202298	5.113606	0.364485
H	-17.765412	6.015736	0.172516
C	-16.496874	4.972683	1.575394
H	-16.541749	5.780262	2.294774
C	-15.757436	3.789916	1.830804
C	-15.046637	3.592040	3.052910
C	-14.477986	4.301673	5.132478
C	-13.773199	2.275233	4.394031
C	-13.068120	1.052927	4.613418
C	-12.431680	0.759829	5.846093
H	-12.465803	1.451672	6.677428
C	-12.977629	0.051751	3.613729
H	-13.437992	0.175765	2.641565
C	-12.290319	-1.147844	3.879555
H	-12.231234	-1.913356	3.118663
C	-11.760562	-0.467192	6.020035
H	-11.280724	-0.687376	6.963046
C	-14.544804	5.289150	6.161257
C	-15.207490	6.527891	5.972209
H	-15.691004	6.775360	5.035632
C	-15.249224	7.470031	7.018178
H	-15.758063	8.412213	6.870659
C	-14.050411	6.087924	8.421272
H	-13.608437	5.924002	9.393065
C	-13.959868	5.086033	7.435842

H	-13.462759	4.159060	7.686040
C	-7.900558	12.673988	0.664460
H	-7.079401	12.868011	-0.010493
C	-10.107487	13.046437	1.225873
H	-11.043370	13.535974	0.997497
C	-10.015048	12.203881	2.350824
H	-10.893588	12.070259	2.968917
C	-8.788487	11.560809	2.654546
C	-7.714539	11.814399	1.764113
H	-6.738930	11.368693	1.909149
C	-8.639028	10.717342	3.796326
C	-9.491968	9.799377	5.689118
C	-7.329622	9.417588	5.119063
N	-12.763050	9.339579	8.420515
C	-10.582822	9.636115	6.595714
C	-11.856363	10.205904	6.343961
H	-12.054076	10.784197	5.450329
C	-12.901119	10.031144	7.270977
H	-13.869578	10.470066	7.076806
C	-10.455981	8.907089	7.804832
H	-9.515226	8.460379	8.096573
C	-11.558934	8.786140	8.673455
H	-11.455569	8.241212	9.599610
C	-6.056998	8.831636	5.394952
C	-5.815559	8.063924	6.562074
H	-6.591307	7.898346	7.297941
C	-4.535014	7.524271	6.799111
H	-4.353885	6.943520	7.692687
C	-3.707358	8.428396	4.842979
H	-2.865600	8.564697	4.178717
C	-4.952265	9.001007	4.522283
H	-5.030994	9.576460	3.608514
C	-15.269741	4.518514	-3.428435
H	-15.533165	3.500377	-3.676675
C	-15.941822	6.588980	-2.666982
H	-16.741869	7.220975	-2.309249
C	-14.638633	7.109516	-2.781933
H	-14.471034	8.143401	-2.508699
C	-13.937824	4.950832	-3.578685
H	-13.204497	4.244629	-3.946431
C	-13.586650	6.285897	-3.256131
C	-12.253401	6.773681	-3.402918
C	-10.748314	8.461388	-3.217989
C	-10.089526	6.434121	-3.995203
C	-10.445283	9.813665	-2.876424
C	-11.436252	10.706003	-2.394805
H	-12.464607	10.394791	-2.263011
C	-11.093502	12.035702	-2.083153
H	-11.852268	12.714153	-1.719973
C	-8.888832	11.689139	-2.663996
H	-7.890712	12.090672	-2.763337
C	-9.137100	10.344893	-3.002750
H	-8.312504	9.742225	-3.361365
C	-9.064107	5.571749	-4.487553
C	-7.722093	6.004855	-4.631370
H	-7.422362	7.014303	-4.380406
C	-6.742284	5.112250	-5.107964
H	-5.720234	5.447097	-5.213474
C	-9.325926	4.227875	-4.854368
H	-10.319406	3.803782	-4.784161
C	-8.279820	3.410311	-5.323237
H	-8.485754	2.386621	-5.601171
C	-4.402967	-0.273306	-5.296267
H	-5.456844	-0.164719	-5.025493
H	-4.345588	-1.104295	-6.010332
H	-3.864204	-0.601822	-4.398597
C	-2.730414	1.499309	-5.050164
H	-2.937556	2.503340	-4.670210
H	-2.503530	0.860986	-4.187188
H	-1.803401	1.569058	-5.632748
C	-3.516664	0.859872	-7.284649
H	-2.451199	1.073274	-7.438884
H	-3.631199	-0.181057	-7.613125
C	-4.287559	1.728350	-8.309243
H	-3.531978	2.300529	-8.862706
H	-4.696546	1.034762	-9.055095
C	-5.051862	4.050490	-8.088519
H	-4.880852	4.605104	-7.161589
H	-4.151553	4.177794	-8.702628
H	-5.864940	4.553663	-8.626749
C	-6.688122	2.241281	-8.313547
H	-7.208861	3.072443	-8.805325
H	-6.639839	1.429488	-9.049946
H	-7.329043	1.889584	-7.499993
N	-16.170345	8.344623	10.720523
N	-14.177045	10.457738	10.945754
C	-17.326832	8.331791	9.784871
H	-17.772310	7.335824	9.669436

H	-18.141790	8.997701	10.095492
H	-17.010532	8.666484	8.793081
C	-15.939273	6.993809	11.297403
H	-16.621369	6.234613	10.894378
H	-14.917633	6.666052	11.082981
H	-16.059435	6.965063	12.387540
C	-16.370093	9.359233	11.796332
H	-16.423816	8.877165	12.780879
H	-17.341370	9.859367	11.690432
C	-15.308464	10.479963	11.918530
H	-15.852700	11.431617	11.866895
H	-14.936097	10.440544	12.950270
C	-14.140346	11.711928	10.146840
H	-13.165849	12.214413	10.185129
H	-14.356481	11.495748	9.096927
H	-14.879899	12.453536	10.473898
C	-12.881372	10.231847	11.639656
H	-12.131216	10.997066	11.403085
H	-12.975168	10.222783	12.732533
H	-12.466172	9.261530	11.350092
N	-12.053352	-4.294582	6.168739
N	-9.265600	-4.856896	5.517066
N	-3.886594	1.008182	-5.846358
N	-5.354648	2.627286	-7.779600
N	-8.806048	16.247571	-0.490606
N	-9.605270	15.467618	-3.186628
N	-4.863881	2.816037	1.412958
N	-5.669734	1.717370	3.399760
N	-6.485624	1.050160	1.233147
N	-8.983759	-1.966732	4.406298
N	-2.440069	5.203618	5.135026
N	-17.220328	4.163830	-0.592982
N	-14.373506	2.428029	3.193334
N	-15.115703	4.578883	3.973879
N	-13.768307	3.180925	5.397411
N	-14.686279	7.261766	8.226555
N	-11.693688	-1.412677	5.059573
N	-9.070787	13.288466	0.397287
N	-9.717660	10.558757	4.594102
N	-8.330532	9.175723	5.996120
N	-7.424989	10.158313	3.993575
N	-3.492628	7.706988	5.961168
N	-16.262473	5.318687	-2.987214
N	-12.028844	8.060547	-3.057092
N	-9.730960	7.697743	-3.675556
N	-11.329666	5.909075	-3.878079
N	-7.005673	3.834568	-5.453300
N	-9.845309	12.528988	-2.216956
Pd	-1.642661	6.764639	6.290517
Pd	-9.334783	14.378816	-1.375715
Pd	-10.492971	-3.126184	5.294813
Pd	-5.446734	2.449458	-5.657570
Pd	-18.126063	4.521563	-2.456003
Pd	-14.443838	8.847034	9.577215

Table5_1b_TSii_TSi-ii_0wat

Property	Value
Charge	0
Electronic Energy, BS1 (a.u.)	-1058.886552
Thermal and entropic correction, BS1 (a.u.)	2.752297
Electronic Energy, BS2 (a.u.)	-1059.239483
Number of Imaginary Frequencies	0
Imaginary frequencies (cm-1)	None

Molecular Geometry in Cartesian Coordinates

C	-8.115559	2.432185	6.835483
C	-9.047189	2.607768	5.755834
C	-8.583366	3.203485	4.514784
C	-7.607657	4.212762	4.658624
C	-7.213050	4.496021	6.027595
C	-7.155744	3.414203	6.973987
H	-9.884688	2.160183	3.143068
H	-9.072624	3.398772	1.158238
H	-9.864038	1.894544	5.681297
C	-9.117650	2.922817	3.248254
C	-7.182854	4.937684	3.534167
H	-6.526924	5.325783	6.182368
H	-7.363440	5.185153	1.409280
C	-7.696362	4.631284	2.279941
C	-8.663778	3.621283	2.136946
H	-6.452052	5.734146	3.649979
C	-8.350981	1.345572	7.856372

H	-7.434574	0.794425	8.085070
H	-8.731029	1.747706	8.800581
H	-9.100711	0.634021	7.500891
C	-6.206725	3.518358	8.138863
H	-6.292904	4.501904	8.612011
H	-6.388384	2.764647	8.903691
H	-5.166446	3.395882	7.817849
C	-10.445611	3.920795	7.807985
C	-10.057144	4.351130	6.434232
C	-9.074837	5.364989	6.573537
C	-8.859719	5.579293	8.035842
H	-10.800858	4.323639	5.650005
H	-8.975028	6.211423	5.907819
C	-9.775698	4.513004	10.170127
C	-8.443783	4.179932	10.857554
C	-10.406871	5.784316	10.764526
H	-10.468509	3.676713	10.310108
C	-8.637932	4.048668	12.376025
H	-7.723301	4.975095	10.641226
H	-8.043379	3.249141	10.444995
C	-10.590994	5.652411	12.283289
H	-9.748346	6.631667	10.539881
H	-11.372191	5.960902	10.266255
C	-9.260408	5.318294	12.972429
H	-7.674142	3.835971	12.848768
H	-9.288486	3.189341	12.587057
H	-11.007595	6.579815	12.691158
H	-11.318653	4.857659	12.493124
H	-9.418248	5.194764	14.048335
H	-8.565311	6.159225	12.847701
N	-9.646261	4.640498	8.707745
O	-8.122361	6.392411	8.571767
O	-11.266568	3.081236	8.134762
C	-12.443716	-3.626458	7.439194
H	-12.370762	-4.286399	8.312706
H	-11.783161	-2.776822	7.632139
H	-13.473411	-3.247528	7.425110
C	-13.187241	-4.303843	5.205068
H	-14.079863	-3.794875	5.589733
H	-12.897473	-3.801067	4.278271
H	-13.507837	-5.315588	4.926686
C	-11.559074	-5.677778	6.421708
H	-12.159870	-6.415857	5.874948
H	-11.693077	-5.952655	7.475812
C	-10.078958	-5.976003	6.079520
H	-10.084495	-6.837528	5.399638
H	-9.622637	-6.365289	6.998751
C	-8.747951	-5.206404	4.166902
H	-9.189245	-4.547957	3.413683
H	-7.657984	-5.108560	4.089403
H	-8.985457	-6.234129	3.864630
C	-8.154121	-4.494813	6.434020
H	-8.299666	-3.478907	6.809872
H	-8.087038	-5.149228	7.312060
H	-7.170602	-4.529273	5.948707
N	0.232761	5.794545	6.602133
N	-0.835862	8.379058	7.437648
C	-0.043331	4.521831	7.319967
H	-1.105540	4.457114	7.569708
H	0.210791	3.633337	6.728371
H	0.508349	4.430019	8.263892
C	0.816120	5.530412	5.259674
H	0.181342	5.967423	4.483625
H	1.816021	5.963126	5.130124
H	0.913480	4.460119	5.038820
C	1.126030	6.678180	7.407510
H	1.461563	6.167317	8.319101
H	2.055665	6.893606	6.865263
C	0.555806	8.044956	7.859524
H	1.264103	8.806713	7.509251
H	0.651185	8.074249	8.952570
C	-0.852751	9.585878	6.568506
H	-1.526129	10.369532	6.937638
H	0.133525	10.054403	6.460850
H	-1.183765	9.316979	5.561518
C	-1.719734	8.563940	8.618772
H	-2.483374	7.782423	8.638480
H	-1.182495	8.504376	9.573620
H	-2.238254	9.530919	8.617838
N	-20.017778	3.724582	-1.877737
N	-19.017072	4.902298	-4.353514
C	-19.792991	2.310163	-1.476698
H	-19.990234	2.131497	-0.412302
H	-20.420605	1.599771	-2.029228
H	-18.754041	2.027455	-1.669631
C	-20.501853	4.533337	-0.727473
H	-20.575227	3.954042	0.201423
H	-19.817486	5.365994	-0.542375

H	-21.493189	4.972547	-0.895203
C	-20.966087	3.810036	-3.026756
H	-21.862404	4.380814	-2.751993
H	-21.351906	2.816975	-3.290386
C	-20.435219	4.436886	-4.340111
H	-20.610834	3.695425	-5.129985
H	-21.118248	5.259194	-4.588835
C	-18.939704	6.368805	-4.587766
H	-18.299995	6.632801	-5.439269
H	-19.917195	6.826769	-4.783664
H	-18.530164	6.866457	-3.704114
C	-18.223470	4.159684	-5.368553
H	-17.710357	4.821299	-6.077794
H	-17.459934	3.553150	-4.873592
H	-18.829072	3.472174	-5.972264
C	-7.418425	16.114062	0.023407
H	-7.359600	16.185888	1.116703
H	-7.006343	15.142605	-0.263976
H	-6.735099	16.875841	-0.372174
C	-9.764340	16.497326	0.617904
H	-9.283559	16.501181	1.604115
H	-10.289852	17.456246	0.528525
H	-10.532179	15.718859	0.629391
C	-8.892028	17.326827	-1.520805
H	-7.928751	17.841524	-1.629191
H	-9.583423	18.117409	-1.202263
C	-9.330433	16.912689	-2.947391
H	-10.208470	17.524070	-3.192054
H	-8.551368	17.279196	-3.628050
C	-11.014146	15.256000	-3.617129
H	-11.094922	14.703578	-4.561661
H	-11.555362	14.684940	-2.857406
H	-11.566340	16.193313	-3.760249
C	-8.657208	14.901316	-4.184298
H	-9.162724	14.420064	-5.030822
H	-7.984450	15.653637	-4.614652
H	-8.022639	14.147233	-3.710213
N	-5.545262	2.320529	-3.560965
C	-4.792238	3.118567	-2.776858
H	-4.163483	3.842931	-3.275007
C	-4.797167	3.043263	-1.370293
H	-4.154957	3.716557	-0.817348
C	-5.626274	2.098427	-0.713656
C	-6.418828	1.275774	-1.554055
H	-7.083887	0.524071	-1.148703
C	-6.346701	1.421877	-2.953439
H	-6.954361	0.796516	-3.591821
C	-5.656580	1.979398	0.708817
C	-4.906974	2.627086	2.750480
C	-4.090376	3.486285	3.546731
C	-3.261237	4.479482	2.967664
H	-3.207667	4.621128	1.895816
C	-2.465821	5.300323	3.789711
H	-1.827591	6.048907	3.341523
C	-3.229391	4.266937	5.700626
H	-3.196317	4.191792	6.777753
C	-4.055765	3.397051	4.960448
H	-4.644857	2.660126	5.490415
C	-6.437201	0.961566	2.581325
C	-7.289753	-0.007208	3.192853
C	-7.340630	-0.189203	4.597761
H	-6.726199	0.402259	5.263934
C	-8.133540	-0.850081	2.426649
H	-8.160884	-0.796365	1.345866
C	-8.948299	-1.802044	3.067999
H	-9.581508	-2.450509	2.478905
C	-8.193054	-1.166182	5.151356
H	-8.224024	-1.307745	6.222229
C	-16.511610	3.038046	-0.372188
H	-16.527674	2.288761	-1.150621
C	-15.775494	2.809938	0.806535
H	-15.241135	1.874006	0.906572
C	-17.202111	5.113713	0.364566
H	-17.765171	6.015843	0.172448
C	-16.497169	4.972707	1.575751
H	-16.542348	5.780212	2.295194
C	-15.757743	3.789968	1.831320
C	-15.047349	3.592012	3.053648
C	-14.479605	4.301244	5.133604
C	-13.773952	2.275196	4.394811
C	-13.068179	1.053206	4.613787
C	-12.431843	0.759853	5.846461
H	-12.466598	1.451249	6.678151
C	-12.976733	0.052653	3.613547
H	-13.436921	0.176899	2.641330
C	-12.288595	-1.146589	3.878816
H	-12.228731	-1.911630	3.117501
C	-11.759825	-0.466769	6.019843

H	-11.279938	-0.687087	6.962797
C	-14.546488	5.288712	6.162410
C	-15.208728	6.527673	5.973240
H	-15.692062	6.775259	5.036599
C	-15.250123	7.469932	7.019126
H	-15.758583	8.412305	6.871501
C	-14.051943	6.087485	8.422415
H	-13.610054	5.923455	9.394235
C	-13.961843	5.085443	7.437096
H	-13.465279	4.158229	7.687436
C	-7.899765	12.676548	0.666389
H	-7.078399	12.871007	-0.008183
C	-10.106840	13.048521	1.227374
H	-11.042688	13.538137	0.999039
C	-10.014651	12.205417	2.351936
H	-10.893336	12.071444	2.969746
C	-8.788110	11.562349	2.655714
C	-7.713963	11.816443	1.765670
H	-6.738335	11.370799	1.910772
C	-8.638824	10.718486	3.797223
C	-9.491932	9.800018	5.689680
C	-7.329511	9.418448	5.119785
N	-12.763342	9.338961	8.420447
C	-10.582898	9.636364	6.596067
C	-11.856642	10.205595	6.344039
H	-12.054426	10.783742	5.450325
C	-12.901529	10.030377	7.270833
H	-13.870162	10.468821	7.076443
C	-10.455969	8.907497	7.805267
H	-9.515037	8.461359	8.097291
C	-11.559031	8.786089	8.673677
H	-11.455558	8.241288	9.599886
C	-6.056945	8.832310	5.395539
C	-5.815601	8.064152	6.562397
H	-6.591427	7.898243	7.298127
C	-4.535074	7.524361	6.799272
H	-4.353966	6.943271	7.692638
C	-3.707332	8.429096	4.843450
H	-2.865570	8.565537	4.179221
C	-4.952195	9.001883	4.522930
H	-5.030873	9.577607	3.609327
C	-15.269338	4.518475	-3.428820
H	-15.532781	3.500367	-3.677158
C	-15.941355	6.588868	-2.667126
H	-16.741386	7.220839	-2.309315
C	-14.638158	7.109382	-2.782038
H	-14.470542	8.143234	-2.508688
C	-13.937413	4.950783	-3.579069
H	-13.204103	4.244614	-3.946915
C	-13.586197	6.285791	-3.256342
C	-12.252929	6.773549	-3.403035
C	-10.747764	8.461120	-3.217493
C	-10.089021	6.434033	-3.995233
C	-10.444659	9.813197	-2.875190
C	-11.435615	10.705323	-2.393119
H	-12.464001	10.394109	-2.261573
C	-11.092783	12.034772	-2.080515
H	-11.851465	12.713048	-1.716821
C	-8.888019	11.688360	-2.661378
H	-7.889838	12.089866	-2.760197
C	-9.136398	10.344366	-3.001049
H	-8.311818	9.741815	-3.359899
C	-9.063572	5.571673	-4.487551
C	-7.721493	6.004695	-4.631048
H	-7.421738	7.014095	-4.379923
C	-6.741626	5.112041	-5.107434
H	-5.719515	5.446801	-5.212638
C	-9.325412	4.227850	-4.854552
H	-10.318938	3.803828	-4.784575
C	-8.279244	3.410213	-5.323175
H	-8.485173	2.386537	-5.601160
C	-4.402246	-0.273554	-5.297753
H	-5.456093	-0.165067	-5.026816
H	-4.344945	-1.104263	-6.012149
H	-3.863380	-0.602414	-4.400270
C	-2.729685	1.498980	-5.051142
H	-2.936775	2.502866	-4.670780
H	-2.502700	0.860326	-4.188437
H	-1.802737	1.568950	-5.633805
C	-3.516155	0.860426	-7.285784
H	-2.450764	1.074151	-7.440084
H	-3.630468	-0.180423	-7.614596
C	-4.287409	1.729043	-8.309985
H	-3.532061	2.301617	-8.863358
H	-4.696304	1.035593	-9.056017
C	-5.052463	4.050848	-8.088697
H	-4.881434	4.605411	-7.161743
H	-4.152321	4.178536	-8.702971

H	-5.865816	4.553805	-8.626713
C	-6.688169	2.241086	-8.313508
H	-7.209384	3.072143	-8.804958
H	-6.639799	1.429450	-9.050073
H	-7.328695	1.888980	-7.499814
N	-16.169970	8.345138	10.722038
N	-14.176148	10.457833	10.946160
C	-17.326720	8.331347	9.786718
H	-17.771962	7.335179	9.672108
H	-18.141761	8.997256	10.097121
H	-17.010830	8.665439	8.794598
C	-15.938429	6.994839	11.299928
H	-16.620730	6.235244	10.897997
H	-14.916895	6.666988	11.085136
H	-16.057861	6.967028	12.390165
C	-16.369662	9.360561	11.797092
H	-16.423721	8.879189	12.781960
H	-17.340775	9.860899	11.690662
C	-15.307697	10.481025	11.918764
H	-15.851608	11.432832	11.866528
H	-14.935471	10.442068	12.950575
C	-14.138480	11.711869	10.147045
H	-13.163592	12.213596	10.185284
H	-14.354756	11.495718	9.097158
H	-14.877469	12.454101	10.473966
C	-12.880741	10.231160	11.640293
H	-12.130145	10.996033	11.403993
H	-12.974775	10.221962	12.733148
H	-12.465994	9.260665	11.350681
N	-12.053844	-4.293126	6.168613
N	-9.266522	-4.857627	5.517041
N	-3.885951	1.008151	-5.847400
N	-5.354682	2.627495	-7.779895
N	-8.806708	16.249134	-0.492365
N	-9.605795	15.465488	-3.187381
N	-4.863045	2.815580	1.412717
N	-5.669436	1.717339	3.399546
N	-6.484461	1.049392	1.232827
N	-8.982398	-1.967865	4.405862
N	-2.440377	5.204033	5.134932
N	-17.219698	4.164019	-0.592998
N	-14.373960	2.428152	3.193994
N	-15.116890	4.578654	3.974806
N	-13.769894	3.180503	5.398556
N	-14.687278	7.261590	8.227545
N	-11.692043	-1.411629	5.058822
N	-9.069953	13.291102	0.399176
N	-9.717589	10.559533	4.594752
N	-8.330489	9.176387	5.996713
N	-7.424752	10.159577	3.994556
N	-3.492657	7.707339	5.961418
N	-16.262042	5.318616	-2.987466
N	-12.028340	8.060321	-3.056902
N	-9.730428	7.697573	-3.675266
N	-11.329199	5.909011	-3.878337
N	-7.005040	3.834403	-5.452906
N	-9.844517	12.527940	-2.213909
Pd	-1.642739	6.764850	6.290509
Pd	-9.334597	14.379056	-1.375141
Pd	-10.492584	-3.126032	5.294448
Pd	-5.446088	2.449313	-5.657865
Pd	-18.125548	4.521449	-2.456029
Pd	-14.443881	8.847041	9.577874

Table5_1b_TSii_DG_0wat

Property	Value
Charge	0
Electronic Energy, BS1 (a.u.)	-1058.932947
Thermal and entropic correction, BS1 (a.u.)	2.756600
Electronic Energy, BS2 (a.u.)	-1059.283070
Number of Imaginary Frequencies	0
Imaginary frequencies (cm-1)	None

Molecular Geometry in Cartesian Coordinates

C	-8.106781	2.438882	6.846702
C	-9.107547	2.692400	5.792934
C	-8.584349	3.210215	4.510463
C	-7.610169	4.213409	4.651335
C	-7.298613	4.546805	6.055642
C	-7.161014	3.401911	6.982206
H	-9.889127	2.164840	3.147343
H	-9.077517	3.403316	1.157669

H	-9.896125	1.945683	5.702689
C	-9.113519	2.921610	3.252251
C	-7.179702	4.932355	3.534866
H	-6.576178	5.350661	6.197429
H	-7.367041	5.188758	1.408251
C	-7.698488	4.628317	2.276112
C	-8.661473	3.624125	2.136022
H	-6.453439	5.733451	3.650507
C	-8.346844	1.338972	7.854095
H	-7.435318	0.784779	8.088761
H	-8.737299	1.738933	8.797490
H	-9.097865	0.632169	7.492674
C	-6.200875	3.513834	8.135783
H	-6.289911	4.496187	8.608913
H	-6.373513	2.757411	8.899446
H	-5.157763	3.399663	7.809254
C	-10.437883	3.911301	7.805576
C	-9.997849	4.258524	6.410579
C	-8.978647	5.314858	6.554945
C	-8.847683	5.575580	8.032368
H	-10.811308	4.368970	5.680581
H	-9.023469	6.216704	5.929222
C	-9.775612	4.514662	10.167563
C	-8.441187	4.182017	10.856728
C	-10.406501	5.785921	10.762320
H	-10.467313	3.677548	10.305516
C	-8.636327	4.052995	12.375277
H	-7.721607	4.977754	10.639529
H	-8.039168	3.251035	10.446267
C	-10.591764	5.653480	12.281109
H	-9.748908	6.631152	10.536989
H	-11.371542	5.960445	10.262825
C	-9.261228	5.322586	12.971868
H	-7.672580	3.842519	12.849104
H	-9.285737	3.193018	12.587203
H	-11.010158	6.580868	12.687204
H	-11.318345	4.857920	12.491603
H	-9.419718	5.200508	14.047846
H	-8.567359	6.164416	12.846347
N	-9.648077	4.643208	8.699768
O	-8.123613	6.394394	8.573103
O	-11.266799	3.082251	8.137391
C	-12.444459	-3.627846	7.438912
H	-12.370888	-4.287261	8.312771
H	-11.785098	-2.777240	7.631648
H	-13.474651	-3.250289	7.424344
C	-13.186314	-4.306793	5.204708
H	-14.079578	-3.798482	5.588755
H	-12.896592	-3.804123	4.277833
H	-13.505813	-5.318961	4.926601
C	-11.557270	-5.678638	6.422516
H	-12.157448	-6.417523	5.876167
H	-11.690933	-5.953145	7.476758
C	-10.076936	-5.975701	6.080276
H	-10.081817	-6.837568	5.400821
H	-9.620129	-6.364129	6.999628
C	-8.747603	-5.205590	4.166695
H	-9.189782	-4.547551	3.413631
H	-7.657746	-5.107076	4.088525
H	-8.984649	-6.233518	3.864750
C	-8.152901	-4.493138	6.433313
H	-8.298968	-3.477304	6.809146
H	-8.084769	-5.147411	7.311376
H	-7.169657	-4.526916	5.947399
N	0.232906	5.794506	6.602359
N	-0.835952	8.379033	7.437516
C	-0.043089	4.521947	7.320501
H	-1.105301	4.457201	7.570223
H	0.211138	3.633328	6.729138
H	0.508567	4.430423	8.264467
C	0.816245	5.530098	5.259943
H	0.181422	5.966890	4.483807
H	1.816117	5.962840	5.130265
H	0.913666	4.459761	5.039330
C	1.126144	6.678390	7.407498
H	1.461835	6.167720	8.319139
H	2.055697	6.893853	6.865126
C	0.555781	8.045156	7.859358
H	1.263961	8.806953	7.508935
H	0.651233	8.074605	8.952393
C	-0.853028	9.585772	6.568269
H	-1.526483	10.369377	6.937363
H	0.133189	10.054405	6.460532
H	-1.184055	9.316740	5.561318
C	-1.719815	8.563907	8.618647
H	-2.483307	7.782252	8.638480
H	-1.182543	8.504577	9.573492
H	-2.238514	9.530791	8.617595

N	-20.018297	3.724508	-1.877936
N	-19.017552	4.902950	-4.353377
C	-19.793482	2.310031	-1.477109
H	-19.990757	2.131196	-0.412746
H	-20.421046	1.599696	-2.029773
H	-18.754516	2.027387	-1.670034
C	-20.502471	4.533061	-0.727566
H	-20.576031	3.953570	0.201193
H	-19.818060	5.365617	-0.542173
H	-21.493744	4.972397	-0.895337
C	-20.966532	3.810119	-3.027007
H	-21.862923	4.380755	-2.752190
H	-21.352242	2.817078	-3.290871
C	-20.435628	4.437298	-4.340197
H	-20.611030	3.695952	-5.130227
H	-21.118783	5.259528	-4.588831
C	-18.940362	6.369555	-4.587079
H	-18.300699	6.633947	-5.438493
H	-19.917900	6.827494	-4.782788
H	-18.530855	6.866908	-3.703245
C	-18.223799	4.160834	-5.368662
H	-17.710678	4.822792	-6.077576
H	-17.460249	3.554145	-4.873912
H	-18.829305	3.473552	-5.972728
C	-7.417848	16.111194	0.025034
H	-7.359030	16.181611	1.118420
H	-7.006218	15.139914	-0.263593
H	-6.734152	16.873158	-0.369553
C	-9.763621	16.494866	0.619908
H	-9.282929	16.497044	1.606164
H	-10.288471	17.454267	0.531802
H	-10.532003	15.716919	0.630215
C	-8.890826	17.326584	-1.517703
H	-7.927295	17.840935	-1.625471
H	-9.581824	18.117116	-1.198178
C	-9.329482	16.914426	-2.944776
H	-10.207358	17.526383	-3.188578
H	-8.550370	17.281588	-3.625027
C	-11.013608	15.259124	-3.616884
H	-11.094374	14.708088	-4.562224
H	-11.555071	14.687089	-2.858069
H	-11.565544	16.196782	-3.758734
C	-8.656702	14.904376	-4.184082
H	-9.162237	14.424596	-5.031430
H	-7.983427	15.656952	-4.613174
H	-8.022679	14.149276	-3.710882
N	-5.546440	2.321190	-3.560678
C	-4.793262	3.119100	-2.776593
H	-4.164327	3.843298	-3.274754
C	-4.798251	3.043868	-1.370027
H	-4.155864	3.717006	-0.817097
C	-5.627555	2.099223	-0.713374
C	-6.420355	1.276777	-1.553748
H	-7.085615	0.525258	-1.148385
C	-6.348129	1.422774	-2.953135
H	-6.955898	0.797512	-3.591513
C	-5.657710	1.980104	0.709093
C	-4.907299	2.627127	2.750679
C	-4.090341	3.486029	3.546888
C	-3.261309	4.479298	2.967803
H	-3.207954	4.621107	1.895965
C	-2.465720	5.300003	3.789813
H	-1.827552	6.048624	3.341596
C	-3.228954	4.266411	5.700749
H	-3.195857	4.191117	6.777877
C	-4.055316	3.396525	4.960582
H	-4.644153	2.659556	5.490746
C	-6.437937	0.961973	2.581598
C	-7.290574	-0.006702	3.193156
C	-7.341490	-0.188590	4.598075
H	-6.726947	0.405490	5.264181
C	-8.134494	-0.849480	2.426992
H	-8.161784	-0.795877	1.346200
C	-8.949452	-1.801222	3.068407
H	-9.582758	-2.449657	2.479378
C	-8.194357	-1.165100	5.151804
H	-8.225680	-1.306216	6.222736
C	-16.512203	3.037841	-0.372338
H	-16.528614	2.288472	-1.150684
C	-15.775645	2.809789	0.806125
H	-15.241314	1.873831	0.906062
C	-17.202298	5.113606	0.364485
H	-17.765412	6.015736	0.172516
C	-16.496874	4.972683	1.575394
H	-16.541749	5.780262	2.294774
C	-15.757436	3.789916	1.830804
C	-15.046637	3.592040	3.052910
C	-14.477986	4.301673	5.132478

C	-13.773199	2.275233	4.394031
C	-13.068120	1.052927	4.613418
C	-12.431680	0.759829	5.846093
H	-12.465803	1.451672	6.677428
C	-12.977629	0.051751	3.613729
H	-13.437992	0.175765	2.641565
C	-12.290319	-1.147844	3.879555
H	-12.231234	-1.913356	3.118663
C	-11.760562	-0.467192	6.020035
H	-11.280724	-0.687376	6.963046
C	-14.544804	5.289150	6.161257
C	-15.207490	6.527891	5.972209
H	-15.691004	6.775360	5.035632
C	-15.249224	7.470031	7.018178
H	-15.758063	8.412213	6.870659
C	-14.050411	6.087924	8.421272
H	-13.608437	5.924002	9.393065
C	-13.959868	5.086033	7.435842
H	-13.462759	4.159060	7.686040
C	-7.900558	12.673988	0.664460
H	-7.079401	12.868011	-0.010493
C	-10.107487	13.046437	1.225873
H	-11.043370	13.535974	0.997497
C	-10.015048	12.203881	2.350824
H	-10.893588	12.070259	2.968917
C	-8.788487	11.560809	2.654546
C	-7.714539	11.814399	1.764113
H	-6.738930	11.368693	1.909149
C	-8.639028	10.717342	3.796326
C	-9.491968	9.799377	5.689118
C	-7.329622	9.417588	5.119063
N	-12.763050	9.339579	8.420515
C	-10.582822	9.636115	6.595714
C	-11.856363	10.205904	6.343961
H	-12.054076	10.784197	5.450329
C	-12.901119	10.031144	7.270977
H	-13.869578	10.470066	7.076806
C	-10.455981	8.907089	7.804832
H	-9.515226	8.460379	8.096573
C	-11.558934	8.786140	8.673455
H	-11.455569	8.241212	9.599610
C	-6.056998	8.831636	5.394952
C	-5.815559	8.063924	6.562074
H	-6.591307	7.898346	7.297941
C	-4.535014	7.524271	6.799111
H	-4.353885	6.943520	7.692687
C	-3.707358	8.428396	4.842979
H	-2.865600	8.564697	4.178717
C	-4.952265	9.001007	4.522283
H	-5.030994	9.576460	3.608514
C	-15.269741	4.518514	-3.428435
H	-15.533165	3.500377	-3.676675
C	-15.941822	6.588980	-2.666982
H	-16.741869	7.220975	-2.309249
C	-14.638633	7.109516	-2.781933
H	-14.471034	8.143401	-2.508699
C	-13.937824	4.950832	-3.578685
H	-13.204497	4.244629	-3.946431
C	-13.586650	6.285897	-3.256131
C	-12.253401	6.773681	-3.402918
C	-10.748314	8.461388	-3.217989
C	-10.089526	6.434121	-3.995203
C	-10.445283	9.813665	-2.876424
C	-11.436252	10.706003	-2.394805
H	-12.464607	10.394791	-2.263011
C	-11.093502	12.035702	-2.083153
H	-11.852268	12.714153	-1.719973
C	-8.888832	11.689139	-2.663996
H	-7.890712	12.090672	-2.763337
C	-9.137100	10.344893	-3.002750
H	-8.312504	9.742225	-3.361365
C	-9.064107	5.571749	-4.487553
C	-7.722093	6.004855	-4.631370
H	-7.422362	7.014303	-4.380406
C	-6.742284	5.112250	-5.107964
H	-5.720234	5.447097	-5.213474
C	-9.325926	4.227875	-4.854368
H	-10.319406	3.803782	-4.784161
C	-8.279820	3.410311	-5.323237
H	-8.485754	2.386621	-5.601171
C	-4.402967	-0.273306	-5.296267
H	-5.456844	-0.164719	-5.025493
H	-4.345588	-1.104295	-6.010332
H	-3.864204	-0.601822	-4.398597
C	-2.730414	1.499309	-5.050164
H	-2.937556	2.503340	-4.670210
H	-2.503530	0.860986	-4.187188
H	-1.803401	1.569058	-5.632748

C	-3.516664	0.859872	-7.284649
H	-2.451199	1.073274	-7.438884
H	-3.631199	-0.181057	-7.613125
C	-4.287559	1.728350	-8.309243
H	-3.531978	2.300529	-8.862706
H	-4.696546	1.034762	-9.055095
C	-5.051862	4.050490	-8.088519
H	-4.880852	4.605104	-7.161589
H	-4.151553	4.177794	-8.702628
H	-5.864940	4.553663	-8.626749
C	-6.688122	2.241281	-8.313547
H	-7.208861	3.072443	-8.805325
H	-6.639839	1.429488	-9.049946
H	-7.329043	1.889584	-7.499993
N	-16.170345	8.344623	10.720523
N	-14.177045	10.457738	10.945754
C	-17.326832	8.331791	9.784871
H	-17.772310	7.335824	9.669436
H	-18.141790	8.997701	10.095492
H	-17.010532	8.666484	8.793081
C	-15.939273	6.993809	11.297403
H	-16.621369	6.234613	10.894378
H	-14.917633	6.666052	11.082981
H	-16.059435	6.965063	12.387540
C	-16.370093	9.359233	11.796332
H	-16.423816	8.877165	12.780879
H	-17.341370	9.859367	11.690432
C	-15.308464	10.479963	11.918530
H	-15.852700	11.431617	11.866895
H	-14.936097	10.440544	12.950270
C	-14.140346	11.711928	10.146840
H	-13.165849	12.214413	10.185129
H	-14.356481	11.495748	9.096927
H	-14.879899	12.453536	10.473898
C	-12.881372	10.231847	11.639656
H	-12.131216	10.997066	11.403085
H	-12.975168	10.222783	12.732533
H	-12.466172	9.261530	11.350092
N	-12.053352	-4.294582	6.168739
N	-9.265600	-4.856896	5.517066
N	-3.886594	1.008182	-5.846358
N	-5.354648	2.627286	-7.779600
N	-8.806048	16.247571	-0.490606
N	-9.605270	15.467618	-3.186628
N	-4.863881	2.816037	1.412958
N	-5.669734	1.717370	3.399760
N	-6.485624	1.050160	1.233147
N	-8.983759	-1.966732	4.406298
N	-2.440069	5.203618	5.135026
N	-17.220328	4.163830	-0.592982
N	-14.373506	2.428029	3.193334
N	-15.115703	4.578883	3.973879
N	-13.768307	3.180925	5.397411
N	-14.686279	7.261766	8.226555
N	-11.693688	-1.412677	5.059573
N	-9.070787	13.288466	0.397287
N	-9.717660	10.558757	4.594102
N	-8.330532	9.175723	5.996120
N	-7.424989	10.158313	3.993575
N	-3.492628	7.706988	5.961168
N	-16.262473	5.318687	-2.987214
N	-12.028844	8.060547	-3.057092
N	-9.730960	7.697743	-3.675556
N	-11.329666	5.909075	-3.878079
N	-7.005673	3.834568	-5.453300
N	-9.845309	12.528988	-2.216956
Pd	-1.642661	6.764639	6.290517
Pd	-9.334783	14.378816	-1.375715
Pd	-10.492971	-3.126184	5.294813
Pd	-5.446734	2.449458	-5.657570
Pd	-18.126063	4.521563	-2.456003
Pd	-14.443838	8.847034	9.577215

Table5_1b_TSiii_TSi-ii_0wat

Property	Value
Charge	0
Electronic Energy, BS1 (a.u.)	-1058.887452
Thermal and entropic correction, BS1 (a.u.)	2.755695
Electronic Energy, BS2 (a.u.)	-1059.239787
Number of Imaginary Frequencies	0
Imaginary frequencies (cm-1)	None

Molecular Geometry in Cartesian Coordinates

C	-9.238989	8.901994	4.698761
H	-10.049265	8.668423	5.400655
H	-8.319175	8.505986	5.138333
H	-9.152235	9.995634	4.681511
C	-9.520430	9.379597	2.312060
H	-9.366869	10.385797	2.721680
H	-8.753044	9.214921	1.550910
H	-10.483466	9.405874	1.787008
C	-10.670837	7.461533	3.319658
H	-11.383214	7.820545	2.565811
H	-11.221824	7.538033	4.265855
C	-10.473622	5.949497	3.052386
H	-11.097852	5.702461	2.184141
H	-10.956363	5.424605	3.886761
C	-8.919060	4.811238	1.534635
H	-8.250278	5.400927	0.901514
H	-8.500407	3.799431	1.603866
H	-9.865071	4.714283	0.987238
C	-8.676800	4.543855	3.958268
H	-7.874636	4.994025	4.549848
H	-9.495265	4.324127	4.655114
H	-8.314878	3.574696	3.592808
N	2.654931	-1.137009	7.494753
N	4.590105	0.626902	8.778223
C	1.233118	-1.149220	7.928719
H	0.873638	-0.123996	8.052248
H	0.570337	-1.647591	7.210125
H	1.080158	-1.653962	8.890814
C	2.820124	-1.842416	6.195462
H	3.252958	-1.166188	5.452863
H	3.486834	-2.711679	6.257680
H	1.871401	-2.212960	5.787404
C	3.537488	-1.733975	8.539537
H	2.938738	-2.184828	9.341384
H	4.116119	-2.573673	8.133753
C	4.551368	-0.793042	9.235131
H	5.536199	-1.266337	9.131488
H	4.338900	-0.851083	10.310399
C	5.938540	0.977583	8.257725
H	6.360420	1.871698	8.733365
H	6.676810	0.178097	8.398290
H	5.887894	1.169707	7.182749
C	4.184408	1.549708	9.870800
H	3.251669	2.054342	9.604071
H	4.002879	1.036585	10.823499
H	4.933188	2.325056	10.075993
N	-1.680674	17.629793	-3.475229
N	0.272179	16.895474	-5.516145
C	-3.047149	17.047817	-3.400524
H	-3.530730	17.215031	-2.429894
H	-3.730217	17.452412	-4.157824
H	-3.002898	15.967756	-3.564685
C	-1.337045	18.343126	-2.216686
H	-2.129829	18.281494	-1.460763
H	-0.431449	17.913067	-1.779420
H	-1.138428	19.411750	-2.366522
C	-1.550509	18.534782	-4.654542
H	-1.286555	19.552725	-4.340128
H	-2.515454	18.654131	-5.163678
C	-0.529010	18.133386	-5.747540
H	-1.090654	18.066127	-6.688185
H	0.127275	19.001543	-5.889681
C	1.721834	17.211390	-5.415606
H	2.335977	16.628736	-6.113643
H	1.948846	18.266384	-5.614146
H	2.080577	16.998127	-4.404785
C	0.020049	15.891081	-6.583547
H	0.939207	15.553401	-7.078347
H	-0.466274	15.008370	-6.158520
H	-0.638248	16.262242	-7.378913
C	12.324292	8.495220	2.709902
H	12.123234	8.504561	3.788425
H	11.573249	7.853288	2.241311
H	13.291343	7.992996	2.582165
C	12.026467	10.890097	3.134010
H	11.881291	10.469268	4.136888
H	12.838422	11.622852	3.222488
H	11.123767	11.451943	2.879802
C	13.545977	10.139885	1.358781
H	14.267298	9.321621	1.480228
H	14.058401	11.013233	1.782200
C	13.429982	10.397039	-0.163985
H	13.896998	11.372707	-0.349560
H	14.105105	9.681537	-0.650641
C	11.686778	11.649334	-1.355917
H	11.389471	11.576043	-2.409517

H	10.842577	12.074722	-0.805938
H	12.494823	12.390358	-1.315397
C	11.983548	9.255279	-1.785666
H	11.633770	9.612686	-2.762249
H	12.944548	8.757869	-1.967024
H	11.287513	8.480784	-1.451119
N	0.537762	2.774181	-3.865415
C	1.292047	2.358486	-2.826959
H	2.250014	1.918292	-3.065367
C	0.884100	2.461402	-1.482647
H	1.547773	2.088414	-0.713011
C	-0.377894	3.024985	-1.167159
C	-1.157446	3.459540	-2.268493
H	-2.141184	3.890630	-2.132688
C	-0.662771	3.319236	-3.579638
H	-1.262760	3.647801	-4.416422
C	-0.841890	3.136104	0.178094
C	-0.555901	2.730291	2.390649
C	0.240241	2.203400	3.452643
C	1.544822	1.694972	3.231005
H	1.982473	1.668544	2.241717
C	2.301917	1.200458	4.310777
H	3.293639	0.807168	4.135578
C	0.610649	1.649225	5.808193
H	0.253353	1.621862	6.827095
C	-0.223455	2.154597	4.790634
H	-1.213484	2.501307	5.056649
C	-2.433765	3.775297	1.661784
C	-3.635038	4.496416	1.941735
C	-4.128968	4.656196	3.261452
H	-3.622875	4.215828	4.110792
C	-4.395982	5.111829	0.914697
H	-4.106938	5.042044	-0.126068
C	-5.567305	5.825467	1.235469
H	-6.149347	6.288888	0.451170
C	-5.310478	5.391414	3.487619
H	-5.687245	5.511830	4.493028
C	-1.862726	14.174774	-1.818170
H	-2.367387	13.956584	-2.748670
C	-2.253124	13.513661	-0.637643
H	-3.065835	12.800448	-0.693953
C	-0.229248	15.367659	-0.705256
H	0.560863	16.103376	-0.753150
C	-0.548262	14.753005	0.522292
H	0.008436	15.042905	1.404256
C	-1.591924	13.794325	0.585283
C	-1.966934	13.158556	1.808132
C	-1.707551	12.889081	4.044428
C	-3.331085	11.745851	2.946756
C	-4.359162	10.754072	2.948913
C	-4.759182	10.089132	4.135752
H	-4.307936	10.321183	5.091276
C	-5.027348	10.362411	1.761527
H	-4.796333	10.816089	0.806538
C	-6.025753	9.369916	1.810157
H	-6.537807	9.077715	0.904394
C	-5.767341	9.105902	4.087510
H	-6.071808	8.602577	4.993604
C	-1.011605	13.181505	5.256481
C	0.092383	14.070607	5.297915
H	0.452979	14.567735	4.406605
C	0.726970	14.347745	6.525632
H	1.557971	15.038029	6.559436
C	-0.694982	12.939119	7.671557
H	-0.992864	12.503221	8.614314
C	-1.393795	12.606788	6.495140
H	-2.229439	11.922764	6.570369
C	8.845030	8.157816	2.473576
H	9.361174	7.383552	1.924101
C	8.567964	10.408990	2.897625
H	8.865076	11.426476	2.686676
C	7.553534	10.155639	3.841860
H	7.096787	10.998030	4.344643
C	7.158631	8.821994	4.119622
C	7.843410	7.809464	3.401326
H	7.618672	6.760851	3.547144
C	6.132469	8.518225	5.065247
C	4.593168	9.212972	6.579164
C	4.844767	7.000607	6.154774
N	2.641360	12.429177	8.633436
C	3.928872	10.281651	7.254414
C	4.282344	11.637139	7.034186
H	5.070737	11.914466	6.346514
C	3.620670	12.661751	7.736614
H	3.901145	13.692698	7.573156
C	2.888398	10.055154	8.190535
H	2.557089	9.054633	8.435275
C	2.281785	11.146142	8.846391

H	1.495636	10.972145	9.567501
C	4.470381	5.640722	6.385425
C	3.476354	5.278752	7.330696
H	2.968129	6.024264	7.928577
C	3.145077	3.921035	7.521918
H	2.389005	3.646704	8.243928
C	4.675875	3.242251	5.934086
H	5.139655	2.427984	5.394991
C	5.073122	4.568860	5.678411
H	5.848699	4.738361	4.942273
C	0.561423	13.182473	-4.275271
H	-0.409257	13.164083	-4.750521
C	2.223901	14.415652	-3.264171
H	2.579104	15.380533	-2.931727
C	3.030908	13.274569	-3.085223
H	4.001109	13.397377	-2.621083
C	1.294134	11.987654	-4.135727
H	0.860714	11.072615	-4.518238
C	2.572012	12.006554	-3.522520
C	3.348789	10.819677	-3.358308
C	5.220974	9.780374	-2.600862
C	3.580354	8.574753	-3.607844
C	6.482157	9.828632	-1.933134
C	7.000286	11.029091	-1.385035
H	6.464312	11.966032	-1.465237
C	8.242078	11.019365	-0.717889
H	8.633693	11.935535	-0.299256
C	8.517335	8.760700	-1.089895
H	9.127348	7.876792	-0.968512
C	7.287881	8.673259	-1.770904
H	6.986854	7.710259	-2.162751
C	3.074187	7.321323	-4.068146
C	3.809400	6.117056	-3.923238
H	4.783823	6.099470	-3.451877
C	3.284117	4.907576	-4.418243
H	3.848584	3.991790	-4.314253
C	1.813114	7.208225	-4.707161
H	1.179757	8.071139	-4.869197
C	1.368937	5.953934	-5.170677
H	0.411934	5.869058	-5.665626
C	-0.830032	1.154267	-6.404897
H	-1.158862	2.149750	-6.094115
H	-1.318536	0.947547	-7.365362
H	-1.242885	0.432410	-5.689091
C	1.201144	0.061279	-5.580422
H	1.887991	0.509386	-4.856869
H	0.422248	-0.462208	-5.012081
H	1.765623	-0.713907	-6.113443
C	1.109240	0.897542	-7.886692
H	1.693830	-0.027417	-7.972696
H	0.253101	0.735296	-8.554065
C	1.961682	2.015534	-8.536173
H	2.882112	1.534639	-8.891345
H	1.440531	2.307214	-9.456992
C	3.741348	3.343833	-7.492749
H	3.984981	3.195799	-6.437378
H	4.325465	2.605440	-8.056176
H	4.126916	4.328383	-7.785518
C	1.710329	4.445818	-8.306538
H	2.461598	5.231552	-8.455194
H	1.248627	4.272919	-9.286720
H	0.929557	4.855924	-7.659969
N	0.368061	15.638113	10.193839
N	2.713169	14.210683	11.176908
C	0.392808	16.716524	9.169685
H	-0.596706	16.923868	8.743497
H	0.765563	17.672542	9.558247
H	1.052530	16.434261	8.344246
C	-1.016884	15.140014	10.406404
H	-1.751302	15.623831	9.750316
H	-1.060598	14.064900	10.212458
H	-1.376228	15.288502	11.432345
C	0.967561	16.109199	11.476680
H	0.224959	16.089330	12.284659
H	1.260770	17.164728	11.409195
C	2.204220	15.342405	12.005677
H	2.988243	16.093348	12.166734
H	1.948449	15.002055	13.017296
C	4.109063	14.465918	10.731665
H	4.795416	13.648945	10.987322
H	4.138140	14.591374	9.645982
H	4.541002	15.376622	11.165162
C	2.618453	12.924225	11.916926
H	3.578850	12.398009	11.984168
H	2.261459	13.043883	12.947551
H	1.912086	12.256611	11.415169
N	-9.447597	8.315397	3.348538
N	-9.075051	5.465207	2.861495

N	0.654631	1.111163	-6.481205
N	2.274587	3.211080	-7.699410
N	12.284291	9.851569	2.101430
N	12.066463	10.336960	-0.768300
N	-0.020918	2.684022	1.150275
N	-1.756181	3.264508	2.715674
N	-2.046833	3.716729	0.367684
N	-6.024536	5.963432	2.496303
N	1.854435	1.176212	5.583198
N	-0.874912	15.091649	-1.856599
N	-2.995055	12.283965	1.752396
N	-1.243657	13.462648	2.910301
N	-2.755495	12.042164	4.134479
N	0.342554	13.799647	7.696981
N	-6.397587	8.750683	2.949298
N	9.210050	9.432414	2.224835
N	5.567640	9.561541	5.710060
N	4.183879	7.951673	6.855324
N	5.812271	7.215922	5.235373
N	3.733238	2.915471	6.841099
N	1.009571	14.382182	-3.851598
N	4.541808	10.942739	-2.731935
N	4.786385	8.563734	-2.999590
N	2.795362	9.660594	-3.783039
N	2.090267	4.822129	-5.039145
N	8.995894	9.909591	-0.570788
Pd	3.201369	0.901480	7.175606
Pd	10.634291	9.887110	0.744657
Pd	-7.732969	7.121492	2.908886
Pd	1.378656	2.987333	-5.775342
Pd	-0.309109	16.004465	-3.669001
Pd	1.515303	14.014204	9.423011
C	0.437849	5.196765	5.244018
C	1.388028	5.834120	4.343091
C	0.938216	5.990761	2.957410
C	-0.410607	6.354531	2.802340
C	-1.159924	6.537997	4.068987
C	-0.866005	5.520165	5.091202
H	2.804700	5.605476	1.957796
H	0.787512	4.591749	6.073225
H	2.449882	5.663839	4.491151
C	1.760937	5.875288	1.831256
C	-0.903074	6.637932	1.525838
H	-2.202162	6.822712	3.942706
H	-1.634708	5.189859	5.780669
C	-0.091466	6.509258	0.396397
C	1.259109	6.110227	0.552091
H	-1.927039	6.983438	1.412308
C	2.166471	6.018067	-0.649490
H	1.761637	5.348158	-1.417049
H	2.282486	7.002129	-1.121230
H	3.158557	5.657281	-0.374856
C	-0.625636	6.859992	-0.968348
H	-0.146490	7.768723	-1.352480
H	-0.427157	6.073687	-1.703515
H	-1.701459	7.038537	-0.949636
C	-0.450385	9.063206	3.717970
C	-0.376113	8.028414	4.811969
C	1.051714	7.806049	5.049073
C	1.800664	8.696031	4.121102
H	-1.022855	8.227874	5.660722
H	1.453143	7.688560	6.047436
C	1.159782	10.302501	2.246358
C	1.994468	11.505177	2.712161
C	1.836088	9.533281	1.104241
H	0.181666	10.658287	1.904516
C	2.317538	12.420321	1.523930
H	2.922819	11.139552	3.161766
H	1.446142	12.051705	3.488266
C	2.204745	10.458789	-0.059231
H	2.736768	9.054976	1.498966
H	1.166146	8.738970	0.770639
C	3.047617	11.648806	0.416143
H	2.914818	13.272991	1.856478
H	1.380804	12.825729	1.116050
H	2.741191	9.870795	-0.812252
H	1.289522	10.830610	-0.540996
H	3.283126	12.318081	-0.417188
H	4.006674	11.283055	0.805314
N	0.851914	9.397666	3.360150
O	3.008981	8.806784	3.995696
O	-1.454393	9.490343	3.180398

Table5_1b_TSiii_DG_0wat

Property	Value

Charge	0
Electronic Energy, BS1 (a.u.)	-1058.936493
Thermal and entropic correction, BS1 (a.u.)	2.759347
Electronic Energy, BS2 (a.u.)	-1059.285580
Number of Imaginary Frequencies	0
Imaginary frequencies (cm-1)	None

Molecular Geometry in Cartesian Coordinates

C	-9.240282	8.904726	4.711685
H	-10.050934	8.675659	5.414610
H	-8.322274	8.502836	5.149700
H	-9.146859	9.997794	4.694872
C	-9.521973	9.385281	2.325512
H	-9.360727	10.390254	2.735175
H	-8.757152	9.215506	1.562898
H	-10.485741	9.418279	1.802187
C	-10.683191	7.474191	3.333912
H	-11.394770	7.838827	2.582021
H	-11.231766	7.552860	4.281316
C	-10.496258	5.961293	3.064096
H	-11.124242	5.719391	2.197115
H	-10.980289	5.438346	3.898952
C	-8.952122	4.817022	1.539935
H	-8.277602	5.402002	0.908496
H	-8.543722	3.800776	1.605784
H	-9.899147	4.731227	0.992457
C	-8.706987	4.541200	3.962363
H	-7.900168	4.984132	4.553114
H	-9.525251	4.325356	4.660645
H	-8.352560	3.570560	3.593494
N	2.624704	-1.135317	7.513796
N	4.549809	0.635702	8.801926
C	1.202573	-1.147881	7.946589
H	0.843147	-0.122822	8.071280
H	0.540388	-1.645495	7.226932
H	1.048940	-1.653452	8.908137
C	2.791965	-1.843274	6.216118
H	3.223766	-1.167692	5.472099
H	3.460388	-2.711052	6.280758
H	1.844178	-2.216789	5.808493
C	3.506615	-1.729095	8.560859
H	2.907040	-2.185647	9.358867
H	4.092632	-2.564029	8.155859
C	4.510109	-0.782584	9.263961
H	5.497231	-1.253526	9.172104
H	4.286816	-0.837235	10.337211
C	5.901286	0.986059	8.289084
H	6.317632	1.884254	8.761918
H	6.640275	0.189186	8.440252
H	5.858210	1.170773	7.212534
C	4.135593	1.562463	9.887957
H	3.205637	2.067180	9.611515
H	3.945632	1.052610	10.840766
H	4.883164	2.338009	10.096788
N	-1.680908	17.605795	-3.443660
N	0.268432	16.881131	-5.491368
C	-3.046041	17.021198	-3.365252
H	-3.527224	17.187608	-2.393298
H	-3.731967	17.424592	-4.120605
H	-3.000207	15.941113	-3.529415
C	-1.334071	18.317210	-2.184937
H	-2.122898	18.250442	-1.425358
H	-0.424580	17.889830	-1.753357
H	-1.140396	19.386996	-2.332883
C	-1.556354	18.513625	-4.621421
H	-1.297230	19.532250	-4.305180
H	-2.522235	18.629211	-5.129636
C	-0.533098	18.120114	-5.715578
H	-1.093880	18.058422	-6.657128
H	0.122766	18.989553	-5.851675
C	1.718455	17.196056	-5.392841
H	2.330573	16.617299	-6.095896
H	1.945236	18.252118	-5.585883
H	2.079913	16.976498	-4.384265
C	0.013539	15.881602	-6.562718
H	0.931588	15.544606	-7.060044
H	-0.473769	14.997939	-6.141044
H	-0.645108	16.257091	-7.355759
C	12.354727	8.478376	2.689937
H	12.171324	8.485596	3.771625
H	11.599625	7.832614	2.233321
H	13.322180	7.981286	2.545930
C	12.052158	10.871346	3.121552
H	11.928739	10.448857	4.126622
H	12.861023	11.609053	3.195369

H	11.140874	11.427351	2.885318
C	13.544767	10.130221	1.319447
H	14.272331	9.315754	1.428564
H	14.059676	11.006296	1.734169
C	13.401517	10.386589	-0.201150
H	13.866085	11.361827	-0.395035
H	14.067001	9.670573	-0.700137
C	11.634558	11.644897	-1.350539
H	11.322289	11.580323	-2.400340
H	10.797058	12.062219	-0.784517
H	12.440541	12.388390	-1.314907
C	11.927311	9.255180	-1.805221
H	11.558846	9.620213	-2.772080
H	12.885821	8.761538	-2.008770
H	11.238861	8.476461	-1.465042
N	0.558494	2.813539	-3.855721
C	1.315391	2.409067	-2.814741
H	2.276938	1.973996	-3.048511
C	0.905515	2.518102	-1.471556
H	1.571039	2.154524	-0.699000
C	-0.361445	3.073143	-1.160542
C	-1.142182	3.498365	-2.264891
H	-2.127950	3.925594	-2.131917
C	-0.645294	3.354117	-3.575023
H	-1.245593	3.675698	-4.414295
C	-0.829774	3.181995	0.183530
C	-0.554168	2.758720	2.394159
C	0.234147	2.218883	3.455765
C	1.543815	1.720909	3.240163
H	1.992357	1.711891	2.255356
C	2.292289	1.214322	4.320556
H	3.287577	0.828190	4.149876
C	0.583308	1.630503	5.806586
H	0.214715	1.585605	6.820919
C	-0.243683	2.144802	4.787796
H	-1.240232	2.475781	5.048405
C	-2.435848	3.795795	1.662736
C	-3.644166	4.504769	1.943761
C	-4.142545	4.654163	3.263156
H	-3.636528	4.213535	4.112028
C	-4.410316	5.115786	0.918281
H	-4.119672	5.051563	-0.122392
C	-5.588725	5.817772	1.240024
H	-6.173256	6.278925	0.456201
C	-5.330708	5.378045	3.490594
H	-5.710140	5.490418	4.495942
C	-1.850176	14.141904	-1.796659
H	-2.348635	13.917158	-2.728978
C	-2.243699	13.484524	-0.615158
H	-3.052253	12.766642	-0.672853
C	-0.229183	15.348476	-0.680306
H	0.557257	16.088224	-0.726774
C	-0.552838	14.738909	0.548735
H	-0.006853	15.039698	1.433912
C	-1.590908	13.774393	0.610043
C	-1.969213	13.142116	1.833459
C	-1.709572	12.870067	4.069440
C	-3.332240	11.726991	2.970010
C	-4.362210	10.737463	2.969969
C	-4.761047	10.067508	4.154215
H	-4.308283	10.294575	5.110156
C	-5.033203	10.352792	1.781972
H	-4.801153	10.808061	0.827791
C	-6.031620	9.360363	1.826716
H	-6.543552	9.071612	0.919778
C	-5.769515	9.084707	4.102472
H	-6.072217	8.576334	5.006300
C	-1.016188	13.165460	5.282205
C	0.082089	14.061501	5.325016
H	0.440448	14.560932	4.434018
C	0.712142	14.343706	6.553938
H	1.537574	15.040599	6.589972
C	-0.701395	12.925237	7.697891
H	-0.997449	12.486435	8.639998
C	-1.396469	12.588542	6.520480
H	-2.227952	11.899360	6.595094
C	8.862669	8.139843	2.515625
H	9.391692	7.362318	1.983216
C	8.570269	10.392990	2.915520
H	8.868936	11.409714	2.702892
C	7.537880	10.144668	3.841457
H	7.067274	10.990059	4.326229
C	7.143408	8.812270	4.124457
C	7.845499	7.796010	3.427957
H	7.622654	6.747882	3.579808
C	6.102660	8.512990	5.055589
C	4.548522	9.212614	6.552518
C	4.809923	6.998039	6.143868

N	2.632604	12.422347	8.651426
C	3.892402	10.280290	7.237331
C	4.250244	11.635778	7.024534
H	5.032883	11.914419	6.330808
C	3.603123	12.657238	7.745417
H	3.889177	13.687927	7.590313
C	2.860506	10.051402	8.182385
H	2.527516	9.050648	8.424347
C	2.267549	11.139457	8.855109
H	1.489237	10.962689	9.584010
C	4.438951	5.638784	6.383379
C	3.434281	5.281082	7.319126
H	2.915100	6.029224	7.904196
C	3.104337	3.923890	7.516471
H	2.339554	3.652724	8.230409
C	4.656318	3.238009	5.952596
H	5.128265	2.421622	5.424045
C	5.053427	4.563625	5.692236
H	5.836645	4.729668	4.963472
C	0.561959	13.165548	-4.275161
H	-0.406638	13.152006	-4.754739
C	2.221964	14.388469	-3.247617
H	2.577731	15.350302	-2.906997
C	3.025400	13.244349	-3.072459
H	3.993864	13.362055	-2.603003
C	1.291651	11.968150	-4.140813
H	0.858139	11.056067	-4.530502
C	2.565432	11.980106	-3.519349
C	3.337777	10.790528	-3.354482
C	5.199981	9.746349	-2.580767
C	3.579089	8.549684	-3.629072
C	6.456290	9.793374	-1.904145
C	6.969950	10.993567	-1.351225
H	6.433734	11.930064	-1.433867
C	8.208798	10.985042	-0.678997
H	8.598002	11.900957	-0.257703
C	8.488067	8.726246	-1.051521
H	9.098707	7.843218	-0.926821
C	7.261712	8.637980	-1.738330
H	6.963396	7.675156	-2.132489
C	3.084640	7.300453	-4.113021
C	3.824735	6.098256	-3.976070
H	4.795658	6.079943	-3.497729
C	3.308327	4.891584	-4.486393
H	3.876460	3.977466	-4.387582
C	1.829983	7.188799	-4.764676
H	1.194759	8.050848	-4.923594
C	1.394398	5.937069	-5.243360
H	0.442110	5.853440	-5.747876
C	-0.846909	1.114693	-6.320394
H	-1.168713	2.120661	-6.037698
H	-1.352117	0.877141	-7.264990
H	-1.248797	0.417787	-5.574236
C	1.195828	0.042130	-5.497641
H	1.896427	0.511095	-4.800812
H	0.425951	-0.461229	-4.899547
H	1.749323	-0.750518	-6.016365
C	1.065868	0.808511	-7.826424
H	1.643648	-0.122110	-7.895177
H	0.197876	0.632367	-8.474669
C	1.913667	1.902111	-8.521847
H	2.823858	1.404666	-8.880653
H	1.377671	2.173224	-9.440426
C	3.722091	3.237523	-7.538188
H	3.976754	3.115592	-6.482097
H	4.290500	2.477001	-8.088040
H	4.115629	4.209101	-7.862199
C	1.694748	4.341094	-8.358019
H	2.453854	5.112601	-8.537987
H	1.218908	4.146670	-9.327316
H	0.927864	4.779852	-7.713650
N	0.360756	15.625905	10.225146
N	2.710269	14.198655	11.198528
C	0.384376	16.708176	9.205020
H	-0.605737	16.917683	8.781245
H	0.758258	17.662528	9.596593
H	1.042622	16.428793	8.377446
C	-1.023903	15.126620	10.436891
H	-1.758865	15.612342	9.782857
H	-1.067508	14.052167	10.239230
H	-1.382675	15.271544	11.463554
C	0.961095	16.092264	11.509340
H	0.220391	16.065169	12.318847
H	1.249991	17.149296	11.446885
C	2.202145	15.327777	12.031369
H	2.984977	16.080424	12.190230
H	1.951826	14.985084	13.043568
C	4.104554	14.456725	10.749923

H	4.792731	13.640197	11.002096
H	4.130417	14.584132	9.664370
H	4.536482	15.367136	11.184065
C	2.619426	12.910377	11.935911
H	3.581796	12.387737	12.003681
H	2.260485	13.026683	12.966238
H	1.916251	12.241074	11.431982
N	-9.454476	8.320199	3.361470
N	-9.101301	5.468293	2.868958
N	0.636168	1.065457	-6.420365
N	2.251386	3.116792	-7.723309
N	12.297510	9.835179	2.083501
N	12.027134	10.328569	-0.780553
N	-0.009725	2.731108	1.157496
N	-1.760801	3.280830	2.715882
N	-2.042335	3.748818	0.370073
N	-6.048761	5.947426	2.500762
N	1.832748	1.169178	5.588218
N	-0.866634	15.063238	-1.834136
N	-2.997318	12.268125	1.776888
N	-1.246877	13.446622	2.936216
N	-2.753051	12.017269	4.157676
N	0.330115	13.793054	7.724818
N	-6.401603	8.735473	2.963288
N	9.228565	9.413438	2.262916
N	5.524097	9.559455	5.684066
N	4.132930	7.952595	6.823664
N	5.786581	7.210579	5.233700
N	3.703328	2.915110	6.850084
N	1.010191	14.361586	-3.840644
N	4.517598	10.907287	-2.703387
N	4.775666	8.533956	-3.002075
N	2.791571	9.635059	-3.798175
N	2.119212	4.806831	-5.116254
N	8.962842	9.875852	-0.530580
Pd	3.170620	0.902282	7.189899
Pd	10.624149	9.864068	0.755925
Pd	-7.748302	7.115510	2.918953
Pd	1.377313	2.959513	-5.783348
Pd	-0.307969	15.982658	-3.646133
Pd	1.507572	14.004962	9.447498
C	0.552010	5.255057	5.082109
C	1.473712	6.182073	4.314617
C	1.085352	6.165080	2.848706
C	-0.274455	6.421713	2.637817
C	-1.045510	6.624314	3.931003
C	-0.750064	5.480299	4.884371
H	3.008325	5.873177	1.938423
H	0.944298	4.516727	5.771013
H	2.535325	5.981827	4.455042
C	1.951204	6.052194	1.768171
C	-0.750400	6.604730	1.343637
H	-2.107772	6.804606	3.762936
H	-1.547223	4.949266	5.390933
C	0.114422	6.501139	0.243307
C	1.477402	6.209595	0.458891
H	-1.792876	6.866081	1.180533
C	2.433696	6.138696	-0.705330
H	2.131824	5.378349	-1.435718
H	2.462284	7.096287	-1.239503
H	3.447245	5.904324	-0.378200
C	-0.391952	6.775505	-1.150719
H	-0.027435	7.744637	-1.513261
H	-0.044040	6.027551	-1.870466
H	-1.481583	6.802904	-1.190868
C	-0.433631	9.062660	3.650386
C	-0.389068	7.887156	4.605991
C	1.111306	7.626936	4.838258
C	1.825682	8.694688	4.023004
H	-0.930395	8.129942	5.520808
H	1.403768	7.712160	5.886630
C	1.155425	10.496804	2.365830
C	2.154061	11.568621	2.822920
C	1.611756	9.764945	1.094285
H	0.189362	10.971584	2.164879
C	2.449463	12.543488	1.674446
H	3.081201	11.083450	3.140172
H	1.748094	12.100303	3.691702
C	1.929206	10.754022	-0.030610
H	2.502388	9.176328	1.334898
H	0.832469	9.060678	0.790982
C	2.952320	11.800184	0.429027
H	3.181309	13.289033	1.995406
H	1.535562	13.092441	1.409573
H	2.293456	10.194230	-0.898822
H	1.006038	11.258942	-0.348237
H	3.166070	12.515277	-0.371112
H	3.900075	11.299586	0.666411

N	0.868476	9.507888	3.418108
O	3.029929	8.798399	3.880625
O	-1.423494	9.516774	3.113186

Table5_1b_TSiv_DDG_0wat

Property	Value
Charge	0
Electronic Energy, BS1 (a.u.)	-1058.925339
Thermal and entropic correction, BS1 (a.u.)	2.746500
Electronic Energy, BS2 (a.u.)	-1059.282523
Number of Imaginary Frequencies	0
Imaginary frequencies (cm-1)	None

Molecular Geometry in Cartesian Coordinates

C	-8.083408	2.879692	5.687893
C	-8.882787	2.868180	4.542645
C	-8.430053	3.543958	3.355514
C	-7.512984	4.612653	3.528242
C	-7.119742	4.917692	4.877459
C	-7.183913	3.925938	5.860259
H	-9.568536	2.398738	1.927240
H	-8.289429	2.194886	6.506024
H	-9.702427	2.159532	4.457175
C	-8.861296	3.211448	2.055563
C	-7.055907	5.310417	2.392554
H	-6.545383	5.823410	5.061733
H	-6.686143	4.064255	6.817361
C	-7.454675	4.956225	1.113642
C	-8.380898	3.882101	0.942935
H	-6.362928	6.139682	2.524328
C	-8.860552	3.503426	-0.436673
H	-9.464105	4.309745	-0.868923
H	-8.027805	3.323209	-1.126999
H	-9.482817	2.607431	-0.407994
C	-6.939904	5.703928	-0.089362
H	-6.460309	5.029829	-0.808753
H	-7.757424	6.206193	-0.619627
H	-6.211600	6.464690	0.193853
C	-10.516925	4.180856	6.507343
C	-10.137969	4.751733	5.186471
C	-9.258542	5.760187	5.391795
C	-9.011578	5.883922	6.855633
H	-10.750544	4.569907	4.300787
H	-9.008610	6.575280	4.709923
C	-9.776547	4.556567	8.891430
C	-8.401529	4.064568	9.359046
C	-10.259335	5.738817	9.746190
H	-10.498081	3.737898	8.980892
C	-8.438681	3.683305	10.846690
H	-7.671782	4.865370	9.194531
H	-8.094472	3.205151	8.749032
C	-10.306209	5.350027	11.231174
H	-9.567855	6.575125	9.599354
H	-11.246266	6.052733	9.378140
C	-8.935134	4.849660	11.709890
H	-7.442615	3.364356	11.168749
H	-9.103704	2.819963	10.978918
H	-10.628065	6.205994	11.834425
H	-11.052721	4.558323	11.379004
H	-8.994657	4.543732	12.758882
H	-8.212218	5.674608	11.661370
N	-9.779442	4.889576	7.462843
O	-8.294154	6.677031	7.448425
O	-11.298839	3.281767	6.764698
C	-12.387893	-3.826433	7.706447
H	-12.230307	-4.494629	8.562315
H	-11.775500	-2.937987	7.884891
H	-13.439470	-3.515800	7.747585
C	-13.190281	-4.513271	5.495726
H	-14.094863	-4.070818	5.931330
H	-12.978105	-3.976801	4.567072
H	-13.456952	-5.539187	5.212476
C	-11.419692	-5.796804	6.606895
H	-11.990712	-6.562607	6.066479
H	-11.493105	-6.101718	7.658730
C	-9.937429	-5.986176	6.201695
H	-9.911684	-6.826072	5.495639
H	-9.422526	-6.369812	7.091848
C	-8.710991	-5.081443	4.279644
H	-9.221568	-4.447303	3.549508
H	-7.633816	-4.898110	4.179681
H	-8.875150	-6.119059	3.962708

C	-8.119714	-4.374183	6.550227
H	-8.341873	-3.389614	6.970692
H	-7.971517	-5.052382	7.399944
H	-7.151461	-4.304963	6.038886
N	0.115901	5.660810	6.809161
N	-0.788473	8.285152	7.700497
C	-0.226033	4.445159	7.593505
H	-1.280847	4.473898	7.881477
H	-0.060347	3.514979	7.035563
H	0.350968	4.354942	8.522376
C	0.631730	5.297776	5.461778
H	0.015348	5.758462	4.684936
H	1.660870	5.636321	5.287918
H	0.631286	4.215421	5.282469
C	1.099811	6.511017	7.541580
H	1.471043	5.992479	8.434889
H	2.000598	6.680880	6.937917
C	0.618690	7.903683	8.017881
H	1.327011	8.635787	7.609371
H	0.795256	7.936153	9.100727
C	-0.828413	9.539193	6.901358
H	-1.474994	10.306882	7.344420
H	0.157897	10.004023	6.778363
H	-1.204846	9.333585	5.895858
C	-1.597470	8.417636	8.940528
H	-2.375433	7.649142	8.960086
H	-1.006926	8.290011	9.856397
H	-2.092752	9.392951	9.025945
N	-20.106897	3.739554	-2.026401
N	-18.701510	4.988695	-4.257643
C	-19.998300	2.292900	-1.699961
H	-20.361799	2.053554	-0.692792
H	-20.559552	1.650875	-2.390338
H	-18.952819	1.976328	-1.755384
C	-20.718435	4.494784	-0.900675
H	-20.948726	3.860240	-0.035756
H	-20.033921	5.277038	-0.560015
H	-21.656478	4.993398	-1.174690
C	-20.875088	3.946692	-3.288870
H	-21.770371	4.554808	-3.106541
H	-21.270496	2.994856	-3.666044
C	-20.129008	4.609827	-4.473451
H	-20.226500	3.922656	-5.323637
H	-20.726027	5.483304	-4.765515
C	-18.512809	6.456339	-4.405528
H	-17.733221	6.715964	-5.132594
H	-19.422108	6.978115	-4.729249
H	-18.225125	6.893954	-3.445533
C	-17.807053	4.241635	-5.181214
H	-17.156725	4.899251	-5.771461
H	-17.161835	3.567092	-4.611139
H	-18.352949	3.621030	-5.902686
C	-7.452587	16.386550	-0.146481
H	-7.393437	16.647624	0.917609
H	-7.004972	15.395853	-0.265407
H	-6.798870	17.093051	-0.673063
C	-9.810630	16.768637	0.399440
H	-9.326246	16.963005	1.364595
H	-10.383663	17.671780	0.155174
H	-10.538425	15.966972	0.553982
C	-8.977215	17.272738	-1.854302
H	-8.033331	17.794430	-2.057802
H	-9.692985	18.081619	-1.659245
C	-9.412112	16.617783	-3.188717
H	-10.300114	17.167014	-3.526967
H	-8.641373	16.875742	-3.926284
C	-11.089401	14.853647	-3.508897
H	-11.198309	14.160543	-4.352516
H	-11.586322	14.400720	-2.646315
H	-11.666135	15.749428	-3.770603
C	-8.751017	14.441626	-4.112069
H	-9.280643	13.817649	-4.842751
H	-8.116482	15.123323	-4.692187
H	-8.078037	13.788079	-3.550571
N	-5.201662	1.963844	-3.361496
C	-4.501452	2.801505	-2.569051
H	-3.831313	3.492127	-3.060869
C	-4.595618	2.800781	-1.164184
H	-3.981207	3.496271	-0.606876
C	-5.461514	1.886399	-0.513745
C	-6.198485	1.019108	-1.358426
H	-6.877910	0.277991	-0.957240
C	-6.036599	1.094241	-2.755503
H	-6.591506	0.423212	-3.395594
C	-5.568194	1.828477	0.907912
C	-4.894210	2.537609	2.953274
C	-4.127042	3.439135	3.752616
C	-3.347266	4.473996	3.176186

H	-3.292650	4.613603	2.104319
C	-2.610597	5.345250	4.001858
H	-2.009528	6.127414	3.559688
C	-3.339214	4.279687	5.913351
H	-3.322463	4.215591	6.991288
C	-4.100479	3.357406	5.167263
H	-4.651573	2.590654	5.696130
C	-6.416798	0.863532	2.775919
C	-7.330223	-0.051297	3.384154
C	-7.410917	-0.213355	4.790179
H	-6.762480	0.338918	5.457748
C	-8.217324	-0.848040	2.615865
H	-8.226845	-0.805373	1.534376
C	-9.109023	-1.730250	3.256851
H	-9.780585	-2.339939	2.668575
C	-8.342071	-1.114629	5.344770
H	-8.407537	-1.230174	6.416901
C	-16.851593	2.845174	-0.076020
H	-16.865623	2.093412	-0.852065
C	-16.151747	2.597907	1.120776
H	-15.643073	1.649180	1.233857
C	-17.512269	4.931477	0.645497
H	-18.054227	5.844334	0.444558
C	-16.840275	4.775459	1.872932
H	-16.886842	5.583241	2.592010
C	-16.130469	3.578830	2.143665
C	-15.436555	3.374022	3.373850
C	-14.809966	4.120586	5.423368
C	-14.174366	2.056212	4.723946
C	-13.428571	0.854578	4.925986
C	-12.750928	0.581603	6.140710
H	-12.793993	1.267687	6.976777
C	-13.314672	-0.135373	3.917001
H	-13.803847	-0.027281	2.957345
C	-12.554474	-1.297260	4.152073
H	-12.472049	-2.050329	3.381124
C	-12.007597	-0.606765	6.283695
H	-11.489087	-0.806116	7.209783
C	-14.750075	5.173988	6.386155
C	-15.356481	6.434905	6.152768
H	-15.894453	6.646004	5.237418
C	-15.262118	7.453065	7.120856
H	-15.719462	8.414699	6.936100
C	-14.037959	6.102026	8.536919
H	-13.529470	5.987157	9.482852
C	-14.073757	5.028110	7.624138
H	-13.583786	4.100218	7.892162
C	-7.779541	13.005754	0.980763
H	-6.957812	13.189945	0.303635
C	-9.986005	13.374042	1.530234
H	-10.926158	13.850468	1.291398
C	-9.886555	12.567250	2.680189
H	-10.763999	12.447083	3.302621
C	-8.656322	11.940412	3.000753
C	-7.583752	12.182691	2.106560
H	-6.602570	11.755457	2.268114
C	-8.505620	11.118657	4.157848
C	-9.374469	10.210164	6.047652
C	-7.203498	9.830222	5.497966
N	-12.761642	9.441979	8.561182
C	-10.498621	9.964228	6.893882
C	-11.790333	10.464047	6.587627
H	-11.973123	11.063146	5.704645
C	-12.877457	10.175406	7.435437
H	-13.861689	10.551970	7.195026
C	-10.395223	9.197063	8.081656
H	-9.447193	8.787955	8.406648
C	-11.539016	8.964092	8.871966
H	-11.456784	8.382405	9.778060
C	-5.960967	9.169040	5.743756
C	-5.733351	8.404921	6.915447
H	-6.485522	8.322304	7.688530
C	-4.502491	7.746100	7.103359
H	-4.340214	7.157636	7.994474
C	-3.681057	8.548539	5.101949
H	-2.857970	8.601401	4.403416
C	-4.881324	9.232508	4.826172
H	-4.948697	9.804638	3.909717
C	-15.159887	4.369203	-2.858914
H	-15.430732	3.358699	-3.129562
C	-15.837626	6.451125	-2.128588
H	-16.646713	7.096541	-1.817307
C	-14.518700	6.944395	-2.156617
H	-14.347542	7.973151	-1.866484
C	-13.811927	4.773695	-2.916498
H	-13.071943	4.053917	-3.241413
C	-13.455110	6.100508	-2.566061
C	-12.106849	6.564444	-2.636352

C	-10.598878	8.252666	-2.458496
C	-9.930691	6.204958	-3.170148
C	-10.311021	9.627215	-2.202264
C	-11.316096	10.539846	-1.792564
H	-12.341409	10.226289	-1.643194
C	-10.992300	11.893351	-1.577859
H	-11.759980	12.587146	-1.267044
C	-8.777774	11.533288	-2.119882
H	-7.784347	11.941354	-2.241266
C	-9.007851	10.164036	-2.357700
H	-8.174038	9.548083	-2.668219
C	-8.914155	5.349362	-3.692570
C	-7.579677	5.792035	-3.876542
H	-7.273706	6.796397	-3.612928
C	-6.623557	4.926678	-4.443772
H	-5.610394	5.270467	-4.597996
C	-9.187353	4.014531	-4.085429
H	-10.176535	3.585018	-3.990345
C	-8.165675	3.222588	-4.643808
H	-8.380395	2.210346	-4.955100
C	-4.266484	-0.365871	-5.523989
H	-5.274410	-0.318354	-5.101813
H	-4.300936	-1.099481	-6.339132
H	-3.607563	-0.789982	-4.755888
C	-2.599022	1.411070	-5.270383
H	-2.773470	2.346605	-4.731659
H	-2.247470	0.671880	-4.539767
H	-1.758077	1.591792	-5.951539
C	-3.653741	1.025017	-7.452008
H	-2.628608	1.318992	-7.711266
H	-3.761094	0.021856	-7.884222
C	-4.594013	1.950761	-8.262822
H	-3.946376	2.613802	-8.850647
H	-5.078722	1.315158	-9.014993
C	-5.411348	4.195244	-7.701916
H	-5.118215	4.668944	-6.761063
H	-4.624588	4.428035	-8.430346
H	-6.316914	4.702617	-8.056981
C	-6.988188	2.328528	-7.873469
H	-7.608250	3.172122	-8.201696
H	-7.015201	1.592578	-8.686635
H	-7.487499	1.867880	-7.016367
N	-16.210695	8.326103	10.758135
N	-14.291950	10.502769	11.044418
C	-17.346167	8.317606	9.797213
H	-17.772122	7.317590	9.646840
H	-18.178905	8.962118	10.105606
H	-17.013106	8.683141	8.821954
C	-15.970509	6.965842	11.308273
H	-16.640189	6.208123	10.882580
H	-14.944047	6.654766	11.096146
H	-16.097638	6.911269	12.396770
C	-16.451035	9.309693	11.854441
H	-16.492324	8.806286	12.828873
H	-17.439247	9.776512	11.753381
C	-15.433505	10.466863	12.004787
H	-16.013018	11.397819	11.960719
H	-15.071349	10.426230	13.040080
C	-14.307103	11.759673	10.249161
H	-13.359187	12.309923	10.300732
H	-14.499506	11.534889	9.196308
H	-15.087018	12.462400	10.568027
C	-12.995291	10.338582	11.753463
H	-12.278371	11.136482	11.522703
H	-13.099913	10.326323	12.845505
H	-12.533158	9.388130	11.471093
N	-12.015866	-4.443830	6.405921
N	-9.222270	-4.799125	5.647949
N	-3.840342	0.986771	-5.971755
N	-5.607824	2.735019	-7.498322
N	-8.846806	16.381139	-0.664919
N	-9.669362	15.147555	-3.179237
N	-4.818404	2.701228	1.614102
N	-5.652610	1.620394	3.597378
N	-6.424064	0.920913	1.425354
N	-9.174412	-1.867809	4.596585
N	-2.599832	5.255759	5.347247
N	-17.526928	3.987945	-0.318578
N	-14.780140	2.202257	3.524363
N	-15.467129	4.382740	4.272134
N	-14.179489	2.964166	5.725271
N	-14.620981	7.295053	8.296350
N	-11.910473	-1.537812	5.312387
N	-8.955530	13.599263	0.688758
N	-9.593128	10.957219	4.943240
N	-8.197152	9.644005	6.395289
N	-7.296542	10.547126	4.356161
N	-3.487114	7.817300	6.218436

N	-16.160635	5.189646	-2.479597
N	-11.882762	7.856878	-2.312170
N	-9.568516	7.466492	-2.847383
N	-11.169557	5.679708	-3.045882
N	-6.905969	3.665809	-4.831368
N	-9.749689	12.389672	-1.743956
Pd	-1.695027	6.752124	6.518422
Pd	-9.302793	14.373737	-1.226892
Pd	-10.577882	-3.159907	5.486785
Pd	-5.379208	2.350393	-5.413580
Pd	-18.122823	4.477431	-2.272073
Pd	-14.464110	8.884833	9.666091

Table5_1b_TSiv_TSi-ii_0wat

Property	Value
Charge	0
Electronic Energy, BS1 (a.u.)	-1058.883978
Thermal and entropic correction, BS1 (a.u.)	2.750585
Electronic Energy, BS2 (a.u.)	-1059.237227
Number of Imaginary Frequencies	0
Imaginary frequencies (cm-1)	None

Molecular Geometry in Cartesian Coordinates

C	-7.993887	2.934510	5.769160
C	-9.033926	3.105204	4.718091
C	-8.476784	3.640468	3.442579
C	-7.595897	4.721304	3.603509
C	-7.381547	5.130021	5.024855
C	-7.147461	3.965649	5.923245
H	-9.536226	2.399784	2.043427
H	-8.020976	2.090245	6.449012
H	-9.744370	2.286717	4.614432
C	-8.837152	3.223014	2.163959
C	-7.078790	5.360231	2.477664
H	-6.719714	5.981720	5.173085
H	-6.436120	4.026301	6.739509
C	-7.432310	4.944558	1.187597
C	-8.328693	3.864619	1.029247
H	-6.407181	6.207133	2.597174
C	-8.779191	3.433186	-0.344894
H	-9.415349	4.200875	-0.800950
H	-7.936561	3.277863	-1.028124
H	-9.360117	2.510132	-0.303478
C	-6.889661	5.665210	-0.021303
H	-6.389853	4.977237	-0.713270
H	-7.695574	6.150045	-0.585044
H	-6.170790	6.436863	0.256784
C	-10.482313	4.139302	6.682232
C	-9.993691	4.536685	5.316372
C	-9.042777	5.672170	5.533756
C	-8.978252	5.864591	7.031469
H	-10.781189	4.667758	4.579276
H	-9.222768	6.588224	4.976747
C	-9.860895	4.623607	9.067853
C	-8.515405	4.065455	9.558700
C	-10.292937	5.838140	9.898676
H	-10.622177	3.842216	9.158612
C	-8.597413	3.701287	11.048582
H	-7.744136	4.827172	9.401194
H	-8.245355	3.191583	8.957134
C	-10.382410	5.468283	11.387131
H	-9.560400	6.638713	9.754649
H	-11.258554	6.197657	9.515761
C	-9.047106	4.901773	11.893477
H	-7.624165	3.336097	11.390538
H	-9.307654	2.874247	11.180639
H	-10.667071	6.347014	11.975865
H	-11.171971	4.718740	11.531406
H	-9.140318	4.612060	12.944601
H	-8.281618	5.687568	11.848198
N	-9.818017	4.926090	7.625692
O	-8.276363	6.652484	7.638526
O	-11.262243	3.246946	6.954154
C	-12.371357	-3.853770	7.676384
H	-12.205183	-4.526409	8.527146
H	-11.763597	-2.962649	7.857294
H	-13.424629	-3.549899	7.724870
C	-13.180731	-4.531298	5.465401
H	-14.086027	-4.098338	5.909000
H	-12.977165	-3.986401	4.539682
H	-13.441943	-5.556906	5.176024
C	-11.396637	-5.811089	6.559295

H	-11.963974	-6.576561	6.014546
H	-11.465399	-6.124253	7.609028
C	-9.914058	-5.987501	6.149323
H	-9.884174	-6.823415	5.438724
H	-9.393997	-6.372074	7.036065
C	-8.699761	-5.063676	4.228737
H	-9.217991	-4.430456	3.503240
H	-7.624520	-4.870700	4.126255
H	-8.856162	-6.101113	3.907339
C	-8.107027	-4.364089	6.501297
H	-8.335322	-3.383499	6.927812
H	-7.951391	-5.045816	7.346829
H	-7.140801	-4.284836	5.987525
N	0.118396	5.661053	6.762979
N	-0.781879	8.285332	7.658094
C	-0.219530	4.448258	7.553428
H	-1.272595	4.478763	7.847532
H	-0.057824	3.516060	6.997680
H	0.362924	4.360820	8.479157
C	0.626207	5.292939	5.413829
H	0.004851	5.749456	4.638545
H	1.654002	5.631561	5.232353
H	0.625810	4.209832	5.239070
C	1.107142	6.513120	7.486608
H	1.485785	5.995872	8.377561
H	2.003046	6.683281	6.875817
C	0.627979	7.905593	7.965464
H	1.332580	8.638324	7.551678
H	0.812133	7.938808	9.047021
C	-0.829001	9.540577	6.861125
H	-1.472596	10.307098	7.310482
H	0.155956	10.006375	6.731135
H	-1.213332	9.336511	5.858300
C	-1.582899	8.414653	8.903634
H	-2.360006	7.645343	8.926778
H	-0.986230	8.285895	9.815374
H	-2.078631	9.389289	8.994148
N	-20.101631	3.752332	-2.019782
N	-18.712557	4.993678	-4.265495
C	-19.990712	2.306693	-1.689704
H	-20.346767	2.070462	-0.679151
H	-20.556996	1.662566	-2.373992
H	-18.945683	1.989981	-1.751897
C	-20.704582	4.511118	-0.891815
H	-20.927700	3.879469	-0.022893
H	-20.017669	5.294785	-0.559252
H	-21.645004	5.008364	-1.160121
C	-20.879197	3.955609	-3.277121
H	-21.772556	4.565144	-3.090185
H	-21.278185	3.002759	-3.647900
C	-20.141491	4.613774	-4.469708
H	-20.244909	3.923138	-5.316381
H	-20.740614	5.486052	-4.761077
C	-18.525267	6.460846	-4.419900
H	-17.750311	6.718111	-5.152744
H	-19.436725	6.981186	-4.739808
H	-18.231732	6.902033	-3.463351
C	-17.824365	4.243647	-5.192708
H	-17.179568	4.899414	-5.791025
H	-17.173649	3.572537	-4.624748
H	-18.375159	3.619202	-5.907093
C	-7.453061	16.359864	-0.135993
H	-7.402768	16.603688	0.932686
H	-7.004422	15.371219	-0.267350
H	-6.794800	17.074728	-0.645406
C	-9.814909	16.739210	0.394744
H	-9.338046	16.922346	1.365833
H	-10.385186	17.645652	0.156305
H	-10.544565	15.936461	0.534507
C	-8.961836	17.269428	-1.845768
H	-8.016298	17.794260	-2.032837
H	-9.680362	18.075271	-1.648281
C	-9.381603	16.630526	-3.192861
H	-10.264430	17.185366	-3.535479
H	-8.601473	16.895138	-3.918108
C	-11.058727	14.873768	-3.552160
H	-11.159526	14.190591	-4.404809
H	-11.566062	14.412113	-2.700381
H	-11.630634	15.773689	-3.810224
C	-8.714795	14.463270	-4.133685
H	-9.237512	13.848052	-4.876661
H	-8.072655	15.149882	-4.699438
H	-8.049669	13.802199	-3.571523
N	-5.150857	1.910706	-3.395507
C	-4.459160	2.755980	-2.603233
H	-3.785452	3.443404	-3.094642
C	-4.566590	2.769729	-1.199496
H	-3.959843	3.472772	-0.643352

C	-5.438617	1.862235	-0.548291
C	-6.162047	0.982050	-1.390862
H	-6.843819	0.243468	-0.989065
C	-5.986812	1.043789	-2.786743
H	-6.534231	0.364269	-3.424301
C	-5.567003	1.825426	0.872033
C	-4.918171	2.558602	2.916136
C	-4.146864	3.457649	3.713868
C	-3.371388	4.495499	3.137096
H	-3.327813	4.643069	2.065760
C	-2.625150	5.359421	3.961961
H	-2.027127	6.143881	3.519665
C	-3.334841	4.279825	5.872493
H	-3.304925	4.205721	6.949496
C	-4.105608	3.364916	5.127186
H	-4.648787	2.591736	5.655022
C	-6.435748	0.880613	2.740123
C	-7.348786	-0.034298	3.348389
C	-7.425627	-0.199398	4.754042
H	-6.771092	0.347345	5.420073
C	-8.235708	-0.831515	2.580480
H	-8.247743	-0.786678	1.499136
C	-9.125024	-1.715840	3.221806
H	-9.797398	-2.325070	2.633927
C	-8.354044	-1.103134	5.309176
H	-8.415955	-1.221874	6.381147
C	-16.832880	2.862427	-0.091707
H	-16.853560	2.108587	-0.865554
C	-16.124622	2.617469	1.100503
H	-15.616568	1.668264	1.212325
C	-17.485533	4.951562	0.628972
H	-18.027918	5.864505	0.429601
C	-16.804734	4.797914	1.851925
H	-16.845192	5.607387	2.569457
C	-16.094668	3.601021	2.120638
C	-15.393575	3.397485	3.346907
C	-14.762285	4.141479	5.396032
C	-14.135265	2.074746	4.695697
C	-13.404231	0.864551	4.899223
C	-12.737175	0.580604	6.117041
H	-12.779182	1.263459	6.955503
C	-13.298537	-0.125497	3.889523
H	-13.781196	-0.010082	2.927405
C	-12.555553	-1.297887	4.127051
H	-12.479959	-2.051178	3.355598
C	-12.011598	-0.618429	6.262349
H	-11.503539	-0.827635	7.192057
C	-14.706063	5.192449	6.361682
C	-15.297435	6.459387	6.121922
H	-15.821797	6.676920	5.200145
C	-15.201240	7.477085	7.090132
H	-15.644968	8.444186	6.900249
C	-14.011809	6.112029	8.522196
H	-13.518073	5.990405	9.474810
C	-14.047826	5.038805	7.608439
H	-13.575340	4.104219	7.884277
C	-7.790613	12.975076	0.948012
H	-6.965648	13.164527	0.276354
C	-10.002512	13.332361	1.484706
H	-10.942929	13.805805	1.240952
C	-9.906710	12.522571	2.633119
H	-10.787167	12.396305	3.250073
C	-8.675538	11.900571	2.959467
C	-7.598445	12.150231	2.072753
H	-6.615895	11.728486	2.239758
C	-8.526567	11.077588	4.115787
C	-9.391531	10.176762	6.010851
C	-7.220428	9.797775	5.460494
N	-12.758323	9.489888	8.577903
C	-10.509829	9.952256	6.870705
C	-11.804969	10.439928	6.559204
H	-11.995891	11.009702	5.658672
C	-12.884208	10.181095	7.426580
H	-13.869995	10.552000	7.183216
C	-10.395833	9.225427	8.082298
H	-9.444392	8.830504	8.415074
C	-11.531739	9.024763	8.892590
H	-11.438589	8.483015	9.821741
C	-5.973043	9.146704	5.708304
C	-5.736792	8.393400	6.885023
H	-6.485006	8.314016	7.661833
C	-4.500235	7.745584	7.074900
H	-4.331290	7.165941	7.970459
C	-3.689334	8.540218	5.066340
H	-2.868004	8.595171	4.365901
C	-4.895710	9.211977	4.788074
H	-4.969281	9.776250	3.867316
C	-15.158852	4.371424	-2.876126

H	-15.432963	3.361859	-3.147050
C	-15.831362	6.459212	-2.158655
H	-16.639343	7.110434	-1.856789
C	-14.509692	6.945148	-2.176969
H	-14.335922	7.974064	-1.888987
C	-13.808019	4.768023	-2.922576
H	-13.069177	4.043437	-3.239245
C	-13.447008	6.094040	-2.573688
C	-12.095986	6.551309	-2.634074
C	-10.586314	8.238578	-2.462253
C	-9.916222	6.181937	-3.145897
C	-10.301109	9.616820	-2.223324
C	-11.310267	10.533982	-1.834469
H	-12.336397	10.221673	-1.688277
C	-10.990204	11.890934	-1.637966
H	-11.761908	12.587640	-1.344138
C	-8.771809	11.526716	-2.158813
H	-7.778311	11.934512	-2.280759
C	-8.997821	10.153406	-2.377502
H	-8.161325	9.533882	-2.673407
C	-8.898938	5.321793	-3.659645
C	-7.564294	5.763322	-3.846024
H	-7.257791	6.768720	-3.586913
C	-6.609067	4.895944	-4.411549
H	-5.596145	5.238953	-4.569114
C	-9.172297	3.984572	-4.044596
H	-10.161403	3.555735	-3.946092
C	-8.151484	3.189997	-4.601080
H	-8.365763	2.175782	-4.906489
C	-4.255916	-0.376186	-5.617547
H	-5.254835	-0.340657	-5.173374
H	-4.304681	-1.094901	-6.445137
H	-3.579434	-0.811168	-4.871126
C	-2.591262	1.403404	-5.365302
H	-2.759772	2.326768	-4.804144
H	-2.220721	0.652006	-4.656802
H	-1.765743	1.602905	-6.059969
C	-3.688727	1.052568	-7.531970
H	-2.671442	1.359503	-7.806680
H	-3.796804	0.056285	-7.979662
C	-4.653301	1.984461	-8.306606
H	-4.023514	2.662173	-8.897074
H	-5.149885	1.357875	-9.058580
C	-5.475985	4.212864	-7.692145
H	-5.161665	4.674107	-6.752014
H	-4.710635	4.463504	-8.437297
H	-6.394596	4.718341	-8.014965
C	-7.041099	2.335638	-7.856669
H	-7.675518	3.178666	-8.157967
H	-7.080850	1.611281	-8.679714
H	-7.516484	1.858320	-6.995041
N	-16.195889	8.278921	10.744129
N	-14.325853	10.491148	11.065230
C	-17.315968	8.192852	9.768786
H	-17.686266	7.168967	9.632761
H	-18.185980	8.796998	10.055360
H	-16.991996	8.557439	8.790610
C	-15.901826	6.946697	11.334728
H	-16.533331	6.148559	10.924888
H	-14.859999	6.675862	11.141005
H	-16.037257	6.918158	12.423183
C	-16.498611	9.279916	11.808603
H	-16.571935	8.794076	12.790052
H	-17.488898	9.728640	11.657823
C	-15.504515	10.454366	11.979877
H	-16.094288	11.376506	11.900355
H	-15.182090	10.433237	13.028842
C	-14.301709	11.758138	10.285510
H	-13.355120	12.303798	10.386385
H	-14.448795	11.550049	9.222135
H	-15.091329	12.460812	10.579731
C	-13.058986	10.307841	11.821288
H	-12.333368	11.110766	11.640566
H	-13.208334	10.266131	12.907362
H	-12.585967	9.364418	11.532715
N	-12.002133	-4.460579	6.370088
N	-9.209074	-4.792336	5.599986
N	-3.844710	0.986250	-6.049171
N	-5.655793	2.748022	-7.507008
N	-8.842874	16.363155	-0.666318
N	-9.641937	15.160836	-3.203337
N	-4.830917	2.711116	1.576439
N	-5.678252	1.643731	3.561681
N	-6.429767	0.924334	1.389251
N	-9.187077	-1.855957	4.561423
N	-2.600227	5.259430	5.306519
N	-17.508543	4.005354	-0.332361
N	-14.741777	2.223476	3.497049

N	-15.417381	4.406889	4.244402
N	-14.127741	2.985930	5.694637
N	-14.576638	7.312132	8.273499
N	-11.921284	-1.549564	5.290399
N	-8.967158	13.564305	0.650958
N	-9.614832	10.911425	4.898966
N	-8.214590	9.607353	6.357114
N	-7.315566	10.512217	4.317747
N	-3.487083	7.818500	6.187357
N	-16.158586	5.198503	-2.508319
N	-11.871391	7.845909	-2.319867
N	-9.554329	7.446904	-2.835240
N	-11.157542	5.660454	-3.027481
N	-6.892763	3.633696	-4.793422
N	-9.747437	12.387609	-1.802392
Pd	-1.693808	6.752937	6.479600
Pd	-9.298209	14.363656	-1.256917
Pd	-10.576900	-3.162291	5.451813
Pd	-5.376662	2.332306	-5.434492
Pd	-18.119496	4.489368	-2.282595
Pd	-14.458806	8.888662	9.663965

Table5_1b_TSiv_DG_0wat

Property	Value
Charge	0
Electronic Energy, BS1 (a.u.)	-1058.927686
Thermal and entropic correction, BS1 (a.u.)	2.757184
Electronic Energy, BS2 (a.u.)	-1059.278165
Number of Imaginary Frequencies	0
Imaginary frequencies (cm-1)	None

Molecular Geometry in Cartesian Coordinates

C	-8.052588	2.887397	5.711008
C	-9.075413	3.107037	4.619696
C	-8.437758	3.567073	3.340104
C	-7.536099	4.620358	3.512832
C	-7.397124	5.064089	4.946805
C	-7.191618	3.887413	5.875669
H	-9.576241	2.406443	1.934945
H	-8.158443	2.071605	6.428973
H	-9.787183	2.282813	4.503406
C	-8.845886	3.203743	2.063268
C	-7.048202	5.302712	2.400259
H	-6.691780	5.885051	5.100258
H	-6.532042	3.964089	6.740310
C	-7.454675	4.940815	1.105937
C	-8.365488	3.882101	0.935230
H	-6.370633	6.139682	2.532033
C	-8.860552	3.503426	-0.436673
H	-9.464105	4.309745	-0.868923
H	-8.035510	3.323209	-1.134704
H	-9.482817	2.607431	-0.407994
C	-6.939904	5.703928	-0.089362
H	-6.460309	5.037534	-0.816458
H	-7.757424	6.206193	-0.619627
H	-6.211600	6.464690	0.193853
C	-10.501515	4.157741	6.499638
C	-9.968457	4.497466	5.124830
C	-8.973454	5.636906	5.345564
C	-8.980758	5.876217	6.847928
H	-10.789069	4.685483	4.385543
H	-9.178122	6.590690	4.771564
C	-9.784252	4.564272	8.883725
C	-8.401529	4.064568	9.351341
C	-10.259335	5.738817	9.746190
H	-10.505786	3.745603	8.973187
C	-8.438681	3.683305	10.838985
H	-7.671782	4.865370	9.186826
H	-8.102177	3.212856	8.741327
C	-10.306209	5.350027	11.231174
H	-9.567855	6.575125	9.599354
H	-11.246266	6.060438	9.385845
C	-8.935134	4.849660	11.709890
H	-7.442615	3.364356	11.161044
H	-9.103704	2.819963	10.978918
H	-10.628065	6.205994	11.834425
H	-11.052721	4.558323	11.379004
H	-8.994657	4.543732	12.758882
H	-8.212218	5.674608	11.661370
N	-9.787147	4.904986	7.447433
O	-8.301859	6.677031	7.456130
O	-11.306544	3.289472	6.772403

C	-12.387893	-3.826433	7.706447
H	-12.230307	-4.494629	8.562315
H	-11.775500	-2.937987	7.884891
H	-13.439470	-3.515800	7.747585
C	-13.190281	-4.513271	5.495726
H	-14.094863	-4.070818	5.931330
H	-12.978105	-3.976801	4.567072
H	-13.456952	-5.539187	5.212476
C	-11.419692	-5.796804	6.606895
H	-11.990712	-6.562607	6.066479
H	-11.493105	-6.101718	7.658730
C	-9.937429	-5.986176	6.201695
H	-9.911684	-6.826072	5.495639
H	-9.422526	-6.369812	7.091848
C	-8.710991	-5.081443	4.279644
H	-9.221568	-4.447303	3.549508
H	-7.633816	-4.898110	4.179681
H	-8.875150	-6.119059	3.962708
C	-8.119714	-4.374183	6.550227
H	-8.341873	-3.389614	6.970692
H	-7.971517	-5.052382	7.399944
H	-7.151461	-4.304963	6.038886
N	0.115901	5.660810	6.809161
N	-0.788473	8.285152	7.700497
C	-0.226033	4.445159	7.593505
H	-1.280847	4.473898	7.881477
H	-0.060347	3.514979	7.035563
H	0.350968	4.354942	8.522376
C	0.631730	5.297776	5.461778
H	0.015348	5.758462	4.684936
H	1.660870	5.636321	5.287918
H	0.631286	4.215421	5.282469
C	1.099811	6.511017	7.541580
H	1.471043	5.992479	8.434889
H	2.000598	6.680880	6.937917
C	0.618690	7.903683	8.017881
H	1.327011	8.635787	7.609371
H	0.795256	7.936153	9.100727
C	-0.828413	9.539193	6.901358
H	-1.474994	10.306882	7.344420
H	0.157897	10.004023	6.778363
H	-1.204846	9.333585	5.895858
C	-1.597470	8.417636	8.940528
H	-2.375433	7.649142	8.960086
H	-1.006926	8.290011	9.856397
H	-2.092752	9.392951	9.025945
N	-20.106897	3.739554	-2.026401
N	-18.701510	4.988695	-4.257643
C	-19.998300	2.292900	-1.699961
H	-20.361799	2.053554	-0.692792
H	-20.559552	1.650875	-2.390338
H	-18.952819	1.976328	-1.755384
C	-20.718435	4.494784	-0.900675
H	-20.948726	3.860240	-0.035756
H	-20.033921	5.277038	-0.560015
H	-21.656478	4.993398	-1.174690
C	-20.875088	3.946692	-3.288870
H	-21.770371	4.554808	-3.106541
H	-21.270496	2.994856	-3.666044
C	-20.129008	4.609827	-4.473451
H	-20.226500	3.922656	-5.323637
H	-20.726027	5.483304	-4.765515
C	-18.512809	6.456339	-4.405528
H	-17.733221	6.715964	-5.132594
H	-19.422108	6.978115	-4.729249
H	-18.225125	6.893954	-3.445533
C	-17.807053	4.241635	-5.181214
H	-17.156725	4.899251	-5.771461
H	-17.161835	3.567092	-4.611139
H	-18.352949	3.621030	-5.902686
C	-7.452587	16.386550	-0.146481
H	-7.393437	16.647624	0.917609
H	-7.004972	15.395853	-0.265407
H	-6.798870	17.093051	-0.673063
C	-9.810630	16.768637	0.399440
H	-9.326246	16.963005	1.364595
H	-10.383663	17.671780	0.155174
H	-10.538425	15.966972	0.553982
C	-8.977215	17.272738	-1.854302
H	-8.033331	17.794430	-2.057802
H	-9.692985	18.081619	-1.659245
C	-9.412112	16.617783	-3.188717
H	-10.300114	17.167014	-3.526967
H	-8.641373	16.875742	-3.926284
C	-11.089401	14.853647	-3.508897
H	-11.198309	14.160543	-4.352516
H	-11.586322	14.400720	-2.646315
H	-11.666135	15.749428	-3.770603

C	-8.751017	14.441626	-4.112069
H	-9.280643	13.817649	-4.842751
H	-8.116482	15.123323	-4.692187
H	-8.078037	13.788079	-3.550571
N	-5.201662	1.963844	-3.361496
C	-4.501452	2.801505	-2.569051
H	-3.831313	3.492127	-3.060869
C	-4.595618	2.800781	-1.164184
H	-3.981207	3.496271	-0.606876
C	-5.461514	1.886399	-0.513745
C	-6.198485	1.019108	-1.358426
H	-6.877910	0.277991	-0.957240
C	-6.036599	1.094241	-2.755503
H	-6.591506	0.423212	-3.395594
C	-5.568194	1.828477	0.907912
C	-4.894210	2.537609	2.953274
C	-4.127042	3.439135	3.752616
C	-3.347266	4.473996	3.176186
H	-3.292650	4.613603	2.104319
C	-2.610597	5.345250	4.001858
H	-2.009528	6.127414	3.559688
C	-3.339214	4.279687	5.913351
H	-3.322463	4.215591	6.991288
C	-4.100479	3.357406	5.167263
H	-4.651573	2.590654	5.696130
C	-6.416798	0.863532	2.775919
C	-7.330223	-0.051297	3.384154
C	-7.410917	-0.213355	4.790179
H	-6.762480	0.338918	5.457748
C	-8.217324	-0.848040	2.615865
H	-8.226845	-0.805373	1.534376
C	-9.109023	-1.730250	3.256851
H	-9.780585	-2.339939	2.668575
C	-8.342071	-1.114629	5.344770
H	-8.407537	-1.230174	6.416901
C	-16.851593	2.845174	-0.076020
H	-16.865623	2.093412	-0.852065
C	-16.151747	2.597907	1.120776
H	-15.643073	1.649180	1.233857
C	-17.512269	4.931477	0.645497
H	-18.054227	5.844334	0.444558
C	-16.840275	4.775459	1.872932
H	-16.886842	5.583241	2.592010
C	-16.130469	3.578830	2.143665
C	-15.436555	3.374022	3.373850
C	-14.809966	4.120586	5.423368
C	-14.174366	2.056212	4.723946
C	-13.428571	0.854578	4.925986
C	-12.750928	0.581603	6.140710
H	-12.793993	1.267687	6.976777
C	-13.314672	-0.135373	3.917001
H	-13.803847	-0.027281	2.957345
C	-12.554474	-1.297260	4.152073
H	-12.472049	-2.050329	3.381124
C	-12.007597	-0.606765	6.283695
H	-11.489087	-0.806116	7.209783
C	-14.750075	5.173988	6.386155
C	-15.356481	6.434905	6.152768
H	-15.894453	6.646004	5.237418
C	-15.262118	7.453065	7.120856
H	-15.719462	8.414699	6.936100
C	-14.037959	6.102026	8.536919
H	-13.529470	5.987157	9.482852
C	-14.073757	5.028110	7.624138
H	-13.583786	4.100218	7.892162
C	-7.779541	13.005754	0.980763
H	-6.957812	13.189945	0.303635
C	-9.986005	13.374042	1.530234
H	-10.926158	13.850468	1.291398
C	-9.886555	12.567250	2.680189
H	-10.763999	12.447083	3.302621
C	-8.656322	11.940412	3.000753
C	-7.583752	12.182691	2.106560
H	-6.602570	11.755457	2.268114
C	-8.505620	11.118657	4.157848
C	-9.374469	10.210164	6.047652
C	-7.203498	9.830222	5.497966
N	-12.761642	9.441979	8.561182
C	-10.498621	9.964228	6.893882
C	-11.790333	10.464047	6.587627
H	-11.973123	11.063146	5.704645
C	-12.877457	10.175406	7.435437
H	-13.861689	10.551970	7.195026
C	-10.395223	9.197063	8.081656
H	-9.447193	8.787955	8.406648
C	-11.539016	8.964092	8.871966
H	-11.456784	8.382405	9.778060
C	-5.960967	9.169040	5.743756

C	-5.733351	8.404921	6.915447
H	-6.485522	8.322304	7.688530
C	-4.502491	7.746100	7.103359
H	-4.340214	7.157636	7.994474
C	-3.681057	8.548539	5.101949
H	-2.857970	8.601401	4.403416
C	-4.881324	9.232508	4.826172
H	-4.948697	9.804638	3.909717
C	-15.159887	4.369203	-2.858914
H	-15.430732	3.358699	-3.129562
C	-15.837626	6.451125	-2.128588
H	-16.646713	7.096541	-1.817307
C	-14.518700	6.944395	-2.156617
H	-14.347542	7.973151	-1.866484
C	-13.811927	4.773695	-2.916498
H	-13.071943	4.053917	-3.241413
C	-13.455110	6.100508	-2.566061
C	-12.106849	6.564444	-2.636352
C	-10.598878	8.252666	-2.458496
C	-9.930691	6.204958	-3.170148
C	-10.311021	9.627215	-2.202264
C	-11.316096	10.539846	-1.792564
H	-12.341409	10.226289	-1.643194
C	-10.992300	11.893351	-1.577859
H	-11.759980	12.587146	-1.267044
C	-8.777774	11.533288	-2.119882
H	-7.784347	11.941354	-2.241266
C	-9.007851	10.164036	-2.357700
H	-8.174038	9.548083	-2.668219
C	-8.914155	5.349362	-3.692570
C	-7.579677	5.792035	-3.876542
H	-7.273706	6.796397	-3.612928
C	-6.623557	4.926678	-4.443772
H	-5.610394	5.270467	-4.597996
C	-9.187353	4.014531	-4.085429
H	-10.176535	3.585018	-3.990345
C	-8.165675	3.222588	-4.643808
H	-8.380395	2.210346	-4.955100
C	-4.266484	-0.365871	-5.523989
H	-5.274410	-0.318354	-5.101813
H	-4.300936	-1.099481	-6.339132
H	-3.607563	-0.789982	-4.755888
C	-2.599022	1.411070	-5.270383
H	-2.773470	2.346605	-4.731659
H	-2.247470	0.671880	-4.539767
H	-1.758077	1.591792	-5.951539
C	-3.653741	1.025017	-7.452008
H	-2.628608	1.318992	-7.711266
H	-3.761094	0.021856	-7.884222
C	-4.594013	1.950761	-8.262822
H	-3.946376	2.613802	-8.850647
H	-5.078722	1.315158	-9.014993
C	-5.411348	4.195244	-7.701916
H	-5.118215	4.668944	-6.761063
H	-4.624588	4.428035	-8.430346
H	-6.316914	4.702617	-8.056981
C	-6.988188	2.328528	-7.873469
H	-7.608250	3.172122	-8.201696
H	-7.015201	1.592578	-8.686635
H	-7.487499	1.867880	-7.016367
N	-16.210695	8.326103	10.758135
N	-14.291950	10.502769	11.044418
C	-17.346167	8.317606	9.797213
H	-17.772122	7.317590	9.646840
H	-18.178905	8.962118	10.105606
H	-17.013106	8.683141	8.821954
C	-15.970509	6.965842	11.308273
H	-16.640189	6.208123	10.882580
H	-14.944047	6.654766	11.096146
H	-16.097638	6.911269	12.396770
C	-16.451035	9.309693	11.854441
H	-16.492324	8.806286	12.828873
H	-17.439247	9.776512	11.753381
C	-15.433505	10.466863	12.004787
H	-16.013018	11.397819	11.960719
H	-15.071349	10.426230	13.040080
C	-14.307103	11.759673	10.249161
H	-13.359187	12.309923	10.300732
H	-14.499506	11.534889	9.196308
H	-15.087018	12.462400	10.568027
C	-12.995291	10.338582	11.753463
H	-12.278371	11.136482	11.522703
H	-13.099913	10.326323	12.845505
H	-12.533158	9.388130	11.471093
N	-12.015866	-4.443830	6.405921
N	-9.222270	-4.799125	5.647949
N	-3.840342	0.986771	-5.971755
N	-5.607824	2.735019	-7.498322

N	-8.846806	16.381139	-0.664919
N	-9.669362	15.147555	-3.179237
N	-4.818404	2.701228	1.614102
N	-5.652610	1.620394	3.597378
N	-6.424064	0.920913	1.425354
N	-9.174412	-1.867809	4.596585
N	-2.599832	5.255759	5.347247
N	-17.526928	3.987945	-0.318578
N	-14.780140	2.202257	3.524363
N	-15.467129	4.382740	4.272134
N	-14.179489	2.964166	5.725271
N	-14.620981	7.295053	8.296350
N	-11.910473	-1.537812	5.312387
N	-8.955530	13.599263	0.688758
N	-9.593128	10.957219	4.943240
N	-8.197152	9.644005	6.395289
N	-7.296542	10.547126	4.356161
N	-3.487114	7.817300	6.218436
N	-16.160635	5.189646	-2.479597
N	-11.882762	7.856878	-2.312170
N	-9.568516	7.466492	-2.847383
N	-11.169557	5.679708	-3.045882
N	-6.905969	3.665809	-4.831368
N	-9.749689	12.389672	-1.743956
Pd	-1.695027	6.752124	6.518422
Pd	-9.302793	14.373737	-1.226892
Pd	-10.577882	-3.159907	5.486785
Pd	-5.379208	2.350393	-5.413580
Pd	-18.122823	4.477431	-2.272073
Pd	-14.464110	8.884833	9.666091

Table5_1b_TSiii_TSi-ii_h2o_2_2

Property	Value
Charge	0
Electronic Energy, BS1 (a.u.)	-1135.375891
Thermal and entropic correction, BS1 (a.u.)	2.773481
Electronic Energy, BS2 (a.u.)	-1135.769436
Number of Imaginary Frequencies	0
Imaginary frequencies (cm-1)	None

Molecular Geometry in Cartesian Coordinates

C	8.376144	1.050101	6.914237
H	8.759112	0.593798	7.835627
H	7.623323	1.783410	7.216753
H	7.858046	0.251985	6.367957
C	9.539336	1.093463	4.758051
H	8.806610	0.292644	4.597560
H	9.374123	1.847593	3.983454
H	10.524322	0.651656	4.562213
C	10.747368	1.677119	6.812942
H	11.504012	1.160128	6.208997
H	10.680684	1.076216	7.729003
C	11.368300	3.033689	7.226450
H	12.374279	3.060827	6.788325
H	11.549195	2.973180	8.307286
C	11.401722	5.156998	5.998108
H	10.924960	5.232302	5.017158
H	11.504410	6.175540	6.392817
H	12.421468	4.791127	5.824574
C	10.180704	4.989946	8.115301
H	9.090101	4.981233	8.194348
H	10.567387	4.536130	9.036325
H	10.504033	6.038383	8.129395
N	3.147009	15.042966	10.469511
N	0.269486	15.324706	10.864580
C	3.908037	13.869131	10.972827
H	3.356255	12.949389	10.760860
H	4.900076	13.773819	10.513939
H	4.072089	13.893604	12.057416
C	3.956870	15.825996	9.498399
H	3.427363	15.905424	8.545452
H	4.156471	16.852195	9.831801
H	4.935819	15.373755	9.296080
C	2.692228	15.906478	11.598631
H	3.097569	15.547196	12.553220
H	3.095554	16.922686	11.502932
C	1.165236	16.059796	11.804737
H	0.955211	17.136912	11.784966
H	0.963500	15.767321	12.843234
C	-0.608228	16.267219	10.120280
H	-1.674232	16.026559	10.218618
H	-0.497518	17.309055	10.446087

H	-0.365061	16.243630	9.054076
C	-0.537572	14.303866	11.584368
H	-0.256602	13.301527	11.248703
H	-0.390326	14.325357	12.671350
H	-1.616352	14.415686	11.417340
N	2.418164	1.256201	-6.220636
N	2.374955	3.688408	-7.832793
C	3.655952	0.705305	-5.607830
H	3.452319	-0.036531	-4.825418
H	4.315107	0.213694	-6.334166
H	4.236859	1.511939	-5.151632
C	1.207055	0.624580	-5.632684
H	1.443486	-0.100407	-4.843805
H	0.562457	1.390655	-5.192653
H	0.598744	0.087493	-6.371149
C	2.437549	1.094227	-7.703969
H	1.592406	0.480803	-8.041856
H	3.322622	0.529483	-8.023933
C	2.409858	2.385088	-8.559477
H	3.280729	2.332517	-9.225394
H	1.550477	2.290588	-9.235571
C	1.141785	4.450163	-8.165993
H	1.351120	5.463911	-8.529814
H	0.531817	3.966916	-8.939357
H	0.508159	4.543838	-7.279538
C	3.591518	4.492082	-8.124412
H	3.360320	5.496580	-8.500346
H	4.187258	4.609909	-7.214906
H	4.246913	4.027780	-8.871817
C	-6.552385	15.795253	-0.668103
H	-7.085666	15.514835	0.248899
H	-5.490549	15.873190	-0.419671
H	-6.890872	16.807336	-0.923383
C	-7.651358	13.715899	-1.354480
H	-7.991350	13.812960	-0.315758
H	-8.558123	13.647411	-1.968377
H	-7.138208	12.754251	-1.443052
C	-7.254698	15.492721	-3.000850
H	-7.477383	16.548845	-2.801558
H	-8.219711	15.069689	-3.308136
C	-6.345852	15.468253	-4.254789
H	-6.953219	15.051077	-5.068198
H	-6.192392	16.515210	-4.546289
C	-4.979197	13.611753	-5.097476
H	-4.099766	13.650095	-5.752333
H	-4.939674	12.666400	-4.549411
H	-5.852388	13.554824	-5.759400
C	-3.902073	15.682996	-4.354898
H	-3.217444	15.349065	-5.144603
H	-4.219008	16.696240	-4.631808
H	-3.319840	15.776556	-3.433888
N	8.056390	13.640080	-0.028209
C	7.163145	14.189851	0.820775
H	6.839523	15.197726	0.603055
C	6.658022	13.521641	1.954104
H	5.954741	14.042610	2.590269
C	7.092425	12.205675	2.251855
C	8.028745	11.641900	1.348901
H	8.425433	10.644652	1.490115
C	8.471105	12.384546	0.237449
H	9.184277	11.949164	-0.447704
C	6.624887	11.492637	3.396945
C	5.386130	11.416932	5.296571
C	4.435190	12.019933	6.176363
C	3.895111	13.307767	5.928132
H	4.179011	13.884952	5.057565
C	2.984672	13.878582	6.838619
H	2.581314	14.864107	6.653557
C	3.065474	12.023995	8.207365
H	2.722232	11.533157	9.106973
C	3.982904	11.375008	7.355851
H	4.335085	10.391242	7.633637
C	6.717704	9.664931	4.738138
C	7.218262	8.353899	5.002459
C	6.906769	7.651993	6.194572
H	6.296536	8.096786	6.968973
C	8.059225	7.675834	4.083772
H	8.354682	8.125136	3.144709
C	8.523774	6.379696	4.377654
H	9.157514	5.862299	3.671921
C	7.421508	6.356943	6.404726
H	7.187165	5.824169	7.314784
C	3.469998	3.066030	-2.939343
H	4.396443	3.074941	-3.495934
C	3.499555	3.104769	-1.531770
H	4.463056	3.134236	-1.039287
C	1.165583	2.996028	-2.969605
H	0.256542	2.945787	-3.551721

C	1.096089	3.033217	-1.563031
H	0.121160	2.999590	-1.094477
C	2.287482	3.088557	-0.796487
C	2.269423	3.115894	0.630652
C	1.114286	3.137003	2.583363
C	3.383703	3.131999	2.606778
C	4.614980	3.156351	3.328199
C	4.660203	3.084462	4.742806
H	3.757868	2.979866	5.330945
C	5.866799	3.258056	2.670996
H	5.940208	3.310766	1.592859
C	7.055675	3.298152	3.424011
H	8.007754	3.380227	2.919494
C	5.899986	3.132137	5.410753
H	5.931195	3.075475	6.489085
C	-0.129955	3.230796	3.277507
C	-1.369079	3.272066	2.589315
H	-1.420841	3.225455	1.509379
C	-2.573573	3.358535	3.312887
H	-3.518119	3.382530	2.787981
C	-1.454516	3.385894	5.330492
H	-1.507167	3.440449	6.408338
C	-0.200947	3.298204	4.692096
H	0.692334	3.282394	5.303340
C	-3.932208	13.811746	0.692829
H	-3.708597	14.850064	0.494181
C	-4.906150	11.846232	-0.013924
H	-5.461281	11.318544	-0.776831
C	-4.530415	11.176551	1.167208
H	-4.818979	10.140143	1.285613
C	-3.818119	11.869701	2.177654
C	-3.519350	13.228697	1.906121
H	-2.992798	13.848588	2.620218
C	-3.445010	11.244744	3.405493
C	-3.484700	9.446854	4.785801
C	-2.502081	11.376443	5.464888
N	-4.495744	5.345588	5.541299
C	-3.829090	8.084779	5.038618
C	-4.513607	7.291185	4.084699
H	-4.828412	7.696263	3.131338
C	-4.818557	5.947199	4.377183
H	-5.350537	5.347909	3.651929
C	-3.501979	7.442790	6.259592
H	-2.996624	7.968657	7.059649
C	-3.847692	6.092135	6.459109
H	-3.603125	5.609952	7.394111
C	-1.765650	12.109425	6.443358
C	-1.396828	11.536563	7.685506
H	-1.682971	10.527323	7.949663
C	-0.627509	12.278551	8.601136
H	-0.345661	11.832862	9.543813
C	-0.573000	14.103577	7.203127
H	-0.252180	15.121280	7.033286
C	-1.333317	13.439807	6.220699
H	-1.577915	13.969374	5.309551
C	3.369699	6.066210	-4.951176
H	4.312842	5.587217	-5.172512
C	1.066188	5.950878	-4.942414
H	0.175045	5.380075	-5.162429
C	0.953722	7.248369	-4.404960
H	-0.036327	7.650950	-4.235616
C	3.355476	7.370291	-4.418401
H	4.302609	7.866683	-4.248261
C	2.120838	8.006020	-4.132042
C	2.056646	9.337923	-3.621089
C	0.833333	11.133927	-2.965359
C	3.098809	11.257336	-3.000384
C	-0.434636	11.767994	-2.795681
C	-1.651514	11.093307	-3.067663
H	-1.668370	10.065494	-3.406383
C	-2.878020	11.772181	-2.933685
H	-3.804076	11.259815	-3.153480
C	-1.823627	13.714727	-2.269850
H	-1.910930	14.746933	-1.961411
C	-0.551522	13.118163	-2.377356
H	0.319824	13.720684	-2.155457
C	4.297813	12.013418	-2.830923
C	4.277334	13.367253	-2.411523
H	3.348885	13.885621	-2.211226
C	5.484758	14.078257	-2.269787
H	5.464121	15.113140	-1.959662
C	5.579159	11.458302	-3.076782
H	5.700387	10.433149	-3.402982
C	6.733606	12.246681	-2.901462
H	7.708410	11.819534	-3.089946
C	11.155603	14.495368	-0.762298
H	10.751660	13.498475	-0.959621
H	12.130069	14.545009	-1.264180

H	11.360956	14.560436	0.313539
C	9.795867	16.428207	-0.116692
H	8.709927	16.399657	0.010864
H	10.246712	16.145198	0.842721
H	10.066197	17.478004	-0.285695
C	10.778954	16.316490	-2.363601
H	10.858929	17.379348	-2.101776
H	11.814566	16.011369	-2.561081
C	10.039011	16.255818	-3.722808
H	9.832942	17.295268	-4.008639
H	10.775205	15.916481	-4.462695
C	7.613781	16.276097	-4.100064
H	6.923703	16.277302	-3.251850
H	7.871191	17.324162	-4.297958
H	7.056100	15.925567	-4.977516
C	8.940715	14.334004	-4.783768
H	8.142205	14.332122	-5.536149
H	9.885740	14.375458	-5.339551
H	8.915629	13.364738	-4.277429
N	-4.408497	1.120869	5.804081
N	-6.328501	3.125094	6.696963
C	-4.259250	0.592396	4.421326
H	-3.294595	0.096792	4.255643
H	-5.030144	-0.141591	4.154675
H	-4.340773	1.405721	3.695841
C	-3.232227	0.762518	6.639990
H	-2.461197	0.216493	6.082052
H	-2.770484	1.668949	7.041444
H	-3.490787	0.134821	7.502016
C	-5.673274	0.627195	6.423763
H	-5.460435	0.004743	7.302321
H	-6.208759	-0.046507	5.742692
C	-6.699437	1.691855	6.883512
H	-7.642665	1.459581	6.372533
H	-6.908854	1.480098	7.939951
C	-7.338035	3.828982	5.861071
H	-7.741131	4.727634	6.344679
H	-6.889991	4.140639	4.913436
H	-8.203258	3.202506	5.610271
C	-6.156379	3.806418	8.007100
H	-6.781456	4.702456	8.109089
H	-6.397250	3.163502	8.862957
H	-5.114774	4.116318	8.130244
N	9.433784	1.713326	6.106275
N	10.603772	4.269197	6.885584
N	10.199466	15.532667	-1.233480
N	8.797222	15.430763	-3.789436
N	-6.743618	14.817645	-1.772147
N	-5.041495	14.751015	-4.143407
N	5.715013	12.111148	4.183976
N	5.855560	10.194780	5.638228
N	7.127919	10.255228	3.594649
N	8.219794	5.728126	5.517997
N	2.578989	13.258672	7.964917
N	2.327868	3.007187	-3.654199
N	3.463284	3.184741	1.258218
N	1.059497	3.123300	1.231891
N	2.241334	3.105730	3.330864
N	-2.624799	3.406467	4.659173
N	7.083456	3.234849	4.770846
N	-4.620882	13.142829	-0.254517
N	-3.780389	9.943821	3.563509
N	-2.848056	10.106544	5.775582
N	-2.760076	11.999801	4.292403
N	-0.212011	13.542261	8.376391
N	2.249024	5.364573	-5.218946
N	0.827883	9.842986	-3.367384
N	1.932480	11.876019	-2.704591
N	3.228548	9.984099	-3.434552
N	6.696852	13.537602	-2.510637
N	-2.969890	13.061516	-2.550379
Pd	1.446504	14.288663	9.416407
Pd	-4.838120	13.943206	-2.179993
Pd	8.832138	3.741161	5.823079
Pd	8.445353	14.520981	-1.894318
Pd	2.346126	3.331225	-5.731448
Pd	-4.463532	3.252951	5.671391
C	2.777787	5.650682	6.019180
C	3.004641	6.079818	4.703504
C	1.885507	6.344372	3.851590
C	0.649307	6.651087	4.477354
C	0.634944	6.717546	5.905667
C	1.580405	5.981350	6.639196
H	2.932262	6.162124	1.982312
H	3.584872	5.212600	6.600985
H	3.977948	5.958232	4.235178
C	1.959242	6.324808	2.440287
C	-0.486102	6.864395	3.670168

H	-0.247639	7.113437	6.398654
H	1.438779	5.809531	7.702893
C	-0.428794	6.750791	2.292419
C	0.828021	6.492595	1.661190
H	-1.431962	7.097885	4.150539
C	0.910617	6.428712	0.156908
H	0.229926	5.675072	-0.250403
H	1.924535	6.189881	-0.170691
H	0.627203	7.385262	-0.298662
C	-1.662436	6.971211	1.454817
H	-1.540942	7.840037	0.797857
H	-2.539099	7.154787	2.078668
H	-1.880163	6.120609	0.802236
C	3.202613	8.955612	4.038696
C	3.201311	8.300984	5.373032
C	2.051306	8.643024	6.015497
C	1.285009	9.554671	5.133249
H	4.139207	7.979740	5.822002
H	1.853634	8.630426	7.083598
C	1.646965	10.487302	2.785183
C	0.243549	10.160865	2.251796
C	1.797065	11.979617	3.124477
H	2.383861	10.230931	2.016350
C	-0.105659	11.045311	1.046838
H	-0.480984	10.319815	3.053823
H	0.207501	9.105492	1.983582
C	1.437742	12.851095	1.914815
H	1.133991	12.212157	3.968928
H	2.826407	12.172802	3.447084
C	0.028114	12.533233	1.398987
H	-1.119921	10.813528	0.699793
H	0.575406	10.808184	0.216892
H	1.521368	13.910079	2.173048
H	2.162659	12.666614	1.110548
H	-0.206208	13.159338	0.531302
H	-0.700044	12.786263	2.181117
N	2.002253	9.645205	3.930494
O	0.263929	10.169006	5.383983
O	4.081647	8.957685	3.181856
O	5.379908	6.514472	2.608876
H	5.602408	6.492236	1.669861
H	5.056553	7.423997	2.759368

Table5_1b_TSiii_TSi-ii_h2o_1wat

Property	Value
Charge	0
Electronic Energy, BS1 (a.u.)	-1135.336660
Thermal and entropic correction, BS1 (a.u.)	2.775370
Electronic Energy, BS2 (a.u.)	-1135.726038
Number of Imaginary Frequencies	0
Imaginary frequencies (cm-1)	None

Molecular Geometry in Cartesian Coordinates

C	8.377789	1.049798	6.914644
H	8.761251	0.594217	7.836187
H	7.624602	1.782819	7.216954
H	7.860033	0.251092	6.368897
C	9.540242	1.092941	4.758051
H	8.807907	0.291651	4.598136
H	9.374334	1.846662	3.983206
H	10.525396	0.651590	4.562037
C	10.748675	1.678003	6.812291
H	11.505508	1.161621	6.208062
H	10.682749	1.076993	7.728337
C	11.368693	3.035024	7.225696
H	12.374553	3.062905	6.787344
H	11.549867	2.974624	8.306492
C	11.400417	5.158589	5.997745
H	10.923955	5.233194	5.016603
H	11.501718	6.177314	6.392344
H	12.420667	4.793907	5.824662
C	10.179397	4.990158	8.114825
H	9.088779	4.980683	8.193670
H	10.566219	4.536394	9.035814
H	10.502026	6.038806	8.129209
N	3.146608	15.042402	10.470113
N	0.269096	15.324708	10.864908
C	3.907255	13.868389	10.973590
H	3.355255	12.948782	10.761571
H	4.899333	13.772800	10.514846
H	4.071154	13.892855	12.058203
C	3.956812	15.825158	9.499065

H	3.427348	15.904895	8.546124
H	4.156845	16.851249	9.832551
H	4.935572	15.372516	9.296722
C	2.691890	15.906138	11.599084
H	3.097140	15.546947	12.553747
H	3.095347	16.922282	11.503251
C	1.164910	16.059689	11.805094
H	0.955064	17.136839	11.785280
H	0.963060	15.767275	12.843585
C	-0.608437	16.267343	10.120535
H	-1.674479	16.026811	10.218769
H	-0.497643	17.309160	10.446370
H	-0.365177	16.243752	9.054349
C	-0.538158	14.304009	11.584663
H	-0.257387	13.301630	11.248965
H	-0.390915	14.325454	12.671646
H	-1.616915	14.416046	11.417630
N	2.417356	1.255979	-6.221801
N	2.374232	3.688744	-7.833104
C	3.655234	0.704748	-5.609489
H	3.451716	-0.037332	-4.827279
H	4.314159	0.213315	-6.336154
H	4.236331	1.511176	-5.153160
C	1.206320	0.624314	-5.633737
H	1.442855	-0.100845	-4.845044
H	0.561859	1.390343	-5.193418
H	0.597831	0.087423	-6.372197
C	2.436324	1.094502	-7.705192
H	1.590958	0.481382	-8.043069
H	3.321195	0.529672	-8.025567
C	2.408708	2.385664	-8.560251
H	3.279423	2.333140	-9.226374
H	1.549170	2.291556	-9.236200
C	1.141174	4.450899	-8.165821
H	1.350683	5.464713	-8.529362
H	0.530928	3.968063	-8.939223
H	0.507738	4.544439	-7.279221
C	3.590918	4.492263	-8.124621
H	3.359870	5.496961	-8.500106
H	4.186826	4.609608	-7.215161
H	4.246090	4.028113	-8.872312
C	-6.552256	15.795585	-0.669668
H	-7.085846	15.515763	0.247336
H	-5.490467	15.873381	-0.421011
H	-6.890412	16.807627	-0.925552
C	-7.651316	13.715978	-1.355174
H	-7.991251	13.813465	-0.316474
H	-8.558120	13.647363	-1.969000
H	-7.138278	12.754242	-1.443418
C	-7.254375	15.491994	-3.002344
H	-7.477387	16.548125	-2.803442
H	-8.219195	15.068632	-3.309775
C	-6.345153	15.467358	-4.256011
H	-6.952268	15.050061	-5.069547
H	-6.191619	16.514272	-4.547621
C	-4.978178	13.610704	-5.097949
H	-4.098505	13.648912	-5.752484
H	-4.938851	12.665464	-4.549663
H	-5.851133	13.553626	-5.760172
C	-3.901355	15.682118	-4.355429
H	-3.216357	15.347944	-5.144710
H	-4.218222	16.695244	-4.632847
H	-3.319545	15.776026	-3.434184
N	8.057211	13.640252	-0.027104
C	7.163859	14.190023	0.821805
H	6.840334	15.197934	0.604107
C	6.658585	13.521831	1.955088
H	5.955282	14.042794	2.591233
C	7.092884	12.205845	2.252847
C	8.029263	11.642045	1.349967
H	8.425904	10.644778	1.491163
C	8.471815	12.384697	0.238607
H	9.185099	11.949295	-0.446420
C	6.625202	11.492787	3.397868
C	5.386215	11.416884	5.297349
C	4.435173	12.019831	6.177084
C	3.895112	13.307671	5.928850
H	4.179172	13.884893	5.058359
C	2.984441	13.878397	6.839168
H	2.581053	14.863913	6.654099
C	3.065080	12.023770	8.207855
H	2.721714	11.532897	9.107398
C	3.982713	11.374861	7.356480
H	4.337653	10.391131	7.634313
C	6.717560	9.664778	4.738684
C	7.217722	8.353494	5.002521
C	6.905635	7.651037	6.194147
H	6.295074	8.095506	6.968457

C	8.058766	7.675652	4.083728
H	8.354625	8.125325	3.144970
C	8.522802	6.379207	4.377034
H	9.156510	5.861945	3.671175
C	7.419956	6.355734	6.403791
H	7.185138	5.822517	7.313467
C	3.469737	3.064625	-2.939971
H	4.396147	3.073188	-3.496625
C	3.499426	3.103512	-1.532408
H	4.462976	3.132755	-1.040010
C	1.165313	2.995236	-2.970033
H	0.256211	2.945083	-3.552064
C	1.095936	3.032659	-1.563458
H	0.121037	2.999366	-1.094817
C	2.287413	3.087729	-0.797025
C	2.269494	3.115216	0.630109
C	1.114541	3.136926	2.582923
C	3.383953	3.131024	2.606120
C	4.615281	3.155003	3.327457
C	4.660578	3.082402	4.742018
H	3.758328	2.977338	5.330185
C	5.867067	3.257069	2.670248
H	5.940428	3.310313	1.592131
C	7.055965	3.296947	3.423249
H	8.008021	3.379349	2.918737
C	5.900378	3.129874	5.409930
H	5.931600	3.072682	6.488232
C	-0.129600	3.231448	3.277143
C	-1.368756	3.272816	2.589029
H	-1.420582	3.225818	1.509114
C	-2.573184	3.359758	3.312658
H	-3.517744	3.383707	2.787777
C	-1.453983	3.387699	5.330155
H	-1.506522	3.442733	6.407983
C	-0.200474	3.299498	4.691704
H	0.692852	3.283789	5.302884
C	-3.932091	13.811600	0.692271
H	-3.708461	14.849903	0.493563
C	-4.906383	11.846154	-0.014141
H	-5.461812	11.318484	-0.776842
C	-4.530378	11.176470	1.166897
H	-4.819048	10.140104	1.285429
C	-3.817884	11.869647	2.177194
C	-3.518961	13.228571	1.905479
H	-2.992202	13.848470	2.619417
C	-3.444906	11.244838	3.405146
C	-3.485203	9.447216	4.785778
C	-2.502563	11.376838	5.464798
N	-4.496423	5.346036	5.541651
C	-3.829717	8.085197	5.038716
C	-4.514173	7.291501	4.084840
H	-4.828931	7.696438	3.131399
C	-4.819179	5.947568	4.377483
H	-5.351175	5.348201	3.652309
C	-3.502761	7.443355	6.259803
H	-2.997526	7.969328	7.059863
C	-3.848485	6.092718	6.459437
H	-3.604036	5.610661	7.394540
C	-1.766279	12.109846	6.443371
C	-1.397810	11.537000	7.685627
H	-1.684262	10.527860	7.949822
C	-0.628432	12.278855	8.601315
H	-0.346851	11.833148	9.544061
C	-0.573258	14.103772	7.203196
H	-0.252166	15.121390	7.033363
C	-1.333629	13.440129	6.220708
H	-1.578010	13.969699	5.309508
C	3.369379	6.065629	-4.951167
H	4.312463	5.586832	-5.173176
C	1.065891	5.950062	-4.941335
H	0.174707	5.379259	-5.161182
C	0.953538	7.247294	-4.403246
H	-0.036502	7.649628	-4.233202
C	3.355280	7.369489	-4.417841
H	4.302433	7.865943	-4.247984
C	2.120716	8.004980	-4.130648
C	2.056645	9.336750	-3.619336
C	0.833360	11.132742	-2.963617
C	3.098831	11.256240	-2.998863
C	-0.434607	11.766925	-2.794407
C	-1.651478	11.092147	-3.066213
H	-1.668335	10.064128	-3.404286
C	-2.877954	11.771198	-2.932917
H	-3.804010	11.258802	-3.152635
C	-1.823555	13.714046	-2.269955
H	-1.910842	14.746438	-1.962130
C	-0.551473	13.117329	-2.376852
H	0.319884	13.719899	-2.155128

C	4.297819	12.012374	-2.829468
C	4.277276	13.366220	-2.410075
H	3.348827	13.884589	-2.209807
C	5.484657	14.077281	-2.268342
H	5.463999	15.112168	-1.958243
C	5.579180	11.457297	-3.075312
H	5.700398	10.432133	-3.401484
C	6.733598	12.245722	-2.899953
H	7.708428	11.818613	-3.088380
C	11.155825	14.495818	-0.762400
H	10.751929	13.498785	-0.959113
H	12.130124	14.545318	-1.264619
H	11.361535	14.561432	0.313336
C	9.796001	16.428735	-0.117249
H	8.710089	16.400149	0.010540
H	10.247055	16.146127	0.842184
H	10.066252	17.478477	-0.286730
C	10.778517	16.316223	-2.364353
H	10.858584	17.379165	-2.102892
H	11.814057	16.011041	-2.562111
C	10.038066	16.255119	-3.723257
H	9.831739	17.294468	-4.009270
H	10.774027	15.915712	-4.463342
C	7.612690	16.274943	-4.099582
H	6.922909	16.276227	-3.251126
H	7.869848	17.323012	-4.297783
H	7.054771	15.924142	-4.976776
C	8.939631	14.332862	-4.783299
H	8.140789	14.330642	-5.535328
H	9.884411	14.374320	-5.339499
H	8.914931	13.363738	-4.276675
N	-4.406577	1.121388	5.804187
N	-6.327834	3.124440	6.696930
C	-4.256433	0.592904	4.421533
H	-3.291186	0.098365	4.256134
H	-5.026436	-0.141994	4.154813
H	-4.338708	1.406040	3.695919
C	-3.230414	0.763876	6.640609
H	-2.458983	0.217934	6.083142
H	-2.769184	1.670664	7.041838
H	-3.488974	0.136431	7.502819
C	-5.671293	0.626927	6.423361
H	-5.458389	0.004261	7.301751
H	-6.206324	-0.046764	5.741922
C	-6.698021	1.690974	6.883253
H	-7.641128	1.458271	6.372247
H	-6.907325	1.478931	7.939657
C	-7.337759	3.827965	5.861190
H	-7.741418	4.726232	6.345045
H	-6.889886	4.140176	4.913657
H	-8.202599	3.201052	5.610169
C	-6.156007	3.805648	8.007167
H	-6.781505	4.701376	8.109301
H	-6.396536	3.162495	8.862942
H	-5.114553	4.116049	8.130320
N	9.434821	1.713257	6.106081
N	10.603162	4.269969	6.885027
N	10.199422	15.532790	-1.233768
N	8.796378	15.429858	-3.789199
N	-6.743458	14.817470	-1.773265
N	-5.040822	14.750151	-4.144129
N	5.715278	12.111262	4.184879
N	5.855457	10.194596	5.638849
N	7.128155	10.255356	3.595495
N	8.218287	5.727150	5.516944
N	2.578545	13.258420	7.965353
N	2.327542	3.006011	-3.654746
N	3.463427	3.183884	1.257565
N	1.059626	3.123051	1.231457
N	2.241647	3.105197	3.330323
N	-2.624333	3.408190	4.658939
N	7.083831	3.233027	4.770061
N	-4.621123	13.142739	-0.254861
N	-3.780476	9.943982	3.563304
N	-2.848691	10.106997	5.775591
N	-2.760070	11.999975	4.292078
N	-0.212566	13.542440	8.376542
N	2.248643	5.363995	-5.218701
N	0.827920	9.841692	-3.365235
N	1.932493	11.874883	-2.702961
N	3.228565	9.983025	-3.433126
N	6.696756	13.536650	-2.509128
N	-2.969803	13.060757	-2.550373
Pd	1.446044	14.288493	9.416822
Pd	-4.838005	13.942686	-2.180547
Pd	8.832048	3.740654	5.822359
Pd	8.445312	14.520521	-1.893729
Pd	2.345666	3.330825	-5.731878

Pd	-4.462948	3.253429	5.671345
C	2.770990	5.649316	6.031921
C	3.023662	6.188953	4.726685
C	1.879905	6.352548	3.845296
C	0.652643	6.659239	4.468656
C	0.691094	6.817783	5.910792
C	1.590901	5.974541	6.641178
H	2.930954	6.159550	1.983839
H	3.562933	5.151257	6.580718
H	3.983319	6.012503	4.250352
C	1.958377	6.318502	2.442173
C	-0.485394	6.862850	3.672758
H	-0.210580	7.175730	6.400877
H	1.407829	5.747971	7.685643
C	-0.428941	6.750998	2.289516
C	0.821654	6.490450	1.660671
H	-1.427838	7.106735	4.153366
C	0.909039	6.428453	0.156376
H	0.226882	5.675825	-0.253064
H	1.922419	6.187687	-0.171473
H	0.627237	7.385317	-0.299530
C	-1.662457	6.976385	1.455038
H	-1.539374	7.844964	0.797993
H	-2.538500	7.161909	2.079190
H	-1.884873	6.126205	0.802593
C	3.202594	8.942509	4.037299
C	3.184198	8.200432	5.332881
C	1.983935	8.561501	5.999827
C	1.278962	9.545084	5.136202
H	4.124373	8.047944	5.839791
H	1.888439	8.665290	7.070643
C	1.650587	10.488696	2.788794
C	0.247534	10.162263	2.254489
C	1.797881	11.980972	3.128219
H	2.387941	10.229577	2.023152
C	-0.101034	11.046831	1.049455
H	-0.480285	10.320984	3.056037
H	0.209160	9.106954	1.986066
C	1.439116	12.852486	1.915694
H	1.134438	12.213559	3.969653
H	2.827103	12.176782	3.448527
C	0.029713	12.534694	1.399202
H	-1.115063	10.812283	0.701760
H	0.577878	10.809907	0.217197
H	1.522685	13.911467	2.173973
H	2.164359	12.667989	1.111709
H	-0.204211	13.160920	0.531509
H	-0.698867	12.787566	2.181000
N	2.007798	9.646359	3.939654
O	0.266271	10.173423	5.384003
O	4.081747	8.961160	3.183252
O	5.379003	6.514284	2.609276
H	5.601351	6.492551	1.670215
H	5.058143	7.423652	2.757546

Table5_1b_TSiii_DG_h2o_2

Property	Value
Charge	0
Electronic Energy, BS1 (a.u.)	-1135.380676
Thermal and entropic correction, BS1 (a.u.)	2.781115
Electronic Energy, BS2 (a.u.)	-1135.767313
Number of Imaginary Frequencies	0
Imaginary frequencies (cm-1)	None

Molecular Geometry in Cartesian Coordinates

C	8.376144	1.050101	6.914237
H	8.759112	0.593798	7.835627
H	7.623323	1.783410	7.216753
H	7.858046	0.251985	6.367957
C	9.539336	1.093463	4.758051
H	8.806610	0.292644	4.597560
H	9.374123	1.847593	3.983454
H	10.524322	0.651656	4.562213
C	10.747368	1.677119	6.812942
H	11.504012	1.160128	6.208997
H	10.680684	1.076216	7.729003
C	11.368300	3.033689	7.226450
H	12.374279	3.060827	6.788325
H	11.549195	2.973180	8.307286
C	11.401722	5.156998	5.998108
H	10.924960	5.232302	5.017158
H	11.504410	6.175540	6.392817

H	12.421468	4.791127	5.824574
C	10.180704	4.989946	8.115301
H	9.090101	4.981233	8.194348
H	10.567387	4.536130	9.036325
H	10.504033	6.038383	8.129395
N	3.147009	15.042966	10.469511
N	0.269486	15.324706	10.864580
C	3.908037	13.869131	10.972827
H	3.356255	12.949389	10.760860
H	4.900076	13.773819	10.513939
H	4.072089	13.893604	12.057416
C	3.956870	15.825996	9.498399
H	3.427363	15.905424	8.545452
H	4.156471	16.852195	9.831801
H	4.935819	15.373755	9.296080
C	2.692228	15.906478	11.598631
H	3.097569	15.547196	12.553220
H	3.095554	16.922686	11.502932
C	1.165236	16.059796	11.804737
H	0.955211	17.136912	11.784966
H	0.963500	15.767321	12.843234
C	-0.608228	16.267219	10.120280
H	-1.674232	16.026559	10.218618
H	-0.497518	17.309055	10.446087
H	-0.365061	16.243630	9.054076
C	-0.537572	14.303866	11.584368
H	-0.256602	13.301527	11.248703
H	-0.390326	14.325357	12.671350
H	-1.616352	14.415686	11.417340
N	2.418164	1.256201	-6.220636
N	2.374955	3.688408	-7.832793
C	3.655952	0.705305	-5.607830
H	3.452319	-0.036531	-4.825418
H	4.315107	0.213694	-6.334166
H	4.236859	1.511939	-5.151632
C	1.207055	0.624580	-5.632684
H	1.443486	-0.100407	-4.843805
H	0.562457	1.390655	-5.192653
H	0.598744	0.087493	-6.371149
C	2.437549	1.094227	-7.703969
H	1.592406	0.480803	-8.041856
H	3.322622	0.529483	-8.023933
C	2.409858	2.385088	-8.559477
H	3.280729	2.332517	-9.225394
H	1.550477	2.290588	-9.235571
C	1.141785	4.450163	-8.165993
H	1.351120	5.463911	-8.529814
H	0.531817	3.966916	-8.939357
H	0.508159	4.543838	-7.279538
C	3.591518	4.492082	-8.124412
H	3.360320	5.496580	-8.500346
H	4.187258	4.609909	-7.214906
H	4.246913	4.027780	-8.871817
C	-6.552385	15.795253	-0.668103
H	-7.085666	15.514835	0.248899
H	-5.490549	15.873190	-0.419671
H	-6.890872	16.807336	-0.923383
C	-7.651358	13.715899	-1.354480
H	-7.991350	13.812960	-0.315758
H	-8.558123	13.647411	-1.968377
H	-7.138208	12.754251	-1.443052
C	-7.254698	15.492721	-3.000850
H	-7.477383	16.548845	-2.801558
H	-8.219711	15.069689	-3.308136
C	-6.345852	15.468253	-4.254789
H	-6.953219	15.051077	-5.068198
H	-6.192392	16.515210	-4.546289
C	-4.979197	13.611753	-5.097476
H	-4.099766	13.650095	-5.752333
H	-4.939674	12.666400	-4.549411
H	-5.852388	13.554824	-5.759400
C	-3.902073	15.682996	-4.354898
H	-3.217444	15.349065	-5.144603
H	-4.219008	16.696240	-4.631808
H	-3.319840	15.776556	-3.433888
N	8.056390	13.640080	-0.028209
C	7.163145	14.189851	0.820775
H	6.839523	15.197726	0.603055
C	6.658022	13.521641	1.954104
H	5.954741	14.042610	2.590269
C	7.092425	12.205675	2.251855
C	8.028745	11.641900	1.348901
H	8.425433	10.644652	1.490115
C	8.471105	12.384546	0.237449
H	9.184277	11.949164	-0.447704
C	6.624887	11.492637	3.396945
C	5.386130	11.416932	5.296571
C	4.435190	12.019933	6.176363

C	3.895111	13.307767	5.928132
H	4.179011	13.884952	5.057565
C	2.984672	13.878582	6.838619
H	2.581314	14.864107	6.653557
C	3.065474	12.023995	8.207365
H	2.722232	11.533157	9.106973
C	3.982904	11.375008	7.355851
H	4.340750	10.391242	7.633637
C	6.717704	9.664931	4.738138
C	7.218262	8.353899	5.002459
C	6.906769	7.651993	6.194572
H	6.296536	8.096786	6.968973
C	8.059225	7.675834	4.083772
H	8.354682	8.125136	3.144709
C	8.523774	6.379696	4.377654
H	9.157514	5.862299	3.671921
C	7.421508	6.356943	6.404726
H	7.187165	5.824169	7.314784
C	3.469998	3.066030	-2.939343
H	4.396443	3.074941	-3.495934
C	3.499555	3.104769	-1.531770
H	4.463056	3.134236	-1.039287
C	1.165583	2.996028	-2.969605
H	0.256542	2.945787	-3.551721
C	1.096089	3.033217	-1.563031
H	0.121160	2.999590	-1.094477
C	2.287482	3.088557	-0.796487
C	2.269423	3.115894	0.630652
C	1.114286	3.137003	2.583363
C	3.383703	3.131999	2.606778
C	4.614980	3.156351	3.328199
C	4.660203	3.084462	4.742806
H	3.757868	2.979866	5.330945
C	5.866799	3.258056	2.670996
H	5.940208	3.310766	1.592859
C	7.055675	3.298152	3.424011
H	8.007754	3.380227	2.919494
C	5.899986	3.132137	5.410753
H	5.931195	3.075475	6.489085
C	-0.129955	3.230796	3.277507
C	-1.369079	3.272066	2.589315
H	-1.420841	3.225455	1.509379
C	-2.573573	3.358535	3.312887
H	-3.518119	3.382530	2.787981
C	-1.454516	3.385894	5.330492
H	-1.507167	3.440449	6.408338
C	-0.200947	3.298204	4.692096
H	0.692334	3.282394	5.303340
C	-3.932208	13.811746	0.692829
H	-3.708597	14.850064	0.494181
C	-4.906150	11.846232	-0.013924
H	-5.461281	11.318544	-0.776831
C	-4.530415	11.176551	1.167208
H	-4.818979	10.140143	1.285613
C	-3.818119	11.869701	2.177654
C	-3.519350	13.228697	1.906121
H	-2.992798	13.848588	2.620218
C	-3.445010	11.244744	3.405493
C	-3.484700	9.446854	4.785801
C	-2.502081	11.376443	5.464888
N	-4.495744	5.345588	5.541299
C	-3.829090	8.084779	5.038618
C	-4.513607	7.291185	4.084699
H	-4.828412	7.696263	3.131338
C	-4.818557	5.947199	4.377183
H	-5.350537	5.347909	3.651929
C	-3.501979	7.442790	6.259592
H	-2.996624	7.968657	7.059649
C	-3.847692	6.092135	6.459109
H	-3.603125	5.609952	7.394111
C	-1.765650	12.109425	6.443358
C	-1.396828	11.536563	7.685506
H	-1.682971	10.527323	7.949663
C	-0.627509	12.278551	8.601136
H	-0.345661	11.832862	9.543813
C	-0.573000	14.103577	7.203127
H	-0.252180	15.121280	7.033286
C	-1.333317	13.439807	6.220699
H	-1.577915	13.969374	5.309551
C	3.369699	6.066210	-4.951176
H	4.312842	5.587217	-5.172512
C	1.066188	5.950878	-4.942414
H	0.175045	5.380075	-5.162429
C	0.953722	7.248369	-4.404960
H	-0.036327	7.650950	-4.235616
C	3.355476	7.370291	-4.418401
H	4.302609	7.866683	-4.248261
C	2.120838	8.006020	-4.132042

C	2.056646	9.337923	-3.621089
C	0.833333	11.133927	-2.965359
C	3.098809	11.257336	-3.000384
C	-0.434636	11.767994	-2.795681
C	-1.651514	11.093307	-3.067663
H	-1.668370	10.065494	-3.406383
C	-2.878020	11.772181	-2.933685
H	-3.804076	11.259815	-3.153480
C	-1.823627	13.714727	-2.269850
H	-1.910930	14.746933	-1.961411
C	-0.551522	13.118163	-2.377356
H	0.319824	13.720684	-2.155457
C	4.297813	12.013418	-2.830923
C	4.277334	13.367253	-2.411523
H	3.348885	13.885621	-2.211226
C	5.484758	14.078257	-2.269787
H	5.464121	15.113140	-1.959662
C	5.579159	11.458302	-3.076782
H	5.700387	10.433149	-3.402982
C	6.733606	12.246681	-2.901462
H	7.708410	11.819534	-3.089946
C	11.155603	14.495368	-0.762298
H	10.751660	13.498475	-0.959621
H	12.130069	14.545009	-1.264180
H	11.360956	14.560436	0.313539
C	9.795867	16.428207	-0.116692
H	8.709927	16.399657	0.010864
H	10.246712	16.145198	0.842721
H	10.066197	17.478004	-0.285695
C	10.778954	16.316490	-2.363601
H	10.858929	17.379348	-2.101776
H	11.814566	16.011369	-2.561081
C	10.039011	16.255818	-3.722808
H	9.832942	17.295268	-4.008639
H	10.775205	15.916481	-4.462695
C	7.613781	16.276097	-4.100064
H	6.923703	16.277302	-3.251850
H	7.871191	17.324162	-4.297958
H	7.056100	15.925567	-4.977516
C	8.940715	14.334004	-4.783768
H	8.142205	14.332122	-5.536149
H	9.885740	14.375458	-5.339551
H	8.915629	13.364738	-4.277429
N	-4.408497	1.120869	5.804081
N	-6.328501	3.125094	6.696963
C	-4.259250	0.592396	4.421326
H	-3.294595	0.096792	4.255643
H	-5.030144	-0.141591	4.154675
H	-4.340773	1.405721	3.695841
C	-3.232227	0.762518	6.639990
H	-2.461197	0.216493	6.082052
H	-2.770484	1.668949	7.041444
H	-3.490787	0.134821	7.502016
C	-5.673274	0.627195	6.423763
H	-5.460435	0.004743	7.302321
H	-6.208759	-0.046507	5.742692
C	-6.699437	1.691855	6.883512
H	-7.642665	1.459581	6.372533
H	-6.908854	1.480098	7.939951
C	-7.338035	3.828982	5.861071
H	-7.741131	4.727634	6.344679
H	-6.889991	4.140639	4.913436
H	-8.203258	3.202506	5.610271
C	-6.156379	3.806418	8.007100
H	-6.781456	4.702456	8.109089
H	-6.397250	3.163502	8.862957
H	-5.114774	4.116318	8.130244
N	9.433784	1.713326	6.106275
N	10.603772	4.269197	6.885584
N	10.199466	15.532667	-1.233480
N	8.797222	15.430763	-3.789436
N	-6.743618	14.817645	-1.772147
N	-5.041495	14.751015	-4.143407
N	5.715013	12.111148	4.183976
N	5.855560	10.194780	5.638228
N	7.127919	10.255228	3.594649
N	8.219794	5.728126	5.517997
N	2.578989	13.258672	7.964917
N	2.327868	3.007187	-3.654199
N	3.463284	3.184741	1.258218
N	1.059497	3.123300	1.231891
N	2.241334	3.105730	3.330864
N	-2.624799	3.406467	4.659173
N	7.083456	3.234849	4.770846
N	-4.620882	13.142829	-0.254517
N	-3.780389	9.943821	3.563509
N	-2.848056	10.106544	5.775582
N	-2.760076	11.999801	4.292403

N	-0.212011	13.542261	8.376391
N	2.249024	5.364573	-5.218946
N	0.827883	9.842986	-3.367384
N	1.932480	11.876019	-2.704591
N	3.228548	9.984099	-3.434552
N	6.696852	13.537602	-2.510637
N	-2.969890	13.061516	-2.550379
Pd	1.446504	14.288663	9.416407
Pd	-4.838120	13.943206	-2.179993
Pd	8.832138	3.741161	5.823079
Pd	8.445353	14.520981	-1.894318
Pd	2.346126	3.331225	-5.731448
Pd	-4.463532	3.252951	5.671391
C	2.766457	5.650682	6.047505
C	3.044296	6.312079	4.754489
C	1.874177	6.361367	3.840260
C	0.654972	6.662417	4.460359
C	0.748242	6.915818	5.916997
C	1.603065	5.964355	6.644861
H	2.932262	6.162124	1.987977
H	3.545217	5.093637	6.561330
H	3.989278	6.077195	4.269167
C	1.959242	6.313478	2.445952
C	-0.486102	6.853065	3.675833
H	-0.173995	7.232400	6.404319
H	1.376465	5.679238	7.668904
C	-0.428794	6.745126	2.286754
C	0.816691	6.486930	1.661190
H	-1.426297	7.103550	4.156204
C	0.910617	6.428712	0.156908
H	0.229926	5.675072	-0.256068
H	1.924535	6.189881	-0.170691
H	0.627203	7.385262	-0.298662
C	-1.662436	6.971211	1.454817
H	-1.540942	7.840037	0.797857
H	-2.539099	7.154787	2.078668
H	-1.885828	6.120609	0.802236
C	3.202613	8.927287	4.033031
C	3.167322	8.091382	5.288058
C	1.915348	8.473077	5.981508
C	1.273679	9.532011	5.138914
H	4.110882	8.121363	5.855991
H	1.927278	8.698405	7.055273
C	1.652630	10.487302	2.790848
C	0.249214	10.160865	2.257461
C	1.797065	11.979617	3.130142
H	2.389526	10.225266	2.027680
C	-0.099994	11.045311	1.052503
H	-0.480984	10.319815	3.059488
H	0.207501	9.105492	1.989247
C	1.437742	12.851095	1.914815
H	1.133991	12.212157	3.968928
H	2.826407	12.178467	3.447084
C	0.028114	12.533233	1.398987
H	-1.114256	10.807863	0.705458
H	0.575406	10.808184	0.216892
H	1.521368	13.910079	2.173048
H	2.162659	12.666614	1.110548
H	-0.206208	13.159338	0.531302
H	-0.700044	12.786263	2.181117
N	2.013583	9.645205	3.947489
O	0.269594	10.174671	5.383983
O	4.081647	8.963350	3.181856
O	5.379908	6.514472	2.608876
H	5.602408	6.492236	1.669861
H	5.062218	7.423997	2.753703

Table5_1b_TSiii_reactant_2wat

Property	Value
Charge	0
Electronic Energy, BS1 (a.u.)	-1211.818734
Thermal and entropic correction, BS1 (a.u.)	2.796168
Electronic Energy, BS2 (a.u.)	-1212.247176
Number of Imaginary Frequencies	0
Imaginary frequencies (cm-1)	None

Molecular Geometry in Cartesian Coordinates

C	8.411921	1.027625	6.966465
H	8.782614	0.593506	7.903488
H	7.629942	1.741877	7.239207
H	7.931910	0.208300	6.416770
C	9.628227	1.081205	4.840096

H	8.923933	0.257025	4.670629
H	9.458852	1.823092	4.054658
H	10.630737	0.668250	4.672113
C	10.766929	1.718749	6.919019
H	11.545893	1.194966	6.350313
H	10.686914	1.145978	7.851830
C	11.358327	3.097219	7.302288
H	12.373418	3.125233	6.885875
H	11.516051	3.074403	8.388164
C	11.378314	5.163861	5.979202
H	10.904329	5.190775	4.994189
H	11.470951	6.199878	6.328377
H	12.401554	4.799119	5.825238
C	10.151578	5.080455	8.098111
H	9.061527	5.060305	8.179452
H	10.544817	4.675680	9.039075
H	10.460425	6.132671	8.063210
N	3.102113	15.071578	10.543915
N	0.213656	15.331234	10.864523
C	3.867517	13.904846	11.057080
H	3.332720	12.978997	10.828603
H	4.870221	13.825045	10.618857
H	4.008378	13.925436	12.145024
C	3.921952	15.869982	9.594062
H	3.413729	15.942117	8.628871
H	4.096369	16.898617	9.933860
H	4.912652	15.434290	9.414067
C	2.611796	15.922598	11.667640
H	2.993952	15.558134	12.629811
H	3.011138	16.941981	11.590818
C	1.078902	16.065260	11.833651
H	0.862943	17.141228	11.814927
H	0.850878	15.765042	12.864471
C	-0.642497	16.274133	10.096013
H	-1.710552	16.031518	10.161303
H	-0.543438	17.315390	10.427308
H	-0.366514	16.253645	9.037685
C	-0.612886	14.306946	11.556901
H	-0.319354	13.305805	11.228366
H	-0.499343	14.326586	12.647927
H	-1.686267	14.417055	11.357167
N	2.416789	1.301875	-6.324937
N	2.329560	3.872458	-7.705287
C	3.667258	0.709156	-5.780186
H	3.479501	-0.102847	-5.066510
H	4.320752	0.291591	-6.556322
H	4.248595	1.475525	-5.259780
C	1.218951	0.606333	-5.783732
H	1.472155	-0.195519	-5.078851
H	0.581282	1.319421	-5.253354
H	0.598277	0.146086	-6.562669
C	2.420494	1.278297	-7.817069
H	1.572258	0.697597	-8.201613
H	3.302269	0.746331	-8.196702
C	2.386415	2.642748	-8.549476
H	3.262249	2.661926	-9.210635
H	1.532927	2.601748	-9.238327
C	1.076978	4.632585	-7.959276
H	1.260198	5.680156	-8.229040
H	0.471158	4.208471	-8.769774
H	0.449271	4.628479	-7.064095
C	3.525425	4.727479	-7.929359
H	3.268599	5.754120	-8.218851
H	4.121583	4.783482	-7.014287
H	4.189188	4.344002	-8.714305
C	-6.518695	15.734352	-0.599248
H	-6.994077	15.434317	0.343132
H	-5.446569	15.833149	-0.409255
H	-6.892119	16.741081	-0.825414
C	-7.615228	13.641082	-1.246609
H	-7.904095	13.724525	-0.191455
H	-8.550321	13.559630	-1.814746
H	-7.090218	12.689618	-1.368267
C	-7.337330	15.438600	-2.894593
H	-7.565656	16.489384	-2.674781
H	-8.310522	15.002227	-3.153507
C	-6.496524	15.438771	-4.195306
H	-7.136482	15.010837	-4.977578
H	-6.382628	16.490078	-4.489450
C	-5.129349	13.624415	-5.125743
H	-4.289205	13.695525	-5.827753
H	-5.030104	12.675098	-4.592057
H	-6.036026	13.547879	-5.738785
C	-4.065753	15.712684	-4.412200
H	-3.415367	15.405402	-5.240700
H	-4.419124	16.721996	-4.657337
H	-3.438648	15.806474	-3.521065
N	8.101363	13.690459	0.080530

C	7.179647	14.215798	0.914394
H	6.858267	15.226794	0.707236
C	6.645850	13.524184	2.019913
H	5.922315	14.030982	2.644913
C	7.083571	12.207421	2.308757
C	8.053714	11.669635	1.427433
H	8.467168	10.679757	1.572298
C	8.520279	12.434970	0.341826
H	9.262617	12.018545	-0.323909
C	6.600000	11.476730	3.435228
C	5.348650	11.390537	5.325799
C	4.417916	12.007070	6.217080
C	3.901766	13.306377	5.977251
H	4.186405	13.880915	5.105407
C	3.016754	13.895450	6.900017
H	2.633083	14.890041	6.722233
C	3.076494	12.036848	8.266421
H	2.736203	11.552917	9.170967
C	3.968747	11.369472	7.401777
H	4.309713	10.379372	7.675190
C	6.676134	9.635833	4.759090
C	7.172923	8.322133	5.018986
C	6.841582	7.609528	6.199500
H	6.218735	8.047561	6.968040
C	8.028006	7.651014	4.108019
H	8.342207	8.109834	3.179770
C	8.482777	6.349306	4.395365
H	9.125172	5.836300	3.694346
C	7.347104	6.309875	6.403676
H	7.095014	5.767248	7.303129
C	3.458120	2.857531	-2.895365
H	4.384297	2.825879	-3.451422
C	3.493873	2.935327	-1.489763
H	4.459144	2.952177	-1.000483
C	1.157552	2.834797	-2.917995
H	0.244844	2.781090	-3.494443
C	1.091713	2.917483	-1.513634
H	0.117325	2.917208	-1.043001
C	2.285224	2.971693	-0.751601
C	2.270963	3.043506	0.673498
C	1.114407	3.138867	2.622373
C	3.383485	3.073666	2.650193
C	4.613873	3.080896	3.374154
C	4.654422	3.051930	4.790416
H	3.748837	2.990182	5.378808
C	5.869956	3.124828	2.718714
H	5.947595	3.134963	1.639623
C	7.057441	3.163827	3.474648
H	8.012386	3.203905	2.970628
C	5.893362	3.092695	5.460561
H	5.920465	3.068308	6.540460
C	-0.132057	3.243246	3.310544
C	-1.367785	3.273827	2.615709
H	-1.414831	3.222717	1.535777
C	-2.576566	3.344489	3.332914
H	-3.518933	3.353448	2.803954
C	-1.468185	3.389205	5.356716
H	-1.528151	3.443253	6.434172
C	-0.210323	3.315856	4.724471
H	0.679962	3.305487	5.339974
C	-3.826006	13.774236	0.618920
H	-3.626527	14.818300	0.423889
C	-4.782889	11.797183	-0.081888
H	-5.347173	11.267275	-0.836469
C	-4.367933	11.124573	1.083768
H	-4.637499	10.082795	1.199040
C	-3.648601	11.822888	2.085921
C	-3.374748	13.187931	1.817659
H	-2.844014	13.810345	2.526630
C	-3.256554	11.197665	3.307752
C	-3.314787	9.406747	4.695664
C	-2.334027	11.336839	5.375869
N	-4.485769	5.360527	5.519946
C	-3.703886	8.060412	4.966744
C	-4.390302	7.267420	4.013790
H	-4.664995	7.658499	3.042629
C	-4.756133	5.944835	4.333573
H	-5.295101	5.347953	3.611335
C	-3.430269	7.437574	6.210111
H	-2.931015	7.965395	7.012480
C	-3.832354	6.106874	6.433946
H	-3.631227	5.641244	7.387448
C	-1.641318	12.081202	6.377128
C	-1.302420	11.512262	7.629522
H	-1.578768	10.498335	7.884832
C	-0.583243	12.268075	8.573576
H	-0.326393	11.826585	9.525251
C	-0.518847	14.097452	7.183259

H	-0.214443	15.122405	7.027502
C	-1.230005	13.420873	6.172666
H	-1.454314	13.947042	5.254395
C	3.344370	5.982231	-4.628996
H	4.288251	5.503869	-4.849071
C	1.039617	5.877602	-4.665032
H	0.150167	5.315951	-4.913648
C	0.923796	7.161496	-4.096639
H	-0.067614	7.563402	-3.933486
C	3.326066	7.270695	-4.059709
H	4.272421	7.759971	-3.868840
C	2.089251	7.907778	-3.786332
C	2.020854	9.237731	-3.270844
C	0.792440	11.077670	-2.771279
C	3.056771	11.168540	-2.680894
C	-0.471032	11.739967	-2.720689
C	-1.682544	11.068438	-3.020152
H	-1.698868	10.021680	-3.294039
C	-2.901700	11.772945	-2.997430
H	-3.821999	11.260845	-3.241174
C	-1.852327	13.734314	-2.397291
H	-1.934976	14.785652	-2.161208
C	-0.586727	13.115908	-2.400032
H	0.281495	13.720720	-2.172543
C	4.253916	11.931525	-2.528278
C	4.228794	13.301886	-2.165623
H	3.299605	13.821967	-1.972227
C	5.431597	14.029640	-2.081032
H	5.405548	15.077159	-1.817081
C	5.536339	11.376130	-2.768219
H	5.661936	10.343242	-3.066184
C	6.685588	12.184377	-2.660672
H	7.660674	11.762427	-2.860838
C	11.157417	14.567269	-0.812620
H	10.761786	13.559943	-0.968336
H	12.106616	14.621020	-1.360414
H	11.411402	14.659924	0.250833
C	9.794712	16.490550	-0.146580
H	8.717428	16.441118	0.037134
H	10.299581	16.240615	0.795016
H	10.033089	17.541503	-0.352373
C	10.675625	16.345814	-2.434135
H	10.735401	17.417388	-2.204379
H	11.711246	16.063528	-2.663132
C	9.887521	16.225415	-3.762254
H	9.652212	17.250804	-4.075044
H	10.601819	15.873358	-4.517486
C	7.449989	16.193385	-4.045924
H	6.795401	16.216412	-3.170611
H	7.681697	17.237255	-4.292199
H	6.862768	15.802462	-4.886394
C	8.783909	14.251521	-4.717572
H	7.960544	14.214920	-5.441798
H	9.708937	14.287285	-5.306453
H	8.789519	13.298932	-4.179743
N	-4.450497	1.141292	5.837438
N	-6.355154	3.178006	6.688240
C	-4.300073	0.594479	4.461928
H	-3.342631	0.081083	4.308891
H	-5.081236	-0.129483	4.197783
H	-4.362831	1.400670	3.726640
C	-3.282608	0.779678	6.683734
H	-2.515849	0.216740	6.136838
H	-2.811063	1.685483	7.075195
H	-3.553040	0.166786	7.552704
C	-5.724115	0.669990	6.456615
H	-5.523591	0.063364	7.349080
H	-6.259280	-0.012833	5.784456
C	-6.745891	1.751575	6.885728
H	-7.683224	1.523751	6.362107
H	-6.974922	1.555398	7.941109
C	-7.346581	3.885818	5.834368
H	-7.743547	4.794268	6.304447
H	-6.883572	4.183016	4.889157
H	-8.216696	3.267597	5.579937
C	-6.188001	3.870679	7.993035
H	-6.802712	4.775505	8.079359
H	-6.446043	3.239596	8.852648
H	-5.143882	4.168217	8.124301
N	9.471675	1.711510	6.178329
N	10.584994	4.309472	6.902743
N	10.162715	15.577736	-1.261727
N	8.659085	15.377932	-3.754435
N	-6.750371	14.762707	-1.700827
N	-5.171770	14.751955	-4.156481
N	5.678603	12.085039	4.214482
N	5.798812	10.156573	5.650366
N	7.093328	10.233647	3.621670

N	8.156553	5.686718	5.523209
N	2.613827	13.282203	8.030202
N	2.315101	2.806323	-3.610932
N	3.466469	3.094149	1.300586
N	1.061986	3.103418	1.272043
N	2.239586	3.097363	3.372492
N	-2.634551	3.392238	4.678492
N	7.081502	3.148614	4.823157
N	-4.526808	13.100594	-0.316510
N	-3.587994	9.895458	3.465651
N	-2.664535	10.060994	5.679696
N	-2.583102	11.957981	4.199636
N	-0.186830	13.540688	8.367546
N	2.225515	5.296036	-4.938096
N	0.788526	9.767495	-3.103266
N	1.887284	11.817818	-2.487195
N	3.193342	9.867029	-3.024125
N	6.643473	13.491581	-2.329789
N	-2.996191	13.084350	-2.695948
Pd	1.436202	14.301541	9.450245
Pd	-4.853946	13.927331	-2.216788
Pd	8.821780	3.718845	5.858281
Pd	8.398379	14.523994	-1.819310
Pd	2.327599	3.321164	-5.645365
Pd	-4.479928	3.271635	5.679133
C	2.624972	5.687886	5.799759
C	2.908409	5.978542	4.474198
C	1.868283	6.389938	3.593689
C	0.640578	6.824673	4.160226
C	0.533617	6.867246	5.580062
C	1.422638	6.143519	6.367664
H	2.961388	6.053422	1.768699
H	3.383028	5.247527	6.445054
H	3.901312	5.809398	4.055453
C	1.991646	6.325672	2.182614
C	-0.434960	7.125016	3.294154
H	-0.314464	7.374661	6.034161
H	1.262791	6.051719	7.443497
C	-0.333513	6.966821	1.926657
C	0.918878	6.567923	1.351475
H	-1.374606	7.454966	3.723732
C	-1.504901	7.289722	1.032613
H	-1.256494	8.108503	0.346653
H	-2.375737	7.599223	1.615293
C	1.056059	6.451393	-0.144600
H	0.304563	5.778978	-0.567225
H	2.049283	6.086124	-0.414387
C	3.300383	8.948567	3.755053
C	3.302689	8.355590	5.110694
C	2.243656	8.766746	5.775124
C	1.506253	9.728152	4.928605
H	4.218668	7.814619	5.479824
H	1.992119	8.670242	6.844806
C	1.872277	10.671648	2.591062
C	0.490247	10.331912	2.019444
C	1.991664	12.154332	2.976763
H	2.617177	10.464874	1.816998
C	0.161646	11.230316	0.826599
H	-0.260089	10.444761	2.808657
H	0.496999	9.286193	1.714902
C	1.662925	13.037586	1.768509
H	1.298021	12.367775	3.801440
H	3.006392	12.354254	3.339963
C	0.271680	12.712673	1.208848
H	-0.838747	10.995918	0.443728
H	0.882262	11.001577	0.027386
H	1.726624	14.093572	2.042456
H	2.413485	12.868249	0.982922
H	0.057002	13.357857	0.347453
H	-0.480439	12.947371	1.974170
N	2.207984	9.789974	3.706296
O	0.551855	10.428124	5.212830
O	4.119547	8.806628	2.836758
O	5.446704	6.324049	2.624030
H	5.718107	6.193262	1.707803
H	5.118715	7.243658	2.664389
O	2.977860	8.930136	0.083140
H	3.385973	8.765317	0.951393
H	3.717303	8.949414	-0.531987
H	-1.795503	6.441055	0.408037
H	0.938292	7.432624	-0.614388

Table5_1b_TSiii_TSi-ii_h2o_2

Property	Value

Charge	0
Electronic Energy, BS1 (a.u.)	-1211.775935
Thermal and entropic correction, BS1 (a.u.)	2.794825
Electronic Energy, BS2 (a.u.)	-1212.202233
Number of Imaginary Frequencies	0
Imaginary frequencies (cm-1)	None

Molecular Geometry in Cartesian Coordinates

C	8.409098	1.027597	6.966042
H	8.778778	0.592755	7.903126
H	7.627482	1.742310	7.238592
H	7.928806	0.208918	6.415621
C	9.626932	1.081089	4.840537
H	8.922655	0.256999	4.670559
H	9.457933	1.823258	4.055291
H	10.629486	0.668165	4.672761
C	10.764633	1.717034	6.920535
H	11.543262	1.191777	6.352729
H	10.683288	1.145015	7.853690
C	11.357676	3.094975	7.303172
H	12.372738	3.121665	6.886605
H	11.515522	3.072367	8.389036
C	11.379819	5.161276	5.979532
H	10.906015	5.188095	4.994403
H	11.473161	6.197396	6.328216
H	12.402819	4.795686	5.825981
C	10.153018	5.079723	8.098482
H	9.062930	5.061005	8.179684
H	10.545562	4.674604	9.039586
H	10.463247	6.131532	8.063466
N	3.101670	15.072253	10.543564
N	0.213045	15.330934	10.863537
C	3.867423	13.905890	11.057048
H	3.332973	12.979814	10.828654
H	4.870206	13.826348	10.618957
H	4.008131	13.926721	12.145005
C	3.921379	15.870809	9.593716
H	3.413373	15.942442	8.628361
H	4.095232	16.899614	9.933284
H	4.912331	15.435540	9.414088
C	2.610821	15.923278	11.667057
H	2.993186	15.559269	12.629317
H	3.009541	16.942886	11.589995
C	1.077838	16.065002	11.833049
H	0.861303	17.140859	11.814764
H	0.849897	15.764293	12.863744
C	-0.642938	16.273792	10.094781
H	-1.711020	16.031253	10.159955
H	-0.543837	17.315099	10.425904
H	-0.366797	16.253184	9.036509
C	-0.613608	14.306473	11.555539
H	-0.319963	13.305399	11.226898
H	-0.500335	14.325893	12.646595
H	-1.686945	14.416577	11.355556
N	2.416422	1.302558	-6.323177
N	2.329524	3.872487	-7.704742
C	3.667238	0.710321	-5.778695
H	3.479957	-0.101496	-5.064685
H	4.320527	0.292662	-6.554949
H	4.248607	1.477002	-5.258780
C	1.218936	0.607080	-5.781107
H	1.472567	-0.194100	-5.075618
H	0.581158	1.320407	-5.251170
H	0.598245	0.145954	-6.559511
C	2.419455	1.278244	-7.815298
H	1.570741	0.697816	-8.199206
H	3.300803	0.745639	-8.195030
C	2.385831	2.642358	-8.548354
H	3.261608	2.660893	-9.209608
H	1.532252	2.601355	-9.237092
C	1.077344	4.633105	-7.959228
H	1.261092	5.680528	-8.229200
H	0.471549	4.209058	-8.769780
H	0.449415	4.629497	-7.064198
C	3.525821	4.726831	-7.929067
H	3.269535	5.753422	-8.219222
H	4.121784	4.783113	-7.013882
H	4.189585	4.342576	-8.713631
C	-6.520729	15.733337	-0.598596
H	-6.996630	15.432814	0.343369
H	-5.448752	15.832302	-0.407909
H	-6.894195	16.740094	-0.824559
C	-7.616254	13.639989	-1.247442
H	-7.905745	13.722920	-0.192418
H	-8.550984	13.558382	-1.816152

H	-7.090832	12.688766	-1.369177
C	-7.338230	15.438408	-2.894412
H	-7.566807	16.489057	-2.674207
H	-8.311288	15.002013	-3.153800
C	-6.497017	15.439238	-4.194861
H	-7.136739	15.011632	-4.977507
H	-6.383077	16.490679	-4.488508
C	-5.129776	13.624964	-5.125375
H	-4.289573	13.696116	-5.827311
H	-5.030563	12.675611	-4.591744
H	-6.036409	13.548469	-5.738486
C	-4.066220	15.713164	-4.411538
H	-3.415922	15.406071	-5.240175
H	-4.419549	16.722557	-4.656399
H	-3.439007	15.806733	-3.520470
N	8.101352	13.690809	0.080624
C	7.179559	14.215951	0.914502
H	6.858183	15.226980	0.707518
C	6.645710	13.524103	2.019834
H	5.922103	14.030785	2.644847
C	7.083571	12.207350	2.308543
C	8.053829	11.669781	1.427220
H	8.467350	10.679918	1.572009
C	8.520354	12.435307	0.341722
H	9.262759	12.019068	-0.324057
C	6.600049	11.476512	3.434937
C	5.348488	11.390195	5.325360
C	4.417810	12.006782	6.216628
C	3.901416	13.305947	5.976561
H	4.185624	13.880218	5.104405
C	3.016875	13.895286	6.899612
H	2.633124	14.889835	6.721790
C	3.077200	12.036997	8.266451
H	2.737258	11.553252	9.171224
C	3.969078	11.369426	7.401608
H	4.310022	10.379325	7.675034
C	6.676995	9.636043	4.759324
C	7.174327	8.322662	5.019759
C	6.842685	7.610124	6.200243
H	6.219368	8.048081	6.968494
C	8.030086	7.651731	4.109302
H	8.344579	8.110553	3.181153
C	8.484943	6.350100	4.396902
H	9.127779	5.837216	3.696205
C	7.348320	6.310560	6.404665
H	7.095989	5.767918	7.304038
C	3.457244	2.859039	-2.894287
H	4.383430	2.826856	-3.450289
C	3.492972	2.937020	-1.488704
H	4.458238	2.953407	-0.999408
C	1.156650	2.837470	-2.917006
H	0.243944	2.784149	-3.493501
C	1.090799	2.920408	-1.512649
H	0.116385	2.920804	-1.042047
C	2.284327	2.974053	-0.750584
C	2.270108	3.045837	0.674515
C	1.113542	3.139899	2.623425
C	3.382639	3.075328	2.651246
C	4.613056	3.082740	3.375189
C	4.653652	3.054557	4.791474
H	3.748064	2.988864	5.379973
C	5.869146	3.126033	2.719703
H	5.946794	3.135463	1.640606
C	7.056671	3.164953	3.475581
H	8.011615	3.204354	2.971521
C	5.892634	3.095234	5.461570
H	5.919769	3.071377	6.541479
C	-0.133024	3.242347	3.311691
C	-1.368759	3.272524	2.616820
H	-1.415741	3.222645	1.536828
C	-2.577646	3.340859	3.334045
H	-3.520023	3.349262	2.805094
C	-1.469356	3.384190	5.358015
H	-1.529462	3.436596	6.435546
C	-0.211394	3.312946	4.725732
H	0.678842	3.302366	5.341324
C	-3.827072	13.774108	0.619479
H	-3.628394	14.818431	0.425011
C	-4.782879	11.796833	-0.082087
H	-5.347117	11.266968	-0.836733
C	-4.367014	11.123826	1.083022
H	-4.635801	10.081795	1.197825
C	-3.647678	11.822013	2.085245
C	-3.374973	13.187433	1.817716
H	-2.844509	13.809876	2.526886
C	-3.254468	11.196280	3.306439
C	-3.311079	9.404772	4.693624
C	-2.330822	11.335021	5.374087

N	-4.483409	5.359238	5.519032
C	-3.700429	8.058550	4.964931
C	-4.387300	7.265589	4.012263
H	-4.661974	7.656537	3.041035
C	-4.753664	5.943241	4.332473
H	-5.292948	5.346357	3.610459
C	-3.426728	7.435869	6.208375
H	-2.927095	7.963667	7.010522
C	-3.829350	6.105424	6.432684
H	-3.628118	5.639908	7.386226
C	-1.638760	12.079593	6.375612
C	-1.299458	11.510501	7.627826
H	-1.574725	10.496178	7.882706
C	-0.581254	12.266699	8.572293
H	-0.324093	11.825101	9.523835
C	-0.518453	14.096646	7.182662
H	-0.215026	15.121945	7.027284
C	-1.228742	13.419739	6.171694
H	-1.453417	13.946038	5.253589
C	3.344114	5.983546	-4.629359
H	4.287965	5.505152	-4.849474
C	1.039365	5.878980	-4.665186
H	0.149906	5.317275	-4.913644
C	0.923617	7.162953	-4.096976
H	-0.067769	7.564932	-3.933855
C	3.325892	7.272068	-4.060199
H	4.272269	7.761348	-3.869455
C	2.089113	7.909223	-3.786811
C	2.020752	9.239188	-3.271354
C	0.792294	11.078871	-2.770913
C	3.056615	11.169868	-2.680886
C	-0.471260	11.741024	-2.720184
C	-1.682651	11.069445	-3.020048
H	-1.698796	10.022791	-3.294332
C	-2.901904	11.773763	-2.997250
H	-3.822083	11.261612	-3.241334
C	-1.852884	13.735069	-2.396285
H	-1.935698	14.786327	-2.159891
C	-0.587189	13.116833	-2.399050
H	0.280918	13.721671	-2.171203
C	4.253786	11.932794	-2.528163
C	4.228729	13.302947	-2.164736
H	3.299567	13.822872	-1.970784
C	5.431545	14.030691	-2.080170
H	5.405576	15.078101	-1.815781
C	5.536161	11.377562	-2.768808
H	5.661711	10.344870	-3.067466
C	6.685424	12.185763	-2.661146
H	7.660467	11.763973	-2.861868
C	11.157487	14.567881	-0.812315
H	10.761890	13.560592	-0.968420
H	12.106723	14.621905	-1.360021
H	11.411377	14.660158	0.251191
C	9.794756	16.490926	-0.145664
H	8.717505	16.441310	0.038206
H	10.299802	16.240820	0.795793
H	10.032941	17.541968	-0.351217
C	10.675584	16.346878	-2.433309
H	10.735480	17.418372	-2.203201
H	11.711148	16.064559	-2.662527
C	9.887302	16.226989	-3.761376
H	9.651618	17.252472	-4.073575
H	10.601638	15.875635	-4.516899
C	7.449822	16.194227	-4.045473
H	6.795107	16.216961	-3.170247
H	7.681269	17.238186	-4.291625
H	6.862824	15.803192	-4.886049
C	8.784431	14.252870	-4.717200
H	7.961543	14.216543	-5.441977
H	9.709839	14.288578	-5.305476
H	8.789580	13.300160	-4.179583
N	-4.455638	1.140172	5.839598
N	-6.356697	3.180828	6.689144
C	-4.306395	0.592319	4.464360
H	-3.350415	0.076055	4.311830
H	-5.089564	-0.129499	4.200300
H	-4.366546	1.398360	3.728683
C	-3.288253	0.776916	6.685885
H	-2.522388	0.212710	6.139042
H	-2.815276	1.682093	7.077076
H	-3.559550	0.164614	7.554998
C	-5.730003	0.671562	6.459285
H	-5.530433	0.065398	7.352279
H	-6.266328	-0.010979	5.787767
C	-6.749952	1.755216	6.887539
H	-7.687610	1.528656	6.363949
H	-6.979450	1.560167	7.943029
C	-7.346870	3.889797	5.834781

H	-7.742360	4.799177	6.304292
H	-6.883327	4.185638	4.889415
H	-8.218007	3.272887	5.580671
C	-6.188399	3.874063	7.993496
H	-6.801520	4.780029	8.079189
H	-6.447628	3.243997	8.853498
H	-5.143751	4.169824	8.124646
N	9.469907	1.711004	6.178892
N	10.585630	4.307954	6.903318
N	10.162755	15.578450	-1.261101
N	8.659122	15.379112	-3.753910
N	-6.751563	14.762118	-1.700734
N	-5.172268	14.752402	-4.156000
N	5.678243	12.084546	4.213902
N	5.799267	10.156558	5.650284
N	7.093821	10.233627	3.621643
N	8.158201	5.687456	5.524563
N	2.614511	13.282315	8.030143
N	2.314232	2.808281	-3.609877
N	3.465623	3.096033	1.301635
N	1.061146	3.105852	1.273061
N	2.238712	3.103290	3.373527
N	-2.635642	3.386852	4.679666
N	7.080770	3.150325	4.824092
N	-4.527761	13.100548	-0.316100
N	-3.584987	9.893758	3.463863
N	-2.660369	10.058839	5.677465
N	-2.580889	11.956535	4.198275
N	-0.186101	13.539789	8.366815
N	2.225213	5.297353	-4.938288
N	0.788426	9.768811	-3.103344
N	1.887108	11.818936	-2.486482
N	3.193242	9.868605	-3.025029
N	6.643361	13.492779	-2.329554
N	-2.996623	13.085048	-2.695333
Pd	1.436289	14.301488	9.449691
Pd	-4.854632	13.927594	-2.216300
Pd	8.821759	3.718948	5.859070
Pd	8.398424	14.524850	-1.818954
Pd	2.327110	3.322150	-5.644589
Pd	-4.481237	3.270424	5.680087
C	2.622454	5.696078	5.817394
C	2.960526	6.178340	4.511304
C	1.867305	6.403936	3.583074
C	0.646511	6.827741	4.149303
C	0.634842	7.001679	5.587426
C	1.445802	6.121874	6.370973
H	2.968153	6.059995	1.772784
H	3.356119	5.163271	6.412121
H	3.920121	5.914976	4.077583
C	1.997269	6.319197	2.186069
C	-0.433613	7.108273	3.296180
H	-0.247341	7.450058	6.037031
H	1.212569	5.941144	7.414375
C	-0.329775	6.958658	1.919309
C	0.915218	6.564239	1.349562
H	-1.370350	7.447817	3.725493
C	-1.500913	7.284097	1.029019
H	-1.253613	8.103645	0.343625
H	-2.373065	7.591360	1.610906
C	1.063344	6.454551	-0.146493
H	0.314445	5.779726	-0.574804
H	2.058072	6.093402	-0.416173
C	3.296358	8.923788	3.744576
C	3.263332	8.181550	5.043853
C	2.124724	8.645821	5.745451
C	1.502436	9.713904	4.933881
H	4.205093	7.953926	5.518110
H	2.041051	8.703202	6.819775
C	1.875144	10.670379	2.593415
C	0.491163	10.326502	2.026126
C	1.991417	12.153125	2.978431
H	2.617512	10.458506	1.821836
C	0.159683	11.229364	0.833752
H	-0.256578	10.445215	2.817657
H	0.491253	9.280568	1.722350
C	1.659640	13.036283	1.766058
H	1.300195	12.367041	3.800157
H	3.007231	12.357567	3.333942
C	0.266732	12.711830	1.210269
H	-0.841874	10.990531	0.453666
H	0.873099	10.999987	0.032608
H	1.724449	14.092290	2.039664
H	2.407963	12.866560	0.978422
H	0.050054	13.351915	0.349201
H	-0.483240	12.947160	1.977474
N	2.218866	9.783999	3.717337
O	0.563352	10.433693	5.215297

O	4.119027	8.809737	2.830078
O	5.453135	6.326281	2.624134
H	5.722763	6.191927	1.707883
H	5.128159	7.245365	2.656130
O	2.980429	8.933494	0.078617
H	3.392964	8.763606	0.942212
H	3.720213	8.952871	-0.540947
H	-1.793685	6.434888	0.403915
H	0.942094	7.435723	-0.615476

Table5_1b_TSiii_DG_h2o_1wat

Property	Value
Charge	0
Electronic Energy, BS1 (a.u.)	-1211.819829
Thermal and entropic correction, BS1 (a.u.)	2.804012
Electronic Energy, BS2 (a.u.)	-1212.243711
Number of Imaginary Frequencies	0
Imaginary frequencies (cm-1)	None

Molecular Geometry in Cartesian Coordinates

C	8.411921	1.027625	6.966465
H	8.782614	0.593506	7.903488
H	7.629942	1.741877	7.239207
H	7.931910	0.208300	6.416770
C	9.628227	1.081205	4.840096
H	8.923933	0.257025	4.670629
H	9.458852	1.823092	4.054658
H	10.630737	0.668250	4.672113
C	10.766929	1.718749	6.919019
H	11.545893	1.194966	6.350313
H	10.686914	1.145978	7.851830
C	11.358327	3.097219	7.302288
H	12.373418	3.125233	6.885875
H	11.516051	3.074403	8.388164
C	11.378314	5.163861	5.979202
H	10.904329	5.190775	4.994189
H	11.470951	6.199878	6.328377
H	12.401554	4.799119	5.825238
C	10.151578	5.080455	8.098111
H	9.061527	5.060305	8.179452
H	10.544817	4.675680	9.039075
H	10.460425	6.132671	8.063210
N	3.102113	15.071578	10.543915
N	0.213656	15.331234	10.864523
C	3.867517	13.904846	11.057080
H	3.332720	12.978997	10.828603
H	4.870221	13.825045	10.618857
H	4.008378	13.925436	12.145024
C	3.921952	15.869982	9.594062
H	3.413729	15.942117	8.628871
H	4.096369	16.898617	9.933860
H	4.912652	15.434290	9.414067
C	2.611796	15.922598	11.667640
H	2.993952	15.558134	12.629811
H	3.011138	16.941981	11.590818
C	1.078902	16.065260	11.833651
H	0.862943	17.141228	11.814927
H	0.850878	15.765042	12.864471
C	-0.642497	16.274133	10.096013
H	-1.710552	16.031518	10.161303
H	-0.543438	17.315390	10.427308
H	-0.366514	16.253645	9.037685
C	-0.612886	14.306946	11.556901
H	-0.319354	13.305805	11.228366
H	-0.499343	14.326586	12.647927
H	-1.686267	14.417055	11.357167
N	2.416789	1.301875	-6.324937
N	2.329560	3.872458	-7.705287
C	3.667258	0.709156	-5.780186
H	3.479501	-0.102847	-5.066510
H	4.320752	0.291591	-6.556322
H	4.248595	1.475525	-5.259780
C	1.218951	0.606333	-5.783732
H	1.472155	-0.195519	-5.078851
H	0.581282	1.319421	-5.253354
H	0.598277	0.146086	-6.562669
C	2.420494	1.278297	-7.817069
H	1.572258	0.697597	-8.201613
H	3.302269	0.746331	-8.196702
C	2.386415	2.642748	-8.549476
H	3.262249	2.661926	-9.210635
H	1.532927	2.601748	-9.238327

C	1.076978	4.632585	-7.959276
H	1.260198	5.680156	-8.229040
H	0.471158	4.208471	-8.769774
H	0.449271	4.628479	-7.064095
C	3.525425	4.727479	-7.929359
H	3.268599	5.754120	-8.218851
H	4.121583	4.783482	-7.014287
H	4.189188	4.344002	-8.714305
C	-6.518695	15.734352	-0.599248
H	-6.994077	15.434317	0.343132
H	-5.446569	15.833149	-0.409255
H	-6.892119	16.741081	-0.825414
C	-7.615228	13.641082	-1.246609
H	-7.904095	13.724525	-0.191455
H	-8.550321	13.559630	-1.814746
H	-7.090218	12.689618	-1.368267
C	-7.337330	15.438600	-2.894593
H	-7.565656	16.489384	-2.674781
H	-8.310522	15.002227	-3.153507
C	-6.496524	15.438771	-4.195306
H	-7.136482	15.010837	-4.977578
H	-6.382628	16.490078	-4.489450
C	-5.129349	13.624415	-5.125743
H	-4.289205	13.695525	-5.827753
H	-5.030104	12.675098	-4.592057
H	-6.036026	13.547879	-5.738785
C	-4.065753	15.712684	-4.412200
H	-3.415367	15.405402	-5.240700
H	-4.419124	16.721996	-4.657337
H	-3.438648	15.806474	-3.521065
N	8.101363	13.690459	0.080530
C	7.179647	14.215798	0.914394
H	6.858267	15.226794	0.707236
C	6.645850	13.524184	2.019913
H	5.922315	14.030982	2.644913
C	7.083571	12.207421	2.308757
C	8.053714	11.669635	1.427433
H	8.467168	10.679757	1.572298
C	8.520279	12.434970	0.341826
H	9.262617	12.018545	-0.323909
C	6.600000	11.476730	3.435228
C	5.348650	11.390537	5.325799
C	4.417916	12.007070	6.217080
C	3.901766	13.306377	5.977251
H	4.186405	13.880915	5.105407
C	3.016754	13.895450	6.900017
H	2.633083	14.890041	6.722233
C	3.076494	12.036848	8.266421
H	2.736203	11.552917	9.170967
C	3.968747	11.369472	7.401777
H	4.309713	10.379372	7.675190
C	6.676134	9.635833	4.759090
C	7.172923	8.322133	5.018986
C	6.841582	7.609528	6.199500
H	6.218735	8.047561	6.968040
C	8.028006	7.651014	4.108019
H	8.342207	8.109834	3.179770
C	8.482777	6.349306	4.395365
H	9.125172	5.836300	3.694346
C	7.347104	6.309875	6.403676
H	7.095014	5.767248	7.303129
C	3.458120	2.857531	-2.895365
H	4.384297	2.825879	-3.451422
C	3.493873	2.935327	-1.489763
H	4.459144	2.952177	-1.000483
C	1.157552	2.834797	-2.917995
H	0.244844	2.781090	-3.494443
C	1.091713	2.917483	-1.513634
H	0.117325	2.917208	-1.043001
C	2.285224	2.971693	-0.751601
C	2.270963	3.043506	0.673498
C	1.114407	3.138867	2.622373
C	3.383485	3.073666	2.650193
C	4.613873	3.080896	3.374154
C	4.654422	3.051930	4.790416
H	3.748837	2.980324	5.378808
C	5.869956	3.124828	2.718714
H	5.947595	3.134963	1.639623
C	7.057441	3.163827	3.474648
H	8.012386	3.203905	2.970628
C	5.893362	3.092695	5.460561
H	5.920465	3.068308	6.540460
C	-0.132057	3.243246	3.310544
C	-1.367785	3.273827	2.615709
H	-1.414831	3.222717	1.535777
C	-2.576566	3.344489	3.332914
H	-3.518933	3.353448	2.803954
C	-1.468185	3.389205	5.356716

H	-1.528151	3.443253	6.434172
C	-0.210323	3.315856	4.724471
H	0.679962	3.305487	5.339974
C	-3.826006	13.774236	0.618920
H	-3.626527	14.818300	0.423889
C	-4.782889	11.797183	-0.081888
H	-5.347173	11.267275	-0.836469
C	-4.367933	11.124573	1.083768
H	-4.637499	10.082795	1.199040
C	-3.648601	11.822888	2.085921
C	-3.374748	13.187931	1.817659
H	-2.844014	13.810345	2.526630
C	-3.256554	11.197665	3.307752
C	-3.314787	9.406747	4.695664
C	-2.334027	11.336839	5.375869
N	-4.485769	5.360527	5.519946
C	-3.703886	8.060412	4.966744
C	-4.390302	7.267420	4.013790
H	-4.664995	7.658499	3.042629
C	-4.756133	5.944835	4.333573
H	-5.295101	5.347953	3.611335
C	-3.430269	7.437574	6.210111
H	-2.931015	7.965395	7.012480
C	-3.832354	6.106874	6.433946
H	-3.631227	5.641244	7.387448
C	-1.641318	12.081202	6.377128
C	-1.302420	11.512262	7.629522
H	-1.578768	10.498335	7.884832
C	-0.583243	12.268075	8.573576
H	-0.326393	11.826585	9.525251
C	-0.518847	14.097452	7.183259
H	-0.214443	15.122405	7.027502
C	-1.230005	13.420873	6.172666
H	-1.454314	13.947042	5.254395
C	3.344370	5.982231	-4.628996
H	4.288251	5.503869	-4.849071
C	1.039617	5.877602	-4.665032
H	0.150167	5.315951	-4.913648
C	0.923796	7.161496	-4.096639
H	-0.067614	7.563402	-3.933486
C	3.326066	7.270695	-4.059709
H	4.272421	7.759971	-3.868840
C	2.089251	7.907778	-3.786332
C	2.020854	9.237731	-3.270844
C	0.792440	11.077670	-2.771279
C	3.056771	11.168540	-2.680894
C	-0.471032	11.739967	-2.720689
C	-1.682544	11.068438	-3.020152
H	-1.698868	10.021680	-3.294039
C	-2.901700	11.772945	-2.997430
H	-3.821999	11.260845	-3.241174
C	-1.852327	13.734314	-2.397291
H	-1.934976	14.785652	-2.161208
C	-0.586727	13.115908	-2.400032
H	0.281495	13.720720	-2.172543
C	4.253916	11.931525	-2.528278
C	4.228794	13.301886	-2.165623
H	3.299605	13.821967	-1.972227
C	5.431597	14.029640	-2.081032
H	5.405548	15.077159	-1.817081
C	5.536339	11.376130	-2.768219
H	5.661936	10.343242	-3.066184
C	6.685588	12.184377	-2.660672
H	7.660674	11.762427	-2.860838
C	11.157417	14.567269	-0.812620
H	10.761786	13.559943	-0.968336
H	12.106616	14.621020	-1.360414
H	11.411402	14.659924	0.250833
C	9.794712	16.490550	-0.146580
H	8.717428	16.441118	0.037134
H	10.299581	16.240615	0.795016
H	10.033089	17.541503	-0.352373
C	10.675625	16.345814	-2.434135
H	10.735401	17.417388	-2.204379
H	11.711246	16.063528	-2.663132
C	9.887521	16.225415	-3.762254
H	9.652212	17.250804	-4.075044
H	10.601819	15.873358	-4.517486
C	7.449989	16.193385	-4.045924
H	6.795401	16.216412	-3.170611
H	7.681697	17.237255	-4.292199
H	6.862768	15.802462	-4.886394
C	8.783909	14.251521	-4.717572
H	7.960544	14.214920	-5.441798
H	9.708937	14.287285	-5.306453
H	8.789519	13.298932	-4.179743
N	-4.450497	1.141292	5.837438
N	-6.355154	3.178006	6.688240

C	-4.300073	0.594479	4.461928
H	-3.342631	0.081083	4.308891
H	-5.081236	-0.129483	4.197783
H	-4.362831	1.400670	3.726640
C	-3.282608	0.779678	6.683734
H	-2.515849	0.216740	6.136838
H	-2.811063	1.685483	7.075195
H	-3.553040	0.166786	7.552704
C	-5.724115	0.669990	6.456615
H	-5.523591	0.063364	7.349080
H	-6.259280	-0.012833	5.784456
C	-6.745891	1.751575	6.885728
H	-7.683224	1.523751	6.362107
H	-6.974922	1.555398	7.941109
C	-7.346581	3.885818	5.834368
H	-7.743547	4.794268	6.304447
H	-6.883572	4.183016	4.889157
H	-8.216696	3.267597	5.579937
C	-6.188001	3.870679	7.993035
H	-6.802712	4.775505	8.079359
H	-6.446043	3.239596	8.852648
H	-5.143882	4.168217	8.124301
N	9.471675	1.711510	6.178329
N	10.584994	4.309472	6.902743
N	10.162715	15.577736	-1.261727
N	8.659085	15.377932	-3.754435
N	-6.750371	14.762707	-1.700827
N	-5.171770	14.751955	-4.156481
N	5.678603	12.085039	4.214482
N	5.798812	10.156573	5.650366
N	7.093328	10.233647	3.621670
N	8.156553	5.686718	5.523209
N	2.613827	13.282203	8.030202
N	2.315101	2.806323	-3.610932
N	3.466469	3.094149	1.300586
N	1.061986	3.103418	1.272043
N	2.239586	3.107221	3.372492
N	-2.634551	3.392238	4.678492
N	7.081502	3.148614	4.823157
N	-4.526808	13.100594	-0.316510
N	-3.587994	9.895458	3.465651
N	-2.664535	10.060994	5.679696
N	-2.583102	11.957981	4.199636
N	-0.186830	13.540688	8.367546
N	2.225515	5.296036	-4.938096
N	0.788526	9.767495	-3.103266
N	1.887284	11.817818	-2.487195
N	3.193342	9.867029	-3.024125
N	6.643473	13.491581	-2.329789
N	-2.996191	13.084350	-2.695948
Pd	1.436202	14.301541	9.450245
Pd	-4.853946	13.927331	-2.216788
Pd	8.821780	3.718845	5.858281
Pd	8.398379	14.523994	-1.819310
Pd	2.327599	3.321164	-5.645365
Pd	-4.479928	3.271635	5.679133
C	2.605255	5.697744	5.839192
C	3.006993	6.382735	4.553065
C	1.858425	6.419513	3.573972
C	0.650436	6.844390	4.140509
C	0.750501	7.172855	5.599779
C	1.462071	6.104086	6.377522
H	2.961388	6.053422	1.778557
H	3.304161	5.050360	6.376046
H	3.930887	6.016423	4.104744
C	1.991646	6.305955	2.192472
C	-0.434960	7.105299	3.304012
H	-0.166589	7.561970	6.044019
H	1.144491	5.824977	7.384347
C	-0.333513	6.956963	1.916799
C	0.899161	6.558065	1.351475
H	-1.364748	7.464824	3.733590
C	-1.504901	7.289722	1.032613
H	-1.256494	8.108503	0.346653
H	-2.375737	7.599223	1.615293
C	1.056059	6.451393	-0.144600
H	0.304563	5.778978	-0.577083
H	2.049283	6.086124	-0.414387
C	3.290525	8.899276	3.745195
C	3.213964	8.000689	4.982536
C	1.977480	8.510428	5.715974
C	1.486536	9.698577	4.938463
H	4.179235	8.110370	5.578408
H	2.080844	8.749109	6.795515
C	1.882135	10.671648	2.600920
C	0.500105	10.322054	2.029302
C	1.991664	12.154332	2.986621
H	2.627035	10.455016	1.836715

C	0.171504	11.230316	0.836457
H	-0.250231	10.444761	2.818515
H	0.496999	9.276335	1.724760
C	1.662925	13.037586	1.768509
H	1.298021	12.367775	3.801440
H	3.006392	12.364112	3.339963
C	0.271680	12.712673	1.208848
H	-0.828889	10.986060	0.453586
H	0.882262	11.001577	0.037244
H	1.726624	14.093572	2.042456
H	2.413485	12.868249	0.982922
H	0.057002	13.347999	0.347453
H	-0.480439	12.947371	1.974170
N	2.227701	9.780116	3.735871
O	0.561713	10.437982	5.212830
O	4.119547	8.816486	2.836758
O	5.446704	6.324049	2.624030
H	5.718107	6.193262	1.707803
H	5.128573	7.243658	2.654531
O	2.977860	8.930136	0.083140
H	3.395831	8.755459	0.941535
H	3.717303	8.949414	-0.541845
H	-1.805361	6.441055	0.408037
H	0.938292	7.432624	-0.614388

Table5_1c_reactant_0wat

Property	Value
Charge	0
Electronic Energy, BS1 (a.u.)	-1137.568721
Thermal and entropic correction, BS1 (a.u.)	2.805765
Electronic Energy, BS2 (a.u.)	-1137.946814
Number of Imaginary Frequencies	0
Imaginary frequencies (cm-1)	None

Molecular Geometry in Cartesian Coordinates

C	8.368714	1.015229	6.897359
H	8.748009	0.545438	7.813499
H	7.622549	1.751468	7.208663
H	7.843606	0.227325	6.343003
C	9.533721	1.068866	4.742346
H	8.796335	0.273814	4.574958
H	9.372864	1.830838	3.974424
H	10.516149	0.623053	4.542582
C	10.744431	1.624638	6.803669
H	11.496746	1.104116	6.197342
H	10.671397	1.018858	7.716038
C	11.377810	2.972604	7.226079
H	12.386821	2.989110	6.794431
H	11.551461	2.906798	8.307804
C	11.438680	5.091108	5.990512
H	10.958948	5.171737	5.011430
H	11.559982	6.108567	6.382741
H	12.451574	4.708397	5.813167
C	10.222300	4.949192	8.112116
H	9.131980	4.958672	8.194395
H	10.604430	4.492228	9.033502
H	10.562460	5.992309	8.122199
N	3.122207	14.979599	10.424163
N	0.249009	15.272925	10.840719
C	3.877471	13.804074	10.932069
H	3.316028	12.887228	10.733147
H	4.864855	13.696207	10.466087
H	4.050360	13.836985	12.015063
C	3.932581	15.750125	9.443417
H	3.397300	15.830281	8.493797
H	4.144279	16.775985	9.770407
H	4.905866	15.287694	9.236694
C	2.679497	15.854424	11.549311
H	3.092897	15.502891	12.503330
H	3.083607	16.869026	11.440718
C	1.154715	16.011663	11.768431
H	0.946423	17.089030	11.744734
H	0.961983	15.725168	12.810286
C	-0.635859	16.212790	10.101527
H	-1.700911	15.972956	10.211575
H	-0.521459	17.255875	10.422040
H	-0.403593	16.184731	9.033047
C	-0.551184	14.255590	11.572875
H	-0.274504	13.251835	11.237797
H	-0.392532	14.281124	12.658164
H	-1.631544	14.367548	11.416601
N	2.404860	1.242748	-6.098408

N	2.406485	3.592564	-7.828440
C	3.615406	0.701491	-5.425559
H	3.376955	0.006477	-4.610692
H	4.282130	0.158839	-6.107339
H	4.202213	1.519940	-4.999006
C	1.166763	0.661248	-5.515322
H	1.367666	-0.027538	-4.685250
H	0.524378	1.459331	-5.132670
H	0.569461	0.098774	-6.243775
C	2.463082	1.008091	-7.570984
H	1.621801	0.385230	-7.901013
H	3.351181	0.421597	-7.839100
C	2.469320	2.255553	-8.488944
H	3.363290	2.171986	-9.120188
H	1.634552	2.126882	-9.189817
C	1.167616	4.319022	-8.214674
H	1.367344	5.317875	-8.622473
H	0.576198	3.790680	-8.972885
H	0.519910	4.443708	-7.342344
C	3.615729	4.398550	-8.144235
H	3.375654	5.379699	-8.572827
H	4.197587	4.570787	-7.234308
H	4.287490	3.907392	-8.859310
C	-6.548345	15.739448	-0.639228
H	-7.076041	15.434369	0.273103
H	-5.486517	15.822246	-0.392925
H	-6.897144	16.753657	-0.871077
C	-7.636530	13.669397	-1.369311
H	-7.976724	13.742447	-0.328661
H	-8.543368	13.610073	-1.983981
H	-7.118193	12.712530	-1.478550
C	-7.248218	15.483141	-2.977734
H	-7.479599	16.532668	-2.754814
H	-8.209273	15.059802	-3.296727
C	-6.336093	15.495416	-4.229604
H	-6.942261	15.102322	-5.055820
H	-6.180969	16.550078	-4.490844
C	-4.976470	13.653964	-5.114430
H	-4.095500	13.699926	-5.766826
H	-4.945272	12.697324	-4.586280
H	-5.848580	13.616571	-5.779274
C	-3.891719	15.706415	-4.332216
H	-3.211164	15.387776	-5.131762
H	-4.206716	16.726265	-4.586158
H	-3.305364	15.777969	-3.411684
N	8.066385	13.615033	-0.025108
C	7.178472	14.166312	0.828227
H	6.859952	15.176889	0.615809
C	6.672761	13.495879	1.959696
H	5.975034	14.018331	2.600757
C	7.101869	12.176677	2.251088
C	8.029986	11.609915	1.341385
H	8.418160	10.608249	1.475606
C	8.473791	12.355280	0.232133
H	9.181610	11.918875	-0.457923
C	6.637397	11.464101	3.397453
C	5.397293	11.387509	5.295795
C	4.430708	11.980729	6.165294
C	3.870458	13.257397	5.905310
H	4.152705	13.834977	5.034463
C	2.937059	13.814071	6.801235
H	2.516066	14.789843	6.604817
C	3.036396	11.968878	8.179517
H	2.690301	11.474772	9.076192
C	3.976912	11.333825	7.342895
H	4.346269	10.357989	7.629175
C	6.730667	9.637062	4.738106
C	7.229585	8.324823	5.002466
C	6.917628	7.623021	6.194685
H	6.300797	8.065837	6.965293
C	8.076316	7.648627	4.087823
H	8.374087	8.100125	3.149860
C	8.556178	6.359755	4.390741
H	9.203788	5.849600	3.691711
C	7.446068	6.334609	6.413319
H	7.216523	5.805421	7.326936
C	3.462519	3.176355	-2.897953
H	4.390527	3.205914	-3.451298
C	3.485708	3.187608	-1.490048
H	4.447935	3.218876	-0.994837
C	1.157441	3.085902	-2.938532
H	0.251253	3.041772	-3.525512
C	1.082627	3.094279	-1.530833
H	0.106746	3.047992	-1.065084
C	2.271210	3.141877	-0.758966
C	2.250421	3.140478	0.669047
C	1.099937	3.115050	2.626247
C	3.368359	3.162239	2.643861

C	4.604262	3.184590	3.358443
C	4.659158	3.102393	4.772045
H	3.759845	2.996625	5.365237
C	5.852872	3.282528	2.694119
H	5.921272	3.336281	1.614949
C	7.047867	3.303093	3.438661
H	7.997412	3.374108	2.926949
C	5.904340	3.133625	5.431859
H	5.942569	3.065478	6.509464
C	-0.144339	3.122123	3.326556
C	-1.383015	3.014716	2.646211
H	-1.432487	2.912942	1.569945
C	-2.588924	3.014544	3.372597
H	-3.531277	2.930720	2.849965
C	-1.472796	3.227750	5.381554
H	-1.529470	3.310871	6.457364
C	-0.217481	3.231328	4.738944
H	0.675156	3.317224	5.345301
C	-3.929836	13.757189	0.685541
H	-3.709017	14.798434	0.499680
C	-4.881678	11.792377	-0.053780
H	-5.422435	11.269522	-0.830329
C	-4.519964	11.113851	1.127765
H	-4.803443	10.074889	1.234442
C	-3.826938	11.802089	2.154809
C	-3.531453	13.165207	1.898744
H	-3.018698	13.783286	2.624356
C	-3.465473	11.171255	3.383485
C	-3.486863	9.365690	4.755552
C	-2.541058	11.309054	5.451291
N	-4.373617	5.234470	5.494674
C	-3.792918	7.991900	5.000113
C	-4.432498	7.174232	4.034076
H	-4.738941	7.568134	3.073405
C	-4.698586	5.819647	4.322722
H	-5.194425	5.199320	3.589045
C	-3.459681	7.359695	6.225362
H	-2.981503	7.900281	7.032338
C	-3.761305	5.998466	6.421542
H	-3.508932	5.522971	7.357818
C	-1.813799	12.047984	6.432064
C	-1.446403	11.479870	7.676626
H	-1.732455	10.471355	7.943435
C	-0.676688	12.225266	8.589479
H	-0.395128	11.782851	9.533766
C	-0.622012	14.045124	7.185226
H	-0.300322	15.061860	7.011286
C	-1.382891	13.378264	6.205612
H	-1.624880	13.904010	5.291603
C	3.399175	6.090930	-5.018295
H	4.341559	5.589421	-5.187658
C	1.095986	6.014623	-5.098098
H	0.203500	5.453060	-5.334652
C	0.986078	7.329724	-4.605999
H	-0.002991	7.752090	-4.485813
C	3.387922	7.411331	-4.526350
H	4.335922	7.896723	-4.331149
C	2.154540	8.074107	-4.304311
C	2.090757	9.410874	-3.806067
C	0.864167	11.192381	-3.114531
C	3.130324	11.301303	-3.101653
C	-0.406280	11.810639	-2.909363
C	-1.619422	11.143628	-3.215122
H	-1.630768	10.137059	-3.612606
C	-2.850521	11.801571	-3.032822
H	-3.773267	11.292698	-3.273101
C	-1.807476	13.712299	-2.265160
H	-1.901970	14.724469	-1.897847
C	-0.530879	13.133137	-2.412288
H	0.337706	13.727264	-2.158971
C	4.329962	12.047830	-2.899428
C	4.310972	13.392555	-2.451227
H	3.382608	13.910924	-2.250255
C	5.520343	14.094501	-2.281774
H	5.501977	15.123085	-1.951112
C	5.611402	11.491302	-3.141615
H	5.732177	10.472959	-3.488613
C	6.767428	12.269552	-2.936480
H	7.742076	11.840978	-3.122134
C	11.176630	14.461464	-0.713401
H	10.768314	13.469441	-0.925696
H	12.156896	14.509785	-1.203961
H	11.370332	14.513852	0.365280
C	9.822945	16.397004	-0.062913
H	8.735403	16.375839	0.051318
H	10.260075	16.100367	0.898724
H	10.103014	17.446435	-0.217706
C	10.830377	16.301637	-2.299701

H	10.919153	17.360248	-2.023888
H	11.864448	15.988183	-2.492242
C	10.101329	16.266142	-3.665662
H	9.903463	17.310965	-3.937330
H	10.842050	15.933795	-4.404220
C	7.679893	16.304773	-4.066556
H	6.981057	16.297796	-3.225496
H	7.944762	17.354035	-4.247480
H	7.129325	15.969164	-4.954251
C	9.003274	14.364845	-4.762920
H	8.212466	14.377422	-5.523403
H	9.953926	14.408865	-5.308794
H	8.968460	13.388943	-4.270194
N	-4.569948	1.027794	5.976711
N	-6.357322	3.198659	6.749663
C	-4.493789	0.415472	4.623093
H	-3.574276	-0.162148	4.466341
H	-5.324936	-0.268662	4.410882
H	-4.528780	1.193409	3.856673
C	-3.396318	0.638025	6.802579
H	-2.688146	-0.003280	6.263133
H	-2.852330	1.531300	7.123321
H	-3.671819	0.092810	7.714057
C	-5.846924	0.655386	6.653654
H	-5.651561	0.102732	7.581630
H	-6.424722	-0.048305	6.040854
C	-6.815293	1.805348	7.023751
H	-7.764576	1.594414	6.514762
H	-7.048794	1.676450	8.088459
C	-7.318318	3.908428	5.863651
H	-7.672917	4.855092	6.289969
H	-6.845729	4.138650	4.904695
H	-8.215938	3.318217	5.640234
C	-6.150537	3.948119	8.016911
H	-6.707785	4.892634	8.051384
H	-6.450435	3.381126	8.907060
H	-5.090507	4.185311	8.140372
N	9.431986	1.677533	6.095918
N	10.630525	4.218168	6.883394
N	10.232902	15.510187	-1.184346
N	8.855691	15.449074	-3.755396
N	-6.734379	14.784190	-1.763568
N	-5.033012	14.773274	-4.136607
N	5.728212	12.083327	4.185073
N	5.873880	10.169371	5.641789
N	7.148202	10.230705	3.599461
N	8.256286	5.710127	5.533920
N	2.528407	13.192550	7.925798
N	2.323240	3.120433	-3.616736
N	3.443584	3.220235	1.296574
N	1.041940	3.108226	1.273673
N	2.229115	3.139254	3.371457
N	-2.640840	3.115409	4.715536
N	7.084882	3.228793	4.784780
N	-4.600571	13.093251	-0.277387
N	-3.783864	9.863744	3.532521
N	-2.872238	10.033815	5.754411
N	-2.799440	11.931551	4.279619
N	-0.259591	13.487814	8.359882
N	2.277436	5.398859	-5.307051
N	0.860278	9.925763	-3.585887
N	1.962260	11.917977	-2.806914
N	3.262252	10.034795	-3.555641
N	6.732288	13.552348	-2.520426
N	-2.950071	13.065636	-2.575622
Pd	1.410577	14.229886	9.385172
Pd	-4.824822	13.926555	-2.189312
Pd	8.846677	3.713182	5.828867
Pd	8.479682	14.517266	-1.875379
Pd	2.356482	3.340728	-5.711792
Pd	-4.484557	3.147288	5.727775
C	2.862167	5.709615	5.935148
C	3.053446	6.146404	4.621978
C	1.919779	6.398525	3.792486
C	0.686800	6.684047	4.439692
C	0.680867	6.701844	5.866690
C	1.656936	5.998283	6.575106
H	2.917497	6.207703	1.890586
H	3.694415	5.304370	6.508434
H	4.031236	6.072081	4.147149
C	1.963830	6.390889	2.381985
C	-0.448494	6.942276	3.645590
H	-0.200120	7.077654	6.378390
H	1.544750	5.815174	7.638658
C	-0.410123	6.883169	2.263291
C	0.829142	6.589222	1.614391
H	-1.381541	7.189990	4.143809
C	-1.674647	7.169988	1.476768

C	-2.507968	5.917564	1.172932
H	-1.430096	7.672004	0.538602
H	-2.286479	7.882647	2.044310
H	-3.393675	6.160611	0.581594
H	-1.926187	5.180251	0.612730
H	-2.834877	5.448104	2.106178
C	0.949611	6.564189	0.105422
C	1.191191	7.959223	-0.495341
H	0.060580	6.115949	-0.345032
H	1.795718	5.925985	-0.168784
H	1.326450	7.881161	-1.576487
H	0.348641	8.630754	-0.311514
H	2.088070	8.413828	-0.065006
C	3.212172	9.022290	3.979684
C	3.227502	8.377181	5.326713
C	2.089477	8.703725	5.974077
C	1.294092	9.595025	5.096526
H	4.169937	8.039881	5.758464
H	1.874762	8.651978	7.040030
C	1.625395	10.546588	2.755004
C	0.188497	10.303977	2.278056
C	1.866963	12.028011	3.093060
H	2.317527	10.255139	1.955661
C	-0.155656	11.210499	1.091904
H	-0.493753	10.505888	3.108496
H	0.074955	9.253715	2.014200
C	1.498467	12.932033	1.910216
H	1.254573	12.284192	3.969457
H	2.918303	12.164576	3.369833
C	0.056520	12.687528	1.446006
H	-1.189530	11.028100	0.770923
H	0.484542	10.950357	0.236714
H	1.639763	13.982607	2.178319
H	2.181501	12.726224	1.074709
H	-0.178221	13.331516	0.591369
H	-0.630812	12.968337	2.255344
N	1.983385	9.684392	3.882917
O	0.271896	10.199795	5.373856
O	4.087041	9.033128	3.131733

Table5_1c_TSi_DDG_0wat

Property	Value
Charge	0
Electronic Energy, BS1 (a.u.)	-1137.564849
Thermal and entropic correction, BS1 (a.u.)	2.808228
Electronic Energy, BS2 (a.u.)	-1137.944380
Number of Imaginary Frequencies	0
Imaginary frequencies (cm-1)	None

Molecular Geometry in Cartesian Coordinates

C	8.239713	0.953243	6.678501
H	8.580645	0.408378	7.567821
H	7.532578	1.710714	7.026358
H	7.677684	0.231177	6.073102
C	9.436215	1.063023	4.543092
H	8.664292	0.314424	4.325134
H	9.320803	1.874369	3.819021
H	10.398970	0.583338	4.326086
C	10.645143	1.436631	6.645817
H	11.364869	0.880618	6.031558
H	10.521141	0.813334	7.540682
C	11.368966	2.724829	7.107114
H	12.374728	2.688518	6.669153
H	11.543317	2.611881	8.184798
C	11.558613	4.869053	5.927967
H	11.071318	5.008114	4.958711
H	11.752670	5.865469	6.344370
H	12.541295	4.424751	5.726185
C	10.368012	4.750588	8.064691
H	9.283031	4.837577	8.164663
H	10.727590	4.240252	8.966979
H	10.785081	5.764831	8.099630
N	3.147906	15.078228	10.603120
N	0.247492	15.183367	10.905385
C	3.953883	13.962780	11.165949
H	3.462802	13.007763	10.961013
H	4.966430	13.912767	10.746355
H	4.075171	14.024953	12.254658
C	3.948375	15.880911	9.640147
H	3.451323	15.904620	8.666601
H	4.076697	16.925338	9.951165
H	4.958266	15.480836	9.486262

C	2.603842	15.943413	11.690675
H	3.004957	15.641444	12.666696
H	2.940835	16.981346	11.573351
C	1.064814	15.997272	11.852125
H	0.783924	17.057095	11.801843
H	0.853490	15.714094	12.891286
C	-0.675392	16.048499	10.122518
H	-1.722766	15.728920	10.192457
H	-0.654009	17.099166	10.438149
H	-0.398770	16.032892	9.064697
C	-0.503219	14.121082	11.625566
H	-0.151357	13.135449	11.307364
H	-0.375879	14.165509	12.714388
H	-1.583966	14.160777	11.440402
N	2.371175	1.232302	-6.124762
N	2.342979	3.566625	-7.874384
C	3.595334	0.707966	-5.463557
H	3.373926	0.020906	-4.637246
H	4.255908	0.162360	-6.148887
H	4.181894	1.535802	-5.055224
C	1.146299	0.645062	-5.519718
H	1.364500	-0.032343	-4.684650
H	0.500707	1.440960	-5.137538
H	0.545772	0.068460	-6.234293
C	2.411505	0.984923	-7.595819
H	1.567087	0.358308	-7.910398
H	3.297323	0.397490	-7.869405
C	2.405903	2.224521	-8.524282
H	3.295635	2.139222	-9.161306
H	1.566434	2.086837	-9.217763
C	1.099217	4.285821	-8.258315
H	1.293113	5.280973	-8.677673
H	0.503168	3.748181	-9.006261
H	0.458320	4.417912	-7.382110
C	3.547282	4.374099	-8.204534
H	3.300953	5.353785	-8.632967
H	4.137257	4.550071	-7.300591
H	4.213117	3.882119	-8.924512
C	-6.495306	15.528100	-0.212083
H	-6.904862	15.176512	0.743207
H	-5.414431	15.635894	-0.086801
H	-6.895891	16.538141	-0.365416
C	-7.604422	13.449719	-0.887882
H	-7.811715	13.469708	0.189309
H	-8.579828	13.390254	-1.386653
H	-7.083924	12.512914	-1.105312
C	-7.457120	15.326591	-2.461387
H	-7.678524	16.364211	-2.180531
H	-8.442086	14.891224	-2.673411
C	-6.707032	15.391632	-3.814712
H	-7.392513	14.984306	-4.568881
H	-6.627917	16.455494	-4.072573
C	-5.380172	13.640567	-4.909313
H	-4.586238	13.751291	-5.658478
H	-5.238996	12.671707	-4.422214
H	-6.322525	13.577909	-5.467732
C	-4.300774	15.715464	-4.179725
H	-3.693701	15.446694	-5.053329
H	-4.685356	16.724900	-4.371739
H	-3.626359	15.794060	-3.321970
N	7.938483	13.579016	-0.047764
C	7.064619	14.119649	0.825845
H	6.688309	15.105204	0.592230
C	6.645712	13.467730	2.002411
H	5.952257	13.980459	2.656267
C	7.155426	12.182648	2.319124
C	8.062584	11.624672	1.383294
H	8.504136	10.647605	1.533871
C	8.416687	12.348575	0.228207
H	9.108076	11.919815	-0.482850
C	6.789261	11.496409	3.516144
C	5.621623	11.428123	5.461087
C	4.661945	12.016784	6.340871
C	4.059716	13.270542	6.060821
H	4.302550	13.832136	5.168013
C	3.136105	13.828290	6.965635
H	2.685663	14.788161	6.755642
C	3.322551	12.023611	8.391000
H	3.016818	11.549734	9.312217
C	4.257076	11.389569	7.546594
H	4.664013	10.433889	7.849787
C	6.963157	9.693055	4.881125
C	7.450596	8.371622	5.116221
C	7.146784	7.656645	6.301247
H	6.562519	8.106885	7.091495
C	8.260608	7.692848	4.171050
H	8.549797	8.154565	3.235599
C	8.708799	6.384979	4.437543

H	9.328122	5.872111	3.715089
C	7.636015	6.346800	6.479990
H	7.404627	5.804394	7.385565
C	3.433941	3.176396	-2.947501
H	4.358222	3.208697	-3.506646
C	3.466454	3.178062	-1.539240
H	4.430681	3.206030	-1.047848
C	1.127639	3.097311	-2.974299
H	0.216659	3.065325	-3.554741
C	1.062589	3.096819	-1.566782
H	0.088474	3.055704	-1.096605
C	2.255909	3.136202	-0.801729
C	2.240111	3.130232	0.626006
C	1.085801	3.111231	2.582013
C	3.354198	3.143151	2.603900
C	4.589835	3.213224	3.316797
C	4.648056	3.179299	4.732501
H	3.750301	3.076957	5.328004
C	5.835045	3.322212	2.646372
H	5.901049	3.350460	1.566020
C	7.031209	3.388731	3.385653
H	7.977468	3.470683	2.869904
C	5.894676	3.252657	5.388372
H	5.939914	3.221267	6.467310
C	-0.165636	3.130508	3.270149
C	-1.398993	3.138050	2.569955
H	-1.439850	3.138875	1.488398
C	-2.613544	3.121407	3.280240
H	-3.551237	3.120265	2.742973
C	-1.517565	3.107691	5.311764
H	-1.586268	3.087510	6.390069
C	-0.253654	3.131459	4.685740
H	0.633775	3.129366	5.305077
C	-3.741883	13.595465	0.765584
H	-3.493145	14.625626	0.554228
C	-4.754978	11.641952	0.070014
H	-5.311950	11.118409	-0.694129
C	-4.395544	10.975000	1.257226
H	-4.699946	9.943500	1.378778
C	-3.669963	11.660629	2.264135
C	-3.340643	13.011278	1.983490
H	-2.793500	13.619434	2.692291
C	-3.305185	11.031816	3.493090
C	-3.314364	9.211432	4.847917
C	-2.329389	11.133756	5.540404
N	-4.455170	5.127128	5.519090
C	-3.685945	7.850805	5.074830
C	-4.390000	7.097304	4.101742
H	-4.684323	7.525642	3.152203
C	-4.747289	5.761679	4.365799
H	-5.294750	5.196867	3.624116
C	-3.386041	7.173278	6.283276
H	-2.871369	7.669158	7.095882
C	-3.783846	5.831158	6.455005
H	-3.564174	5.321584	7.382363
C	-1.580276	11.870221	6.508293
C	-1.167637	11.302365	7.739169
H	-1.430047	10.287800	8.007041
C	-0.412441	12.071056	8.648360
H	-0.108808	11.644029	9.592288
C	-0.430071	13.898624	7.250252
H	-0.137849	14.923745	7.072494
C	-1.186445	13.212979	6.281801
H	-1.455136	13.738551	5.374964
C	3.322291	6.106840	-5.118893
H	4.268886	5.613440	-5.287894
C	1.018802	5.996860	-5.157785
H	0.130257	5.415910	-5.359642
C	0.898241	7.322999	-4.699318
H	-0.094892	7.731585	-4.562579
C	3.299937	7.438723	-4.660239
H	4.243804	7.940831	-4.489923
C	2.060750	8.089639	-4.433311
C	1.986946	9.436064	-3.963571
C	0.750786	11.232362	-3.330330
C	3.017064	11.345478	-3.296257
C	-0.521619	11.836322	-3.096125
C	-1.735366	11.135329	-3.310104
H	-1.745721	10.111545	-3.662078
C	-2.968418	11.774698	-3.076321
H	-3.891824	11.239082	-3.245730
C	-1.925323	13.728840	-2.433644
H	-2.019421	14.750029	-2.093421
C	-0.647563	13.173111	-2.640216
H	0.219046	13.795646	-2.460127
C	4.213990	12.077324	-3.033291
C	4.189388	13.414194	-2.561500
H	3.258528	13.936868	-2.384028

C	5.397659	14.101737	-2.335161
H	5.377024	15.124172	-1.985976
C	5.498338	11.512546	-3.237295
H	5.622650	10.499233	-3.597815
C	6.652757	12.276915	-2.979554
H	7.629967	11.843292	-3.138368
C	11.060333	14.459018	-0.737044
H	10.662954	13.468496	-0.974895
H	12.036223	14.532420	-1.232879
H	11.260396	14.484446	0.341464
C	9.689047	16.361240	-0.027365
H	8.602932	16.323083	0.095147
H	10.139044	16.046919	0.922710
H	9.955324	17.417660	-0.157023
C	10.682560	16.336686	-2.271132
H	10.721415	17.395713	-1.985671
H	11.732363	16.069275	-2.447301
C	9.982640	16.278637	-3.651428
H	9.761949	17.317140	-3.929657
H	10.746946	15.965637	-4.374247
C	7.576006	16.246934	-4.127186
H	6.857952	16.249400	-3.302618
H	7.821808	17.296545	-4.332004
H	7.053909	15.873858	-5.017171
C	8.969168	14.329128	-4.747109
H	8.209461	14.315163	-5.538724
H	9.940776	14.380522	-5.254222
H	8.930982	13.361243	-4.238812
N	-4.622339	0.897370	5.661699
N	-6.429603	2.989320	6.596574
C	-4.586292	0.421369	4.252846
H	-3.682048	-0.154134	4.018379
H	-5.435440	-0.222821	3.992734
H	-4.619236	1.273464	3.568662
C	-3.428672	0.423280	6.410803
H	-2.733273	-0.152498	5.787299
H	-2.878174	1.276423	6.814645
H	-3.681320	-0.220776	7.262378
C	-5.880933	0.463879	6.336341
H	-5.661998	-0.165589	7.208249
H	-6.472835	-0.186689	5.679758
C	-6.841498	1.573844	6.828282
H	-7.814699	1.369044	6.363956
H	-7.011090	1.385348	7.896159
C	-7.398643	3.687390	5.709973
H	-7.806011	4.602449	6.157840
H	-6.910929	3.971898	4.773001
H	-8.262205	3.066578	5.440524
C	-6.273900	3.714274	7.885804
H	-6.876866	4.629635	7.937188
H	-6.556221	3.111425	8.758134
H	-5.229399	4.001748	8.028389
N	9.347874	1.600399	5.927265
N	10.705555	4.028118	6.809608
N	10.101444	15.508425	-1.173916
N	8.762626	15.426566	-3.764878
N	-6.785075	14.605047	-1.341432
N	-5.373268	14.726119	-3.892808
N	5.905705	12.112841	4.331399
N	6.125704	10.221505	5.807739
N	7.345630	10.285651	3.727865
N	8.409369	5.717433	5.570542
N	2.775330	13.225397	8.115769
N	2.289132	3.131039	-3.659144
N	3.432995	3.187183	1.255451
N	1.032836	3.116224	1.230294
N	2.213414	3.106384	3.332337
N	-2.679777	3.096386	4.625793
N	7.070030	3.349979	4.732456
N	-4.443698	12.930822	-0.175181
N	-3.640064	9.732041	3.644069
N	-2.629920	9.842964	5.829243
N	-2.646936	11.785164	4.397586
N	-0.043181	13.348589	8.419191
N	2.205756	5.392677	-5.370414
N	0.753803	9.952544	-3.763300
N	1.845655	11.979383	-3.064466
N	3.155723	10.072899	-3.729051
N	6.612093	13.552333	-2.542905
N	-3.070596	13.050701	-2.651077
Pd	1.533003	14.204883	9.508232
Pd	-4.913664	13.826482	-2.016303
Pd	8.875985	3.678333	5.768461
Pd	8.352442	14.516239	-1.881274
Pd	2.307787	3.332647	-5.755808
Pd	-4.536460	3.031856	5.612144
C	2.317667	6.075826	6.482987
C	2.839156	6.312729	5.202692

C	1.970615	6.515789	4.084057
C	0.652872	6.962328	4.369565
C	0.342716	7.223629	5.735378
C	1.033822	6.575524	6.766288
H	3.382609	6.034799	2.517367
H	1.694599	6.160557	0.710725
H	3.883943	6.090504	4.997108
C	2.353806	6.294111	2.740193
C	-0.278973	7.097110	3.311911
H	-0.591163	7.732432	5.962668
H	-0.646679	6.817467	1.223307
C	0.082782	6.762610	2.025454
C	1.412765	6.379831	1.734194
H	-1.287819	7.433599	3.531427
C	0.376471	6.458636	8.121815
C	-0.476130	5.183186	8.222502
H	-0.267584	7.330842	8.282698
H	1.109962	6.462385	8.933969
H	-0.993451	5.132064	9.184494
H	-1.220397	5.173545	7.417311
H	0.139059	4.286057	8.121625
C	3.184689	5.428623	7.544524
C	3.997429	6.422055	8.385864
H	3.871032	4.725982	7.061274
H	2.561718	4.820034	8.207650
H	4.559822	5.907873	9.167183
H	4.704528	6.976640	7.760904
H	3.349842	7.151938	8.875287
C	3.438054	9.016671	4.191841
C	3.082477	8.641599	5.587925
C	1.859719	9.134439	5.871424
C	1.380493	9.900134	4.690914
H	3.867444	8.318852	6.284135
H	1.394631	9.284425	6.846574
C	2.392050	10.472974	2.424689
C	1.160704	10.190939	1.559133
C	2.577102	11.981309	2.667838
H	3.280654	10.080489	1.918950
C	1.251189	10.963412	0.234119
H	0.265085	10.496946	2.109479
H	1.082243	9.117664	1.376370
C	2.667990	12.743359	1.343073
H	1.720547	12.344069	3.249393
H	3.475513	12.144651	3.272255
C	1.436836	12.468958	0.469985
H	0.349663	10.777091	-0.359545
H	2.099874	10.579329	-0.348904
H	2.769487	13.815752	1.535483
H	3.572501	12.424393	0.803808
H	1.526293	13.000826	-0.482915
H	0.547765	12.870331	0.974210
N	2.358667	9.751445	3.700423
O	0.376758	10.582197	4.587695
O	4.460670	8.778571	3.573514

Table5_1c_TSi_TSi-ii_0wat

Property	Value
Charge	0
Electronic Energy, BS1 (a.u.)	-1137.522638
Thermal and entropic correction, BS1 (a.u.)	2.809545
Electronic Energy, BS2 (a.u.)	-1137.898301
Number of Imaginary Frequencies	0
Imaginary frequencies (cm-1)	None

Molecular Geometry in Cartesian Coordinates

C	8.236440	0.953731	6.677765
H	8.576876	0.408286	7.566921
H	7.529976	1.711731	7.025848
H	7.673778	0.232351	6.072132
C	9.433082	1.063075	4.542410
H	8.660410	0.315333	4.324171
H	9.318581	1.874750	3.818559
H	10.395370	0.582475	4.325359
C	10.642318	1.435058	6.645206
H	11.361531	0.878579	6.030767
H	10.517821	0.811642	7.539920
C	11.367280	2.722523	7.106769
H	12.372908	2.685578	6.668555
H	11.541793	2.609061	8.184373
C	11.558450	4.867287	5.928786
H	11.071308	5.007135	4.959565
H	11.753049	5.863379	6.345711

H	12.540891	4.422505	5.726875
C	10.367635	4.748459	8.065357
H	9.282697	4.836146	8.165262
H	10.726760	4.237375	8.967402
H	10.785408	5.762391	8.100887
N	3.146376	15.077980	10.601136
N	0.245968	15.182060	10.903836
C	3.953300	13.962794	11.163103
H	3.462693	13.007569	10.957986
H	4.965646	13.913550	10.742946
H	4.075096	14.024621	12.251778
C	3.945922	15.881620	9.638191
H	3.448425	15.905448	8.664885
H	4.073719	16.925996	9.949596
H	4.956003	15.482266	9.483684
C	2.602194	15.942300	11.689317
H	3.003477	15.639768	12.665094
H	2.938912	16.980397	11.572664
C	1.063162	15.995729	11.850886
H	0.782036	17.055511	11.801056
H	0.851946	15.712068	12.889937
C	-0.676882	16.047329	10.121089
H	-1.724224	15.727570	10.190685
H	-0.655705	17.097889	10.437080
H	-0.400035	16.032078	9.063317
C	-0.504757	14.119555	11.623692
H	-0.152858	13.134005	11.305278
H	-0.377489	14.163704	12.712533
H	-1.585497	14.159268	11.438490
N	2.369391	1.233755	-6.121822
N	2.341986	3.565397	-7.875052
C	3.592755	0.709787	-5.458847
H	3.370356	0.024078	-4.631677
H	4.253558	0.162782	-6.142845
H	4.179466	1.537915	-5.051329
C	1.143707	0.648036	-5.516943
H	1.360836	-0.028142	-4.680606
H	0.498143	1.444844	-5.136616
H	0.543552	0.070582	-6.231149
C	2.410853	0.984127	-7.592460
H	1.566776	0.356862	-7.906653
H	3.296970	0.396436	-7.864515
C	2.405598	2.222302	-8.522835
H	3.295711	2.136206	-9.159218
H	1.566560	2.083383	-9.216590
C	1.097920	4.283433	-8.260171
H	1.291387	5.277997	-8.681121
H	0.502107	3.744335	-9.007253
H	0.456963	4.416644	-7.384170
C	3.545947	4.372923	-8.206351
H	3.299190	5.351773	-8.636449
H	4.135674	4.550730	-7.302608
H	4.212172	3.880077	-8.925382
C	-6.497316	15.526568	-0.212523
H	-6.907684	15.174536	0.742252
H	-5.416518	15.634025	-0.086318
H	-6.897559	16.536782	-0.365595
C	-7.606155	13.448698	-0.890215
H	-7.814114	13.468069	0.186864
H	-8.581242	13.389635	-1.389655
H	-7.085594	12.511970	-1.107838
C	-7.457570	15.326367	-2.462670
H	-7.679030	16.363888	-2.181490
H	-8.442448	14.891215	-2.675545
C	-6.706568	15.391939	-3.815467
H	-7.391792	14.985486	-4.570342
H	-6.626720	16.455928	-4.072575
C	-5.380201	13.640215	-4.909693
H	-4.586172	13.750785	-5.658776
H	-5.239306	12.671310	-4.422602
H	-6.322511	13.577777	-5.468207
C	-4.300024	15.714544	-4.179677
H	-3.692676	15.445366	-5.052967
H	-4.684033	16.724139	-4.372004
H	-3.625931	15.792935	-3.321657
N	7.941963	13.580478	-0.046620
C	7.068948	14.121642	0.827520
H	6.693538	15.107673	0.594449
C	6.649820	13.469706	2.004001
H	5.957093	13.982861	2.658292
C	7.158345	12.184000	2.320054
C	8.064673	11.625504	1.383737
H	8.505360	10.647967	1.533804
C	8.419064	12.349475	0.228779
H	9.109844	11.920331	-0.482647
C	6.791698	11.497596	3.516829
C	5.623041	11.428928	5.461214
C	4.662633	12.017212	6.340453

C	4.060954	13.271286	6.060648
H	4.304897	13.833458	5.168513
C	3.136268	13.828458	6.964730
H	2.686034	14.788449	6.754795
C	3.320840	12.022751	8.389018
H	3.013842	11.548315	9.309536
C	4.256241	11.389158	7.545235
H	4.662582	10.433145	7.848176
C	6.963702	9.693313	4.880803
C	7.451062	8.371891	5.116011
C	7.147447	7.657247	6.301268
H	6.563491	8.107800	7.091531
C	8.261017	7.692921	4.170951
H	8.550091	8.154421	3.235358
C	8.709413	6.385209	4.437856
H	9.328814	5.872221	3.715555
C	7.636840	6.347541	6.480404
H	7.405721	5.805568	7.386305
C	3.432792	3.182699	-2.947480
H	4.357036	3.215578	-3.506650
C	3.465275	3.184455	-1.539219
H	4.429468	3.213023	-1.047795
C	1.126510	3.102154	-2.974328
H	0.215554	3.069686	-3.554782
C	1.061452	3.101625	-1.566816
H	0.087352	3.059943	-1.096651
C	2.254730	3.141736	-0.801741
C	2.238886	3.135528	0.625989
C	1.084557	3.114734	2.581972
C	3.352915	3.148531	2.603884
C	4.588590	3.217978	3.316757
C	4.646800	3.183882	4.732454
H	3.748969	3.082731	5.328041
C	5.833873	3.326131	2.646302
H	5.899867	3.354506	1.565951
C	7.030139	3.391294	3.385538
H	7.976462	3.472458	2.869779
C	5.893534	3.255761	5.388233
H	5.938851	3.224099	6.467141
C	-0.166927	3.131470	3.270085
C	-1.400256	3.137205	2.569833
H	-1.441080	3.138620	1.488273
C	-2.614802	3.117683	3.280041
H	-3.552445	3.114987	2.742705
C	-1.518924	3.104636	5.311626
H	-1.587650	3.083356	6.389910
C	-0.255020	3.131243	4.685676
H	0.632395	3.130195	5.305041
C	-3.743147	13.593899	0.765334
H	-3.494847	14.624263	0.554481
C	-4.756712	11.640752	0.069475
H	-5.314446	11.117671	-0.694427
C	-4.396048	10.973028	1.255915
H	-4.700234	9.941428	1.377186
C	-3.669651	11.658136	2.262579
C	-3.340608	13.008955	1.982417
H	-2.792852	13.616758	2.691045
C	-3.303969	11.028809	3.490992
C	-3.312763	9.208343	4.845697
C	-2.327404	11.130567	5.537989
N	-4.454421	5.124502	5.517886
C	-3.684798	7.847902	5.073003
C	-4.389809	7.094603	4.100450
H	-4.684699	7.523018	3.151114
C	-4.747272	5.759078	4.364796
H	-5.295385	5.194379	3.623510
C	-3.384325	7.170353	6.281317
H	-2.869093	7.666141	7.093626
C	-3.782158	5.828289	6.453310
H	-3.561814	5.318579	7.380443
C	-1.579552	11.867621	6.506378
C	-1.166633	11.299875	7.737190
H	-1.427899	10.284913	8.004648
C	-0.412340	12.069101	8.646660
H	-0.108321	11.642099	9.590460
C	-0.431565	13.897005	7.249043
H	-0.140252	14.922426	7.071552
C	-1.187082	13.210851	6.280308
H	-1.456028	13.736335	5.373506
C	3.321896	6.109022	-5.121390
H	4.268429	5.615567	-5.290605
C	1.018454	5.999797	-5.161327
H	0.129804	5.419380	-5.364187
C	0.898087	7.325506	-4.701585
H	-0.094986	7.734255	-4.564898
C	3.299785	7.440444	-4.661392
H	4.243729	7.942121	-4.490245
C	2.060718	8.091492	-4.434254

C	1.987092	9.437450	-3.963168
C	0.750990	11.233303	-3.328555
C	3.017257	11.346238	-3.294137
C	-0.521561	11.836986	-3.094491
C	-1.735088	11.135973	-3.309705
H	-1.745139	10.112457	-3.662461
C	-2.968371	11.774928	-3.076164
H	-3.891596	11.239272	-3.246451
C	-1.925938	13.728762	-2.431424
H	-2.020458	14.749680	-2.090509
C	-0.647945	13.173400	-2.637641
H	0.218494	13.795893	-2.456591
C	4.214318	12.077797	-3.030954
C	4.190059	13.414515	-2.558699
H	3.259315	13.937301	-2.380924
C	5.398539	14.101732	-2.332358
H	5.378263	15.124082	-1.982944
C	5.498530	11.512811	-3.235311
H	5.622613	10.499580	-3.596139
C	6.653163	12.276763	-2.977346
H	7.630259	11.842912	-3.136252
C	11.062429	14.460225	-0.738483
H	10.664746	13.469494	-0.974937
H	12.038167	14.532856	-1.234740
H	11.262912	14.486923	0.339915
C	9.692061	16.363759	-0.030632
H	8.606040	16.325714	0.092745
H	10.142791	16.050859	0.919568
H	9.958184	17.419992	-0.162089
C	10.684237	16.335835	-2.274979
H	10.724616	17.395075	-1.990507
H	11.733520	16.067102	-2.452225
C	9.982479	16.277380	-3.654335
H	9.761871	17.315823	-3.932850
H	10.745728	15.963684	-4.377968
C	7.575172	16.246403	-4.126889
H	6.858070	16.249225	-3.301484
H	7.821044	17.295894	-4.332236
H	7.051937	15.873276	-5.016182
C	8.966783	14.327818	-4.747818
H	8.205847	14.313641	-5.538240
H	9.937584	14.378804	-5.256510
H	8.929258	13.360164	-4.239052
N	-4.625620	0.895051	5.662346
N	-6.431326	2.988992	6.595909
C	-4.590916	0.418484	4.253652
H	-3.687375	-0.157980	4.018840
H	-5.440861	-0.224952	3.994300
H	-4.623427	1.270372	3.569176
C	-3.431837	0.420247	6.410838
H	-2.737313	-0.156279	5.787054
H	-2.880346	1.273038	6.814087
H	-3.684445	-0.223355	7.262766
C	-5.884128	0.462965	6.338058
H	-5.665154	-0.165872	7.210410
H	-6.476763	-0.187820	5.682352
C	-6.843766	1.573974	6.829482
H	-7.817359	1.369196	6.365968
H	-7.012821	1.386701	7.897658
C	-7.399914	3.686148	5.708101
H	-7.807192	4.601899	6.154628
H	-6.911801	3.969310	4.770930
H	-8.263544	3.065198	5.439180
C	-6.275725	3.715704	7.884173
H	-6.878908	4.630982	7.934354
H	-6.557884	3.113994	8.757340
H	-5.231337	4.003679	8.026355
N	9.345176	1.600135	5.926729
N	10.704791	4.026432	6.809917
N	10.103553	15.509279	-1.176253
N	8.761960	15.425761	-3.765778
N	-6.786397	14.604187	-1.342598
N	-5.373125	14.725731	-3.893149
N	5.908858	12.114510	4.332493
N	6.125879	10.221606	5.807156
N	7.346534	10.285995	3.727726
N	8.410221	5.717974	5.571110
N	2.774092	13.224791	8.114023
N	2.288010	3.136566	-3.659113
N	3.431724	3.192974	1.255450
N	1.031613	3.120388	1.230255
N	2.212146	3.110943	3.332315
N	-2.681074	3.091568	4.625568
N	7.068953	3.352031	4.732322
N	-4.445834	12.929850	-0.175177
N	-3.638970	9.729058	3.642003
N	-2.627426	9.839609	5.826580
N	-2.645069	11.781906	4.395198

N	-0.044274	13.347047	8.417870
N	2.205277	5.395372	-5.374054
N	0.754017	9.953882	-3.762621
N	1.845861	11.980058	-3.061856
N	3.155865	10.073954	-3.727855
N	6.612804	13.552054	-2.540335
N	-3.070969	13.050581	-2.650055
Pd	1.531628	14.204021	9.506531
Pd	-4.914642	13.825766	-2.016490
Pd	8.875081	3.678491	5.768493
Pd	8.353859	14.516531	-1.881150
Pd	2.306734	3.334730	-5.756132
Pd	-4.537851	3.029385	5.611836
C	2.315751	6.090676	6.499489
C	2.866311	6.454472	5.219935
C	1.970604	6.524394	4.079915
C	0.656822	6.962431	4.361948
C	0.401607	7.315187	5.739607
C	1.050077	6.569628	6.776577
H	3.388641	6.047197	2.522929
H	1.699021	6.166065	0.711929
H	3.896299	6.173137	5.007907
C	2.355766	6.293602	2.748074
C	-0.277424	7.086750	3.318101
H	-0.553490	7.784209	5.964162
H	-0.642055	6.814974	1.222905
C	0.086829	6.755646	2.022703
C	1.412464	6.380365	1.735338
H	-1.284948	7.427580	3.534249
C	0.375722	6.447309	8.123543
C	-0.476800	5.172823	8.224126
H	-0.267328	7.320921	8.282091
H	1.103584	6.452592	8.938310
H	-0.994992	5.119219	9.184115
H	-1.219887	5.159814	7.417588
H	0.143040	4.277115	8.123643
C	3.187846	5.428836	7.543351
C	3.997400	6.427446	8.388261
H	3.878676	4.733729	7.053921
H	2.572916	4.815838	8.207351
H	4.566140	5.914081	9.166872
H	4.700910	6.989077	7.766342
H	3.342804	7.150732	8.880589
C	3.441228	8.999323	4.187577
C	3.068365	8.512410	5.554922
C	1.793754	9.046337	5.853512
C	1.382042	9.891971	4.698787
H	3.861389	8.393950	6.279061
H	1.448506	9.317621	6.841434
C	2.398092	10.473221	2.431991
C	1.160731	10.193572	1.566854
C	2.583632	11.981297	2.671153
H	3.284549	10.078904	1.923883
C	1.249199	10.965536	0.241346
H	0.267364	10.501715	2.119667
H	1.079396	9.120429	1.384544
C	2.672538	12.742866	1.342996
H	1.729274	12.345849	3.251760
H	3.483835	12.146162	3.270188
C	1.438492	12.470764	0.473306
H	0.345786	10.777936	-0.349996
H	2.095564	10.579535	-0.343788
H	2.776527	13.815100	1.535018
H	3.574990	12.422052	0.801366
H	1.526407	13.002251	-0.479946
H	0.551665	12.874080	0.979903
N	2.366607	9.754973	3.713931
O	0.389700	10.592898	4.594389
O	4.465663	8.776242	3.568385

Table5_1c_TSi_DG_0wat

Property	Value
Charge	0
Electronic Energy, BS1 (a.u.)	-1137.573573
Thermal and entropic correction, BS1 (a.u.)	2.818359
Electronic Energy, BS2 (a.u.)	-1137.946708
Number of Imaginary Frequencies	0
Imaginary frequencies (cm-1)	None

Molecular Geometry in Cartesian Coordinates

C	8.239713	0.953243	6.678501
H	8.580645	0.408378	7.567821

H	7.532578	1.710714	7.026358
H	7.677684	0.231177	6.073102
C	9.436215	1.063023	4.543092
H	8.664292	0.314424	4.325134
H	9.320803	1.874369	3.819021
H	10.398970	0.583338	4.326086
C	10.645143	1.436631	6.645817
H	11.364869	0.880618	6.031558
H	10.521141	0.813334	7.540682
C	11.368966	2.724829	7.107114
H	12.374728	2.688518	6.669153
H	11.543317	2.611881	8.184798
C	11.558613	4.869053	5.927967
H	11.071318	5.008114	4.958711
H	11.752670	5.865469	6.344370
H	12.541295	4.424751	5.726185
C	10.368012	4.750588	8.064691
H	9.283031	4.837577	8.164663
H	10.727590	4.240252	8.966979
H	10.785081	5.764831	8.099630
N	3.147906	15.078228	10.603120
N	0.247492	15.183367	10.905385
C	3.953883	13.962780	11.165949
H	3.462802	13.007763	10.961013
H	4.966430	13.912767	10.746355
H	4.075171	14.024953	12.254658
C	3.948375	15.880911	9.640147
H	3.451323	15.904620	8.666601
H	4.076697	16.925338	9.951165
H	4.958266	15.480836	9.486262
C	2.603842	15.943413	11.690675
H	3.004957	15.641444	12.666696
H	2.940835	16.981346	11.573351
C	1.064814	15.997272	11.852125
H	0.783924	17.057095	11.801843
H	0.853490	15.714094	12.891286
C	-0.675392	16.048499	10.122518
H	-1.722766	15.728920	10.192457
H	-0.654009	17.099166	10.438149
H	-0.398770	16.032892	9.064697
C	-0.503219	14.121082	11.625566
H	-0.151357	13.135449	11.307364
H	-0.375879	14.165509	12.714388
H	-1.583966	14.160777	11.440402
N	2.371175	1.232302	-6.124762
N	2.342979	3.566625	-7.874384
C	3.595334	0.707966	-5.463557
H	3.373926	0.020906	-4.637246
H	4.255908	0.162360	-6.148887
H	4.181894	1.535802	-5.055224
C	1.146299	0.645062	-5.519718
H	1.364500	-0.032343	-4.684650
H	0.500707	1.440960	-5.137538
H	0.545772	0.068460	-6.234293
C	2.411505	0.984923	-7.595819
H	1.567087	0.358308	-7.910398
H	3.297323	0.397490	-7.869405
C	2.405903	2.224521	-8.524282
H	3.295635	2.139222	-9.161306
H	1.566434	2.086837	-9.217763
C	1.099217	4.285821	-8.258315
H	1.293113	5.280973	-8.677673
H	0.503168	3.748181	-9.006261
H	0.458320	4.417912	-7.382110
C	3.547282	4.374099	-8.204534
H	3.300953	5.353785	-8.632967
H	4.137257	4.550071	-7.300591
H	4.213117	3.882119	-8.924512
C	-6.495306	15.528100	-0.212083
H	-6.904862	15.176512	0.743207
H	-5.414431	15.635894	-0.086801
H	-6.895891	16.538141	-0.365416
C	-7.604422	13.449719	-0.887882
H	-7.811715	13.469708	0.189309
H	-8.579828	13.390254	-1.386653
H	-7.083924	12.512914	-1.105312
C	-7.457120	15.326591	-2.461387
H	-7.678524	16.364211	-2.180531
H	-8.442086	14.891224	-2.673411
C	-6.707032	15.391632	-3.814712
H	-7.392513	14.984306	-4.568881
H	-6.627917	16.455494	-4.072573
C	-5.380172	13.640567	-4.909313
H	-4.586238	13.751291	-5.658478
H	-5.238996	12.671707	-4.422214
H	-6.322525	13.577909	-5.467732
C	-4.300774	15.715464	-4.179725
H	-3.693701	15.446694	-5.053329

H	-4.685356	16.724900	-4.371739
H	-3.626359	15.794060	-3.321970
N	7.938483	13.579016	-0.047764
C	7.064619	14.119649	0.825845
H	6.688309	15.105204	0.592230
C	6.645712	13.467730	2.002411
H	5.952257	13.980459	2.656267
C	7.155426	12.182648	2.319124
C	8.062584	11.624672	1.383294
H	8.504136	10.647605	1.533871
C	8.416687	12.348575	0.228207
H	9.108076	11.919815	-0.482850
C	6.789261	11.496409	3.516144
C	5.621623	11.428123	5.461087
C	4.661945	12.016784	6.340871
C	4.059716	13.270542	6.060821
H	4.302550	13.832136	5.168013
C	3.136105	13.828290	6.965635
H	2.685663	14.788161	6.755642
C	3.322551	12.023611	8.391000
H	3.016818	11.549734	9.312217
C	4.257076	11.389569	7.546594
H	4.664013	10.433889	7.849787
C	6.963157	9.693055	4.881125
C	7.450596	8.371622	5.116221
C	7.146784	7.656645	6.301247
H	6.562519	8.106885	7.091495
C	8.260608	7.692848	4.171050
H	8.549797	8.154565	3.235599
C	8.708799	6.384979	4.437543
H	9.328122	5.872111	3.715089
C	7.636015	6.346800	6.479990
H	7.404627	5.804394	7.385565
C	3.433941	3.176396	-2.947501
H	4.358222	3.208697	-3.506646
C	3.466454	3.178062	-1.539240
H	4.430681	3.206030	-1.047848
C	1.127639	3.097311	-2.974299
H	0.216659	3.065325	-3.554741
C	1.062589	3.096819	-1.566782
H	0.088474	3.055704	-1.096605
C	2.255909	3.136202	-0.801729
C	2.240111	3.130232	0.626006
C	1.085801	3.111231	2.582013
C	3.354198	3.143151	2.603900
C	4.589835	3.213224	3.316797
C	4.648056	3.179299	4.732501
H	3.750301	3.076957	5.328004
C	5.835045	3.322212	2.646372
H	5.901049	3.350460	1.566020
C	7.031209	3.388731	3.385653
H	7.977468	3.470683	2.869904
C	5.894676	3.252657	5.388372
H	5.939914	3.221267	6.467310
C	-0.165636	3.130508	3.270149
C	-1.398993	3.138050	2.569955
H	-1.439850	3.138875	1.488398
C	-2.613544	3.121407	3.280240
H	-3.551237	3.120265	2.742973
C	-1.517565	3.107691	5.311764
H	-1.586268	3.087510	6.390069
C	-0.253654	3.131459	4.685740
H	0.633775	3.129366	5.305077
C	-3.741883	13.595465	0.765584
H	-3.493145	14.625626	0.554228
C	-4.754978	11.641952	0.070014
H	-5.311950	11.118409	-0.694129
C	-4.395544	10.975000	1.257226
H	-4.699946	9.943500	1.378778
C	-3.669963	11.660629	2.264135
C	-3.340643	13.011278	1.983490
H	-2.793500	13.619434	2.692291
C	-3.305185	11.031816	3.493090
C	-3.314364	9.211432	4.847917
C	-2.329389	11.133756	5.540404
N	-4.455170	5.127128	5.519090
C	-3.685945	7.850805	5.074830
C	-4.390000	7.097304	4.101742
H	-4.684323	7.525642	3.152203
C	-4.747289	5.761679	4.365799
H	-5.294750	5.196867	3.624116
C	-3.386041	7.173278	6.283276
H	-2.871369	7.669158	7.095882
C	-3.783846	5.831158	6.455005
H	-3.564174	5.321584	7.382363
C	-1.580276	11.870221	6.508293
C	-1.167637	11.302365	7.739169
H	-1.430047	10.287800	8.007041

C	-0.412441	12.071056	8.648360
H	-0.108808	11.644029	9.592288
C	-0.430071	13.898624	7.250252
H	-0.137849	14.923745	7.072494
C	-1.186445	13.212979	6.281801
H	-1.455136	13.738551	5.374964
C	3.322291	6.106840	-5.118893
H	4.268886	5.613440	-5.287894
C	1.018802	5.996860	-5.157785
H	0.130257	5.415910	-5.359642
C	0.898241	7.322999	-4.699318
H	-0.094892	7.731585	-4.562579
C	3.299937	7.438723	-4.660239
H	4.243804	7.940831	-4.489923
C	2.060750	8.089639	-4.433311
C	1.986946	9.436064	-3.963571
C	0.750786	11.232362	-3.330330
C	3.017064	11.345478	-3.296257
C	-0.521619	11.836322	-3.096125
C	-1.735366	11.135329	-3.310104
H	-1.745721	10.111545	-3.662078
C	-2.968418	11.774698	-3.076321
H	-3.891824	11.239082	-3.245730
C	-1.925323	13.728840	-2.433644
H	-2.019421	14.750029	-2.093421
C	-0.647563	13.173111	-2.640216
H	0.219046	13.795646	-2.460127
C	4.213990	12.077324	-3.033291
C	4.189388	13.414194	-2.561500
H	3.258528	13.936868	-2.384028
C	5.397659	14.101737	-2.335161
H	5.377024	15.124172	-1.985976
C	5.498338	11.512546	-3.237295
H	5.622650	10.499233	-3.597815
C	6.652757	12.276915	-2.979554
H	7.629967	11.843292	-3.138368
C	11.060333	14.459018	-0.737044
H	10.662954	13.468496	-0.974895
H	12.036223	14.532420	-1.232879
H	11.260396	14.484446	0.341464
C	9.689047	16.361240	-0.027365
H	8.602932	16.323083	0.095147
H	10.139044	16.046919	0.922710
H	9.955324	17.417660	-0.157023
C	10.682560	16.336686	-2.271132
H	10.721415	17.395713	-1.985671
H	11.732363	16.069275	-2.447301
C	9.982640	16.278637	-3.651428
H	9.761949	17.317140	-3.929657
H	10.746946	15.965637	-4.374247
C	7.576006	16.246934	-4.127186
H	6.857952	16.249400	-3.302618
H	7.821808	17.296545	-4.332004
H	7.053909	15.873858	-5.017171
C	8.969168	14.329128	-4.747109
H	8.209461	14.315163	-5.538724
H	9.940776	14.380522	-5.254222
H	8.930982	13.361243	-4.238812
N	-4.622339	0.897370	5.661699
N	-6.429603	2.989320	6.596574
C	-4.586292	0.421369	4.252846
H	-3.682048	-0.154134	4.018379
H	-5.435440	-0.222821	3.992734
H	-4.619236	1.273464	3.568662
C	-3.428672	0.423280	6.410803
H	-2.733273	-0.152498	5.787299
H	-2.878174	1.276423	6.814645
H	-3.681320	-0.220776	7.262378
C	-5.880933	0.463879	6.336341
H	-5.661998	-0.165589	7.208249
H	-6.472835	-0.186689	5.679758
C	-6.841498	1.573844	6.828282
H	-7.814699	1.369044	6.363956
H	-7.011090	1.385348	7.896159
C	-7.398643	3.687390	5.709973
H	-7.806011	4.602449	6.157840
H	-6.910929	3.971898	4.773001
H	-8.262205	3.066578	5.440524
C	-6.273900	3.714274	7.885804
H	-6.876866	4.629635	7.937188
H	-6.556221	3.111425	8.758134
H	-5.229399	4.001748	8.028389
N	9.347874	1.600399	5.927265
N	10.705555	4.028118	6.809608
N	10.101444	15.508425	-1.173916
N	8.762626	15.426566	-3.764878
N	-6.785075	14.605047	-1.341432
N	-5.373268	14.726119	-3.892808

N	5.905705	12.112841	4.331399
N	6.125704	10.221505	5.807739
N	7.345630	10.285651	3.727865
N	8.409369	5.717433	5.570542
N	2.775330	13.225397	8.115769
N	2.289132	3.131039	-3.659144
N	3.432995	3.187183	1.255451
N	1.032836	3.116224	1.230294
N	2.213414	3.106384	3.332337
N	-2.679777	3.096386	4.625793
N	7.070030	3.349979	4.732456
N	-4.443698	12.930822	-0.175181
N	-3.640064	9.732041	3.644069
N	-2.629920	9.842964	5.829243
N	-2.646936	11.785164	4.397586
N	-0.043181	13.348589	8.419191
N	2.205756	5.392677	-5.370414
N	0.753803	9.952544	-3.763300
N	1.845655	11.979383	-3.064466
N	3.155723	10.072899	-3.729051
N	6.612093	13.552333	-2.542905
N	-3.070596	13.050701	-2.651077
Pd	1.533003	14.204883	9.508232
Pd	-4.913664	13.826482	-2.016303
Pd	8.875985	3.678333	5.768461
Pd	8.352442	14.516239	-1.881274
Pd	2.307787	3.332647	-5.755808
Pd	-4.536460	3.031856	5.612144
C	2.306121	6.098920	6.511854
C	2.885344	6.572533	5.231559
C	1.964842	6.527335	4.072511
C	0.658645	6.968101	4.352244
C	0.463958	7.419926	5.741151
C	1.062689	6.569751	6.783609
H	3.382609	6.040572	2.523140
H	1.688826	6.160557	0.710725
H	3.901264	6.234840	5.014429
C	2.348033	6.282565	2.751739
C	-0.278973	7.085564	3.323457
H	-0.510335	7.853674	5.962668
H	-0.646679	6.817467	1.223307
C	0.082782	6.751064	2.019681
C	1.401219	6.374058	1.734194
H	-1.282046	7.439372	3.537200
C	0.370698	6.447090	8.121815
C	-0.487676	5.177413	8.222502
H	-0.267584	7.325069	8.276925
H	1.092641	6.450839	8.939742
H	-1.010772	5.126291	9.178721
H	-1.226170	5.161999	7.411538
H	0.133286	4.280284	8.127398
C	3.178916	5.417077	7.538751
C	3.997429	6.416282	8.380091
H	3.865259	4.720209	7.043953
H	2.567491	4.808488	8.207650
H	4.571368	5.902100	9.155637
H	4.698755	6.976640	7.755131
H	3.344069	7.140392	8.875287
C	3.438054	8.987804	4.186068
C	3.047837	8.399114	5.524417
C	1.715383	8.955463	5.831010
C	1.368947	9.877040	4.696687
H	3.844350	8.468961	6.278362
H	1.487006	9.353707	6.829253
C	2.397823	10.472974	2.436235
C	1.160704	10.190939	1.564906
C	2.577102	11.981309	2.673611
H	3.286427	10.080489	1.930496
C	1.251189	10.963412	0.239892
H	0.265085	10.496946	2.115252
H	1.082243	9.117664	1.382143
C	2.667990	12.743359	1.343073
H	1.720547	12.344069	3.249393
H	3.475513	12.150424	3.272255
C	1.436836	12.468958	0.469985
H	0.349663	10.771318	-0.353772
H	2.099874	10.579329	-0.343131
H	2.769487	13.815752	1.535483
H	3.572501	12.424393	0.803808
H	1.526293	13.000826	-0.482915
H	0.547765	12.870331	0.974210
N	2.364440	9.757218	3.723517
O	0.382531	10.587970	4.587695
O	4.466443	8.784344	3.567741

Table5_1c_TSii_reactant_0wat

Property	Value
Charge	0
Electronic Energy, BS1 (a.u.)	-1137.561620
Thermal and entropic correction, BS1 (a.u.)	2.804122
Electronic Energy, BS2 (a.u.)	-1137.940845
Number of Imaginary Frequencies	0
Imaginary frequencies (cm-1)	None

Molecular Geometry in Cartesian Coordinates

C	8.333395	0.951914	6.666089
H	8.662623	0.450185	7.584723
H	7.539990	1.646444	6.953071
H	7.878183	0.174679	6.039653
C	9.648683	1.140401	4.608224
H	8.960612	0.323965	4.355409
H	9.506141	1.931337	3.866234
H	10.661859	0.746925	4.458429
C	10.685087	1.641608	6.776898
H	11.470349	1.094953	6.239057
H	10.543828	1.066299	7.700912
C	11.307600	2.996844	7.192153
H	12.331918	2.999441	6.797721
H	11.441122	2.956395	8.280750
C	11.402207	5.055883	5.858589
H	10.918754	5.103298	4.878387
H	11.542549	6.087102	6.206135
H	12.407293	4.647241	5.695637
C	10.184394	5.029695	7.983166
H	9.095773	5.061487	8.068544
H	10.561648	4.610571	8.924405
H	10.540407	6.066844	7.945066
N	2.902455	15.297589	10.768314
N	-0.008224	15.232030	10.655809
C	3.648275	14.216439	11.465852
H	3.234596	13.240664	11.192107
H	4.716681	14.205236	11.215599
H	3.589894	14.289771	12.558870
C	3.816436	16.120679	9.931446
H	3.484362	16.112283	8.889734
H	3.853242	17.172920	10.240307
H	4.852710	15.760472	9.949613
C	2.161674	16.152543	11.741737
H	2.430724	15.889821	12.772882
H	2.459278	17.204829	11.647907
C	0.614711	16.124395	11.676386
H	0.286516	17.163492	11.545120
H	0.270291	15.860139	12.684491
C	-0.892488	16.007155	9.744077
H	-1.912762	15.606268	9.696466
H	-0.991751	17.061231	10.032432
H	-0.489278	15.997320	8.727722
C	-0.753818	14.120151	11.301999
H	-0.260788	13.167068	11.087680
H	-0.803220	14.213664	12.393825
H	-1.791387	14.042405	10.953530
N	2.359411	1.143293	-6.256636
N	2.307897	3.566963	-7.880685
C	3.595479	0.597299	-5.635913
H	3.389536	-0.138977	-4.848902
H	4.257417	0.101139	-6.356549
H	4.174486	1.407432	-5.183698
C	1.146786	0.512883	-5.670615
H	1.380880	-0.207230	-4.876593
H	0.499580	1.280703	-5.237189
H	0.542424	-0.028875	-6.408858
C	2.385933	0.974025	-7.739012
H	1.542007	0.359537	-8.077967
H	3.272252	0.407262	-8.051803
C	2.363815	2.260780	-8.600816
H	3.244713	2.211409	-9.253631
H	1.514955	2.157156	-9.288743
C	1.064496	4.308675	-8.220747
H	1.259344	5.325945	-8.582812
H	0.466461	3.815570	-8.997173
H	0.424806	4.391273	-7.337604
C	3.513243	4.387212	-8.173074
H	3.268009	5.386162	-8.554952
H	4.104338	4.519073	-7.262253
H	4.177668	3.928846	-8.916145
C	-6.487412	15.579625	-0.453478
H	-6.921015	15.240398	0.495584
H	-5.407507	15.669078	-0.306689

H	-6.867008	16.595658	-0.618853
C	-7.620035	13.517377	-1.137874
H	-7.851230	13.550715	-0.065812
H	-8.584169	13.465253	-1.658795
H	-7.105531	12.572168	-1.332874
C	-7.415382	15.385582	-2.717459
H	-7.605597	16.433213	-2.451276
H	-8.412130	14.978335	-2.930567
C	-6.655044	15.408857	-4.066553
H	-7.354793	15.026926	-4.820916
H	-6.526136	16.465840	-4.332406
C	-5.424982	13.552799	-5.101447
H	-4.638078	13.594307	-5.864973
H	-5.318365	12.601276	-4.572552
H	-6.377853	13.507874	-5.643304
C	-4.242638	15.605586	-4.478501
H	-3.671530	15.279240	-5.356705
H	-4.582246	16.626087	-4.695219
H	-3.542206	15.677732	-3.641932
N	7.898115	13.543827	-0.093993
C	7.042742	14.116668	0.778112
H	6.730280	15.129087	0.565928
C	6.559301	13.463265	1.928602
H	5.882579	13.999861	2.581219
C	6.970706	12.137136	2.214506
C	7.856653	11.544590	1.279578
H	8.227317	10.535792	1.407918
C	8.287519	12.277281	0.157153
H	8.967946	11.824972	-0.550255
C	6.532870	11.444249	3.382955
C	5.395096	11.421590	5.346722
C	4.588577	12.104224	6.306642
C	4.106532	13.417825	6.077226
H	4.323782	13.952351	5.161168
C	3.329874	14.064143	7.056729
H	2.969638	15.067702	6.879304
C	3.457298	12.249561	8.470602
H	3.209610	11.814310	9.425536
C	4.236026	11.520524	7.549360
H	4.577363	10.531190	7.825738
C	6.596294	9.605854	4.713742
C	7.095953	8.292769	4.968440
C	6.784592	7.577498	6.152787
H	6.149272	8.003559	6.919617
C	7.957353	7.636714	4.052644
H	8.260325	8.098757	3.121579
C	8.459812	6.357373	4.349969
H	9.122713	5.864927	3.652541
C	7.334216	6.295766	6.364362
H	7.104948	5.756765	7.272852
C	3.413777	2.955329	-2.988307
H	4.337932	2.985099	-3.547590
C	3.447754	2.964555	-1.580336
H	4.411737	2.995951	-1.089000
C	1.108542	2.875645	-3.013273
H	0.197148	2.841363	-3.592658
C	1.043794	2.881921	-1.606355
H	0.069525	2.846758	-1.136067
C	2.237579	2.927095	-0.842641
C	2.221475	2.932612	0.584442
C	1.062984	2.876722	2.535911
C	3.330635	2.956093	2.563471
C	4.563148	3.038216	3.280579
C	4.609185	3.056308	4.696057
H	3.701999	2.996823	5.282388
C	5.814701	3.116052	2.618144
H	5.887711	3.104634	1.538068
C	7.005262	3.197753	3.366198
H	7.956346	3.251102	2.855688
C	5.850051	3.145738	5.359247
H	5.891566	3.158942	6.438206
C	-0.191106	2.861126	3.219831
C	-1.422788	2.870474	2.517006
H	-1.461319	2.879611	1.435243
C	-2.639410	2.856165	3.224194
H	-3.574724	2.858065	2.682230
C	-1.549560	2.831213	5.256319
H	-1.622669	2.810001	6.333582
C	-0.283573	2.842183	4.634531
H	0.603596	2.829051	5.254580
C	-3.689221	13.653016	0.530683
H	-3.452619	14.684062	0.309777
C	-4.751784	11.705956	-0.102895
H	-5.362297	11.186325	-0.827640
C	-4.304987	11.029291	1.047764
H	-4.594241	9.994788	1.179888
C	-3.509426	11.708370	2.004881
C	-3.198860	13.059959	1.710745

H	-2.594867	13.660546	2.378774
C	-3.062307	11.071388	3.201296
C	-3.026654	9.249929	4.556595
C	-2.021925	11.175802	5.214092
N	-4.147764	5.177936	5.283968
C	-3.381977	7.887793	4.798834
C	-4.110228	7.129028	3.847323
H	-4.419113	7.549953	2.898754
C	-4.464090	5.796574	4.129351
H	-5.024694	5.223613	3.404325
C	-3.044178	7.218022	6.003001
H	-2.499507	7.719851	6.794346
C	-3.445363	5.879453	6.198289
H	-3.203112	5.371696	7.120530
C	-1.295393	11.921983	6.190407
C	-0.994670	11.387261	7.466470
H	-1.344233	10.407987	7.758343
C	-0.264200	12.147705	8.398786
H	-0.040389	11.730552	9.369676
C	-0.117527	13.930549	6.942875
H	0.229444	14.936841	6.755122
C	-0.837702	13.241472	5.946896
H	-1.024737	13.741108	5.004796
C	3.294032	5.962074	-5.008906
H	4.239773	5.468033	-5.179452
C	0.989366	5.855814	-5.057440
H	0.100866	5.277172	-5.265881
C	0.869095	7.176804	-4.583961
H	-0.123336	7.585400	-4.442732
C	3.271907	7.287282	-4.532073
H	4.215600	7.785206	-4.349450
C	2.032541	7.939406	-4.309237
C	1.960385	9.286461	-3.841801
C	0.728983	11.101441	-3.259771
C	2.994953	11.203023	-3.205403
C	-0.538073	11.739046	-3.100597
C	-1.757253	11.065932	-3.364922
H	-1.775215	10.034060	-3.691484
C	-2.981994	11.745344	-3.214955
H	-3.908988	11.230495	-3.422487
C	-1.920172	13.683360	-2.557247
H	-2.001177	14.713210	-2.239511
C	-0.650314	13.088064	-2.679102
H	0.222350	13.686960	-2.452296
C	4.191053	11.950115	-2.985683
C	4.162248	13.302978	-2.562623
H	3.229739	13.821558	-2.380514
C	5.366738	14.008322	-2.379616
H	5.339797	15.040332	-2.060999
C	5.476521	11.387767	-3.187482
H	5.602038	10.362132	-3.510179
C	6.627270	12.172594	-2.978532
H	7.605751	11.742495	-3.137899
C	10.998289	14.402230	-0.745854
H	10.584408	13.407920	-0.935505
H	11.981270	14.432300	-1.232440
H	11.186911	14.479629	0.332159
C	9.643820	16.353695	-0.146286
H	8.555033	16.337274	-0.044460
H	10.069262	16.073900	0.825736
H	9.928143	17.399347	-0.317668
C	10.676009	16.214102	-2.369517
H	10.777581	17.274748	-2.106261
H	11.706647	15.885080	-2.554021
C	9.950399	16.172779	-3.737428
H	9.762778	17.216888	-4.018967
H	10.689761	15.826901	-4.471087
C	7.528209	16.232186	-4.134282
H	6.830128	16.231765	-3.292403
H	7.801780	17.278953	-4.316544
H	6.973857	15.899806	-5.020881
C	8.833344	14.278612	-4.827508
H	8.040357	14.294972	-5.585590
H	9.782853	14.313152	-5.376061
H	8.791893	13.304671	-4.331513
N	-4.944121	1.048019	5.773606
N	-6.416635	3.451856	6.522676
C	-4.976536	0.456166	4.409560
H	-4.167304	-0.264185	4.236108
H	-5.911249	-0.075230	4.191281
H	-4.880409	1.244780	3.657923
C	-3.831928	0.471090	6.573986
H	-3.229004	-0.251303	6.009625
H	-3.162017	1.267346	6.908320
H	-4.174738	-0.053592	7.474553
C	-6.252797	0.861186	6.466110
H	-6.129235	0.273624	7.384807
H	-6.936755	0.259137	5.854193

C	-7.033798	2.137114	6.865055
H	-8.030223	2.043762	6.414496
H	-7.220972	2.061162	7.943893
C	-7.277854	4.213703	5.579113
H	-7.526739	5.218311	5.943323
H	-6.767840	4.330433	4.618700
H	-8.233842	3.717482	5.369266
C	-6.142896	4.244860	7.750421
H	-6.591803	5.245639	7.721869
H	-6.516348	3.767607	8.665204
H	-5.064875	4.370682	7.879981
N	9.427978	1.683910	5.974603
N	10.581060	4.236555	6.789104
N	10.063456	15.445207	-1.246479
N	8.697021	15.367104	-3.822971
N	-6.774645	14.656543	-1.584095
N	-5.354453	14.679696	-4.133770
N	5.712771	12.107281	4.225982
N	5.773314	10.153876	5.637537
N	6.995417	10.188698	3.562397
N	8.164700	5.694661	5.484930
N	3.008817	13.500675	8.238901
N	2.268985	2.906357	-3.699844
N	3.414286	2.986180	1.214802
N	1.012880	2.908814	1.185612
N	2.188235	2.887829	3.287915
N	-2.711461	2.832246	4.569988
N	7.032185	3.208797	4.714499
N	-4.460041	12.996855	-0.361392
N	-3.423097	9.781543	3.380101
N	-2.320906	9.889908	5.515200
N	-2.331495	11.812842	4.062946
N	0.167524	13.401300	8.151183
N	2.176937	5.254656	-5.275357
N	0.726879	9.815324	-3.675976
N	1.825218	11.845960	-2.996507
N	3.132090	9.923729	-3.619614
N	6.582652	13.463160	-2.587521
N	-3.071530	13.033584	-2.824000
Pd	1.518488	14.351561	9.450997
Pd	-4.908718	13.841962	-2.226395
Pd	8.792651	3.710150	5.751000
Pd	8.316457	14.444722	-1.940743
Pd	2.280753	3.220123	-5.777982
Pd	-4.545643	3.131448	5.547047
C	2.670582	5.162423	7.242292
C	3.312351	5.796241	6.169153
C	2.683971	5.936866	4.889411
C	1.267569	5.905700	4.875092
C	0.615518	5.744603	6.145396
C	1.257239	5.129353	7.226126
H	4.472853	6.208910	3.701168
H	0.760398	6.376406	1.535460
H	4.385755	5.958173	6.232376
C	3.387481	6.158144	3.685135
C	0.582765	6.083285	3.651989
H	-0.463274	5.853124	6.170327
H	3.241974	6.447328	1.565112
C	1.289410	6.251252	2.476484
C	2.701252	6.290392	2.492806
H	-0.504402	6.077837	3.644487
C	3.498593	4.611105	8.387342
C	3.573219	3.071395	8.430564
H	3.106572	4.966761	9.341157
H	4.506758	5.032664	8.317070
H	4.188958	2.746516	9.272678
H	2.589602	2.621110	8.552995
H	3.995517	2.646677	7.517448
C	0.441262	4.444130	8.309995
C	-0.642728	5.278830	9.000738
H	1.098096	4.043146	9.080968
H	-0.020107	3.557931	7.850530
H	-1.273513	4.638012	9.618313
H	-0.200986	6.034279	9.648401
H	-1.274280	5.824060	8.294405
C	0.752719	8.095263	8.007189
C	1.285447	8.019525	6.631032
C	2.635434	7.992416	6.699668
C	3.041643	7.989464	8.132994
H	0.659194	8.289395	5.780389
H	3.357117	8.268552	5.923899
C	1.742822	8.139554	10.325754
C	2.408809	6.985715	11.084029
C	2.292991	9.505824	10.773973
H	0.665269	8.121441	10.522455
C	2.273067	7.179144	12.602175
H	3.466505	6.940634	10.804482
H	1.941563	6.042263	10.781262

C	2.160343	9.685250	12.293101
H	3.347320	9.566152	10.479660
H	1.739304	10.288597	10.237565
C	2.841438	8.533139	13.045040
H	2.782015	6.360534	13.120092
H	1.213140	7.118647	12.883444
H	2.593508	10.645402	12.595279
H	1.096828	9.716162	12.564340
H	2.711152	8.662730	14.123837
H	3.921600	8.559325	12.849455
N	1.854467	7.986610	8.871060
O	4.173682	8.025255	8.593855
O	-0.399589	8.272084	8.369972

Table5_1c_TSii_TSi-ii_0wat

Property	Value
Charge	0
Electronic Energy, BS1 (a.u.)	-1137.517044
Thermal and entropic correction, BS1 (a.u.)	2.807833
Electronic Energy, BS2 (a.u.)	-1137.892283
Number of Imaginary Frequencies	0
Imaginary frequencies (cm-1)	None

Molecular Geometry in Cartesian Coordinates

C	8.338658	0.951585	6.673180
H	8.668462	0.454049	7.593879
H	7.542040	1.643885	6.956704
H	7.887396	0.170582	6.048576
C	9.654781	1.139813	4.615836
H	8.969098	0.321026	4.364101
H	9.510763	1.928843	3.872115
H	10.669113	0.748707	4.467679
C	10.688277	1.648571	6.784097
H	11.475120	1.102245	6.248233
H	10.547923	1.075582	7.709686
C	11.307468	3.006502	7.195533
H	12.331167	3.011441	6.799514
H	11.442821	2.968549	8.283998
C	11.394761	5.064877	5.860413
H	10.911301	5.109843	4.880091
H	11.531275	6.096852	6.207232
H	12.401364	4.659804	5.697932
C	10.176679	5.035790	7.984827
H	9.087977	5.063080	8.070480
H	10.555775	4.619028	8.926377
H	10.528391	6.074378	7.945765
N	2.904710	15.301227	10.770384
N	-0.005880	15.232890	10.659465
C	3.650546	14.220913	11.469174
H	3.237388	13.244773	11.195977
H	4.719105	14.209906	11.219549
H	3.591375	14.295030	12.562098
C	3.818660	16.123577	9.932746
H	3.486682	16.114067	8.890994
H	3.855328	17.176138	10.240535
H	4.854971	15.763491	9.951390
C	2.163705	16.157089	11.742832
H	2.433826	15.896449	12.774232
H	2.460035	17.209548	11.646966
C	0.616735	16.126926	11.678772
H	0.287148	17.165501	11.546867
H	0.273440	15.863114	12.687377
C	-0.892182	16.006138	9.748112
H	-1.911851	15.603596	9.701621
H	-0.992882	17.060206	10.036003
H	-0.489871	15.996498	8.731415
C	-0.749158	14.120333	11.307146
H	-0.255202	13.167793	11.092513
H	-0.797111	14.214191	12.399006
H	-1.787116	14.041148	10.960159
N	2.356712	1.140315	-6.259509
N	2.305300	3.566289	-7.880107
C	3.593575	0.593334	-5.641257
H	3.388645	-0.143881	-4.854860
H	4.254386	0.097963	-6.363467
H	4.173373	1.402807	-5.188893
C	1.144840	0.509228	-5.672640
H	1.380008	-0.212148	-4.880080
H	0.498443	1.276495	-5.237003
H	0.539256	-0.031277	-6.410803
C	2.381171	0.973088	-7.742150
H	1.536289	0.359811	-8.080916

H	3.266604	0.405982	-8.056829
C	2.359248	2.261064	-8.602126
H	3.239418	2.211831	-9.255929
H	1.509585	2.159141	-9.289316
C	1.062614	4.309944	-8.218567
H	1.258500	5.327542	-8.579141
H	0.463745	3.818741	-8.995556
H	0.423277	4.391877	-7.335118
C	3.511486	4.385498	-8.171912
H	3.267275	5.385297	-8.552224
H	4.103163	4.515331	-7.261177
H	4.175007	3.927365	-8.915934
C	-6.483684	15.582462	-0.450557
H	-6.917308	15.243826	0.498709
H	-5.403689	15.671288	-0.304038
H	-6.862681	16.598677	-0.616187
C	-7.618022	13.520871	-1.134106
H	-7.849127	13.554791	-0.062043
H	-8.582228	13.469339	-1.654958
H	-7.104304	12.575171	-1.328794
C	-7.411938	15.388287	-2.714418
H	-7.601085	16.436238	-2.448733
H	-8.409119	14.981903	-2.927143
C	-6.651825	15.410111	-4.063665
H	-7.351947	15.028083	-4.817635
H	-6.522319	16.466828	-4.330278
C	-5.422958	13.552863	-5.097828
H	-4.636005	13.593538	-5.861346
H	-5.316994	12.601476	-4.568557
H	-6.375843	13.508382	-5.639694
C	-4.239268	15.605115	-4.475691
H	-3.668385	15.278061	-5.353776
H	-4.578176	16.625768	-4.692781
H	-3.538791	15.677103	-3.639148
N	7.894731	13.539128	-0.097151
C	7.040696	14.113554	0.775230
H	6.729331	15.126241	0.562728
C	6.557208	13.461324	1.926385
H	5.881669	13.999087	2.579271
C	6.967032	12.134771	2.212583
C	7.851449	11.540570	1.277225
H	8.220803	10.531311	1.405694
C	8.282575	12.272165	0.154192
H	8.961842	11.818633	-0.553559
C	6.529365	11.443068	3.381808
C	5.395663	11.423595	5.347902
C	4.590918	12.107505	6.308433
C	4.106134	13.419836	6.077633
H	4.320605	13.952914	5.160078
C	3.329910	14.066353	7.057468
H	2.967473	15.068938	6.879007
C	3.463286	12.254508	8.474337
H	3.218008	11.819841	9.430247
C	4.242486	11.526033	7.553182
H	4.587750	10.538713	7.831022
C	6.593068	9.605558	4.713876
C	7.090921	8.291655	4.968137
C	6.781287	7.577519	6.153681
H	6.147955	8.004924	6.921567
C	7.948574	7.633461	4.050330
H	8.249844	8.094425	3.118182
C	8.449472	6.353343	4.346893
H	9.109443	5.859204	3.647881
C	7.329232	6.294822	6.364449
H	7.101061	5.756381	7.273593
C	3.413011	2.946201	-2.989121
H	4.337001	2.973867	-3.548776
C	3.447709	2.955561	-1.581143
H	4.411980	2.984862	-1.090231
C	1.107646	2.871565	-3.012967
H	0.195911	2.838884	-3.591921
C	1.043572	2.878661	-1.606046
H	0.069413	2.845863	-1.135367
C	2.237797	2.921500	-0.842873
C	2.222214	2.928402	0.584215
C	1.063581	2.881611	2.535911
C	3.331561	2.951021	2.563056
C	4.564066	3.030711	3.280445
C	4.609183	3.050209	4.695858
H	3.701437	2.995698	5.281415
C	5.816191	3.105886	2.618863
H	5.889800	3.093077	1.538837
C	7.006251	3.187656	3.367865
H	7.957773	3.239534	2.858010
C	5.849490	3.139267	5.359980
H	5.889909	3.154099	6.438986
C	-0.190441	2.873057	3.220061
C	-1.422039	2.882209	2.517038

H	-1.460484	2.887064	1.435246
C	-2.638739	2.872588	3.224098
H	-3.574097	2.874222	2.682187
C	-1.548899	2.854536	5.256768
H	-1.622483	2.838615	6.334143
C	-0.282873	2.860986	4.634933
H	0.604316	2.849346	5.254969
C	-3.687213	13.654415	0.534459
H	-3.449262	14.684978	0.312743
C	-4.751675	11.708026	-0.098070
H	-5.362282	11.188446	-0.822772
C	-4.306404	11.031892	1.053461
H	-4.596935	9.997827	1.186195
C	-3.510620	11.710870	2.010485
C	-3.198274	13.061829	1.715379
H	-2.593931	13.662203	2.383286
C	-3.064916	11.074227	3.207606
C	-3.032118	9.253305	4.563663
C	-2.023929	11.177601	5.220271
N	-4.165182	5.184779	5.292966
C	-3.391089	7.892261	4.806412
C	-4.120495	7.134765	3.854797
H	-4.427454	7.555712	2.905615
C	-4.478447	5.803577	4.137574
H	-5.040139	5.231798	3.412490
C	-3.056583	7.222497	6.011385
H	-2.512246	7.723615	6.803262
C	-3.461756	5.885285	6.207250
H	-3.222691	5.377984	7.130512
C	-1.295623	11.923018	6.195901
C	-0.992405	11.387729	7.471246
H	-1.340668	10.408013	7.763367
C	-0.260901	12.148403	8.402687
H	-0.035721	11.731506	9.373334
C	-0.116660	13.931549	6.946902
H	0.229863	14.937929	6.758781
C	-0.838294	13.242582	5.951983
H	-1.026863	13.742608	5.010392
C	3.292685	5.958231	-5.007984
H	4.238254	5.463947	-5.178747
C	0.987935	5.851755	-5.053901
H	0.099280	5.272700	-5.260530
C	0.868065	7.173372	-4.582031
H	-0.124231	7.582073	-4.440150
C	3.270939	7.284089	-4.532929
H	4.214800	7.782282	-4.351912
C	2.031749	7.936378	-4.309449
C	1.960014	9.283873	-3.843159
C	0.729324	11.099235	-3.260707
C	2.995351	11.200627	-3.208567
C	-0.537376	11.737198	-3.100088
C	-1.757093	11.064458	-3.362857
H	-1.775729	10.032564	-3.689314
C	-2.981442	11.744293	-3.211336
H	-3.908891	11.229750	-3.417595
C	-1.918121	13.682023	-2.555039
H	-1.998387	14.711884	-2.237159
C	-0.648648	13.086287	-2.678504
H	0.224458	13.684935	-2.452756
C	4.191621	11.947687	-2.989754
C	4.163112	13.300748	-2.567311
H	3.230744	13.819576	-2.385195
C	5.367728	14.006008	-2.384918
H	5.340994	15.038182	-2.066813
C	5.476943	11.385117	-3.191735
H	5.602212	10.359334	-3.514057
C	6.627831	12.169894	-2.983331
H	7.606209	11.739581	-3.142777
C	10.996569	14.394669	-0.743173
H	10.581613	13.400894	-0.933246
H	11.980325	14.423283	-1.228283
H	11.183658	14.472010	0.335108
C	9.643517	16.347873	-0.145990
H	8.554538	16.332861	-0.046113
H	10.066885	16.067556	0.826789
H	9.929440	17.393161	-0.316921
C	10.679441	16.206940	-2.367381
H	10.782754	17.267275	-2.103548
H	11.709673	15.875993	-2.550702
C	9.955408	16.167689	-3.736196
H	9.769426	17.212266	-4.017089
H	10.695243	15.821501	-4.469234
C	7.533773	16.230314	-4.136209
H	6.834419	16.229898	-3.295373
H	7.808803	17.276934	-4.317099
H	6.980340	15.899379	-5.023926
C	8.837462	14.275669	-4.829091
H	8.045401	14.293439	-5.588118

H	9.787658	14.309569	-5.376491
H	8.794364	13.301415	-4.333869
N	-4.930518	1.047205	5.764559
N	-6.420651	3.436839	6.523577
C	-4.960154	0.461184	4.397934
H	-4.145834	-0.252382	4.220374
H	-5.891196	-0.076074	4.178305
H	-4.870904	1.253776	3.649676
C	-3.813408	0.474830	6.561357
H	-3.205747	-0.240590	5.993197
H	-3.149013	1.274408	6.898792
H	-4.151649	-0.056350	7.459842
C	-6.237121	0.847749	6.457452
H	-6.108303	0.257988	7.374010
H	-6.916885	0.242522	5.843995
C	-7.027716	2.116199	6.861237
H	-8.023515	2.016948	6.410569
H	-7.214021	2.034876	7.939829
C	-7.287566	4.195538	5.582741
H	-7.545409	5.196265	5.951341
H	-6.777776	4.321095	4.623309
H	-8.239074	3.692076	5.369860
C	-6.152769	4.227413	7.754109
H	-6.608001	5.225440	7.728734
H	-6.523315	3.744602	8.667165
H	-5.075553	4.359581	7.884203
N	9.431537	1.685498	5.980951
N	10.576448	4.243233	6.791397
N	10.063928	15.438837	-1.245424
N	8.701145	15.363616	-3.823953
N	-6.771726	14.659178	-1.580805
N	-5.351694	14.680090	-4.130598
N	5.711154	12.107652	4.225520
N	5.773882	10.156064	5.639460
N	6.990353	10.186920	3.561158
N	8.156213	5.691810	5.482979
N	3.011650	13.504190	8.241096
N	2.267772	2.899611	-3.700161
N	3.415364	2.978828	1.214318
N	1.013585	2.909769	1.185550
N	2.188885	2.889152	3.287676
N	-2.710473	2.853959	4.569926
N	7.032217	3.200670	4.716249
N	-4.458254	12.998326	-0.357504
N	-3.427434	9.785033	3.386914
N	-2.324145	9.892000	5.521609
N	-2.333576	11.815057	4.069308
N	0.170171	13.402157	8.154672
N	2.175344	5.250417	-5.272244
N	0.726663	9.812951	-3.676481
N	1.825893	11.843724	-2.998885
N	3.131996	9.921171	-3.622403
N	6.583537	13.460633	-2.592794
N	-3.069990	13.032601	-2.820322
Pd	1.520995	14.353775	9.453840
Pd	-4.906491	13.842815	-2.222892
Pd	8.790292	3.709454	5.753081
Pd	8.316943	14.440535	-1.942856
Pd	2.278828	3.216468	-5.777841
Pd	-4.547442	3.134560	5.547099
C	2.669619	5.169769	7.249448
C	3.303173	5.913113	6.192823
C	2.694077	5.944695	4.873952
C	1.283573	5.928472	4.866467
C	0.657744	5.910540	6.183176
C	1.287153	5.156183	7.239836
H	4.483188	6.189732	3.686843
H	0.764564	6.380194	1.533201
H	4.384815	6.015996	6.237736
C	3.397073	6.139913	3.673678
C	0.591738	6.096548	3.656116
H	-0.427628	5.979183	6.198838
H	3.244045	6.427856	1.550356
C	1.297309	6.252201	2.469205
C	2.703177	6.277379	2.478340
H	-0.495330	6.113869	3.654095
C	3.515100	4.598744	8.372478
C	3.575811	3.061629	8.423392
H	3.141167	4.959894	9.333881
H	4.527370	5.009727	8.290062
H	4.192537	2.732352	9.263092
H	2.585916	2.620377	8.554179
H	3.987236	2.625445	7.509297
C	0.451661	4.452256	8.294402
C	-0.645203	5.271522	8.986514
H	1.101735	4.045442	9.069475
H	-0.001696	3.569472	7.821795
H	-1.269158	4.616426	9.599005

H	-0.218860	6.028133	9.644665
H	-1.283997	5.811898	8.284104
C	0.732531	8.080490	8.000791
C	1.233347	7.883383	6.612905
C	2.652633	7.899770	6.680720
C	3.033057	8.008835	8.119679
H	0.666028	8.326925	5.805647
H	3.291271	8.362800	5.940836
C	1.730061	8.153419	10.317723
C	2.397170	6.990048	11.065206
C	2.283715	9.512401	10.777665
H	0.652995	8.134811	10.514334
C	2.265703	7.171305	12.585156
H	3.454086	6.947210	10.782144
H	1.928571	6.049559	10.755279
C	2.154041	9.680034	12.298436
H	3.337552	9.572670	10.482289
H	1.731459	10.303985	10.248377
C	2.835862	8.521567	13.039988
H	2.775694	6.348424	13.095225
H	1.206502	7.109008	12.868760
H	2.588847	10.637369	12.607026
H	1.091181	9.709907	12.572293
H	2.708016	8.642989	14.120023
H	3.915612	8.548530	12.842256
N	1.841203	8.014221	8.856121
O	4.157132	8.075828	8.590272
O	-0.416637	8.256558	8.365424

Table5_1c_TSii_DDG_0wat

Property	Value
Charge	0
Electronic Energy, BS1 (a.u.)	-1137.561814
Thermal and entropic correction, BS1 (a.u.)	2.808892
Electronic Energy, BS2 (a.u.)	-1137.934400
Number of Imaginary Frequencies	0
Imaginary frequencies (cm-1)	None

Molecular Geometry in Cartesian Coordinates

C	8.333395	0.951914	6.666089
H	8.662623	0.450185	7.584723
H	7.539990	1.646444	6.953071
H	7.878183	0.174679	6.039653
C	9.648683	1.140401	4.608224
H	8.960612	0.323965	4.355409
H	9.506141	1.931337	3.866234
H	10.661859	0.746925	4.458429
C	10.685087	1.641608	6.776898
H	11.470349	1.094953	6.239057
H	10.543828	1.066299	7.700912
C	11.307600	2.996844	7.192153
H	12.331918	2.999441	6.797721
H	11.441122	2.956395	8.280750
C	11.402207	5.055883	5.858589
H	10.918754	5.103298	4.878387
H	11.542549	6.087102	6.206135
H	12.407293	4.647241	5.695637
C	10.184394	5.029695	7.983166
H	9.095773	5.061487	8.068544
H	10.561648	4.610571	8.924405
H	10.540407	6.066844	7.945066
N	2.902455	15.297589	10.768314
N	-0.008224	15.232030	10.655809
C	3.648275	14.216439	11.465852
H	3.234596	13.240664	11.192107
H	4.716681	14.205236	11.215599
H	3.589894	14.289771	12.558870
C	3.816436	16.120679	9.931446
H	3.484362	16.112283	8.889734
H	3.853242	17.172920	10.240307
H	4.852710	15.760472	9.949613
C	2.161674	16.152543	11.741737
H	2.430724	15.889821	12.772882
H	2.459278	17.204829	11.647907
C	0.614711	16.124395	11.676386
H	0.286516	17.163492	11.545120
H	0.270291	15.860139	12.684491
C	-0.892488	16.007155	9.744077
H	-1.912762	15.606268	9.696466
H	-0.991751	17.061231	10.032432
H	-0.489278	15.997320	8.727722
C	-0.753818	14.120151	11.301999

H	-0.260788	13.167068	11.087680
H	-0.803220	14.213664	12.393825
H	-1.791387	14.042405	10.953530
N	2.359411	1.143293	-6.256636
N	2.307897	3.566963	-7.880685
C	3.595479	0.597299	-5.635913
H	3.389536	-0.138977	-4.848902
H	4.257417	0.101139	-6.356549
H	4.174486	1.407432	-5.183698
C	1.146786	0.512883	-5.670615
H	1.380880	-0.207230	-4.876593
H	0.499580	1.280703	-5.237189
H	0.542424	-0.028875	-6.408858
C	2.385933	0.974025	-7.739012
H	1.542007	0.359537	-8.077967
H	3.272252	0.407262	-8.051803
C	2.363815	2.260780	-8.600816
H	3.244713	2.211409	-9.253631
H	1.514955	2.157156	-9.288743
C	1.064496	4.308675	-8.220747
H	1.259344	5.325945	-8.582812
H	0.466461	3.815570	-8.997173
H	0.424806	4.391273	-7.337604
C	3.513243	4.387212	-8.173074
H	3.268009	5.386162	-8.554952
H	4.104338	4.519073	-7.262253
H	4.177668	3.928846	-8.916145
C	-6.487412	15.579625	-0.453478
H	-6.921015	15.240398	0.495584
H	-5.407507	15.669078	-0.306689
H	-6.867008	16.595658	-0.618853
C	-7.620035	13.517377	-1.137874
H	-7.851230	13.550715	-0.065812
H	-8.584169	13.465253	-1.658795
H	-7.105531	12.572168	-1.332874
C	-7.415382	15.385582	-2.717459
H	-7.605597	16.433213	-2.451276
H	-8.412130	14.978335	-2.930567
C	-6.655044	15.408857	-4.066553
H	-7.354793	15.026926	-4.820916
H	-6.526136	16.465840	-4.332406
C	-5.424982	13.552799	-5.101447
H	-4.638078	13.594307	-5.864973
H	-5.318365	12.601276	-4.572552
H	-6.377853	13.507874	-5.643304
C	-4.242638	15.605586	-4.478501
H	-3.671530	15.279240	-5.356705
H	-4.582246	16.626087	-4.695219
H	-3.542206	15.677732	-3.641932
N	7.898115	13.543827	-0.093993
C	7.042742	14.116668	0.778112
H	6.730280	15.129087	0.565928
C	6.559301	13.463265	1.928602
H	5.882579	13.999861	2.581219
C	6.970706	12.137136	2.214506
C	7.856653	11.544590	1.279578
H	8.227317	10.535792	1.407918
C	8.287519	12.277281	0.157153
H	8.967946	11.824972	-0.550255
C	6.532870	11.444249	3.382955
C	5.395096	11.421590	5.346722
C	4.588577	12.104224	6.306642
C	4.106532	13.417825	6.077226
H	4.323782	13.952351	5.161168
C	3.329874	14.064143	7.056729
H	2.969638	15.067702	6.879304
C	3.457298	12.249561	8.470602
H	3.209610	11.814310	9.425536
C	4.236026	11.520524	7.549360
H	4.577363	10.531190	7.825738
C	6.596294	9.605854	4.713742
C	7.095953	8.292769	4.968440
C	6.784592	7.577498	6.152787
H	6.149272	8.003559	6.919617
C	7.957353	7.636714	4.052644
H	8.260325	8.098757	3.121579
C	8.459812	6.357373	4.349969
H	9.122713	5.864927	3.652541
C	7.334216	6.295766	6.364362
H	7.104948	5.756765	7.272852
C	3.413777	2.955329	-2.988307
H	4.337932	2.985099	-3.547590
C	3.447754	2.964555	-1.580336
H	4.411737	2.995951	-1.089000
C	1.108542	2.875645	-3.013273
H	0.197148	2.841363	-3.592658
C	1.043794	2.881921	-1.606355
H	0.069525	2.846758	-1.136067

C	2.237579	2.927095	-0.842641
C	2.221475	2.932612	0.584442
C	1.062984	2.876722	2.535911
C	3.330635	2.956093	2.563471
C	4.563148	3.038216	3.280579
C	4.609185	3.056308	4.696057
H	3.701999	3.002548	5.282388
C	5.814701	3.116052	2.618144
H	5.887711	3.104634	1.538068
C	7.005262	3.197753	3.366198
H	7.956346	3.251102	2.855688
C	5.850051	3.145738	5.359247
H	5.891566	3.158942	6.438206
C	-0.191106	2.861126	3.219831
C	-1.422788	2.870474	2.517006
H	-1.461319	2.879611	1.435243
C	-2.639410	2.856165	3.224194
H	-3.574724	2.858065	2.682230
C	-1.549560	2.831213	5.256319
H	-1.622669	2.810001	6.333582
C	-0.283573	2.842183	4.634531
H	0.603596	2.829051	5.254580
C	-3.689221	13.653016	0.530683
H	-3.452619	14.684062	0.309777
C	-4.751784	11.705956	-0.102895
H	-5.362297	11.186325	-0.827640
C	-4.304987	11.029291	1.047764
H	-4.594241	9.994788	1.179888
C	-3.509426	11.708370	2.004881
C	-3.198860	13.059959	1.710745
H	-2.594867	13.660546	2.378774
C	-3.062307	11.071388	3.201296
C	-3.026654	9.249929	4.556595
C	-2.021925	11.175802	5.214092
N	-4.147764	5.177936	5.283968
C	-3.381977	7.887793	4.798834
C	-4.110228	7.129028	3.847323
H	-4.419113	7.549953	2.898754
C	-4.464090	5.796574	4.129351
H	-5.024694	5.223613	3.404325
C	-3.044178	7.218022	6.003001
H	-2.499507	7.719851	6.794346
C	-3.445363	5.879453	6.198289
H	-3.203112	5.371696	7.120530
C	-1.295393	11.921983	6.190407
C	-0.994670	11.387261	7.466470
H	-1.344233	10.407987	7.758343
C	-0.264200	12.147705	8.398786
H	-0.040389	11.730552	9.369676
C	-0.117527	13.930549	6.942875
H	0.229444	14.936841	6.755122
C	-0.837702	13.241472	5.946896
H	-1.024737	13.741108	5.004796
C	3.294032	5.962074	-5.008906
H	4.239773	5.468033	-5.179452
C	0.989366	5.855814	-5.057440
H	0.100866	5.277172	-5.265881
C	0.869095	7.176804	-4.583961
H	-0.123336	7.585400	-4.442732
C	3.271907	7.287282	-4.532073
H	4.215600	7.785206	-4.349450
C	2.032541	7.939406	-4.309237
C	1.960385	9.286461	-3.841801
C	0.728983	11.101441	-3.259771
C	2.994953	11.203023	-3.205403
C	-0.538073	11.739046	-3.100597
C	-1.757253	11.065932	-3.364922
H	-1.775215	10.034060	-3.691484
C	-2.981994	11.745344	-3.214955
H	-3.908988	11.230495	-3.422487
C	-1.920172	13.683360	-2.557247
H	-2.001177	14.713210	-2.239511
C	-0.650314	13.088064	-2.679102
H	0.222350	13.686960	-2.452296
C	4.191053	11.950115	-2.985683
C	4.162248	13.302978	-2.562623
H	3.229739	13.821558	-2.380514
C	5.366738	14.008322	-2.379616
H	5.339797	15.040332	-2.060999
C	5.476521	11.387767	-3.187482
H	5.602038	10.362132	-3.510179
C	6.627270	12.172594	-2.978532
H	7.605751	11.742495	-3.137899
C	10.998289	14.402230	-0.745854
H	10.584408	13.407920	-0.935505
H	11.981270	14.432300	-1.232440
H	11.186911	14.479629	0.332159
C	9.643820	16.353695	-0.146286

H	8.555033	16.337274	-0.044460
H	10.069262	16.073900	0.825736
H	9.928143	17.399347	-0.317668
C	10.676009	16.214102	-2.369517
H	10.777581	17.274748	-2.106261
H	11.706647	15.885080	-2.554021
C	9.950399	16.172779	-3.737428
H	9.762778	17.216888	-4.018967
H	10.689761	15.826901	-4.471087
C	7.528209	16.232186	-4.134282
H	6.830128	16.231765	-3.292403
H	7.801780	17.278953	-4.316544
H	6.973857	15.899806	-5.020881
C	8.833344	14.278612	-4.827508
H	8.040357	14.294972	-5.585590
H	9.782853	14.313152	-5.376061
H	8.791893	13.304671	-4.331513
N	-4.944121	1.048019	5.773606
N	-6.416635	3.451856	6.522676
C	-4.976536	0.456166	4.409560
H	-4.167304	-0.264185	4.236108
H	-5.911249	-0.075230	4.191281
H	-4.880409	1.244780	3.657923
C	-3.831928	0.471090	6.573986
H	-3.229004	-0.251303	6.009625
H	-3.162017	1.267346	6.908320
H	-4.174738	-0.053592	7.474553
C	-6.252797	0.861186	6.466110
H	-6.129235	0.273624	7.384807
H	-6.936755	0.259137	5.854193
C	-7.033798	2.137114	6.865055
H	-8.030223	2.043762	6.414496
H	-7.220972	2.061162	7.943893
C	-7.277854	4.213703	5.579113
H	-7.526739	5.218311	5.943323
H	-6.767840	4.330433	4.618700
H	-8.233842	3.717482	5.369266
C	-6.142896	4.244860	7.750421
H	-6.591803	5.245639	7.721869
H	-6.516348	3.767607	8.665204
H	-5.064875	4.370682	7.879981
N	9.427978	1.683910	5.974603
N	10.581060	4.236555	6.789104
N	10.063456	15.445207	-1.246479
N	8.697021	15.367104	-3.822971
N	-6.774645	14.656543	-1.584095
N	-5.354453	14.679696	-4.133770
N	5.712771	12.107281	4.225982
N	5.773314	10.153876	5.637537
N	6.995417	10.188698	3.562397
N	8.164700	5.694661	5.484930
N	3.008817	13.500675	8.238901
N	2.268985	2.906357	-3.699844
N	3.414286	2.986180	1.214802
N	1.012880	2.908814	1.185612
N	2.188235	2.887829	3.287915
N	-2.711461	2.832246	4.569988
N	7.032185	3.208797	4.714499
N	-4.460041	12.996855	-0.361392
N	-3.423097	9.781543	3.380101
N	-2.320906	9.889908	5.515200
N	-2.331495	11.812842	4.062946
N	0.167524	13.401300	8.151183
N	2.176937	5.254656	-5.275357
N	0.726879	9.815324	-3.675976
N	1.825218	11.845960	-2.996507
N	3.132090	9.923729	-3.619614
N	6.582652	13.463160	-2.587521
N	-3.071530	13.033584	-2.824000
Pd	1.518488	14.351561	9.450997
Pd	-4.908718	13.841962	-2.226395
Pd	8.792651	3.710150	5.751000
Pd	8.316457	14.444722	-1.940743
Pd	2.280753	3.220123	-5.777982
Pd	-4.545643	3.131448	5.547047
C	2.641954	5.168148	7.265194
C	3.255096	6.048166	6.237860
C	2.678246	5.948317	4.872234
C	1.273294	5.911425	4.857915
C	0.655597	5.956449	6.196926
C	1.285867	5.135078	7.249028
H	4.472853	6.220361	3.706893
H	0.766123	6.376406	1.529735
H	4.345676	6.089861	6.266729
C	3.387481	6.146693	3.685135
C	0.582765	6.071834	3.651989
H	-0.434646	5.996263	6.204680
H	3.241974	6.447328	1.559387

C	1.295135	6.245527	2.465033
C	2.695527	6.284667	2.481355
H	-0.504402	6.083562	3.644487
C	3.504318	4.599654	8.375891
C	3.578944	3.065670	8.430564
H	3.129474	4.955310	9.341157
H	4.512483	5.021213	8.294168
H	4.194683	2.740791	9.272678
H	2.589602	2.615385	8.558720
H	3.995517	2.629500	7.517448
C	0.435537	4.432679	8.292818
C	-0.637003	5.278830	8.995013
H	1.080919	4.008793	9.063791
H	-0.043009	3.563656	7.821902
H	-1.267788	4.638012	9.618313
H	-0.189535	6.028554	9.648401
H	-1.274280	5.829785	8.300130
C	0.746994	8.078086	7.995738
C	1.222465	7.796228	6.608130
C	2.698416	7.746217	6.671040
C	3.053094	7.960836	8.127269
H	0.710724	8.358102	5.820468
H	3.276959	8.388789	5.981154
C	1.742822	8.145279	10.320029
C	2.408809	6.985715	11.078304
C	2.292991	9.505824	10.773973
H	0.665269	8.127166	10.511004
C	2.273067	7.179144	12.596450
H	3.466505	6.940634	10.798757
H	1.941563	6.042263	10.775537
C	2.160343	9.685250	12.293101
H	3.347320	9.566152	10.479660
H	1.739304	10.294322	10.237565
C	2.841438	8.533139	13.045040
H	2.782015	6.360534	13.114367
H	1.213140	7.118647	12.877719
H	2.593508	10.645402	12.595279
H	1.096828	9.716162	12.564340
H	2.711152	8.662730	14.123837
H	3.921600	8.559325	12.849455
N	1.860192	8.003787	8.853883
O	4.173682	8.030980	8.599580
O	-0.393864	8.277809	8.369972

Table5_1c_TSiii_TSi-ii_0wat

Property	Value
Charge	0
Electronic Energy, BS1 (a.u.)	-1137.528179
Thermal and entropic correction, BS1 (a.u.)	2.808207
Electronic Energy, BS2 (a.u.)	-1137.903063
Number of Imaginary Frequencies	0
Imaginary frequencies (cm-1)	None

Molecular Geometry in Cartesian Coordinates

C	8.432493	0.982919	6.930131
H	8.808976	0.549971	7.865451
H	7.653469	1.699092	7.206629
H	7.947475	0.163319	6.385182
C	9.632590	1.032609	4.794578
H	8.926321	0.208711	4.631969
H	9.457998	1.772719	4.008777
H	10.633014	0.617696	4.619133
C	10.789046	1.668381	6.863719
H	11.565027	1.151547	6.284640
H	10.717658	1.087417	7.792175
C	11.379552	3.045038	7.254738
H	12.384709	3.088197	6.816074
H	11.561579	3.005428	8.336369
C	11.370317	5.157128	6.007218
H	10.905331	5.206698	5.019067
H	11.437854	6.182989	6.390437
H	12.402689	4.819304	5.852309
C	10.134373	4.977577	8.114765
H	9.043932	4.940521	8.185873
H	10.525105	4.542139	9.042952
H	10.430828	6.033981	8.122306
N	3.178583	15.046710	10.491227
N	0.297791	15.325872	10.868777
C	3.946635	13.871935	10.981577
H	3.402579	12.950789	10.756157
H	4.940675	13.789747	10.524463
H	4.107301	13.883720	12.066910

C	3.985244	15.847581	9.532171
H	3.452794	15.943600	8.582596
H	4.184415	16.868022	9.883056
H	4.964222	15.400160	9.319420
C	2.715881	15.893331	11.629864
H	3.115356	15.520920	12.581907
H	3.119083	16.911051	11.551095
C	1.187568	16.044411	11.827344
H	0.978552	17.121899	11.823314
H	0.978368	15.735830	12.859676
C	-0.562630	16.282120	10.122124
H	-1.631967	16.051865	10.208302
H	-0.444511	17.320637	10.455878
H	-0.308440	16.263006	9.058417
C	-0.526394	14.305740	11.570095
H	-0.244273	13.303464	11.235587
H	-0.397943	14.321628	12.659567
H	-1.601593	14.423694	11.385253
N	2.528537	1.180956	-6.405215
N	2.325528	3.715539	-7.838139
C	3.780115	0.632859	-5.817530
H	3.596586	-0.176455	-5.099739
H	4.468807	0.226788	-6.568987
H	4.322021	1.422208	-5.289143
C	1.337014	0.462110	-5.881254
H	1.593191	-0.314422	-5.149599
H	0.663769	1.168128	-5.387167
H	0.752941	-0.034687	-6.665987
C	2.571822	1.129899	-7.895958
H	1.773366	0.486302	-8.287000
H	3.495634	0.651182	-8.245181
C	2.458658	2.474409	-8.656525
H	3.333593	2.532113	-9.316678
H	1.610980	2.368723	-9.345677
C	1.024137	4.386524	-8.097717
H	1.136214	5.437149	-8.393628
H	0.440461	3.903821	-8.891535
H	0.405700	4.362970	-7.196109
C	3.461431	4.641830	-8.091566
H	3.135035	5.646103	-8.388986
H	4.067905	4.748211	-7.188045
H	4.135577	4.291696	-8.883263
C	-6.595549	15.907995	-0.944264
H	-7.237577	15.715405	-0.075495
H	-5.564482	15.961577	-0.585127
H	-6.855969	16.911873	-1.302485
C	-7.712631	13.831714	-1.609026
H	-8.160510	14.018481	-0.624838
H	-8.548070	13.754026	-2.316052
H	-7.238663	12.846591	-1.570716
C	-7.053889	15.469396	-3.315506
H	-7.258318	16.542528	-3.209335
H	-7.992691	15.056448	-3.706372
C	-6.007883	15.337216	-4.450107
H	-6.538797	14.906474	-5.308672
H	-5.766702	16.358860	-4.770791
C	-4.666593	13.364677	-5.024097
H	-3.717323	13.311057	-5.571485
H	-4.752534	12.455862	-4.422917
H	-5.459801	13.314485	-5.780576
C	-3.559759	15.424094	-4.290858
H	-2.812430	15.003407	-4.975153
H	-3.787554	16.429549	-4.666367
H	-3.078385	15.554203	-3.317558
N	8.093155	13.543021	-0.026292
C	7.260132	14.133133	0.856256
H	6.961967	15.149527	0.642206
C	6.784161	13.495033	2.018964
H	6.130848	14.047238	2.681495
C	7.177927	12.164415	2.307278
C	8.048820	11.557038	1.367335
H	8.408891	10.544538	1.497509
C	8.471957	12.274762	0.232276
H	9.138083	11.808061	-0.479116
C	6.730536	11.476969	3.475792
C	5.516363	11.426975	5.391666
C	4.555599	12.034697	6.258160
C	4.043932	13.334776	6.014520
H	4.369837	13.922811	5.166229
C	3.093238	13.895607	6.889056
H	2.702277	14.884778	6.697524
C	3.099315	12.018944	8.228218
H	2.712437	11.515719	9.103271
C	4.049232	11.376083	7.407670
H	4.382546	10.383904	7.682554
C	6.767083	9.631359	4.793679
C	7.221533	8.297333	5.028365
C	6.883987	7.577019	6.202299

H	6.274874	8.019709	6.979245
C	8.049542	7.617085	4.098897
H	8.364659	8.079876	3.172136
C	8.490615	6.308263	4.371220
H	9.122721	5.794133	3.660846
C	7.375763	6.268905	6.391885
H	7.127561	5.724559	7.291591
C	3.534370	2.858340	-2.999370
H	4.457318	2.928545	-3.557463
C	3.566081	2.900950	-1.591398
H	4.525612	3.007015	-1.101500
C	1.242974	2.630798	-3.024189
H	0.336339	2.519174	-3.601407
C	1.175121	2.661352	-1.617534
H	0.205566	2.565843	-1.146314
C	2.358648	2.819766	-0.853216
C	2.333037	2.923674	0.571337
C	1.166940	3.078518	2.513288
C	3.435470	3.067798	2.548082
C	4.660448	3.083225	3.280966
C	4.689388	3.023691	4.696474
H	3.779177	2.944604	5.277129
C	5.921645	3.144363	2.637765
H	6.007566	3.178308	1.558954
C	7.103043	3.162712	3.405075
H	8.062234	3.211926	2.909010
C	5.922253	3.040830	5.378054
H	5.939446	2.984399	6.457014
C	-0.077572	3.183655	3.205103
C	-1.319396	3.195719	2.521009
H	-1.372762	3.123349	1.442457
C	-2.522093	3.291586	3.248261
H	-3.468775	3.298358	2.726758
C	-1.394603	3.372575	5.260140
H	-1.444274	3.444877	6.337093
C	-0.144143	3.282163	4.618235
H	0.749918	3.280162	5.228164
C	-4.206843	13.902587	0.814048
H	-3.988633	14.939994	0.605114
C	-5.144503	11.919679	0.121381
H	-5.680178	11.374066	-0.642539
C	-4.793238	11.274704	1.322826
H	-5.074868	10.237399	1.449557
C	-4.106379	11.989959	2.335748
C	-3.823462	13.348465	2.050736
H	-3.326992	13.987096	2.769858
C	-3.728464	11.379105	3.569139
C	-3.670351	9.550304	4.908883
C	-2.714330	11.489193	5.595465
N	-4.372219	5.361723	5.494389
C	-3.897087	8.153731	5.100355
C	-4.537844	7.354672	4.119515
H	-4.894076	7.775970	3.188226
C	-4.752868	5.983379	4.359645
H	-5.258660	5.379302	3.619679
C	-3.492860	7.483010	6.282031
H	-3.001018	8.009913	7.090050
C	-3.747066	6.105090	6.430944
H	-3.448349	5.599619	7.337610
C	-1.892587	12.188195	6.531227
C	-1.470087	11.590769	7.744980
H	-1.778108	10.590505	8.020021
C	-0.618704	12.293940	8.617407
H	-0.297242	11.830128	9.538382
C	-0.573259	14.128284	7.226039
H	-0.219678	15.132924	7.042676
C	-1.419749	13.502269	6.288847
H	-1.696513	14.046859	5.395729
C	3.370209	5.905562	-4.807922
H	4.317003	5.413943	-4.982999
C	1.066331	5.809883	-4.894500
H	0.179841	5.241045	-5.136392
C	0.944066	7.118910	-4.383638
H	-0.047467	7.533288	-4.255430
C	3.345184	7.215980	-4.292179
H	4.289790	7.702490	-4.083840
C	2.105881	7.864568	-4.057530
C	2.036511	9.190176	-3.528994
C	0.818487	10.972747	-2.824365
C	3.083969	11.099407	-2.883056
C	-0.444737	11.598566	-2.593957
C	-1.669811	10.929733	-2.841980
H	-1.694620	9.910495	-3.205228
C	-2.891478	11.604224	-2.648109
H	-3.825058	11.098284	-2.848883
C	-1.816080	13.527760	-1.962173
H	-1.894595	14.548434	-1.614837
C	-0.548604	12.935253	-2.128958

H	0.328829	13.531308	-1.913308
C	4.282757	11.861537	-2.738726
C	4.264595	13.223079	-2.343719
H	3.337368	13.742320	-2.140097
C	5.472542	13.938620	-2.228436
H	5.455202	14.978427	-1.934566
C	5.561995	11.304913	-2.991373
H	5.681540	10.274354	-3.300444
C	6.716673	12.098390	-2.844489
H	7.689440	11.669005	-3.037296
C	11.182078	14.330928	-0.868136
H	10.747215	13.340558	-1.030375
H	12.137505	14.348372	-1.407379
H	11.429936	14.410603	0.197731
C	9.884839	16.300748	-0.205963
H	8.803519	16.296754	-0.042459
H	10.361553	16.021468	0.742059
H	10.170853	17.342363	-0.398380
C	10.790035	16.139266	-2.481509
H	10.888836	17.205622	-2.241280
H	11.816531	15.818503	-2.700697
C	10.013713	16.062923	-3.819604
H	9.802200	17.098556	-4.115055
H	10.730328	15.711422	-4.572906
C	7.584241	16.073777	-4.168940
H	6.901651	16.101640	-3.314820
H	7.840178	17.115044	-4.401556
H	7.018483	15.696720	-5.030157
C	8.901185	14.107178	-4.800766
H	8.090486	14.076415	-5.539447
H	9.837046	14.131888	-5.372737
H	8.888503	13.156449	-4.260478
N	-4.396649	1.133526	5.757254
N	-6.254129	3.187620	6.672244
C	-4.301758	0.601250	4.371550
H	-3.356066	0.079771	4.178080
H	-5.099672	-0.111348	4.127442
H	-4.382003	1.417260	3.649272
C	-3.206361	0.743541	6.558393
H	-2.468470	0.174565	5.979053
H	-2.706707	1.637125	6.942756
H	-3.456483	0.124737	7.429284
C	-5.655490	0.673891	6.413983
H	-5.434411	0.057269	7.294589
H	-6.221093	0.003704	5.754039
C	-6.649189	1.764242	6.883813
H	-7.607547	1.544950	6.395756
H	-6.840061	1.568038	7.946742
C	-7.259948	3.897651	5.837195
H	-7.644552	4.807984	6.313939
H	-6.816572	4.190148	4.881225
H	-8.137358	3.282173	5.602069
C	-6.055924	3.883173	7.971438
H	-6.659539	4.794596	8.064986
H	-6.305309	3.258525	8.838374
H	-5.006673	4.169969	8.084208
N	9.487683	1.664034	6.133754
N	10.586812	4.257222	6.895331
N	10.232798	15.381881	-1.322490
N	8.770397	15.237707	-3.843290
N	-6.712761	14.862821	-1.995542
N	-4.765698	14.565088	-4.151461
N	5.881536	12.134411	4.298278
N	5.940724	10.181895	5.714786
N	7.194345	10.222501	3.657962
N	8.170154	5.642672	5.498589
N	2.629756	13.260286	7.984046
N	2.397512	2.729361	-3.715173
N	3.522872	3.052272	1.199724
N	1.118554	2.969935	1.165940
N	2.290371	3.107490	3.264355
N	-2.566883	3.370143	4.593997
N	7.116108	3.110296	4.753624
N	-4.859035	13.211263	-0.144441
N	-4.038420	10.072680	3.718930
N	-3.063063	10.220283	5.909466
N	-3.020638	12.130130	4.442606
N	-0.171312	13.542682	8.374030
N	2.255527	5.209075	-5.109067
N	0.807371	9.694663	-3.270162
N	1.920180	11.714378	-2.573327
N	3.209914	9.823545	-3.310097
N	6.682255	13.395738	-2.475284
N	-2.970328	12.882601	-2.225651
Pd	1.484727	14.292243	9.425253
Pd	-4.821174	13.880457	-2.128970
Pd	8.839562	3.673384	5.819495
Pd	8.438172	14.398681	-1.912544

Pd	2.371418	3.210658	-5.765472
Pd	-4.396969	3.266647	5.624339
C	3.090069	5.790704	5.547392
C	3.165429	6.556041	4.339128
C	1.962371	6.637444	3.526629
C	0.737001	6.610125	4.228925
C	0.830488	6.549858	5.672764
C	1.908168	5.804607	6.238867
H	2.890383	6.948151	1.609302
H	3.994521	5.392876	5.995608
H	4.117274	6.628689	3.818681
C	1.946472	6.846395	2.138727
C	-0.466724	6.679725	3.508886
H	-0.087266	6.663469	6.243107
H	1.831603	5.408310	7.245556
C	-0.484596	6.789726	2.120826
C	0.750627	6.936472	1.428838
H	-1.408611	6.617521	4.050199
C	-1.820966	6.754668	1.399911
C	-2.502616	5.376667	1.356700
H	-1.657457	7.088173	0.368580
H	-2.508621	7.482596	1.840755
H	-3.405570	5.420058	0.741826
H	-1.828587	4.626150	0.937954
H	-2.793281	5.036506	2.355102
C	0.817855	7.182212	-0.067769
C	1.266301	8.606469	-0.408271
H	-0.176606	7.007199	-0.494248
H	1.490097	6.467945	-0.548106
H	1.319787	8.761795	-1.487253
H	0.560529	9.335616	-0.001068
H	2.257217	8.801072	0.009952
C	3.031081	9.355656	4.130980
C	3.071979	8.417464	5.295435
C	1.829594	8.516250	5.960888
C	1.000467	9.498333	5.206429
H	4.014665	8.294379	5.808000
H	1.693640	8.452151	7.030279
C	1.340055	10.863155	3.053567
C	-0.042780	10.560625	2.459349
C	1.429919	12.309823	3.567370
H	2.093727	10.736738	2.269816
C	-0.381762	11.560085	1.343163
H	-0.790844	10.619160	3.255363
H	-0.055295	9.536607	2.080373
C	1.085069	13.303552	2.448124
H	0.739896	12.429565	4.412673
H	2.444196	12.494177	3.935625
C	-0.292064	13.009182	1.840415
H	-1.382689	11.345377	0.947174
H	0.319557	11.422101	0.507231
H	1.122718	14.329702	2.824062
H	1.848792	13.233863	1.662673
H	-0.500023	13.712496	1.026500
H	-1.057679	13.176674	2.608762
N	1.741123	9.895572	4.084339
O	-0.107657	9.913364	5.496278
O	3.924783	9.619368	3.347183

Table5_1c_TSiii_DG_0wat

Property	Value
Charge	0
Electronic Energy, BS1 (a.u.)	-1137.573274
Thermal and entropic correction, BS1 (a.u.)	2.814186
Electronic Energy, BS2 (a.u.)	-1137.945111
Number of Imaginary Frequencies	0
Imaginary frequencies (cm-1)	None

Molecular Geometry in Cartesian Coordinates

C	8.368714	1.015229	6.897359
H	8.748009	0.545438	7.813499
H	7.622549	1.751468	7.208663
H	7.843606	0.227325	6.343003
C	9.533721	1.068866	4.742346
H	8.796335	0.273814	4.574958
H	9.372864	1.830838	3.974424
H	10.516149	0.623053	4.542582
C	10.744431	1.624638	6.803669
H	11.496746	1.104116	6.197342
H	10.671397	1.018858	7.716038
C	11.377810	2.972604	7.226079
H	12.386821	2.989110	6.794431

H	11.551461	2.906798	8.307804
C	11.438680	5.091108	5.990512
H	10.958948	5.171737	5.011430
H	11.559982	6.108567	6.382741
H	12.451574	4.708397	5.813167
C	10.222300	4.949192	8.112116
H	9.131980	4.958672	8.194395
H	10.604430	4.492228	9.033502
H	10.562460	5.992309	8.122199
N	3.122207	14.979599	10.424163
N	0.249009	15.272925	10.840719
C	3.877471	13.804074	10.932069
H	3.316028	12.887228	10.733147
H	4.864855	13.696207	10.466087
H	4.050360	13.836985	12.015063
C	3.932581	15.750125	9.443417
H	3.397300	15.830281	8.493797
H	4.144279	16.775985	9.770407
H	4.905866	15.287694	9.236694
C	2.679497	15.854424	11.549311
H	3.092897	15.502891	12.503330
H	3.083607	16.869026	11.440718
C	1.154715	16.011663	11.768431
H	0.946423	17.089030	11.744734
H	0.961983	15.725168	12.810286
C	-0.635859	16.212790	10.101527
H	-1.700911	15.972956	10.211575
H	-0.521459	17.255875	10.422040
H	-0.403593	16.184731	9.033047
C	-0.551184	14.255590	11.572875
H	-0.274504	13.251835	11.237797
H	-0.392532	14.281124	12.658164
H	-1.631544	14.367548	11.416601
N	2.404860	1.242748	-6.098408
N	2.406485	3.592564	-7.828440
C	3.615406	0.701491	-5.425559
H	3.376955	0.006477	-4.610692
H	4.282130	0.158839	-6.107339
H	4.202213	1.519940	-4.999006
C	1.166763	0.661248	-5.515322
H	1.367666	-0.027538	-4.685250
H	0.524378	1.459331	-5.132670
H	0.569461	0.098774	-6.243775
C	2.463082	1.008091	-7.570984
H	1.621801	0.385230	-7.901013
H	3.351181	0.421597	-7.839100
C	2.469320	2.255553	-8.488944
H	3.363290	2.171986	-9.120188
H	1.634552	2.126882	-9.189817
C	1.167616	4.319022	-8.214674
H	1.367344	5.317875	-8.622473
H	0.576198	3.790680	-8.972885
H	0.519910	4.443708	-7.342344
C	3.615729	4.398550	-8.144235
H	3.375654	5.379699	-8.572827
H	4.197587	4.570787	-7.234308
H	4.287490	3.907392	-8.859310
C	-6.548345	15.739448	-0.639228
H	-7.076041	15.434369	0.273103
H	-5.486517	15.822246	-0.392925
H	-6.897144	16.753657	-0.871077
C	-7.636530	13.669397	-1.369311
H	-7.976724	13.742447	-0.328661
H	-8.543368	13.610073	-1.983981
H	-7.118193	12.712530	-1.478550
C	-7.248218	15.483141	-2.977734
H	-7.479599	16.532668	-2.754814
H	-8.209273	15.059802	-3.296727
C	-6.336093	15.495416	-4.229604
H	-6.942261	15.102322	-5.055820
H	-6.180969	16.550078	-4.490844
C	-4.976470	13.653964	-5.114430
H	-4.095500	13.699926	-5.766826
H	-4.945272	12.697324	-4.586280
H	-5.848580	13.616571	-5.779274
C	-3.891719	15.706415	-4.332216
H	-3.211164	15.387776	-5.131762
H	-4.206716	16.726265	-4.586158
H	-3.305364	15.777969	-3.411684
N	8.066385	13.615033	-0.025108
C	7.178472	14.166312	0.828227
H	6.859952	15.176889	0.615809
C	6.672761	13.495879	1.959696
H	5.975034	14.018331	2.600757
C	7.101869	12.176677	2.251088
C	8.029986	11.609915	1.341385
H	8.418160	10.608249	1.475606
C	8.473791	12.355280	0.232133

H	9.181610	11.918875	-0.457923
C	6.637397	11.464101	3.397453
C	5.397293	11.387509	5.295795
C	4.430708	11.980729	6.165294
C	3.870458	13.257397	5.905310
H	4.152705	13.834977	5.034463
C	2.937059	13.814071	6.801235
H	2.516066	14.789843	6.604817
C	3.036396	11.968878	8.179517
H	2.690301	11.474772	9.076192
C	3.976912	11.333825	7.342895
H	4.346269	10.357989	7.629175
C	6.730667	9.637062	4.738106
C	7.229585	8.324823	5.002466
C	6.917628	7.623021	6.194685
H	6.300797	8.065837	6.965293
C	8.076316	7.648627	4.087823
H	8.374087	8.100125	3.149860
C	8.556178	6.359755	4.390741
H	9.203788	5.849600	3.691711
C	7.446068	6.334609	6.413319
H	7.216523	5.805421	7.326936
C	3.462519	3.176355	-2.897953
H	4.390527	3.205914	-3.451298
C	3.485708	3.187608	-1.490048
H	4.447935	3.218876	-0.994837
C	1.157441	3.085902	-2.938532
H	0.251253	3.041772	-3.525512
C	1.082627	3.094279	-1.530833
H	0.106746	3.047992	-1.065084
C	2.271210	3.141877	-0.758966
C	2.250421	3.140478	0.669047
C	1.099937	3.115050	2.626247
C	3.368359	3.162239	2.643861
C	4.604262	3.184590	3.358443
C	4.659158	3.102393	4.772045
H	3.759845	2.996625	5.365237
C	5.852872	3.282528	2.694119
H	5.921272	3.336281	1.614949
C	7.047867	3.303093	3.438661
H	7.997412	3.374108	2.926949
C	5.904340	3.133625	5.431859
H	5.942569	3.065478	6.509464
C	-0.144339	3.122123	3.326556
C	-1.383015	3.014716	2.646211
H	-1.432487	2.912942	1.569945
C	-2.588924	3.014544	3.372597
H	-3.531277	2.930720	2.849965
C	-1.472796	3.227750	5.381554
H	-1.529470	3.310871	6.457364
C	-0.217481	3.231328	4.738944
H	0.675156	3.317224	5.345301
C	-3.929836	13.757189	0.685541
H	-3.709017	14.798434	0.499680
C	-4.881678	11.792377	-0.053780
H	-5.422435	11.269522	-0.830329
C	-4.519964	11.113851	1.127765
H	-4.803443	10.074889	1.234442
C	-3.826938	11.802089	2.154809
C	-3.531453	13.165207	1.898744
H	-3.018698	13.783286	2.624356
C	-3.465473	11.171255	3.383485
C	-3.486863	9.365690	4.755552
C	-2.541058	11.309054	5.451291
N	-4.373617	5.234470	5.494674
C	-3.792918	7.991900	5.000113
C	-4.432498	7.174232	4.034076
H	-4.738941	7.568134	3.073405
C	-4.698586	5.819647	4.322722
H	-5.194425	5.199320	3.589045
C	-3.459681	7.359695	6.225362
H	-2.981503	7.900281	7.032338
C	-3.761305	5.998466	6.421542
H	-3.508932	5.522971	7.357818
C	-1.813799	12.047984	6.432064
C	-1.446403	11.479870	7.676626
H	-1.732455	10.471355	7.943435
C	-0.676688	12.225266	8.589479
H	-0.395128	11.782851	9.533766
C	-0.622012	14.045124	7.185226
H	-0.300322	15.061860	7.011286
C	-1.382891	13.378264	6.205612
H	-1.624880	13.904010	5.291603
C	3.399175	6.090930	-5.018295
H	4.341559	5.589421	-5.187658
C	1.095986	6.014623	-5.098098
H	0.203500	5.453060	-5.334652
C	0.986078	7.329724	-4.605999

H	-0.002991	7.752090	-4.485813
C	3.387922	7.411331	-4.526350
H	4.335922	7.896723	-4.331149
C	2.154540	8.074107	-4.304311
C	2.090757	9.410874	-3.806067
C	0.864167	11.192381	-3.114531
C	3.130324	11.301303	-3.101653
C	-0.406280	11.810639	-2.909363
C	-1.619422	11.143628	-3.215122
H	-1.630768	10.137059	-3.612606
C	-2.850521	11.801571	-3.032822
H	-3.773267	11.292698	-3.273101
C	-1.807476	13.712299	-2.265160
H	-1.901970	14.724469	-1.897847
C	-0.530879	13.133137	-2.412288
H	0.337706	13.727264	-2.158971
C	4.329962	12.047830	-2.899428
C	4.310972	13.392555	-2.451227
H	3.382608	13.910924	-2.250255
C	5.520343	14.094501	-2.281774
H	5.501977	15.123085	-1.951112
C	5.611402	11.491302	-3.141615
H	5.732177	10.472959	-3.488613
C	6.767428	12.269552	-2.936480
H	7.742076	11.840978	-3.122134
C	11.176630	14.461464	-0.713401
H	10.768314	13.469441	-0.925696
H	12.156896	14.509785	-1.203961
H	11.370332	14.513852	0.365280
C	9.822945	16.397004	-0.062913
H	8.735403	16.375839	0.051318
H	10.260075	16.100367	0.898724
H	10.103014	17.446435	-0.217706
C	10.830377	16.301637	-2.299701
H	10.919153	17.360248	-2.023888
H	11.864448	15.988183	-2.492242
C	10.101329	16.266142	-3.665662
H	9.903463	17.310965	-3.937330
H	10.842050	15.933795	-4.404220
C	7.679893	16.304773	-4.066556
H	6.981057	16.297796	-3.225496
H	7.944762	17.354035	-4.247480
H	7.129325	15.969164	-4.954251
C	9.003274	14.364845	-4.762920
H	8.212466	14.377422	-5.523403
H	9.953926	14.408865	-5.308794
H	8.968460	13.388943	-4.270194
N	-4.569948	1.027794	5.976711
N	-6.357322	3.198659	6.749663
C	-4.493789	0.415472	4.623093
H	-3.574276	-0.162148	4.466341
H	-5.324936	-0.268662	4.410882
H	-4.528780	1.193409	3.856673
C	-3.396318	0.638025	6.802579
H	-2.688146	-0.003280	6.263133
H	-2.852330	1.531300	7.123321
H	-3.671819	0.092810	7.714057
C	-5.846924	0.655386	6.653654
H	-5.651561	0.102732	7.581630
H	-6.424722	-0.048305	6.040854
C	-6.815293	1.805348	7.023751
H	-7.764576	1.594414	6.514762
H	-7.048794	1.676450	8.088459
C	-7.318318	3.908428	5.863651
H	-7.672917	4.855092	6.289969
H	-6.845729	4.138650	4.904695
H	-8.215938	3.318217	5.640234
C	-6.150537	3.948119	8.016911
H	-6.707785	4.892634	8.051384
H	-6.450435	3.381126	8.907060
H	-5.090507	4.185311	8.140372
N	9.431986	1.677533	6.095918
N	10.630525	4.218168	6.883394
N	10.232902	15.510187	-1.184346
N	8.855691	15.449074	-3.755396
N	-6.734379	14.784190	-1.763568
N	-5.033012	14.773274	-4.136607
N	5.728212	12.083327	4.185073
N	5.873880	10.169371	5.641789
N	7.148202	10.230705	3.599461
N	8.256286	5.710127	5.533920
N	2.528407	13.192550	7.925798
N	2.323240	3.120433	-3.616736
N	3.443584	3.220235	1.296574
N	1.041940	3.108226	1.273673
N	2.229115	3.139254	3.371457
N	-2.640840	3.115409	4.715536
N	7.084882	3.228793	4.784780

N	-4.600571	13.093251	-0.277387
N	-3.783864	9.863744	3.532521
N	-2.872238	10.033815	5.754411
N	-2.799440	11.931551	4.279619
N	-0.259591	13.487814	8.359882
N	2.277436	5.398859	-5.307051
N	0.860278	9.925763	-3.585887
N	1.962260	11.917977	-2.806914
N	3.262252	10.034795	-3.555641
N	6.732288	13.552348	-2.520426
N	-2.950071	13.065636	-2.575622
Pd	1.410577	14.229886	9.385172
Pd	-4.824822	13.926555	-2.189312
Pd	8.846677	3.713182	5.828867
Pd	8.479682	14.517266	-1.875379
Pd	2.356482	3.340728	-5.711792
Pd	-4.484557	3.147288	5.727775
C	2.850780	5.703922	5.963615
C	3.087606	6.385526	4.678912
C	1.908392	6.415605	3.781099
C	0.692493	6.695434	4.422612
C	0.783348	6.895419	5.878077
C	1.685403	5.981203	6.580799
H	2.917497	6.213396	1.896279
H	3.654562	5.184809	6.474274
H	4.036929	6.191642	4.181309
C	1.963830	6.379502	2.387678
C	-0.448494	6.930889	3.651283
H	-0.131799	7.197215	6.384083
H	1.487816	5.689920	7.610191
C	-0.410123	6.877476	2.257598
C	0.817755	6.583529	1.614391
H	-1.375848	7.189990	4.149502
C	-1.674647	7.169988	1.476768
C	-2.507968	5.917564	1.172932
H	-1.430096	7.672004	0.538602
H	-2.280786	7.882647	2.044310
H	-3.393675	6.160611	0.581594
H	-1.926187	5.180251	0.612730
H	-2.834877	5.448104	2.106178
C	0.949611	6.564189	0.105422
C	1.191191	7.959223	-0.495341
H	0.060580	6.115949	-0.350725
H	1.795718	5.925985	-0.168784
H	1.326450	7.881161	-1.576487
H	0.348641	8.630754	-0.311514
H	2.088070	8.413828	-0.065006
C	3.212172	8.993823	3.973991
C	3.193342	8.160833	5.235619
C	1.958530	8.532923	5.939917
C	1.282705	9.572252	5.102219
H	4.141470	8.176523	5.792624
H	1.954469	8.725992	7.017257
C	1.631088	10.546588	2.760697
C	0.188497	10.303977	2.283749
C	1.866963	12.028011	3.098753
H	2.323220	10.249446	1.967048
C	-0.155656	11.210499	1.091904
H	-0.493753	10.505888	3.114189
H	0.074955	9.253715	2.019893
C	1.498467	12.932033	1.910216
H	1.254573	12.284192	3.969457
H	2.918303	12.170269	3.369833
C	0.056520	12.687528	1.446006
H	-1.189530	11.022407	0.776616
H	0.484542	10.950357	0.236714
H	1.639763	13.982607	2.178319
H	2.181501	12.726224	1.074709
H	-0.178221	13.331516	0.591369
H	-0.630812	12.968337	2.255344
N	1.994772	9.684392	3.899997
O	0.277589	10.205488	5.373856
O	4.087041	9.038821	3.131733

Table5_1c_TSiv_DDG_0wat

Property	Value
Charge	0
Electronic Energy, BS1 (a.u.)	-1137.560643
Thermal and entropic correction, BS1 (a.u.)	2.802407
Electronic Energy, BS2 (a.u.)	-1137.941214
Number of Imaginary Frequencies	0
Imaginary frequencies (cm-1)	None

Molecular Geometry in Cartesian Coordinates

C	8.064188	1.014136	6.876055
H	8.440132	0.478556	7.756739
H	7.411547	1.808447	7.248676
H	7.438000	0.300881	6.325510
C	9.127038	1.029564	4.667837
H	8.302245	0.323311	4.511051
H	9.019470	1.828048	3.928522
H	10.046191	0.487204	4.412703
C	10.480304	1.396768	6.680219
H	11.166079	0.871602	6.003054
H	10.397731	0.729424	7.547829
C	11.227005	2.659167	7.176283
H	12.222696	2.633478	6.715177
H	11.423295	2.500395	8.244417
C	11.435421	4.871534	6.137353
H	10.970607	5.068395	5.168012
H	11.619038	5.841480	6.616384
H	12.423263	4.441261	5.930095
C	10.206834	4.622674	8.241816
H	9.119268	4.709158	8.322456
H	10.544480	4.053724	9.117039
H	10.626320	5.630853	8.348860
N	2.742640	15.324369	10.729640
N	-0.156503	15.097991	10.485255
C	3.597017	14.311353	11.404049
H	3.290273	13.305404	11.103894
H	4.661276	14.418699	11.159156
H	3.528696	14.349694	12.498317
C	3.568103	16.261356	9.921322
H	3.250955	16.233236	8.874987
H	3.481125	17.305263	10.247743
H	4.638172	16.020134	9.947411
C	1.911002	16.067033	11.721757
H	2.159376	15.762577	12.746573
H	2.140940	17.140026	11.699526
C	0.372730	15.947913	11.591540
H	-0.012083	16.972990	11.515620
H	0.006036	15.596565	12.564571
C	-0.969310	15.904995	9.536505
H	-1.978452	15.501020	9.387083
H	-1.100111	16.946036	9.857330
H	-0.480779	15.936872	8.558570
C	-0.944790	13.957583	11.022147
H	-0.440029	13.015911	10.792576
H	-1.067383	13.992121	12.111987
H	-1.956066	13.900729	10.600388
N	2.477415	1.162850	-6.013159
N	2.444572	3.414470	-7.869599
C	3.677899	0.693904	-5.271058
H	3.429278	0.039708	-4.426112
H	4.380357	0.131279	-5.898667
H	4.230068	1.549875	-4.872860
C	1.235663	0.575906	-5.442901
H	1.425236	-0.056544	-4.566578
H	0.553669	1.372701	-5.133222
H	0.684872	-0.047385	-6.158468
C	2.594745	0.851602	-7.467798
H	1.794743	0.171338	-7.787042
H	3.517472	0.295504	-7.677009
C	2.568526	2.046225	-8.453254
H	3.475594	1.962603	-9.065574
H	1.751162	1.847216	-9.158182
C	1.189092	4.073277	-8.317305
H	1.361922	5.051838	-8.782567
H	0.627568	3.479556	-9.049338
H	0.524892	4.228656	-7.462271
C	3.631050	4.245133	-8.208554
H	3.365109	5.190576	-8.697694
H	4.187523	4.491057	-7.299897
H	4.335088	3.739134	-8.881014
C	-6.468914	15.446170	-0.408732
H	-6.895669	15.042270	0.517827
H	-5.390324	15.541218	-0.258821
H	-6.861918	16.465664	-0.509580
C	-7.577189	13.417243	-1.221177
H	-7.808086	13.380168	-0.149148
H	-8.540598	13.386764	-1.745215
H	-7.051341	12.492679	-1.476196
C	-7.394326	15.383411	-2.680115
H	-7.586603	16.413333	-2.353271
H	-8.390986	14.985852	-2.910829
C	-6.641849	15.486051	-4.029910
H	-7.346988	15.149682	-4.800824
H	-6.513104	16.556556	-4.234710
C	-5.430247	13.676298	-5.163316
H	-4.654150	13.750093	-5.935449

H	-5.319576	12.702398	-4.677974
H	-6.391047	13.656451	-5.692606
C	-4.235875	15.699877	-4.468093
H	-3.678493	15.412732	-5.368561
H	-4.577967	16.728983	-4.634510
H	-3.522109	15.735580	-3.640168
N	8.080359	13.595630	-0.012145
C	7.254839	14.177239	0.882056
H	6.905791	15.173644	0.651844
C	6.844439	13.551112	2.074924
H	6.183498	14.092680	2.739476
C	7.299674	12.243719	2.381601
C	8.159672	11.643851	1.427502
H	8.559563	10.648190	1.570574
C	8.515875	12.348567	0.261288
H	9.171469	11.888621	-0.464010
C	6.914126	11.571474	3.580234
C	5.761948	11.550093	5.536064
C	4.864368	12.189704	6.444130
C	4.344917	13.484661	6.191994
H	4.613194	14.042257	5.303708
C	3.458858	14.078926	7.110025
H	3.066934	15.066599	6.913011
C	3.544870	12.245352	8.505670
H	3.226546	11.775296	9.424599
C	4.436445	11.571568	7.646198
H	4.791865	10.589848	7.931330
C	7.002286	9.748221	4.929677
C	7.459194	8.417680	5.176358
C	7.138669	7.719896	6.367386
H	6.556838	8.183648	7.152318
C	8.261365	7.714119	4.242690
H	8.569282	8.163137	3.306909
C	8.678109	6.398320	4.522656
H	9.290546	5.866600	3.808300
C	7.598005	6.402304	6.560273
H	7.349233	5.876591	7.470260
C	3.419817	3.283013	-2.907758
H	4.348645	3.392716	-3.449499
C	3.426998	3.252254	-1.500336
H	4.379216	3.333451	-0.991979
C	1.125475	3.040161	-2.981097
H	0.230730	2.961760	-3.581725
C	1.036098	2.994347	-1.575080
H	0.061102	2.864528	-1.124059
C	2.210600	3.098603	-0.787110
C	2.176651	3.047589	0.639592
C	1.016336	2.856794	2.580930
C	3.274189	3.085689	2.625399
C	4.498298	3.200695	3.352412
C	4.546490	3.114857	4.766335
H	3.651256	2.942190	5.349514
C	5.741537	3.402667	2.699892
H	5.814273	3.470085	1.622042
C	6.926144	3.507279	3.454211
H	7.873155	3.654568	2.953976
C	5.779292	3.238816	5.438779
H	5.813170	3.176792	6.516433
C	-0.232116	2.763430	3.268595
C	-1.464001	2.663630	2.575899
H	-1.504133	2.612873	1.496110
C	-2.675635	2.618935	3.291626
H	-3.611313	2.545458	2.755676
C	-1.578185	2.732524	5.314236
H	-1.638780	2.751199	6.392719
C	-0.318377	2.777644	4.683169
H	0.569113	2.826775	5.300171
C	-3.648085	13.567392	0.469275
H	-3.387410	14.595114	0.260078
C	-4.712880	11.636679	-0.211520
H	-5.299858	11.128232	-0.963077
C	-4.325055	10.952042	0.957933
H	-4.635983	9.922998	1.081232
C	-3.551211	11.615356	1.943312
C	-3.215276	12.965125	1.666070
H	-2.626909	13.557830	2.354829
C	-3.134818	10.966743	3.145346
C	-3.103298	9.141547	4.493982
C	-2.093928	11.062901	5.161365
N	-4.147320	5.055120	5.260893
C	-3.453591	7.780031	4.747147
C	-4.117190	6.979298	3.782389
H	-4.394220	7.374047	2.813067
C	-4.436400	5.638600	4.079259
H	-4.944333	5.028961	3.345398
C	-3.160357	7.149197	5.983036
H	-2.672325	7.680314	6.791484
C	-3.517173	5.802256	6.190254

H	-3.287599	5.326474	7.132865
C	-1.324045	11.789229	6.119657
C	-0.934721	11.221419	7.358998
H	-1.239480	10.223077	7.641103
C	-0.155231	11.968446	8.264225
H	0.145582	11.529228	9.204834
C	-0.125428	13.793602	6.850610
H	0.202880	14.806748	6.666436
C	-0.897495	13.120768	5.884061
H	-1.145529	13.643612	4.969038
C	3.378390	6.050564	-5.128907
H	4.326483	5.549202	-5.263019
C	1.081161	5.974257	-5.291207
H	0.196687	5.411352	-5.552847
C	0.953972	7.295248	-4.818307
H	-0.038262	7.719980	-4.736611
C	3.350325	7.373724	-4.646365
H	4.291495	7.860340	-4.423768
C	2.110506	8.040822	-4.478744
C	2.032222	9.384994	-4.003341
C	0.796650	11.196538	-3.416102
C	3.061368	11.270828	-3.271463
C	-0.472951	11.831687	-3.262586
C	-1.685006	11.180605	-3.604005
H	-1.695225	10.176729	-4.008148
C	-2.915473	11.841024	-3.420518
H	-3.836231	11.337546	-3.677339
C	-1.873111	13.730779	-2.611953
H	-1.963874	14.736288	-2.226112
C	-0.597794	13.150267	-2.756625
H	0.268931	13.733475	-2.472754
C	4.259021	11.994010	-2.987872
C	4.234787	13.330980	-2.516061
H	3.303455	13.855580	-2.345251
C	5.442780	14.009322	-2.261796
H	5.421239	15.027266	-1.900048
C	5.543212	11.419948	-3.168012
H	5.667019	10.405853	-3.525416
C	6.697866	12.176359	-2.887952
H	7.675554	11.736978	-3.025873
C	11.149213	14.458191	-0.834841
H	10.740366	13.457889	-1.003096
H	12.108386	14.499716	-1.366008
H	11.385417	14.541178	0.233432
C	9.804052	16.398925	-0.181778
H	8.722206	16.370853	-0.023852
H	10.282303	16.131927	0.768922
H	10.067078	17.446496	-0.374353
C	10.725891	16.255089	-2.452112
H	10.815993	17.320842	-2.205832
H	11.754423	15.945040	-2.676844
C	9.944963	16.180982	-3.787557
H	9.726314	17.217263	-4.075468
H	10.660097	15.838348	-4.546311
C	7.510102	16.187758	-4.096834
H	6.846050	16.198968	-3.228166
H	7.757885	17.233700	-4.316911
H	6.927620	15.823009	-4.952099
C	8.826488	14.243578	-4.796167
H	8.006349	14.228413	-5.524826
H	9.754663	14.284529	-5.379640
H	8.823550	13.279974	-4.278807
N	-4.984852	0.955517	5.941866
N	-6.441548	3.403460	6.566226
C	-4.995950	0.282816	4.615505
H	-4.183074	-0.444640	4.498175
H	-5.926786	-0.262344	4.415063
H	-4.890104	1.024314	3.819383
C	-3.888079	0.426773	6.795427
H	-3.282805	-0.336088	6.289916
H	-3.215981	1.239581	7.084444
H	-4.247070	-0.032417	7.725043
C	-6.306124	0.812144	6.620657
H	-6.199376	0.291252	7.580885
H	-6.975929	0.165652	6.039330
C	-7.099361	2.109383	6.910998
H	-8.064180	2.008228	6.397648
H	-7.359042	2.077046	7.976920
C	-7.273823	4.182293	5.610777
H	-7.490926	5.198351	5.963550
H	-6.758218	4.271584	4.650372
H	-8.245384	3.715649	5.404972
C	-6.155757	4.195786	7.791617
H	-6.578821	5.207478	7.755274
H	-6.546907	3.731834	8.705721
H	-5.075855	4.293680	7.930227
N	9.147012	1.599283	6.042298
N	10.566700	3.978606	6.951151

N	10.178757	15.486867	-1.295362
N	8.705126	15.350490	-3.810111
N	-6.745386	14.591731	-1.594409
N	-5.343441	14.759452	-4.148288
N	6.087123	12.234775	4.417527
N	6.188196	10.307198	5.853632
N	7.393923	10.322375	3.770277
N	8.359044	5.746578	5.660009
N	3.062907	13.480330	8.251552
N	2.294118	3.174455	-3.640913
N	3.357598	3.181820	1.280693
N	0.968620	2.907723	1.228955
N	2.135624	2.930928	3.338352
N	-2.743696	2.653595	4.638209
N	6.952745	3.424874	4.799499
N	-4.388559	12.923403	-0.455407
N	-3.466878	9.663062	3.299930
N	-2.398511	9.779131	5.455583
N	-2.423989	11.708850	4.022752
N	0.240224	13.235693	8.022529
N	2.268543	5.354462	-5.451715
N	0.798465	9.919889	-3.863725
N	1.889856	11.917904	-3.082359
N	3.198933	9.997556	-3.704636
N	6.656183	13.450719	-2.448634
N	-3.018577	13.095918	-2.936517
Pd	1.473324	14.273053	9.373823
Pd	-4.869730	13.839928	-2.285131
Pd	8.751797	3.692118	5.866736
Pd	8.410634	14.461041	-1.894916
Pd	2.358061	3.277931	-5.743775
Pd	-4.575084	3.021251	5.603205
C	2.544836	5.417501	6.111503
C	2.923321	6.088865	4.948086
C	2.006900	6.178962	3.845595
C	0.626244	6.088833	4.151796
C	0.273701	5.888965	5.529601
C	1.184822	5.304668	6.406609
H	3.451373	6.380085	2.258144
H	3.284079	5.148569	6.858783
H	3.972764	6.324512	4.776361
C	2.392757	6.346425	2.500375
C	-0.311900	6.251386	3.117268
H	-0.770167	5.958922	5.818604
H	0.863637	4.934862	7.376163
C	0.070436	6.423770	1.795334
C	1.460017	6.424386	1.473202
H	-1.371166	6.227636	3.364383
C	-1.013863	6.627941	0.752941
C	-1.853852	5.374008	0.476275
H	-0.586900	6.992607	-0.180562
H	-1.677319	7.424788	1.109757
H	-2.626328	5.572876	-0.269947
H	-1.227016	4.556237	0.108885
H	-2.352226	5.031885	1.389799
C	1.959347	6.543306	0.043610
C	2.109244	7.998029	-0.431709
H	1.293057	6.000918	-0.634702
H	2.936337	6.052390	-0.023148
H	2.531205	8.027279	-1.441078
H	1.147660	8.517553	-0.440600
H	2.780290	8.552372	0.228582
C	0.634611	7.952602	7.459832
C	0.880832	8.104507	5.999682
C	2.214565	8.194844	5.793946
C	2.904135	8.093447	7.108338
H	0.075674	8.407672	5.328558
H	2.730177	8.599984	4.918660
C	2.102450	7.671946	9.496736
C	2.918951	6.391505	9.735598
C	2.740132	8.886912	10.185464
H	1.093012	7.530311	9.896522
C	3.107136	6.141195	11.235581
H	3.895443	6.508073	9.248693
H	2.409200	5.545129	9.262513
C	2.930804	8.624889	11.687143
H	3.709022	9.088824	9.713354
H	2.094968	9.762581	10.023691
C	3.752042	7.350192	11.926966
H	3.715314	5.243208	11.385890
H	2.129935	5.937509	11.693229
H	3.416464	9.485544	12.158501
H	1.947442	8.517091	12.163378
H	3.848476	7.164903	13.001367
H	4.769292	7.494252	11.538938
N	1.897162	7.913716	8.061909
O	4.098194	8.167943	7.346481
O	-0.427853	7.889314	8.055910

Table5_1c_TSiv_TSi-ii_0wat

Property	Value
Charge	0
Electronic Energy, BS1 (a.u.)	-1137.518194
Thermal and entropic correction, BS1 (a.u.)	2.810117
Electronic Energy, BS2 (a.u.)	-1137.894576
Number of Imaginary Frequencies	0
Imaginary frequencies (cm-1)	None

Molecular Geometry in Cartesian Coordinates

C	8.060448	1.013387	6.872948
H	8.435695	0.476105	7.752892
H	7.409120	1.808242	7.246689
H	7.433064	0.301812	6.321596
C	9.123073	1.029780	4.664627
H	8.297122	0.325060	4.507061
H	9.016763	1.829329	3.926285
H	10.041342	0.486254	4.408786
C	10.477102	1.392609	6.677263
H	11.161963	0.867060	5.999470
H	10.393596	0.724520	7.544210
C	11.225842	2.653324	7.174505
H	12.221356	2.626720	6.713074
H	11.422233	2.493020	8.242391
C	11.437235	4.866723	6.138336
H	10.972429	5.065667	5.169407
H	11.622488	5.835708	6.618688
H	12.424351	4.435221	5.930178
C	10.208641	4.616896	8.242691
H	9.121204	4.704667	8.323649
H	10.545697	4.046404	9.117136
H	10.629449	5.624399	8.350927
N	2.741441	15.325429	10.728637
N	-0.157591	15.099441	10.482225
C	3.595200	14.312492	11.403916
H	3.288608	13.306534	11.103649
H	4.659672	14.419710	11.159902
H	3.525971	14.351040	12.498117
C	3.567593	16.262111	9.920657
H	3.251172	16.233775	8.874114
H	3.480543	17.306114	10.246757
H	4.637616	16.020755	9.947548
C	1.909190	16.068432	11.719989
H	2.156702	15.764084	12.745044
H	2.139376	17.141373	11.697763
C	0.370995	15.949640	11.588606
H	-0.013521	16.974780	11.512039
H	0.003472	15.598724	12.561481
C	-0.969552	15.906226	9.532571
H	-1.978643	15.502350	9.382538
H	-1.100406	16.947413	9.852897
H	-0.480286	15.937600	8.554982
C	-0.946498	13.959391	11.018964
H	-0.441853	13.017550	10.789932
H	-1.069723	13.994203	12.108727
H	-1.957539	13.902678	10.596618
N	2.478321	1.163263	-6.012155
N	2.445032	3.413756	-7.869927
C	3.678803	0.695159	-5.269524
H	3.430224	0.041235	-4.424355
H	4.381677	0.132549	-5.896680
H	4.230474	1.551565	-4.871548
C	1.236655	0.576232	-5.441794
H	1.426269	-0.055531	-4.564984
H	0.554266	1.372965	-5.132842
H	0.686287	-0.047776	-6.157063
C	2.596022	0.851190	-7.466586
H	1.796441	0.170277	-7.785504
H	3.519090	0.295490	-7.675356
C	2.569176	2.045189	-8.452768
H	3.476102	1.961447	-9.065280
H	1.751669	1.845526	-9.157342
C	1.189509	4.072130	-8.318148
H	1.362243	5.050461	-8.783924
H	0.628187	3.477919	-9.049938
H	0.525171	4.227877	-7.463289
C	3.631427	4.244361	-8.209306
H	3.365413	5.189436	-8.699111
H	4.187748	4.490987	-7.300746
H	4.335604	3.738004	-8.881350
C	-6.472023	15.445512	-0.410759

H	-6.899195	15.041844	0.515708
H	-5.393512	15.540695	-0.260352
H	-6.865055	16.464948	-0.512066
C	-7.579851	13.416322	-1.223155
H	-7.811365	13.379609	-0.151245
H	-8.542949	13.385613	-1.747747
H	-7.053811	12.491691	-1.477538
C	-7.396357	15.382100	-2.682540
H	-7.588718	16.412134	-2.356104
H	-8.392947	14.984518	-2.913524
C	-6.643315	15.484285	-4.032045
H	-7.348112	15.147603	-4.803132
H	-6.514528	16.554722	-4.237180
C	-5.431149	13.674247	-5.164333
H	-4.654523	13.747657	-5.935972
H	-5.320962	12.700492	-4.678595
H	-6.391600	13.654394	-5.694258
C	-4.237175	15.698090	-4.469234
H	-3.679517	15.410796	-5.369487
H	-4.579249	16.727152	-4.635951
H	-3.523673	15.733986	-3.641087
N	8.081180	13.595715	-0.010470
C	7.255960	14.177184	0.884092
H	6.906623	15.173521	0.654010
C	6.846203	13.551012	2.077145
H	6.185444	14.092507	2.741935
C	7.301799	12.243705	2.383672
C	8.161485	11.643967	1.429226
H	8.561583	10.648373	1.572177
C	8.517029	12.348725	0.262818
H	9.172359	11.888851	-0.462762
C	6.916757	11.571424	3.582449
C	5.764125	11.549727	5.538057
C	4.865940	12.189296	6.445558
C	4.347461	13.484645	6.193362
H	4.616967	14.042522	5.305626
C	3.460763	14.079083	7.110600
H	3.069595	15.067030	6.913460
C	3.544215	12.244964	8.505683
H	3.224672	11.774855	9.424165
C	4.436271	11.570855	7.646859
H	4.790629	10.588665	7.931779
C	7.004987	9.748148	4.931897
C	7.462302	8.417750	5.178574
C	7.143170	7.720341	6.370173
H	6.562533	8.184460	7.155770
C	8.263614	7.713968	4.244346
H	8.570676	8.162722	3.308158
C	8.680624	6.398269	4.524240
H	9.292435	5.866434	3.809443
C	7.602558	6.402733	6.562867
H	7.354743	5.877341	7.473305
C	3.419529	3.286511	-2.907819
H	4.348397	3.396295	-3.449479
C	3.426398	3.257320	-1.500356
H	4.378456	3.339750	-0.991897
C	1.125425	3.041711	-2.981429
H	0.230877	2.961954	-3.582176
C	1.035740	2.997296	-1.575392
H	0.060652	2.867203	-1.124640
C	2.209985	3.103211	-0.787229
C	2.175903	3.052766	0.639507
C	1.015870	2.858742	2.580720
C	3.273347	3.091645	2.625378
C	4.497483	3.206556	3.352376
C	4.545751	3.119948	4.766252
H	3.650481	2.947630	5.349475
C	5.740751	3.408558	2.699876
H	5.813427	3.476601	1.622061
C	6.925524	3.511835	3.454134
H	7.872585	3.658822	2.953913
C	5.778733	3.242515	5.438617
H	5.812743	3.179493	6.516197
C	-0.232380	2.762316	3.268367
C	-1.464214	2.661690	2.575731
H	-1.504401	2.611839	1.495919
C	-2.675760	2.615326	3.291510
H	-3.611353	2.541538	2.755476
C	-1.578295	2.727719	5.314040
H	-1.638696	2.745116	6.392569
C	-0.318565	2.774479	4.682951
H	0.568886	2.823657	5.300013
C	-3.650787	13.567091	0.468728
H	-3.390603	14.594944	0.259560
C	-4.715256	11.636105	-0.211760
H	-5.302484	11.127550	-0.963052
C	-4.326512	10.951416	0.957363
H	-4.637016	9.922250	1.080703

C	-3.552273	11.614825	1.942353
C	-3.217050	12.964784	1.665162
H	-2.628483	13.557598	2.353658
C	-3.134727	10.966110	3.143930
C	-3.101308	9.140609	4.492167
C	-2.093018	11.062620	5.159456
N	-4.143335	5.053552	5.258968
C	-3.450495	7.778753	4.745215
C	-4.114429	6.977960	3.780733
H	-4.392234	7.372821	2.811682
C	-4.433174	5.637141	4.077574
H	-4.941432	5.027538	3.343906
C	-3.156058	7.147681	5.980728
H	-2.667374	7.678742	6.788840
C	-3.512753	5.800679	6.188051
H	-3.282646	5.324765	7.130470
C	-1.323460	11.789408	6.117649
C	-0.935124	11.222218	7.357523
H	-1.240833	10.224346	7.640076
C	-0.155583	11.969224	8.262662
H	0.144609	11.530232	9.203580
C	-0.124299	13.793638	6.848107
H	0.204616	14.806512	6.663536
C	-0.896325	13.120677	5.881569
H	-1.143684	13.643085	4.966116
C	3.378085	6.051361	-5.130215
H	4.326306	5.550267	-5.264430
C	1.080906	5.974617	-5.292647
H	0.196557	5.411633	-5.554549
C	0.953402	7.295441	-4.819363
H	-0.038933	7.719954	-4.737685
C	3.349718	7.374331	-4.647198
H	4.290782	7.861078	-4.424457
C	2.109758	8.041092	-4.479374
C	2.031225	9.385048	-4.003439
C	0.795379	11.196219	-3.415655
C	3.060076	11.270641	-3.270498
C	-0.474365	11.831107	-3.262186
C	-1.686253	11.179841	-3.603897
H	-1.696246	10.176025	-4.008190
C	-2.916885	11.839939	-3.420392
H	-3.837505	11.336265	-3.677304
C	-1.875005	13.729774	-2.611340
H	-1.966061	14.735167	-2.225269
C	-0.599540	13.149559	-2.755975
H	0.267041	13.732878	-2.471889
C	4.257702	11.993621	-2.986239
C	4.233484	13.330324	-2.513667
H	3.302149	13.854881	-2.342733
C	5.441493	14.008392	-2.258711
H	5.420002	15.026096	-1.896284
C	5.541896	11.419552	-3.166423
H	5.665718	10.405644	-3.524348
C	6.696562	12.175672	-2.885684
H	7.674248	11.736274	-3.023590
C	11.149317	14.458354	-0.836875
H	10.740440	13.457991	-1.004706
H	12.107860	14.500096	-1.369159
H	11.386729	14.541303	0.231134
C	9.804502	16.398743	-0.182110
H	8.722864	16.370363	-0.022847
H	10.283997	16.131822	0.767981
H	10.067007	17.446404	-0.374910
C	10.723735	16.255335	-2.453516
H	10.813533	17.321150	-2.207384
H	11.752205	15.945793	-2.679230
C	9.941583	16.180751	-3.788214
H	9.722241	17.216903	-4.076057
H	10.656145	15.838297	-4.547588
C	7.506476	16.186497	-4.095446
H	6.843173	16.197783	-3.226210
H	7.753701	17.232454	-4.316078
H	6.923392	15.821265	-4.950097
C	8.823035	14.242658	-4.795378
H	8.002249	14.226953	-5.523297
H	9.750673	14.283885	-5.379683
H	8.820996	13.279176	-4.277783
N	-4.989167	0.955549	5.940714
N	-6.440381	3.406664	6.565709
C	-5.004017	0.283885	4.613869
H	-4.193365	-0.445791	4.494808
H	-5.936697	-0.258429	4.414297
H	-4.897153	1.025759	3.818215
C	-3.892455	0.423274	6.792176
H	-3.290043	-0.340852	6.285165
H	-3.217698	1.233999	7.080753
H	-4.251207	-0.035685	7.721991
C	-6.309715	0.815099	6.621516

H	-6.202663	0.294465	7.581849
H	-6.981587	0.169639	6.041427
C	-7.100049	2.114001	6.912283
H	-8.065822	2.014328	6.400437
H	-7.358232	2.082760	7.978599
C	-7.271652	4.185554	5.609447
H	-7.487234	5.202353	5.961019
H	-6.756013	4.272918	4.648885
H	-8.243888	3.720049	5.404275
C	-6.153192	4.200069	7.790102
H	-6.574754	5.212346	7.752594
H	-6.544909	3.737843	8.704845
H	-5.073135	4.296555	7.928487
N	9.144044	1.597870	6.039738
N	10.567393	3.973983	6.951137
N	10.178125	15.486861	-1.296209
N	8.702062	15.349759	-3.809490
N	-6.747900	14.590723	-1.596327
N	-5.344821	14.757709	-4.149598
N	6.089767	12.234596	4.419791
N	6.190289	10.306771	5.855615
N	7.396661	10.322383	3.772516
N	8.362622	5.746710	5.661986
N	3.063277	13.480361	8.251489
N	2.294099	3.176229	-3.641127
N	3.356621	3.188532	1.280712
N	0.968059	2.910865	1.228782
N	2.134983	2.934800	3.338215
N	-2.743903	2.648790	4.638133
N	6.952242	3.428244	4.799352
N	-4.391539	12.922988	-0.455653
N	-3.465960	9.662197	3.298451
N	-2.396984	9.778701	5.453746
N	-2.423541	11.708387	4.020890
N	0.240649	13.236118	8.020466
N	2.268420	5.355097	-5.453305
N	0.797404	9.919738	-3.863756
N	1.888461	11.917556	-3.081373
N	3.197810	9.997593	-3.704255
N	6.654870	13.449761	-2.445634
N	-3.020288	13.094719	-2.936175
Pd	1.472931	14.273789	9.372268
Pd	-4.871889	13.838789	-2.285942
Pd	8.751922	3.691489	5.866632
Pd	8.409531	14.460703	-1.893783
Pd	2.358325	3.278463	-5.744032
Pd	-4.574617	3.020500	5.602874
C	2.538756	5.412978	6.124163
C	2.900185	6.209205	4.992221
C	2.002724	6.192547	3.844550
C	0.628039	6.092456	4.146412
C	0.294240	5.986965	5.555950
C	1.204735	5.295589	6.412286
H	3.453257	6.402517	2.267774
H	3.293957	5.091011	6.832877
H	3.954929	6.395323	4.805371
C	2.394696	6.350675	2.508893
C	-0.310115	6.239194	3.119725
H	-0.754920	5.995911	5.828951
H	0.863435	4.867063	7.347852
C	0.075427	6.420579	1.793173
C	1.459663	6.427884	1.475103
H	-1.369469	6.217497	3.365948
C	-1.011491	6.626949	0.756018
C	-1.851488	5.373042	0.479507
H	-0.586910	6.990994	-0.180394
H	-1.674917	7.424233	1.112002
H	-2.622174	5.570896	-0.268797
H	-1.223818	4.554451	0.115417
H	-2.352233	5.032584	1.392357
C	1.962817	6.551561	0.048895
C	2.107967	8.006413	-0.427434
H	1.299167	6.006402	-0.632723
H	2.941557	6.063988	-0.016633
H	2.532115	8.036227	-1.435863
H	1.144347	8.522004	-0.439228
H	2.775209	8.564232	0.233758
C	0.637137	7.949762	7.458051
C	0.859567	7.997162	5.985992
C	2.253375	8.087239	5.761329
C	2.913326	8.081249	7.104317
H	0.107967	8.443106	5.350138
H	2.705088	8.647376	4.954985
C	2.105659	7.673205	9.494072
C	2.919334	6.391116	9.733517
C	2.744731	8.886520	10.187332
H	1.095308	7.533388	9.892189
C	3.105507	6.136777	11.236495

H	3.896640	6.503039	9.250758
H	2.408509	5.545845	9.259670
C	2.933294	8.620423	11.689071
H	3.714531	9.084184	9.716442
H	2.101276	9.763369	10.025378
C	3.752031	7.344194	11.929115
H	3.711809	5.237537	11.383995
H	2.127461	5.934739	11.693071
H	3.419715	9.480070	12.161449
H	1.949221	8.513848	12.164094
H	3.847055	7.158183	13.003520
H	4.769933	7.486539	11.542162
N	1.902944	7.919549	8.056279
O	4.104891	8.163109	7.350176
O	-0.422445	7.900537	8.057436

Table5_1c_TSiv_DG_0wat

Property	Value
Charge	0
Electronic Energy, BS1 (a.u.)	-1137.561785
Thermal and entropic correction, BS1 (a.u.)	2.811900
Electronic Energy, BS2 (a.u.)	-1137.935624
Number of Imaginary Frequencies	0
Imaginary frequencies (cm-1)	None

Molecular Geometry in Cartesian Coordinates

C	8.064188	1.014136	6.876055
H	8.440132	0.478556	7.756739
H	7.411547	1.808447	7.248676
H	7.438000	0.300881	6.325510
C	9.127038	1.029564	4.667837
H	8.302245	0.323311	4.511051
H	9.019470	1.828048	3.928522
H	10.046191	0.487204	4.412703
C	10.480304	1.396768	6.680219
H	11.166079	0.871602	6.003054
H	10.397731	0.729424	7.547829
C	11.227005	2.659167	7.176283
H	12.222696	2.633478	6.715177
H	11.423295	2.500395	8.244417
C	11.435421	4.871534	6.137353
H	10.970607	5.068395	5.168012
H	11.619038	5.841480	6.616384
H	12.423263	4.441261	5.930095
C	10.206834	4.622674	8.241816
H	9.119268	4.709158	8.322456
H	10.544480	4.053724	9.117039
H	10.626320	5.630853	8.348860
N	2.742640	15.324369	10.729640
N	-0.156503	15.097991	10.485255
C	3.597017	14.311353	11.404049
H	3.290273	13.305404	11.103894
H	4.661276	14.418699	11.159156
H	3.528696	14.349694	12.498317
C	3.568103	16.261356	9.921322
H	3.250955	16.233236	8.874987
H	3.481125	17.305263	10.247743
H	4.638172	16.020134	9.947411
C	1.911002	16.067033	11.721757
H	2.159376	15.762577	12.746573
H	2.140940	17.140026	11.699526
C	0.372730	15.947913	11.591540
H	-0.012083	16.972990	11.515620
H	0.006036	15.596565	12.564571
C	-0.969310	15.904995	9.536505
H	-1.978452	15.501020	9.387083
H	-1.100111	16.946036	9.857330
H	-0.480779	15.936872	8.558570
C	-0.944790	13.957583	11.022147
H	-0.440029	13.015911	10.792576
H	-1.067383	13.992121	12.111987
H	-1.956066	13.900729	10.600388
N	2.477415	1.162850	-6.013159
N	2.444572	3.414470	-7.869599
C	3.677899	0.693904	-5.271058
H	3.429278	0.039708	-4.426112
H	4.380357	0.131279	-5.898667
H	4.230068	1.549875	-4.872860
C	1.235663	0.575906	-5.442901
H	1.425236	-0.056544	-4.566578
H	0.553669	1.372701	-5.133222
H	0.684872	-0.047385	-6.158468

C	2.594745	0.851602	-7.467798
H	1.794743	0.171338	-7.787042
H	3.517472	0.295504	-7.677009
C	2.568526	2.046225	-8.453254
H	3.475594	1.962603	-9.065574
H	1.751162	1.847216	-9.158182
C	1.189092	4.073277	-8.317305
H	1.361922	5.051838	-8.782567
H	0.627568	3.479556	-9.049338
H	0.524892	4.228656	-7.462271
C	3.631050	4.245133	-8.208554
H	3.365109	5.190576	-8.697694
H	4.187523	4.491057	-7.299897
H	4.335088	3.739134	-8.881014
C	-6.468914	15.446170	-0.408732
H	-6.895669	15.042270	0.517827
H	-5.390324	15.541218	-0.258821
H	-6.861918	16.465664	-0.509580
C	-7.577189	13.417243	-1.221177
H	-7.808086	13.380168	-0.149148
H	-8.540598	13.386764	-1.745215
H	-7.051341	12.492679	-1.476196
C	-7.394326	15.383411	-2.680115
H	-7.586603	16.413333	-2.353271
H	-8.390986	14.985852	-2.910829
C	-6.641849	15.486051	-4.029910
H	-7.346988	15.149682	-4.800824
H	-6.513104	16.556556	-4.234710
C	-5.430247	13.676298	-5.163316
H	-4.654150	13.750093	-5.935449
H	-5.319576	12.702398	-4.677974
H	-6.391047	13.656451	-5.692606
C	-4.235875	15.699877	-4.468093
H	-3.678493	15.412732	-5.368561
H	-4.577967	16.728983	-4.634510
H	-3.522109	15.735580	-3.640168
N	8.080359	13.595630	-0.012145
C	7.254839	14.177239	0.882056
H	6.905791	15.173644	0.651844
C	6.844439	13.551112	2.074924
H	6.183498	14.092680	2.739476
C	7.299674	12.243719	2.381601
C	8.159672	11.643851	1.427502
H	8.559563	10.648190	1.570574
C	8.515875	12.348567	0.261288
H	9.171469	11.888621	-0.464010
C	6.914126	11.571474	3.580234
C	5.761948	11.550093	5.536064
C	4.864368	12.189704	6.444130
C	4.344917	13.484661	6.191994
H	4.613194	14.042257	5.303708
C	3.458858	14.078926	7.110025
H	3.066934	15.066599	6.913011
C	3.544870	12.245352	8.505670
H	3.226546	11.775296	9.424599
C	4.436445	11.571568	7.646198
H	4.791865	10.589848	7.931330
C	7.002286	9.748221	4.929677
C	7.459194	8.417680	5.176358
C	7.138669	7.719896	6.367386
H	6.556838	8.183648	7.152318
C	8.261365	7.714119	4.242690
H	8.569282	8.163137	3.306909
C	8.678109	6.398320	4.522656
H	9.290546	5.866600	3.808300
C	7.598005	6.402304	6.560273
H	7.349233	5.876591	7.470260
C	3.419817	3.283013	-2.907758
H	4.348645	3.392716	-3.449499
C	3.426998	3.252254	-1.500336
H	4.379216	3.333451	-0.991979
C	1.125475	3.040161	-2.981097
H	0.230730	2.961760	-3.581725
C	1.036098	2.994347	-1.575080
H	0.061102	2.864528	-1.124059
C	2.210600	3.098603	-0.787110
C	2.176651	3.047589	0.639592
C	1.016336	2.856794	2.580930
C	3.274189	3.085689	2.625399
C	4.498298	3.200695	3.352412
C	4.546490	3.114857	4.766335
H	3.651256	2.942190	5.349514
C	5.741537	3.402667	2.699892
H	5.814273	3.470085	1.622042
C	6.926144	3.507279	3.454211
H	7.873155	3.654568	2.953976
C	5.779292	3.238816	5.438779
H	5.813170	3.176792	6.516433

C	-0.232116	2.763430	3.268595
C	-1.464001	2.663630	2.575899
H	-1.504133	2.612873	1.496110
C	-2.675635	2.618935	3.291626
H	-3.611313	2.545458	2.755676
C	-1.578185	2.732524	5.314236
H	-1.638780	2.751199	6.392719
C	-0.318377	2.777644	4.683169
H	0.569113	2.826775	5.300171
C	-3.648085	13.567392	0.469275
H	-3.387410	14.595114	0.260078
C	-4.712880	11.636679	-0.211520
H	-5.299858	11.128232	-0.963077
C	-4.325055	10.952042	0.957933
H	-4.635983	9.922998	1.081232
C	-3.551211	11.615356	1.943312
C	-3.215276	12.965125	1.666070
H	-2.626909	13.557830	2.354829
C	-3.134818	10.966743	3.145346
C	-3.103298	9.141547	4.493982
C	-2.093928	11.062901	5.161365
N	-4.147320	5.055120	5.260893
C	-3.453591	7.780031	4.747147
C	-4.117190	6.979298	3.782389
H	-4.394220	7.374047	2.813067
C	-4.436400	5.638600	4.079259
H	-4.944333	5.028961	3.345398
C	-3.160357	7.149197	5.983036
H	-2.672325	7.680314	6.791484
C	-3.517173	5.802256	6.190254
H	-3.287599	5.326474	7.132865
C	-1.324045	11.789229	6.119657
C	-0.934721	11.221419	7.358998
H	-1.239480	10.223077	7.641103
C	-0.155231	11.968446	8.264225
H	0.145582	11.529228	9.204834
C	-0.125428	13.793602	6.850610
H	0.202880	14.806748	6.666436
C	-0.897495	13.120768	5.884061
H	-1.145529	13.643612	4.969038
C	3.378390	6.050564	-5.128907
H	4.326483	5.549202	-5.263019
C	1.081161	5.974257	-5.291207
H	0.196687	5.411352	-5.552847
C	0.953972	7.295248	-4.818307
H	-0.038262	7.719980	-4.736611
C	3.350325	7.373724	-4.646365
H	4.291495	7.860340	-4.423768
C	2.110506	8.040822	-4.478744
C	2.032222	9.384994	-4.003341
C	0.796650	11.196538	-3.416102
C	3.061368	11.270828	-3.271463
C	-0.472951	11.831687	-3.262586
C	-1.685006	11.180605	-3.604005
H	-1.695225	10.176729	-4.008148
C	-2.915473	11.841024	-3.420518
H	-3.836231	11.337546	-3.677339
C	-1.873111	13.730779	-2.611953
H	-1.963874	14.736288	-2.226112
C	-0.597794	13.150267	-2.756625
H	0.268931	13.733475	-2.472754
C	4.259021	11.994010	-2.987872
C	4.234787	13.330980	-2.516061
H	3.303455	13.855580	-2.345251
C	5.442780	14.009322	-2.261796
H	5.421239	15.027266	-1.900048
C	5.543212	11.419948	-3.168012
H	5.667019	10.405853	-3.525416
C	6.697866	12.176359	-2.887952
H	7.675554	11.736978	-3.025873
C	11.149213	14.458191	-0.834841
H	10.740366	13.457889	-1.003096
H	12.108386	14.499716	-1.366008
H	11.385417	14.541178	0.233432
C	9.804052	16.398925	-0.181778
H	8.722206	16.370853	-0.023852
H	10.282303	16.131927	0.768922
H	10.067078	17.446496	-0.374353
C	10.725891	16.255089	-2.452112
H	10.815993	17.320842	-2.205832
H	11.754423	15.945040	-2.676844
C	9.944963	16.180982	-3.787557
H	9.726314	17.217263	-4.075468
H	10.660097	15.838348	-4.546311
C	7.510102	16.187758	-4.096834
H	6.846050	16.198968	-3.228166
H	7.757885	17.233700	-4.316911
H	6.927620	15.823009	-4.952099

C	8.826488	14.243578	-4.796167
H	8.006349	14.228413	-5.524826
H	9.754663	14.284529	-5.379640
H	8.823550	13.279974	-4.278807
N	-4.984852	0.955517	5.941866
N	-6.441548	3.403460	6.566226
C	-4.995950	0.282816	4.615505
H	-4.183074	-0.444640	4.498175
H	-5.926786	-0.262344	4.415063
H	-4.890104	1.024314	3.819383
C	-3.888079	0.426773	6.795427
H	-3.282805	-0.336088	6.289916
H	-3.215981	1.239581	7.084444
H	-4.247070	-0.032417	7.725043
C	-6.306124	0.812144	6.620657
H	-6.199376	0.291252	7.580885
H	-6.975929	0.165652	6.039330
C	-7.099361	2.109383	6.910998
H	-8.064180	2.008228	6.397648
H	-7.359042	2.077046	7.976920
C	-7.273823	4.182293	5.610777
H	-7.490926	5.198351	5.963550
H	-6.758218	4.271584	4.650372
H	-8.245384	3.715649	5.404972
C	-6.155757	4.195786	7.791617
H	-6.578821	5.207478	7.755274
H	-6.546907	3.731834	8.705721
H	-5.075855	4.293680	7.930227
N	9.147012	1.599283	6.042298
N	10.566700	3.978606	6.951151
N	10.178757	15.486867	-1.295362
N	8.705126	15.350490	-3.810111
N	-6.745386	14.591731	-1.594409
N	-5.343441	14.759452	-4.148288
N	6.087123	12.234775	4.417527
N	6.188196	10.307198	5.853632
N	7.393923	10.322375	3.770277
N	8.359044	5.746578	5.660009
N	3.062907	13.480330	8.251552
N	2.294118	3.174455	-3.640913
N	3.357598	3.181820	1.280693
N	0.968620	2.907723	1.228955
N	2.135624	2.930928	3.338352
N	-2.743696	2.653595	4.638209
N	6.952745	3.424874	4.799499
N	-4.388559	12.923403	-0.455407
N	-3.466878	9.663062	3.299930
N	-2.398511	9.779131	5.455583
N	-2.423989	11.708850	4.022752
N	0.240224	13.235693	8.022529
N	2.268543	5.354462	-5.451715
N	0.798465	9.919889	-3.863725
N	1.889856	11.917904	-3.082359
N	3.198933	9.997556	-3.704636
N	6.656183	13.450719	-2.448634
N	-3.018577	13.095918	-2.936517
Pd	1.473324	14.273053	9.373823
Pd	-4.869730	13.839928	-2.285131
Pd	8.751797	3.692118	5.866736
Pd	8.410634	14.461041	-1.894916
Pd	2.358061	3.277931	-5.743775
Pd	-4.575084	3.021251	5.603205
C	2.521593	5.394258	6.128935
C	2.876836	6.303860	5.023624
C	1.995278	6.196394	3.833973
C	0.626244	6.106265	4.134364
C	0.314376	6.121392	5.581897
C	1.213875	5.293046	6.412420
H	3.451373	6.391707	2.263955
H	3.289890	5.003302	6.794865
H	3.937900	6.434915	4.822846
C	2.392757	6.334803	2.506186
C	-0.311900	6.239764	3.117268
H	-0.741114	6.069325	5.836036
H	0.846205	4.807028	7.312245
C	0.076247	6.417959	1.783712
C	1.454206	6.418575	1.467391
H	-1.371166	6.233447	3.364383
C	-1.013863	6.627941	0.752941
C	-1.853852	5.374008	0.476275
H	-0.592711	6.992607	-0.186373
H	-1.677319	7.424788	1.109757
H	-2.626328	5.572876	-0.269947
H	-1.227016	4.556237	0.108885
H	-2.352226	5.031885	1.389799
C	1.959347	6.543306	0.043610
C	2.109244	7.998029	-0.431709
H	1.293057	6.000918	-0.640513

H	2.936337	6.052390	-0.023148
H	2.531205	8.027279	-1.441078
H	1.147660	8.517553	-0.440600
H	2.780290	8.552372	0.228582
C	0.628800	7.935170	7.459832
C	0.822725	7.866270	5.970629
C	2.278483	7.985659	5.730028
C	2.909946	8.076015	7.096716
H	0.127970	8.477401	5.380854
H	2.660448	8.698765	4.988389
C	2.102450	7.677757	9.490925
C	2.918951	6.397316	9.729787
C	2.740132	8.892723	10.185464
H	1.093012	7.536122	9.890711
C	3.107136	6.141195	11.235581
H	3.895443	6.508073	9.248693
H	2.409200	5.550940	9.256702
C	2.930804	8.624889	11.687143
H	3.709022	9.088824	9.713354
H	2.094968	9.768392	10.023691
C	3.752042	7.350192	11.926966
H	3.715314	5.243208	11.380079
H	2.129935	5.937509	11.693229
H	3.416464	9.485544	12.158501
H	1.947442	8.517091	12.163378
H	3.848476	7.164903	13.001367
H	4.769292	7.494252	11.538938
N	1.897162	7.925338	8.050287
O	4.098194	8.173754	7.352292
O	-0.427853	7.895125	8.061721

Table5_1c_reactant_1wat

Property	Value
Charge	0
Electronic Energy, BS1 (a.u.)	-1214.016793
Thermal and entropic correction, BS1 (a.u.)	2.827257
Electronic Energy, BS2 (a.u.)	-1214.432006
Number of Imaginary Frequencies	0
Imaginary frequencies (cm-1)	None

Molecular Geometry in Cartesian Coordinates

C	8.401132	1.034535	6.890766
H	8.782109	0.583704	7.815733
H	7.631725	1.753623	7.186160
H	7.903408	0.227966	6.337584
C	9.586990	1.102222	4.747266
H	8.871228	0.287661	4.578088
H	9.415698	1.853951	3.971870
H	10.582285	0.679395	4.560950
C	10.762548	1.703236	6.815729
H	11.533234	1.196996	6.220210
H	10.695937	1.102699	7.732027
C	11.359131	3.069760	7.232627
H	12.369108	3.110548	6.804552
H	11.530138	3.014199	8.315377
C	11.371575	5.187992	5.994406
H	10.898051	5.254982	5.011306
H	11.461051	6.208853	6.386756
H	12.396211	4.833194	5.825776
C	10.140840	5.013766	8.105749
H	9.050176	4.987894	8.180952
H	10.531259	4.571091	9.030652
H	10.447300	6.067421	8.115583
N	3.137889	15.110185	10.431464
N	0.264481	15.391235	10.857808
C	3.907973	13.942434	10.935349
H	3.354304	13.019893	10.741468
H	4.893241	13.843690	10.462817
H	4.088162	13.978207	12.017096
C	3.933931	15.887336	9.444342
H	3.391369	15.960460	8.498328
H	4.137275	16.915848	9.768336
H	4.910463	15.434551	9.231834
C	2.695058	15.981453	11.559414
H	3.110948	15.629256	12.512122
H	3.096588	16.997255	11.452028
C	1.170336	16.135168	11.781278
H	0.958923	17.211884	11.754845
H	0.980809	15.851344	12.824483
C	-0.622278	16.326621	10.115242
H	-1.687143	16.088226	10.230558
H	-0.506105	17.371803	10.428246

H	-0.393663	16.291004	9.046111
C	-0.533818	14.376461	11.595575
H	-0.254632	13.371506	11.266478
H	-0.375792	14.408526	12.680858
H	-1.614342	14.485004	11.438096
N	2.376968	1.265358	-6.269861
N	2.346414	3.676515	-7.913371
C	3.607080	0.706520	-5.648659
H	3.393171	-0.017717	-4.852486
H	4.257510	0.191335	-6.366709
H	4.201502	1.512367	-5.208844
C	1.157143	0.656737	-5.675697
H	1.383044	-0.062089	-4.877937
H	0.522317	1.436778	-5.245664
H	0.542436	0.119222	-6.408597
C	2.396922	1.083985	-7.751043
H	1.545798	0.475179	-8.082205
H	3.276485	0.505870	-8.062536
C	2.384119	2.363851	-8.623144
H	3.260634	2.299387	-9.280721
H	1.530356	2.263881	-9.305628
C	1.108564	4.428488	-8.251478
H	1.311469	5.439769	-8.625993
H	0.499266	3.934045	-9.018367
H	0.476722	4.527449	-7.364342
C	3.558260	4.481590	-8.220918
H	3.321000	5.479595	-8.610538
H	4.157009	4.615328	-7.315312
H	4.212768	4.009863	-8.964615
C	-6.574709	15.753017	-0.661934
H	-7.114711	15.468913	0.249997
H	-5.515537	15.835761	-0.404288
H	-6.915620	16.764031	-0.918406
C	-7.660594	13.671160	-1.361488
H	-8.010828	13.765743	-0.325867
H	-8.561438	13.601404	-1.983898
H	-7.143654	12.711069	-1.446510
C	-7.252709	15.451836	-3.001708
H	-7.482501	16.506418	-2.802159
H	-8.212358	15.025183	-3.320645
C	-6.329726	15.435563	-4.245496
H	-6.928573	15.024186	-5.068153
H	-6.172238	16.484194	-4.528755
C	-4.960570	13.577890	-5.080036
H	-4.074899	13.613820	-5.726636
H	-4.930739	12.631606	-4.533324
H	-5.827552	13.525284	-5.750512
C	-3.884631	15.645826	-4.326490
H	-3.194539	15.311122	-5.111120
H	-4.197351	16.659891	-4.605334
H	-3.309424	15.737317	-3.400784
N	8.073701	13.646537	-0.018816
C	7.187761	14.204204	0.832798
H	6.870160	15.213665	0.613678
C	6.684812	13.543736	1.971641
H	5.988828	14.072231	2.609692
C	7.112160	12.226148	2.272453
C	8.039880	11.653137	1.365728
H	8.431228	10.654045	1.507887
C	8.481977	12.389318	0.249258
H	9.189734	11.947517	-0.437496
C	6.645554	11.521409	3.423657
C	5.412619	11.464860	5.329047
C	4.452293	12.072424	6.197701
C	3.896777	13.350635	5.931469
H	4.181633	13.922196	5.057441
C	2.961940	13.915287	6.823076
H	2.542957	14.890986	6.621265
C	3.054459	12.077744	8.210557
H	2.705745	11.588734	9.108981
C	3.996206	11.436668	7.379967
H	4.362839	10.461741	7.673035
C	6.723143	9.692878	4.769002
C	7.204205	8.371121	5.026490
C	6.885032	7.659788	6.213290
H	6.283448	8.112358	6.994566
C	8.035742	7.690852	4.100347
H	8.335845	8.146641	3.165911
C	8.493108	6.389388	4.382287
H	9.126109	5.876764	3.672407
C	7.389089	6.354912	6.408304
H	7.154953	5.817282	7.315456
C	3.473857	3.073831	-3.018905
H	4.394783	3.098458	-3.584287
C	3.516274	3.057893	-1.611099
H	4.484469	3.062414	-1.126502
C	1.167325	3.021970	-3.030782
H	0.253097	3.002538	-3.606662

C	1.111301	3.007328	-1.623327
H	0.140505	2.970025	-1.146134
C	2.310263	3.021702	-0.866524
C	2.300295	3.000376	0.560388
C	1.147039	2.982973	2.510940
C	3.417313	3.000361	2.536694
C	4.642377	3.042529	3.269755
C	4.673212	2.981832	4.686614
H	3.765885	2.866773	5.265876
C	5.899657	3.162636	2.626873
H	5.984057	3.208816	1.549199
C	7.078052	3.235049	3.395473
H	8.034695	3.331665	2.901909
C	5.903143	3.062137	5.369828
H	5.922460	3.014863	6.448441
C	-0.093089	3.046309	3.213218
C	-1.339717	3.011736	2.538396
H	-1.387688	2.918618	1.457556
C	-2.539291	3.073603	3.279105
H	-3.490956	3.042403	2.767449
C	-1.392631	3.216332	5.279480
H	-1.433473	3.300338	6.355851
C	-0.147738	3.152867	4.625747
H	0.752671	3.189411	5.225460
C	-3.962723	13.782336	0.721707
H	-3.747709	14.822973	0.524929
C	-4.913766	11.808645	0.008466
H	-5.459871	11.276732	-0.758090
C	-4.539071	11.141936	1.192566
H	-4.818492	10.102705	1.309244
C	-3.839021	11.841478	2.207605
C	-3.551273	13.202784	1.936773
H	-3.032948	13.831539	2.648434
C	-3.462853	11.221087	3.437128
C	-3.454295	9.414796	4.809940
C	-2.521332	11.369463	5.498052
N	-4.319537	5.267612	5.513566
C	-3.751002	8.037200	5.046964
C	-4.400317	7.229013	4.079299
H	-4.723337	7.633241	3.127554
C	-4.661074	5.871506	4.355366
H	-5.171784	5.262653	3.622517
C	-3.396383	7.388562	6.258557
H	-2.908016	7.921024	7.065259
C	-3.689102	6.021734	6.441538
H	-3.416375	5.538730	7.371473
C	-1.802583	12.121928	6.476677
C	-1.428313	11.565674	7.725292
H	-1.707951	10.557655	8.000654
C	-0.660465	12.322548	8.631360
H	-0.374095	11.888175	9.577958
C	-0.622751	14.132823	7.215476
H	-0.309716	15.151096	7.034204
C	-1.383380	13.454819	6.242594
H	-1.631552	13.973481	5.326092
C	3.375176	6.081497	-5.043464
H	4.315576	5.589010	-5.246975
C	1.071136	5.995830	-5.072213
H	0.175880	5.435690	-5.302295
C	0.966408	7.298963	-4.547420
H	-0.021559	7.714103	-4.396840
C	3.368848	7.390709	-4.520728
H	4.318976	7.876859	-4.338127
C	2.137666	8.043725	-4.258137
C	2.076899	9.374141	-3.741191
C	0.852536	11.153401	-3.038691
C	3.119210	11.272370	-3.058054
C	-0.417940	11.772621	-2.832946
C	-1.632237	11.096099	-3.112273
H	-1.644077	10.079381	-3.483220
C	-2.863402	11.757149	-2.939017
H	-3.787097	11.242115	-3.162159
C	-1.819052	13.687408	-2.224674
H	-1.912681	14.707999	-1.881285
C	-0.542201	13.106531	-2.366409
H	0.326722	13.709271	-2.135461
C	4.318307	12.024745	-2.868998
C	4.295842	13.371688	-2.427936
H	3.365961	13.886326	-2.225450
C	5.502435	14.081343	-2.269458
H	5.480372	15.112052	-1.945683
C	5.601149	11.473760	-3.116423
H	5.724792	10.454275	-3.459067
C	6.754604	12.259014	-2.920929
H	7.730321	11.834383	-3.110212
C	11.171670	14.494408	-0.748573
H	10.766166	13.498348	-0.947126
H	12.147433	14.542291	-1.248116

H	11.374615	14.558758	0.327759
C	9.814818	16.430237	-0.105740
H	8.728522	16.405103	0.018761
H	10.262224	16.145635	0.854842
H	10.088575	17.479325	-0.273693
C	10.802427	16.316935	-2.350210
H	10.887946	17.378773	-2.085994
H	11.836452	16.006966	-2.548492
C	10.061850	16.263670	-3.709267
H	9.856247	17.304594	-3.990042
H	10.797896	15.927593	-4.450789
C	7.636966	16.285516	-4.090097
H	6.944755	16.283164	-3.243525
H	7.894667	17.334361	-4.283445
H	7.081442	15.938211	-4.970175
C	8.964967	14.345346	-4.777127
H	8.167696	14.345559	-5.530873
H	9.910767	14.388612	-5.331445
H	8.939720	13.374635	-4.273791
N	-4.447606	1.041816	5.883648
N	-6.237695	3.173250	6.754355
C	-4.388216	0.463831	4.513574
H	-3.463610	-0.097203	4.326047
H	-5.214088	-0.226675	4.299640
H	-4.448672	1.259654	3.767283
C	-3.255859	0.644693	6.680200
H	-2.545759	0.031056	6.110868
H	-2.721240	1.536221	7.021215
H	-3.509928	0.067932	7.578453
C	-5.709319	0.638343	6.572256
H	-5.492204	0.060231	7.479869
H	-6.293146	-0.051750	5.948968
C	-6.679688	1.768713	6.995266
H	-7.637723	1.565032	6.498469
H	-6.891136	1.606802	8.060442
C	-7.225753	3.902438	5.914125
H	-7.574583	4.834239	6.377383
H	-6.779494	4.163217	4.950061
H	-8.126111	3.313372	5.696610
C	-6.004565	3.884434	8.039868
H	-6.571075	4.821053	8.119705
H	-6.274130	3.285991	8.919297
H	-4.943406	4.129582	8.142513
N	9.455749	1.718025	6.095440
N	10.580284	4.293479	6.881227
N	10.218823	15.533839	-1.221760
N	8.819824	15.439263	-3.779850
N	-6.752064	14.776048	-1.769026
N	-5.027506	14.715960	-4.124749
N	5.744055	12.150745	4.212189
N	5.871549	10.237259	5.671085
N	7.132648	10.277406	3.621721
N	8.187244	5.727760	5.516027
N	2.547494	13.300211	7.949459
N	2.323837	3.050207	-3.723198
N	3.496944	3.060033	1.187599
N	1.089927	2.989078	1.161008
N	2.273835	2.971018	3.255351
N	-2.570536	3.171031	4.626370
N	7.091520	3.185549	4.744101
N	-4.638674	13.108062	-0.230481
N	-3.768974	9.912497	3.591862
N	-2.825390	10.085407	5.800008
N	-2.792976	11.987579	4.325670
N	-0.250943	13.586254	8.392598
N	2.250086	5.391135	-5.324208
N	0.847789	9.881060	-3.494957
N	1.951931	11.886009	-2.752469
N	3.249659	10.004725	-3.510605
N	6.715833	13.544223	-2.511486
N	-2.962648	13.031973	-2.512615
Pd	1.425499	14.343177	9.404992
Pd	-4.840126	13.906497	-2.159401
Pd	8.821046	3.736908	5.810790
Pd	8.464058	14.525106	-1.886944
Pd	2.329684	3.347937	-5.806580
Pd	-4.389296	3.168636	5.685945
C	2.434783	5.449119	6.158306
C	2.734375	5.750134	4.836037
C	1.687786	6.155970	3.950055
C	0.402151	6.468831	4.495185
C	0.212367	6.390947	5.911800
C	1.161506	5.785037	6.703142
H	2.865085	6.031986	2.148513
H	3.196079	5.053012	6.816764
H	3.735835	5.642607	4.424217
C	1.867947	6.219669	2.542483
C	-0.638917	6.809124	3.599369

H	-0.713582	6.769707	6.346590
H	0.968035	5.641517	7.759422
C	-0.473490	6.807602	2.227433
C	0.817448	6.499025	1.681169
H	-1.610040	7.061641	4.015482
C	-1.653924	7.154063	1.343038
C	-2.461721	5.913067	0.932478
H	-1.316882	7.695308	0.453713
H	-2.316631	7.845253	1.880139
H	-3.301593	6.173732	0.280547
H	-1.847790	5.175857	0.404213
H	-2.861496	5.426722	1.829134
C	1.078520	6.543520	0.185812
C	1.434076	7.946915	-0.332325
H	0.214194	6.156813	-0.370286
H	1.919176	5.878218	-0.038785
H	1.709628	7.896484	-1.391434
H	0.587689	8.631852	-0.236110
H	2.280877	8.364997	0.223463
C	3.177540	9.067632	4.115884
C	3.250291	8.552340	5.525000
C	2.148769	8.989805	6.196259
C	1.354385	9.839127	5.244814
H	4.108616	8.069340	5.860765
H	1.906789	8.909934	7.222618
C	1.631680	10.610710	2.816155
C	0.177933	10.360609	2.385046
C	1.900717	12.103942	3.070476
H	2.291875	10.259272	2.017325
C	-0.179662	11.214010	1.157757
H	-0.489041	10.615840	3.216703
H	0.051607	9.295308	2.181889
C	1.510344	12.954991	1.852532
H	1.315331	12.413128	3.942287
H	2.959649	12.244117	3.313060
C	0.054910	12.704448	1.437735
H	-1.220812	11.023699	0.869045
H	0.449419	10.917492	0.306114
H	1.670343	14.014931	2.067143
H	2.168421	12.701871	1.009355
H	-0.190825	13.309449	0.558169
H	-0.613518	13.029267	2.247256
N	2.008179	9.808694	3.995978
O	0.374271	10.501557	5.499395
O	4.032174	8.910070	3.260175
O	5.148995	6.386408	2.591631
H	5.464771	6.418085	1.679220
H	4.745865	7.260982	2.728371

Table5_1c_TSiii_TSi-ii_h2o

Property	Value
Charge	0
Electronic Energy, BS1 (a.u.)	-1213.976762
Thermal and entropic correction, BS1 (a.u.)	2.831730
Electronic Energy, BS2 (a.u.)	-1214.388474
Number of Imaginary Frequencies	0
Imaginary frequencies (cm-1)	None

Molecular Geometry in Cartesian Coordinates

C	8.400712	1.058841	6.906613
H	8.783551	0.610562	7.831983
H	7.642716	1.789703	7.202182
H	7.888666	0.254339	6.364005
C	9.568811	1.095605	4.753055
H	8.841460	0.289167	4.596050
H	9.400358	1.843962	3.973628
H	10.556901	0.658847	4.561628
C	10.769059	1.697291	6.807431
H	11.528763	1.177595	6.209681
H	10.702390	1.104173	7.728544
C	11.384748	3.059515	7.210193
H	12.391830	3.085769	6.774578
H	11.562956	3.009369	8.292005
C	11.412605	5.168832	5.957364
H	10.932292	5.234873	4.977489
H	11.516983	6.191012	6.342093
H	12.431589	4.800962	5.783543
C	10.194470	5.023523	8.077807
H	9.104150	5.010183	8.160167
H	10.586223	4.583385	9.003331
H	10.512211	6.073724	8.077697
N	3.135573	14.989127	10.420214

N	0.267696	15.291612	10.867196
C	3.898435	13.809049	10.905787
H	3.335559	12.893620	10.704381
H	4.879848	13.707403	10.426055
H	4.085512	13.830810	11.986706
C	3.932142	15.770225	9.436628
H	3.384097	15.858910	8.495168
H	4.146953	16.793124	9.770690
H	4.903087	15.311092	9.212556
C	2.707666	15.852109	11.560139
H	3.126283	15.485983	12.506372
H	3.117687	16.865314	11.460990
C	1.185790	16.017595	11.792782
H	0.984210	17.096409	11.778651
H	0.999800	15.724737	12.834088
C	-0.614927	16.242301	10.139452
H	-1.681187	16.012307	10.258602
H	-0.487445	17.283161	10.462259
H	-0.392723	16.215123	9.068703
C	-0.535335	14.276587	11.599396
H	-0.267200	13.272655	11.258348
H	-0.370683	14.296047	12.683969
H	-1.615729	14.396394	11.449453
N	2.402750	1.250038	-6.170191
N	2.396336	3.632715	-7.854833
C	3.624908	0.700203	-5.525996
H	3.400970	-0.009922	-4.720117
H	4.282332	0.171905	-6.227751
H	4.215847	1.512546	-5.093595
C	1.175425	0.653460	-5.579420
H	1.391212	-0.052076	-4.767342
H	0.537444	1.441578	-5.169619
H	0.567755	0.104692	-6.309741
C	2.438799	1.043443	-7.647655
H	1.590143	0.430751	-7.977905
H	3.320525	0.458478	-7.939242
C	2.438833	2.308164	-8.541575
H	3.322623	2.230588	-9.187761
H	1.592697	2.198440	-9.231970
C	1.164689	4.381807	-8.220503
H	1.374785	5.385894	-8.609803
H	0.563150	3.875819	-8.985925
H	0.522727	4.497685	-7.342850
C	3.613790	4.429653	-8.161261
H	3.383772	5.421396	-8.570393
H	4.201827	4.577413	-7.250944
H	4.275994	3.943974	-8.888798
C	-6.551473	15.766449	-0.660072
H	-7.092079	15.478766	0.250356
H	-5.491663	15.842424	-0.402755
H	-6.887848	16.780716	-0.909495
C	-7.647197	13.694235	-1.372423
H	-7.996002	13.783446	-0.335865
H	-8.548955	13.632537	-1.994314
H	-7.134585	12.732534	-1.464365
C	-7.233167	15.483655	-3.001266
H	-7.458291	16.537874	-2.794698
H	-8.194928	15.063139	-3.321956
C	-6.311546	15.471249	-4.246145
H	-6.912937	15.067591	-5.070742
H	-6.149922	16.520949	-4.522950
C	-4.950666	13.613046	-5.093558
H	-4.065471	13.649420	-5.740770
H	-4.923981	12.663327	-4.552600
H	-5.818521	13.568005	-5.763448
C	-3.865852	15.671996	-4.328369
H	-3.177797	15.339385	-5.115669
H	-4.174751	16.688958	-4.600732
H	-3.289457	15.755584	-3.402707
N	8.053745	13.651258	-0.031455
C	7.155921	14.197692	0.815046
H	6.837484	15.208316	0.602550
C	6.640558	13.523078	1.940067
H	5.934460	14.041863	2.575017
C	7.068722	12.203556	2.231280
C	8.009243	11.643115	1.330432
H	8.402172	10.643737	1.467000
C	8.462383	12.392482	0.227884
H	9.179633	11.960140	-0.455049
C	6.592894	11.483784	3.368844
C	5.344975	11.397544	5.262706
C	4.387365	11.993264	6.140521
C	3.843366	13.280127	5.896379
H	4.130561	13.863430	5.030967
C	2.921120	13.839924	6.802123
H	2.512003	14.823301	6.618101
C	3.000931	11.978884	8.160324
H	2.651575	11.480556	9.053448

C	3.929095	11.339855	7.312890
H	4.286385	10.356243	7.587757
C	6.677284	9.647303	4.698828
C	7.187699	8.340540	4.965381
C	6.879320	7.637670	6.157722
H	6.261431	8.076786	6.929260
C	8.039467	7.669721	4.051221
H	8.334551	8.121039	3.113003
C	8.518487	6.379886	4.350341
H	9.161460	5.868431	3.648635
C	7.409438	6.349829	6.373453
H	7.179124	5.817241	7.284590
C	3.468881	3.115580	-2.938713
H	4.395325	3.130783	-3.495201
C	3.497507	3.124780	-1.530808
H	4.461448	3.138336	-1.038396
C	1.162902	3.062211	-2.970847
H	0.254045	3.031658	-3.554641
C	1.093324	3.071781	-1.563023
H	0.118274	3.041617	-1.094152
C	2.285348	3.100432	-0.795433
C	2.269643	3.102508	0.632554
C	1.121558	3.104632	2.590387
C	3.390998	3.130477	2.605714
C	4.624223	3.153495	3.323547
C	4.671962	3.097672	4.738834
H	3.770540	3.007733	5.330667
C	5.875281	3.235459	2.662173
H	5.946247	3.273401	1.583224
C	7.066230	3.273567	3.412153
H	8.017852	3.340795	2.904599
C	5.913726	3.140481	5.402917
H	5.947159	3.094738	6.481636
C	-0.120817	3.143177	3.292750
C	-1.363367	3.072370	2.614813
H	-1.417972	2.976146	1.538319
C	-2.566760	3.103763	3.344552
H	-3.512862	3.047460	2.825063
C	-1.440299	3.281862	5.351147
H	-1.491068	3.367004	6.427035
C	-0.187427	3.252586	4.705328
H	0.708461	3.314513	5.309673
C	-3.944672	13.774077	0.706728
H	-3.722419	14.814279	0.516307
C	-4.910819	11.811584	-0.018684
H	-5.460685	11.288644	-0.788681
C	-4.542845	11.134953	1.161557
H	-4.830666	10.097516	1.272952
C	-3.839405	11.823422	2.181190
C	-3.538572	13.184025	1.918583
H	-3.017254	13.801511	2.638667
C	-3.474534	11.194198	3.409340
C	-3.499319	9.389917	4.782757
C	-2.543424	11.330274	5.474170
N	-4.393656	5.258328	5.513111
C	-3.812377	8.017790	5.026747
C	-4.456240	7.204315	4.060080
H	-4.764764	7.601079	3.101298
C	-4.722882	5.849258	4.345120
H	-5.222615	5.232507	3.610969
C	-3.476613	7.381343	6.248727
H	-2.996308	7.919193	7.056024
C	-3.777534	6.019305	6.440251
H	-3.520359	5.540193	7.373385
C	-1.821501	12.071792	6.456731
C	-1.456617	11.505723	7.702862
H	-1.742643	10.497621	7.970834
C	-0.689045	12.252759	8.616115
H	-0.408203	11.810764	9.560884
C	-0.636225	14.071984	7.211802
H	-0.316691	15.089531	7.038285
C	-1.394149	13.403321	6.231100
H	-1.636055	13.928505	5.316684
C	3.393978	6.084952	-5.019016
H	4.336161	5.589859	-5.207176
C	1.090098	5.997264	-5.064645
H	0.197096	5.432891	-5.293112
C	0.980700	7.306923	-4.558202
H	-0.008629	7.722624	-4.419013
C	3.383025	7.401123	-4.515351
H	4.331291	7.890256	-4.330941
C	2.149692	8.055717	-4.269520
C	2.086157	9.389948	-3.764086
C	0.858983	11.167512	-3.064287
C	3.124832	11.288041	-3.078300
C	-0.411120	11.785317	-2.855891
C	-1.625791	11.110798	-3.138361
H	-1.638871	10.095840	-3.514183

C	-2.856046	11.772611	-2.962113
H	-3.780571	11.260182	-3.187752
C	-1.809008	13.697793	-2.236830
H	-1.901462	14.716442	-1.887423
C	-0.533334	13.116507	-2.381765
H	0.336249	13.716686	-2.146725
C	4.321787	12.042016	-2.886901
C	4.296484	13.389180	-2.446323
H	3.366019	13.903659	-2.245444
C	5.501733	14.099797	-2.285954
H	5.478088	15.130324	-1.961791
C	5.605450	11.492517	-3.132716
H	5.730320	10.473140	-3.475282
C	6.757568	12.279336	-2.936030
H	7.734189	11.856141	-3.123766
C	11.158084	14.511000	-0.731304
H	10.755488	13.515390	-0.937746
H	12.137273	14.562840	-1.223701
H	11.353354	14.570269	0.346746
C	9.793435	16.441186	-0.088249
H	8.706242	16.413705	0.027964
H	10.233929	16.151990	0.874108
H	10.066937	17.491517	-0.248591
C	10.797545	16.340920	-2.326331
H	10.879548	17.401556	-2.056255
H	11.833435	16.033432	-2.518650
C	10.066841	16.293215	-3.691027
H	9.862129	17.335245	-3.968318
H	10.808464	15.961363	-4.428890
C	7.644632	16.314868	-4.088120
H	6.946741	16.307443	-3.246285
H	7.902657	17.364939	-4.274232
H	7.095305	15.971710	-4.973701
C	8.978696	14.379329	-4.776232
H	8.186355	14.382757	-5.535168
H	9.928014	14.426274	-5.324209
H	8.950936	13.405989	-4.278038
N	-4.477318	1.041487	5.926541
N	-6.314626	3.151800	6.747189
C	-4.385285	0.453801	4.562988
H	-3.449578	-0.094236	4.396284
H	-5.196372	-0.251005	4.340991
H	-4.444707	1.242339	3.808853
C	-3.293718	0.669313	6.746131
H	-2.565015	0.062358	6.194337
H	-2.778580	1.571527	7.088483
H	-3.554118	0.095763	7.644500
C	-5.743739	0.624050	6.597072
H	-5.533371	0.056772	7.512885
H	-6.305852	-0.079986	5.970251
C	-6.738187	1.742540	6.993638
H	-7.683727	1.519268	6.482834
H	-6.965173	1.586577	8.056147
C	-7.298348	3.857530	5.883105
H	-7.669717	4.787758	6.330764
H	-6.838987	4.116469	4.925048
H	-8.184984	3.252453	5.655599
C	-6.115942	3.878687	8.028749
H	-6.698765	4.806338	8.089333
H	-6.391044	3.284133	8.908860
H	-5.061574	4.142749	8.147071
N	9.457011	1.722838	6.097425
N	10.617603	4.289629	6.855889
N	10.207004	15.551388	-1.205959
N	8.826144	15.468021	-3.774319
N	-6.734209	14.797787	-1.773504
N	-5.012227	14.745451	-4.131266
N	5.682997	12.100019	4.157809
N	5.808140	10.170649	5.595785
N	7.088379	10.241977	3.558157
N	8.218729	5.728107	5.491749
N	2.508944	13.212098	7.921894
N	2.326376	3.078779	-3.653698
N	3.465516	3.171490	1.256717
N	1.061508	3.092174	1.238420
N	2.251434	3.114178	3.333622
N	-2.612426	3.202333	4.687849
N	7.096709	3.225165	4.759633
N	-4.626000	13.110373	-0.249196
N	-3.798511	9.888826	3.561199
N	-2.868633	10.052759	5.775463
N	-2.801518	11.952280	4.302004
N	-0.272773	13.515732	8.386703
N	2.271411	5.390072	-5.297667
N	0.855808	9.896516	-3.523395
N	1.956868	11.900269	-2.774754
N	3.257881	10.020671	-3.529945
N	6.716063	13.564269	-2.526277

N	-2.953017	13.045365	-2.528818
Pd	1.410313	14.248379	9.396975
Pd	-4.826407	13.922951	-2.171365
Pd	8.845866	3.746279	5.801880
Pd	8.458789	14.543881	-1.888723
Pd	2.353402	3.340003	-5.743164
Pd	-4.448752	3.166701	5.712531
C	2.661441	5.660948	6.058115
C	2.942124	6.170560	4.747151
C	1.812025	6.359993	3.854216
C	0.592798	6.728424	4.460562
C	0.623554	6.899731	5.901193
C	1.486787	6.035571	6.650565
H	2.874111	6.110331	2.009662
H	3.429541	5.142584	6.621799
H	3.899182	5.955752	4.281408
C	1.904167	6.317261	2.455199
C	-0.513545	7.002790	3.644624
H	-0.266465	7.299422	6.380044
H	1.285190	5.830697	7.696253
C	-0.443711	6.910560	2.258384
C	0.794840	6.562665	1.651686
H	-1.442406	7.312861	4.110589
C	-1.678429	7.225784	1.437517
C	-2.527698	5.988808	1.113502
H	-1.396605	7.720533	0.504882
H	-2.290277	7.951434	1.985667
H	-3.393359	6.247690	0.499539
H	-1.945480	5.239782	0.569446
H	-2.888163	5.526558	2.038277
C	0.973661	6.547118	0.148296
C	1.304636	7.939800	-0.414381
H	0.084447	6.147531	-0.347942
H	1.799678	5.874025	-0.101766
H	1.515583	7.874294	-1.484167
H	0.475328	8.638540	-0.275359
H	2.184407	8.354740	0.085735
C	3.158199	8.922931	3.988125
C	3.157817	8.192277	5.293130
C	1.986757	8.594341	5.986869
C	1.301257	9.610454	5.147528
H	4.108572	8.026310	5.776233
H	1.916972	8.692918	7.060097
C	1.631841	10.530593	2.782689
C	0.179169	10.316053	2.331662
C	1.910161	12.009012	3.100047
H	2.302495	10.209832	1.978936
C	-0.164727	11.218660	1.139462
H	-0.484850	10.541678	3.170844
H	0.038602	9.266678	2.079023
C	1.542430	12.907467	1.909840
H	1.319018	12.289788	3.980034
H	2.968546	12.129053	3.353618
C	0.086958	12.693975	1.473440
H	-1.207549	11.052598	0.841260
H	0.454412	10.934956	0.276270
H	1.716072	13.956203	2.164824
H	2.204508	12.675183	1.064345
H	-0.145876	13.333304	0.615050
H	-0.580939	13.000621	2.289680
N	1.987883	9.670483	3.922612
O	0.335957	10.294754	5.434057
O	4.008775	8.903894	3.105198
O	5.320633	6.452248	2.644804
H	5.586744	6.416353	1.717605
H	4.990238	7.362887	2.763477

Table5_1c_reactant_1wat

Property	Value
Charge	0
Electronic Energy, BS1 (a.u.)	-1214.016793
Thermal and entropic correction, BS1 (a.u.)	2.827257
Electronic Energy, BS2 (a.u.)	-1214.432006
Number of Imaginary Frequencies	0
Imaginary frequencies (cm-1)	None

Molecular Geometry in Cartesian Coordinates

C	8.401132	1.034535	6.890766
H	8.782109	0.583704	7.815733
H	7.631725	1.753623	7.186160
H	7.903408	0.227966	6.337584
C	9.586990	1.102222	4.747266

H	8.871228	0.287661	4.578088
H	9.415698	1.853951	3.971870
H	10.582285	0.679395	4.560950
C	10.762548	1.703236	6.815729
H	11.533234	1.196996	6.220210
H	10.695937	1.102699	7.732027
C	11.359131	3.069760	7.232627
H	12.369108	3.110548	6.804552
H	11.530138	3.014199	8.315377
C	11.371575	5.187992	5.994406
H	10.898051	5.254982	5.011306
H	11.461051	6.208853	6.386756
H	12.396211	4.833194	5.825776
C	10.140840	5.013766	8.105749
H	9.050176	4.987894	8.180952
H	10.531259	4.571091	9.030652
H	10.447300	6.067421	8.115583
N	3.137889	15.110185	10.431464
N	0.264481	15.391235	10.857808
C	3.907973	13.942434	10.935349
H	3.354304	13.019893	10.741468
H	4.893241	13.843690	10.462817
H	4.088162	13.978207	12.017096
C	3.933931	15.887336	9.444342
H	3.391369	15.960460	8.498328
H	4.137275	16.915848	9.768336
H	4.910463	15.434551	9.231834
C	2.695058	15.981453	11.559414
H	3.110948	15.629256	12.512122
H	3.096588	16.997255	11.452028
C	1.170336	16.135168	11.781278
H	0.958923	17.211884	11.754845
H	0.980809	15.851344	12.824483
C	-0.622278	16.326621	10.115242
H	-1.687143	16.088226	10.230558
H	-0.506105	17.371803	10.428246
H	-0.393663	16.291004	9.046111
C	-0.533818	14.376461	11.595575
H	-0.254632	13.371506	11.266478
H	-0.375792	14.408526	12.680858
H	-1.614342	14.485004	11.438096
N	2.376968	1.265358	-6.269861
N	2.346414	3.676515	-7.913371
C	3.607080	0.706520	-5.648659
H	3.393171	-0.017717	-4.852486
H	4.257510	0.191335	-6.366709
H	4.201502	1.512367	-5.208844
C	1.157143	0.656737	-5.675697
H	1.383044	-0.062089	-4.877937
H	0.522317	1.436778	-5.245664
H	0.542436	0.119222	-6.408597
C	2.396922	1.083985	-7.751043
H	1.545798	0.475179	-8.082205
H	3.276485	0.505870	-8.062536
C	2.384119	2.363851	-8.623144
H	3.260634	2.299387	-9.280721
H	1.530356	2.263881	-9.305628
C	1.108564	4.428488	-8.251478
H	1.311469	5.439769	-8.625993
H	0.499266	3.934045	-9.018367
H	0.476722	4.527449	-7.364342
C	3.558260	4.481590	-8.220918
H	3.321000	5.479595	-8.610538
H	4.157009	4.615328	-7.315312
H	4.212768	4.009863	-8.964615
C	-6.574709	15.753017	-0.661934
H	-7.114711	15.468913	0.249997
H	-5.515537	15.835761	-0.404288
H	-6.915620	16.764031	-0.918406
C	-7.660594	13.671160	-1.361488
H	-8.010828	13.765743	-0.325867
H	-8.561438	13.601404	-1.983898
H	-7.143654	12.711069	-1.446510
C	-7.252709	15.451836	-3.001708
H	-7.482501	16.506418	-2.802159
H	-8.212358	15.025183	-3.320645
C	-6.329726	15.435563	-4.245496
H	-6.928573	15.024186	-5.068153
H	-6.172238	16.484194	-4.528755
C	-4.960570	13.577890	-5.080036
H	-4.074899	13.613820	-5.726636
H	-4.930739	12.631606	-4.533324
H	-5.827552	13.525284	-5.750512
C	-3.884631	15.645826	-4.326490
H	-3.194539	15.311122	-5.111120
H	-4.197351	16.659891	-4.605334
H	-3.309424	15.737317	-3.400784
N	8.073701	13.646537	-0.018816

C	7.187761	14.204204	0.832798
H	6.870160	15.213665	0.613678
C	6.684812	13.543736	1.971641
H	5.988828	14.072231	2.609692
C	7.112160	12.226148	2.272453
C	8.039880	11.653137	1.365728
H	8.431228	10.654045	1.507887
C	8.481977	12.389318	0.249258
H	9.189734	11.947517	-0.437496
C	6.645554	11.521409	3.423657
C	5.412619	11.464860	5.329047
C	4.452293	12.072424	6.197701
C	3.896777	13.350635	5.931469
H	4.181633	13.922196	5.057441
C	2.961940	13.915287	6.823076
H	2.542957	14.890986	6.621265
C	3.054459	12.077744	8.210557
H	2.705745	11.588734	9.108981
C	3.996206	11.436668	7.379967
H	4.362839	10.461741	7.673035
C	6.723143	9.692878	4.769002
C	7.204205	8.371121	5.026490
C	6.885032	7.659788	6.213290
H	6.283448	8.112358	6.994566
C	8.035742	7.690852	4.100347
H	8.335845	8.146641	3.165911
C	8.493108	6.389388	4.382287
H	9.126109	5.876764	3.672407
C	7.389089	6.354912	6.408304
H	7.154953	5.817282	7.315456
C	3.473857	3.073831	-3.018905
H	4.394783	3.098458	-3.584287
C	3.516274	3.057893	-1.611099
H	4.484469	3.062414	-1.126502
C	1.167325	3.021970	-3.030782
H	0.253097	3.002538	-3.606662
C	1.111301	3.007328	-1.623327
H	0.140505	2.970025	-1.146134
C	2.310263	3.021702	-0.866524
C	2.300295	3.000376	0.560388
C	1.147039	2.982973	2.510940
C	3.417313	3.000361	2.536694
C	4.642377	3.042529	3.269755
C	4.673212	2.981832	4.686614
H	3.765885	2.866773	5.265876
C	5.899657	3.162636	2.626873
H	5.984057	3.208816	1.549199
C	7.078052	3.235049	3.395473
H	8.034695	3.331665	2.901909
C	5.903143	3.062137	5.369828
H	5.922460	3.014863	6.448441
C	-0.093089	3.046309	3.213218
C	-1.339717	3.011736	2.538396
H	-1.387688	2.918618	1.457556
C	-2.539291	3.073603	3.279105
H	-3.490956	3.042403	2.767449
C	-1.392631	3.216332	5.279480
H	-1.433473	3.300338	6.355851
C	-0.147738	3.152867	4.625747
H	0.752671	3.189411	5.225460
C	-3.962723	13.782336	0.721707
H	-3.747709	14.822973	0.524929
C	-4.913766	11.808645	0.008466
H	-5.459871	11.276732	-0.758090
C	-4.539071	11.141936	1.192566
H	-4.818492	10.102705	1.309244
C	-3.839021	11.841478	2.207605
C	-3.551273	13.202784	1.936773
H	-3.032948	13.831539	2.648434
C	-3.462853	11.221087	3.437128
C	-3.454295	9.414796	4.809940
C	-2.521332	11.369463	5.498052
N	-4.319537	5.267612	5.513566
C	-3.751002	8.037200	5.046964
C	-4.400317	7.229013	4.079299
H	-4.723337	7.633241	3.127554
C	-4.661074	5.871506	4.355366
H	-5.171784	5.262653	3.622517
C	-3.396383	7.388562	6.258557
H	-2.908016	7.921024	7.065259
C	-3.689102	6.021734	6.441538
H	-3.416375	5.538730	7.371473
C	-1.802583	12.121928	6.476677
C	-1.428313	11.565674	7.725292
H	-1.707951	10.557655	8.000654
C	-0.660465	12.322548	8.631360
H	-0.374095	11.888175	9.577958
C	-0.622751	14.132823	7.215476

H	-0.309716	15.151096	7.034204
C	-1.383380	13.454819	6.242594
H	-1.631552	13.973481	5.326092
C	3.375176	6.081497	-5.043464
H	4.315576	5.589010	-5.246975
C	1.071136	5.995830	-5.072213
H	0.175880	5.435690	-5.302295
C	0.966408	7.298963	-4.547420
H	-0.021559	7.714103	-4.396840
C	3.368848	7.390709	-4.520728
H	4.318976	7.876859	-4.338127
C	2.137666	8.043725	-4.258137
C	2.076899	9.374141	-3.741191
C	0.852536	11.153401	-3.038691
C	3.119210	11.272370	-3.058054
C	-0.417940	11.772621	-2.832946
C	-1.632237	11.096099	-3.112273
H	-1.644077	10.079381	-3.483220
C	-2.863402	11.757149	-2.939017
H	-3.787097	11.242115	-3.162159
C	-1.819052	13.687408	-2.224674
H	-1.912681	14.707999	-1.881285
C	-0.542201	13.106531	-2.366409
H	0.326722	13.709271	-2.135461
C	4.318307	12.024745	-2.868998
C	4.295842	13.371688	-2.427936
H	3.365961	13.886326	-2.225450
C	5.502435	14.081343	-2.269458
H	5.480372	15.112052	-1.945683
C	5.601149	11.473760	-3.116423
H	5.724792	10.454275	-3.459067
C	6.754604	12.259014	-2.920929
H	7.730321	11.834383	-3.110212
C	11.171670	14.494408	-0.748573
H	10.766166	13.498348	-0.947126
H	12.147433	14.542291	-1.248116
H	11.374615	14.558758	0.327759
C	9.814818	16.430237	-0.105740
H	8.728522	16.405103	0.018761
H	10.262224	16.145635	0.854842
H	10.088575	17.479325	-0.273693
C	10.802427	16.316935	-2.350210
H	10.887946	17.378773	-2.085994
H	11.836452	16.006966	-2.548492
C	10.061850	16.263670	-3.709267
H	9.856247	17.304594	-3.990042
H	10.797896	15.927593	-4.450789
C	7.636966	16.285516	-4.090097
H	6.944755	16.283164	-3.243525
H	7.894667	17.334361	-4.283445
H	7.081442	15.938211	-4.970175
C	8.964967	14.345346	-4.777127
H	8.167696	14.345559	-5.530873
H	9.910767	14.388612	-5.331445
H	8.939720	13.374635	-4.273791
N	-4.447606	1.041816	5.883648
N	-6.237695	3.173250	6.754355
C	-4.388216	0.463831	4.513574
H	-3.463610	-0.097203	4.326047
H	-5.214088	-0.226675	4.299640
H	-4.448672	1.259654	3.767283
C	-3.255859	0.644693	6.680200
H	-2.545759	0.031056	6.110868
H	-2.721240	1.536221	7.021215
H	-3.509928	0.067932	7.578453
C	-5.709319	0.638343	6.572256
H	-5.492204	0.060231	7.479869
H	-6.293146	-0.051750	5.948968
C	-6.679688	1.768713	6.995266
H	-7.637723	1.565032	6.498469
H	-6.891136	1.606802	8.060442
C	-7.225753	3.902438	5.914125
H	-7.574583	4.834239	6.377383
H	-6.779494	4.163217	4.950061
H	-8.126111	3.313372	5.696610
C	-6.004565	3.884434	8.039868
H	-6.571075	4.821053	8.119705
H	-6.274130	3.285991	8.919297
H	-4.943406	4.129582	8.142513
N	9.455749	1.718025	6.095440
N	10.580284	4.293479	6.881227
N	10.218823	15.533839	-1.221760
N	8.819824	15.439263	-3.779850
N	-6.752064	14.776048	-1.769026
N	-5.027506	14.715960	-4.124749
N	5.744055	12.150745	4.212189
N	5.871549	10.237259	5.671085
N	7.132648	10.277406	3.621721

N	8.187244	5.727760	5.516027
N	2.547494	13.300211	7.949459
N	2.323837	3.050207	-3.723198
N	3.496944	3.060033	1.187599
N	1.089927	2.989078	1.161008
N	2.273835	2.971018	3.255351
N	-2.570536	3.171031	4.626370
N	7.091520	3.185549	4.744101
N	-4.638674	13.108062	-0.230481
N	-3.768974	9.912497	3.591862
N	-2.825390	10.085407	5.800008
N	-2.792976	11.987579	4.325670
N	-0.250943	13.586254	8.392598
N	2.250086	5.391135	-5.324208
N	0.847789	9.881060	-3.494957
N	1.951931	11.886009	-2.752469
N	3.249659	10.004725	-3.510605
N	6.715833	13.544223	-2.511486
N	-2.962648	13.031973	-2.512615
Pd	1.425499	14.343177	9.404992
Pd	-4.840126	13.906497	-2.159401
Pd	8.821046	3.736908	5.810790
Pd	8.464058	14.525106	-1.886944
Pd	2.329684	3.347937	-5.806580
Pd	-4.389296	3.168636	5.685945
C	2.434783	5.449119	6.158306
C	2.734375	5.750134	4.836037
C	1.687786	6.155970	3.950055
C	0.402151	6.468831	4.495185
C	0.212367	6.390947	5.911800
C	1.161506	5.785037	6.703142
H	2.865085	6.031986	2.148513
H	3.196079	5.053012	6.816764
H	3.735835	5.642607	4.424217
C	1.867947	6.219669	2.542483
C	-0.638917	6.809124	3.599369
H	-0.713582	6.769707	6.346590
H	0.968035	5.641517	7.759422
C	-0.473490	6.807602	2.227433
C	0.817448	6.499025	1.681169
H	-1.610040	7.061641	4.015482
C	-1.653924	7.154063	1.343038
C	-2.461721	5.913067	0.932478
H	-1.316882	7.695308	0.453713
H	-2.316631	7.845253	1.880139
H	-3.301593	6.173732	0.280547
H	-1.847790	5.175857	0.404213
H	-2.861496	5.426722	1.829134
C	1.078520	6.543520	0.185812
C	1.434076	7.946915	-0.332325
H	0.214194	6.156813	-0.370286
H	1.919176	5.878218	-0.038785
H	1.709628	7.896484	-1.391434
H	0.587689	8.631852	-0.236110
H	2.280877	8.364997	0.223463
C	3.177540	9.067632	4.115884
C	3.250291	8.552340	5.525000
C	2.148769	8.989805	6.196259
C	1.354385	9.839127	5.244814
H	4.108616	8.069340	5.860765
H	1.906789	8.909934	7.222618
C	1.631680	10.610710	2.816155
C	0.177933	10.360609	2.385046
C	1.900717	12.103942	3.070476
H	2.291875	10.259272	2.017325
C	-0.179662	11.214010	1.157757
H	-0.489041	10.615840	3.216703
H	0.051607	9.295308	2.181889
C	1.510344	12.954991	1.852532
H	1.315331	12.413128	3.942287
H	2.959649	12.244117	3.313060
C	0.054910	12.704448	1.437735
H	-1.220812	11.023699	0.869045
H	0.449419	10.917492	0.306114
H	1.670343	14.014931	2.067143
H	2.168421	12.701871	1.009355
H	-0.190825	13.309449	0.558169
H	-0.613518	13.029267	2.247256
N	2.008179	9.808694	3.995978
O	0.374271	10.501557	5.499395
O	4.032174	8.910070	3.260175
O	5.148995	6.386408	2.591631
H	5.464771	6.418085	1.679220
H	4.745865	7.260982	2.728371

Table5_1c_TSiii_DG_h2o

Property	Value
Charge	0
Electronic Energy, BS1 (a.u.)	-1214.020153
Thermal and entropic correction, BS1 (a.u.)	2.839546
Electronic Energy, BS2 (a.u.)	-1214.429460
Number of Imaginary Frequencies	0
Imaginary frequencies (cm-1)	None

Molecular Geometry in Cartesian Coordinates

C	8.401547	1.059228	6.907188
H	8.784435	0.611490	7.832800
H	7.643164	1.789857	7.202349
H	7.889950	0.254280	6.364818
C	9.569845	1.095772	4.753739
H	8.842916	0.288904	4.596972
H	9.401010	1.843790	3.974071
H	10.558170	0.659475	4.562476
C	10.769655	1.698574	6.808067
H	11.529523	1.178653	6.210719
H	10.703033	1.106027	7.729550
C	11.385033	3.061184	7.209998
H	12.391984	3.087556	6.774092
H	11.563554	3.011596	8.291784
C	11.411947	5.170113	5.956455
H	10.931625	5.235457	4.976535
H	11.515735	6.192530	6.340716
H	12.431144	4.802730	5.782854
C	10.194107	5.025022	8.077077
H	9.103801	5.011365	8.159536
H	10.586049	4.585283	9.002711
H	10.511540	6.075315	8.076632
N	3.135948	14.988859	10.419666
N	0.268205	15.291276	10.867660
C	3.899313	13.808784	10.904459
H	3.336452	12.893312	10.703216
H	4.880418	13.707407	10.424040
H	4.087120	13.830320	11.985256
C	3.931859	15.770440	9.435938
H	3.383390	15.859151	8.494728
H	4.146474	16.793334	9.770134
H	4.902859	15.311670	9.211369
C	2.708420	15.851358	11.560102
H	3.127327	15.484834	12.506054
H	3.118437	16.864595	11.461249
C	1.186621	16.016799	11.793290
H	0.985083	17.095626	11.779703
H	1.000943	15.723496	12.834527
C	-0.614298	16.242323	10.140250
H	-1.680601	16.012664	10.259675
H	-0.486380	17.283115	10.463100
H	-0.392396	16.215149	9.069436
C	-0.534968	14.276272	11.599741
H	-0.267040	13.272355	11.258496
H	-0.370240	14.295486	12.684309
H	-1.615350	14.396314	11.449886
N	2.402810	1.249933	-6.170113
N	2.396566	3.632830	-7.854426
C	3.624968	0.699965	-5.526032
H	3.401030	-0.010254	-4.720236
H	4.282343	0.171728	-6.227879
H	4.215957	1.512227	-5.093547
C	1.175483	0.653334	-5.579365
H	1.391279	-0.052333	-4.767404
H	0.537574	1.441428	-5.169402
H	0.567739	0.104708	-6.309731
C	2.438803	1.043530	-7.647607
H	1.590093	0.430951	-7.977925
H	3.320478	0.458533	-7.939289
C	2.438927	2.308371	-8.541356
H	3.322689	2.230801	-9.187579
H	1.592759	2.198819	-9.231739
C	1.165003	4.382098	-8.220016
H	1.375207	5.386214	-8.609185
H	0.563423	3.876269	-8.985511
H	0.523041	4.497931	-7.342359
C	3.614107	4.429690	-8.160707
H	3.384195	5.421501	-8.569731
H	4.202116	4.577283	-7.250343
H	4.276305	3.944044	-8.888271
C	-6.550825	15.766762	-0.659679
H	-7.091270	15.479039	0.250832
H	-5.490972	15.842735	-0.402521

H	-6.887277	16.781018	-0.909042
C	-7.646731	13.694618	-1.371945
H	-7.995481	13.783872	-0.335373
H	-8.548518	13.632936	-1.993797
H	-7.134179	12.732890	-1.463890
C	-7.232683	15.483999	-3.000830
H	-7.457719	16.538234	-2.794249
H	-8.194492	15.063556	-3.321473
C	-6.311133	15.471510	-4.245761
H	-6.912615	15.067892	-5.070310
H	-6.149449	16.521194	-4.522595
C	-4.950444	13.613192	-5.093221
H	-4.065309	13.649507	-5.740518
H	-4.923784	12.663482	-4.552253
H	-5.818350	13.568172	-5.763044
C	-3.865418	15.672077	-4.328155
H	-3.177467	15.339399	-5.115518
H	-4.174267	16.689067	-4.600472
H	-3.288924	15.755606	-3.402545
N	8.053388	13.651262	-0.032049
C	7.155442	14.197684	0.814335
H	6.837097	15.208340	0.601861
C	6.639848	13.523022	1.939228
H	5.933660	14.041798	2.574087
C	7.067917	12.203469	2.230440
C	8.008523	11.643029	1.329676
H	8.401405	10.643634	1.466247
C	8.461896	12.392442	0.227260
H	9.179246	11.960109	-0.455571
C	6.592031	11.483688	3.367980
C	5.344168	11.397452	5.261892
C	4.386653	11.993149	6.139825
C	3.842794	13.280116	5.895930
H	4.130023	13.863518	5.030596
C	2.920655	13.839862	6.801830
H	2.511621	14.823312	6.618004
C	3.000280	11.978551	8.159665
H	2.650869	11.480083	9.052692
C	3.928346	11.339580	7.312089
H	4.285499	10.355866	7.586767
C	6.676346	9.647148	4.697894
C	7.186791	8.340406	4.964493
C	6.878510	7.637638	6.156921
H	6.260728	8.076832	6.928509
C	8.038489	7.669516	4.050318
H	8.333512	8.120775	3.112051
C	8.517482	6.379675	4.349477
H	9.160344	5.868127	3.647737
C	7.408588	6.349793	6.372685
H	7.178303	5.817256	7.283858
C	3.468906	3.114907	-2.938364
H	4.395374	3.129874	-3.494818
C	3.497491	3.124237	-1.530461
H	4.461422	3.137662	-1.038024
C	1.162924	3.061990	-2.970570
H	0.254080	3.031553	-3.554392
C	1.093301	3.071719	-1.562747
H	0.118231	3.041791	-1.093901
C	2.285304	3.100207	-0.795122
C	2.269557	3.102423	0.632863
C	1.121429	3.105039	2.590672
C	3.390870	3.130459	2.606050
C	4.624108	3.153214	3.323862
C	4.671864	3.097992	4.739167
H	3.770432	3.008814	5.331085
C	5.875185	3.234279	2.662420
H	5.946143	3.271677	1.583452
C	7.066170	3.272153	3.412348
H	8.017796	3.338694	2.904716
C	5.913677	3.140479	5.403181
H	5.947136	3.095166	6.481919
C	-0.120977	3.143795	3.292971
C	-1.363490	3.073184	2.614949
H	-1.418047	2.976934	1.538455
C	-2.566923	3.104848	3.344598
H	-3.513000	3.048737	2.825044
C	-1.440586	3.282758	5.351273
H	-1.491419	3.367947	6.427157
C	-0.187671	3.253228	4.705544
H	0.708189	3.314990	5.309945
C	-3.944169	13.774061	0.707040
H	-3.721792	14.814237	0.516628
C	-4.910516	11.811688	-0.018439
H	-5.460400	11.288820	-0.788476
C	-4.542687	11.135000	1.161817
H	-4.830625	10.097591	1.273184
C	-3.839232	11.823384	2.181498
C	-3.538190	13.183945	1.918907

H	-3.016804	13.801352	2.639012
C	-3.474532	11.194125	3.409679
C	-3.499565	9.389841	4.783090
C	-2.543449	11.330079	5.474534
N	-4.394861	5.258452	5.513570
C	-3.812998	8.017810	5.027136
C	-4.456973	7.204426	4.060469
H	-4.765347	7.601204	3.101643
C	-4.723894	5.849431	4.345547
H	-5.223722	5.232761	3.611396
C	-3.477482	7.381350	6.249172
H	-2.997167	7.919142	7.056503
C	-3.778687	6.019378	6.440719
H	-3.521713	5.540265	7.373906
C	-1.821663	12.071611	6.457179
C	-1.456636	11.505462	7.703228
H	-1.742443	10.497269	7.971082
C	-0.689108	12.252514	8.616508
H	-0.408090	11.810432	9.561186
C	-0.636724	14.071926	7.212443
H	-0.317406	15.089562	7.039044
C	-1.394565	13.403243	6.231693
H	-1.636590	13.928488	5.317343
C	3.394181	6.084719	-5.018423
H	4.336345	5.589586	-5.206569
C	1.090290	5.997053	-5.063961
H	0.197277	5.432657	-5.292332
C	0.980927	7.306757	-4.557630
H	-0.008390	7.722485	-4.418449
C	3.383259	7.400932	-4.514863
H	4.331535	7.890073	-4.330526
C	2.149938	8.055556	-4.269040
C	2.086431	9.389828	-3.763713
C	0.859293	11.167467	-3.064044
C	3.125136	11.287993	-3.078201
C	-0.410785	11.785327	-2.855665
C	-1.625488	11.110822	-3.138029
H	-1.638611	10.095834	-3.513768
C	-2.855721	11.772681	-2.961772
H	-3.780270	11.260252	-3.187315
C	-1.808593	13.697886	-2.236690
H	-1.901009	14.716557	-1.887337
C	-0.532941	13.116562	-2.381654
H	0.336667	13.716742	-2.146702
C	4.322078	12.042062	-2.887102
C	4.296744	13.389322	-2.446843
H	3.366271	13.903785	-2.245970
C	5.501959	14.100065	-2.286813
H	5.478276	15.130668	-1.962900
C	5.605739	11.492602	-3.132985
H	5.730628	10.473154	-3.475334
C	6.757821	12.279572	-2.936678
H	7.734437	11.856413	-3.124514
C	11.157881	14.510829	-0.730939
H	10.755279	13.515276	-0.937654
H	12.137271	14.562654	-1.222937
H	11.352704	14.569955	0.347199
C	9.793271	16.441178	-0.088264
H	8.706071	16.413649	0.027809
H	10.233669	16.152094	0.874170
H	10.066685	17.491525	-0.248643
C	10.798021	16.340805	-2.326056
H	10.880393	17.401370	-2.055815
H	11.833821	16.032975	-2.518306
C	10.067436	16.293583	-3.690836
H	9.862706	17.335709	-3.967746
H	10.809131	15.962051	-4.428772
C	7.645364	16.315159	-4.088665
H	6.947222	16.307705	-3.247043
H	7.903396	17.365250	-4.274647
H	7.096305	15.972022	-4.974423
C	8.979701	14.379690	-4.776489
H	8.187667	14.383228	-5.535745
H	9.929260	14.426603	-5.324051
H	8.951663	13.406319	-4.278363
N	-4.476720	1.041472	5.926023
N	-6.314957	3.150846	6.747019
C	-4.384256	0.454111	4.562358
H	-3.448236	-0.093378	4.395613
H	-5.194943	-0.251095	4.340162
H	-4.444062	1.242765	3.808373
C	-3.293067	0.669628	6.745689
H	-2.563992	0.063162	6.193846
H	-2.778406	1.571987	7.088374
H	-3.553342	0.095712	7.643862
C	-5.743050	0.623354	6.596303
H	-5.532557	0.055913	7.511986
H	-6.304819	-0.080733	5.969230

C	-6.737978	1.741351	6.993067
H	-7.683382	1.517827	6.482122
H	-6.964995	1.585031	8.055517
C	-7.298890	3.856442	5.883060
H	-7.670588	4.786440	6.330926
H	-6.839568	4.115760	4.925081
H	-8.185319	3.251128	5.655378
C	-6.116631	3.877477	8.028777
H	-6.699905	4.804829	8.089606
H	-6.391451	3.282551	8.908724
H	-5.062393	4.142024	8.147172
N	9.457679	1.723380	6.097900
N	10.617388	4.290889	6.855352
N	10.207063	15.551332	-1.205856
N	8.826812	15.468319	-3.774565
N	-6.733700	14.798114	-1.773092
N	-5.011850	14.745627	-4.130951
N	5.682293	12.100008	4.157070
N	5.807178	10.170464	5.594832
N	7.087381	10.241808	3.557196
N	8.217779	5.727988	5.490950
N	2.508450	13.211876	7.921505
N	2.326422	3.078261	-3.653390
N	3.465425	3.171163	1.257044
N	1.061412	3.092415	1.238703
N	2.251286	3.114580	3.333944
N	-2.612680	3.203460	4.687884
N	7.096693	3.224342	4.759848
N	-4.625540	13.110452	-0.248912
N	-3.798681	9.888796	3.561530
N	-2.868562	10.052513	5.775717
N	-2.801540	11.952148	4.302404
N	-0.273058	13.515589	8.387237
N	2.271591	5.389838	-5.296969
N	0.856090	9.896426	-3.523026
N	1.957194	11.900227	-2.774600
N	3.258173	10.020547	-3.529634
N	6.716303	13.564601	-2.527211
N	-2.952634	13.045475	-2.528581
Pd	1.410341	14.248082	9.397022
Pd	-4.825977	13.923150	-2.171054
Pd	8.845764	3.746507	5.801692
Pd	8.458925	14.544046	-1.889125
Pd	2.353558	3.339830	-5.742803
Pd	-4.449036	3.166760	5.712480
C	2.650169	5.661454	6.083317
C	2.980081	6.372471	4.787259
C	1.801031	6.374864	3.844953
C	0.596193	6.737966	4.446073
C	0.723980	7.060141	5.911145
C	1.504501	6.015849	6.655863
H	2.873462	6.110577	2.015409
H	3.388973	5.040654	6.588478
H	3.907733	6.059218	4.306987
C	1.903376	6.307631	2.460672
C	-0.514821	6.992753	3.649345
H	-0.205165	7.391509	6.384967
H	1.224426	5.717818	7.667226
C	-0.444501	6.905681	2.253354
C	0.784466	6.557814	1.651933
H	-1.439008	7.317217	4.115049
C	-1.678930	7.225928	1.437001
C	-2.528356	5.989083	1.112941
H	-1.396722	7.720469	0.504378
H	-2.290789	7.951783	1.984869
H	-3.393696	6.248000	0.498539
H	-1.946071	5.239774	0.569346
H	-2.889348	5.527152	2.037674
C	0.973490	6.547102	0.148601
C	1.304733	7.939736	-0.414036
H	0.084363	6.147572	-0.352743
H	1.799519	5.873932	-0.101221
H	1.515659	7.874208	-1.483825
H	0.475560	8.638633	-0.274989
H	2.184585	8.354486	0.086093
C	3.157546	8.897447	3.982705
C	3.122826	8.009930	5.224279
C	1.864633	8.453094	5.962588
C	1.292002	9.596432	5.152805
H	4.083623	8.157791	5.814854
H	1.970841	8.741707	7.035849
C	1.636698	10.530170	2.787332
C	0.183714	10.316422	2.336933
C	1.911148	12.008508	3.104113
H	2.306792	10.203898	1.988265
C	-0.159900	11.218761	1.144439
H	-0.484723	10.542909	3.176260
H	0.042307	9.267027	2.084799

C	1.543719	12.906777	1.908780
H	1.320417	12.289974	3.979250
H	2.969667	12.132865	3.352459
C	0.088005	12.694053	1.472784
H	-1.202919	11.048421	0.846589
H	0.453890	10.934241	0.276296
H	1.718043	13.955489	2.163413
H	2.205509	12.673762	1.063243
H	-0.144568	13.333139	0.614147
H	-0.579523	13.001562	2.289003
N	1.997605	9.670156	3.937116
O	0.342190	10.301411	5.434603
O	4.007957	8.907146	3.104552
O	5.319673	6.450433	2.644791
H	5.586589	6.414675	1.717817
H	4.994271	7.361108	2.758444

Table5_1c_reactant

Property	Value
Charge	0
Electronic Energy, BS1 (a.u.)	-1290.455014
Thermal and entropic correction, BS1 (a.u.)	2.850834
Electronic Energy, BS2 (a.u.)	-1290.908198
Number of Imaginary Frequencies	0
Imaginary frequencies (cm-1)	None

Molecular Geometry in Cartesian Coordinates

C	8.297136	1.028886	6.862178
H	8.651496	0.544286	7.780601
H	7.556047	1.773111	7.166373
H	7.771002	0.254749	6.289580
C	9.505880	1.085096	4.731353
H	8.764764	0.298012	4.543735
H	9.364793	1.856193	3.968777
H	10.488249	0.633665	4.543921
C	10.682190	1.605946	6.821783
H	11.432896	1.062169	6.234199
H	10.578198	1.010469	7.737905
C	11.340018	2.942709	7.242512
H	12.354541	2.933453	6.823852
H	11.498383	2.881683	8.326834
C	11.457111	5.041655	5.976697
H	10.981954	5.117353	4.994602
H	11.600510	6.062149	6.353359
H	12.461303	4.632599	5.809337
C	10.233227	4.960003	8.097153
H	9.143447	4.994721	8.179097
H	10.605013	4.508778	9.025558
H	10.596262	5.995370	8.091753
N	3.136140	14.989118	10.379485
N	0.268250	15.270290	10.839755
C	3.903095	13.820614	10.885933
H	3.340791	12.900485	10.705824
H	4.883063	13.711070	10.404971
H	4.093815	13.864494	11.965562
C	3.929125	15.754702	9.380961
H	3.380800	15.822569	8.437824
H	4.139561	16.784777	9.695358
H	4.902135	15.295639	9.165928
C	2.706654	15.871306	11.503958
H	3.143806	15.534602	12.452749
H	3.096485	16.889325	11.376396
C	1.185069	16.012185	11.753990
H	0.966614	17.087677	11.739811
H	1.015103	15.719031	12.797940
C	-0.629095	16.207272	10.112154
H	-1.692007	15.965293	10.237400
H	-0.512397	17.250981	10.429807
H	-0.411692	16.178675	9.040712
C	-0.519450	14.251901	11.583883
H	-0.247028	13.248552	11.243856
H	-0.344585	14.277346	12.666732
H	-1.602150	14.362720	11.444100
N	2.395953	1.270027	-6.135995
N	2.410855	3.639438	-7.838885
C	3.607909	0.718030	-5.474538
H	3.371180	0.016901	-4.664445
H	4.268843	0.178829	-6.164633
H	4.200216	1.530314	-5.044161
C	1.158897	0.685151	-5.554002
H	1.361922	-0.017484	-4.736148
H	0.522721	1.480020	-5.154385

H	0.554420	0.136800	-6.287290
C	2.447492	1.051856	-7.611323
H	1.599747	0.440459	-7.946197
H	3.329604	0.460580	-7.888660
C	2.463856	2.309708	-8.514743
H	3.357511	2.226705	-9.146475
H	1.628554	2.195377	-9.217479
C	1.179431	4.380863	-8.220518
H	1.388622	5.384281	-8.612047
H	0.587934	3.868360	-8.989461
H	0.528132	4.497421	-7.349959
C	3.627812	4.438797	-8.141339
H	3.397500	5.424839	-8.563960
H	4.205206	4.599953	-7.226366
H	4.300171	3.948150	-8.856185
C	-6.570324	15.722552	-0.616502
H	-7.099982	15.417369	0.294659
H	-5.508944	15.805156	-0.367873
H	-6.918779	16.736750	-0.848927
C	-7.654470	13.650907	-1.348109
H	-7.993961	13.721910	-0.307093
H	-8.561739	13.591289	-1.962128
H	-7.135093	12.694953	-1.459544
C	-7.268702	15.465708	-2.955601
H	-7.498171	16.515899	-2.733867
H	-8.230913	15.043357	-3.272448
C	-6.358955	15.474094	-4.209225
H	-6.964592	15.073471	-5.032212
H	-6.209047	16.527922	-4.476826
C	-4.988541	13.638655	-5.090537
H	-4.107715	13.689371	-5.742790
H	-4.951115	12.682752	-4.561283
H	-5.860238	13.595104	-5.755549
C	-3.915714	15.697714	-4.309350
H	-3.232795	15.381990	-5.107996
H	-4.235766	16.715700	-4.564427
H	-3.330678	15.773222	-3.388354
N	8.144864	13.662784	0.007703
C	7.248931	14.207131	0.857161
H	6.955410	15.229291	0.664284
C	6.706782	13.520132	1.961097
H	6.012067	14.044923	2.603327
C	7.107442	12.187724	2.229967
C	8.046992	11.627729	1.328828
H	8.422850	10.620121	1.451361
C	8.525743	12.391034	0.247080
H	9.244043	11.959182	-0.434957
C	6.617658	11.462022	3.356437
C	5.370831	11.385202	5.250172
C	4.405659	11.975248	6.123646
C	3.823359	13.240449	5.856703
H	4.085760	13.811873	4.975982
C	2.897071	13.796278	6.761522
H	2.461842	14.765394	6.562608
C	3.041420	11.969116	8.158993
H	2.716379	11.480944	9.066805
C	3.978462	11.337199	7.316201
H	4.366895	10.371360	7.611959
C	6.720940	9.640789	4.703006
C	7.235202	8.338157	4.982083
C	6.920849	7.642965	6.177360
H	6.293532	8.084000	6.940211
C	8.096636	7.663907	4.079760
H	8.399507	8.110756	3.141896
C	8.581979	6.379810	4.394534
H	9.237601	5.868736	3.704117
C	7.455729	6.359620	6.407418
H	7.221716	5.833651	7.321584
C	3.455796	3.151968	-2.914020
H	4.384350	3.161494	-3.467112
C	3.479370	3.164532	-1.506071
H	4.441815	3.174323	-1.010776
C	1.149640	3.108946	-2.954851
H	0.242783	3.080807	-3.541955
C	1.075153	3.122293	-1.547139
H	0.098332	3.097277	-1.081786
C	2.264438	3.148531	-0.775047
C	2.243715	3.157194	0.652952
C	1.089132	3.166939	2.607528
C	3.358585	3.194534	2.630043
C	4.591166	3.217245	3.349529
C	4.637744	3.175876	4.765482
H	3.734917	3.100753	5.357310
C	5.843490	3.281508	2.688223
H	5.915952	3.305589	1.608972
C	7.034642	3.313464	3.438040
H	7.987223	3.364538	2.930765
C	5.879671	3.213097	5.430275

H	5.912771	3.178706	6.509604
C	-0.156922	3.188164	3.304517
C	-1.394945	3.101722	2.619851
H	-1.443123	3.010645	1.542615
C	-2.602483	3.107517	3.343175
H	-3.544806	3.038109	2.818422
C	-1.489469	3.288834	5.357072
H	-1.548295	3.361599	6.433576
C	-0.232594	3.288020	4.717520
H	0.659229	3.358329	5.327084
C	-3.943108	13.742984	0.708361
H	-3.716288	14.782471	0.520001
C	-4.906111	11.782359	-0.027872
H	-5.449251	11.261261	-0.803934
C	-4.549375	11.103908	1.155188
H	-4.838545	10.066453	1.263073
C	-3.853705	11.790311	2.181693
C	-3.549201	13.151006	1.923271
H	-3.032606	13.766787	2.648137
C	-3.495294	11.158649	3.410673
C	-3.509869	9.347718	4.776258
C	-2.554801	11.286751	5.472745
N	-4.461982	5.228473	5.513500
C	-3.835777	7.978405	5.020302
C	-4.486004	7.170683	4.053106
H	-4.784877	7.568423	3.091678
C	-4.773716	5.820501	4.341180
H	-5.279490	5.209155	3.606663
C	-3.516135	7.341113	6.246037
H	-3.036360	7.874659	7.056576
C	-3.841482	5.985170	6.441217
H	-3.598729	5.507329	7.379936
C	-1.831291	12.028401	6.454039
C	-1.441717	11.456857	7.690182
H	-1.713222	10.443760	7.953934
C	-0.669904	12.206607	8.598476
H	-0.370838	11.763188	9.536954
C	-0.658655	14.036854	7.207377
H	-0.354027	15.059325	7.036059
C	-1.424601	13.367286	6.234072
H	-1.688463	13.898189	5.329269
C	3.396937	6.100635	-4.982905
H	4.339638	5.601279	-5.156938
C	1.094944	6.032261	-5.086427
H	0.202951	5.479049	-5.343920
C	0.983033	7.335794	-4.565105
H	-0.006308	7.757101	-4.443787
C	3.383781	7.408712	-4.458562
H	4.330804	7.887244	-4.242780
C	2.149968	8.069481	-4.233079
C	2.083738	9.393077	-3.700419
C	0.853519	11.169563	-3.005052
C	3.119385	11.258117	-2.925291
C	-0.418436	11.793746	-2.829123
C	-1.628822	11.124453	-3.141294
H	-1.637408	10.110491	-3.519726
C	-2.860896	11.787889	-2.987142
H	-3.781331	11.277375	-3.232762
C	-1.824424	13.708828	-2.237379
H	-1.921278	14.727673	-1.889506
C	-0.547157	13.124718	-2.357222
H	0.319660	13.721802	-2.104631
C	4.319507	12.002353	-2.719572
C	4.301382	13.356041	-2.299506
H	3.373514	13.875761	-2.101325
C	5.510139	14.067120	-2.169413
H	5.493293	15.104471	-1.867539
C	5.599426	11.441249	-2.958522
H	5.718938	10.414831	-3.280360
C	6.754722	12.229315	-2.795195
H	7.728106	11.799869	-2.984924
C	11.218932	14.510105	-0.824904
H	10.805114	13.511677	-0.992061
H	12.175049	14.549266	-1.361715
H	11.462639	14.590746	0.241872
C	9.881037	16.451016	-0.159053
H	8.800100	16.420954	0.005222
H	10.364286	16.180370	0.788073
H	10.143394	17.499246	-0.349140
C	10.792335	16.313963	-2.434585
H	10.862944	17.382605	-2.194551
H	11.827682	16.019124	-2.648492
C	10.025188	16.215825	-3.776867
H	9.801614	17.246276	-4.081473
H	10.750137	15.867953	-4.523856
C	7.595467	16.196777	-4.123366
H	6.914858	16.217867	-3.267685
H	7.838383	17.240817	-4.357626

H	7.031982	15.812354	-4.982829
C	8.935658	14.247096	-4.759004
H	8.126461	14.209462	-5.498994
H	9.872243	14.281436	-5.329349
H	8.930592	13.295632	-4.219516
N	-4.508305	1.008551	5.903082
N	-6.371536	3.097425	6.721005
C	-4.402070	0.428319	4.537267
H	-3.460030	-0.109804	4.373790
H	-5.204914	-0.283266	4.306985
H	-4.464539	1.219575	3.786188
C	-3.326310	0.643292	6.728317
H	-2.589207	0.044571	6.178604
H	-2.820705	1.548288	7.077610
H	-3.587207	0.063859	7.622767
C	-5.774864	0.575917	6.563686
H	-5.564903	0.006495	7.478269
H	-6.326128	-0.130632	5.930098
C	-6.782661	1.682995	6.958773
H	-7.722612	1.452759	6.440838
H	-7.015059	1.520261	8.019100
C	-7.355749	3.796899	5.852413
H	-7.738366	4.722512	6.300186
H	-6.891900	4.062716	4.898296
H	-8.235436	3.184656	5.617230
C	-6.189580	3.821254	8.006792
H	-6.783749	4.741700	8.067597
H	-6.463196	3.219857	8.882680
H	-5.139239	4.097241	8.132900
N	9.385386	1.683116	6.087959
N	10.625785	4.201852	6.879739
N	10.249262	15.542981	-1.277731
N	8.792357	15.374973	-3.800301
N	-6.754083	14.767470	-1.741349
N	-5.052265	14.758781	-4.114053
N	5.683691	12.068434	4.125600
N	5.859752	10.174045	5.601167
N	7.110772	10.219660	3.547080
N	8.276336	5.734565	5.538519
N	2.513358	13.183007	7.899592
N	2.315839	3.118736	-3.633283
N	3.437364	3.227316	1.281070
N	1.033926	3.152736	1.255241
N	2.216595	3.188190	3.354852
N	-2.656607	3.193754	4.686881
N	7.063255	3.277333	4.785600
N	-4.617033	13.081025	-0.253744
N	-3.814399	9.851664	3.557871
N	-2.860472	10.001757	5.763683
N	-2.827864	11.915756	4.307900
N	-0.271386	13.475893	8.372176
N	2.276629	5.417928	-5.298537
N	0.851998	9.907951	-3.489173
N	1.948127	11.886031	-2.665417
N	3.252450	9.999418	-3.402065
N	6.719475	13.523517	-2.417248
N	-2.964420	13.059557	-2.552253
Pd	1.412097	14.226664	9.370107
Pd	-4.842193	13.914487	-2.166198
Pd	8.833333	3.728757	5.827560
Pd	8.475679	14.527634	-1.871366
Pd	2.353177	3.363711	-5.725530
Pd	-4.498727	3.135350	5.699943
C	2.760644	5.810861	5.978453
C	2.961521	6.088944	4.641527
C	1.860273	6.442733	3.808708
C	0.639819	6.826911	4.435325
C	0.608423	6.886660	5.855469
C	1.571552	6.225316	6.605533
H	2.879466	6.168651	1.935873
H	3.566094	5.413573	6.595105
H	3.937662	5.954827	4.170949
C	1.918443	6.395133	2.395840
C	-0.477136	7.106376	3.616719
H	-0.238274	7.365349	6.342191
H	1.471232	6.140706	7.689443
C	-0.431419	6.986282	2.240515
C	0.806472	6.629297	1.613650
H	-1.401868	7.411658	4.094628
C	-1.679703	7.278785	1.429367
C	-2.517441	6.029351	1.123271
H	-1.415009	7.771848	0.490580
H	-2.293801	7.999306	1.981327
H	-3.388176	6.274981	0.518057
H	-1.932917	5.284413	0.576193
H	-2.860308	5.568353	2.055368
C	0.957225	6.602206	0.110032
C	1.302341	7.990616	-0.452664

H	0.056911	6.217783	-0.372514
H	1.769269	5.916384	-0.150555
H	1.476350	7.919261	-1.526285
H	0.489105	8.703301	-0.291601
H	2.203015	8.393899	0.019738
C	3.175627	9.068528	3.840094
C	3.250411	8.498745	5.202042
C	2.188314	8.873247	5.887981
C	1.362680	9.750275	5.031705
H	4.204404	8.039480	5.559723
H	1.985513	8.814382	6.970071
C	1.591830	10.616109	2.640284
C	0.117228	10.420461	2.259915
C	1.913972	12.094936	2.915721
H	2.210805	10.291108	1.798558
C	-0.245795	11.294884	1.056473
H	-0.503813	10.689121	3.115025
H	-0.056334	9.367276	2.045302
C	1.535112	12.962673	1.710321
H	1.354248	12.414789	3.807166
H	2.980615	12.193766	3.155497
C	0.059001	12.772233	1.337156
H	-1.301718	11.151718	0.794293
H	0.342202	10.970018	0.183764
H	1.741613	14.014433	1.921728
H	2.159535	12.672151	0.854747
H	-0.191450	13.394176	0.471762
H	-0.568461	13.120256	2.168593
N	1.989679	9.770381	3.767508
O	0.399847	10.428818	5.339287
O	4.026511	9.026004	2.941307
O	5.357659	6.539645	2.602252
H	5.638662	6.436442	1.684863
H	5.023501	7.455172	2.660300
O	4.252266	10.967387	0.698443
H	4.278734	10.231469	1.332989
H	4.192520	11.746723	1.264647

Table5_1c_TSi_TSi-ii_h2o_2

Property	Value
Charge	0
Electronic Energy, BS1 (a.u.)	-1290.415402
Thermal and entropic correction, BS1 (a.u.)	2.856011
Electronic Energy, BS2 (a.u.)	-1290.864747
Number of Imaginary Frequencies	0
Imaginary frequencies (cm-1)	None

Molecular Geometry in Cartesian Coordinates

C	8.291177	1.029373	6.857415
H	8.642826	0.542260	7.775553
H	7.551118	1.774586	7.161628
H	7.764408	0.257473	6.282384
C	9.503894	1.087081	4.728869
H	8.761711	0.301665	4.538518
H	9.365400	1.859972	3.967641
H	10.485787	0.634324	4.542085
C	10.677510	1.601372	6.822431
H	11.427327	1.055041	6.236077
H	10.570115	1.006343	7.738449
C	11.339140	2.936035	7.243848
H	12.353835	2.923898	6.825683
H	11.496796	2.874214	8.328226
C	11.462683	5.034658	5.977926
H	10.987776	5.111516	4.995775
H	11.608932	6.054823	6.354386
H	12.465726	4.622711	5.810806
C	10.238802	4.956794	8.098492
H	9.149153	4.994738	8.180728
H	10.609488	4.504564	9.026849
H	10.604876	5.991086	8.092958
N	3.137979	14.988326	10.376131
N	0.270591	15.270088	10.839035
C	3.905453	13.819767	10.881662
H	3.342809	12.899686	10.702354
H	4.884773	13.710077	10.399424
H	4.097593	13.863709	11.961037
C	3.929883	15.753946	9.376789
H	3.380572	15.821686	8.434216
H	4.140533	16.784056	9.690918
H	4.902713	15.294960	9.160787
C	2.709734	15.870423	11.501136
H	3.147716	15.533494	12.449463

H	3.099664	16.888377	11.373360
C	1.188412	16.011536	11.752628
H	0.970169	17.087076	11.738917
H	1.019349	15.718126	12.796651
C	-0.626884	16.207475	10.112121
H	-1.689815	15.966020	10.238226
H	-0.509418	17.251146	10.429619
H	-0.410339	16.178670	9.040510
C	-0.517011	14.251997	11.583674
H	-0.245333	13.248556	11.243306
H	-0.341326	14.277272	12.666397
H	-1.599762	14.363325	11.444715
N	2.396714	1.270004	-6.132596
N	2.411719	3.637326	-7.838345
C	3.608360	0.718567	-5.470119
H	3.371253	0.018482	-4.659235
H	4.269384	0.178390	-6.159366
H	4.200711	1.531262	-5.040589
C	1.159365	0.686104	-5.550244
H	1.362003	-0.015521	-4.731430
H	0.523216	1.481613	-5.151843
H	0.555020	0.136911	-6.283010
C	2.448625	1.050034	-7.607644
H	1.600748	0.438567	-7.942052
H	3.330606	0.458091	-7.883981
C	2.465795	2.306804	-8.512560
H	3.360124	2.223230	-9.143259
H	1.631259	2.191464	-9.216041
C	1.179755	4.377351	-8.220953
H	1.388208	5.380428	-8.613756
H	0.588633	3.863422	-8.989236
H	0.528359	4.494509	-7.350547
C	3.628094	4.437232	-8.141727
H	3.397057	5.422582	-8.565576
H	4.205329	4.599986	-7.226937
H	4.300853	3.946207	-8.855939
C	-6.572428	15.724210	-0.618841
H	-7.103885	15.419968	0.291585
H	-5.511424	15.806287	-0.368427
H	-6.919811	16.738488	-0.852501
C	-7.656627	13.652784	-1.350984
H	-7.997731	13.724587	-0.310544
H	-8.562970	13.593461	-1.966400
H	-7.137702	12.696410	-1.461027
C	-7.267021	15.466358	-2.958960
H	-7.496608	16.516713	-2.738124
H	-8.228749	15.044125	-3.277425
C	-6.354863	15.473973	-4.210839
H	-6.959195	15.073630	-5.034915
H	-6.203758	16.527654	-4.478350
C	-4.984197	13.637328	-5.089093
H	-4.102018	13.687058	-5.739592
H	-4.948728	12.681569	-4.559477
H	-5.854637	13.594332	-5.755793
C	-3.911319	15.695896	-4.306617
H	-3.227251	15.379535	-5.104023
H	-4.230207	16.714065	-4.562434
H	-3.327901	15.771207	-3.384578
N	8.142770	13.661093	0.005175
C	7.245974	14.204882	0.854072
H	6.952703	15.227200	0.661636
C	6.702695	13.517174	1.957002
H	6.007448	14.041638	2.598932
C	7.103182	12.184648	2.225541
C	8.043374	11.625089	1.324797
H	8.419136	10.617415	1.447133
C	8.523253	12.389106	0.244021
H	9.242268	11.957715	-0.437567
C	6.613191	11.458772	3.351811
C	5.367100	11.382499	5.246083
C	4.402805	11.973128	6.120079
C	3.820775	13.238489	5.853326
H	4.082621	13.809593	4.972241
C	2.895449	13.794819	6.758828
H	2.460329	14.764017	6.560137
C	3.040083	11.967765	8.156392
H	2.715282	11.479662	9.064313
C	3.976325	11.335469	7.313060
H	4.364180	10.369293	7.608380
C	6.718708	9.638846	4.699936
C	7.236090	8.337934	4.981267
C	6.923539	7.644130	6.177822
H	6.295417	8.085075	6.940087
C	8.099280	7.664235	4.080234
H	8.401224	8.110252	3.141679
C	8.587731	6.381871	4.397299
H	9.244899	5.871367	3.707910
C	7.461460	6.362446	6.410040

H	7.228862	5.837654	7.325244
C	3.456615	3.155082	-2.912713
H	4.385216	3.163825	-3.465741
C	3.480093	3.168685	-1.504760
H	4.442497	3.178373	-1.009390
C	1.150442	3.112900	-2.953675
H	0.243612	3.084643	-3.540820
C	1.075870	3.127413	-1.545983
H	0.099000	3.103066	-1.080724
C	2.265101	3.153818	-0.773804
C	2.244229	3.163492	0.654198
C	1.089356	3.173182	2.608590
C	3.358800	3.201732	2.631510
C	4.591305	3.224591	3.351151
C	4.637671	3.186298	4.767220
H	3.734684	3.114058	5.359158
C	5.843798	3.285832	2.689826
H	5.916413	3.307377	1.610533
C	7.034941	3.317792	3.439659
H	7.987630	3.366583	2.932391
C	5.879594	3.223216	5.432060
H	5.912671	3.191212	6.511475
C	-0.156830	3.192417	3.305393
C	-1.394665	3.105513	2.620438
H	-1.442587	3.015554	1.543096
C	-2.602318	3.109215	3.343576
H	-3.544509	3.039322	2.818639
C	-1.489762	3.289148	5.357872
H	-1.548796	3.360472	6.434459
C	-0.232806	3.290369	4.718507
H	0.658856	3.360593	5.328312
C	-3.947629	13.743556	0.711091
H	-3.721128	14.783172	0.523055
C	-4.910179	11.782933	-0.025531
H	-5.453416	11.261921	-0.801582
C	-4.552928	11.104170	1.157216
H	-4.841623	10.066530	1.264741
C	-3.857060	11.790414	2.183691
C	-3.553259	13.151344	1.925711
H	-3.036576	13.767069	2.650562
C	-3.497171	11.158232	3.411965
C	-3.509229	9.346533	4.776485
C	-2.554136	11.285540	5.472968
N	-4.462613	5.227638	5.514010
C	-3.835567	7.977354	5.020669
C	-4.486342	7.169880	4.053629
H	-4.785354	7.567750	3.092298
C	-4.774441	5.819806	4.341777
H	-5.280764	5.208679	3.607457
C	-3.515711	7.339914	6.246284
H	-3.035538	7.873325	7.056697
C	-3.841524	5.984087	6.441545
H	-3.603447	5.506086	7.380183
C	-1.831011	12.027424	6.454292
C	-1.440804	11.455839	7.690206
H	-1.711448	10.442473	7.953753
C	-0.669217	12.205860	8.598446
H	-0.369451	11.762320	9.536646
C	-0.659638	14.036479	7.207862
H	-0.355644	15.059149	7.036619
C	-1.425455	13.366702	6.234634
H	-1.689816	13.897731	5.330062
C	3.397686	6.101603	-4.984029
H	4.340480	5.602246	-5.157556
C	1.095792	6.033323	-5.088886
H	0.203916	5.480205	-5.346948
C	0.983587	7.336836	-4.567570
H	-0.005793	7.758256	-4.446977
C	3.384276	7.409682	-4.459709
H	4.331204	7.888159	-4.243382
C	2.150332	8.070452	-4.234838
C	2.083844	9.393997	-3.702121
C	0.853518	11.169708	-3.004873
C	3.119388	11.258662	-2.926117
C	-0.418533	11.793253	-2.827220
C	-1.628888	11.124079	-3.139790
H	-1.637316	10.110794	-3.520016
C	-2.861116	11.786798	-2.983735
H	-3.781520	11.276306	-3.229486
C	-1.824834	13.706919	-2.231490
H	-1.921901	14.725120	-1.881798
C	-0.547432	13.123403	-2.353001
H	0.319336	13.720289	-2.099783
C	4.319509	12.003116	-2.721311
C	4.301513	13.356783	-2.301235
H	3.373699	13.876325	-2.102319
C	5.510284	14.068034	-2.172165
H	5.493496	15.105377	-1.870269

C	5.599299	11.442232	-2.961397
H	5.718721	10.415844	-3.283360
C	6.754580	12.230480	-2.799088
H	7.727844	11.801185	-2.989759
C	11.217771	14.508998	-0.823398
H	10.804062	13.510679	-0.991457
H	12.174477	14.548284	-1.359152
H	11.460350	14.589142	0.243671
C	9.879325	16.449705	-0.158045
H	8.798211	16.419600	0.005053
H	10.361539	16.178555	0.789462
H	10.141939	17.498008	-0.347362
C	10.793138	16.313712	-2.432629
H	10.863929	17.382164	-2.191804
H	11.828581	16.018658	-2.645768
C	10.027183	16.216766	-3.775682
H	9.804422	17.247516	-4.079873
H	10.752615	15.868948	-4.522226
C	7.597627	16.199384	-4.123610
H	6.916540	16.219880	-3.268290
H	7.841229	17.243541	-4.356632
H	7.034433	15.816155	-4.983798
C	8.937168	14.249695	-4.760559
H	8.128481	14.213392	-5.501170
H	9.874168	14.284074	-5.330222
H	8.931124	13.297644	-4.222134
N	-4.508022	1.007698	5.902508
N	-6.372022	3.095875	6.720505
C	-4.401131	0.427892	4.536557
H	-3.458965	-0.110089	4.373328
H	-5.203800	-0.283707	4.305704
H	-4.463332	1.219363	3.785677
C	-3.326228	0.642501	6.728061
H	-2.588772	0.044131	6.178438
H	-2.820979	1.547514	7.077820
H	-3.587304	0.062730	7.622241
C	-5.774699	0.574550	6.562557
H	-5.564883	0.004755	7.476941
H	-6.325668	-0.131815	5.928509
C	-6.782787	1.681277	6.957903
H	-7.722640	1.450927	6.439841
H	-7.015223	1.518222	8.018173
C	-7.356230	3.795223	5.851804
H	-7.739105	4.720728	6.299583
H	-6.892262	4.061203	4.897790
H	-8.235759	3.182832	5.616416
C	-6.190569	3.819538	8.006460
H	-6.785279	4.739626	8.067427
H	-6.463912	3.217800	8.882199
H	-5.140399	4.096120	8.132700
N	9.382224	1.682732	6.086401
N	10.628890	4.197411	6.881043
N	10.248698	15.542211	-1.276767
N	8.793904	15.376624	-3.800727
N	-6.754886	14.768545	-1.743409
N	-5.048845	14.757794	-4.113075
N	5.679386	12.065242	4.121081
N	5.856515	10.171623	5.597381
N	7.106868	10.216686	3.542925
N	8.283551	5.737683	5.542296
N	2.512359	13.181829	7.897253
N	2.316684	3.121743	-3.632030
N	3.437798	3.233506	1.282495
N	1.034348	3.159622	1.256296
N	2.216663	3.195374	3.356094
N	-2.656693	3.193702	4.687382
N	7.063266	3.284409	4.787275
N	-4.621510	13.081755	-0.251159
N	-3.815179	9.850922	3.558634
N	-2.858350	10.000068	5.763287
N	-2.829196	11.915159	4.308939
N	-0.271541	13.475453	8.372357
N	2.277559	5.418886	-5.300287
N	0.852059	9.908654	-3.490536
N	1.948135	11.886218	-2.665321
N	3.252498	10.000222	-3.403490
N	6.719525	13.524644	-2.421021
N	-2.964767	13.057726	-2.546736
Pd	1.412866	14.226034	9.368534
Pd	-4.842822	13.914073	-2.164491
Pd	8.834973	3.730079	5.828985
Pd	8.475685	14.527344	-1.872888
Pd	2.354196	3.364173	-5.724637
Pd	-4.498964	3.134545	5.699929
C	2.752180	5.824376	6.005089
C	2.997175	6.293660	4.674896
C	1.849010	6.455161	3.803319
C	0.637357	6.827231	4.423502

C	0.686230	7.017319	5.857236
C	1.584290	6.201568	6.612411
H	2.884271	6.183087	1.946429
H	3.540985	5.338675	6.569003
H	3.945339	6.071341	4.194627
C	1.921012	6.394615	2.404209
C	-0.482770	7.088074	3.619658
H	-0.193589	7.429328	6.343991
H	1.413324	6.029520	7.669174
C	-0.431148	6.981225	2.234484
C	0.801263	6.630531	1.614880
H	-1.406626	7.398432	4.096047
C	-1.677158	7.278902	1.424663
C	-2.512899	6.029513	1.113105
H	-1.409628	7.774499	0.488041
H	-2.293666	7.997626	1.976300
H	-3.385621	6.275630	0.504117
H	-1.925552	5.285343	0.567993
H	-2.860222	5.567308	2.042976
C	0.965464	6.611223	0.111749
C	1.312111	8.000615	-0.447512
H	0.066236	6.227811	-0.378342
H	1.777942	5.925618	-0.148025
H	1.491649	7.931446	-1.521126
H	0.499185	8.713394	-0.285309
H	2.212810	8.402331	0.026066
C	3.171433	9.040356	3.828544
C	3.217627	8.323758	5.142077
C	2.081859	8.749347	5.872160
C	1.363066	9.742042	5.043525
H	4.184873	8.157201	5.589324
H	2.038546	8.846947	6.946090
C	1.592243	10.615292	2.646149
C	0.115251	10.421803	2.274183
C	1.913310	12.094002	2.918166
H	2.205929	10.283859	1.805983
C	-0.252863	11.295201	1.066799
H	-0.504901	10.692763	3.132675
H	-0.061431	9.368633	2.062134
C	1.529192	12.961004	1.709092
H	1.358883	12.415595	3.807484
H	2.981346	12.196417	3.147393
C	0.050768	12.772417	1.344125
H	-1.310155	11.148347	0.814612
H	0.325496	10.968594	0.191499
H	1.738428	14.012693	1.918180
H	2.148431	12.668691	0.850312
H	-0.203507	13.393719	0.479355
H	-0.571675	13.122279	2.178532
N	1.999271	9.769657	3.781048
O	0.419537	10.445670	5.349501
O	4.019254	9.021113	2.931903
O	5.362619	6.538760	2.607704
H	5.643880	6.429185	1.691127
H	5.030114	7.453570	2.653794
O	4.238975	10.956957	0.686109
H	4.272149	10.222050	1.321678
H	4.184166	11.737180	1.251096

Table5_1d_TSi-ii_0wat

Property	Value
Charge	0
Electronic Energy, BS1 (a.u.)	-1208.106659
Thermal and entropic correction, BS1 (a.u.)	2.641923
Electronic Energy, BS2 (a.u.)	-1208.526435
Number of Imaginary Frequencies	0
Imaginary frequencies (cm-1)	None

Molecular Geometry in Cartesian Coordinates

C	-9.074897	8.980943	4.670142
H	-9.884651	8.863977	5.401133
H	-8.185556	8.522204	5.110946
H	-8.885686	10.058884	4.592057
C	-9.405224	9.366210	2.273326
H	-9.169073	10.377239	2.628219
H	-8.674479	9.109762	1.501552
H	-10.379007	9.435799	1.772552
C	-10.660719	7.592772	3.413073
H	-11.386556	8.001498	2.698414
H	-11.147441	7.717551	4.388838
C	-10.598153	6.069020	3.147658
H	-11.293259	5.869975	2.321931

H	-11.067493	5.585140	4.013805
C	-9.216484	4.868119	1.512330
H	-8.503073	5.407055	0.882343
H	-8.913496	3.813481	1.524479
H	-10.183015	4.903005	0.994423
C	-8.893732	4.475791	3.908750
H	-8.024656	4.824756	4.472870
H	-9.693609	4.300007	4.639034
H	-8.640716	3.494496	3.488147
N	2.675241	-1.024338	7.435602
N	4.609163	0.742347	8.723881
C	1.223664	-1.055555	7.757800
H	0.836735	-0.036332	7.835684
H	0.628082	-1.576222	6.997489
H	1.002977	-1.550011	8.712239
C	2.951296	-1.744296	6.163667
H	3.426184	-1.068250	5.446631
H	3.629141	-2.597822	6.288942
H	2.043758	-2.139934	5.690600
C	3.482134	-1.591007	8.556010
H	2.831351	-1.969464	9.354762
H	4.040967	-2.475950	8.225476
C	4.511051	-0.655409	9.236918
H	5.481320	-1.164495	9.173320
H	4.274783	-0.663586	10.308700
C	5.962913	1.011525	8.170589
H	6.440102	1.892691	8.617388
H	6.661562	0.177813	8.314230
H	5.898049	1.184004	7.092855
C	4.265706	1.723500	9.787190
H	3.342046	2.247712	9.528625
H	4.099237	1.257577	10.766469
H	5.044522	2.481753	9.936803
N	-1.727896	17.589388	-3.227284
N	0.154480	16.923671	-5.356234
C	-3.097263	17.017318	-3.133856
H	-3.546583	17.143637	-2.140942
H	-3.800655	17.462463	-3.848617
H	-3.069673	15.945715	-3.349587
C	-1.337380	18.247356	-1.952230
H	-2.110055	18.168988	-1.177230
H	-0.427089	17.786692	-1.557757
H	-1.125605	19.317913	-2.066157
C	-1.625755	18.540584	-4.372598
H	-1.337387	19.541501	-4.026717
H	-2.605887	18.692242	-4.842661
C	-0.648573	18.171567	-5.516328
H	-1.244801	18.138679	-6.437267
H	0.006703	19.041345	-5.653066
C	1.607405	17.232601	-5.283630
H	2.200391	16.676793	-6.020797
H	1.831813	18.294218	-5.446319
H	1.993704	16.979377	-4.292588
C	-0.129794	15.962467	-6.454716
H	0.774293	15.643768	-6.988763
H	-0.605059	15.064055	-6.050363
H	-0.809984	16.365366	-7.215483
C	12.154501	8.563853	2.727930
H	11.895217	8.582574	3.793847
H	11.427346	7.921588	2.223380
H	13.124473	8.055985	2.656868
C	11.855750	10.964661	3.118991
H	11.657415	10.553246	4.116728
H	12.669131	11.690820	3.242138
H	10.973034	11.533402	2.815247
C	13.454458	10.188316	1.427346
H	14.161442	9.364292	1.588030
H	13.953858	11.059119	1.871091
C	13.415847	10.438655	-0.100350
H	13.894924	11.411969	-0.266692
H	14.112241	9.718693	-0.548993
C	11.743122	11.687797	-1.392422
H	11.497192	11.604342	-2.458478
H	10.875960	12.125120	-0.889275
H	12.553554	12.423841	-1.321095
C	12.047087	9.288208	-1.782374
H	11.754360	9.638128	-2.780328
H	13.011745	8.780325	-1.905723
H	11.326634	8.523659	-1.478043
N	0.613540	3.043946	-3.907479
C	1.344282	2.560001	-2.882296
H	2.307524	2.134218	-3.125586
C	0.904636	2.584634	-1.543781
H	1.548339	2.163258	-0.782317
C	-0.361852	3.136651	-1.223314
C	-1.110188	3.650449	-2.312721
H	-2.090136	4.088403	-2.172174
C	-0.587955	3.584611	-3.619393

H	-1.160938	3.975758	-4.448342
C	-0.855409	3.171827	0.116243
C	-0.566581	2.755230	2.327544
C	0.242702	2.270815	3.398557
C	1.523819	1.706381	3.178123
H	1.935259	1.606981	2.181938
C	2.284471	1.241268	4.268817
H	3.256208	0.800826	4.095816
C	0.648731	1.847958	5.774116
H	0.319931	1.902884	6.801778
C	-0.187385	2.326154	4.747355
H	-1.154071	2.729030	5.017124
C	-2.463737	3.765360	1.602693
C	-3.704836	4.410612	1.888069
C	-4.206931	4.521923	3.208762
H	-3.676904	4.103082	4.053437
C	-4.506750	4.983323	0.869222
H	-4.217037	4.939359	-0.172881
C	-5.722027	5.613949	1.201251
H	-6.335277	6.042196	0.420946
C	-5.430038	5.178288	3.445798
H	-5.806833	5.261987	4.454966
C	-1.898889	14.085201	-1.698112
H	-2.437291	13.908582	-2.618344
C	-2.267768	13.395357	-0.527329
H	-3.098796	12.703315	-0.579039
C	-0.199169	15.199177	-0.601910
H	0.607618	15.916594	-0.651323
C	-0.490965	14.549069	0.614028
H	0.104068	14.792047	1.484817
C	-1.562312	13.621736	0.681877
C	-1.923509	12.966789	1.898221
C	-1.628830	12.655798	4.124579
C	-3.259268	11.524308	3.029269
C	-4.270010	10.515847	3.011023
C	-4.635915	9.793688	4.175014
H	-4.167480	9.989618	5.130327
C	-4.958411	10.169922	1.820433
H	-4.747633	10.661467	0.879207
C	-5.950377	9.171573	1.845006
H	-6.475669	8.912291	0.936675
C	-5.640220	8.807521	4.103918
H	-5.921335	8.258719	4.991174
C	-0.940363	12.978323	5.333334
C	0.133357	13.904539	5.360742
H	0.479120	14.400151	4.462792
C	0.756355	14.224842	6.582785
H	1.563419	14.943656	6.604143
C	-0.616439	12.785871	7.752576
H	-0.897985	12.354419	8.702464
C	-1.303133	12.410140	6.580718
H	-2.116916	11.701687	6.664995
C	8.721793	8.252212	2.356520
H	9.218530	7.473553	1.795145
C	8.447258	10.508112	2.758760
H	8.728173	11.523606	2.518423
C	7.476963	10.263022	3.751366
H	7.037783	11.108318	4.265105
C	7.109733	8.930740	4.069917
C	7.764090	7.912195	3.331316
H	7.554367	6.863864	3.500714
C	6.143556	8.629242	5.077704
C	4.668017	9.312633	6.658302
C	4.915962	7.104112	6.222236
N	2.745833	12.495133	8.795890
C	4.019089	10.370897	7.363547
C	4.323518	11.732930	7.116032
H	5.065976	12.024623	6.384688
C	3.671586	12.744282	7.847140
H	3.916289	13.780922	7.663563
C	3.039853	10.124846	8.358489
H	2.750671	9.118209	8.629936
C	2.438431	11.203692	9.036872
H	1.699044	11.012409	9.801543
C	4.514938	5.747750	6.417599
C	3.487503	5.385813	7.325882
H	2.965286	6.128971	7.914144
C	3.126520	4.031387	7.476052
H	2.333873	3.757233	8.157766
C	4.702064	3.357216	5.929116
H	5.169916	2.545421	5.390168
C	5.120153	4.681837	5.704416
H	5.917713	4.853753	4.992848
C	0.521337	13.182446	-4.270586
H	-0.458218	13.167831	-4.727469
C	2.175881	14.395557	-3.220537
H	2.516182	15.348253	-2.841579
C	3.007282	13.264081	-3.112335

H	3.980131	13.382677	-2.653175
C	1.280974	11.997943	-4.207273
H	0.859451	11.093321	-4.626826
C	2.569441	12.013486	-3.616274
C	3.378741	10.840656	-3.534512
C	5.284553	9.815443	-2.849612
C	3.656096	8.614380	-3.879167
C	6.529031	9.866979	-2.151483
C	7.034797	11.073517	-1.604743
H	6.506635	12.011770	-1.713854
C	8.259756	11.072477	-0.909416
H	8.643726	11.995054	-0.498242
C	8.538874	8.808781	-1.240788
H	9.144423	7.925919	-1.092755
C	7.325815	8.712080	-1.950186
H	7.030629	7.744247	-2.335076
C	3.161492	7.369295	-4.373316
C	3.911801	6.170218	-4.275321
H	4.896176	6.152296	-3.825282
C	3.386737	4.966905	-4.786507
H	3.963245	4.055581	-4.713830
C	1.892897	7.259307	-4.997327
H	1.247450	8.118707	-5.127209
C	1.450520	6.011904	-5.479551
H	0.484710	5.932629	-5.958189
C	-0.948402	1.204426	-6.142305
H	-1.229222	2.236040	-5.913488
H	-1.524162	0.911972	-7.029242
H	-1.309630	0.574215	-5.320050
C	1.123779	0.128818	-5.400231
H	1.886351	0.619334	-4.788749
H	0.388475	-0.314949	-4.717436
H	1.617892	-0.709504	-5.907105
C	0.844761	0.757069	-7.756013
H	1.395668	-0.190771	-7.807531
H	-0.070857	0.565020	-8.329951
C	1.664782	1.784205	-8.574903
H	2.538970	1.243995	-8.960318
H	1.073191	2.009304	-9.471558
C	3.561005	3.142227	-7.811670
H	3.890844	3.087186	-6.770488
H	4.075778	2.334875	-8.347350
H	3.944738	4.081390	-8.229640
C	1.494944	4.232693	-8.549203
H	2.250588	4.974562	-8.836408
H	0.940924	3.987982	-9.464103
H	0.788140	4.727962	-7.877494
N	0.247289	15.606552	10.196090
N	2.666870	14.376158	11.261536
C	0.199266	16.638401	9.125519
H	-0.800675	16.754602	8.689190
H	0.498964	17.635167	9.472780
H	0.882440	16.370995	8.314574
C	-1.098976	15.019611	10.429148
H	-1.864523	15.424237	9.755345
H	-1.065072	13.937367	10.276773
H	-1.469981	15.181214	11.449028
C	0.805592	16.176189	11.457341
H	0.059687	16.146478	12.261907
H	1.030138	17.244156	11.341116
C	2.085410	15.517173	12.027582
H	2.820831	16.322584	12.150584
H	1.845867	15.215019	13.055221
C	4.054928	14.684418	10.824324
H	4.782065	13.927504	11.144382
H	4.100458	14.741668	9.733432
H	4.424605	15.644593	11.205445
C	2.628184	13.122553	12.060033
H	3.612782	12.648997	12.161185
H	2.251648	13.271828	13.079636
H	1.963247	12.397499	11.581951
N	-9.375974	8.349285	3.358014
N	-9.257379	5.475411	2.869520
N	0.522341	1.109774	-6.342107
N	2.078832	3.039036	-7.881194
N	12.155055	9.915740	2.108331
N	12.084567	10.379612	-0.772773
N	-0.050536	2.673879	1.081251
N	-1.762381	3.292716	2.657086
N	-2.068147	3.737157	0.310595
N	-6.185661	5.712509	2.464447
N	1.862698	1.305502	5.548980
N	-0.891086	14.979883	-1.738766
N	-2.977857	12.125231	1.851249
N	-1.184930	13.251051	2.994265
N	-2.649328	11.772815	4.212958
N	0.389826	13.684073	7.762526
N	-6.294710	8.503475	2.964042

N	9.067885	9.524476	2.075097
N	5.580995	9.672703	5.726652
N	4.292126	8.048342	6.963898
N	5.870542	7.323807	5.290971
N	3.727310	3.029957	6.800029
N	0.954321	14.367185	-3.792141
N	4.587168	10.969477	-2.943562
N	4.870362	8.604208	-3.284643
N	2.849632	9.691263	-4.008716
N	2.180692	4.881962	-5.384644
N	9.008314	9.964990	-0.727893
Pd	3.210533	1.019559	7.129251
Pd	10.573854	9.952751	0.669255
Pd	-7.773496	7.006153	2.920145
Pd	1.350183	3.018869	-5.876824
Pd	-0.380427	15.961093	-3.531065
Pd	1.511740	14.035065	9.502269
C	-0.236679	5.484855	5.459082
C	0.902158	5.828368	4.673953
C	0.737731	5.874576	3.231745
C	-0.511383	6.357655	2.774392
C	-1.470568	6.705392	3.809285
C	-1.464422	5.919909	5.003627
H	2.716060	5.224903	2.664852
H	1.876643	5.552896	5.064248
C	1.754145	5.579972	2.310287
C	-0.710228	6.587107	1.403394
H	-2.421818	7.129716	3.500407
C	0.293607	6.262610	0.499819
C	1.518679	5.743028	0.951381
H	-1.646930	7.012136	1.058571
C	-0.218509	9.170097	3.344457
C	-0.451462	8.383842	4.592060
C	0.818653	7.944423	5.051595
C	1.842750	8.506506	4.122130
H	-1.272153	8.669664	5.237128
H	1.090273	7.834453	6.093315
C	1.741676	9.882728	1.963473
C	2.194022	11.279348	2.422582
C	2.879477	9.117189	1.278382
H	0.915168	10.000540	1.254687
C	2.752480	12.084539	1.241496
H	2.966691	11.159005	3.192651
H	1.347536	11.801884	2.882354
C	3.436635	9.928535	0.103121
H	3.673907	8.929583	2.004673
H	2.514254	8.144936	0.941020
C	3.891824	11.326027	0.546489
H	3.097445	13.064861	1.583244
H	1.943759	12.263192	0.517032
H	4.270528	9.372008	-0.337703
H	2.666608	10.024846	-0.675292
H	4.263171	11.903622	-0.306741
H	4.730952	11.223635	1.248019
N	1.154010	9.144844	3.087705
O	3.055866	8.428130	4.213653
O	-1.041244	9.707343	2.625078
H	2.295608	5.488185	0.239085
H	0.129621	6.419755	-0.561010
C	-1.126615	5.365736	7.614912
H	-1.009831	4.773698	8.522664
H	-1.040777	6.428549	7.863972
C	-2.465591	5.087955	6.954790
H	-3.291028	5.371612	7.608046
H	-2.554117	4.024052	6.705949
O	-0.044888	4.996097	6.727511
O	-2.606099	5.865447	5.745784

Table5_1d_TSi_TSi-ii_0wat

Property	Value
Charge	0
Electronic Energy, BS1 (a.u.)	-1208.096168
Thermal and entropic correction, BS1 (a.u.)	2.644499
Electronic Energy, BS2 (a.u.)	-1208.514910
Number of Imaginary Frequencies	0
Imaginary frequencies (cm-1)	None

Molecular Geometry in Cartesian Coordinates

C	-9.413973	8.827500	4.589148
H	-10.234823	8.576183	5.272383
H	-8.495430	8.458519	5.053651
H	-9.355875	9.922964	4.567196

C	-9.654221	9.284605	2.194039
H	-9.560087	10.298028	2.603846
H	-8.852361	9.151730	1.462554
H	-10.597611	9.266725	1.634128
C	-10.768055	7.336358	3.186513
H	-11.472689	7.667140	2.412775
H	-11.344117	7.403464	4.118403
C	-10.518035	5.828770	2.938915
H	-11.126594	5.552407	2.068362
H	-10.990199	5.296627	3.774705
C	-8.910080	4.749638	1.433554
H	-8.251012	5.363067	0.813173
H	-8.462773	3.750021	1.501111
H	-9.845517	4.628690	0.873042
C	-8.689900	4.472124	3.857948
H	-7.904298	4.939335	4.458610
H	-9.508119	4.226501	4.546422
H	-8.299575	3.514997	3.489933
N	2.547064	-1.072805	7.314739
N	4.562118	0.492993	8.729038
C	1.112043	-1.024298	7.700699
H	0.806158	0.012345	7.867165
H	0.448445	-1.445545	6.935098
H	0.896281	-1.568725	8.628495
C	2.718473	-1.717391	5.984923
H	3.221629	-1.032627	5.296557
H	3.327095	-2.629508	6.022457
H	1.765346	-2.005255	5.524163
C	3.356208	-1.775599	8.353257
H	2.706645	-2.213839	9.121811
H	3.882447	-2.638246	7.924873
C	4.420574	-0.943055	9.108767
H	5.372822	-1.476006	8.991606
H	4.188282	-1.042929	10.176831
C	5.937996	0.778800	8.241719
H	6.419065	1.604181	8.781026
H	6.614543	-0.080652	8.329435
H	5.912295	1.049809	7.182893
C	4.205302	1.381922	9.865971
H	3.311752	1.961611	9.618758
H	3.979089	0.832740	10.788491
H	5.001527	2.093199	10.118825
N	-1.682244	17.618022	-3.336402
N	0.215309	16.929384	-5.443911
C	-3.055043	17.053668	-3.246700
H	-3.509349	17.189516	-2.257332
H	-3.751996	17.497281	-3.968690
H	-3.031760	15.980358	-3.454421
C	-1.296073	18.283983	-2.064341
H	-2.070604	18.209097	-1.291006
H	-0.386545	17.826468	-1.664859
H	-1.084828	19.354024	-2.184060
C	-1.568087	18.559612	-4.488417
H	-1.281585	19.562877	-4.147747
H	-2.543379	18.708788	-4.969217
C	-0.579831	18.180328	-5.619163
H	-1.166096	18.145550	-6.546430
H	0.081098	19.046249	-5.753150
C	1.669034	17.231045	-5.358333
H	2.266291	16.668600	-6.086919
H	1.900635	18.290668	-5.524044
H	2.044182	16.980432	-4.362257
C	-0.063107	15.963598	-6.539811
H	0.844818	15.635143	-7.061081
H	-0.549659	15.071214	-6.135726
H	-0.731324	16.366671	-7.311039
C	12.266562	8.499168	2.641468
H	12.056394	8.500859	3.718323
H	11.521767	7.857798	2.162028
H	13.236484	8.001252	2.518836
C	11.956361	10.890083	3.078274
H	11.799506	10.461930	4.076298
H	12.767148	11.622141	3.181947
H	11.057576	11.454620	2.817197
C	13.494862	10.157593	1.313118
H	14.219574	9.342936	1.438699
H	13.998075	11.032083	1.745130
C	13.393396	10.420771	-0.209554
H	13.843369	11.406325	-0.384916
H	14.088966	9.720745	-0.689917
C	11.654678	11.632342	-1.448498
H	11.371141	11.536745	-2.504051
H	10.799715	12.060123	-0.917183
H	12.455366	12.381568	-1.412503
C	11.984058	9.234631	-1.832140
H	11.645573	9.571007	-2.820220
H	12.952963	8.744534	-1.989845
H	11.291847	8.458331	-1.493950

N	0.606346	3.044913	-3.912212
C	1.324936	2.537760	-2.889036
H	2.259760	2.055030	-3.137399
C	0.907005	2.606592	-1.544782
H	1.538316	2.163012	-0.785082
C	-0.322681	3.232638	-1.216122
C	-1.058618	3.768678	-2.303360
H	-2.009724	4.264296	-2.154708
C	-0.560459	3.653342	-3.615906
H	-1.126619	4.056248	-4.443957
C	-0.792903	3.318633	0.129110
C	-0.501131	2.916696	2.342931
C	0.304717	2.416191	3.409921
C	1.592237	1.867240	3.183909
H	2.013384	1.798637	2.189164
C	2.350231	1.380036	4.266484
H	3.327511	0.953150	4.089524
C	0.695628	1.927957	5.776881
H	0.355762	1.941416	6.802242
C	-0.134749	2.434460	4.757315
H	-1.106537	2.825826	5.029212
C	-2.382567	3.956560	1.617268
C	-3.608006	4.635631	1.894828
C	-4.115487	4.770152	3.212265
H	-3.594559	4.350395	4.063289
C	-4.386896	5.222269	0.865014
H	-4.086986	5.171314	-0.173956
C	-5.591969	5.879057	1.179370
H	-6.187072	6.318883	0.391213
C	-5.332716	5.446917	3.432000
H	-5.723348	5.543956	4.434676
C	-1.892917	14.123149	-1.792152
H	-2.406632	13.927990	-2.722763
C	-2.297153	13.461936	-0.616441
H	-3.129365	12.771816	-0.675422
C	-0.218455	15.254979	-0.675622
H	0.595052	15.965240	-0.719406
C	-0.549334	14.637033	0.547101
H	0.022634	14.897355	1.428293
C	-1.622077	13.710711	0.605682
C	-2.010265	13.074894	1.824097
C	-1.761342	12.793015	4.060463
C	-3.389066	11.671037	2.953057
C	-4.432474	10.696411	2.941244
C	-4.884968	10.063535	4.125945
H	-4.462274	10.307675	5.091646
C	-5.076252	10.302224	1.740997
H	-4.805146	10.730292	0.784374
C	-6.104961	9.341313	1.776408
H	-6.600037	9.050785	0.860571
C	-5.920921	9.110189	4.063058
H	-6.270263	8.637096	4.968955
C	-1.038866	13.054877	5.264192
C	0.077467	13.929421	5.293635
H	0.425080	14.436728	4.402893
C	0.745306	14.178407	6.508467
H	1.586471	14.856847	6.533444
C	-0.664090	12.763217	7.666129
H	-0.939186	12.311662	8.608284
C	-1.397172	12.460863	6.501163
H	-2.237227	11.782881	6.580853
C	8.803759	8.182030	2.412169
H	9.298400	7.405807	1.845746
C	8.527543	10.436377	2.822793
H	8.805731	11.453150	2.584143
C	7.563520	10.186487	3.820156
H	7.124532	11.029552	4.337611
C	7.198980	8.852681	4.135646
C	7.852230	7.837355	3.391703
H	7.644542	6.788304	3.559045
C	6.233783	8.547916	5.143508
C	4.743961	9.231384	6.711170
C	5.007232	7.020926	6.287679
N	2.724437	12.420625	8.747360
C	4.063987	10.291388	7.384739
C	4.361110	11.653663	7.127716
H	5.121864	11.943984	6.414606
C	3.675350	12.667859	7.823332
H	3.913641	13.704706	7.632528
C	3.055806	10.048014	8.351603
H	2.767205	9.042273	8.627423
C	2.422879	11.129534	8.997042
H	1.662717	10.940868	9.741797
C	4.625594	5.661407	6.504007
C	3.637164	5.295071	7.453132
H	3.141399	6.036601	8.066060
C	3.294250	3.938207	7.625376
H	2.543147	3.660232	8.350862

C	4.808407	3.268738	6.017357
H	5.263228	2.458351	5.465052
C	5.212345	4.595556	5.775093
H	5.982835	4.769698	5.034690
C	0.549193	13.190556	-4.335664
H	-0.421228	13.182981	-4.811910
C	2.198234	14.394923	-3.268954
H	2.543833	15.348161	-2.895957
C	3.014122	13.254703	-3.134346
H	3.981559	13.368475	-2.662533
C	1.293608	11.997803	-4.246618
H	0.869845	11.095499	-4.668247
C	2.568806	12.002363	-3.627761
C	3.359735	10.819180	-3.514264
C	5.252843	9.782712	-2.806189
C	3.641685	8.597353	-3.881422
C	6.491869	9.826569	-2.097365
C	6.981608	11.022068	-1.512153
H	6.441100	11.956382	-1.592099
C	8.206063	11.014687	-0.814827
H	8.580183	11.927837	-0.374217
C	8.511988	8.763957	-1.212305
H	9.126366	7.883742	-1.086854
C	7.302070	8.675443	-1.928369
H	7.020244	7.716521	-2.344057
C	3.154336	7.356791	-4.395213
C	3.909567	6.159237	-4.308423
H	4.896656	6.140939	-3.863431
C	3.384983	4.956035	-4.820591
H	3.964607	4.046173	-4.753910
C	1.884732	7.246501	-5.017533
H	1.239565	8.106253	-5.147807
C	1.444759	5.999219	-5.504038
H	0.480945	5.919006	-5.987423
C	-1.013407	1.252855	-6.131110
H	-1.262114	2.288991	-5.886830
H	-1.606741	0.987517	-7.015096
H	-1.384666	0.623406	-5.312794
C	1.035342	0.115659	-5.415983
H	1.818146	0.581683	-4.811056
H	0.295264	-0.309928	-4.726850
H	1.500472	-0.733915	-5.931444
C	0.747755	0.761068	-7.766200
H	1.235308	-0.220317	-7.828197
H	-0.178521	0.635604	-8.341486
C	1.636675	1.738872	-8.573326
H	2.478032	1.146564	-8.955429
H	1.067234	2.005207	-9.473044
C	3.607778	2.951332	-7.754002
H	3.899238	2.896233	-6.701387
H	4.074274	2.094031	-8.255235
H	4.076107	3.847920	-8.179295
C	1.654897	4.191831	-8.558661
H	2.473187	4.874116	-8.820627
H	1.118516	3.985042	-9.493195
H	0.962936	4.741947	-7.915329
N	0.214911	15.546124	10.103090
N	2.607980	14.303478	11.212974
C	0.182773	16.569552	9.024018
H	-0.808191	16.674954	8.565110
H	0.468511	17.570838	9.370151
H	0.884788	16.300685	8.229954
C	-1.136255	14.967099	10.326294
H	-1.892743	15.369558	9.641079
H	-1.105560	13.883471	10.184348
H	-1.517216	15.139366	11.340690
C	0.762034	16.123681	11.365499
H	0.002688	16.116239	12.157919
H	1.005828	17.186331	11.239266
C	2.020619	15.450499	11.965636
H	2.761502	16.247950	12.106188
H	1.753881	15.151106	12.987380
C	4.005944	14.601415	10.800571
H	4.722424	13.842300	11.138857
H	4.072959	14.652692	9.710569
H	4.374017	15.561553	11.183422
C	2.546057	13.052390	12.014138
H	3.525344	12.572254	12.134060
H	2.152318	13.206914	13.026482
H	1.884882	12.330589	11.526039
N	-9.572896	8.228060	3.237862
N	-9.102790	5.389117	2.762852
N	0.451940	1.117141	-6.347396
N	2.125279	2.960582	-7.868954
N	12.227121	9.859128	2.041375
N	12.039753	10.334470	-0.832589
N	0.016100	2.834521	1.096224
N	-1.696369	3.458962	2.671048

N	-1.987263	3.917043	0.325483
N	-6.065711	5.988130	2.436972
N	1.915954	1.402780	5.543208
N	-0.878554	15.010640	-1.826281
N	-3.043457	12.208314	1.761361
N	-1.300560	13.381361	2.932728
N	-2.824243	11.962434	4.148144
N	0.383120	13.613219	7.677685
N	-6.529723	8.752956	2.913254
N	9.145182	9.455943	2.132026
N	5.677685	9.590086	5.800275
N	4.369575	7.966139	7.016539
N	5.951683	7.242162	5.346766
N	3.870517	2.937268	6.927488
N	0.986162	14.375299	-3.860676
N	4.555194	10.937283	-2.894891
N	4.844004	8.577588	-3.264434
N	2.825046	9.669386	-3.985229
N	2.177023	4.869683	-5.414474
N	8.966358	9.910454	-0.665852
Pd	3.218321	0.945678	7.128902
Pd	10.589521	9.895806	0.667307
Pd	-7.813132	7.085896	2.841908
Pd	1.337389	3.001845	-5.885476
Pd	-0.342256	15.979825	-3.618896
Pd	1.480871	13.964652	9.433840
C	0.168462	5.490769	5.707067
C	1.191832	5.864730	4.784526
C	0.839544	5.995191	3.387798
C	-0.474144	6.450405	3.117998
C	-1.294669	6.733169	4.282328
C	-1.105413	5.917977	5.447052
H	2.777418	5.523472	2.521493
H	0.424449	5.037238	6.658831
H	2.227701	5.628697	5.007609
C	1.756061	5.835920	2.338123
C	-0.843358	6.769083	1.801660
H	-2.275619	7.163330	4.097398
H	-1.896332	5.808208	6.179946
C	0.059321	6.583658	0.767512
C	1.366272	6.107008	1.037368
H	-1.817581	7.186414	1.573244
C	-0.167507	9.214016	3.715959
C	-0.243817	8.405125	4.972310
C	1.080171	8.013782	5.284344
C	1.982372	8.587388	4.247751
H	-0.987197	8.676023	5.708966
H	1.466487	7.874965	6.283697
C	1.585741	9.951400	2.101559
C	1.860616	11.419392	2.469093
C	2.789460	9.335537	1.377886
H	0.714332	9.917917	1.437407
C	2.206634	12.241349	1.220529
H	2.697708	11.444787	3.178285
H	0.984195	11.833691	2.975898
C	3.120170	10.153329	0.123423
H	3.650846	9.332629	2.049090
H	2.592871	8.294441	1.119803
C	3.391909	11.624627	0.464789
H	2.429173	13.275073	1.502539
H	1.329381	12.267854	0.557474
H	3.989329	9.702334	-0.365913
H	2.285305	10.097842	-0.591272
H	3.609175	12.199966	-0.441012
H	4.287376	11.685497	1.095816
N	1.165232	9.204691	3.293842
O	3.199314	8.530116	4.203269
O	-1.074065	9.759754	3.113111
O	2.303177	5.974136	0.051760
O	-0.323532	6.880669	-0.514222
C	1.850890	6.184298	-1.284563
H	2.737343	6.470666	-1.853500
H	1.456212	5.247039	-1.693766
C	0.779368	7.257827	-1.341978
H	0.385636	7.373588	-2.352952
H	1.184612	8.219513	-1.004824

Table5_1d_TSi_DG_0wat

Property	Value
Charge	0
Electronic Energy, BS1 (a.u.)	-1208.145760
Thermal and entropic correction, BS1 (a.u.)	2.647505

Electronic Energy, BS2 (a.u.)	-1208.561771
Number of Imaginary Frequencies	0
Imaginary frequencies (cm-1)	None

Molecular Geometry in Cartesian Coordinates

C	8.617572	0.967806	6.754563
H	8.984498	0.546025	7.698692
H	7.750145	1.584936	7.001421
H	8.257191	0.118768	6.160293
C	9.942635	1.199470	4.707737
H	9.360604	0.294555	4.492296
H	9.717435	1.928523	3.923499
H	10.997543	0.922463	4.588773
C	10.876267	1.919922	6.862102
H	11.731290	1.452471	6.356966
H	10.786842	1.364914	7.804705
C	11.323224	3.353858	7.237690
H	12.362858	3.449576	6.898798
H	11.395052	3.382502	8.332618
C	11.259177	5.304649	5.749108
H	10.806134	5.221407	4.757034
H	11.285928	6.369358	6.013014
H	12.304071	4.986928	5.643844
C	9.994238	5.321648	7.847015
H	8.907570	5.236117	7.922527
H	10.401356	5.025238	8.821955
H	10.234141	6.385346	7.725531
N	3.086359	15.124362	10.560499
N	0.194797	15.380880	10.859084
C	3.858365	13.958311	11.065333
H	3.333767	13.030298	10.822039
H	4.864703	13.892516	10.633263
H	3.991845	13.967588	12.154413
C	3.904207	15.937842	9.621758
H	3.397442	16.019551	8.656796
H	4.072999	16.963231	9.974004
H	4.897443	15.509291	9.438534
C	2.586006	15.961320	11.690352
H	2.953074	15.581096	12.652305
H	2.992452	16.979213	11.633317
C	1.052046	16.113419	11.836473
H	0.842032	17.190471	11.812424
H	0.810096	15.817337	12.865313
C	-0.652780	16.325153	10.082815
H	-1.721976	16.085870	10.140896
H	-0.553137	17.366485	10.413751
H	-0.369405	16.302781	9.026418
C	-0.639698	14.358330	11.544469
H	-0.339423	13.356274	11.225716
H	-0.541691	14.383043	12.636918
H	-1.710337	14.465757	11.329184
N	2.462092	1.345053	-6.438769
N	2.366695	3.910970	-7.826801
C	3.709420	0.758972	-5.879768
H	3.517600	-0.052496	-5.166568
H	4.372756	0.342773	-6.648252
H	4.282007	1.528631	-5.354586
C	1.261520	0.645879	-5.908343
H	1.510402	-0.150485	-5.195814
H	0.613715	1.358717	-5.390037
H	0.652284	0.178316	-6.691967
C	2.481013	1.317506	-7.930731
H	1.644029	0.724944	-8.321732
H	3.373018	0.795770	-8.300483
C	2.434858	2.679328	-8.667259
H	3.310958	2.704435	-9.327829
H	1.582316	2.628734	-9.356614
C	1.110716	4.662599	-8.088598
H	1.288690	5.710735	-8.359703
H	0.511251	4.232794	-8.900781
H	0.479139	4.656674	-7.196128
C	3.558457	4.772657	-8.048042
H	3.296560	5.797609	-8.338997
H	4.151541	4.833049	-7.131310
H	4.227103	4.392342	-8.830373
C	-6.615744	15.865655	-0.905362
H	-7.232965	15.640677	-0.026470
H	-5.578393	15.939596	-0.567605
H	-6.910123	16.868595	-1.239133
C	-7.690207	13.770322	-1.581822
H	-8.106865	13.917748	-0.577516
H	-8.548073	13.691375	-2.261202
H	-7.190872	12.797083	-1.589304
C	-7.120388	15.456126	-3.272997
H	-7.341232	16.523601	-3.145212
H	-8.062208	15.030813	-3.642779
C	-6.106042	15.357813	-4.439364

H	-6.650371	14.918586	-5.285146
H	-5.900620	16.387333	-4.759693
C	-4.728617	13.425501	-5.066504
H	-3.794363	13.402364	-5.641408
H	-4.769467	12.510984	-4.468474
H	-5.542415	13.356976	-5.799326
C	-3.657619	15.508241	-4.347125
H	-2.915994	15.110342	-5.051036
H	-3.921220	16.508743	-4.712282
H	-3.156411	15.646695	-3.385044
N	8.151250	13.703045	0.116979
C	7.250945	14.249612	0.961135
H	6.955330	15.270223	0.763295
C	6.704839	13.566527	2.065714
H	6.001553	14.090405	2.699631
C	7.100899	12.233561	2.338186
C	8.045983	11.671904	1.444013
H	8.420755	10.664293	1.571019
C	8.532140	12.432928	0.364321
H	9.255207	11.999049	-0.311477
C	6.596993	11.504661	3.456628
C	5.333722	11.412591	5.337707
C	4.417744	12.042063	6.235970
C	3.945945	13.362043	6.017249
H	4.260184	13.944689	5.160963
C	3.057871	13.956134	6.933380
H	2.699956	14.961668	6.763957
C	3.049496	12.073611	8.267907
H	2.685871	11.585886	9.161406
C	3.935130	11.396785	7.403092
H	4.248227	10.394664	7.663606
C	6.586381	9.622013	4.723749
C	7.025984	8.277506	4.925494
C	6.624449	7.503565	6.043971
H	5.969072	7.906886	6.803538
C	7.899960	7.636986	4.010061
H	8.263733	8.137657	3.121698
C	8.324617	6.316469	4.244405
H	8.987610	5.831739	3.541888
C	7.097655	6.183082	6.193592
H	6.786035	5.589592	7.041864
C	3.476799	2.891213	-3.005205
H	4.404772	2.913032	-3.558475
C	3.504807	2.895210	-1.597781
H	4.469210	2.913416	-1.106381
C	1.175061	2.822835	-3.040207
H	0.265759	2.786758	-3.623362
C	1.102944	2.824843	-1.632536
H	0.127504	2.783490	-1.164825
C	2.292372	2.865085	-0.863318
C	2.274910	2.872539	0.564208
C	1.132440	2.929960	2.525198
C	3.400043	2.905170	2.532474
C	4.635389	2.913091	3.244848
C	4.682911	2.863759	4.657883
H	3.782026	2.796836	5.252878
C	5.888000	2.967084	2.584824
H	5.961153	2.987081	1.504912
C	7.078411	2.993442	3.337642
H	8.030597	3.035400	2.828094
C	5.923249	2.898077	5.322322
H	5.949014	2.870018	6.401957
C	-0.101576	3.034812	3.235735
C	-1.352705	3.036120	2.569188
H	-1.420786	2.945008	1.492762
C	-2.544943	3.142297	3.311517
H	-3.498443	3.138964	2.802159
C	-1.388655	3.264182	5.306615
H	-1.422547	3.364448	6.382404
C	-0.147833	3.157436	4.648250
H	0.754329	3.168581	5.244850
C	-4.127163	13.891113	0.756843
H	-3.921603	14.932620	0.555754
C	-5.055630	11.906729	0.054024
H	-5.593446	11.362338	-0.709394
C	-4.679974	11.250728	1.242222
H	-4.946142	10.208112	1.359703
C	-3.984636	11.960158	2.252688
C	-3.717061	13.324578	1.979056
H	-3.209562	13.957672	2.695406
C	-3.589933	11.341532	3.476939
C	-3.552454	9.527310	4.836214
C	-2.610777	11.473114	5.517512
N	-4.265084	5.354320	5.494981
C	-3.783623	8.135051	5.050984
C	-4.425507	7.321265	4.083763
H	-4.779924	7.729049	3.145556
C	-4.646099	5.955386	4.349174

H	-5.151956	5.339200	3.618932
C	-3.382472	7.484589	6.245582
H	-2.890069	8.023352	7.045028
C	-3.636210	6.109962	6.418418
H	-3.331404	5.618256	7.330936
C	-1.816304	12.183965	6.466948
C	-1.433467	11.604224	7.701946
H	-1.748134	10.607119	7.980805
C	-0.630019	12.329957	8.600988
H	-0.346573	11.884269	9.543045
C	-0.545805	14.145915	7.188639
H	-0.196196	15.151995	7.005892
C	-1.345099	13.498525	6.225332
H	-1.592455	14.029805	5.315607
C	3.403411	6.020119	-4.751081
H	4.343729	5.526013	-4.950514
C	1.097708	5.946703	-4.817033
H	0.202803	5.394739	-5.067730
C	0.992463	7.235583	-4.259920
H	0.003457	7.648916	-4.109377
C	3.395486	7.313101	-4.190100
H	4.343532	7.789189	-3.973612
C	2.163395	7.964074	-3.928519
C	2.102250	9.282191	-3.382378
C	0.871731	11.072696	-2.726383
C	3.136436	11.179081	-2.693358
C	-0.400160	11.702430	-2.569225
C	-1.611970	11.018994	-2.843681
H	-1.621745	9.986748	-3.169666
C	-2.843348	11.688132	-2.711042
H	-3.765653	11.166514	-2.924205
C	-1.803349	13.639778	-2.047443
H	-1.900352	14.669833	-1.734966
C	-0.526735	13.052092	-2.152138
H	0.341016	13.658093	-1.925803
C	4.330862	11.943466	-2.527030
C	4.301769	13.301266	-2.119981
H	3.371497	13.808651	-1.901539
C	5.500727	14.033914	-2.027980
H	5.472988	15.073755	-1.735197
C	5.613525	11.402441	-2.795669
H	5.740030	10.375671	-3.114773
C	6.759422	12.212342	-2.670647
H	7.734475	11.798834	-2.886167
C	11.223060	14.526218	-0.779975
H	10.804972	13.527667	-0.935532
H	12.167624	14.563665	-1.337021
H	11.489075	14.609186	0.281374
C	9.896962	16.466947	-0.090177
H	8.818967	16.440392	0.092600
H	10.395513	16.195128	0.848604
H	10.158374	17.514664	-0.284358
C	10.766371	16.330035	-2.381362
H	10.851502	17.396896	-2.138087
H	11.793817	16.027801	-2.621551
C	9.965431	16.246581	-3.704477
H	9.731940	17.279973	-3.991059
H	10.672379	15.909364	-4.473309
C	7.527842	16.221630	-3.997484
H	6.865596	16.232515	-3.127269
H	7.761500	17.268506	-4.228378
H	6.947270	15.841555	-4.847499
C	8.860764	14.282156	-4.677192
H	8.036571	14.248318	-5.400577
H	9.785172	14.323225	-5.266593
H	8.869060	13.326435	-4.145314
N	-4.498404	1.146871	5.904782
N	-6.235593	3.321459	6.775912
C	-4.467503	0.571375	4.533932
H	-3.559672	-0.011856	4.335457
H	-5.312334	-0.097331	4.326679
H	-4.514044	1.371568	3.791304
C	-3.309955	0.717411	6.687901
H	-2.623825	0.084712	6.111145
H	-2.748269	1.593802	7.022875
H	-3.569677	0.146985	7.588422
C	-5.762502	0.774177	6.605193
H	-5.551461	0.179220	7.502943
H	-6.375267	0.109561	5.982681
C	-6.691333	1.928187	7.054743
H	-7.671163	1.736217	6.599018
H	-6.863710	1.782517	8.128879
C	-7.217414	4.039292	5.919645
H	-7.558177	4.984346	6.360891
H	-6.769629	4.273194	4.949637
H	-8.121843	3.452620	5.715497
C	-5.991608	4.063143	8.041534
H	-6.541718	5.010994	8.094381

H	-6.271943	3.494070	8.936911
H	-4.927204	4.293973	8.139231
N	9.637760	1.782067	6.041403
N	10.494507	4.483837	6.724991
N	10.244744	15.558309	-1.215285
N	8.734746	15.402379	-3.707075
N	-6.729982	14.836596	-1.972913
N	-4.836197	14.616636	-4.182356
N	5.696415	12.126463	4.248735
N	5.727749	10.149850	5.630698
N	7.060082	10.249423	3.626027
N	7.941160	5.597943	5.317119
N	2.620447	13.333528	8.045230
N	2.337461	2.853576	-3.725827
N	3.472842	2.934571	1.185042
N	1.067594	2.876254	1.173967
N	2.264589	2.933520	3.264599
N	-2.569850	3.247764	4.655969
N	7.111253	2.965787	4.686799
N	-4.788413	13.205088	-0.198653
N	-3.898890	10.034346	3.632393
N	-2.970300	10.211263	5.842498
N	-2.889711	12.099253	4.351174
N	-0.187589	13.581169	8.361282
N	2.278349	5.345679	-5.067235
N	0.871009	9.799285	-3.175786
N	1.966601	11.808565	-2.434109
N	3.274112	9.912440	-3.147223
N	6.712757	13.509501	-2.302065
N	-2.943652	12.976922	-2.328933
Pd	1.429482	14.349921	9.454499
Pd	-4.816634	13.913723	-2.168885
Pd	8.788397	3.710450	5.701767
Pd	8.462423	14.539509	-1.778423
Pd	2.357307	3.365853	-5.765074
Pd	-4.389570	3.271004	5.707391
C	2.938858	5.738900	5.887011
C	3.262803	6.597083	4.684261
C	2.208136	6.384178	3.609509
C	0.894818	6.536528	4.090044
C	0.840255	6.856566	5.570323
C	1.668307	5.835303	6.311186
H	3.453916	6.137894	1.871404
H	4.280265	6.450846	4.318746
C	2.438982	6.209625	2.249444
C	-0.186540	6.481526	3.218254
H	-0.173873	6.935987	5.964218
C	0.040861	6.261851	1.854902
C	1.346338	6.137561	1.373154
H	-1.197570	6.618941	3.591903
C	3.037072	9.046192	3.970135
C	3.033257	8.083792	5.152857
C	1.622871	8.218918	5.760352
C	0.982117	9.356631	4.990510
H	3.812552	8.366658	5.861737
H	1.629056	8.461783	6.823709
C	1.480190	10.718984	2.913654
C	0.197528	10.317418	2.168896
C	1.389991	12.141670	3.479815
H	2.303651	10.673781	2.199351
C	-0.062985	11.311500	1.029320
H	-0.642794	10.313087	2.869702
H	0.306571	9.300578	1.782120
C	1.120072	13.134569	2.340094
H	0.585302	12.180624	4.223171
H	2.327750	12.389262	3.988891
C	-0.143670	12.750240	1.559377
H	-0.987598	11.042274	0.505720
H	0.757656	11.224721	0.306115
H	1.032804	14.151253	2.733595
H	1.979363	13.133863	1.656386
H	-0.302617	13.458042	0.739005
H	-1.008593	12.840719	2.229564
N	1.834281	9.740115	3.956644
O	-0.106946	9.846143	5.211163
O	3.928777	9.170857	3.145996
O	2.683847	9.212144	0.321055
H	3.363743	9.157126	1.010177
H	2.112257	8.459460	0.521966
O	1.136932	5.190433	7.385218
O	3.932769	5.081659	6.575886
C	2.076583	4.247530	7.941140
H	1.747486	4.035745	8.959065
H	2.048630	3.323373	7.350544
C	3.490676	4.821035	7.930445
H	4.192506	4.110097	8.367550
H	3.516545	5.756036	8.501110
H	-0.798952	6.188875	1.174661

H	1.515744	5.976255	0.312820

Table5_TSiv_TSi-ii_0wat

Property	Value
Charge	0
Electronic Energy, BS1 (a.u.)	-1208.098432
Thermal and entropic correction, BS1 (a.u.)	2.641322
Electronic Energy, BS2 (a.u.)	-1208.518577
Number of Imaginary Frequencies	0
Imaginary frequencies (cm-1)	None

Molecular Geometry in Cartesian Coordinates

C	-7.781722	2.453170	6.611850
C	-8.849241	2.591198	5.673674
C	-8.530125	3.237416	4.405016
C	-7.605392	4.304478	4.479189
C	-7.097121	4.600225	5.812622
C	-6.875634	3.490237	6.681151
H	-9.875611	2.131209	3.130085
H	-9.599137	1.805499	5.678366
C	-9.149375	2.937920	3.183428
C	-7.313310	5.055876	3.332889
H	-6.424714	5.443718	5.932404
C	-7.905902	4.726282	2.119004
C	-8.823381	3.666463	2.044247
H	-6.618783	5.889246	3.396821
C	-10.281322	3.840044	7.803026
C	-9.896027	4.278676	6.432754
C	-8.944443	5.326176	6.574072
C	-8.712286	5.511829	8.043315
H	-10.658489	4.254743	5.666068
H	-8.929349	6.210848	5.951252
C	-9.787820	4.555639	10.156645
C	-8.583243	4.307166	11.071654
C	-10.485885	5.881342	10.508677
H	-10.501912	3.735853	10.283411
C	-9.001692	4.369905	12.550991
H	-7.810573	5.054352	10.860867
H	-8.169060	3.317073	10.861071
C	-10.910116	5.921284	11.982135
H	-9.784750	6.698744	10.304983
H	-11.348299	6.000609	9.836437
C	-9.699365	5.691753	12.897389
H	-8.119419	4.227471	13.182271
H	-9.680698	3.534472	12.767851
H	-11.383087	6.884398	12.211735
H	-11.662686	5.144307	12.170026
H	-10.014698	5.690435	13.945227
H	-8.992035	6.522919	12.778275
N	-9.466966	4.536873	8.713336
O	-8.018215	6.352716	8.588185
O	-11.145362	3.044025	8.121997
C	-12.371442	-3.601870	7.489983
H	-12.292541	-4.259480	8.364658
H	-11.694158	-2.761564	7.666862
H	-13.395181	-3.206889	7.493158
C	-13.167796	-4.281591	5.274783
H	-14.046657	-3.761184	5.675434
H	-12.891962	-3.786108	4.339890
H	-13.504937	-5.291026	5.007843
C	-11.530708	-5.668272	6.465038
H	-12.147891	-6.402080	5.930996
H	-11.649992	-5.937838	7.522280
C	-10.059116	-5.982022	6.099918
H	-10.083259	-6.842388	5.418973
H	-9.593316	-6.377002	7.011932
C	-8.735218	-5.228040	4.175998
H	-9.174878	-4.567984	3.423112
H	-7.644907	-5.139319	4.092185
H	-8.983020	-6.254620	3.878060
C	-8.123670	-4.510007	6.436733
H	-8.262573	-3.490851	6.807062
H	-8.055167	-5.159795	7.318168
H	-7.143272	-4.552072	5.945816
N	0.185550	5.691229	6.634727
N	-0.845182	8.260868	7.549257
C	-0.113413	4.431299	7.364460
H	-1.173562	4.400144	7.629603
H	0.106530	3.532351	6.774864
H	0.449404	4.330895	8.300869
C	0.744763	5.404616	5.286497
H	0.115871	5.858844	4.515489

H	1.756223	5.805234	5.144204
H	0.806320	4.330969	5.069038
C	1.110779	6.558775	7.421229
H	1.466303	6.035632	8.318270
H	2.026902	6.770742	6.855214
C	0.564934	7.925436	7.903258
H	1.257476	8.689043	7.526627
H	0.708431	7.947565	8.991206
C	-0.907460	9.514446	6.750964
H	-1.583019	10.262807	7.183846
H	0.067188	10.007076	6.644075
H	-1.262001	9.298559	5.739293
C	-1.691019	8.363021	8.767573
H	-2.437064	7.563211	8.769578
H	-1.119849	8.262700	9.698997
H	-2.228521	9.317161	8.836492
N	-19.879456	3.638587	-1.640778
N	-19.192169	4.814371	-4.222586
C	-19.545772	2.238300	-1.267652
H	-19.617244	2.055039	-0.188213
H	-20.195365	1.496900	-1.749401
H	-18.522101	2.004400	-1.572919
C	-20.277985	4.426926	-0.444416
H	-20.217490	3.850034	0.486871
H	-19.624246	5.296578	-0.335286
H	-21.304372	4.810279	-0.502163
C	-20.947478	3.673931	-2.682324
H	-21.831070	4.213795	-2.318570
H	-21.319947	2.663644	-2.894799
C	-20.583756	4.302558	-4.050642
H	-20.817210	3.545573	-4.810274
H	-21.318132	5.097868	-4.231714
C	-19.193163	6.274637	-4.504668
H	-18.654675	6.532278	-5.425277
H	-20.201607	6.693828	-4.610642
H	-18.713007	6.813819	-3.682976
C	-18.478220	4.067568	-5.292558
H	-18.070009	4.722956	-6.072136
H	-17.641275	3.509275	-4.863733
H	-19.114223	3.336265	-5.806811
C	-7.432225	15.919964	0.194316
H	-7.401373	15.912262	1.291075
H	-7.030079	14.964154	-0.152314
H	-6.724723	16.694155	-0.127921
C	-9.784736	16.315726	0.755008
H	-9.331517	16.237627	1.751123
H	-10.283210	17.292396	0.719295
H	-10.572393	15.559827	0.691539
C	-8.838767	17.263761	-1.301601
H	-7.859406	17.755679	-1.360679
H	-9.513063	18.051980	-0.943076
C	-9.263961	16.956728	-2.758865
H	-10.116600	17.612483	-2.977270
H	-8.461309	17.339282	-3.402247
C	-10.998404	15.402314	-3.536478
H	-11.096321	14.924804	-4.519539
H	-11.554442	14.791970	-2.818906
H	-11.523363	16.363143	-3.607318
C	-8.653722	15.014546	-4.131257
H	-9.173731	14.608897	-5.008201
H	-7.957677	15.773335	-4.510080
H	-8.043641	14.209896	-3.711151
N	-5.652922	2.399669	-3.587444
C	-4.905892	3.203984	-2.803765
H	-4.294698	3.943311	-3.301668
C	-4.897757	3.118703	-1.397517
H	-4.262198	3.798097	-0.844331
C	-5.705974	2.155795	-0.741129
C	-6.493157	1.327493	-1.580829
H	-7.143307	0.562513	-1.176257
C	-6.435365	1.485207	-2.979235
H	-7.039267	0.856054	-3.617667
C	-5.719667	2.021495	0.680078
C	-4.938670	2.637028	2.718882
C	-4.118359	3.483471	3.523802
C	-3.322481	4.513872	2.964301
H	-3.292006	4.690485	1.896748
C	-2.534555	5.326723	3.802446
H	-1.920065	6.104647	3.371598
C	-3.247434	4.215181	5.691369
H	-3.214042	4.109722	6.766754
C	-4.050194	3.342722	4.931686
H	-4.601229	2.569218	5.448859
C	-6.449115	0.954223	2.544405
C	-7.280601	-0.030050	3.158851
C	-7.294941	-0.231261	4.561247
H	-6.656295	0.342398	5.218266
C	-8.141297	-0.864684	2.403383

H	-8.191913	-0.798671	1.324124
C	-8.943268	-1.821005	3.055522
H	-9.589560	-2.464832	2.475766
C	-8.146289	-1.199520	5.127954
H	-8.161685	-1.336252	6.200130
C	-16.290032	3.129182	-0.427954
H	-16.298582	2.385552	-1.212086
C	-15.565376	2.892435	0.756230
H	-15.034058	1.954707	0.855555
C	-16.975593	5.206319	0.312679
H	-17.529571	6.113187	0.116609
C	-16.280632	5.056871	1.528466
H	-16.324938	5.862887	2.249649
C	-15.550402	3.868612	1.784994
C	-14.851662	3.661798	3.012443
C	-14.302268	4.357848	5.102007
C	-13.616365	2.324097	4.367317
C	-12.945845	1.084755	4.596741
C	-12.318558	0.785490	5.831854
H	-12.334520	1.484638	6.656298
C	-12.886463	0.070599	3.607971
H	-13.346174	0.196131	2.635735
C	-12.238184	-1.147227	3.888371
H	-12.206079	-1.924158	3.137432
C	-11.682780	-0.457519	6.018984
H	-11.206930	-0.678946	6.963801
C	-14.393197	5.333239	6.140501
C	-15.048888	6.575134	5.946413
H	-15.500378	6.836463	4.997720
C	-15.131058	7.500696	7.004213
H	-15.636996	8.443988	6.854307
C	-13.986455	6.096634	8.429729
H	-13.589229	5.915399	9.417209
C	-13.851329	5.111827	7.431328
H	-13.358467	4.182631	7.684525
C	-7.947542	12.505413	0.608342
H	-7.134863	12.677829	-0.082999
C	-10.152406	12.887319	1.176251
H	-11.093114	13.362038	0.936301
C	-10.046829	12.082195	2.327129
H	-10.920704	11.963035	2.954734
C	-8.814950	11.453954	2.641162
C	-7.748820	11.683477	1.734555
H	-6.770965	11.244587	1.884954
C	-8.654650	10.645072	3.806347
C	-9.495061	9.777282	5.728556
C	-7.333301	9.390290	5.159708
N	-12.747954	9.399117	8.497358
C	-10.579781	9.637241	6.646274
C	-11.853916	10.203874	6.389628
H	-12.056913	10.759781	5.483097
C	-12.892220	10.057401	7.328963
H	-13.860020	10.496427	7.130928
C	-10.445145	8.940377	7.873359
H	-9.505127	8.492156	8.169278
C	-11.541538	8.854331	8.754530
H	-11.432894	8.346161	9.699568
C	-6.058079	8.815614	5.446093
C	-5.813258	8.075730	6.629743
H	-6.585023	7.930399	7.372891
C	-4.536494	7.530793	6.871541
H	-4.358997	6.962025	7.773032
C	-3.711054	8.395676	4.897552
H	-2.869073	8.519598	4.230971
C	-4.955276	8.965265	4.567773
H	-5.035116	9.524091	3.643875
C	-15.341301	4.570115	-3.611817
H	-15.605508	3.551455	-3.856276
C	-16.007822	6.639930	-2.854766
H	-16.804790	7.275082	-2.494998
C	-14.706627	7.162206	-2.984754
H	-14.538269	8.198022	-2.719376
C	-14.010928	5.001546	-3.775651
H	-13.280916	4.293972	-4.147363
C	-13.656686	6.336522	-3.458905
C	-12.322330	6.820470	-3.607423
C	-10.811825	8.502550	-3.417535
C	-10.155511	6.469197	-4.181311
C	-10.500711	9.847718	-3.055978
C	-11.488310	10.741815	-2.571531
H	-12.521593	10.439598	-2.458903
C	-11.134992	12.061060	-2.229086
H	-11.892076	12.739913	-1.863711
C	-8.928467	11.702769	-2.788453
H	-7.924870	12.095369	-2.868023
C	-9.186292	10.368331	-3.157635
H	-8.363499	9.764122	-3.517716
C	-9.126607	5.598386	-4.651097

C	-7.786881	6.034181	-4.806283
H	-7.492055	7.051773	-4.584053
C	-6.803018	5.134565	-5.259668
H	-5.783160	5.472412	-5.375879
C	-9.381296	4.243864	-4.981825
H	-10.372739	3.817038	-4.900293
C	-8.330135	3.419313	-5.427364
H	-8.530585	2.387390	-5.677289
C	-4.330531	-0.184799	-5.149648
H	-5.395373	-0.115646	-4.910331
H	-4.211899	-1.057441	-5.804033
H	-3.798188	-0.423055	-4.220314
C	-2.741656	1.674446	-5.003163
H	-2.998231	2.690088	-4.689619
H	-2.494929	1.102191	-4.100184
H	-1.812790	1.749789	-5.582434
C	-3.478128	0.863259	-7.198391
H	-2.423019	1.118668	-7.359926
H	-3.539006	-0.200582	-7.461202
C	-4.282865	1.626943	-8.279216
H	-3.548971	2.183918	-8.875595
H	-4.669323	0.868288	-8.971739
C	-5.110470	3.936868	-8.196276
H	-4.948330	4.544527	-7.301299
H	-4.216612	4.051414	-8.822004
H	-5.937617	4.391385	-8.755816
C	-6.696131	2.075134	-8.331910
H	-7.233363	2.862866	-8.875000
H	-6.620907	1.223870	-9.020046
H	-7.334710	1.754808	-7.503696
N	-16.156528	8.263093	10.728830
N	-14.226712	10.420719	11.026307
C	-17.289178	8.164906	9.768628
H	-17.668937	7.141209	9.659513
H	-18.151231	8.780610	10.054612
H	-16.976323	8.506112	8.777965
C	-15.863077	6.941125	11.342316
H	-16.498392	6.137518	10.949340
H	-14.821979	6.663077	11.150627
H	-15.996911	6.934363	12.431043
C	-16.438886	9.288944	11.774992
H	-16.536096	8.818933	12.762141
H	-17.413022	9.766555	11.608028
C	-15.406500	10.431488	11.939895
H	-15.963937	11.372948	11.853292
H	-15.084991	10.406501	12.989064
C	-14.143951	11.690655	10.255740
H	-13.167774	12.182900	10.348053
H	-14.314406	11.499522	9.192789
H	-14.891144	12.432290	10.565051
C	-12.969225	10.172192	11.780016
H	-12.211569	10.948618	11.614322
H	-13.122206	10.118243	12.865063
H	-12.533922	9.215408	11.474932
N	-12.015124	-4.279381	6.215296
N	-9.243227	-4.870993	5.528056
N	-3.867168	1.082762	-5.774442
N	-5.378117	2.525705	-7.810173
N	-8.803653	16.119210	-0.345073
N	-9.584468	15.538478	-3.096346
N	-4.928151	2.859476	1.385783
N	-5.674041	1.701969	3.361421
N	-6.525723	1.069353	1.200094
N	-8.954110	-1.992560	4.393594
N	-2.492265	5.189995	5.144271
N	-16.990519	4.260167	-0.647959
N	-14.196598	2.488617	3.158532
N	-14.919234	4.646957	3.935000
N	-13.606649	3.229605	5.371242
N	-14.615969	7.273159	8.229768
N	-11.647298	-1.416366	5.070487
N	-9.123074	13.105645	0.332654
N	-9.728308	10.505913	4.614534
N	-8.330427	9.165627	6.045655
N	-7.437401	10.095862	4.012562
N	-3.496147	7.691947	6.027448
N	-16.333006	5.366636	-3.161261
N	-12.095414	8.108084	-3.266143
N	-9.796095	7.735238	-3.872140
N	-11.397648	5.948237	-4.067051
N	-7.057807	3.846102	-5.568855
N	-9.880243	12.544917	-2.334811
Pd	-1.668093	6.703987	6.341211
Pd	-9.347601	14.322581	-1.363464
Pd	-10.459191	-3.134956	5.305698
Pd	-5.489279	2.463579	-5.683481
Pd	-18.099416	4.525790	-2.416736
Pd	-14.433627	8.838246	9.616261

H	-7.671265	5.301802	1.231018
H	-9.292023	3.425509	1.096509
C	-6.602761	1.338174	8.296448
H	-5.802051	0.878280	7.704893
H	-6.837674	0.694395	9.144820
C	-6.179414	2.719228	8.768780
H	-5.260356	2.663264	9.352096
H	-6.966893	3.173895	9.370172
O	-7.799462	1.409356	7.490788
O	-5.911668	3.584642	7.644083

Table5_1d_TSi_reactant_1wat

Property	Value
Charge	0
Electronic Energy, BS1 (a.u.)	-1284.590231
Thermal and entropic correction, BS1 (a.u.)	2.656968
Electronic Energy, BS2 (a.u.)	-1285.048466
Number of Imaginary Frequencies	0
Imaginary frequencies (cm-1)	None

Molecular Geometry in Cartesian Coordinates

C	8.540676	0.971399	6.787691
H	8.899372	0.531540	7.726745
H	7.715807	1.639435	7.048136
H	8.119865	0.143861	6.202840
C	9.824887	1.132426	4.708063
H	9.171265	0.276471	4.497771
H	9.639480	1.883766	3.935050
H	10.852980	0.777465	4.563633
C	10.858922	1.773500	6.841200
H	11.669113	1.258853	6.308820
H	10.759574	1.214925	7.780780
C	11.405997	3.172077	7.217128
H	12.430301	3.216991	6.825339
H	11.537046	3.171854	8.306743
C	11.397068	5.189440	5.819561
H	10.921597	5.171292	4.834733
H	11.474236	6.239102	6.129529
H	12.425611	4.834421	5.678922
C	10.173965	5.166274	7.941425
H	9.084537	5.142727	8.020869
H	10.567275	4.796077	8.896528
H	10.476885	6.218486	7.872377
N	3.143665	15.006581	10.437797
N	0.278198	15.338479	10.879320
C	3.903659	13.818338	10.907762
H	3.338230	12.907012	10.695918
H	4.884107	13.719770	10.425377
H	4.092503	13.826402	11.988569
C	3.939776	15.795314	9.460277
H	3.388926	15.893340	8.521295
H	4.157237	16.814649	9.803303
H	4.909004	15.336129	9.229221
C	2.722353	15.858123	11.588755
H	3.132242	15.473210	12.531351
H	3.146216	16.867060	11.505712
C	1.201860	16.041381	11.817056
H	1.014890	17.122916	11.813780
H	1.007382	15.739837	12.854323
C	-0.581305	16.309439	10.150895
H	-1.652480	16.098168	10.260506
H	-0.437377	17.345920	10.480841
H	-0.350804	16.284251	9.081867
C	-0.548713	14.333228	11.598592
H	-0.294852	13.326524	11.254617
H	-0.393669	14.343947	12.684714
H	-1.625506	14.472406	11.439633
N	2.491949	1.352299	-6.411930
N	2.416377	3.930996	-7.777103
C	3.738900	0.752481	-5.866772
H	3.546373	-0.063304	-5.158679
H	4.393791	0.337895	-6.643316
H	4.320606	1.513967	-5.339512
C	1.290209	0.657361	-5.878613
H	1.538522	-0.147889	-5.175882
H	0.651971	1.369884	-5.348177
H	0.671688	0.201919	-6.662043
C	2.500077	1.337479	-7.904172
H	1.652749	0.759331	-8.294484
H	3.382804	0.807570	-8.284441
C	2.467800	2.706197	-8.628624
H	3.342421	2.726859	-9.291324

H	1.612784	2.671676	-9.315918
C	1.168332	4.699684	-8.028207
H	1.357732	5.748282	-8.289823
H	0.561652	4.284550	-8.842708
H	0.539116	4.692869	-7.134042
C	3.617184	4.780874	-7.994140
H	3.366427	5.811397	-8.275095
H	4.213239	4.826006	-7.078413
H	4.279049	4.399735	-8.781778
C	-6.601205	15.848203	-0.850435
H	-7.202048	15.596866	0.032624
H	-5.558930	15.921748	-0.528166
H	-6.908386	16.856943	-1.153753
C	-7.676063	13.764692	-1.562375
H	-8.079340	13.885916	-0.549213
H	-8.542803	13.697005	-2.231555
H	-7.171891	12.794617	-1.600356
C	-7.140783	15.495441	-3.219276
H	-7.354372	16.560707	-3.063834
H	-8.091696	15.082030	-3.579282
C	-6.150778	15.418347	-4.407949
H	-6.712458	14.995181	-5.250555
H	-5.951178	16.453579	-4.713225
C	-4.789978	13.494301	-5.095340
H	-3.871251	13.482044	-5.694919
H	-4.812341	12.571214	-4.509473
H	-5.622743	13.434140	-5.807268
C	-3.700889	15.565070	-4.368562
H	-2.974890	15.176875	-5.093874
H	-3.971220	16.571087	-4.713157
H	-3.179120	15.688846	-3.415414
N	8.118451	13.681579	0.084976
C	7.203444	14.212919	0.922715
H	6.904933	15.234267	0.733197
C	6.646031	13.513516	2.010984
H	5.931759	14.025715	2.641906
C	7.049371	12.181343	2.276419
C	8.007874	11.634074	1.387597
H	8.387275	10.627544	1.509578
C	8.502925	12.410254	0.322370
H	9.237739	11.988701	-0.348526
C	6.544302	11.444283	3.388939
C	5.279276	11.348353	5.269181
C	4.338596	11.963323	6.152024
C	3.837001	13.269789	5.918316
H	4.142300	13.852050	5.058383
C	2.934377	13.853557	6.827540
H	2.557678	14.850854	6.649777
C	2.953818	11.980661	8.172171
H	2.588475	11.489927	9.062885
C	3.859225	11.315860	7.318857
H	4.179642	10.318617	7.579123
C	6.591841	9.587022	4.690990
C	7.090022	8.270003	4.936223
C	6.747402	7.530324	6.097096
H	6.100850	7.943774	6.859319
C	7.970695	7.626616	4.029094
H	8.294924	8.105019	3.113573
C	8.452607	6.333770	4.307529
H	9.121802	5.847991	3.611556
C	7.276672	6.237320	6.291065
H	7.015455	5.672588	7.174798
C	3.521782	2.865464	-2.965983
H	4.450539	2.853376	-3.518310
C	3.550215	2.899829	-1.558473
H	4.513679	2.908811	-1.064899
C	1.220139	2.846244	-3.000779
H	0.309857	2.815010	-3.582796
C	1.147541	2.884361	-1.594417
H	0.171120	2.872570	-1.127720
C	2.337156	2.917146	-0.825381
C	2.316140	2.958225	0.601092
C	1.159682	3.032303	2.553501
C	3.427884	2.985681	2.578301
C	4.661401	3.005054	3.295344
C	4.708888	2.974745	4.710043
H	3.807528	2.906046	5.305107
C	5.914282	3.056606	2.634413
H	5.988838	3.067416	1.554348
C	7.105089	3.093056	3.384901
H	8.057116	3.133367	2.874791
C	5.950199	3.019269	5.374393
H	5.979157	3.001205	6.454304
C	-0.085055	3.137251	3.245186
C	-1.322453	3.169724	2.553680
H	-1.372737	3.109070	1.474161
C	-2.528272	3.267664	3.272793
H	-3.471081	3.286183	2.744509

C	-1.414037	3.321358	5.293470
H	-1.470959	3.389889	6.370507
C	-0.159037	3.222380	4.659015
H	0.732374	3.203883	5.272323
C	-4.061633	13.876002	0.722808
H	-3.843966	14.915618	0.524255
C	-5.017151	11.904834	0.015702
H	-5.565538	11.370855	-0.747468
C	-4.643223	11.239460	1.199173
H	-4.924670	10.200910	1.315309
C	-3.936358	11.936977	2.210042
C	-3.653348	13.299421	1.941009
H	-3.135798	13.923979	2.657560
C	-3.546369	11.308834	3.430877
C	-3.521052	9.485928	4.778940
C	-2.586185	11.429886	5.482844
N	-4.390823	5.343299	5.465566
C	-3.806689	8.105337	5.003501
C	-4.457386	7.303245	4.032552
H	-4.779240	7.712329	3.083244
C	-4.726410	5.947359	4.306414
H	-5.238436	5.341813	3.571515
C	-3.455964	7.454769	6.213621
H	-2.968287	7.985396	7.021430
C	-3.761155	6.091749	6.394354
H	-3.500266	5.602273	7.321260
C	-1.844307	12.156333	6.462068
C	-1.470373	11.574692	7.698601
H	-1.758641	10.565245	7.959766
C	-0.694863	12.308592	8.614903
H	-0.410348	11.856336	9.553688
C	-0.640962	14.141748	7.228452
H	-0.317086	15.159452	7.063088
C	-1.411144	13.487625	6.246671
H	-1.660019	14.023461	5.340323
C	3.427580	6.029444	-4.704792
H	4.371922	5.550623	-4.920846
C	1.122437	5.920980	-4.731870
H	0.232429	5.356756	-4.972163
C	1.006612	7.205779	-4.166577
H	0.014913	7.604854	-3.996968
C	3.409323	7.320288	-4.140747
H	4.354299	7.809058	-3.940204
C	2.172046	7.952548	-3.857825
C	2.102756	9.273090	-3.318943
C	0.868901	11.070899	-2.692094
C	3.132312	11.185769	-2.669732
C	-0.400923	11.712922	-2.570298
C	-1.612148	11.039431	-2.869320
H	-1.623381	10.004834	-3.187467
C	-2.839862	11.721920	-2.774791
H	-3.761005	11.207228	-3.008373
C	-1.797695	13.669768	-2.105153
H	-1.891009	14.704683	-1.807997
C	-0.524926	13.068564	-2.172205
H	0.342361	13.669402	-1.931308
C	4.325041	11.957024	-2.527817
C	4.295632	13.318406	-2.134722
H	3.365851	13.823919	-1.909163
C	5.493101	14.055987	-2.064707
H	5.463938	15.098170	-1.780697
C	5.606077	11.418889	-2.809036
H	5.733093	10.389873	-3.120552
C	6.749852	12.234447	-2.707385
H	7.723415	11.822614	-2.932715
C	11.190886	14.554606	-0.736821
H	10.787072	13.552655	-0.907510
H	12.148190	14.604196	-1.270697
H	11.430556	14.634752	0.330938
C	9.831459	16.479992	-0.069382
H	8.750274	16.439075	0.091144
H	10.314037	16.211022	0.878529
H	10.083968	17.531384	-0.255179
C	10.752600	16.359493	-2.342063
H	10.826276	17.425662	-2.092074
H	11.786366	16.064869	-2.563706
C	9.978049	16.277035	-3.681015
H	9.748386	17.310738	-3.969659
H	10.699932	15.942782	-4.437133
C	7.544863	16.253919	-4.006019
H	6.872403	16.256470	-3.143695
H	7.781208	17.302948	-4.224131
H	6.974550	15.881998	-4.866530
C	8.887603	14.322561	-4.689620
H	8.074235	14.297673	-5.425556
H	9.820666	14.369095	-5.264832
H	8.886912	13.360887	-4.168394
N	-4.439404	1.120426	5.778113

N	-6.297771	3.194030	6.644811
C	-4.323193	0.574916	4.398946
H	-3.379536	0.041898	4.228583
H	-5.123749	-0.131447	4.145587
H	-4.380520	1.385003	3.667585
C	-3.265905	0.731065	6.603881
H	-2.519935	0.154879	6.042238
H	-2.770014	1.625320	6.991452
H	-3.534778	0.119536	7.474284
C	-5.712256	0.674432	6.417209
H	-5.510336	0.066748	7.308610
H	-6.269159	-0.000502	5.754790
C	-6.708498	1.775218	6.856860
H	-7.657264	1.559749	6.348681
H	-6.924615	1.588198	7.916603
C	-7.283692	3.908412	5.790174
H	-7.663036	4.827886	6.253165
H	-6.823393	4.187083	4.838095
H	-8.165348	3.301539	5.548611
C	-6.113918	3.895118	7.942979
H	-6.714251	4.810004	8.023873
H	-6.378450	3.275862	8.809261
H	-5.064761	4.177222	8.068046
N	9.593159	1.723080	6.052999
N	10.616337	4.358492	6.774104
N	10.212779	15.579364	-1.189867
N	8.748255	15.431987	-3.708868
N	-6.727114	14.845318	-1.941460
N	-4.876513	14.672000	-4.191289
N	5.628833	12.056104	4.172270
N	5.721826	10.108919	5.590122
N	7.030112	10.198782	3.569884
N	8.121337	5.647799	5.419098
N	2.503164	13.231603	7.942563
N	2.381972	2.837760	-3.687560
N	3.509559	3.006870	1.230528
N	1.106953	2.999267	1.202253
N	2.286243	3.017001	3.303380
N	-2.581973	3.334755	4.617918
N	7.136141	3.076922	4.733386
N	-4.736639	13.201317	-0.231075
N	-3.848163	9.997893	3.571818
N	-2.908549	10.150640	5.779982
N	-2.866462	12.065158	4.321585
N	-0.274080	13.571301	8.395999
N	2.308157	5.339631	-5.005349
N	0.869137	9.786942	-3.113197
N	1.962272	11.811376	-2.404432
N	3.272604	9.916739	-3.112294
N	6.704279	13.534469	-2.349408
N	-2.937533	13.015500	-2.407970
Pd	1.413242	14.284100	9.411325
Pd	-4.811779	13.937612	-2.191025
Pd	8.860739	3.705760	5.752034
Pd	8.452663	14.546698	-1.793937
Pd	2.407751	3.367669	-5.720661
Pd	-4.425009	3.251716	5.625688
C	2.816628	5.663911	5.866441
C	3.012441	6.015129	4.546519
C	1.902589	6.301263	3.702276
C	0.643044	6.566276	4.316431
C	0.574159	6.543428	5.730423
C	1.544773	5.895554	6.465237
H	2.956576	6.098902	1.822944
H	4.021353	6.005616	4.136993
C	1.988556	6.268959	2.284204
C	-0.499877	6.750662	3.497121
H	-0.318510	6.882724	6.249660
C	-0.407594	6.614693	2.128531
C	0.849478	6.380419	1.518340
H	-1.459231	6.946305	3.968335
C	3.058568	9.003218	3.910353
C	3.123636	8.397221	5.266261
C	2.020348	8.662897	5.927674
C	1.184596	9.560818	5.100646
H	4.084600	7.931809	5.633497
H	1.758195	8.488417	6.985709
C	1.461274	10.602075	2.800178
C	0.042701	10.302302	2.293987
C	1.629955	12.073572	3.208984
H	2.154922	10.397499	1.979987
C	-0.301409	11.228479	1.119528
H	-0.661343	10.446093	3.115213
H	-0.021578	9.253031	1.993504
C	1.274292	12.991432	2.035332
H	0.978431	12.281404	4.070862
H	2.665403	12.238174	3.529733
C	-0.141827	12.702514	1.521546

H	-1.319710	11.027742	0.769015
H	0.382233	10.997772	0.292809
H	1.369108	14.040520	2.328296
H	1.993114	12.823924	1.221658
H	-0.376339	13.365018	0.677937
H	-0.858818	12.940908	2.318560
N	1.851891	9.701566	3.875026
O	0.174786	10.156771	5.420055
O	3.892784	8.972262	3.011464
O	2.539181	9.049563	0.169995
H	3.251550	8.980041	0.824154
H	1.848296	8.499602	0.560043
O	1.295523	5.617449	7.763748
O	3.888797	5.262677	6.618867
C	2.318092	4.851232	8.431971
H	2.131542	4.958054	9.501649
H	2.222688	3.800802	8.151964
C	3.681724	5.387545	8.050953
H	4.475561	4.811706	8.530232
H	3.773850	6.438777	8.335708
H	-1.295604	6.698567	1.513617
H	0.909316	6.281185	0.439730

Table5_1d_TSi_reactant_2wat

Property	Value
Charge	0
Electronic Energy, BS1 (a.u.)	-1361.039395
Thermal and entropic correction, BS1 (a.u.)	2.791547
Electronic Energy, BS2 (a.u.)	-1361.534255
Number of Imaginary Frequencies	0
Imaginary frequencies (cm-1)	None

Molecular Geometry in Cartesian Coordinates

C	8.153923	1.050560	6.706913
H	8.388114	0.509149	7.632280
H	7.403781	1.808747	6.953021
H	7.685709	0.325483	6.029176
C	9.583391	1.285557	4.702200
H	8.870235	0.523411	4.362759
H	9.486680	2.151988	4.041080
H	10.586496	0.867986	4.548277
C	10.556142	1.600888	6.957489
H	11.365943	1.097420	6.413133
H	10.364730	0.955565	7.824844
C	11.139435	2.937943	7.502910
H	12.194289	2.939531	7.200064
H	11.188464	2.811867	8.591867
C	11.427854	5.089715	6.336915
H	10.988565	5.268502	5.351143
H	11.639621	6.069039	6.783937
H	12.400538	4.611844	6.165747
C	10.112624	4.987208	8.397453
H	9.023774	5.072032	8.448516
H	10.430274	4.480532	9.317223
H	10.525745	6.002338	8.449131
N	2.522360	15.737596	11.030760
N	-0.398192	15.214409	10.604373
C	3.397119	14.799606	11.783838
H	3.248931	13.779451	11.419122
H	4.464422	15.032826	11.682262
H	3.188205	14.782294	12.860559
C	3.331772	16.791430	10.362368
H	3.158069	16.764515	9.282434
H	3.084861	17.806518	10.697123
H	4.410307	16.670565	10.522947
C	1.495282	16.356890	11.944476
H	1.705776	16.079971	12.985151
H	1.595015	17.449587	11.945363
C	-0.022335	16.073494	11.747462
H	-0.524952	17.046790	11.674232
H	-0.393524	15.640147	12.685599
C	-1.216534	15.910428	9.584389
H	-2.139444	15.365662	9.348129
H	-1.517856	16.917008	9.902399
H	-0.646675	16.020132	8.656824
C	-0.952048	13.901279	11.006517
H	-0.267237	13.098745	10.714345
H	-1.102054	13.824433	12.091015
H	-1.924002	13.699952	10.538977
N	2.303704	1.318224	-5.980200
N	2.353252	3.519244	-7.881658
C	3.417114	0.799635	-5.140607

H	3.066759	0.197740	-4.293009
H	4.117220	0.164395	-5.697594
H	4.002628	1.628801	-4.734865
C	0.992832	0.807019	-5.501515
H	1.079625	0.177425	-4.607095
H	0.337013	1.646922	-5.253371
H	0.464630	0.206442	-6.252722
C	2.521034	0.971998	-7.414620
H	1.745384	0.280303	-7.767804
H	3.458428	0.417634	-7.550637
C	2.550983	2.143785	-8.425291
H	3.501424	2.065662	-8.968502
H	1.790157	1.915264	-9.182571
C	1.066533	4.097657	-8.349810
H	1.188462	5.071850	-8.839838
H	0.539892	3.453819	-9.065369
H	0.392606	4.237710	-7.499174
C	3.497392	4.397103	-8.248199
H	3.182925	5.314623	-8.761016
H	4.048843	4.694603	-7.352298
H	4.221207	3.908303	-8.912162
C	-6.790254	15.877430	-0.414386
H	-7.310256	15.521629	0.484173
H	-5.717480	15.884955	-0.196763
H	-7.100760	16.919146	-0.566088
C	-7.879905	13.830611	-1.279628
H	-8.209841	13.817169	-0.232934
H	-8.785437	13.764154	-1.896283
H	-7.295962	12.921621	-1.456936
C	-7.553478	15.783695	-2.770136
H	-7.738183	16.830970	-2.496787
H	-8.533488	15.402864	-3.086828
C	-6.639263	15.795873	-4.030751
H	-7.299018	15.527457	-4.865810
H	-6.401129	16.850298	-4.219514
C	-5.548704	13.828257	-5.037165
H	-4.769100	13.806785	-5.808912
H	-5.505388	12.877501	-4.498104
H	-6.509081	13.844842	-5.567041
C	-4.214743	15.792972	-4.441918
H	-3.662282	15.404571	-5.306595
H	-4.480266	16.828429	-4.689210
H	-3.515625	15.839342	-3.601823
N	8.252382	13.905391	0.262718
C	7.617414	14.587824	1.240055
H	7.364956	15.617428	1.032016
C	7.291187	14.030497	2.493761
H	6.797084	14.658802	3.228836
C	7.616836	12.676120	2.765675
C	8.283907	11.974438	1.730813
H	8.582371	10.939830	1.844025
C	8.578825	12.623045	0.518209
H	9.097507	12.089273	-0.265351
C	7.280164	12.034936	3.995082
C	6.192780	12.045312	5.983708
C	5.295110	12.694038	6.883994
C	4.831995	14.014264	6.657782
H	5.175424	14.601462	5.815892
C	3.890438	14.589093	7.532620
H	3.532403	15.591816	7.348223
C	3.841077	12.704771	8.851323
H	3.447822	12.202099	9.723609
C	4.781191	12.046313	8.034142
H	5.090770	11.045785	8.306878
C	7.228778	10.156415	5.267967
C	7.584596	8.785586	5.452750
C	7.205156	8.057088	6.608400
H	6.637697	8.519023	7.406255
C	8.338421	8.071999	4.485448
H	8.681631	8.541952	3.572321
C	8.670639	6.721516	4.706477
H	9.253499	6.181271	3.973362
C	7.570008	6.702551	6.736928
H	7.269648	6.145485	7.613054
C	3.396641	3.474674	-2.924415
H	4.295871	3.687720	-3.484341
C	3.455881	3.315742	-1.524703
H	4.416935	3.407743	-1.036076
C	1.119235	3.110840	-2.952945
H	0.205727	3.064645	-3.527137
C	1.080654	2.943406	-1.552655
H	0.125331	2.744145	-1.085341
C	2.270895	3.057618	-0.789048
C	2.267361	2.956725	0.636670
C	1.100146	2.933852	2.582035
C	3.362365	3.106034	2.622334
C	4.564379	3.279489	3.373229
C	4.551602	3.393865	4.787688

H	3.633150	3.329845	5.356125
C	5.834747	3.377779	2.751874
H	5.944806	3.288969	1.679328
C	6.986174	3.592518	3.536501
H	7.954734	3.671747	3.064024
C	5.752993	3.610324	5.489081
H	5.744852	3.702576	6.565856
C	-0.144977	2.969612	3.277498
C	-1.385855	2.866346	2.601699
H	-1.432211	2.722587	1.530617
C	-2.590312	2.910078	3.332918
H	-3.535825	2.824306	2.819381
C	-1.463037	3.142285	5.331626
H	-1.511624	3.240173	6.406648
C	-0.212744	3.117166	4.686243
H	0.678820	3.199155	5.294327
C	-3.768263	13.926580	0.612787
H	-3.535701	14.957392	0.384968
C	-4.869572	11.992178	0.011073
H	-5.510354	11.484151	-0.695587
C	-4.406805	11.314085	1.156075
H	-4.711224	10.286220	1.303193
C	-3.561223	11.978465	2.080495
C	-3.256909	13.331008	1.782878
H	-2.633201	13.928841	2.434853
C	-3.038658	11.317826	3.233953
C	-2.856807	9.448569	4.510362
C	-1.755046	11.341253	5.108221
N	-4.023435	5.412288	5.347485
C	-3.236986	8.100239	4.785295
C	-4.129058	7.386788	3.944702
H	-4.559912	7.840000	3.061626
C	-4.481180	6.058974	4.256209
H	-5.155497	5.517214	3.609900
C	-2.758896	7.400751	5.922159
H	-2.090578	7.865875	6.634587
C	-3.176637	6.076770	6.159846
H	-2.825181	5.548387	7.034587
C	-0.932034	12.050117	6.036565
C	-0.413747	11.432287	7.203366
H	-0.615597	10.394113	7.432984
C	0.364472	12.178854	8.110551
H	0.748115	11.712376	9.007028
C	0.188977	14.082425	6.819475
H	0.436734	15.126833	6.687603
C	-0.603134	13.418806	5.860627
H	-0.957152	13.982415	5.006729
C	3.281026	6.245112	-5.170448
H	4.227849	5.730442	-5.246998
C	0.998800	6.192689	-5.462651
H	0.122228	5.633927	-5.754707
C	0.861658	7.528228	-5.025118
H	-0.128268	7.963257	-4.998990
C	3.243032	7.577559	-4.713990
H	4.177076	8.051818	-4.443413
C	2.002489	8.256833	-4.602973
C	1.909505	9.579641	-4.069078
C	0.669804	11.357459	-3.386681
C	2.928849	11.394888	-3.157019
C	-0.594329	11.992894	-3.184570
C	-1.821270	11.363459	-3.515375
H	-1.847069	10.382027	-3.969675
C	-3.042847	12.023968	-3.264012
H	-3.977592	11.540233	-3.508333
C	-1.960624	13.876816	-2.417172
H	-2.034251	14.867485	-1.990767
C	-0.696874	13.294187	-2.627743
H	0.178823	13.869357	-2.357273
C	4.113025	12.069710	-2.728690
C	4.072452	13.370898	-2.164819
H	3.139269	13.902909	-2.031306
C	5.267581	14.012642	-1.783997
H	5.238187	15.008123	-1.363911
C	5.400037	11.488688	-2.855765
H	5.537009	10.503791	-3.283094
C	6.540545	12.207903	-2.444879
H	7.523687	11.772413	-2.555101
C	11.075368	14.811563	-0.979591
H	10.677916	13.793263	-0.940628
H	11.983153	14.773147	-1.594700
H	11.404155	15.074031	0.033558
C	9.769159	16.835182	-0.543171
H	8.709449	16.826627	-0.270061
H	10.349291	16.743904	0.383570
H	9.980690	17.832942	-0.947904
C	10.490409	16.306674	-2.836643
H	10.776223	17.358155	-2.704371
H	11.419791	15.827179	-3.169270

C	9.534872	16.303545	-4.065213
H	9.326629	17.352664	-4.313005
H	10.110295	15.925714	-4.920663
C	7.068704	16.412322	-3.886866
H	6.603638	16.363444	-2.897247
H	7.303801	17.465832	-4.085720
H	6.316985	16.110653	-4.627325
C	8.150638	14.416408	-4.872265
H	7.211103	14.445736	-5.438769
H	8.962958	14.400425	-5.610277
H	8.185886	13.465684	-4.331574
N	-4.863556	1.313215	5.986942
N	-6.287541	3.758578	6.691032
C	-5.028732	0.684897	4.649504
H	-4.252871	-0.058245	4.426622
H	-5.990155	0.169809	4.531946
H	-4.985957	1.450331	3.869441
C	-3.693599	0.737848	6.703152
H	-3.147847	-0.002196	6.104596
H	-2.988477	1.530838	6.967216
H	-3.965419	0.234088	7.639234
C	-6.106278	1.170742	6.801263
H	-5.902101	0.642511	7.741317
H	-6.836272	0.530974	6.288770
C	-6.859225	2.469885	7.180746
H	-7.889029	2.340608	6.823777
H	-6.953617	2.470933	8.274381
C	-7.204616	4.413895	5.721538
H	-7.444429	5.449464	5.993215
H	-6.745722	4.429677	4.729187
H	-8.164367	3.893108	5.613970
C	-5.977814	4.666523	7.827981
H	-6.453831	5.650160	7.729623
H	-6.298192	4.267439	8.798607
H	-4.898388	4.829149	7.896936
N	9.348605	1.689894	6.107734
N	10.505338	4.262356	7.159210
N	10.045251	15.743156	-1.512415
N	8.270969	15.549195	-3.925197
N	-7.065967	15.027058	-1.595914
N	-5.389904	14.955121	-4.081203
N	6.570944	12.750545	4.894318
N	6.488533	10.747836	6.232809
N	7.615065	10.731989	4.109856
N	8.294686	6.042488	5.808210
N	3.388956	13.954274	8.611805
N	2.252934	3.379692	-3.633334
N	3.457088	3.095132	1.271243
N	1.057272	2.847110	1.233591
N	2.214834	3.058871	3.331098
N	-2.635695	3.037387	4.675260
N	6.949816	3.709967	4.879844
N	-4.557916	13.274092	-0.264108
N	-3.374846	10.018401	3.399649
N	-1.996585	10.036891	5.372497
N	-2.216128	12.028772	4.039199
N	0.663725	13.480395	7.928894
N	2.184133	5.549500	-5.534479
N	0.669949	10.109952	-3.917306
N	1.760032	12.043541	-2.978102
N	3.070758	10.154571	-3.674242
N	6.484350	13.449665	-1.921577
N	-3.117720	13.259720	-2.726121
Pd	1.583306	14.595801	9.453594
Pd	-4.962518	14.092239	-2.150536
Pd	8.705315	3.987835	5.999635
Pd	8.225958	14.601721	-1.716113
Pd	2.273813	3.444817	-5.751131
Pd	-4.453313	3.381541	5.668864
C	4.863960	6.666601	3.719554
C	4.172389	6.426518	2.554678
C	2.754219	6.396730	2.551681
C	2.046667	6.625807	3.778029
C	2.792946	6.833923	4.968252
C	4.170147	6.850520	4.938714
H	2.525396	5.942708	0.437564
H	5.946441	6.708046	3.700320
H	4.705457	6.257567	1.625333
C	2.014904	6.147465	1.371447
C	0.626996	6.654348	3.755983
H	2.262375	6.969986	5.904204
H	4.737201	6.966241	5.854538
C	-0.070090	6.434375	2.589158
C	0.639821	6.140129	1.386341
H	0.069765	6.860205	4.664511
C	2.712816	8.935238	-0.600125
C	3.415425	7.784143	-1.250084
C	4.647452	7.714265	-0.735919

C	4.794160	8.776208	0.316314
H	2.890703	7.138173	-1.948318
H	5.449486	7.022921	-0.954465
C	3.159788	10.512915	1.288851
C	2.681300	9.800768	2.564390
C	4.255611	11.543508	1.566715
H	2.305461	11.017887	0.828586
C	2.270557	10.808091	3.645638
H	3.494792	9.173717	2.945860
H	1.851399	9.132646	2.314255
C	3.855325	12.517013	2.685816
H	5.164713	11.016032	1.865337
H	4.487210	12.087407	0.642855
C	3.436735	11.760157	3.951369
H	1.967713	10.264886	4.547534
H	1.400203	11.387102	3.309490
H	4.727488	13.152003	2.846808
H	3.036852	13.167048	2.360208
H	3.169326	12.459342	4.751517
H	4.269650	11.137294	4.305619
N	3.597871	9.523202	0.287570
O	5.732038	8.945809	1.066374
O	1.574502	9.340833	-0.803520
O	1.073765	6.043669	-2.307907
H	0.902199	5.670099	-1.431059
H	0.420856	6.770733	-2.305288
O	-0.721717	8.034316	-1.614181
H	-1.154472	8.575964	-2.284246
H	0.055335	8.549252	-1.317584
O	-0.010685	5.834869	0.228127
O	-1.439705	6.475120	2.587823
C	-1.443608	5.718552	0.289533
H	-1.805300	5.923102	-0.716977
H	-1.709910	4.696159	0.584189
C	-1.988268	6.725179	1.277663
H	-3.070669	6.634588	1.368765
H	-1.729729	7.736566	0.944395
